# Megafauna of the German exploration licence area for seafloor massive sulphides along the Central and South East Indian Ridge (Indian Ocean)

**DOI:** 10.3897/BDJ.9.e69955

**Published:** 2021-09-28

**Authors:** Klaas Gerdes, Terue Cristina Kihara, Pedro Martínez Arbizu, Thomas Kuhn, Ulrich Schwarz-Schampera, Christopher L Mah, Jon L Norenburg, Thomas D Linley, Kate Shalaeva, Enrique Macpherson, Dennis Gordon, Sabine Stöhr, Charles G Messing, Simon Bober, Theresa Guggolz, Magdalini Christodoulou, Andrey Gebruk, Antonina Kremenetskaia, Andreas Kroh, Karen Sanamyan, Kathrin Bolstad, Leon Hoffman, Andrew J Gooday, Tina Molodtsova

**Affiliations:** 1 INES – Integrated Environmental Solutions, Wilhelmshaven, Germany INES – Integrated Environmental Solutions Wilhelmshaven Germany; 2 Senckenberg am Meer, German Centre for Marine Biodiversity Research, Wilhelmshaven, Germany Senckenberg am Meer, German Centre for Marine Biodiversity Research Wilhelmshaven Germany; 3 Federal Institute for Geosciences and Natural Resources, Hannover, Germany Federal Institute for Geosciences and Natural Resources Hannover Germany; 4 International Seabed Authority, Kingston, Jamaica International Seabed Authority Kingston Jamaica; 5 Smithsonian Institution National Museum of Natural History, Washington, DC, United States of America Smithsonian Institution National Museum of Natural History Washington, DC United States of America; 6 Newcastle University, School of Natural and Environmental Sciences, Newcastle, United Kingdom Newcastle University, School of Natural and Environmental Sciences Newcastle United Kingdom; 7 Natural History Museum London, London, United Kingdom Natural History Museum London London United Kingdom; 8 Centro de Estudios Avanzados de Blanes (CEAB), Blanes, Girona, Spain Centro de Estudios Avanzados de Blanes (CEAB) Blanes, Girona Spain; 9 NIWA, Newmarket, Auckland, New Zealand NIWA Newmarket, Auckland New Zealand; 10 Swedish Museum of Natural History, Stockholm, Sweden Swedish Museum of Natural History Stockholm Sweden; 11 Department of Marine and Environmental Sciences, Nova Southeastern University, Dania Beach, United States of America Department of Marine and Environmental Sciences, Nova Southeastern University Dania Beach United States of America; 12 University of Hamburg, Hamburg, Germany University of Hamburg Hamburg Germany; 13 P.P. Shirshov Institute of Oceanology, Moscow, Russia P.P. Shirshov Institute of Oceanology Moscow Russia; 14 Naturhistorisches Museum, Vienna, Austria Naturhistorisches Museum Vienna Austria; 15 Far-Eastern Branch of the Russian Academy of Sciences, Petropavlovsk-Kamchatsky, Russia Far-Eastern Branch of the Russian Academy of Sciences Petropavlovsk-Kamchatsky Russia; 16 Auckland University of Technology, Auckland, New Zealand Auckland University of Technology Auckland New Zealand; 17 National Oceanography Centre, University of Southampton Waterfront Campus, Southampton, United Kingdom National Oceanography Centre, University of Southampton Waterfront Campus Southampton United Kingdom

**Keywords:** deep-sea mining, INDEX, fauna catalogue, video imagery, photographs, biodiversity

## Abstract

**Background:**

The growing interest in mineral resources of the deep sea, such as seafloor massive sulphide deposits, has led to an increasing number of exploration licences issued by the International Seabed Authority. In the Indian Ocean, four licence areas exist, resulting in an increasing number of new hydrothermal vent fields and the discovery of new species. Most studies focus on active venting areas including their ecology, but the non-vent megafauna of the Central Indian Ridge and South East Indian Ridge remains poorly known.

In the framework of the Indian Ocean Exploration project in the German license area for seafloor massive sulphides, baseline imagery and sampling surveys were conducted yearly during research expeditions from 2013 to 2018, using video sledges and Remotely Operated Vehicles.

**New information:**

This is the first report of an imagery collection of megafauna from the southern Central Indian- and South East Indian Ridge, reporting the taxonomic richness and their distribution. A total of 218 taxa were recorded and identified, based on imagery, with additional morphological and molecular confirmed identifications of 20 taxa from 89 sampled specimens. The compiled fauna catalogue is a synthesis of megafauna occurrences aiming at a consistent morphological identification of taxa and showing their regional distribution. The imagery data were collected during multiple research cruises in different exploration clusters of the German licence area, located 500 km north of the Rodriguez Triple Junction along the Central Indian Ridge and 500 km southeast of it along the Southeast Indian Ridge.

## Introduction

The Central Indian Ridge (CIR) and South East Indian Ridge (SEIR) are part of a global oceanic ridge system with high magmatic activity, which creates new seafloor, volcanoes and hydrothermal vent fields (Kelley 2001, Wang et al. 2012). High-temperature hydrothermal activity is often focused along topographically shallow portions of a single ridge segment, where a large magma reservoir causes crustal buoyancy (Ballard et al. 1981, Francheteau and Ballard 1983, Herzig and Hannington 1995). The hydrothermal activity accumulates polymetallic sulphides on the seafloor that may form seafloor massive sulphide (SMS) deposits with high content of iron, copper, zinc and rare earth metals in economically valuable amounts (Van Dover 2011, Miller et al. 2018, Van Dover 2019). The highly abundant and mostly symbiotic fauna that exist in hydrothermally active deposits in the Indian Ocean benefit from bacterial primary production and consist mainly of shrimp swarms, bivalve mussel beds and dense anemone fields (Hashimoto et al. 2001).

Along the spreading axes of the Indian Ocean, exploration licences for SMS deposits as potential mineral resources are issued by the International Seabed Authority (ISA; www.isa.org.jm). The ISA manages the areas beyond national jurisdiction (Levin et al. 2016) and regulates the human activities in the context of mineral resource exploration and exploitation that may take place in the near future (Ramirez-Llodra et al. 2011). This includes the protection of the marine environment from anthropogenic impacts, such as mineral extraction and mining technology testing (Rogers et al. 2012, Levin et al. 2016, Miller et al. 2018). To date, seven exploration licences for SMS deposits have been issued by the ISA; three on the Mid-Atlantic Ridge (MAR) and four in the Indian Ocean (www.isa.org.jm).

Possible future mining events at inactive hydrothermal vents will physically remove hard substrates and the local fauna, thereby flattening the vertical topography and permanently reducing habitat heterogeneity (Van Dover 2014, Levin et al. 2016, Van Dover et al. 2020). Since inactive vents may act as stepping stones for population recolonisation, removal of substrate during SMS mineral extraction may halt recolonisation (Van Dover 2014, Van Dover et al. 2020).

Such mining-related activities and disturbances will likely affect the hydrothermal vent fields and their surrounding areas, making taxonomic and ecological baseline studies essential for describing undisturbed environmental conditions and assessing potential mining impacts (Copley et al. 2016). Imagery-based studies are an accurate and cost-effective tool (Sen et al. 2014, Van Dover 2014), as they are able to detect environmental changes or serious harm (Van Dover 2019) to the ecosystem.

Deep-sea megafauna are important components of biodiversity and play significant roles in ecosystem functioning, as pointed out for megafauna occurring in nodule areas (Smith et al. 2008, Vanreusel et al. 2016). These ecosystem functions include utilisation rates of surface derived detritus, dietary composition or the locomotion mode. Megafauna are likely subject to drastic changes due to mining activities and are expected to recover slowly related with slow rates of growth and recruitment (Ramirez-Llodra et al. 2011, Amon et al. 2016, Gollner et al. 2017). Many megafauna taxa are considered as indicators of physical disturbance responding to disturbance events with changes in densities, dominating taxa or the community composition (Bluhm and Gebruk 1999, Amon et al. 2017).

**Figure 1.**
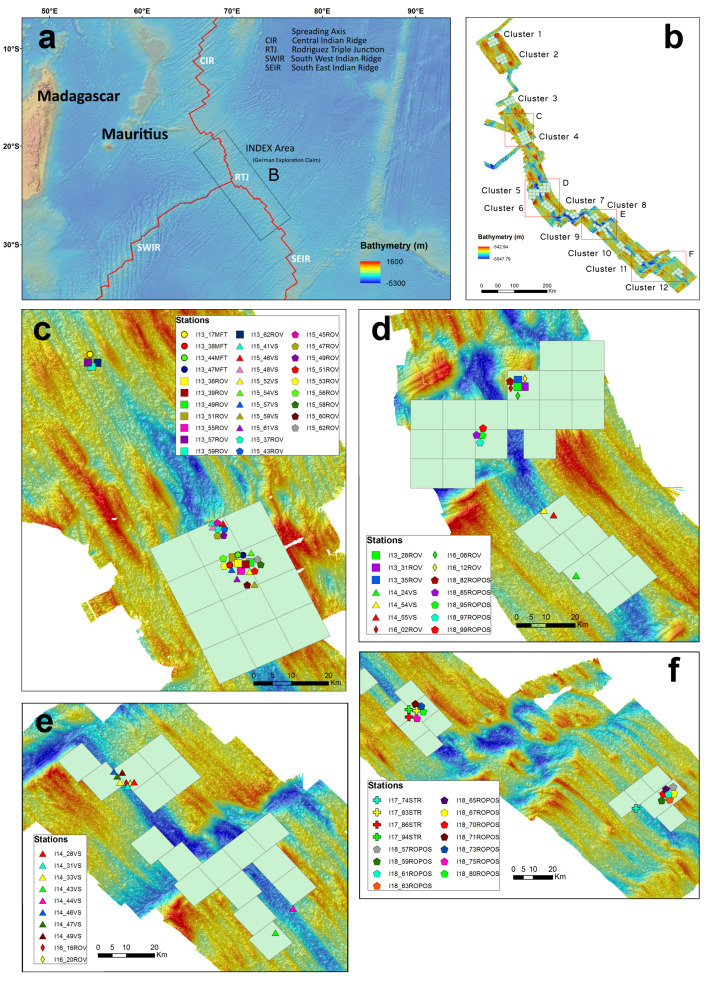
Locations of imagery stations within the INDEX exploration area. a) Overview map of the southwestern Indian Ocean showing the ridge spreading axes (red line). b) Cluster 1-12 of the GLA for polymetallic massive sulphide exploration. The red boxes show the areas enlarged in C-F; each includes imagery transects conducted in each area. c) MESO area and Cluster 4. d) Cluster 5 and 6. e) Cluster 8 and 9. f) Cluster 11 and 12. Bathymetry data provided by the Federal Institute for Geoscience and Natural Resources (BGR).

Environmental research has been conducted in the framework of the German Indian Ocean Exploration (INDEX) project and includes biological benthic baseline studies in accordance with ISA environmental guidelines (www.isa.org.jm (ISBA/25/LTC/6/Rev.1); Ardron et al. 2011, Miller et al. 2018). The German licence area (GLA) in the Indian Ocean is located along the southern CIR and northern SEIR (Fig. 1). The INDEX project is conducted by the Federal Institute for Geoscience and Natural Resources (BGR, www.bgr.bund.de) and aims to find massive sulphide deposits in economically valuable amounts for potential exploitation. The GLA covers 100 exploration blocks, each 10 x 10 km in size, with a total area of 10,000 km^2^ of deep-sea floor. A vast number of photographs and video material has been collected during annual expeditions from 2013 to 2018 and has been used to create annual fauna catalogues of specific regions or clusters within the licence area.

This study combined the existing imagery of the taxa observed within the GLA to create a taxonomic expert-revised faunal catalogue with consistent identifications for the six years of exploration. The goal of the image analysis was to assess the species richness of benthic megafauna within the GLA, covering active and inactive hydrothermal vent fields and their surrounding non-vent areas, namely the abyssal deep-sea areas along the spreading axis of the southern CIR, the Rodriguez Triple Junction (RTJ) and the northern SEIR.

## Materials and methods

### INDEX expeditions and imagery acquisition

Six expeditions in consecutive years from 2013 to 2018 were used for this imagery study and covered a cumulative bottom track distance of 220,983 m. A total of 122,918 photographs and 367 hours 10 minutes of video imagery were collected. All photographs were reviewed and faunal occurrences annotated and extracted; video imagery was only used when additional imagery was necessary to clarify the imagery-based identification. The study area has a bathymetrical range of 2,280 – 3,770 m and spanned a geographical range from the MESO areas (after RV Meteor and RV Sonne; Muench et al. 1999) outside the German licence area on the southern CIR at 23°23'S, 69°14'E, to Cluster 12 on the northern SEIR at 27°42'S, 73°44'E (Fig. 1). The MESO area was studied during the pre-exploration period and later excluded before signing the exploration licence in 2015.

All imagery transects were conducted using three different video sledges and three Remotely Operated Vehicles (ROVs). The towed video platforms were the Multifunctional Tool (MFT) and the *STROMER* (STR), both belonging to the BGR and the video sledge (VS) of the Royal Netherlands Institute for Sea Research (NIOZ). The ROVs were the *Kiel 6000* from the GEOMAR, the Remotely Operated Platform for Ocean Science (*ROPOS*) of the Canadian Scientific Submersible Facility (CSSF) and the ROV *Victor 6000* of the IFREMER. Table 1 lists gear details and total photographs and video imagery collected. Table 2 lists specifications of the sampling gear, such as the camera equipment used and the imagery resolution.

### Megafaunal imagery analysis

All taxa visible in photographs and video were annotated, cut out, and the individuals identified to the lowest taxonomic level possible. Photographic analysis was carried out using Adobe Photoshop Lightroom 5.7 (©2019 Adobe Systems Software Ireland Ltd.; www.adobe.com). Photographs were imported and automatically white-balanced and tone-corrected; year and station were added to the metadata for standardisation purposes and use in an imagery database.

Each photograph was magnified until identification was impossible due to pixelation and each section searched for both sessile and motile animals (Schoening et al. 2012). For each individual organism, a copy was created and the section containing the animal cut out. Folders were created in Lightroom in a taxonomic hierarchy following the classification in the World Register of Marine Species (WoRMS Editorial Board 2021). The morphotype or species and the abundance in the photographs were written into the metadata and resulted in up to 100 cut-outs from different photographs of individual taxa. The initial identification was based on taxonomic and imagery databases, photograph and video imagery galleries and scientific publications. In addition, selected images of all taxa were validated by the respective taxonomic experts of the team to check for consistency of the initial identification (Suppl. material 1).

The metadata of the processed photographs were exported and included taxon, abundance, expedition, year, station, GPS coordinates and technical information about the camera using the plug-in ListView (www.lightroomsolutions.com) for Adobe Photoshop Lightroom; the fauna catalogue shows selected images of each taxon.

The video imagery analysis was carried out with the video processing programme Magix Video deluxe 2014 Premium (©2003-2020 MAGIX Software GmbH; www.magix.com) primarily using video tracks from the ROVs; MFT video imagery was only processed in addition to photographs, if supporting the identification.

Both frame grabs from the video and short video films were exported and the frame grabs were used for the extension of the fauna catalogue. Short video films were cut out and added if the movement was characteristic for the animal and, therefore, helpful for identification (pers. comm. H. Singh Woods Hole Oceanographic Institute, Massachusetts, USA). The identification process was carried out in the same way as for the photographs. Detailed metadata information, such as sample locations, gear, depth, sampled specimens and camera information are listed in Suppl. material 1.

### Sample collection

A total of 89 specimens were collected throughout the expeditions using ROVs, mainly within active hydrothermal vent fields. Megafaunal specimens were quickly transferred to chilled seawater and photographed, and tissue subsamples or the entire animal were preserved in 96% ethanol for molecular analysis. Onboard photographs of the sampled specimens were included in this manuscript if they showed characteristic details helpful for taxonomic identification. In the laboratory, the specimens were assigned to the lowest taxonomic level possible using a sequenced segment of approximately 650 bp of the cytochrome oxidase subunit I (COI) gene. Molecular samples were used to verify identifications based on images.

### Applied open taxonomic nomenclature and technical notes

Note: Scaling of photographs was, in most cases, not possible due to missing lasers or lasers visible on a different focal plane. Only size estimates, based on samples or in relation to known sizes of taxa, are given. The identification of taxa, based only on imagery, is very difficult and many of the taxa presented herein might be new species. Llife traces and the phylum Porifera, with the exception of a single trace listed as poriferan taxon, *Paleodictyonnodosum*, are excluded from this catalogue. The phylum Porifera was excluded from this megafauna catalogue because of the high diversity of this group and the difficulties to discriminate these morphologically very similar taxa from one another, based on imagery alone without physical samples within the INDEX area.

Some putative taxa presented have an asterisk followed by an additional taxon name in squared brackets as, for example, “Genus species *[Genus species sp. inc.]”. This means that the image shown in the catalogue has the “Genus species” identification level, even though there do exist certain images in Suppl. material 1, where the species level identification remains uncertain. These images are indicated by “*[Genus species sp. inc.]”, but are assumed to belong to this higher ranked identification level. This was applied where only part, but not all, of the imagery samples could confirm a taxonomic group, based on morphological or molecular results (or both). It has been suggested, in these cases, to move the identification rank up to the level where all images could be reliably identified (Horton et al. 2021). Since this meant a loss of accuracy of the generated dataset and omission of valid identifications made, we decided to keep the higher level of accuracy and introduced and defined the use of the asterisk and squared brackets.

Several abbreviations were used in this fauna catalogue following the recommendations for standardisation of imagery-based annotations by Horton et al. (2021). The taxonomic ranks ˈcl.ˈ (“class”), ˈord.ˈ (“order”), ˈfam.ˈ (“family”), ˈgen.ˈ (“genus”) and ˈsp.ˈ (species) indicate the taxonomic rank and are always combined with the open nomenclature (ON) signs ˈindet.ˈ, ˈinc.ˈ or a unique code applied for this taxon (*taxon rank* (*unique code*)). Several morphotypes have a unique code in brackets (DZMB_2021_00xx), that has been assigned to all morphotypes where more than one taxon could be distinguished, but not identified to species level, based on the available imagery. These ON signs are defined according to Horton et al. (2021) as follows:

- The ˈspp.ˈ (“species (plural)”) was used when there is more than one species present within an identified group of organisms, but could not be further distinguished, based on the imagery.
- The ˈindet.ˈ (“indeterminabilis”) means that no further identification was possible because diagnostic characteristics were not visible. Missing diagnostic characteristics are often related to blurry imagery, low resolution, orientation of the organism and missing physical samples.
- The ˈinc.ˈ (“incerta”) means that diagnostic characteristics and (or) physical sample were present, but the identification is still uncertain and needs further comparable material for validation.

Table 3 contains all taxa included in this catalogue.

## Data resources

The following Table 4 contains a brief description of content of all fields present in the Suppl. material 1.

## Checklists

### Megafauna of the German exploration license area

#### Animalia

221EC24D-89E7-554F-BD5F-92AA56627748

#### Annelida

Lamarck, 1809

240121EB-4D4A-52F0-A922-67836A185E7C

#### Polychaeta

Grube, 1850

E173DCBE-1B89-524F-8AA1-10C9F51CCDD7

#### Amphinomida

6DFA7991-3A29-5390-8F33-6F5D81F1AE7E

#### Amphinomidae

Lamarck, 1818

CB214452-ABC0-5A47-BFB4-A9F3445631BE

#### Archinome

Kudenov, 1991

572D6587-861D-5ACF-A76A-C6761A767731

#### Archinome jasoni f. *[Archinome jasoni sp. inc.]

Borda, Kudenov, Chevaldonné, Blake, Desbruyères, Fabri, Hourdez, Pleijel, Shank, Wilson, Schulze & Rouse, 2013

2BCBBC50-6C45-5C77-A8B5-C028765425D4

##### Materials

- **Type status:** Other material. **Occurrence:** recordedBy: BGR/ GEOMAR; individualCount: 100; lifeStage: Adult; behavior: moving at basis of active chimney; occurrenceStatus: present; preparations: DNA voucher and animal stored in 96% ethanol; associatedMedia: 2013-12-05_11-07-30_Sonne_INDEX2013-2_028ROV01_Logo.jpg; associatedOccurrences: none; associatedSequences: COI; **Taxon:** taxonID: I13_390; scientificNameID: Archinomejasoni; taxonConceptID: Archinomejasoni; scientificName: Archinomejasoni; kingdom: Animalia; phylum: Annelida; class: Polychaeta; order: Amphinomida; family: Amphinomidae; genus: Archinome; taxonRank: Species; scientificNameAuthorship: Borda, Kudenov, Chevaldonné, Blake, Desbruyčres, Fabri, Hourdez, Pleijel, Shank, Wilson, Schulze & Rouse, 2013; **Location:** waterBody: Indian Ocean; stateProvince: Rodriguez Triple Junction; locality: Kairei; verbatimLocality: Cluster 5; maximumDepthInMeters: 2432; locationRemarks: FS Sonne Cruise INDEX2013 Leg 2; decimalLatitude: -25.3205; decimalLongitude: 70.0401; geodeticDatum: WGS84; coordinateUncertaintyInMeters: 25; **Identification:** identifiedBy: Theresa Guggolz; identificationRemarks: Identified by morphology and DNA of collected specimen; **Event:** eventDate: 2013-12-05; eventTime: 11:07:30 am; year: 2013; fieldNumber: INDEX2013-28ROV; fieldNotes: 1.8°C; **Record Level:** language: en; institutionCode: DZMB; collectionCode: I13_28RO_SG1_2; datasetName: INDEX; basisOfRecord: Preserved Specimen

##### Notes

Fig. 2

#### Phyllodocida

Dales, 1962

6C9FAB24-630D-522F-971E-3270AB698FA4

#### Polynoidae

Kinberg, 1856

CC44CA48-CCD3-5B55-97F8-5865F4A1ACEA

#### Polynoidae gen. indet.

8E8830B8-8724-50E7-94B1-F386B1AA1ED5

##### Materials

- **Type status:** Other material. **Occurrence:** recordedBy: ROPOS.COM; individualCount: 2; lifeStage: Adult; behavior: on anemone; occurrenceStatus: present; preparations: Imaged only; associatedMedia: R2103_00177.jpg; associatedOccurrences: Actinostolidae gen. indet.; **Taxon:** taxonConceptID: Polynoidae gen. indet.; kingdom: Animalia; phylum: Annelida; class: Polychaeta; order: Phyllodocida; family: Polynoidae; taxonRank: Family; scientificNameAuthorship: Kinberg, 1856; **Location:** waterBody: Indian Ocean; stateProvince: Rodriguez Triple Junction; locality: RTJ; verbatimLocality: Cluster 5; maximumDepthInMeters: 2398; locationRemarks: RV Pelagia Cruise INDEX2018 Leg 2; geodeticDatum: WGS84; coordinateUncertaintyInMeters: 25; **Identification:** identifiedBy: Theresa Guggolz; identificationRemarks: Identified only from imagery - commensal morphotype only observed on Actinostolidae gen. indet.; identificationQualifier: gen. indet.; **Event:** eventDate: 2018-12-03; eventTime: 9:02:06 am; year: 2018; fieldNumber: INDEX2018-82ROPOS; fieldNotes: 1.8°C, 34.7 ppt; **Record Level:** language: en; institutionCode: DZMB; datasetName: INDEX; basisOfRecord: Human Observation

##### Notes

Fig. 3

#### Branchipolynoe

Pettibone, 1984

8DF5E696-9090-5900-A1EE-A557F759AF0A

#### Branchipolynoe gen. inc.

6CDCC255-44A4-5103-8C50-C081AF86456C

##### Materials

- **Type status:** Other material. **Occurrence:** recordedBy: ROPOS.COM; individualCount: 1; lifeStage: Adult; behavior: on sediment; occurrenceStatus: present; preparations: Imaged only; associatedMedia: R1913_01678.jpg; **Taxon:** taxonConceptID: Branchipolynoe gen. inc.; kingdom: Animalia; phylum: Annelida; class: Polychaeta; order: Phyllodocida; family: Polynoidae; genus: Branchipolynoe; taxonRank: Genus; scientificNameAuthorship: Pettibone, 1984; **Location:** waterBody: Indian Ocean; stateProvince: Central Indian Ridge; locality: EGS; verbatimLocality: Cluster 4; maximumDepthInMeters: 3280; locationRemarks: RV Pelagia Cruise INDEX2015 Leg 2; geodeticDatum: WGS84; coordinateUncertaintyInMeters: 33; **Identification:** identifiedBy: Theresa Guggolz; identificationRemarks: Identified only from imagery; identificationQualifier: gen. inc.; **Event:** eventDate: 2015-12-06; eventTime: 5:19:55 am; year: 2015; fieldNumber: INDEX2015-58ROV; fieldNotes: 1.8°C, 34.7 ppt; **Record Level:** language: en; institutionCode: DZMB; datasetName: INDEX; basisOfRecord: Human Observation

##### Notes

Fig. 4

#### Lepidonotopodium

Pettibone, 1983

7FF05E9C-B03F-5F0F-BF70-651F83C74567

#### Lepidonotopodium gen. inc. (DZMB_2021_0001)

033BE3C9-87EA-5658-B8A3-397E2FC6548A

##### Materials

- **Type status:** Other material. **Occurrence:** recordedBy: ROPOS.COM; individualCount: 1; lifeStage: Adult; behavior: moving on active chimney; occurrenceStatus: present; preparations: Imaged only; associatedMedia: R2106_00050.jpg; **Taxon:** taxonConceptID: Lepidonotopodium gen. inc. (DZMB_2021_0001); kingdom: Animalia; phylum: Annelida; class: Polychaeta; order: Phyllodocida; family: Polynoidae; genus: Lepidonotopodium; taxonRank: Genus; scientificNameAuthorship: Pettibone, 1983; **Location:** waterBody: Indian Ocean; stateProvince: Rodriguez Triple Junction; locality: Vent site 4; verbatimLocality: Cluster 5; maximumDepthInMeters: 2630; locationRemarks: RV Pelagia Cruise INDEX2018 Leg 2; geodeticDatum: WGS84; coordinateUncertaintyInMeters: 23; **Identification:** identifiedBy: Theresa Guggolz; identificationRemarks: Identified only from imagery; identificationQualifier: gen. inc.; **Event:** eventDate: 2018-12-10; eventTime: 5:51:39 am; year: 2018; fieldNumber: INDEX2018-97ROPOS; fieldNotes: 1.8°C, 34.7 ppt; **Record Level:** language: en; institutionCode: DZMB; datasetName: INDEX; basisOfRecord: Human Observation

##### Notes

Fig. 5

#### Lepidonotopodium gen. inc. (DZMB_2021_0002)

DC5CBA81-74AD-5D03-AD52-6D25B133DC57

##### Materials

- **Type status:** Other material. **Occurrence:** recordedBy: ROPOS.COM; individualCount: 1; lifeStage: Adult; behavior: moving on active chimney; occurrenceStatus: present; preparations: Imaged only; associatedMedia: R2102_00026.jpg; associatedOccurrences: none; **Taxon:** taxonConceptID: Lepidonotopodium gen. inc. (DZMB_2021_0002); kingdom: Animalia; phylum: Annelida; class: Polychaeta; order: Phyllodocida; family: Polynoidae; genus: Lepidonotopodium; taxonRank: Genus; scientificNameAuthorship: Pettibone, 1983; **Location:** waterBody: Indian Ocean; stateProvince: South East Indian Ridge; locality: Vent site 5; verbatimLocality: Cluster 11; maximumDepthInMeters: 2911; locationRemarks: RV Pelagia Cruise INDEX2018 Leg 2; geodeticDatum: WGS84; coordinateUncertaintyInMeters: 30; **Identification:** identifiedBy: Theresa Guggolz; identificationRemarks: Identified only from imagery; identificationQualifier: gen. inc.; **Event:** eventDate: 2018-12-01; eventTime: 6:15:16 am; year: 2018; fieldNumber: INDEX2018-80ROPOS; fieldNotes: 1.7°C, 34.7 ppt; **Record Level:** language: en; institutionCode: DZMB; datasetName: INDEX; basisOfRecord: Human Observation

##### Notes

Fig. 6

#### Lepidonotopodium gen. inc. (DZMB_2021_0003)

6F762306-6385-5B14-B0B0-3BD39B35EE77

##### Materials

- **Type status:** Other material. **Occurrence:** recordedBy: ROPOS.COM; individualCount: 1; lifeStage: Adult; behavior: moving on active chimney; occurrenceStatus: present; preparations: Imaged only; associatedMedia: R2095_00136.jpg; **Taxon:** taxonConceptID: Lepidonotopodium gen. inc. (DZMB_2021_0003); kingdom: Animalia; phylum: Annelida; class: Polychaeta; order: Phyllodocida; family: Polynoidae; genus: Lepidonotopodium; taxonRank: Genus; scientificNameAuthorship: Pettibone, 1983; **Location:** waterBody: Indian Ocean; stateProvince: South East Indian Ridge; locality: Vent site 6; verbatimLocality: Cluster 12; maximumDepthInMeters: 2479; locationRemarks: RV Pelagia Cruise INDEX2018 Leg 2; geodeticDatum: WGS84; coordinateUncertaintyInMeters: 24; **Identification:** identifiedBy: Theresa Guggolz; identificationRemarks: Identified only from imagery; identificationQualifier: gen. inc.; **Event:** eventDate: 2018-11-23; eventTime: 9:42:28 am; year: 2018; fieldNumber: INDEX2018-63ROPOS; **Record Level:** language: en; institutionCode: DZMB; datasetName: INDEX; basisOfRecord: Human Observation

##### Notes

Fig. 7

#### Sabellida

Levinsen, 1883

306A7CC2-57FC-5ACA-BEAA-409FB6C67F21

#### Sabellidae

Latreille, 1825

522B16B0-9E4E-551E-B20A-9630164EBA3A

#### Sabellidae gen. indet.

CEAB417F-2367-5CD5-9430-CCABCA1925BD

##### Materials

- **Type status:** Other material. **Occurrence:** recordedBy: BGR; individualCount: 1; lifeStage: Adult; behavior: on sediment; occurrenceStatus: present; preparations: Imaged only; associatedMedia: IMG_5003.jpg; **Taxon:** taxonConceptID: Sabellidae gen. indet.; kingdom: Animalia; phylum: Annelida; class: Polychaeta; order: Sabellida; family: Sabellidae; taxonRank: Family; scientificNameAuthorship: Latreille, 1825; **Location:** waterBody: Indian Ocean; stateProvince: South East Indian Ridge; locality: SEIR; verbatimLocality: Cluster 11; maximumDepthInMeters: 2928; locationRemarks: FS Sonne Cruise INDEX2017 Leg 1; decimalLatitude: -27.2562; decimalLongitude: 72.7216; geodeticDatum: WGS84; coordinateUncertaintyInMeters: 29; **Identification:** identifiedBy: Theresa Guggolz; identificationRemarks: Identified only from imagery; identificationQualifier: gen. indet.; **Event:** eventDate: 2017-09-25; eventTime: 3:47:53 am; year: 2017; fieldNumber: INDEX2017-86STR; fieldNotes: 1.7°C, 34.7 ppt; **Record Level:** language: en; institutionCode: DZMB; datasetName: INDEX; basisOfRecord: Human Observation

##### Notes

Fig. 8

#### Siboglinidae

Caullery, 1914

DFA8289E-1BF2-5978-A2BF-BB94B388390E

#### Oasisia

Jones, 1985

6D349137-D6CD-5B0D-BB18-69DF1305DB29

#### Oasisia gen. inc.

44C7BBA3-DF1A-5C0F-9917-4CE2B2F1C3E6

##### Materials

- **Type status:** Other material. **Occurrence:** recordedBy: BGR/ GEOMAR; individualCount: 100; lifeStage: Adult; behavior: in sulphidic sediment; occurrenceStatus: present; preparations: Imaged only; associatedMedia: 2013-12-08_10-50-40_Sonne_INDEX2013-2_036ROV04_Logo.jpg; **Taxon:** taxonConceptID: Oasisia gen. inc.; kingdom: Animalia; phylum: Annelida; class: Polychaeta; order: Sabellida; family: Siboglinidae; genus: Oasisia; taxonRank: Genus; scientificNameAuthorship: Jones, 1985; **Location:** waterBody: Indian Ocean; stateProvince: Central Indian Ridge; locality: Edmond; verbatimLocality: Cluster 4; maximumDepthInMeters: 3269; locationRemarks: FS Sonne Cruise INDEX2013 Leg 2; geodeticDatum: WGS84; coordinateUncertaintyInMeters: 33; **Identification:** identifiedBy: Theresa Guggolz; identificationRemarks: Identified only from imagery; identificationQualifier: gen. inc.; **Event:** eventDate: 2013-12-08; eventTime: 10:50:40 am; year: 2013; fieldNumber: INDEX2013-36ROV; fieldNotes: 1.8°C; **Record Level:** language: en; institutionCode: DZMB; datasetName: INDEX; basisOfRecord: Human Observation

##### Notes

Fig. 9

#### Terebellida

sensu Rouse & Fauchald, 1997

F1026BDD-6BBA-5131-BA3A-E116F343B52B

#### Alvinellidae

Desbruyeres & Laubier, 1986

37FCB6CC-DE75-5709-8D64-39A2028390FD

#### Alvinella

Desbruyères & Laubier, 1980

DECCF05C-B238-5850-AC6A-1569C84BB580

#### Alvinella gen. inc.

DA62AED3-E16E-5656-B2C0-6C96EB6D2A0D

##### Materials

- **Type status:** Other material. **Occurrence:** recordedBy: ROPOS.COM; individualCount: 100; lifeStage: Adult; behavior: attached to active chimney; occurrenceStatus: present; preparations: DNA voucher and animal stored in 96% ethanol; associatedMedia: Alvinella sp.tif; associatedOccurrences: none; associatedSequences: COI; **Taxon:** taxonID: I18_1138; scientificNameID: -; taxonConceptID: Alvinella gen. inc.; kingdom: Animalia; phylum: Annelida; class: Polychaeta; order: Terebellida; family: Alvinellidae; genus: Alvinella; taxonRank: Genus; scientificNameAuthorship: Desbruyeres & Laubier, 1980; **Location:** waterBody: Indian Ocean; stateProvince: South East Indian Ridge; locality: Vent site 6; verbatimLocality: Cluster 12; maximumDepthInMeters: 2449; locationRemarks: RV Pelagia Cruise INDEX2018 Leg 2; geodeticDatum: WGS84; coordinateUncertaintyInMeters: 24; **Identification:** identifiedBy: Theresa Guggolz; identificationRemarks: Identified by morphology and DNA of collected specimen; identificationQualifier: gen. inc.; **Event:** eventDate: 2018-11-24; eventTime: 9:31:00 am; year: 2018; fieldNumber: INDEX2018-65ROPOS; fieldNotes: 1.8°C, 34.7 ppt; **Record Level:** language: en; institutionCode: DZMB; collectionCode: I18_065RO_B_005; datasetName: INDEX; basisOfRecord: Human Observation

##### Notes

Fig. 10

#### Arthropoda

von Siebold, 1848

645D3CDA-4BEE-58D3-963B-6F10BA2D63FE

#### Hexanauplia

Oakley, Wolfe, Lindgren & Zaharof, 2013

CC49FEBC-D597-593F-94CA-A0A4ED8F80A8

#### Thoracica

Darwin, 1854

BFAE7E6A-02B9-5CE6-8850-C61398C393DF

#### Lepadiformes

Buckeridge & Newman, 2006

B9339163-B611-52B8-A76F-28C22B833456

#### Poecilasmatidae

Annandale, 1909

41E3CF20-93F6-5FC0-BE8F-6DD67DDAE49E

#### Glyptelasma

Pilsbry, 1907

BE386F82-66D6-58BF-A098-7F9195B589B0

#### Glyptelasma gen. inc.

FD0EFFA2-B53E-57DB-A7FE-A74290E7BF26

##### Materials

- **Type status:** Other material. **Occurrence:** recordedBy: ROPOS.COM; individualCount: 100; lifeStage: Adult; behavior: Attached to coral stalk; occurrenceStatus: present; preparations: Imaged only; associatedMedia: R2097_00145.jpg; associatedOccurrences: Isididae Jasonisis gen. inc.; **Taxon:** taxonConceptID: Glyptelasma gen. inc.; kingdom: Animalia; phylum: Arthropoda; class: Hexanauplia; order: Lepadiformes; family: Poecilasmatidae; genus: Glyptelasma; taxonRank: Genus; scientificNameAuthorship: Pilsbry, 1907; **Location:** waterBody: Indian Ocean; stateProvince: South East Indian Ridge; locality: Vent site 6; verbatimLocality: Cluster 12; maximumDepthInMeters: 2374; locationRemarks: RV Pelagia Cruise INDEX2018 Leg 2; geodeticDatum: WGS84; coordinateUncertaintyInMeters: 24; **Identification:** identifiedBy: Kate Shalaeva; identificationRemarks: Identified only from imagery; identificationQualifier: gen. inc.; **Event:** eventDate: 2018-11-25; eventTime: 8:14:41 am; year: 2018; fieldNumber: INDEX2018-67ROPOS; **Record Level:** language: en; institutionCode: DZMB; datasetName: INDEX; basisOfRecord: Human Observation

##### Notes

Fig. 11

#### Scalpelliformes

Buckeridge & Newman, 2006

11B7EC57-FED6-53AC-AA7C-F8C9639C035C

#### Eolepadidae

Buckeridge, 1983

39957641-2D65-5090-926D-02F3E8E65469

#### Neolepas

Newman, 1979

54F772AF-4EC2-517A-A236-B4AE82F7824C

#### Neolepas marisindica sp. inc.

Watanabe, Chen & Chan, 2018

6D134EE3-AAF3-5879-96FF-105387BDE9FF

##### Materials

- **Type status:** Other material. **Occurrence:** recordedBy: ROPOS.COM; individualCount: 100; lifeStage: Adult; behavior: attached to active chimney; occurrenceStatus: present; preparations: Imaged only; associatedMedia: R2095_00131.jpg; **Taxon:** taxonConceptID: Neolepasmarisindica sp. inc.; scientificName: Neolepasmarisindica; kingdom: Animalia; phylum: Arthropoda; class: Hexanauplia; order: Scalpelliformes; family: Eolepadidae; genus: Neolepas; taxonRank: Species; scientificNameAuthorship: Watanabe, Chen & Chan, 2018; **Location:** waterBody: Indian Ocean; stateProvince: South East Indian Ridge; locality: Vent site 6; verbatimLocality: Cluster 12; maximumDepthInMeters: 2468; locationRemarks: RV Pelagia Cruise INDEX2018 Leg 2; geodeticDatum: WGS84; coordinateUncertaintyInMeters: 24; **Identification:** identifiedBy: Kate Shalaeva; identificationRemarks: Identified only from imagery; identificationQualifier: sp. inc.; **Event:** eventDate: 2018-11-23; eventTime: 9:25:45 am; year: 2018; fieldNumber: INDEX2018-63ROPOS; **Record Level:** language: en; institutionCode: DZMB; datasetName: INDEX; basisOfRecord: Human Observation

##### Notes

Fig. 12

#### Scalpellidae

Pilsbry, 1907

95464930-B35C-5B33-B5E6-37B4CB35B294

#### Regioscalpellum

Gale, 2015

BA7CB5E6-847C-522D-918D-1B581A6BFCD0

#### Regioscalpellum regium sp. inc.

(Wyville Thomson, 1873)

FAFFB341-EB2B-58E0-A47F-230C19CE42A7

##### Materials

- **Type status:** Other material. **Occurrence:** recordedBy: ROPOS.COM; individualCount: 100; lifeStage: Adult; behavior: attached to sulfides; occurrenceStatus: present; preparations: Imaged only; associatedMedia: R2095_00029-1.jpg; **Taxon:** taxonConceptID: Regioscalpellumregium sp. inc.; scientificName: Regioscalpellumregium; kingdom: Animalia; phylum: Arthropoda; class: Hexanauplia; order: Scalpelliformes; family: Scalpellidae; genus: Regioscalpellum; taxonRank: Species; scientificNameAuthorship: (Wyville Thomson, 1873); **Location:** waterBody: Indian Ocean; stateProvince: South East Indian Ridge; locality: Vent site 6; verbatimLocality: Cluster 12; maximumDepthInMeters: 2380; locationRemarks: RV Pelagia Cruise INDEX2018 Leg 2; geodeticDatum: WGS84; coordinateUncertaintyInMeters: 24; **Identification:** identifiedBy: Kate Shalaeva; identificationRemarks: Identified only from imagery; identificationQualifier: sp. inc.; **Event:** eventDate: 2018-11-23; eventTime: 5:50:33 am; year: 2018; fieldNumber: INDEX2018-63ROPOS; **Record Level:** language: en; institutionCode: DZMB; datasetName: INDEX; basisOfRecord: Human Observation

##### Notes

Fig. 13

#### Sessilia

Lamarck, 1818

09A01532-A761-5B39-8C7C-A6C260DF0936

#### Verrucidae

Darwin, 1854

16B59BD1-FA74-52C6-9280-4A77DBC7EAB7

#### Verrucidae fam. inc.

FD035FBC-782E-5FC7-B6C1-637041C2809C

##### Materials

- **Type status:** Other material. **Occurrence:** recordedBy: ROPOS.COM; individualCount: 100; lifeStage: Adult; behavior: attached to sulfides; occurrenceStatus: present; preparations: Imaged only; associatedMedia: R2095_00029-2.jpg; **Taxon:** taxonConceptID: Verrucidae fam. inc.; kingdom: Animalia; phylum: Arthropoda; class: Hexanauplia; order: Sessilia; family: Verrucidae; taxonRank: Family; scientificNameAuthorship: Darwin, 1854; **Location:** waterBody: Indian Ocean; stateProvince: South East Indian Ridge; locality: Vent site 6; verbatimLocality: Cluster 12; maximumDepthInMeters: 2380; locationRemarks: RV Pelagia Cruise INDEX2018 Leg 2; geodeticDatum: WGS84; coordinateUncertaintyInMeters: 24; **Identification:** identifiedBy: Kate Shalaeva; identificationRemarks: Identified only from imagery; identificationQualifier: fam. inc.; **Event:** eventDate: 2018-11-23; eventTime: 5:50:33 am; year: 2018; fieldNumber: INDEX2018-63ROPOS; **Record Level:** language: en; institutionCode: DZMB; datasetName: INDEX; basisOfRecord: Human Observation

##### Notes

Fig. 14

#### Malacostraca

Latreille, 1802

EC7920BE-BC62-5D62-8818-9097444ADC25

#### Amphipoda

Latreille, 1816

CF5364BC-D905-5CB3-9CB8-D3922B5C594C

#### Amphipoda ord. inc.

6BD79075-2D27-5602-9A0B-B41A0E045DBF

##### Materials

- **Type status:** Other material. **Occurrence:** recordedBy: IFREMER; individualCount: 1; lifeStage: Adult; behavior: sitting on hydrozoa stalk; occurrenceStatus: present; preparations: Imaged only; associatedMedia: 160111221731A Kopie.jpg; associatedOccurrences: Hydrozoa ord. indet.; **Taxon:** taxonConceptID: Amphipoda ord. inc.; kingdom: Animalia; phylum: Arthropoda; class: Malacostraca; order: Amphipoda; taxonRank: Order; scientificNameAuthorship: Latreille, 1816; **Location:** waterBody: Indian Ocean; stateProvince: Rodriguez Triple Junction; locality: Kairei; verbatimLocality: Cluster 5; maximumDepthInMeters: 2468; locationRemarks: RV Pourqoui pas? Cruise INDEX2016 Leg 1; geodeticDatum: WGS84; coordinateUncertaintyInMeters: 25; **Identification:** identifiedBy: Simon Bober; identificationRemarks: Identified only from imagery; identificationQualifier: ord. inc.; **Event:** eventDate: 2016-01-11; eventTime: 10:17:31 pm; year: 2016; fieldNumber: INDEX2016-06ROV; **Record Level:** language: en; institutionCode: DZMB; datasetName: INDEX; basisOfRecord: Human Observation

##### Notes

Fig. 15

#### Decapoda

Latreille, 1802

A17B56FB-68D7-5727-AEFD-E5C6AD8079A7

#### Anomura

MacLeay, 1838

4C8C05F0-796A-5394-AAFC-37C2FCA3787C

#### Anomura fam. indet.

D2FFB485-7346-59B9-98A1-388844746C46

##### Materials

- **Type status:** Other material. **Occurrence:** recordedBy: BGR; individualCount: 1; lifeStage: Adult; behavior: moving on seafloor; occurrenceStatus: present; preparations: Imaged only; associatedMedia: 17MFT Fotos 2013-63-2.jpg; **Taxon:** taxonConceptID: Anomura fam. indet.; kingdom: Animalia; phylum: Arthropoda; class: Malacostraca; order: Decapoda; taxonRank: Infraorder; scientificNameAuthorship: MacLeay, 1838; **Location:** waterBody: Indian Ocean; stateProvince: Central Indian Ridge; locality: MESO; verbatimLocality: outside INDEX claim; maximumDepthInMeters: 2825; locationRemarks: FS Sonne Cruise INDEX2013 Leg 1; decimalLatitude: -23.3922; decimalLongitude: 69.2423; geodeticDatum: WGS84; coordinateUncertaintyInMeters: 28; **Identification:** identifiedBy: Magdalini Christodoulou, Terue C. Kihara; identificationRemarks: Identified only from imagery; identificationQualifier: fam. indet.; **Event:** eventDate: 2013-11-25; eventTime: 3:21:40 am; year: 2013; fieldNumber: INDEX2013-17MFT; fieldNotes: 1.8°C, 34.7 ppt; **Record Level:** language: en; institutionCode: DZMB; datasetName: INDEX; basisOfRecord: Human Observation

##### Notes

Fig. 16

#### Galatheidae

Samouelle, 1819

EA34ED42-A7B2-54B2-92CF-9A5A88EAA411

#### Galatheidae fam. inc.

C7838E19-90D1-5A1A-8F19-2AD30A1927C4

##### Materials

- **Type status:** Other material. **Occurrence:** recordedBy: ROPOS.COM; individualCount: 1; lifeStage: Adult; behavior: on basalt; occurrenceStatus: present; preparations: Imaged only; associatedMedia: R2098_00075.jpg; **Taxon:** taxonConceptID: Galatheidae fam. inc.; kingdom: Animalia; phylum: Arthropoda; class: Malacostraca; order: Decapoda; family: Galatheidae; taxonRank: Family; scientificNameAuthorship: Samouelle, 1819; **Location:** waterBody: Indian Ocean; stateProvince: South East Indian Ridge; locality: Vent site 3; verbatimLocality: Cluster 12; maximumDepthInMeters: 2478; locationRemarks: RV Pelagia Cruise INDEX2018 Leg 2; geodeticDatum: WGS84; coordinateUncertaintyInMeters: 26; **Identification:** identifiedBy: Magdalini Christodoulou, Terue C. Kihara; identificationRemarks: Identified only from imagery; identificationQualifier: fam. inc.; **Event:** eventDate: 2018-11-26; eventTime: 6:16:31 am; year: 2018; fieldNumber: INDEX2018-70ROPOS; fieldNotes: 1.8°C, 34.7 ppt; **Record Level:** language: en; institutionCode: DZMB; datasetName: INDEX; basisOfRecord: Human Observation

##### Notes

Fig. 17

#### Munidopsidae

Ortmann, 1898

DA2BFA30-4BDA-5067-A4E6-C180C6DDB0AC

#### Munidopsis

Whiteaves, 1874

99DB7F3D-17DA-5F39-BCCF-DA47A044003C

#### Munidopsis aries sp. inc.

(A. Milne Edwards, 1880)

29B1939F-3E21-5D9A-A5EF-C3589833EFDC

##### Materials

- **Type status:** Other material. **Occurrence:** recordedBy: ROPOS.COM; individualCount: 1; lifeStage: Adult; behavior: on seafloor; occurrenceStatus: present; preparations: Imaged only; associatedMedia: R2105_00030.jpg; **Taxon:** taxonConceptID: Munidopsisaries sp. inc.; scientificName: Munidopsisaries; kingdom: Animalia; phylum: Arthropoda; class: Malacostraca; order: Decapoda; family: Munidopsidae; genus: Munidopsis; taxonRank: Species; scientificNameAuthorship: (A. Milne Edwards, 1880); **Location:** waterBody: Indian Ocean; stateProvince: Rodriguez Triple Junction; locality: Vent site 4; verbatimLocality: Cluster 5; maximumDepthInMeters: 2576; locationRemarks: RV Pelagia Cruise INDEX2018 Leg 2; geodeticDatum: WGS84; coordinateUncertaintyInMeters: 26; **Identification:** identifiedBy: Enrique MacPherson; identificationRemarks: Identified only from imagery; identificationQualifier: sp. inc.; **Event:** eventDate: 2018-12-09; eventTime: 5:42:12 am; year: 2018; fieldNumber: INDEX2018-95ROPOS; fieldNotes: 1.8°C, 34.7 ppt; **Record Level:** language: en; institutionCode: DZMB; datasetName: INDEX; basisOfRecord: Human Observation

##### Notes

Fig. 18

#### Munidopsis pallida sp. inc.

Alcock, 1894

16C3E11A-003E-562A-819B-074A25A324F8

##### Materials

- **Type status:** Other material. **Occurrence:** recordedBy: BGR/ GEOMAR; individualCount: 1; lifeStage: Adult; behavior: on seafloor; occurrenceStatus: present; preparations: Imaged only; associatedMedia: 2013-12-13_09-23-30_Sonne_INDEX2013-2_051ROV07_Logo.jpg; **Taxon:** taxonConceptID: Munidopsispallida sp. inc.; scientificName: Munidopsispallida; kingdom: Animalia; phylum: Arthropoda; class: Malacostraca; order: Decapoda; family: Munidopsidae; genus: Munidopsis; taxonRank: Species; scientificNameAuthorship: Alcock, 1894; **Location:** waterBody: Indian Ocean; stateProvince: Central Indian Ridge; locality: Vent site 2; verbatimLocality: Cluster 4; maximumDepthInMeters: 3048; locationRemarks: FS Sonne Cruise INDEX2013 Leg 2; geodeticDatum: WGS84; coordinateUncertaintyInMeters: 30; **Identification:** identifiedBy: Enrique MacPherson; identificationRemarks: Identified only from imagery; identificationQualifier: sp. inc.; **Event:** eventDate: 2013-12-13; eventTime: 9:23:30 am; year: 2013; fieldNumber: INDEX2013-51ROV; **Record Level:** language: en; institutionCode: DZMB; datasetName: INDEX; basisOfRecord: Human Observation

##### Notes

Fig. 19

#### Paguroidea

Latreille, 1802

695E4B80-3786-528A-8F9C-C0D946457C9B

#### Paguroidea superfam. inc.

A9547836-F699-5719-AF97-91CCB8675005

##### Materials

- **Type status:** Other material. **Occurrence:** recordedBy: ROPOS.COM; individualCount: 1; lifeStage: Adult; behavior: moving on sediment; occurrenceStatus: present; preparations: Imaged only; associatedMedia: R1905_00014.jpg; associatedOccurrences: Epizoanthus sp. indet.; **Taxon:** taxonConceptID: Paguroidea superfam. inc.; kingdom: Animalia; phylum: Arthropoda; class: Malacostraca; order: Decapoda; taxonRank: Superfamily; scientificNameAuthorship: Latreille, 1802; **Location:** waterBody: Indian Ocean; stateProvince: Central Indian Ridge; locality: Vent site 1; verbatimLocality: Cluster 4; maximumDepthInMeters: 3072; locationRemarks: RV Pelagia Cruise INDEX2015 Leg 2; geodeticDatum: WGS84; coordinateUncertaintyInMeters: 30; **Identification:** identifiedBy: Magdalini Christodoulou, Terue C. Kihara; identificationRemarks: Identified only from imagery; identificationQualifier: superfam. inc.; **Event:** eventDate: 2015-11-27; eventTime: 9:20:39 am; year: 2015; fieldNumber: INDEX2015-37ROV; fieldNotes: 1.8°C, 34.7 ppt; **Record Level:** language: en; institutionCode: DZMB; datasetName: INDEX; basisOfRecord: Human Observation

##### Notes

Fig. 20

#### Astacidea

Latraeille, 1802

4181414C-05A6-5740-BC7F-0AF443A3B4CA

#### Nephropidae

Dana, 1852

DF1AF4CD-C28A-53D1-9A51-92560041FE2D

#### Thymopides

Burukovsky & Averin, 1977

29CE23E7-2479-5D7E-8E17-643DC8917BDA

#### Thymopides laurentae sp. inc.

Segonzac & Macpherson, 2003

5A4D715B-7B07-556B-88D8-5896A8E115AC

##### Materials

- **Type status:** Other material. **Occurrence:** recordedBy: ROPOS.COM; individualCount: 1; lifeStage: Adult; behavior: on basalt; occurrenceStatus: present; preparations: Imaged only; associatedMedia: R1906_00143.jpg; **Taxon:** taxonConceptID: Thymopideslaurentae sp. inc.; scientificName: Thymopideslaurentae; kingdom: Animalia; phylum: Arthropoda; class: Malacostraca; order: Decapoda; family: Nephropidae; genus: Thymopides; taxonRank: Species; scientificNameAuthorship: Segonzac & Macpherson, 2003; **Location:** waterBody: Indian Ocean; stateProvince: Central Indian Ridge; locality: Vent site 1; verbatimLocality: Cluster 4; maximumDepthInMeters: 3036; locationRemarks: RV Pelagia Cruise INDEX2015 Leg 2; geodeticDatum: WGS84; coordinateUncertaintyInMeters: 30; **Identification:** identifiedBy: Enrique MacPherson; identificationRemarks: Identified only from imagery; identificationQualifier: sp. inc.; **Event:** eventDate: 2015-11-29; eventTime: 8:55:19 am; year: 2015; fieldNumber: INDEX2015-43ROV; fieldNotes: 1.8°C, 34.7 ppt; **Record Level:** language: en; institutionCode: DZMB; datasetName: INDEX; basisOfRecord: Human Observation

##### Notes

Fig. 21

#### Brachyura

Linnaeus, 1754

4AA8E231-2243-572D-8D63-D557DF75747F

#### Bythograeidae

Williams, 1980

DBA4FD86-AA18-5B3A-852F-D4EA74225AF7

#### Austinograea

Hessler & Martin, 1989

61191859-DE2C-5D34-83A4-2AC5D0D122AD

#### Austinograea rodriguezensis

Tsuchida & Hashimoto, 2002

ADD770DC-21BA-537F-9922-A881CCE17022

##### Materials

- **Type status:** Other material. **Occurrence:** recordedBy: BGR/ GEOMAR; individualCount: 3; lifeStage: Adult; behavior: moving on seafloor; occurrenceStatus: present; preparations: DNA voucher and animal stored in 96% ethanol; associatedMedia: 2013-12-06_10-57-36_Sonne_INDEX2013-2_031ROV02_Logo-4.jpg; associatedOccurrences: none; associatedSequences: COI; **Taxon:** taxonID: I13_80; scientificNameID: Austinograearodriguezensis; taxonConceptID: Austinograearodriguezensis; scientificName: Austinograearodriguezensis; kingdom: Animalia; phylum: Arthropoda; class: Malacostraca; order: Decapoda; family: Bythograeidae; genus: Austinograea; taxonRank: Species; scientificNameAuthorship: Tsuchida & Hashimoto, 2002; **Location:** waterBody: Indian Ocean; stateProvince: Rodriguez Triple Junction; locality: Kairei; verbatimLocality: Cluster 5; maximumDepthInMeters: 2424; locationRemarks: FS Sonne Cruise INDEX2013 Leg 2; decimalLatitude: -25.3203; decimalLongitude: 70.0404; geodeticDatum: WGS84; coordinateUncertaintyInMeters: 24; **Identification:** identifiedBy: Magdalini Christodoulou, Terue C. Kihara; identificationRemarks: Identified by morphology and DNA of collected specimen; **Event:** eventDate: 2013-12-06; eventTime: 10:57:36 am; year: 2013; fieldNumber: INDEX2013-31ROV; fieldNotes: 2°C; **Record Level:** language: en; institutionCode: DZMB; collectionCode: I13_31RO_SG2_1; datasetName: INDEX; basisOfRecord: Human Observation

##### Notes

Fig. 22

#### Caridea

Dana, 1852

8992362B-ACFD-5A13-BB0E-C9BA13248007

#### Alvinocaridae

Christoffersen, 1986

51386804-B5A1-5BCB-B01A-2601024C684C

#### Alvinocaris

Williams & Chace, 1982

7FD1F4B3-9820-55A2-9BB9-D6E3571E3107

#### Alvinocaris solitaire sp. inc.

Yahagi, Watanabe, Kojima & Beedesse, 2014

DE46F4F9-2DB8-581D-9761-635EFE56F7D5

##### Materials

- **Type status:** Other material. **Occurrence:** recordedBy: ROPOS.COM; individualCount: 100; lifeStage: Adult; behavior: moving on seafloor; occurrenceStatus: present; preparations: Imaged only; associatedMedia: R2106_00061.jpg; **Taxon:** taxonConceptID: Alvinocarissolitaire sp. inc.; scientificName: Alvinocarissolitaire; kingdom: Animalia; phylum: Arthropoda; class: Malacostraca; order: Decapoda; family: Alvinocarididae; genus: Alvinocaris; taxonRank: Species; scientificNameAuthorship: Yahagi, Watanabe, Kojima & Beedesse, 2014; **Location:** waterBody: Indian Ocean; stateProvince: Rodriguez Triple Junction; locality: Vent site 4; verbatimLocality: Cluster 5; maximumDepthInMeters: 2631; locationRemarks: RV Pelagia Cruise INDEX2018 Leg 2; geodeticDatum: WGS84; coordinateUncertaintyInMeters: 23; **Identification:** identifiedBy: Magdalini Christodoulou, Terue C. Kihara; identificationRemarks: Identified only from imagery; identificationQualifier: sp. inc.; **Event:** eventDate: 2018-12-10; eventTime: 5:59:55 am; year: 2018; fieldNumber: INDEX2018-97ROPOS; fieldNotes: 1.8°C, 34.7 ppt; **Record Level:** language: en; institutionCode: DZMB; datasetName: INDEX; basisOfRecord: Human Observation

##### Notes

Fig. 23

#### Mirocaris

Vereshchaka, 1997

3A813518-B457-53D5-A859-9A5017B9A9FB

#### Mirocaris indica sp. inc.

Komai, Martin, Zala, Tsuchida & Hashimoto, 2006

2085D1A9-31CF-574F-9C15-6275B73496FA

##### Materials

- **Type status:** Other material. **Occurrence:** recordedBy: ROPOS.COM; individualCount: 100; lifeStage: Adult; behavior: moving on sulfides; occurrenceStatus: present; preparations: Imaged only; associatedMedia: R1910_00877.jpg; **Taxon:** taxonConceptID: Mirocarisindica sp. inc.; scientificName: Mirocarisindica; kingdom: Animalia; phylum: Arthropoda; class: Malacostraca; order: Decapoda; family: Alvinocarididae; genus: Mirocaris; taxonRank: Species; scientificNameAuthorship: Komai, Martin, Zala, Tsuchida & Hashimoto, 2006; **Location:** waterBody: Indian Ocean; stateProvince: Central Indian Ridge; locality: EGS; verbatimLocality: Cluster 4; maximumDepthInMeters: 3270; locationRemarks: RV Pelagia Cruise INDEX2015 Leg 2; geodeticDatum: WGS84; coordinateUncertaintyInMeters: 32; **Identification:** identifiedBy: Magdalini Christodoulou, Terue C. Kihara; identificationRemarks: Identified only from imagery; identificationQualifier: sp. inc.; **Event:** eventDate: 2015-12-03; eventTime: 7:08:32 am; year: 2015; fieldNumber: INDEX2015-51ROV; **Record Level:** language: en; institutionCode: DZMB; datasetName: INDEX; basisOfRecord: Human Observation

##### Notes

Fig. 24

#### Rimicaris

Williams & Rona, 1986

DAC18AAD-BD8F-5A28-8286-7328F7ABA901

#### Rimicaris kairei

Watabe & Hashimoto, 2002

316E22FB-1799-5294-B4DF-BA5637B75BBC

##### Materials

- **Type status:** Other material. **Occurrence:** recordedBy: ROPOS.COM; individualCount: 100; lifeStage: Adult; behavior: moving on active chimney; occurrenceStatus: present; preparations: DNA voucher and animal stored freeze dried; associatedMedia: R2105_00156.jpg; associatedOccurrences: Bacteria; associatedSequences: COI; **Taxon:** taxonID: I18_1337; scientificNameID: Rimicariskairei; taxonConceptID: Rimicariskairei; scientificName: Rimicariskairei; kingdom: Animalia; phylum: Arthropoda; class: Malacostraca; order: Decapoda; family: Alvinocarididae; genus: Rimicaris; taxonRank: Species; scientificNameAuthorship: Watabe & Hashimoto, 2002; **Location:** waterBody: Indian Ocean; stateProvince: Rodriguez Triple Junction; locality: Vent site 4; verbatimLocality: Cluster 5; maximumDepthInMeters: 2629; locationRemarks: RV Pelagia Cruise INDEX2018 Leg 2; geodeticDatum: WGS84; coordinateUncertaintyInMeters: 26; **Identification:** identifiedBy: Magdalini Christodoulou, Terue C. Kihara; identificationRemarks: Identified by morphology and DNA of collected specimen; **Event:** eventDate: 2018-12-09; eventTime: 7:16:44 am; year: 2018; fieldNumber: INDEX2018-95ROPOS; fieldNotes: 2.4°C, 34.6 ppt; **Record Level:** language: en; institutionCode: DZMB; collectionCode: I18_095RO_SG1_002; datasetName: INDEX; basisOfRecord: Human Observation

##### Notes

Fig. 25

#### Nematocarcinidae

Smith, 1884

3971EF21-C529-5A08-98A6-1AB31B3576BD

#### Nematocarcinus

A. Milne-Edwards, 1881

0DD53059-D738-5B7B-ADAB-0B815F61E0FC

#### Nematocarcinus gen. inc. (DZMB_2021_0004)

53A992D5-B9B7-5412-8943-14EA67FDC44B

##### Materials

- **Type status:** Other material. **Occurrence:** recordedBy: ROPOS.COM; individualCount: 1; lifeStage: Adult; behavior: on seafloor; occurrenceStatus: present; preparations: Imaged only; associatedMedia: R2103_00288.jpg; **Taxon:** taxonConceptID: Nematocarcinus gen. inc. (DZMB_2021_0004); kingdom: Animalia; phylum: Arthropoda; class: Malacostraca; order: Decapoda; family: Nematocarcinidae; genus: Nematocarcinus; taxonRank: Genus; scientificNameAuthorship: A. Milne-Edwards, 1881; **Location:** waterBody: Indian Ocean; stateProvince: Rodriguez Triple Junction; locality: RTJ; verbatimLocality: Cluster 5; maximumDepthInMeters: 2642; locationRemarks: RV Pelagia Cruise INDEX2018 Leg 2; geodeticDatum: WGS84; coordinateUncertaintyInMeters: 25; **Identification:** identifiedBy: Magdalini Christodoulou, Terue C. Kihara; identificationRemarks: Identified only from imagery; identificationQualifier: gen. inc.; **Event:** eventDate: 2018-12-03; eventTime: 12:10:26 pm; year: 2018; fieldNumber: INDEX2018-82ROPOS; fieldNotes: 1.8°C, 34.7 ppt; **Record Level:** language: en; institutionCode: DZMB; datasetName: INDEX; basisOfRecord: Human Observation

##### Notes

Fig. 26

#### Nematocarcinus gen. inc. (DZMB_2021_0005)

25031A1B-07C3-5A3F-A41D-BA91A387D474

##### Materials

- **Type status:** Other material. **Occurrence:** recordedBy: ROPOS.COM; individualCount: 1; lifeStage: Adult; behavior: on sulfides; occurrenceStatus: present; preparations: Imaged only; associatedMedia: R1911_01051.jpg; **Taxon:** taxonConceptID: Nematocarcinus gen. inc. (DZMB_2021_0005); kingdom: Animalia; phylum: Arthropoda; class: Malacostraca; order: Decapoda; family: Nematocarcinidae; genus: Nematocarcinus; taxonRank: Genus; scientificNameAuthorship: A. Milne-Edwards, 1881; **Location:** waterBody: Indian Ocean; stateProvince: Central Indian Ridge; locality: EGS; verbatimLocality: Cluster 4; maximumDepthInMeters: 3075; locationRemarks: RV Pelagia Cruise INDEX2015 Leg 2; geodeticDatum: WGS84; coordinateUncertaintyInMeters: 30; **Identification:** identifiedBy: Magdalini Christodoulou, Terue C. Kihara; identificationRemarks: Identified only from imagery; identificationQualifier: gen. inc.; **Event:** eventDate: 2015-12-04; eventTime: 6:16:41 am; year: 2015; fieldNumber: INDEX2015-53ROV; fieldNotes: 1.8°C, 34.7 ppt; **Record Level:** language: en; institutionCode: DZMB; datasetName: INDEX; basisOfRecord: Human Observation

##### Notes

Fig. 27

#### Dendrobranchiata

Bate, 1888

ED9995A9-4428-571B-A749-36E24205CACD

#### Dendrobranchiata subord. inc.

F66FDB25-F838-5CC6-82DF-DBD832BA0A7D

##### Materials

- **Type status:** Other material. **Occurrence:** recordedBy: ROPOS.COM; individualCount: 1; lifeStage: Adult; behavior: Swimming; occurrenceStatus: present; preparations: Imaged only; associatedMedia: R2100_00098.jpg; **Taxon:** taxonConceptID: Dendrobranchiata subord. inc.; kingdom: Animalia; phylum: Arthropoda; class: Malacostraca; order: Decapoda; taxonRank: Suborder; scientificNameAuthorship: Bate, 1888; **Location:** waterBody: Indian Ocean; stateProvince: South East Indian Ridge; locality: Vent site 5; verbatimLocality: Cluster 11; maximumDepthInMeters: 2913; locationRemarks: RV Pelagia Cruise INDEX2018 Leg 2; geodeticDatum: WGS84; coordinateUncertaintyInMeters: 29; **Identification:** identifiedBy: Magdalini Christodoulou, Terue C. Kihara; identificationRemarks: Identified only from imagery; identificationQualifier: subord. inc.; **Event:** eventDate: 2018-11-28; eventTime: 8:12:11 am; year: 2018; fieldNumber: INDEX2018-73ROPOS; fieldNotes: 1.7°C, 34.7 ppt; **Record Level:** language: en; institutionCode: DZMB; datasetName: INDEX; basisOfRecord: Human Observation

##### Notes

Fig. 28

#### Aristeidae

Wood-Mason in Wood-Mason & Alcock, 1891

A0415C0B-A18D-5439-9AD9-73046666AF4B

#### Cerataspis

Gray, 1828

F5A52F0F-6E2A-535E-949F-F5A168E879FF

#### Cerataspis monstrosus sp. inc.

Gray, 1828

00A0ACC3-CFA6-5BD0-B14B-B01076337900

##### Materials

- **Type status:** Other material. **Occurrence:** recordedBy: ROPOS.COM; individualCount: 1; lifeStage: Adult; behavior: Swimming; occurrenceStatus: present; preparations: Imaged only; associatedMedia: R2095_00024.jpg; **Taxon:** taxonConceptID: Cerataspismonstrosus sp. inc.; scientificName: Cerataspis montrosus; kingdom: Animalia; phylum: Arthropoda; class: Malacostraca; order: Decapoda; family: Aristeidae; genus: Cerataspis; taxonRank: Species; scientificNameAuthorship: Gray, 1828; **Location:** waterBody: Indian Ocean; stateProvince: South East Indian Ridge; locality: Vent site 6; verbatimLocality: Cluster 12; maximumDepthInMeters: 2382; locationRemarks: RV Pelagia Cruise INDEX2018 Leg 2; geodeticDatum: WGS84; coordinateUncertaintyInMeters: 24; **Identification:** identifiedBy: Magdalini Christodoulou, Terue C. Kihara; identificationRemarks: Identified only from imagery; identificationQualifier: sp. inc.; **Event:** eventDate: 2018-11-23; eventTime: 5:48:25 am; year: 2018; fieldNumber: INDEX2018-63ROPOS; **Record Level:** language: en; institutionCode: DZMB; datasetName: INDEX; basisOfRecord: Human Observation

##### Notes

Fig. 29

#### Isopoda

Latreille, 1817

E486FD36-3C46-5A80-A38C-E157CDC43C82

#### Munnopsidae

Lilljeborg, 1864

523E606F-B576-5311-BA11-1DEA9D0F9804

#### Munnopsidae fam. inc. (DZMB_2021_0006)

B33FF604-7C10-53F0-83D6-6D932DC1949A

##### Materials

- **Type status:** Other material. **Occurrence:** recordedBy: ROPOS.COM; individualCount: 100; lifeStage: Adult; behavior: on basalt; occurrenceStatus: present; preparations: DNA voucher and animal stored in 96% ethanol; associatedMedia: R1906_00142.jpg; associatedOccurrences: none; associatedSequences: COI; **Taxon:** taxonID: I15_53; scientificNameID: -; taxonConceptID: Munnopsidae fam. inc. (DZMB_2021_0006); kingdom: Animalia; phylum: Arthropoda; class: Malacostraca; order: Isopoda; family: Munnopsidae; taxonRank: Family; scientificNameAuthorship: Lilljeborg, 1864; **Location:** waterBody: Indian Ocean; stateProvince: Central Indian Ridge; locality: Vent site 1; verbatimLocality: Cluster 4; maximumDepthInMeters: 3036; locationRemarks: RV Pelagia Cruise INDEX2015 Leg 2; geodeticDatum: WGS84; coordinateUncertaintyInMeters: 30; **Identification:** identifiedBy: Simon Bober; identificationRemarks: Identified by morphology and DNA of collected specimen; identificationQualifier: fam. inc.; **Event:** eventDate: 2015-11-29; eventTime: 8:54:59 am; year: 2015; fieldNumber: INDEX2015-43ROV; fieldNotes: 1.8°C, 34.7 ppt; **Record Level:** language: en; institutionCode: DZMB; collectionCode: I15_43RO_D_11; datasetName: INDEX; basisOfRecord: Human Observation

##### Notes

Fig. 30

#### Munnopsidae fam. inc. (DZMB_2021_0007)

42BF8F5B-3036-5D09-BF60-48445C42937C

##### Materials

- **Type status:** Other material. **Occurrence:** recordedBy: ROPOS.COM; individualCount: 100; lifeStage: Adult; behavior: on sulfides; occurrenceStatus: present; preparations: Imaged only; associatedMedia: R2105_00319.jpg; **Taxon:** taxonConceptID: Munnopsidae fam. inc. (DZMB_2021_0007); kingdom: Animalia; phylum: Arthropoda; class: Malacostraca; order: Isopoda; family: Munnopsidae; taxonRank: Family; scientificNameAuthorship: Lilljeborg, 1864; **Location:** waterBody: Indian Ocean; stateProvince: Rodriguez Triple Junction; locality: Vent site 4; verbatimLocality: Cluster 5; maximumDepthInMeters: 2652; locationRemarks: RV Pelagia Cruise INDEX2018 Leg 2; geodeticDatum: WGS84; coordinateUncertaintyInMeters: 26; **Identification:** identifiedBy: Simon Bober; identificationRemarks: Identified only from imagery; identificationQualifier: fam. inc.; **Event:** eventDate: 2018-12-09; eventTime: 9:36:47 am; year: 2018; fieldNumber: INDEX2018-95ROPOS; fieldNotes: 1.8°C, 34.7 ppt; **Record Level:** language: en; institutionCode: DZMB; datasetName: INDEX; basisOfRecord: Human Observation

##### Notes

Fig. 31

#### Pycnogonida

Latreille, 1810

1F53AB23-4228-5241-9FD0-C5B471218FFC

#### Pantopoda

Gerstaecker, 1863

D4C36286-C40A-5689-B2F4-4767ADF2CD7D

#### Pantopoda ord. inc.

F34BBFFD-867C-5FC8-8DE8-A84DF39E5F6C

##### Materials

- **Type status:** Other material. **Occurrence:** recordedBy: ROPOS.COM; individualCount: 1; lifeStage: Adult; behavior: moving at basis of active chimney; occurrenceStatus: present; preparations: Imaged only; associatedMedia: R2100_00194.jpg; **Taxon:** taxonConceptID: Pantopoda ord. inc.; kingdom: Animalia; phylum: Arthropoda; class: Pycnogonida; order: Pantopoda; taxonRank: Order; scientificNameAuthorship: Gerstaecker, 1863; **Location:** waterBody: Indian Ocean; stateProvince: South East Indian Ridge; locality: Vent site 5; verbatimLocality: Cluster 11; maximumDepthInMeters: 2908; locationRemarks: RV Pelagia Cruise INDEX2018 Leg 2; geodeticDatum: WGS84; coordinateUncertaintyInMeters: 29; **Identification:** identifiedBy: Magdalini Christodoulou, Terue C. Kihara; identificationRemarks: Identified only from imagery; identificationQualifier: ord. inc.; **Event:** eventDate: 2018-11-28; eventTime: 10:53:01 am; year: 2018; fieldNumber: INDEX2018-73ROPOS; fieldNotes: 1.7°C, 34.8 ppt; **Record Level:** language: en; institutionCode: DZMB; datasetName: INDEX; basisOfRecord: Human Observation

##### Notes

Fig. 32

#### Bryozoa

0C327041-8F9C-531A-892C-3C723796AF84

#### Gymnolaemata

Allman, 1856

68897D9C-7BE6-5295-BCDB-E432DF2985CD

#### Cheilostomatida

Busk, 1852

06BE8B07-0F77-577E-81CE-FF79A3376E0F

#### Cheilostomatida fam. indet. (DZMB_2021_0008)

33B4EA06-7921-5A13-9614-30F2DBFA12BD

##### Materials

- **Type status:** Other material. **Occurrence:** recordedBy: BGR/ GEOMAR; individualCount: 5; lifeStage: Adult; behavior: attached to basalt; occurrenceStatus: present; preparations: Imaged only; associatedMedia: 2013-12-17_09-26-37_Sonne_INDEX2013-2_062ROV11_Logo-2.jpg; **Taxon:** taxonConceptID: Cheilostomatida fam. indet. (DZMB_2021_0008); kingdom: Animalia; phylum: Bryozoa; class: Gymnolaemata; order: Cheilostomatida; taxonRank: Order; scientificNameAuthorship: Busk, 1852; **Location:** waterBody: Indian Ocean; stateProvince: Central Indian Ridge; locality: MESO; verbatimLocality: outside INDEX claim; maximumDepthInMeters: 2820; locationRemarks: FS Sonne Cruise INDEX2013 Leg 2; geodeticDatum: WGS84; coordinateUncertaintyInMeters: 28; **Identification:** identifiedBy: Dennis Gordon; identificationRemarks: Identified only from imagery; identificationQualifier: fam. indet.; **Event:** eventDate: 2013-12-17; eventTime: 9:26:37 am; year: 2013; fieldNumber: INDEX2013-62ROV; **Record Level:** language: en; institutionCode: DZMB; datasetName: INDEX; basisOfRecord: Human Observation

##### Notes

Fig. 33

#### Cheilostomatida fam. indet. (DZMB_2021_0009)

CA6200C6-5EFE-551F-AAD9-3D62C0AE11B1

##### Materials

- **Type status:** Other material. **Occurrence:** recordedBy: BGR; individualCount: 100; lifeStage: Adult; behavior: attached to basalt; occurrenceStatus: present; preparations: Imaged only; associatedMedia: IMG_5416.jpg; **Taxon:** taxonConceptID: Cheilostomatida fam. indet. (DZMB_2021_0009); kingdom: Animalia; phylum: Bryozoa; class: Gymnolaemata; order: Cheilostomatida; taxonRank: Order; scientificNameAuthorship: Busk, 1852; **Location:** waterBody: Indian Ocean; stateProvince: South East Indian Ridge; locality: Vent site 5; verbatimLocality: Cluster 11; maximumDepthInMeters: 2917; locationRemarks: FS Sonne Cruise INDEX2017 Leg 1; geodeticDatum: WGS84; coordinateUncertaintyInMeters: 29; **Identification:** identifiedBy: Dennis Gordon; identificationRemarks: Identified only from imagery; identificationQualifier: fam. indet.; **Event:** eventDate: 2017-09-27; eventTime: 9:16:24 am; year: 2017; fieldNumber: INDEX2017-94STR; fieldNotes: 1.7°C, 34.7 ppt; **Record Level:** language: en; institutionCode: DZMB; datasetName: INDEX; basisOfRecord: Human Observation

##### Notes

Fig. 34

#### Bifaxariidae

Busk, 1884

69FAA3D6-CA3B-5F06-81DA-02EC09C16276

#### Bifaxaria

Busk, 1884

03836063-6371-5DAA-9ED1-9211D61A48C4

#### Bifaxaria gen. inc.

3C8099AD-B324-596E-A913-8DCA31675E57

##### Materials

- **Type status:** Other material. **Occurrence:** recordedBy: ROPOS.COM; individualCount: 1; lifeStage: Adult; behavior: attached to basalt; occurrenceStatus: present; preparations: Imaged only; associatedMedia: R2101_00131.jpg; **Taxon:** taxonConceptID: Bifaxaria gen. inc.; kingdom: Animalia; phylum: Bryozoa; class: Gymnolaemata; order: Cheilostomatida; family: Bifaxariidae; genus: Bifaxaria; taxonRank: Genus; scientificNameAuthorship: Busk, 1884; **Location:** waterBody: Indian Ocean; stateProvince: South East Indian Ridge; locality: Vent site 5; verbatimLocality: Cluster 11; maximumDepthInMeters: 2909; locationRemarks: RV Pelagia Cruise INDEX2018 Leg 2; geodeticDatum: WGS84; coordinateUncertaintyInMeters: 29; **Identification:** identifiedBy: Dennis Gordon; identificationRemarks: Identified only from imagery; identificationQualifier: gen. inc.; **Event:** eventDate: 2018-11-29; eventTime: 10:01:21 am; year: 2018; fieldNumber: INDEX2018-75ROPOS; fieldNotes: 1.7°C, 34.7 ppt; **Record Level:** language: en; institutionCode: DZMB; datasetName: INDEX; basisOfRecord: Human Observation

##### Notes

Fig. 35

#### Tessaradomidae

Jullien, 1903

5B09B535-A1F5-5B0C-B3A7-35D9593EB60C

#### Tessaradoma

Norman, 1869

3502C18D-ADC6-52B7-9078-1D4847925899

#### Tessaradoma gen. inc.

C3CFF312-ED87-56E7-BEE8-8619B7104724

##### Materials

- **Type status:** Other material. **Occurrence:** recordedBy: BGR/ GEOMAR; individualCount: 1; lifeStage: Adult; behavior: attached to basalt; occurrenceStatus: present; preparations: Imaged only; associatedMedia: 2013-12-15_07-25-31_Sonne_INDEX2013-2_057ROV09_Logo-2.jpg; **Taxon:** taxonConceptID: Tessaradoma gen. inc.; kingdom: Animalia; phylum: Bryozoa; class: Gymnolaemata; order: Cheilostomatida; family: Tessaradomidae; genus: Tessaradoma; taxonRank: Genus; scientificNameAuthorship: Norman, 1869; **Location:** waterBody: Indian Ocean; stateProvince: Central Indian Ridge; locality: MESO; verbatimLocality: outside INDEX claim; maximumDepthInMeters: 2823; locationRemarks: FS Sonne Cruise INDEX2013 Leg 2; geodeticDatum: WGS84; coordinateUncertaintyInMeters: 30; **Identification:** identifiedBy: Dennis Gordon; identificationRemarks: Identified only from imagery; identificationQualifier: gen. inc.; **Event:** eventDate: 2013-12-15; eventTime: 7:25:31 am; year: 2013; fieldNumber: INDEX2013-57ROV; **Record Level:** language: en; institutionCode: DZMB; datasetName: INDEX; basisOfRecord: Human Observation

##### Notes

Fig. 36

#### Chordata

Haeckel, 1874

71652DA1-B8E6-5DDD-819B-B3D6DA94777F

#### Actinopterygii

D3C81712-52CA-5FB9-B741-7F197C1D37B3

#### Anguilliformes

45F22BD8-D9FB-5D8C-920B-AFF968480AF5

#### Synaphobranchidae

Johnson, 1862

BCF72828-27BE-5906-BA63-223771F0DA6F

#### Synaphobranchidae gen. indet.

9737B40F-8F84-587E-9F66-6EC40305FA3A

##### Materials

- **Type status:** Other material. **Occurrence:** recordedBy: ROPOS.COM; individualCount: 1; lifeStage: Adult; behavior: Swimming; occurrenceStatus: present; preparations: Imaged only; associatedMedia: R2097_00220.jpg; **Taxon:** taxonConceptID: Synaphobranchidae gen. indet.; kingdom: Animalia; phylum: Chordata; class: Actinopterygii; order: Anguilliformes; family: Synaphobranchidae; taxonRank: Family; scientificNameAuthorship: Johnson, 1862; **Location:** waterBody: Indian Ocean; stateProvince: South East Indian Ridge; locality: Vent site 6; verbatimLocality: Cluster 12; maximumDepthInMeters: 2479; locationRemarks: RV Pelagia Cruise INDEX2018 Leg 2; geodeticDatum: WGS84; coordinateUncertaintyInMeters: 24; **Identification:** identifiedBy: Thomas D. Linley; identificationRemarks: Identified only from imagery; identificationQualifier: gen. indet.; **Event:** eventDate: 2018-11-25; eventTime: 10:09:43 am; year: 2018; fieldNumber: INDEX2018-67ROPOS; fieldNotes: 1.8°C, 34.7 ppt; **Record Level:** language: en; institutionCode: DZMB; datasetName: INDEX; basisOfRecord: Human Observation

##### Notes

Fig. 37

#### Histiobranchus

Gill, 1883

8CC960FB-F853-5647-92C7-613791B3DF45

#### Histiobranchus gen. inc.

ADF1A65E-2E0D-5B1F-9052-ABF886C31FAF

##### Materials

- **Type status:** Other material. **Occurrence:** recordedBy: ROPOS.COM; individualCount: 1; lifeStage: Adult; behavior: Swimming; occurrenceStatus: present; preparations: Imaged only; associatedMedia: R1905_00044.jpg; **Taxon:** taxonConceptID: Histiobranchus gen. inc.; kingdom: Animalia; phylum: Chordata; class: Actinopterygii; order: Anguilliformes; family: Synaphobranchidae; genus: Histiobranchus; taxonRank: Genus; scientificNameAuthorship: Gill, 1883; **Location:** waterBody: Indian Ocean; stateProvince: Central Indian Ridge; locality: Vent site 1; verbatimLocality: Cluster 4; maximumDepthInMeters: 3025; locationRemarks: RV Pelagia Cruise INDEX2015 Leg 2; geodeticDatum: WGS84; coordinateUncertaintyInMeters: 30; **Identification:** identifiedBy: Thomas D. Linley; identificationRemarks: Identified only from imagery; identificationQualifier: gen. inc.; **Event:** eventDate: 2015-11-27; eventTime: 9:47:09 am; year: 2015; fieldNumber: INDEX2015-37ROV; fieldNotes: 1.8°C, 34.7 ppt; **Record Level:** language: en; institutionCode: DZMB; datasetName: INDEX; basisOfRecord: Human Observation

##### Notes

Fig. 38

#### Ilyophis

Gilbert, 1891

93FA3E9A-093F-5EE0-AA56-C4BCB9F97138

#### Synaphobranchidae Ilyophis brunneus fam. inc.

Gilbert, 1891

49407C4B-8D2D-5ABE-9CDB-159CDCEB80B4

##### Materials

- **Type status:** Other material. **Occurrence:** recordedBy: BGR/ GEOMAR; individualCount: 1; lifeStage: Adult; behavior: Swimming; occurrenceStatus: present; preparations: Imaged only; associatedMedia: 2013-12-05_10-02-51_Sonne_INDEX2013-2_028ROV01_Logo.jpg; **Taxon:** taxonConceptID: Synaphobranchidae Ilyophisbrunneus fam. inc.; scientificName: Ilyophisbrunneus; kingdom: Animalia; phylum: Chordata; class: Actinopterygii; order: Anguilliformes; family: Synaphobranchidae; genus: Ilyophis; taxonRank: Species; scientificNameAuthorship: Gilbert, 1891; **Location:** waterBody: Indian Ocean; stateProvince: Rodriguez Triple Junction; locality: Kairei; verbatimLocality: Cluster 5; maximumDepthInMeters: 2498; locationRemarks: FS Sonne Cruise INDEX2013 Leg 2; geodeticDatum: WGS84; coordinateUncertaintyInMeters: 25; **Identification:** identifiedBy: Thomas D. Linley; identificationRemarks: Identified only from imagery; identificationQualifier: fam. inc.; **Event:** eventDate: 2013-12-05; eventTime: 10:02:51 am; year: 2013; fieldNumber: INDEX2013-28ROV; fieldNotes: 1.8°C; **Record Level:** language: en; institutionCode: DZMB; datasetName: INDEX; basisOfRecord: Human Observation

##### Notes

Fig. 39

#### Aulopiformes

142DA9BA-E2EC-5AF9-83DE-352B89E19E8D

#### Bathysauridae

Fowler, 1944

0B12E476-99BD-5287-BFDC-4EEAFD55B031

#### Bathysaurus

Günther, 1878

9449C1F7-82CA-52FB-9E0B-2D7E76DD67F9

#### Bathysaurus mollis sp. inc.

Günther, 1878

E45E455E-52D5-58D2-A79D-BD3D6B99BA3E

##### Materials

- **Type status:** Other material. **Occurrence:** recordedBy: ROPOS.COM; individualCount: 1; lifeStage: Adult; behavior: on seafloor; occurrenceStatus: present; preparations: Imaged only; associatedMedia: R1907_00295.jpg; **Taxon:** taxonConceptID: Bathysaurusmollis sp. inc.; scientificName: Bathysaurusmollis; kingdom: Animalia; phylum: Chordata; class: Actinopterygii; order: Aulopiformes; family: Bathysauridae; genus: Bathysaurus; taxonRank: Species; scientificNameAuthorship: Günther, 1878; **Location:** waterBody: Indian Ocean; stateProvince: Central Indian Ridge; locality: Vent site 1; verbatimLocality: Cluster 4; maximumDepthInMeters: 3041; locationRemarks: RV Pelagia Cruise INDEX2015 Leg 2; geodeticDatum: WGS84; coordinateUncertaintyInMeters: 30; **Identification:** identifiedBy: Thomas D. Linley; identificationRemarks: Identified only from imagery; identificationQualifier: sp. inc.; **Event:** eventDate: 2015-11-30; eventTime: 8:37:35 am; year: 2015; fieldNumber: INDEX2015-45ROV; fieldNotes: 1.8°C, 34.7 ppt; **Record Level:** language: en; institutionCode: DZMB; datasetName: INDEX; basisOfRecord: Human Observation

##### Notes

Fig. 40

#### Ipnopidae

Gill, 1884

CE55CC88-4E53-54B6-95F5-913903CED17A

#### Bathypterois

Günther, 1878

8AB719AA-7ED6-5C66-A1F9-D412F40A2A0F

#### Bathypterois sp. indet.

84046FE8-7B84-5830-BC13-3040E6ABB099

##### Materials

- **Type status:** Other material. **Occurrence:** recordedBy: ROPOS.COM; individualCount: 1; lifeStage: Adult; behavior: on sediment; occurrenceStatus: present; preparations: Imaged only; associatedMedia: R1914_00197.jpg; **Taxon:** taxonConceptID: Bathypterois sp. indet.; kingdom: Animalia; phylum: Chordata; class: Actinopterygii; order: Aulopiformes; family: Ipnopidae; genus: Bathypterois; taxonRank: Genus; scientificNameAuthorship: Günther, 1878; **Location:** waterBody: Indian Ocean; stateProvince: Central Indian Ridge; locality: EGS; verbatimLocality: Cluster 4; maximumDepthInMeters: 3301; locationRemarks: RV Pelagia Cruise INDEX2015 Leg 2; geodeticDatum: WGS84; coordinateUncertaintyInMeters: 31; **Identification:** identifiedBy: Thomas D. Linley; identificationRemarks: Identified only from imagery; identificationQualifier: sp. indet.; **Event:** eventDate: 2015-12-07; eventTime: 7:29:18 am; year: 2015; fieldNumber: INDEX2015-60ROV; fieldNotes: 1.8°C, 34.7 ppt; **Record Level:** language: en; institutionCode: DZMB; datasetName: INDEX; basisOfRecord: Human Observation

##### Notes

Fig. 41

#### Ipnops

Günther, 1878

64CB1198-6CF2-50B7-B8E5-99DB22A81BE4

#### Ipnops agassizii sp. inc.

Garman, 1899

CDA05A94-A31D-5285-96B0-BF6F2EF4E649

##### Materials

- **Type status:** Other material. **Occurrence:** recordedBy: BGR; individualCount: 1; lifeStage: Adult; behavior: on seafloor; occurrenceStatus: present; preparations: Imaged only; associatedMedia: 38MFT Fotos 2013-284.jpg; **Taxon:** taxonConceptID: Ipnopsagassizii sp. inc.; scientificName: Ipnopsagassizii; kingdom: Animalia; phylum: Chordata; class: Actinopterygii; order: Aulopiformes; family: Ipnopidae; genus: Ipnops; taxonRank: Species; scientificNameAuthorship: Garman, 1899; **Location:** waterBody: Indian Ocean; stateProvince: Central Indian Ridge; locality: Edmond; verbatimLocality: Cluster 4; maximumDepthInMeters: 3284; locationRemarks: FS Sonne Cruise INDEX2013 Leg 2; decimalLatitude: -23.8787; decimalLongitude: 69.6007; geodeticDatum: WGS84; coordinateUncertaintyInMeters: 33; **Identification:** identifiedBy: Thomas D. Linley; identificationRemarks: Identified only from imagery; identificationQualifier: sp. inc.; **Event:** eventDate: 2013-12-09; eventTime: 1:46:37 am; year: 2013; fieldNumber: INDEX2013-38MFT; fieldNotes: 1.8°C, 34.7 ppt; **Record Level:** language: en; institutionCode: DZMB; datasetName: INDEX; basisOfRecord: Human Observation

##### Notes

Fig. 42

#### Gadiformes

52E90FAA-BA27-5D28-9CAA-5AB8380D29D0

#### Macrouridae

Bonaparte, 1831

4DF411FD-FFAC-539F-AA60-1C5E15284EA3

#### Gadiformes Macrouridae ord. inc. (DZMB_2021_0010)

A3133E5D-41EB-5FD1-AA3A-AA5F2252DA67

##### Materials

- **Type status:** Other material. **Occurrence:** recordedBy: ROPOS.COM; individualCount: 1; lifeStage: Adult; behavior: Swimming; occurrenceStatus: present; preparations: Imaged only; associatedMedia: R2093_00612.jpg; **Taxon:** taxonConceptID: Gadiformes Macrouridae ord. inc. (DZMB_2021_0010); kingdom: Animalia; phylum: Chordata; class: Actinopterygii; order: Gadiformes; family: Macrouridae; taxonRank: Family; scientificNameAuthorship: Bonaparte, 1831; **Location:** waterBody: Indian Ocean; stateProvince: South East Indian Ridge; locality: Vent site 6; verbatimLocality: Cluster 12; maximumDepthInMeters: 2544; locationRemarks: RV Pelagia Cruise INDEX2018 Leg 2; geodeticDatum: WGS84; coordinateUncertaintyInMeters: 23; **Identification:** identifiedBy: Thomas D. Linley; identificationRemarks: Identified only from imagery; identificationQualifier: ord. inc.; **Event:** eventDate: 2018-11-21; eventTime: 6:23:52 am; year: 2018; fieldNumber: INDEX2018-59ROPOS; fieldNotes: 1.8°C, 34.7 ppt; **Record Level:** language: en; institutionCode: DZMB; datasetName: INDEX; basisOfRecord: Human Observation

##### Notes

Fig. 43

#### Gadiformes Macrouridae ord. inc. (DZMB_2021_0011)

50ECEED4-3CD3-580D-8C5D-FE01C5F2EAED

##### Materials

- **Type status:** Other material. **Occurrence:** recordedBy: ROPOS.COM; individualCount: 1; lifeStage: Adult; behavior: Swimming; occurrenceStatus: present; preparations: Imaged only; associatedMedia: R2092_00558.jpg; **Taxon:** taxonConceptID: Gadiformes Macrouridae ord. inc. (DZMB_2021_0011); kingdom: Animalia; phylum: Chordata; class: Actinopterygii; order: Gadiformes; family: Macrouridae; taxonRank: Family; scientificNameAuthorship: Bonaparte, 1831; **Location:** waterBody: Indian Ocean; stateProvince: South East Indian Ridge; locality: Vent site 6; verbatimLocality: Cluster 12; maximumDepthInMeters: 2463; locationRemarks: RV Pelagia Cruise INDEX2018 Leg 2; geodeticDatum: WGS84; coordinateUncertaintyInMeters: 25; **Identification:** identifiedBy: Thomas D. Linley; identificationRemarks: Identified only from imagery; identificationQualifier: ord. inc.; **Event:** eventDate: 2018-11-20; eventTime: 10:43:48 am; year: 2018; fieldNumber: INDEX2018-57ROPOS; fieldNotes: 1.8°C, 34.7 ppt; **Record Level:** language: en; institutionCode: DZMB; datasetName: INDEX; basisOfRecord: Human Observation

##### Notes

Fig. 44

#### Coryphaenoides

Gunnerus, 1765

B77BE094-B4A2-518E-9ACB-36410215489E

#### Coryphaenoides gen. inc. (DZMB_2021_0012)

D40933A7-51D5-5ECC-834F-457403453339

##### Materials

- **Type status:** Other material. **Occurrence:** recordedBy: ROPOS.COM; individualCount: 1; lifeStage: Adult; behavior: Swimming; occurrenceStatus: present; preparations: Imaged only; associatedMedia: R2101_00005.jpg; **Taxon:** taxonConceptID: Coryphaenoides gen. inc. (DZMB_2021_0012); kingdom: Animalia; phylum: Chordata; class: Actinopterygii; order: Gadiformes; family: Macrouridae; genus: Coryphaenoides; taxonRank: Genus; scientificNameAuthorship: Gunnerus, 1765; **Location:** waterBody: Indian Ocean; stateProvince: South East Indian Ridge; locality: Vent site 5; verbatimLocality: Cluster 11; maximumDepthInMeters: 2922; locationRemarks: RV Pelagia Cruise INDEX2018 Leg 2; geodeticDatum: WGS84; coordinateUncertaintyInMeters: 29; **Identification:** identifiedBy: Thomas D. Linley; identificationRemarks: Identified only from imagery; identificationQualifier: gen. inc.; **Event:** eventDate: 2018-11-29; eventTime: 5:20:34 am; year: 2018; fieldNumber: INDEX2018-75ROPOS; fieldNotes: 1.7°C, 34.7 ppt; **Record Level:** language: en; institutionCode: DZMB; datasetName: INDEX; basisOfRecord: Human Observation

##### Notes

Fig. 45

#### Coryphaenoides gen. inc. (DZMB_2021_0013)

3734EAE6-A354-5215-B53A-D58F535B4DF1

##### Materials

- **Type status:** Other material. **Occurrence:** recordedBy: ROPOS.COM; individualCount: 1; lifeStage: Adult; behavior: Swimming; occurrenceStatus: present; preparations: Imaged only; associatedMedia: R2092_00403.jpg; **Taxon:** taxonConceptID: Coryphaenoides gen. inc. (DZMB_2021_0013); kingdom: Animalia; phylum: Chordata; class: Actinopterygii; order: Gadiformes; family: Macrouridae; genus: Coryphaenoides; taxonRank: Genus; scientificNameAuthorship: Gunnerus, 1765; **Location:** waterBody: Indian Ocean; stateProvince: South East Indian Ridge; locality: Vent site 6; verbatimLocality: Cluster 12; maximumDepthInMeters: 2462; locationRemarks: RV Pelagia Cruise INDEX2018 Leg 2; geodeticDatum: WGS84; coordinateUncertaintyInMeters: 25; **Identification:** identifiedBy: Thomas D. Linley; identificationRemarks: Identified only from imagery; identificationQualifier: gen. inc.; **Event:** eventDate: 2018-11-20; eventTime: 7:23:30 am; year: 2018; fieldNumber: INDEX2018-57ROPOS; fieldNotes: 1.8°C, 34.7 ppt; **Record Level:** language: en; institutionCode: DZMB; datasetName: INDEX; basisOfRecord: Human Observation

##### Notes

Fig. 46

#### Coryphaenoides armatus sp. inc.

(Hector, 1875)

B2BF9E0E-778A-5A99-9DD8-4B198001632C

##### Materials

- **Type status:** Other material. **Occurrence:** recordedBy: BGR/ GEOMAR; individualCount: 1; lifeStage: Adult; behavior: Swimming; occurrenceStatus: present; preparations: Imaged only; associatedMedia: 2013-12-15_09-28-38_Sonne_INDEX2013-2_057ROV09_Logo.jpg; **Taxon:** taxonConceptID: Coryphaenoidesarmatus sp. inc.; scientificName: Coryphaenoidesarmatus; kingdom: Animalia; phylum: Chordata; class: Actinopterygii; order: Gadiformes; family: Macrouridae; genus: Coryphaenoides; taxonRank: Species; scientificNameAuthorship: (Hector, 1875); **Location:** waterBody: Indian Ocean; stateProvince: Central Indian Ridge; locality: MESO; verbatimLocality: outside INDEX claim; maximumDepthInMeters: 2816; locationRemarks: FS Sonne Cruise INDEX2013 Leg 2; geodeticDatum: WGS84; coordinateUncertaintyInMeters: 30; **Identification:** identifiedBy: Thomas D. Linley; identificationRemarks: Identified only from imagery; identificationQualifier: sp. inc.; **Event:** eventDate: 2013-12-15; eventTime: 9:28:38 am; year: 2013; fieldNumber: INDEX2013-57ROV; **Record Level:** language: en; institutionCode: DZMB; datasetName: INDEX; basisOfRecord: Human Observation

##### Notes

Fig. 47

#### Coryphaenoides longifilis sp. inc.

Günther, 1877

42F926A6-DAEB-59F9-9C5D-D4E5FAE86E78

##### Materials

- **Type status:** Other material. **Occurrence:** recordedBy: BGR/ GEOMAR; individualCount: 1; lifeStage: Adult; behavior: Swimming; occurrenceStatus: present; preparations: Imaged only; associatedMedia: 2013-12-05_09-26-25_Sonne_INDEX2013-2_028ROV01_Logo.jpg; **Taxon:** taxonConceptID: Coryphaenoideslongifilis sp. inc.; scientificName: Coryphaenoideslongifilis; kingdom: Animalia; phylum: Chordata; class: Actinopterygii; order: Gadiformes; family: Macrouridae; genus: Coryphaenoides; taxonRank: Species; scientificNameAuthorship: Günther, 1877; **Location:** waterBody: Indian Ocean; stateProvince: Rodriguez Triple Junction; locality: Kairei; verbatimLocality: Cluster 5; maximumDepthInMeters: 2554; locationRemarks: FS Sonne Cruise INDEX2013 Leg 2; geodeticDatum: WGS84; coordinateUncertaintyInMeters: 25; **Identification:** identifiedBy: Thomas D. Linley; identificationRemarks: Identified only from imagery; identificationQualifier: sp. inc.; **Event:** eventDate: 2013-12-05; eventTime: 9:26:25 am; year: 2013; fieldNumber: INDEX2013-28ROV; fieldNotes: 1.8°C; **Record Level:** language: en; institutionCode: DZMB; datasetName: INDEX; basisOfRecord: Human Observation

##### Notes

Fig. 48

#### Moridae

Moreau, 1881

C5EFC856-E3B9-53EE-9935-5551D4A17AD4

#### Antimora

Günther, 1878

6CC1F9CF-7AE7-59D7-AB35-09699E6E0292

#### Antimora rostrata

(Günther, 1878)

F8CFFF2D-307B-5D89-835F-48187647833C

##### Materials

- **Type status:** Other material. **Occurrence:** recordedBy: ROPOS.COM; individualCount: 1; lifeStage: Adult; behavior: Swimming; occurrenceStatus: present; preparations: Imaged only; associatedMedia: R2106_00291.jpg; **Taxon:** taxonConceptID: Antimorarostrata; scientificName: Antimorarostrata; kingdom: Animalia; phylum: Chordata; class: Actinopterygii; order: Gadiformes; family: Moridae; genus: Antimora; taxonRank: Species; scientificNameAuthorship: (Günther, 1878); **Location:** waterBody: Indian Ocean; stateProvince: Rodriguez Triple Junction; locality: Vent site 4; verbatimLocality: Cluster 5; maximumDepthInMeters: 2281; locationRemarks: RV Pelagia Cruise INDEX2018 Leg 2; geodeticDatum: WGS84; coordinateUncertaintyInMeters: 23; **Identification:** identifiedBy: Thomas D. Linley; identificationRemarks: Identified only from imagery; **Event:** eventDate: 2018-12-10; eventTime: 12:22:53 pm; year: 2018; fieldNumber: INDEX2018-97ROPOS; fieldNotes: 1.8°C, 34.7 ppt; **Record Level:** language: en; institutionCode: DZMB; datasetName: INDEX; basisOfRecord: Human Observation

##### Notes

Fig. 49

#### Lophiiformes

EC6BECEC-F8D5-5BC9-8C0D-D820DB724995

#### Chaunacidae

Gill, 1863

68F85CC3-C371-5CFF-8F9B-68C66870E511

#### Chaunacops

Garman, 1899

C960CF55-B68F-5F74-A449-7FA4B791FE6C

#### Chaunacops gen. inc.

5E60576F-75D6-5371-8D2D-DCB188F594CF

##### Materials

- **Type status:** Other material. **Occurrence:** recordedBy: ROPOS.COM; lifeStage: Adult; behavior: Swimming; occurrenceStatus: present; preparations: Imaged only; associatedMedia: R2101_00051.jpg; **Taxon:** taxonConceptID: Chaunacops gen. inc.; kingdom: Animalia; phylum: Chordata; class: Actinopterygii; order: Lophiiformes; family: Chaunacidae; genus: Chaunacops; taxonRank: Genus; scientificNameAuthorship: Garman, 1899; **Location:** waterBody: Indian Ocean; stateProvince: South East Indian Ridge; locality: Vent site 5; verbatimLocality: Cluster 11; maximumDepthInMeters: 2922; locationRemarks: RV Pelagia Cruise INDEX2018 Leg 2; geodeticDatum: WGS84; coordinateUncertaintyInMeters: 29; **Identification:** identifiedBy: Thomas D. Linley; identificationRemarks: Identified only from imagery; identificationQualifier: gen. inc.; **Event:** eventDate: 2018-11-29; eventTime: 6:55:14 am; year: 2018; fieldNumber: INDEX2018-75ROPOS; fieldNotes: 1.7°C, 34.7 ppt; **Record Level:** language: en; institutionCode: DZMB; datasetName: INDEX; basisOfRecord: Human Observation

##### Notes

Fig. 50

#### Notacanthiformes

L. S. Berg, 1947

4E21F549-65F5-5C6E-91BB-5082A58A34C4

#### Notacanthiformes ord. inc.

4086EF17-A810-548B-987E-AC3F7CB9835A

##### Materials

- **Type status:** Other material. **Occurrence:** recordedBy: BGR; individualCount: 1; lifeStage: Adult; behavior: Swimming; occurrenceStatus: present; preparations: Imaged only; associatedMedia: 44MFT Fotos 2013-298.jpg; **Taxon:** taxonConceptID: Notacanthiformes ord. inc.; kingdom: Animalia; phylum: Chordata; class: Actinopterygii; order: Notacanthiformes; taxonRank: Order; scientificNameAuthorship: L. S. Berg, 1947; **Location:** waterBody: Indian Ocean; stateProvince: Central Indian Ridge; locality: Edmond; verbatimLocality: Cluster 4; maximumDepthInMeters: 3252; locationRemarks: FS Sonne Cruise INDEX2013 Leg 2; decimalLatitude: -23.8769; decimalLongitude: 69.6009; geodeticDatum: WGS84; coordinateUncertaintyInMeters: 33; **Identification:** identifiedBy: Thomas D. Linley; identificationRemarks: Identified only from imagery; identificationQualifier: ord. inc.; **Event:** eventDate: 2013-12-10; eventTime: 10:41:13 pm; year: 2013; fieldNumber: INDEX2013-44MFT; fieldNotes: 1.8°C, 34.7 ppt; **Record Level:** language: en; institutionCode: DZMB; datasetName: INDEX; basisOfRecord: Human Observation

##### Notes

Fig. 51

#### Halosauridae

Günther, 1868

C0DB4E09-FE4F-5B47-8612-E67707612116

#### Aldrovandia

Goode & Bean, 1896

098F631B-829D-59AD-BFB3-CAA53DE4A163

#### Aldrovandia affinis gen. inc.

(Günther, 1877)

B1F1BD28-8453-5F6E-911B-D4F059BB2B45

##### Materials

- **Type status:** Other material. **Occurrence:** recordedBy: ROPOS.COM; individualCount: 1; lifeStage: Adult; behavior: Swimming; occurrenceStatus: present; preparations: Imaged only; associatedMedia: R2097_00204.jpg; **Taxon:** taxonConceptID: Aldrovandiaaffinis gen. inc.; scientificName: Aldrovandiaaffinis; kingdom: Animalia; phylum: Chordata; class: Actinopterygii; order: Notacanthiformes; family: Halosauridae; genus: Aldrovandia; taxonRank: Species; scientificNameAuthorship: (Günther, 1877); **Location:** waterBody: Indian Ocean; stateProvince: South East Indian Ridge; locality: Vent site 6; verbatimLocality: Cluster 12; maximumDepthInMeters: 2494; locationRemarks: RV Pelagia Cruise INDEX2018 Leg 2; geodeticDatum: WGS84; coordinateUncertaintyInMeters: 24; **Identification:** identifiedBy: Thomas D. Linley; identificationRemarks: Identified only from imagery; identificationQualifier: gen. inc.; **Event:** eventDate: 2018-11-25; eventTime: 10:02:00 am; year: 2018; fieldNumber: INDEX2018-67ROPOS; fieldNotes: 1.8°C, 34.7 ppt; **Record Level:** language: en; institutionCode: DZMB; datasetName: INDEX; basisOfRecord: Human Observation

##### Notes

Fig. 52

#### Halosauropsis

Collett, 1896

7496EDA1-F4E8-5155-A5E4-F76ADE76AA53

#### Halosauropsis macrochir gen. inc.

(Günther, 1878)

F3985924-1228-5E64-BD5D-46DDF71B7472

##### Materials

- **Type status:** Other material. **Occurrence:** recordedBy: ROPOS.COM; individualCount: 1; lifeStage: Adult; behavior: Swimming; occurrenceStatus: present; preparations: Imaged only; associatedMedia: R2103_00196.jpg; **Taxon:** taxonConceptID: Halosauropsismacrochir gen. inc.; scientificName: Halosauropsismacrochir; kingdom: Animalia; phylum: Chordata; class: Actinopterygii; order: Notacanthiformes; family: Halosauridae; genus: Halosauropsis; taxonRank: Species; scientificNameAuthorship: (Günther, 1878); **Location:** waterBody: Indian Ocean; stateProvince: Rodriguez Triple Junction; locality: RTJ; verbatimLocality: Cluster 5; maximumDepthInMeters: 2431; locationRemarks: RV Pelagia Cruise INDEX2018 Leg 2; geodeticDatum: WGS84; coordinateUncertaintyInMeters: 25; **Identification:** identifiedBy: Thomas D. Linley; identificationRemarks: Identified only from imagery; identificationQualifier: gen. inc.; **Event:** eventDate: 2018-12-03; eventTime: 9:26:58 am; year: 2018; fieldNumber: INDEX2018-82ROPOS; fieldNotes: 1.8°C, 34.7 ppt; **Record Level:** language: en; institutionCode: DZMB; datasetName: INDEX; basisOfRecord: Human Observation

##### Notes

Fig. 53

#### Ophidiiformes

C29526B0-A795-5ADE-880F-C305B6AA4790

#### Ophidiidae

Rafinesque, 1810

2D981FBA-9379-5033-9F63-63EB82E75F0C

#### Ophidiidae gen. indet. (DZMB_2021_0014)

AA8730BE-B143-5F71-B3F7-5C8DD739DCD0

##### Materials

- **Type status:** Other material. **Occurrence:** recordedBy: ROPOS.COM; individualCount: 1; lifeStage: Adult; behavior: Swimming; occurrenceStatus: present; preparations: Imaged only; associatedMedia: R2097_00050.jpg; **Taxon:** taxonConceptID: Ophidiidae gen. indet. (DZMB_2021_0014); kingdom: Animalia; phylum: Chordata; class: Actinopterygii; order: Ophidiiformes; family: Ophidiidae; taxonRank: Family; scientificNameAuthorship: Rafinesque, 1810; **Location:** waterBody: Indian Ocean; stateProvince: South East Indian Ridge; locality: Vent site 6; verbatimLocality: Cluster 12; maximumDepthInMeters: 2347; locationRemarks: RV Pelagia Cruise INDEX2018 Leg 2; geodeticDatum: WGS84; coordinateUncertaintyInMeters: 24; **Identification:** identifiedBy: Thomas D. Linley; identificationRemarks: Identified only from imagery; identificationQualifier: gen. indet.; **Event:** eventDate: 2018-11-25; eventTime: 6:17:39 am; year: 2018; fieldNumber: INDEX2018-67ROPOS; **Record Level:** language: en; institutionCode: DZMB; datasetName: INDEX; basisOfRecord: Human Observation

##### Notes

Fig. 54

#### Ophidiidae gen. indet. (DZMB_2021_0015)

C5EDE247-1AE5-5247-9FC9-3D8F7182BE11

##### Materials

- **Type status:** Other material. **Occurrence:** recordedBy: ROPOS.COM; individualCount: 1; lifeStage: Adult; behavior: Swimming; occurrenceStatus: present; preparations: Imaged only; associatedMedia: R2103_00080.jpg; **Taxon:** taxonConceptID: Ophidiidae gen. indet. (DZMB_2021_0015); kingdom: Animalia; phylum: Chordata; class: Actinopterygii; order: Ophidiiformes; family: Ophidiidae; taxonRank: Family; scientificNameAuthorship: Rafinesque, 1810; **Location:** waterBody: Indian Ocean; stateProvince: Rodriguez Triple Junction; locality: RTJ; verbatimLocality: Cluster 5; maximumDepthInMeters: 2415; locationRemarks: RV Pelagia Cruise INDEX2018 Leg 2; geodeticDatum: WGS84; coordinateUncertaintyInMeters: 25; **Identification:** identifiedBy: Thomas D. Linley; identificationRemarks: Identified only from imagery; identificationQualifier: gen. indet.; **Event:** eventDate: 2018-12-03; eventTime: 6:11:25 am; year: 2018; fieldNumber: INDEX2018-82ROPOS; fieldNotes: 1.8°C, 34.7 ppt; **Record Level:** language: en; institutionCode: DZMB; datasetName: INDEX; basisOfRecord: Human Observation

##### Notes

Fig. 55

#### Ophidiidae fam. inc. (DZMB_2021_0016)

4EFCC132-7ADD-5AB4-A9B9-DD33F32517FC

##### Materials

- **Type status:** Other material. **Occurrence:** recordedBy: IFREMER; individualCount: 1; lifeStage: Adult; behavior: Swimming; occurrenceStatus: present; preparations: Imaged only; associatedMedia: 160109121510914_02_1080i Kopie.jpg; **Taxon:** taxonConceptID: Ophidiidae fam. inc. (DZMB_2021_0016); kingdom: Animalia; phylum: Chordata; class: Actinopterygii; order: Ophidiiformes; family: Ophidiidae; taxonRank: Family; scientificNameAuthorship: Rafinesque, 1810; **Location:** waterBody: Indian Ocean; stateProvince: Rodriguez Triple Junction; locality: Kairei; verbatimLocality: Cluster 5; maximumDepthInMeters: 2687; locationRemarks: RV Pourqoui pas? Cruise INDEX2016 Leg 1; geodeticDatum: WGS84; coordinateUncertaintyInMeters: 28; **Identification:** identifiedBy: Thomas D. Linley; identificationRemarks: Identified only from imagery; identificationQualifier: fam. inc.; **Event:** eventDate: 2016-01-09; eventTime: 12:15:10 pm; year: 2016; fieldNumber: INDEX2016-02ROV; fieldNotes: 1.8°C, 34.7 ppt; **Record Level:** language: en; institutionCode: DZMB; datasetName: INDEX; basisOfRecord: Human Observation

##### Notes

Fig. 56

#### Acanthonus

Günther, 1878

6B93D99A-14CA-5A71-A967-5C59A8F77F71

#### Acanthonus armatus gen. inc.

Günther, 1878

44759D3A-E6DC-5E64-B019-5EE03EA78061

##### Materials

- **Type status:** Other material. **Occurrence:** recordedBy: ROPOS.COM; individualCount: 1; lifeStage: Adult; behavior: Swimming; occurrenceStatus: present; preparations: Imaged only; associatedMedia: R2093_00611.jpg; **Taxon:** taxonConceptID: Acanthonusarmatus gen. inc.; scientificName: Acanthonusarmatus; kingdom: Animalia; phylum: Chordata; class: Actinopterygii; order: Ophidiiformes; family: Ophidiidae; genus: Acanthonus; taxonRank: Species; scientificNameAuthorship: Günther, 1878; **Location:** waterBody: Indian Ocean; stateProvince: South East Indian Ridge; locality: Vent site 6; verbatimLocality: Cluster 12; maximumDepthInMeters: 2544; locationRemarks: RV Pelagia Cruise INDEX2018 Leg 2; geodeticDatum: WGS84; coordinateUncertaintyInMeters: 23; **Identification:** identifiedBy: Thomas D. Linley; identificationRemarks: Identified only from imagery; identificationQualifier: gen. inc.; **Event:** eventDate: 2018-11-21; eventTime: 6:20:48 am; year: 2018; fieldNumber: INDEX2018-59ROPOS; fieldNotes: 1.8°C, 34.7 ppt; **Record Level:** language: en; institutionCode: DZMB; datasetName: INDEX; basisOfRecord: Human Observation

##### Notes

Fig. 57

#### Barathrites

Zugmayer, 1911

651E0B57-F6BB-5FCF-BE4A-3AC7D0B3D015

#### Barathrites iris sp. inc.

Zugmayer, 1911

225594EF-80B6-5FAD-B517-197BF75071BE

##### Materials

- **Type status:** Other material. **Occurrence:** recordedBy: ROPOS.COM; individualCount: 1; lifeStage: Adult; behavior: Swimming; occurrenceStatus: present; preparations: Imaged only; associatedMedia: R1909_00549.jpg; **Taxon:** taxonConceptID: Barathritesiris gen. inc.; scientificName: Barathritesiris; kingdom: Animalia; phylum: Chordata; class: Actinopterygii; order: Ophidiiformes; family: Ophidiidae; genus: Barathrites; taxonRank: Species; scientificNameAuthorship: Zugmayer, 1911; **Location:** waterBody: Indian Ocean; stateProvince: Central Indian Ridge; locality: Vent site 1; verbatimLocality: Cluster 4; maximumDepthInMeters: 3039; locationRemarks: RV Pelagia Cruise INDEX2015 Leg 2; geodeticDatum: WGS84; coordinateUncertaintyInMeters: 30; **Identification:** identifiedBy: Thomas D. Linley; identificationRemarks: Identified only from imagery; identificationQualifier: gen. inc.; **Event:** eventDate: 2015-12-02; eventTime: 7:57:31 am; year: 2015; fieldNumber: INDEX2015-49ROV; fieldNotes: 1.8°C, 34.7 ppt; **Record Level:** language: en; institutionCode: DZMB; datasetName: INDEX; basisOfRecord: Human Observation

##### Notes

Fig. 58

#### Bassozetus

Gill, 1883

7890BC87-A2BF-5E98-B720-590F6CCCF6BD

#### Bassozetus gen. inc.

A7A4CD6B-390B-53E6-B0B3-4643C678F3D3

##### Materials

- **Type status:** Other material. **Occurrence:** recordedBy: ROPOS.COM; individualCount: 1; lifeStage: Adult; behavior: Swimming; occurrenceStatus: present; preparations: Imaged only; associatedMedia: R1913_01969.jpg; **Taxon:** taxonConceptID: Bassozetus gen. inc.; kingdom: Animalia; phylum: Chordata; class: Actinopterygii; order: Ophidiiformes; family: Ophidiidae; genus: Bassozetus; taxonRank: Genus; scientificNameAuthorship: Gill, 1883; **Location:** waterBody: Indian Ocean; stateProvince: Central Indian Ridge; locality: EGS; verbatimLocality: Cluster 4; maximumDepthInMeters: 3314; locationRemarks: RV Pelagia Cruise INDEX2015 Leg 2; geodeticDatum: WGS84; coordinateUncertaintyInMeters: 33; **Identification:** identifiedBy: Thomas D. Linley; identificationRemarks: Identified only from imagery; identificationQualifier: gen. inc.; **Event:** eventDate: 2015-12-06; eventTime: 11:05:00 am; year: 2015; fieldNumber: INDEX2015-58ROV; fieldNotes: 1.9°C, 34.7 ppt; **Record Level:** language: en; institutionCode: DZMB; datasetName: INDEX; basisOfRecord: Human Observation

##### Notes

Fig. 59

#### Spectrunculus

Jordan & Thompson, 1914

D5564305-523E-55A2-BF3B-9FC27ED4DE05

#### Spectrunculus crassus sp. inc.

(Vaillant, 1888)

590EE7FC-F3DB-56FB-89FA-A205A151801A

##### Materials

- **Type status:** Other material. **Occurrence:** recordedBy: ROPOS.COM; individualCount: 1; lifeStage: Adult; behavior: Swimming; occurrenceStatus: present; preparations: Imaged only; associatedMedia: R2096_00237.jpg; **Taxon:** taxonConceptID: Spectrunculuscrassus sp. inc.; scientificName: Spectrunculuscrassus; kingdom: Animalia; phylum: Chordata; class: Actinopterygii; order: Ophidiiformes; family: Ophidiidae; genus: Spectrunculus; taxonRank: Species; scientificNameAuthorship: (Vaillant, 1888); **Location:** waterBody: Indian Ocean; stateProvince: South East Indian Ridge; locality: Vent site 6; verbatimLocality: Cluster 12; maximumDepthInMeters: 2462; locationRemarks: RV Pelagia Cruise INDEX2018 Leg 2; geodeticDatum: WGS84; coordinateUncertaintyInMeters: 24; **Identification:** identifiedBy: Thomas D. Linley; identificationRemarks: Identified only from imagery; identificationQualifier: sp. inc.; **Event:** eventDate: 2018-11-24; eventTime: 10:27:36 am; year: 2018; fieldNumber: INDEX2018-65ROPOS; **Record Level:** language: en; institutionCode: DZMB; datasetName: INDEX; basisOfRecord: Human Observation

##### Notes

Fig. 60

#### Spectrunculus grandis sp. inc.

(Günther, 1877)

CFE3D55D-A52B-5733-A414-3885149F09A5

##### Materials

- **Type status:** Other material. **Occurrence:** recordedBy: ROPOS.COM; individualCount: 1; lifeStage: Adult; behavior: Swimming; occurrenceStatus: present; preparations: Imaged only; associatedMedia: R2102_00053.jpg; **Taxon:** taxonConceptID: Spectrunculusgrandis sp. inc.; scientificName: Spectrunculusgrandis; kingdom: Animalia; phylum: Chordata; class: Actinopterygii; order: Ophidiiformes; family: Ophidiidae; genus: Spectrunculus; taxonRank: Species; scientificNameAuthorship: (Günther, 1877); **Location:** waterBody: Indian Ocean; stateProvince: South East Indian Ridge; locality: Vent site 5; verbatimLocality: Cluster 11; maximumDepthInMeters: 2896; locationRemarks: RV Pelagia Cruise INDEX2018 Leg 2; geodeticDatum: WGS84; coordinateUncertaintyInMeters: 30; **Identification:** identifiedBy: Thomas D. Linley; identificationRemarks: Identified only from imagery; identificationQualifier: sp. inc.; **Event:** eventDate: 2018-12-01; eventTime: 6:36:14 am; year: 2018; fieldNumber: INDEX2018-80ROPOS; fieldNotes: 1.7°C, 34.7 ppt; **Record Level:** language: en; institutionCode: DZMB; datasetName: INDEX; basisOfRecord: Human Observation

##### Notes

Fig. 61

#### Xyelacyba

Cohen, 1961

83422649-AEDD-58B0-9A7C-CD58BBECB625

#### Xyelacyba myersi gen. inc.

Cohen, 1961

86D41E7A-5369-5162-AE98-844838FAA8CF

##### Materials

- **Type status:** Other material. **Occurrence:** recordedBy: ROPOS.COM; individualCount: 1; lifeStage: Adult; behavior: Swimming; occurrenceStatus: present; preparations: Imaged only; associatedMedia: R2103_00024.jpg; **Taxon:** taxonConceptID: Xyelacybamyersi gen. inc.; scientificName: Xyelacybamyersi; kingdom: Animalia; phylum: Chordata; class: Actinopterygii; order: Ophidiiformes; family: Ophidiidae; genus: Xyelacyba; taxonRank: Species; scientificNameAuthorship: Cohen, 1961; **Location:** waterBody: Indian Ocean; stateProvince: Rodriguez Triple Junction; locality: RTJ; verbatimLocality: Cluster 5; maximumDepthInMeters: 2304; locationRemarks: RV Pelagia Cruise INDEX2018 Leg 2; geodeticDatum: WGS84; coordinateUncertaintyInMeters: 25; **Identification:** identifiedBy: Thomas D. Linley; identificationRemarks: Identified only from imagery; identificationQualifier: gen. inc.; **Event:** eventDate: 2018-12-03; eventTime: 5:10:56 am; year: 2018; fieldNumber: INDEX2018-82ROPOS; fieldNotes: 1.8°C, 34.7 ppt; **Record Level:** language: en; institutionCode: DZMB; datasetName: INDEX; basisOfRecord: Human Observation

##### Notes

Fig. 62

#### Perciformes

D31D9FEF-8ADC-5025-90B6-4F65B9A3A643

#### Zoarcidae

Swainson, 1839

10FB5287-025A-5CFE-B521-EE8E551CAEC6

#### Pachycara

Zugmayer, 1911

A4169F3F-C7D8-5FD4-950C-2642D5468B22

#### Pachycara angeloi

Thiel, Knebelsberger, Kihara & Gerdes, 2021

DAE10BF2-1138-5A75-9BB5-F5784CC7B429

##### Materials

- **Type status:** Other material. **Occurrence:** recordedBy: ROPOS.COM; individualCount: 1; lifeStage: Adult; behavior: Swimming; occurrenceStatus: present; preparations: DNA voucher and animal stored in 96% ethanol; associatedMedia: R2100_00194.jpg; associatedOccurrences: none; associatedSequences: COI; **Taxon:** taxonID: I18_1240; taxonConceptID: Pachycaraangeloi; scientificName: Pachycaraangeloi; kingdom: Animalia; phylum: Chordata; class: Actinopterygii; order: Perciformes; family: Zoarcidae; genus: Pachycara; taxonRank: Species; scientificNameAuthorship: Thiel, Knebelsberger, Kihara & Gerdes, 2021; **Location:** waterBody: Indian Ocean; stateProvince: South East Indian Ridge; locality: Vent site 5; verbatimLocality: Cluster 11; maximumDepthInMeters: 2908; locationRemarks: RV Pelagia Cruise INDEX2018 Leg 2; geodeticDatum: WGS84; coordinateUncertaintyInMeters: 29; **Identification:** identifiedBy: Thomas D. Linley; identificationRemarks: Identified by morphology and DNA of collected specimen; **Event:** eventDate: 2018-11-28; eventTime: 10:53:01 am; year: 2018; fieldNumber: INDEX2018-73ROPOS; fieldNotes: 1.7°C, 34.8 ppt; **Record Level:** language: en; institutionCode: DZMB; collectionCode: I18_073RO_SG1_001; datasetName: INDEX; basisOfRecord: Human Observation

##### Notes

Fig. 63

#### Ascidiacea

Blainville, 1824

447B65B9-28FC-52AE-963D-AFC70386B63B

#### Phlebobranchia

Lahille, 1886

F44C8F21-E038-59B7-804D-F036AEF8675A

#### Octacnemidae

4D5C1888-EB27-57D7-BD71-DC913C1D7681

#### Octacnemidae gen. indet.

D2F8CD2F-254C-5F12-9E4E-14F9793BFB3D

##### Materials

- **Type status:** Other material. **Occurrence:** recordedBy: BGR/ GEOMAR; individualCount: 1; lifeStage: Adult; behavior: attached to basalt; occurrenceStatus: present; preparations: Imaged only; associatedMedia: 2013-12-16_06-40-29_Sonne_INDEX2013-2_059ROV10_Logo.jpg; **Taxon:** taxonConceptID: Octacnemidae gen. indet.; kingdom: Animalia; phylum: Chordata; class: Ascidiacea; order: Phlebobranchia; family: Octacnemidae; taxonRank: Family; **Location:** waterBody: Indian Ocean; stateProvince: Central Indian Ridge; locality: MESO; verbatimLocality: outside INDEX claim; maximumDepthInMeters: 2839; locationRemarks: FS Sonne Cruise INDEX2013 Leg 2; geodeticDatum: WGS84; coordinateUncertaintyInMeters: 28; **Identification:** identifiedBy: Karen Sanamyan; identificationRemarks: Identified only from imagery; identificationQualifier: gen. indet.; **Event:** eventDate: 2013-12-16; eventTime: 6:40:29 am; year: 2013; fieldNumber: INDEX2013-59ROV; **Record Level:** language: en; institutionCode: DZMB; datasetName: INDEX; basisOfRecord: Human Observation

##### Notes

Fig. 64

#### Stoliobranchia

Lahille, 1886

9FD66AA6-7F4E-5B36-A673-FEB650309B5F

#### Pyuridae

Hartmeyer, 1908

8EE349FF-0DDA-5A67-BD13-EBEE0ECAD0A5

#### Culeolus

Herdman, 1881

0E8E2454-9A0F-50C3-B89B-BCD897C9392C

#### Culeolus spp. indet.

1685680F-48AE-5B23-9368-31A4829648E8

##### Materials

- **Type status:** Other material. **Occurrence:** recordedBy: ROPOS.COM; individualCount: 1; lifeStage: Adult; behavior: attached to basalt; occurrenceStatus: present; preparations: Imaged only; associatedMedia: R2104_00106.jpg; **Taxon:** taxonConceptID: Culeolus spp. indet.; kingdom: Animalia; phylum: Chordata; class: Ascidiacea; order: Stolidobranchia; family: Pyuridae; genus: Culeolus; taxonRank: Genus; scientificNameAuthorship: Herdman, 1881; **Location:** waterBody: Indian Ocean; stateProvince: Rodriguez Triple Junction; locality: Vent site 4; verbatimLocality: Cluster 5; maximumDepthInMeters: 2508; locationRemarks: RV Pelagia Cruise INDEX2018 Leg 2; geodeticDatum: WGS84; coordinateUncertaintyInMeters: 25; **Identification:** identifiedBy: Karen Sanamyan; identificationRemarks: Identified only from imagery; identificationQualifier: spp. indet.; **Event:** eventDate: 2018-12-04; eventTime: 8:09:09 am; year: 2018; fieldNumber: INDEX2018-85ROPOS; fieldNotes: 1.8°C, 34.7 ppt; **Record Level:** language: en; institutionCode: DZMB; datasetName: INDEX; basisOfRecord: Human Observation

##### Notes

Fig. 65

#### Elasmobranchii

8F32F011-52D4-5465-B0D2-D5A4AB820F9C

#### Rajiformes

B6FAFAEE-1D82-58C8-9024-7DB6018526B0

#### Arhynchobatidae

Fowler, 1934

F98F0E89-DEAA-5049-85E2-737D8F068D9A

#### Bathyraja

Ishiyama, 1958

E2A91FB3-8819-588A-BA1E-1AEBBC7BC298

#### Bathyraja tunae sp. inc.

Stehmann, 2005

06A7F582-8C58-5864-9508-9C53C8A1FCCC

##### Materials

- **Type status:** Other material. **Occurrence:** recordedBy: ROPOS.COM; lifeStage: Adult; behavior: Swimming; occurrenceStatus: present; preparations: Imaged only; associatedMedia: R2106_00207.jpg; **Taxon:** taxonConceptID: Bathyrajatunae sp. inc.; scientificName: Bathyrajatunae; kingdom: Animalia; phylum: Chordata; class: Elasmobranchii; order: Rajiformes; family: Arhynchobatidae; genus: Bathyraja; taxonRank: Species; scientificNameAuthorship: Stehmann, 2005; **Location:** waterBody: Indian Ocean; stateProvince: Rodriguez Triple Junction; locality: Vent site 4; verbatimLocality: Cluster 5; maximumDepthInMeters: 2482; locationRemarks: RV Pelagia Cruise INDEX2018 Leg 2; geodeticDatum: WGS84; coordinateUncertaintyInMeters: 23; **Identification:** identifiedBy: Thomas D. Linley; identificationRemarks: Identified only from imagery; identificationQualifier: sp. inc.; **Event:** eventDate: 2018-12-10; eventTime: 10:16:47 am; year: 2018; fieldNumber: INDEX2018-97ROPOS; fieldNotes: 1.8°C, 34.7 ppt; **Record Level:** language: en; institutionCode: DZMB; datasetName: INDEX; basisOfRecord: Human Observation

##### Notes

Fig. 66

#### Cnidaria

Hatschek, 1888

0604CA94-7049-5436-8C97-96DF0F761CEC

#### Cnidaria cl. indet.

F90DAC5A-964E-5C88-897E-26D5753C2ACC

##### Materials

- **Type status:** Other material. **Occurrence:** recordedBy: NIOZ; individualCount: 1; lifeStage: Adult; behavior: attached to basalt; occurrenceStatus: present; preparations: Imaged only; associatedMedia: 20141204011100792.jpg; **Taxon:** taxonConceptID: Cnidaria cl. indet.; kingdom: Animalia; phylum: Cnidaria; class: -; order: -; family: -; genus: -; taxonRank: Phylum; scientificNameAuthorship: Hatschek, 1888; **Location:** waterBody: Indian Ocean; stateProvince: South East Indian Ridge; locality: SEIR; verbatimLocality: Cluster 6; maximumDepthInMeters: 3582; locationRemarks: RV Pelagia Cruise INDEX2014 Leg 1; geodeticDatum: WGS84; coordinateUncertaintyInMeters: 37; **Identification:** identifiedBy: Tina Molodtsova; identificationRemarks: Identified only from imagery; identificationQualifier: cl. indet.; **Event:** eventDate: 2014-12-04; eventTime: 1:11:00 am; year: 2014; fieldNumber: INDEX2014-55VS; fieldNotes: 1.8°C; **Record Level:** language: en; institutionCode: DZMB; datasetName: INDEX; basisOfRecord: Human Observation

##### Notes

Fig. 67

#### Anthozoa

Ehrenberg, 1834

08905B76-9C74-5556-BF4E-6899FE5EDB77

#### Ceriantharia

Perrier, 1893

0B681A60-B62B-57BC-B61D-D96F3666CD2F

#### Ceriantharia ord. indet.

71A2A647-6EBF-5FEE-83DD-1C0BDB4F2884

##### Materials

- **Type status:** Other material. **Occurrence:** recordedBy: IFREMER; individualCount: 1; lifeStage: Adult; behavior: attached to hard substrates; occurrenceStatus: present; preparations: Imaged only; associatedMedia: 160111221731A Kopie.jpg; **Taxon:** taxonConceptID: Ceriantharia ord. indet.; kingdom: Animalia; phylum: Cnidaria; class: Anthozoa; order: -; family: -; genus: -; taxonRank: Subclass; scientificNameAuthorship: Perrier, 1893; **Location:** waterBody: Indian Ocean; stateProvince: Rodriguez Triple Junction; locality: Kairei; verbatimLocality: Cluster 5; maximumDepthInMeters: 2148; locationRemarks: RV Pourqoui pas? Cruise INDEX2016 Leg 1; geodeticDatum: WGS84; coordinateUncertaintyInMeters: 25; **Identification:** identifiedBy: Tina Molodtsova; identificationRemarks: Identified only from imagery; identificationQualifier: ord. indet.; **Event:** eventDate: 2016-01-11; eventTime: 6:55:33 am; year: 2016; fieldNumber: INDEX2016-06ROV; fieldNotes: 1.8°C, 34.7 ppt; **Record Level:** language: en; institutionCode: DZMB; datasetName: INDEX; basisOfRecord: Human Observation

##### Notes

Fig. 68

#### Spirularia

den Hartog, 1977

A13FE7D4-7999-5724-BBB8-0C7CA9F2E1AC

#### Spirularia fam. indet.

3242028D-B0B6-5568-9CF4-84C75746472F

##### Materials

- **Type status:** Other material. **Occurrence:** recordedBy: ROPOS.COM; individualCount: 1; lifeStage: Adult; behavior: on sediment; occurrenceStatus: present; preparations: DNA voucher and animal stored in 96% ethanol; associatedMedia: R1914_00139.jpg; associatedSequences: COI; **Taxon:** taxonID: I15_191; scientificNameID: -; taxonConceptID: Spirularia fam. indet.; kingdom: Animalia; phylum: Cnidaria; class: Anthozoa; order: Spirularia; family: -; genus: -; taxonRank: Order; scientificNameAuthorship: den Hartog, 1977; **Location:** waterBody: Indian Ocean; stateProvince: Central Indian Ridge; locality: EGS; verbatimLocality: Cluster 4; maximumDepthInMeters: 3223; locationRemarks: RV Pelagia Cruise INDEX2015 Leg 2; decimalLatitude: -23.9206; decimalLongitude: 69.6157; geodeticDatum: WGS84; coordinateUncertaintyInMeters: 31; **Identification:** identifiedBy: Tina Molodtsova; identificationRemarks: Identified by morphology and DNA of collected specimen; identificationQualifier: fam. indet.; **Event:** eventDate: 2015-12-07; eventTime: 6:25:26 am; year: 2015; fieldNumber: INDEX2015-60ROV; fieldNotes: 1.8°C, 34.7 ppt; **Record Level:** language: en; institutionCode: DZMB; collectionCode: I15_60RO_S_2; datasetName: INDEX; basisOfRecord: Human Observation

##### Notes

Fig. 69

#### Actiniaria

Hertwig, 1882

8CCDBE9A-97AD-5DAA-936D-0CD4D421ED29

#### Actiniaria fam. indet. (DZMB_2021_0017)

BEDD6966-C77F-5980-9723-18E48DFB8984

##### Materials

- **Type status:** Other material. **Occurrence:** recordedBy: ROPOS.COM; individualCount: 1; lifeStage: Adult; behavior: attached to hard substrates; occurrenceStatus: present; preparations: Imaged only; associatedMedia: R1912_01544.jpg; **Taxon:** taxonConceptID: Actiniaria fam. indet. (DZMB_2021_0017); kingdom: Animalia; phylum: Cnidaria; class: Anthozoa; order: Actiniaria; family: -; genus: -; taxonRank: Order; scientificNameAuthorship: Hertwig, 1882; **Location:** waterBody: Indian Ocean; stateProvince: Central Indian Ridge; locality: EGS; verbatimLocality: Cluster 4; maximumDepthInMeters: 3220; locationRemarks: RV Pelagia Cruise INDEX2015 Leg 2; geodeticDatum: WGS84; coordinateUncertaintyInMeters: 33; **Identification:** identifiedBy: Tina Molodtsova; identificationRemarks: Identified only from imagery; identificationQualifier: fam. indet.; **Event:** eventDate: 2015-12-05; eventTime: 10:48:55 am; year: 2015; fieldNumber: INDEX2015-56ROV; fieldNotes: 1.8°C; **Record Level:** language: en; institutionCode: DZMB; datasetName: INDEX; basisOfRecord: Human Observation

##### Notes

Fig. 70

#### Actiniaria fam. indet. (DZMB_2021_0018)

8C96E2B2-7999-5EC3-B348-6B6E3C516D63

##### Materials

- **Type status:** Other material. **Occurrence:** recordedBy: BGR; individualCount: 1; lifeStage: Adult; behavior: attached to hard substrates; occurrenceStatus: present; preparations: Imaged only; associatedMedia: IMG_4539.jpg; **Taxon:** taxonConceptID: Actiniaria fam. indet. (DZMB_2021_0018); kingdom: Animalia; phylum: Cnidaria; class: Anthozoa; order: Actiniaria; family: -; genus: -; taxonRank: Order; scientificNameAuthorship: Hertwig, 1882; **Location:** waterBody: Indian Ocean; stateProvince: South East Indian Ridge; locality: SEIR; verbatimLocality: Cluster 11; maximumDepthInMeters: 2899; locationRemarks: FS Sonne Cruise INDEX2017 Leg 1; decimalLatitude: -27.2565; decimalLongitude: 72.7243; geodeticDatum: WGS84; coordinateUncertaintyInMeters: 29; **Identification:** identifiedBy: Tina Molodtsova; identificationRemarks: Identified only from imagery; identificationQualifier: fam. indet.; **Event:** eventDate: 2017-09-24; eventTime: 3:26:59 pm; year: 2017; fieldNumber: INDEX2017-83STR; fieldNotes: 1.8°C, 34.7 ppt; **Record Level:** language: en; institutionCode: DZMB; datasetName: INDEX; basisOfRecord: Human Observation

##### Notes

Fig. 71

#### Actiniaria fam. indet. (DZMB_2021_0019)

67F28D27-40C9-5832-8747-281C6B474807

##### Materials

- **Type status:** Other material. **Occurrence:** recordedBy: ROPOS.COM; individualCount: 1; lifeStage: Adult; behavior: attached to hard substrates; occurrenceStatus: present; preparations: Imaged only; associatedMedia: R1909_00494-4.jpg; **Taxon:** taxonConceptID: Actiniaria fam. indet. (DZMB_2021_0019); kingdom: Animalia; phylum: Cnidaria; class: Anthozoa; order: Actiniaria; family: -; genus: -; taxonRank: Order; scientificNameAuthorship: Hertwig, 1882; **Location:** waterBody: Indian Ocean; stateProvince: Central Indian Ridge; locality: Vent site 1; verbatimLocality: Cluster 4; maximumDepthInMeters: 3048; locationRemarks: RV Pelagia Cruise INDEX2015 Leg 2; geodeticDatum: WGS84; coordinateUncertaintyInMeters: 30; **Identification:** identifiedBy: Tina Molodtsova; identificationRemarks: Identified only from imagery; identificationQualifier: fam. indet.; **Event:** eventDate: 2015-12-02; eventTime: 7:31:44 am; year: 2015; fieldNumber: INDEX2015-49ROV; fieldNotes: 1.8°C, 34.7 ppt; **Record Level:** language: en; institutionCode: DZMB; datasetName: INDEX; basisOfRecord: Human Observation

##### Notes

Fig. 72

#### Actiniaria fam. indet. (DZMB_2021_0020)

6377E73F-C156-59B0-8450-65AD21221500

##### Materials

- **Type status:** Other material. **Occurrence:** recordedBy: ROPOS.COM; individualCount: 1; lifeStage: Adult; behavior: attached to hard substrates; occurrenceStatus: present; preparations: Imaged only; associatedMedia: R1908_00374.jpg; **Taxon:** taxonConceptID: Actiniaria fam. indet. (DZMB_2021_0020); kingdom: Animalia; phylum: Cnidaria; class: Anthozoa; order: Actiniaria; family: -; genus: -; taxonRank: Order; scientificNameAuthorship: Hertwig, 1882; **Location:** waterBody: Indian Ocean; stateProvince: Central Indian Ridge; locality: Vent site 1; verbatimLocality: Cluster 4; maximumDepthInMeters: 3065; locationRemarks: RV Pelagia Cruise INDEX2015 Leg 2; geodeticDatum: WGS84; coordinateUncertaintyInMeters: 30; **Identification:** identifiedBy: Tina Molodtsova; identificationRemarks: Identified only from imagery; identificationQualifier: fam. indet.; **Event:** eventDate: 2015-12-01; eventTime: 6:22:02 am; year: 2015; fieldNumber: INDEX2015-47ROV; fieldNotes: 1.8°C, 34.7 ppt; **Record Level:** language: en; institutionCode: DZMB; datasetName: INDEX; basisOfRecord: Human Observation

##### Notes

Fig. 73

#### Actiniaria fam. indet. (DZMB_2021_0021)

7F362880-70E3-5476-A87A-F55214A1D38D

##### Materials

- **Type status:** Other material. **Occurrence:** recordedBy: BGR; individualCount: 1; lifeStage: Adult; behavior: attached to basalt; occurrenceStatus: present; preparations: Imaged only; associatedMedia: 38MFT Fotos 2013-9.jpg; **Taxon:** taxonConceptID: Actiniaria fam. indet. (DZMB_2021_0021); kingdom: Animalia; phylum: Cnidaria; class: Anthozoa; order: Actiniaria; family: -; genus: -; taxonRank: Order; scientificNameAuthorship: Hertwig, 1882; **Location:** waterBody: Indian Ocean; stateProvince: Central Indian Ridge; locality: Edmond; verbatimLocality: Cluster 4; maximumDepthInMeters: 3301; locationRemarks: FS Sonne Cruise INDEX2013 Leg 2; decimalLatitude: -23.8763; decimalLongitude: 69.5966; geodeticDatum: WGS84; coordinateUncertaintyInMeters: 33; **Identification:** identifiedBy: Tina Molodtsova; identificationRemarks: Identified only from imagery; identificationQualifier: fam. indet.; **Event:** eventDate: 2013-12-09; eventTime: 12:20:26 am; year: 2013; fieldNumber: INDEX2013-38MFT; fieldNotes: 1.8°C, 34.7 ppt; **Record Level:** language: en; institutionCode: DZMB; datasetName: INDEX; basisOfRecord: Human Observation

##### Notes

Fig. 74

#### Actiniaria fam. indet. (DZMB_2021_0022)

7526CDC8-F19E-541B-A769-E917A9970926

##### Materials

- **Type status:** Other material. **Occurrence:** recordedBy: BGR; individualCount: 1; lifeStage: Adult; behavior: on basalt/ sediment; occurrenceStatus: present; preparations: Imaged only; associatedMedia: IMG_3346.jpg; **Taxon:** taxonConceptID: Actiniaria fam. indet. (DZMB_2021_0022); kingdom: Animalia; phylum: Cnidaria; class: Anthozoa; order: Actiniaria; family: -; genus: -; taxonRank: Order; scientificNameAuthorship: Hertwig, 1882; **Location:** waterBody: Indian Ocean; stateProvince: South East Indian Ridge; locality: SEIR; verbatimLocality: Cluster 11; maximumDepthInMeters: 2859; locationRemarks: FS Sonne Cruise INDEX2017 Leg 1; decimalLatitude: -27.2462; decimalLongitude: 72.7151; geodeticDatum: WGS84; coordinateUncertaintyInMeters: 29; **Identification:** identifiedBy: Tina Molodtsova; identificationRemarks: Identified only from imagery; identificationQualifier: fam. indet.; **Event:** eventDate: 2017-09-24; eventTime: 1:39:04 pm; year: 2017; fieldNumber: INDEX2017-83STR; fieldNotes: 1.8°C, 34.7 ppt; **Record Level:** language: en; institutionCode: DZMB; datasetName: INDEX; basisOfRecord: Human Observation

##### Notes

Fig. 75

#### Actiniaria fam. indet. (DZMB_2021_0023)

08C95676-BB4E-57EF-9E28-C8924DBE5B83

##### Materials

- **Type status:** Other material. **Occurrence:** recordedBy: BGR; individualCount: 1; lifeStage: Adult; behavior: on basalt/ sediment; occurrenceStatus: present; preparations: Imaged only; associatedMedia: 38MFT Fotos 2013-31.jpg; **Taxon:** taxonConceptID: Actiniaria fam. indet. (DZMB_2021_0023); kingdom: Animalia; phylum: Cnidaria; class: Anthozoa; order: Actiniaria; family: -; genus: -; taxonRank: Order; scientificNameAuthorship: Hertwig, 1882; **Location:** waterBody: Indian Ocean; stateProvince: Central Indian Ridge; locality: Edmond; verbatimLocality: Cluster 4; maximumDepthInMeters: 3311; locationRemarks: FS Sonne Cruise INDEX2013 Leg 2; decimalLatitude: -23.8764; decimalLongitude: 69.5969; geodeticDatum: WGS84; coordinateUncertaintyInMeters: 33; **Identification:** identifiedBy: Tina Molodtsova; identificationRemarks: Identified only from imagery; identificationQualifier: fam. indet.; **Event:** eventDate: 2013-12-09; eventTime: 12:27:34 am; year: 2013; fieldNumber: INDEX2013-38MFT; fieldNotes: 1.8°C, 34.7 ppt; **Record Level:** language: en; institutionCode: DZMB; datasetName: INDEX; basisOfRecord: Human Observation

##### Notes

Fig. 76

#### Actiniaria fam. indet. (DZMB_2021_0024)

B0E059D9-601C-58DA-8C49-6326977CD43B

##### Materials

- **Type status:** Other material. **Occurrence:** recordedBy: BGR; individualCount: 2; lifeStage: Adult; behavior: attached to basalt; occurrenceStatus: present; preparations: Imaged only; associatedMedia: 38MFT Fotos 2013-471-3.jpg; **Taxon:** taxonConceptID: Actiniaria fam. indet. (DZMB_2021_0024); kingdom: Animalia; phylum: Cnidaria; class: Anthozoa; order: Actiniaria; family: -; genus: -; taxonRank: Order; scientificNameAuthorship: Hertwig, 1882; **Location:** waterBody: Indian Ocean; stateProvince: Central Indian Ridge; locality: Edmond; verbatimLocality: Cluster 4; maximumDepthInMeters: 3327; locationRemarks: FS Sonne Cruise INDEX2013 Leg 2; decimalLatitude: -23.8792; decimalLongitude: 69.5965; geodeticDatum: WGS84; coordinateUncertaintyInMeters: 33; **Identification:** identifiedBy: Tina Molodtsova; identificationRemarks: Identified only from imagery; identificationQualifier: fam. indet.; **Event:** eventDate: 2013-12-09; eventTime: 2:57:30 am; year: 2013; fieldNumber: INDEX2013-38MFT; fieldNotes: 1.8°C, 34.7 ppt; **Record Level:** language: en; institutionCode: DZMB; datasetName: INDEX; basisOfRecord: Human Observation

##### Notes

Fig. 77

#### Actiniaria fam. indet. (DZMB_2021_0025)

963B6E6E-5224-5A60-97F0-9114B66D937C

##### Materials

- **Type status:** Other material. **Occurrence:** recordedBy: ROPOS.COM; individualCount: 1; lifeStage: Adult; behavior: attached to sulphides/ basalt; occurrenceStatus: present; preparations: Imaged only; associatedMedia: R1906_00149-2.jpg; **Taxon:** taxonConceptID: Actiniaria fam. indet. (DZMB_2021_0025); kingdom: Animalia; phylum: Cnidaria; class: Anthozoa; order: Actiniaria; family: -; genus: -; taxonRank: Order; scientificNameAuthorship: Hertwig, 1882; **Location:** waterBody: Indian Ocean; stateProvince: Central Indian Ridge; locality: Vent site 1; verbatimLocality: Cluster 4; maximumDepthInMeters: 3024; locationRemarks: RV Pelagia Cruise INDEX2015 Leg 2; geodeticDatum: WGS84; coordinateUncertaintyInMeters: 30; **Identification:** identifiedBy: Tina Molodtsova; identificationRemarks: Identified only from imagery; identificationQualifier: fam. indet.; **Event:** eventDate: 2015-11-29; eventTime: 9:13:39 am; year: 2015; fieldNumber: INDEX2015-43ROV; fieldNotes: 1.8°C, 34.7 ppt; **Record Level:** language: en; institutionCode: DZMB; datasetName: INDEX; basisOfRecord: Human Observation

##### Notes

Fig. 78

#### Actinoscyphiidae

Stephenson, 1920

5EE32694-B71F-5E3B-A783-DDCCD6B79D3C

#### Actinoscyphiidae gen. indet. (DZMB_2021_0026)

11B0464A-E811-5F88-BE3E-01535E558FB7

##### Materials

- **Type status:** Other material. **Occurrence:** recordedBy: ROPOS.COM; individualCount: 1; lifeStage: Adult; behavior: attached to hard substrates; occurrenceStatus: present; preparations: Imaged only; associatedMedia: R1906_00140-3.jpg; **Taxon:** taxonConceptID: Actinoscyphiidae gen. indet. (DZMB_2021_0026); kingdom: Animalia; phylum: Cnidaria; class: Anthozoa; order: Actiniaria; family: Actinoscyphiidae; genus: -; taxonRank: Family; scientificNameAuthorship: Stephenson, 1920; **Location:** waterBody: Indian Ocean; stateProvince: Central Indian Ridge; locality: Vent site 1; verbatimLocality: Cluster 4; maximumDepthInMeters: 3035; locationRemarks: RV Pelagia Cruise INDEX2015 Leg 2; geodeticDatum: WGS84; coordinateUncertaintyInMeters: 30; **Identification:** identifiedBy: Tina Molodtsova; identificationRemarks: Identified only from imagery; identificationQualifier: gen. indet.; **Event:** eventDate: 2015-11-29; eventTime: 8:51:41 am; year: 2015; fieldNumber: INDEX2015-43ROV; fieldNotes: 1.8°C, 34.7 ppt; **Record Level:** language: en; institutionCode: DZMB; datasetName: INDEX; basisOfRecord: Human Observation

##### Notes

Fig. 79

#### Actinoscyphiidae gen. indet. (DZMB_2021_0027)

5DF4CB62-384E-5366-B9CD-20CC519526E3

##### Materials

- **Type status:** Other material. **Occurrence:** recordedBy: ROPOS.COM; individualCount: 1; lifeStage: Adult; behavior: Attached to coral stalk; occurrenceStatus: present; preparations: Imaged only; associatedMedia: R2095_00100-2.jpg; associatedOccurrences: Isididae Acanella gen. inc.; **Taxon:** taxonConceptID: Actinoscyphiidae gen. indet. (DZMB_2021_0027); kingdom: Animalia; phylum: Cnidaria; class: Anthozoa; order: Actiniaria; family: Actinoscyphiidae; genus: -; taxonRank: Family; scientificNameAuthorship: Stephenson, 1920; **Location:** waterBody: Indian Ocean; stateProvince: South East Indian Ridge; locality: Vent site 6; verbatimLocality: Cluster 12; maximumDepthInMeters: 2386; locationRemarks: RV Pelagia Cruise INDEX2018 Leg 2; geodeticDatum: WGS84; coordinateUncertaintyInMeters: 24; **Identification:** identifiedBy: Tina Molodtsova; identificationRemarks: Identified only from imagery; identificationQualifier: gen. indet.; **Event:** eventDate: 2018-11-23; eventTime: 7:10:30 am; year: 2018; fieldNumber: INDEX2018-63ROPOS; fieldNotes: 1.8°C; **Record Level:** language: en; institutionCode: DZMB; datasetName: INDEX; basisOfRecord: Human Observation

##### Notes

Fig. 80

#### Actinoscyphia

Stephenson, 1920

56984E2D-A749-540A-B1DA-A02380B14D6F

#### Actinoscyphia sp. indet.

B275B717-9455-562D-B094-7B87979DF264

##### Materials

- **Type status:** Other material. **Occurrence:** recordedBy: ROPOS.COM; individualCount: 1; lifeStage: Adult; behavior: Attached to coral stalk; occurrenceStatus: present; preparations: Imaged only; associatedMedia: R2105_00314-2.jpg; associatedOccurrences: Isididae Keratoisis gen. inc.; **Taxon:** taxonConceptID: Actinoscyphia sp. indet.; kingdom: Animalia; phylum: Cnidaria; class: Anthozoa; order: Actiniaria; family: Actinoscyphiidae; genus: Actinoscyphia; taxonRank: Genus; scientificNameAuthorship: Stephenson, 1920; **Location:** waterBody: Indian Ocean; stateProvince: Rodriguez Triple Junction; locality: Vent site 4; verbatimLocality: Cluster 5; maximumDepthInMeters: 2661; locationRemarks: RV Pelagia Cruise INDEX2018 Leg 2; geodeticDatum: WGS84; coordinateUncertaintyInMeters: 26; **Identification:** identifiedBy: Tina Molodtsova; identificationRemarks: Identified only from imagery; identificationQualifier: sp. indet.; **Event:** eventDate: 2018-12-09; eventTime: 9:21:20 am; year: 2018; fieldNumber: INDEX2018-95ROPOS; fieldNotes: 1.8°C, 34.7 ppt; **Record Level:** language: en; institutionCode: DZMB; datasetName: INDEX; basisOfRecord: Human Observation

##### Notes

Fig. 81

#### Actinostolidae

Carlgren, 1932

ABAC9CD2-7A51-5FB8-8AEF-D46C3CE0270D

#### Actinostolidae gen. indet.

8989B3A5-9BE1-5B42-95F8-E67A4D03D336

##### Materials

- **Type status:** Other material. **Occurrence:** recordedBy: ROPOS.COM; individualCount: 1; lifeStage: Adult; behavior: attached to hard substrates; occurrenceStatus: present; preparations: Imaged only; associatedMedia: R2097_00308.jpg; associatedOccurrences: Polynoidae gen. indet. (commensal); **Taxon:** taxonConceptID: Actinostolidae gen. indet.; kingdom: Animalia; phylum: Cnidaria; class: Anthozoa; order: Actiniaria; family: Actinostolidae; genus: -; taxonRank: Family; scientificNameAuthorship: Carlgren, 1932; **Location:** waterBody: Indian Ocean; stateProvince: South East Indian Ridge; locality: Vent site 6; verbatimLocality: Cluster 12; maximumDepthInMeters: 2485; locationRemarks: RV Pelagia Cruise INDEX2018 Leg 2; geodeticDatum: WGS84; coordinateUncertaintyInMeters: 24; **Identification:** identifiedBy: Tina Molodtsova; identificationRemarks: Identified only from imagery; identificationQualifier: gen. indet.; **Event:** eventDate: 2018-11-25; eventTime: 11:37:11 am; year: 2018; fieldNumber: INDEX2018-67ROPOS; fieldNotes: 1.8°C, 34.7 ppt; **Record Level:** language: en; institutionCode: DZMB; datasetName: INDEX; basisOfRecord: Human Observation

##### Notes

Fig. 82

#### Actinostola

Verrill, 1883

DD3A4DB2-5C14-5E53-B917-0DEC00229839

#### Actinostola sp. indet. (DZMB_2021_0028)

0E2D8408-0FB0-5492-9C2F-D1CDDC12053E

##### Materials

- **Type status:** Other material. **Occurrence:** recordedBy: ROPOS.COM; individualCount: 1; lifeStage: Adult; behavior: attached to hard substrates; occurrenceStatus: present; preparations: Imaged only; associatedMedia: R2102_00166.jpg; **Taxon:** taxonConceptID: Actinostola sp. indet. (DZMB_2021_0028); kingdom: Animalia; phylum: Cnidaria; class: Anthozoa; order: Actiniaria; family: Actinostolidae; genus: Actinostola; taxonRank: Genus; scientificNameAuthorship: Verrill, 1883; **Location:** waterBody: Indian Ocean; stateProvince: South East Indian Ridge; locality: Vent site 5; verbatimLocality: Cluster 11; maximumDepthInMeters: 2991; locationRemarks: RV Pelagia Cruise INDEX2018 Leg 2; geodeticDatum: WGS84; coordinateUncertaintyInMeters: 30; **Identification:** identifiedBy: Tina Molodtsova; identificationRemarks: Identified only from imagery; identificationQualifier: sp. indet.; **Event:** eventDate: 2018-12-01; eventTime: 8:42:41 am; year: 2018; fieldNumber: INDEX2018-80ROPOS; fieldNotes: 1.8°C, 34.7 ppt; **Record Level:** language: en; institutionCode: DZMB; datasetName: INDEX; basisOfRecord: Human Observation

##### Notes

Fig. 83

#### Actinostola sp. indet. (DZMB_2021_0029)

DB866757-3376-5370-A60B-FDB6626FC73F

##### Materials

- **Type status:** Other material. **Occurrence:** recordedBy: ROPOS.COM; individualCount: 1; lifeStage: Adult; behavior: attached to hard substrates; occurrenceStatus: present; preparations: Imaged only; associatedMedia: R2100_00020.jpg; **Taxon:** taxonConceptID: Actinostola sp. indet. (DZMB_2021_0029); kingdom: Animalia; phylum: Cnidaria; class: Anthozoa; order: Actiniaria; family: Actinostolidae; genus: Actinostola; taxonRank: Genus; scientificNameAuthorship: Verrill, 1883; **Location:** waterBody: Indian Ocean; stateProvince: South East Indian Ridge; locality: Vent site 5; verbatimLocality: Cluster 11; maximumDepthInMeters: 2910; locationRemarks: RV Pelagia Cruise INDEX2018 Leg 2; geodeticDatum: WGS84; coordinateUncertaintyInMeters: 29; **Identification:** identifiedBy: Tina Molodtsova; identificationRemarks: Identified only from imagery - burrowing morphotype; identificationQualifier: sp. indet.; **Event:** eventDate: 2018-11-28; eventTime: 6:11:14 am; year: 2018; fieldNumber: INDEX2018-73ROPOS; fieldNotes: 1.8°C, 34.7 ppt; **Record Level:** language: en; institutionCode: DZMB; datasetName: INDEX; basisOfRecord: Human Observation

##### Notes

Fig. 84

#### Actinostola sp. indet. (DZMB_2021_0030)

347E5C00-9BF8-544C-8056-FF99217ADA61

##### Materials

- **Type status:** Other material. **Occurrence:** recordedBy: ROPOS.COM; individualCount: 1; lifeStage: Adult; behavior: attached to hard substrates; occurrenceStatus: present; preparations: Imaged only; associatedMedia: R1906_00139.jpg; **Taxon:** taxonConceptID: Actinostola sp. indet. (DZMB_2021_0030); kingdom: Animalia; phylum: Cnidaria; class: Anthozoa; order: Actiniaria; family: Actinostolidae; genus: Actinostola; taxonRank: Genus; scientificNameAuthorship: Verrill, 1883; **Location:** waterBody: Indian Ocean; stateProvince: Central Indian Ridge; locality: Vent site 1; verbatimLocality: Cluster 4; maximumDepthInMeters: 3023; locationRemarks: RV Pelagia Cruise INDEX2015 Leg 2; geodeticDatum: WGS84; coordinateUncertaintyInMeters: 30; **Identification:** identifiedBy: Tina Molodtsova; identificationRemarks: Identified only from imagery; identificationQualifier: sp. indet.; **Event:** eventDate: 2015-11-29; eventTime: 8:40:12 am; year: 2015; fieldNumber: INDEX2015-43ROV; fieldNotes: 1.8°C, 34.7 ppt; **Record Level:** language: en; institutionCode: DZMB; datasetName: INDEX; basisOfRecord: Human Observation

##### Notes

Fig. 85

#### Actinostola sp. indet. (DZMB_2021_0031)

C953E85A-DA7B-5C73-904B-F62F38B40197

##### Materials

- **Type status:** Other material. **Occurrence:** recordedBy: BGR; individualCount: 1; lifeStage: Adult; behavior: on basalt/ sediment; occurrenceStatus: present; preparations: Imaged only; associatedMedia: IMG_5410.jpg; **Taxon:** taxonConceptID: Actinostola sp. indet. (DZMB_2021_0031); kingdom: Animalia; phylum: Cnidaria; class: Anthozoa; order: Actiniaria; family: Actinostolidae; genus: Actinostola; taxonRank: Genus; scientificNameAuthorship: Verrill, 1883; **Location:** waterBody: Indian Ocean; stateProvince: South East Indian Ridge; locality: Vent site 5; verbatimLocality: Cluster 11; maximumDepthInMeters: 2408; locationRemarks: FS Sonne Cruise INDEX2017 Leg 1; geodeticDatum: WGS84; coordinateUncertaintyInMeters: 29; **Identification:** identifiedBy: Tina Molodtsova; identificationRemarks: Identified only from imagery; identificationQualifier: sp. indet.; **Event:** eventDate: 2017-09-27; eventTime: 9:16:09 am; year: 2017; fieldNumber: INDEX2017-94STR; fieldNotes: 1.8°C, 34.7 ppt; **Record Level:** language: en; institutionCode: DZMB; datasetName: INDEX; basisOfRecord: Human Observation

##### Notes

Fig. 86

#### Bathyphellidae

Carlgren, 1932

4AF72BD3-C6FF-587B-A189-53C4F03ED417

#### Bathyphellia

Carlgren, 1932

A2BB9C99-BD6D-5521-8F75-9894F9176838

#### Bathyphellia sp. indet. (DZMB_2021_0032)

91C24BED-A0AA-5436-A16E-74AF5F35C19D

##### Materials

- **Type status:** Other material. **Occurrence:** recordedBy: BGR; individualCount: 2; lifeStage: Adult; behavior: attached to basalt; occurrenceStatus: present; preparations: Imaged only; associatedMedia: IMG_5093.jpg; **Taxon:** taxonConceptID: Bathyphellia sp. indet. (DZMB_2021_0032); kingdom: Animalia; phylum: Cnidaria; class: Anthozoa; order: Actiniaria; family: Bathyphelliidae; genus: Bathyphellia; taxonRank: Genus; scientificNameAuthorship: Carlgren, 1932; **Location:** waterBody: Indian Ocean; stateProvince: South East Indian Ridge; locality: SEIR; verbatimLocality: Cluster 11; maximumDepthInMeters: 2784; locationRemarks: FS Sonne Cruise INDEX2017 Leg 1; decimalLatitude: -27.2562; decimalLongitude: 72.7216; geodeticDatum: WGS84; coordinateUncertaintyInMeters: 29; **Identification:** identifiedBy: Tina Molodtsova; identificationRemarks: Identified only from imagery; identificationQualifier: sp. indet.; **Event:** eventDate: 2017-09-25; eventTime: 3:51:39 am; year: 2017; fieldNumber: INDEX2017-86STR; fieldNotes: 1.8°C, 34.7 ppt; **Record Level:** language: en; institutionCode: DZMB; datasetName: INDEX; basisOfRecord: Human Observation

##### Notes

Fig. 87

#### Bathyphellia sp. indet. (DZMB_2021_0033)

B0B1A996-FDF3-58BA-824C-92E1212844EA

##### Materials

- **Type status:** Other material. **Occurrence:** recordedBy: BGR; individualCount: 3; lifeStage: Adult; behavior: attached to basalt; occurrenceStatus: present; preparations: Imaged only; associatedMedia: IMG_5423.jpg; **Taxon:** taxonConceptID: Bathyphellia sp. indet. (DZMB_2021_0033); kingdom: Animalia; phylum: Cnidaria; class: Anthozoa; order: Actiniaria; family: Bathyphelliidae; genus: Bathyphellia; taxonRank: Genus; scientificNameAuthorship: Carlgren, 1932; **Location:** waterBody: Indian Ocean; stateProvince: South East Indian Ridge; locality: Vent site 5; verbatimLocality: Cluster 11; maximumDepthInMeters: 2920; locationRemarks: FS Sonne Cruise INDEX2017 Leg 1; geodeticDatum: WGS84; coordinateUncertaintyInMeters: 29; **Identification:** identifiedBy: Tina Molodtsova; identificationRemarks: Identified only from imagery; identificationQualifier: sp. indet.; **Event:** eventDate: 2017-09-27; eventTime: 9:16:42 am; year: 2017; fieldNumber: INDEX2017-94STR; fieldNotes: 1.8°C, 34.7 ppt; **Record Level:** language: en; institutionCode: DZMB; datasetName: INDEX; basisOfRecord: Human Observation

##### Notes

Fig. 88

#### Hormathiidae

Carlgren, 1932

AAE4A30E-D116-5EEE-9F28-3401A000E4B3

#### Chondrophellia

Carlgren, 1925

7F6FF114-82C1-5105-8EBC-84392037EB68

#### Chondrophellia sp. indet.

F5174E53-3ACD-5558-8CEA-AAA2578A1BB5

##### Materials

- **Type status:** Other material. **Occurrence:** recordedBy: ROPOS.COM; individualCount: 1; lifeStage: Adult; behavior: attached to sulphides; occurrenceStatus: present; preparations: Imaged only; associatedMedia: R1909_00519.jpg; **Taxon:** taxonConceptID: Chondrophellia sp. indet.; kingdom: Animalia; phylum: Cnidaria; class: Anthozoa; order: Actiniaria; family: Hormathiidae; genus: Chondrophellia; taxonRank: Genus; scientificNameAuthorship: Carlgren, 1925; **Location:** waterBody: Indian Ocean; stateProvince: Central Indian Ridge; locality: Vent site 1; verbatimLocality: Cluster 4; maximumDepthInMeters: 3049; locationRemarks: RV Pelagia Cruise INDEX2015 Leg 2; geodeticDatum: WGS84; coordinateUncertaintyInMeters: 30; **Identification:** identifiedBy: Tina Molodtsova; identificationRemarks: Identified only from imagery; identificationQualifier: sp. indet.; **Event:** eventDate: 2015-12-02; eventTime: 7:40:38 am; year: 2015; fieldNumber: INDEX2015-49ROV; fieldNotes: 1.8°C, 34.7 ppt; **Record Level:** language: en; institutionCode: DZMB; datasetName: INDEX; basisOfRecord: Human Observation

##### Notes

Fig. 89

#### Kadosactinidae

Riemann-Zürneck, 1991

6B160587-56B2-566A-B861-75BFABFEDFD4

#### Maractis

Fautin & Barber, 1999

B864F41D-796E-51CC-A128-4EE9EC053116

#### Maractis sp. indet.

9010704A-B706-5D1D-9249-ECCE738D4505

##### Materials

- **Type status:** Other material. **Occurrence:** recordedBy: BGR/ GEOMAR; individualCount: 2; lifeStage: Adult; behavior: attached to sulphides/ basalt; occurrenceStatus: present; preparations: Imaged only; associatedMedia: 2013-12-06_08-13-55_Sonne_INDEX2013-2_031ROV02_Logo-2.jpg; **Taxon:** taxonConceptID: Maractis sp. indet.; kingdom: Animalia; phylum: Cnidaria; class: Anthozoa; order: Actiniaria; family: Kadosactinidae; genus: Maractis; taxonRank: Genus; scientificNameAuthorship: Fautin & Barber, 1999; **Location:** waterBody: Indian Ocean; stateProvince: Rodriguez Triple Junction; locality: Kairei; verbatimLocality: Cluster 5; maximumDepthInMeters: 2446; locationRemarks: FS Sonne Cruise INDEX2013 Leg 2; geodeticDatum: WGS84; coordinateUncertaintyInMeters: 24; **Identification:** identifiedBy: Tina Molodtsova; identificationRemarks: Identified only from imagery; identificationQualifier: sp. indet.; **Event:** eventDate: 2013-12-06; eventTime: 8:13:55 am; year: 2013; fieldNumber: INDEX2013-31ROV; fieldNotes: 1.8°C, 34.6 ppt; **Record Level:** language: en; institutionCode: DZMB; datasetName: INDEX; basisOfRecord: Preserved Specimen

##### Notes

Fig. 90

#### Relicanthidae

Rodríguez & Daly, 2014

A1D226BF-8A20-53D2-B4E1-698DE5C1D707

#### Relicanthus

Rodriguez & Daly, 2014

0DC3AF6A-4900-5BAB-8D99-5BC260528846

#### Relicanthus daphneae sp. inc.

(Daly, 2006)

67292DE1-1101-540F-AAEF-B6943DB7B9EC

##### Materials

- **Type status:** Other material. **Occurrence:** recordedBy: BGR; individualCount: 1; lifeStage: Adult; behavior: on basalt; occurrenceStatus: present; preparations: Imaged only; associatedMedia: IMG_3341.jpg; **Taxon:** taxonConceptID: Relicanthusdaphneae sp. inc.; scientificName: Relicanthusdaphneae; kingdom: Animalia; phylum: Cnidaria; class: Anthozoa; order: Actiniaria; family: Relicanthidae; genus: Relicanthus; taxonRank: Species; scientificNameAuthorship: (Daly, 2006); **Location:** waterBody: Indian Ocean; stateProvince: South East Indian Ridge; locality: Vent site 5; verbatimLocality: Cluster 11; maximumDepthInMeters: 3005; locationRemarks: FS Sonne Cruise INDEX2017 Leg 1; geodeticDatum: WGS84; coordinateUncertaintyInMeters: 29; **Identification:** identifiedBy: Tina Molodtsova; identificationRemarks: Identified only from imagery; identificationQualifier: sp. inc.; **Event:** eventDate: 2017-09-27; eventTime: 7:48:41 am; year: 2017; fieldNumber: INDEX2017-94STR; fieldNotes: 1.8°C, 34.7 ppt; **Record Level:** language: en; institutionCode: DZMB; datasetName: INDEX; basisOfRecord: Human Observation

##### Notes

Fig. 91

#### Alcyonacea

Lamouroux, 1812

E5DC53E2-6C21-5F3D-842F-9759CDB56D9D

#### Alcyonacea fam. indet.

A6F4D517-3FB1-5D53-8C3F-65319535D611

##### Materials

- **Type status:** Other material. **Occurrence:** recordedBy: BGR/ GEOMAR; individualCount: 2; lifeStage: Adult; behavior: attached to basalt; occurrenceStatus: present; preparations: Imaged only; associatedMedia: 2013-12-15_07-35-33_Sonne_INDEX2013-2_057ROV09_Logo-3.jpg; **Taxon:** taxonConceptID: Alcyonacea fam. indet.; kingdom: Animalia; phylum: Cnidaria; class: Anthozoa; order: Alcyonacea; family: -; genus: -; taxonRank: Order; scientificNameAuthorship: Lamouroux, 1812; **Location:** waterBody: Indian Ocean; stateProvince: Central Indian Ridge; locality: MESO; verbatimLocality: outside INDEX claim; maximumDepthInMeters: 2824; locationRemarks: FS Sonne Cruise INDEX2013 Leg 2; geodeticDatum: WGS84; coordinateUncertaintyInMeters: 30; **Identification:** identifiedBy: Tina Molodtsova; identificationRemarks: Identified only from imagery; identificationQualifier: fam. indet.; **Event:** eventDate: 2013-12-15; eventTime: 7:35:33 am; year: 2013; fieldNumber: INDEX2013-57ROV; fieldNotes: 1.8°C, 34.5 ppt; **Record Level:** language: en; institutionCode: DZMB; datasetName: INDEX; basisOfRecord: Human Observation

##### Notes

Fig. 92

#### Alcyoniidae

Lamouroux, 1812

99688577-EA9E-5ADD-AE07-4FFDDE736458

#### Anthomastus

Verrill, 1878

C94C170A-95E8-590B-BDFA-1223366F6C39

#### Anthomastus gen. inc.

6E5396FF-20E3-541A-B762-5CB6D0B2E4F0

##### Materials

- **Type status:** Other material. **Occurrence:** recordedBy: ROPOS.COM; individualCount: 1; lifeStage: Adult; behavior: on basalt/sediment; occurrenceStatus: present; preparations: Imaged only; associatedMedia: R2093_00822.jpg; **Taxon:** taxonConceptID: Alcyonacea Anthomastus gen. inc.; kingdom: Animalia; phylum: Cnidaria; class: Anthozoa; order: Alcyonacea; family: Alcyoniidae; genus: Anthomastus; taxonRank: Genus; scientificNameAuthorship: Verrill, 1878; **Location:** waterBody: Indian Ocean; stateProvince: South East Indian Ridge; locality: Vent site 6; verbatimLocality: Cluster 12; maximumDepthInMeters: 2361; locationRemarks: RV Pelagia Cruise INDEX2018 Leg 2; geodeticDatum: WGS84; coordinateUncertaintyInMeters: 23; **Identification:** identifiedBy: Tina Molodtsova; identificationRemarks: Identified only from imagery; identificationQualifier: gen. inc.; **Event:** eventDate: 2018-11-21; eventTime: 9:20:48 am; year: 2018; fieldNumber: INDEX2018-59ROPOS; fieldNotes: 1.8°C, 34.7 ppt; **Record Level:** language: en; institutionCode: DZMB; datasetName: INDEX; basisOfRecord: Human Observation

##### Notes

Fig. 93

#### Anthomastus sp. indet.

323567B4-C93B-57CD-915F-D511801F431F

##### Materials

- **Type status:** Other material. **Occurrence:** recordedBy: BGR/ GEOMAR; individualCount: 1; lifeStage: Adult; behavior: attached to basalt; occurrenceStatus: present; preparations: Imaged only; associatedMedia: 2013-12-15_08-18-40_Sonne_INDEX2013-2_057ROV09_Logo-2.jpg; **Taxon:** taxonConceptID: Anthomastus sp. indet.; kingdom: Animalia; phylum: Cnidaria; class: Anthozoa; order: Alcyonacea; family: Alcyoniidae; genus: Anthomastus; taxonRank: Genus; scientificNameAuthorship: Verrill, 1878; **Location:** waterBody: Indian Ocean; stateProvince: Central Indian Ridge; locality: MESO; verbatimLocality: outside INDEX claim; maximumDepthInMeters: 2826; locationRemarks: FS Sonne Cruise INDEX2013 Leg 2; geodeticDatum: WGS84; coordinateUncertaintyInMeters: 30; **Identification:** identifiedBy: Tina Molodtsova; identificationRemarks: Identified only from imagery; identificationQualifier: sp. indet.; **Event:** eventDate: 2013-12-15; eventTime: 8:18:40 am; year: 2013; fieldNumber: INDEX2013-57ROV; fieldNotes: 1.8°C, 34.5 ppt; **Record Level:** language: en; institutionCode: DZMB; datasetName: INDEX; basisOfRecord: Human Observation

##### Notes

Fig. 94

#### Chrysogorgiidae

Verrill, 1883

6A842795-1EA6-531D-BBE8-388FD8F3692D

#### Chrysogorgia

Duchassaing & Michelotti, 1864

E86EC535-A528-5B8D-B7D0-165C719C85B7

#### Chrysogorgia sp. indet. (DZMB_2021_0034)

C8CA963B-E0B6-5545-A37C-4A90B0C27A87

##### Materials

- **Type status:** Other material. **Occurrence:** recordedBy: BGR/ GEOMAR; individualCount: 1; lifeStage: Adult; behavior: attached to sulphides; occurrenceStatus: present; preparations: Imaged only; associatedMedia: 2013-12-16_06-53-32_Sonne_INDEX2013-2_059ROV10_Logo.jpg; **Taxon:** taxonConceptID: Chrysogorgia sp. indet. (DZMB_2021_0034); kingdom: Animalia; phylum: Cnidaria; class: Anthozoa; order: Alcyonacea; family: Chrysogorgiidae; genus: Chrysogorgia; taxonRank: Genus; scientificNameAuthorship: Duchassaing & Michelotti, 1864; **Location:** waterBody: Indian Ocean; stateProvince: Central Indian Ridge; locality: MESO; verbatimLocality: outside INDEX claim; maximumDepthInMeters: 2817; locationRemarks: FS Sonne Cruise INDEX2013 Leg 2; geodeticDatum: WGS84; coordinateUncertaintyInMeters: 28; **Identification:** identifiedBy: Tina Molodtsova; identificationRemarks: Identified only from imagery; identificationQualifier: sp. indet.; **Event:** eventDate: 2013-12-16; eventTime: 6:53:32 am; year: 2013; fieldNumber: INDEX2013-59ROV; fieldNotes: 1.8°C, 34.5 ppt; **Record Level:** language: en; institutionCode: DZMB; datasetName: INDEX; basisOfRecord: Human Observation

##### Notes

Fig. 95

#### Chrysogorgia sp. indet. (DZMB_2021_0035)

D5F0F442-2878-55FC-83AD-BFCBF1BF35D3

##### Materials

- **Type status:** Other material. **Occurrence:** recordedBy: BGR/ GEOMAR; individualCount: 1; lifeStage: Adult; behavior: attached to sulphides; occurrenceStatus: present; preparations: Imaged only; associatedMedia: 2013-12-17_12-37-23_Sonne_INDEX2013-2_062ROV11_Logo-2.jpg; **Taxon:** taxonConceptID: Chrysogorgia sp. indet. (DZMB_2021_0035); kingdom: Animalia; phylum: Cnidaria; class: Anthozoa; order: Alcyonacea; family: Chrysogorgiidae; genus: Chrysogorgia; taxonRank: Genus; scientificNameAuthorship: Duchassaing & Michelotti, 1864; **Location:** waterBody: Indian Ocean; stateProvince: Central Indian Ridge; locality: MESO; verbatimLocality: outside INDEX claim; maximumDepthInMeters: 2828; locationRemarks: FS Sonne Cruise INDEX2013 Leg 2; geodeticDatum: WGS84; coordinateUncertaintyInMeters: 28; **Identification:** identifiedBy: Tina Molodtsova; identificationRemarks: Identified only from imagery; identificationQualifier: sp. indet.; **Event:** eventDate: 2013-12-17; eventTime: 12:37:23 pm; year: 2013; fieldNumber: INDEX2013-62ROV; fieldNotes: 1.8°C, 34.4 ppt; **Record Level:** language: en; institutionCode: DZMB; datasetName: INDEX; basisOfRecord: Human Observation

##### Notes

Fig. 96

#### Iridogorgia

Verrill, 1883

DECCE411-9B89-5F0E-B485-6B375A203414

#### Iridogorgia magnispiralis sp. inc.

Watling, 2007

3D525916-678D-5783-A0B2-C3AE7EF2B89B

##### Materials

- **Type status:** Other material. **Occurrence:** recordedBy: ROPOS.COM; individualCount: 1; lifeStage: Adult; behavior: attached to basalt; occurrenceStatus: present; preparations: Imaged only; associatedMedia: R2092_00358.jpg; **Taxon:** taxonConceptID: Iridogorgiamagnispiralis sp. inc.; scientificName: Iridogorgiamagnispiralis; kingdom: Animalia; phylum: Cnidaria; class: Anthozoa; order: Alcyonacea; family: Chrysogorgiidae; genus: Iridogorgia; taxonRank: Species; scientificNameAuthorship: Watling, 2007; **Location:** waterBody: Indian Ocean; stateProvince: South East Indian Ridge; locality: Vent site 6; verbatimLocality: Cluster 12; maximumDepthInMeters: 2461; locationRemarks: RV Pelagia Cruise INDEX2018 Leg 2; geodeticDatum: WGS84; coordinateUncertaintyInMeters: 25; **Identification:** identifiedBy: Tina Molodtsova; identificationRemarks: Identified only from imagery; identificationQualifier: sp. inc.; **Event:** eventDate: 2018-11-20; eventTime: 6:33:26 am; year: 2018; fieldNumber: INDEX2018-57ROPOS; fieldNotes: 1.8°C, 34.7 ppt; **Record Level:** language: en; institutionCode: DZMB; datasetName: INDEX; basisOfRecord: Human Observation

##### Notes

Fig. 97

#### Clavulariidae

Hickson, 1894

95C2EF3F-AB0C-50B3-8C99-118544A38F45

#### Clavulariidae gen. indet. (DZMB_2021_0036)

236B1AD5-9F1E-5838-8376-48450D5E5466

##### Materials

- **Type status:** Other material. **Occurrence:** recordedBy: ROPOS.COM; individualCount: 100; lifeStage: Adult; behavior: attached to sulphides/ basalt; occurrenceStatus: present; preparations: Imaged only; associatedMedia: R1909_00483-2.jpg; **Taxon:** taxonConceptID: Clavulariidae gen. indet. (DZMB_2021_0036); kingdom: Animalia; phylum: Cnidaria; class: Anthozoa; order: Alcyonacea; family: Clavulariidae; genus: -; taxonRank: Family; scientificNameAuthorship: Hickson, 1894; **Location:** waterBody: Indian Ocean; stateProvince: Central Indian Ridge; locality: Vent site 1; verbatimLocality: Cluster 4; maximumDepthInMeters: 3048; locationRemarks: RV Pelagia Cruise INDEX2015 Leg 2; geodeticDatum: WGS84; coordinateUncertaintyInMeters: 30; **Identification:** identifiedBy: Tina Molodtsova; identificationRemarks: Identified only from imagery; identificationQualifier: gen. indet.; **Event:** eventDate: 2015-12-02; eventTime: 6:51:26 am; year: 2015; fieldNumber: INDEX2015-49ROV; fieldNotes: 1.8°C, 34.7 ppt; **Record Level:** language: en; institutionCode: DZMB; datasetName: INDEX; basisOfRecord: Human Observation

##### Notes

Fig. 98

#### Clavulariidae gen. indet. (DZMB_2021_0037)

7EFEB58B-7ADC-51E4-A18F-0A96D51397C8

##### Materials

- **Type status:** Other material. **Occurrence:** recordedBy: BGR; individualCount: 1; lifeStage: Adult; behavior: attached to sulphides/ basalt; occurrenceStatus: present; preparations: Imaged only; associatedMedia: 17MFT Fotos 2013-365.jpg; **Taxon:** taxonConceptID: Clavulariidae gen. indet. (DZMB_2021_0037); kingdom: Animalia; phylum: Cnidaria; class: Anthozoa; order: Alcyonacea; family: Clavulariidae; genus: -; taxonRank: Family; scientificNameAuthorship: Hickson, 1894; **Location:** waterBody: Indian Ocean; stateProvince: Central Indian Ridge; locality: MESO; verbatimLocality: outside INDEX claim; maximumDepthInMeters: 2830; locationRemarks: FS Sonne Cruise INDEX2013 Leg 1; decimalLatitude: -23.3799; decimalLongitude: 69.2352; geodeticDatum: WGS84; coordinateUncertaintyInMeters: 28; **Identification:** identifiedBy: Tina Molodtsova; identificationRemarks: Identified only from imagery; identificationQualifier: gen. indet.; **Event:** eventDate: 2013-11-25; eventTime: 6:34:34 am; year: 2013; fieldNumber: INDEX2013-17MFT; fieldNotes: 1.8°C, 34.7 ppt; **Record Level:** language: en; institutionCode: DZMB; datasetName: INDEX; basisOfRecord: Human Observation

##### Notes

Fig. 99

#### Clavulariidae fam. inc. (DZMB_2021_0038)

9E7A3DBE-BEE0-5163-A13A-59C413983DBC

##### Materials

- **Type status:** Other material. **Occurrence:** recordedBy: BGR; individualCount: 1; lifeStage: Adult; behavior: on sediment; occurrenceStatus: present; preparations: Imaged only; associatedMedia: 17MFT Fotos 2013-200.jpg; **Taxon:** taxonConceptID: Clavulariidae fam. inc. (DZMB_2021_0038); kingdom: Animalia; phylum: Cnidaria; class: Anthozoa; order: Alcyonacea; family: Clavulariidae; genus: -; taxonRank: Family; scientificNameAuthorship: Hickson, 1894; **Location:** waterBody: Indian Ocean; stateProvince: Central Indian Ridge; locality: MESO; verbatimLocality: outside INDEX claim; maximumDepthInMeters: 2799; locationRemarks: FS Sonne Cruise INDEX2013 Leg 1; decimalLatitude: -23.3878; decimalLongitude: 69.2403; geodeticDatum: WGS84; coordinateUncertaintyInMeters: 28; **Identification:** identifiedBy: Tina Molodtsova; identificationRemarks: Identified only from imagery; identificationQualifier: fam. inc.; **Event:** eventDate: 2013-11-25; eventTime: 4:30:29 am; year: 2013; fieldNumber: INDEX2013-17MFT; fieldNotes: 1.8°C, 34.7 ppt; **Record Level:** language: en; institutionCode: DZMB; datasetName: INDEX; basisOfRecord: Human Observation

##### Notes

Fig. 100

#### Clavulariidae fam. inc. (DZMB_2021_0039)

3D9C7F44-F53D-51C5-AABE-470D13D251BD

##### Materials

- **Type status:** Other material. **Occurrence:** recordedBy: ROPOS.COM; individualCount: 1; lifeStage: Adult; behavior: attached to sulphides; occurrenceStatus: present; preparations: Imaged only; associatedMedia: R1909_00497.jpg; **Taxon:** taxonConceptID: Clavulariidae fam. inc. (DZMB_2021_0039); kingdom: Animalia; phylum: Cnidaria; class: Anthozoa; order: Alcyonacea; family: Clavulariidae; genus: -; taxonRank: Family; scientificNameAuthorship: Hickson, 1894; **Location:** waterBody: Indian Ocean; stateProvince: Central Indian Ridge; locality: Vent site 1; verbatimLocality: Cluster 4; maximumDepthInMeters: 3046; locationRemarks: RV Pelagia Cruise INDEX2015 Leg 2; geodeticDatum: WGS84; coordinateUncertaintyInMeters: 30; **Identification:** identifiedBy: Tina Molodtsova; identificationRemarks: Identified only from imagery; identificationQualifier: fam. inc.; **Event:** eventDate: 2015-12-02; eventTime: 7:32:34 am; year: 2015; fieldNumber: INDEX2015-49ROV; fieldNotes: 1.8°C, 34.7 ppt; **Record Level:** language: en; institutionCode: DZMB; datasetName: INDEX; basisOfRecord: Human Observation

##### Notes

Fig. 101

#### Isididae

Lamouroux, 1812

E1E731E9-6995-5B4F-8EA2-4B6916AFCDCC

#### Isididae gen. indet. (DZMB_2021_0040)

00BC8F04-13F9-5A4B-BDCB-5ABF29B5E09B

##### Materials

- **Type status:** Other material. **Occurrence:** recordedBy: ROPOS.COM; individualCount: 1; lifeStage: Adult; behavior: attached to basalt; occurrenceStatus: present; preparations: Imaged only; associatedMedia: R2096_00254.jpg; **Taxon:** taxonConceptID: Isididae gen. indet. (DZMB_2021_0040); kingdom: Animalia; phylum: Cnidaria; class: Anthozoa; order: Alcyonacea; family: Isididae; genus: -; taxonRank: Family; scientificNameAuthorship: Lamouroux, 1812; **Location:** waterBody: Indian Ocean; stateProvince: South East Indian Ridge; locality: Vent site 6; verbatimLocality: Cluster 12; maximumDepthInMeters: 2481; locationRemarks: RV Pelagia Cruise INDEX2018 Leg 2; geodeticDatum: WGS84; coordinateUncertaintyInMeters: 24; **Identification:** identifiedBy: Tina Molodtsova; identificationRemarks: Identified only from imagery; identificationQualifier: gen. indet.; **Event:** eventDate: 2018-11-24; eventTime: 10:50:56 am; year: 2018; fieldNumber: INDEX2018-65ROPOS; fieldNotes: 1.8°C; **Record Level:** language: en; institutionCode: DZMB; datasetName: INDEX; basisOfRecord: Human Observation

##### Notes

Fig. 102

#### Isididae gen. indet. (DZMB_2021_0041)

145DC8A4-017F-52B6-A952-D37D048F17B7

##### Materials

- **Type status:** Other material. **Occurrence:** recordedBy: ROPOS.COM; individualCount: 1; lifeStage: Adult; behavior: attached to basalt; occurrenceStatus: present; preparations: Imaged only; associatedMedia: R2092_00602.jpg; **Taxon:** taxonConceptID: Isididae gen. indet. (DZMB_2021_0041); kingdom: Animalia; phylum: Cnidaria; class: Anthozoa; order: Alcyonacea; family: Isididae; genus: -; taxonRank: Family; scientificNameAuthorship: Lamouroux, 1812; **Location:** waterBody: Indian Ocean; stateProvince: South East Indian Ridge; locality: Vent site 6; verbatimLocality: Cluster 12; maximumDepthInMeters: 2465; locationRemarks: RV Pelagia Cruise INDEX2018 Leg 2; geodeticDatum: WGS84; coordinateUncertaintyInMeters: 25; **Identification:** identifiedBy: Tina Molodtsova; identificationRemarks: Identified only from imagery; identificationQualifier: gen. indet.; **Event:** eventDate: 2018-11-20; eventTime: 12:20:38 pm; year: 2018; fieldNumber: INDEX2018-57ROPOS; fieldNotes: 1.8°C, 34.7 ppt; **Record Level:** language: en; institutionCode: DZMB; datasetName: INDEX; basisOfRecord: Human Observation

##### Notes

Fig. 103

#### Isididae gen. indet. (DZMB_2021_0042)

B03994D5-6AE8-5BF4-8B71-93DE2748245A

##### Materials

- **Type status:** Other material. **Occurrence:** recordedBy: ROPOS.COM; individualCount: 1; lifeStage: Adult; behavior: attached to basalt; occurrenceStatus: present; preparations: Imaged only; associatedMedia: R2097_00142.jpg; **Taxon:** taxonConceptID: Isididae gen. indet. (DZMB_2021_0042); kingdom: Animalia; phylum: Cnidaria; class: Anthozoa; order: Alcyonacea; family: Isididae; genus: -; taxonRank: Family; scientificNameAuthorship: Lamouroux, 1812; **Location:** waterBody: Indian Ocean; stateProvince: South East Indian Ridge; locality: Vent site 6; verbatimLocality: Cluster 12; maximumDepthInMeters: 2374; locationRemarks: RV Pelagia Cruise INDEX2018 Leg 2; geodeticDatum: WGS84; coordinateUncertaintyInMeters: 24; **Identification:** identifiedBy: Tina Molodtsova; identificationRemarks: Identified only from imagery; identificationQualifier: gen. indet.; **Event:** eventDate: 2018-11-25; eventTime: 8:13:14 am; year: 2018; fieldNumber: INDEX2018-67ROPOS; fieldNotes: 1.8°C; **Record Level:** language: en; institutionCode: DZMB; datasetName: INDEX; basisOfRecord: Human Observation

##### Notes

Fig. 104

#### Isididae gen. indet. (DZMB_2021_0043)

59CD786A-2A7C-5BF2-B8AF-1C5746B5FB79

##### Materials

- **Type status:** Other material. **Occurrence:** recordedBy: ROPOS.COM; individualCount: 1; lifeStage: Adult; behavior: attached to basalt; occurrenceStatus: present; preparations: Imaged only; associatedMedia: R2092_00603-2.jpg; **Taxon:** taxonConceptID: Isididae gen. indet. (DZMB_2021_0043); kingdom: Animalia; phylum: Cnidaria; class: Anthozoa; order: Alcyonacea; family: Isididae; genus: -; taxonRank: Family; scientificNameAuthorship: Lamouroux, 1812; **Location:** waterBody: Indian Ocean; stateProvince: South East Indian Ridge; locality: Vent site 6; verbatimLocality: Cluster 12; maximumDepthInMeters: 2465; locationRemarks: RV Pelagia Cruise INDEX2018 Leg 2; geodeticDatum: WGS84; coordinateUncertaintyInMeters: 25; **Identification:** identifiedBy: Tina Molodtsova; identificationRemarks: Identified only from imagery; identificationQualifier: gen. indet.; **Event:** eventDate: 2018-11-20; eventTime: 12:21:14 pm; year: 2018; fieldNumber: INDEX2018-57ROPOS; fieldNotes: 1.8°C, 34.7 ppt; **Record Level:** language: en; institutionCode: DZMB; datasetName: INDEX; basisOfRecord: Human Observation

##### Notes

Fig. 105

#### Isididae fam. inc. (DZMB_2021_0044)

5113E056-7536-57E6-9A2E-FFB4FEB88A4F

##### Materials

- **Type status:** Other material. **Occurrence:** recordedBy: ROPOS.COM; individualCount: 1; lifeStage: Adult; behavior: attached to basalt; occurrenceStatus: present; preparations: Imaged only; associatedMedia: R2092_00475.jpg; **Taxon:** taxonConceptID: Isididae fam. inc. (DZMB_2021_0044); kingdom: Animalia; phylum: Cnidaria; class: Anthozoa; order: Alcyonacea; family: Isididae; genus: -; taxonRank: Family; scientificNameAuthorship: Lamouroux, 1812; **Location:** waterBody: Indian Ocean; stateProvince: South East Indian Ridge; locality: Vent site 6; verbatimLocality: Cluster 12; maximumDepthInMeters: 2448; locationRemarks: RV Pelagia Cruise INDEX2018 Leg 2; geodeticDatum: WGS84; coordinateUncertaintyInMeters: 25; **Identification:** identifiedBy: Tina Molodtsova; identificationRemarks: Identified only from imagery; identificationQualifier: fam. inc.; **Event:** eventDate: 2018-11-20; eventTime: 8:36:21 am; year: 2018; fieldNumber: INDEX2018-57ROPOS; fieldNotes: 1.8°C, 34.7 ppt; **Record Level:** language: en; institutionCode: DZMB; datasetName: INDEX; basisOfRecord: Human Observation

##### Notes

Fig. 106

#### Isididae gen. indet. (DZMB_2021_0045)

D3D34A23-C691-53AA-ACB0-233BBC299B52

##### Materials

- **Type status:** Other material. **Occurrence:** recordedBy: BGR/ GEOMAR; individualCount: 1; lifeStage: Adult; behavior: on basalt; occurrenceStatus: present; preparations: Imaged only; associatedMedia: 2013-12-17_12-37-23_Sonne_INDEX2013-2_062ROV11_Logo.jpg; **Taxon:** taxonConceptID: Isididae gen. indet. (DZMB_2021_0045); kingdom: Animalia; phylum: Cnidaria; class: Anthozoa; order: Alcyonacea; family: Isididae; genus: -; taxonRank: Family; scientificNameAuthorship: Lamouroux, 1812; **Location:** waterBody: Indian Ocean; stateProvince: Central Indian Ridge; locality: MESO; verbatimLocality: outside INDEX claim; maximumDepthInMeters: 2828; locationRemarks: FS Sonne Cruise INDEX2013 Leg 2; geodeticDatum: WGS84; coordinateUncertaintyInMeters: 28; **Identification:** identifiedBy: Tina Molodtsova; identificationRemarks: Identified only from imagery - forked morphotype; identificationQualifier: gen. indet.; **Event:** eventDate: 2013-12-17; eventTime: 12:37:23 pm; year: 2013; fieldNumber: INDEX2013-62ROV; fieldNotes: 1.8°C, 34.4 ppt; **Record Level:** language: en; institutionCode: DZMB; datasetName: INDEX; basisOfRecord: Human Observation

##### Notes

Fig. 107

#### Acanella

Gray, 1870

E2F41864-60CF-5E42-93D4-429EC7F80371

#### Isididae Acanella gen. inc.

725DB422-687A-5A47-9FDC-FE24F47FF92D

##### Materials

- **Type status:** Other material. **Occurrence:** recordedBy: ROPOS.COM; individualCount: 1; lifeStage: Adult; behavior: attached to basalt; occurrenceStatus: present; preparations: Imaged only; associatedMedia: R2095_00087.jpg; associatedOccurrences: Goniasteridae gen. indet.; **Taxon:** taxonConceptID: Isididae Acanella gen. inc.; kingdom: Animalia; phylum: Cnidaria; class: Anthozoa; order: Alcyonacea; family: Isididae; genus: Acanella; taxonRank: Genus; scientificNameAuthorship: Gray, 1870; **Location:** waterBody: Indian Ocean; stateProvince: South East Indian Ridge; locality: Vent site 6; verbatimLocality: Cluster 12; maximumDepthInMeters: 2370; locationRemarks: RV Pelagia Cruise INDEX2018 Leg 2; geodeticDatum: WGS84; coordinateUncertaintyInMeters: 24; **Identification:** identifiedBy: Tina Molodtsova; identificationRemarks: Identified only from imagery; identificationQualifier: gen. inc.; **Event:** eventDate: 2018-11-23; eventTime: 6:50:03 am; year: 2018; fieldNumber: INDEX2018-63ROPOS; fieldNotes: 1.8°C; **Record Level:** language: en; institutionCode: DZMB; datasetName: INDEX; basisOfRecord: Human Observation

##### Notes

Fig. 108

#### Bathygorgia

Wright, 1885

7619F87B-DFA4-588E-B9E7-FB2F8D2F4D30

#### Isididae Bathygorgia gen. inc.

887C7103-9AA7-5204-B71B-8DE3B181926D

##### Materials

- **Type status:** Other material. **Occurrence:** recordedBy: ROPOS.COM; individualCount: 1; lifeStage: Adult; behavior: attached to basalt; occurrenceStatus: present; preparations: Imaged only; associatedMedia: R2105_00090.jpg; **Taxon:** taxonConceptID: Isididae Bathygorgia gen. inc.; kingdom: Animalia; phylum: Cnidaria; class: Anthozoa; order: Alcyonacea; family: Isididae; genus: Bathygorgia; taxonRank: Genus; scientificNameAuthorship: Wright, 1885; **Location:** waterBody: Indian Ocean; stateProvince: Rodriguez Triple Junction; locality: Vent site 4; verbatimLocality: Cluster 5; maximumDepthInMeters: 2663; locationRemarks: RV Pelagia Cruise INDEX2018 Leg 2; geodeticDatum: WGS84; coordinateUncertaintyInMeters: 26; **Identification:** identifiedBy: Tina Molodtsova; identificationRemarks: Identified only from imagery; identificationQualifier: gen. inc.; **Event:** eventDate: 2018-12-09; eventTime: 6:49:45 am; year: 2018; fieldNumber: INDEX2018-95ROPOS; fieldNotes: 1.8°C, 34.7 ppt; **Record Level:** language: en; institutionCode: DZMB; datasetName: INDEX; basisOfRecord: Human Observation

##### Notes

Fig. 109

#### Jasonisis

Alderslade & McFadden, 2012

D07F4438-4F90-51F0-BA4B-F262D09E0F55

#### Isididae Jasonisis gen. inc.

0E397393-1E9E-5A2A-A3DC-4FF224D40053

##### Materials

- **Type status:** Other material. **Occurrence:** recordedBy: ROPOS.COM; individualCount: 1; lifeStage: Adult; behavior: attached to basalt; occurrenceStatus: present; preparations: Imaged only; associatedMedia: R2093_00938.jpg; **Taxon:** taxonConceptID: Isididae Jasonisis gen. inc.; kingdom: Animalia; phylum: Cnidaria; class: Anthozoa; order: Alcyonacea; family: Isididae; genus: Jasonisis; taxonRank: Genus; scientificNameAuthorship: Alderslade & McFadden, 2012; **Location:** waterBody: Indian Ocean; stateProvince: South East Indian Ridge; locality: Vent site 6; verbatimLocality: Cluster 12; maximumDepthInMeters: 2368; locationRemarks: RV Pelagia Cruise INDEX2018 Leg 2; geodeticDatum: WGS84; coordinateUncertaintyInMeters: 23; **Identification:** identifiedBy: Tina Molodtsova; identificationRemarks: Identified only from imagery; identificationQualifier: gen. inc.; **Event:** eventDate: 2018-11-21; eventTime: 10:48:28 am; year: 2018; fieldNumber: INDEX2018-59ROPOS; fieldNotes: 1.8°C, 34.7 ppt; **Record Level:** language: en; institutionCode: DZMB; datasetName: INDEX; basisOfRecord: Human Observation

##### Notes

Fig. 110

#### Keratoisis

Wright, 1869

9F4D47C5-83F0-5EAE-8305-800A52CAD68E

#### Isididae Keratoisis gen. inc. (DZMB_2021_0046)

78071E06-2121-5A32-9ECB-B4B0CE9BF974

##### Materials

- **Type status:** Other material. **Occurrence:** recordedBy: ROPOS.COM; individualCount: 1; lifeStage: Adult; behavior: attached to basalt; occurrenceStatus: present; preparations: Imaged only; associatedMedia: R2105_00094.jpg; **Taxon:** taxonConceptID: Isididae Keratoisis gen. inc. (DZMB_2021_0046); kingdom: Animalia; phylum: Cnidaria; class: Anthozoa; order: Alcyonacea; family: Isididae; genus: Keratoisis; taxonRank: Genus; scientificNameAuthorship: Wright, 1869; **Location:** waterBody: Indian Ocean; stateProvince: Rodriguez Triple Junction; locality: Vent site 4; verbatimLocality: Cluster 5; maximumDepthInMeters: 2662; locationRemarks: RV Pelagia Cruise INDEX2018 Leg 2; geodeticDatum: WGS84; coordinateUncertaintyInMeters: 26; **Identification:** identifiedBy: Tina Molodtsova; identificationRemarks: Identified only from imagery; identificationQualifier: gen. inc.; **Event:** eventDate: 2018-12-09; eventTime: 6:50:43 am; year: 2018; fieldNumber: INDEX2018-95ROPOS; fieldNotes: 1.8°C, 34.7 ppt; **Record Level:** language: en; institutionCode: DZMB; datasetName: INDEX; basisOfRecord: Human Observation

##### Notes

Fig. 111

#### Isididae Keratoisis gen. inc. (DZMB_2021_0047)

BABE3DF0-5446-58DF-BD03-C9252A3E71A1

##### Materials

- **Type status:** Other material. **Occurrence:** recordedBy: ROPOS.COM; individualCount: 1; lifeStage: Adult; behavior: attached to basalt; occurrenceStatus: present; preparations: Imaged only; associatedMedia: R2105_00311.jpg; associatedOccurrences: Actinoscyphia sp. indet.; **Taxon:** taxonConceptID: Isididae Keratoisis gen. inc. (DZMB_2021_0047); kingdom: Animalia; phylum: Cnidaria; class: Anthozoa; order: Alcyonacea; family: Isididae; genus: Keratoisis; taxonRank: Genus; scientificNameAuthorship: Wright, 1869; **Location:** waterBody: Indian Ocean; stateProvince: Rodriguez Triple Junction; locality: Vent site 4; verbatimLocality: Cluster 5; maximumDepthInMeters: 2658; locationRemarks: RV Pelagia Cruise INDEX2018 Leg 2; geodeticDatum: WGS84; coordinateUncertaintyInMeters: 26; **Identification:** identifiedBy: Tina Molodtsova; identificationRemarks: Identified only from imagery; identificationQualifier: gen. inc.; **Event:** eventDate: 2018-12-09; eventTime: 9:20:43 am; year: 2018; fieldNumber: INDEX2018-95ROPOS; fieldNotes: 1.8°C, 34.7 ppt; **Record Level:** language: en; institutionCode: DZMB; datasetName: INDEX; basisOfRecord: Human Observation

##### Notes

Fig. 112

#### Lepidisis

Verrill, 1883

599B40B6-BC1B-5276-AB6C-0F8FF95BFF1A

#### Isididae Lepidisis gen. inc.

D5CDB5F3-5CFA-5B36-B965-911C51E58400

##### Materials

- **Type status:** Other material. **Occurrence:** recordedBy: ROPOS.COM; individualCount: 1; lifeStage: Adult; behavior: on basalt; occurrenceStatus: present; preparations: Imaged only; associatedMedia: R2098_00067.jpg; **Taxon:** taxonConceptID: Isididae Lepidisis gen. inc.; kingdom: Animalia; phylum: Cnidaria; class: Anthozoa; order: Alcyonacea; family: Isididae; genus: Lepidisis; taxonRank: Genus; scientificNameAuthorship: Verrill, 1883; **Location:** waterBody: Indian Ocean; stateProvince: South East Indian Ridge; locality: Vent site 3; verbatimLocality: Cluster 12; maximumDepthInMeters: 2483; locationRemarks: RV Pelagia Cruise INDEX2018 Leg 2; geodeticDatum: WGS84; coordinateUncertaintyInMeters: 26; **Identification:** identifiedBy: Tina Molodtsova; identificationRemarks: Identified only from imagery; identificationQualifier: gen. inc.; **Event:** eventDate: 2018-11-26; eventTime: 6:12:27 am; year: 2018; fieldNumber: INDEX2018-70ROPOS; fieldNotes: 1.8°C, 34.7 ppt; **Record Level:** language: en; institutionCode: DZMB; datasetName: INDEX; basisOfRecord: Human Observation

##### Notes

Fig. 113

#### Lepidisis spp. indet.

4705A03B-395C-5F62-AEB8-8A21BD4FDA5A

##### Materials

- **Type status:** Other material. **Occurrence:** recordedBy: ROPOS.COM; individualCount: 1; lifeStage: Adult; behavior: attached to basalt; occurrenceStatus: present; preparations: Imaged only; associatedMedia: R2102_00153.jpg; **Taxon:** taxonConceptID: Lepidisis spp. indet.; kingdom: Animalia; phylum: Cnidaria; class: Anthozoa; order: Alcyonacea; family: Isididae; genus: Lepidisis; taxonRank: Genus; scientificNameAuthorship: Verrill, 1883; **Location:** waterBody: Indian Ocean; stateProvince: South East Indian Ridge; locality: Vent site 5; verbatimLocality: Cluster 11; maximumDepthInMeters: 2969; locationRemarks: RV Pelagia Cruise INDEX2018 Leg 2; geodeticDatum: WGS84; coordinateUncertaintyInMeters: 30; **Identification:** identifiedBy: Tina Molodtsova; identificationRemarks: Identified only from imagery; identificationQualifier: spp. indet.; **Event:** eventDate: 2018-12-01; eventTime: 8:27:44 am; year: 2018; fieldNumber: INDEX2018-80ROPOS; fieldNotes: 1.8°C, 34.7 ppt; **Record Level:** language: en; institutionCode: DZMB; datasetName: INDEX; basisOfRecord: Human Observation

##### Notes

Fig. 114

#### Paragorgiidae

Kükenthal, 1916

A392D1A2-4815-5D38-9DAD-8E7B5FDB8098

#### Paragorgiidae fam. inc.

332E9754-A777-5A10-8062-79EDD7E954EC

##### Materials

- **Type status:** Other material. **Occurrence:** recordedBy: ROPOS.COM; individualCount: 1; lifeStage: Adult; behavior: attached to basalt; occurrenceStatus: present; preparations: Imaged only; associatedMedia: R2095_00011.jpg; **Taxon:** taxonConceptID: Paragorgiidae fam. inc.; kingdom: Animalia; phylum: Cnidaria; class: Anthozoa; order: Alcyonacea; family: Paragorgiidae; genus: -; taxonRank: Family; scientificNameAuthorship: Kükenthal, 1916; **Location:** waterBody: Indian Ocean; stateProvince: South East Indian Ridge; locality: Vent site 6; verbatimLocality: Cluster 12; maximumDepthInMeters: 2396; locationRemarks: RV Pelagia Cruise INDEX2018 Leg 2; geodeticDatum: WGS84; coordinateUncertaintyInMeters: 24; **Identification:** identifiedBy: Tina Molodtsova; identificationRemarks: Identified only from imagery; identificationQualifier: fam. inc.; **Event:** eventDate: 2018-11-23; eventTime: 5:40:24 am; year: 2018; fieldNumber: INDEX2018-63ROPOS; fieldNotes: 1.8°C; **Record Level:** language: en; institutionCode: DZMB; datasetName: INDEX; basisOfRecord: Human Observation

##### Notes

Fig. 115

#### Primnoidae

Milne Edwards, 1857

32BAA956-9C43-5F37-9884-4613B021F8A8

#### Primnoidae gen. indet. (DZMB_2021_0048)

7FA66A54-3929-5037-AC04-076CA29867D2

##### Materials

- **Type status:** Other material. **Occurrence:** recordedBy: ROPOS.COM; individualCount: 1; lifeStage: Adult; behavior: attached to basalt/ sulphides; occurrenceStatus: present; preparations: Imaged only; associatedMedia: R2107_00037.jpg; associatedOccurrences: Arthropoda cl. indet.; **Taxon:** taxonConceptID: Primnoidae gen. indet. (DZMB_2021_0048); kingdom: Animalia; phylum: Cnidaria; class: Anthozoa; order: Alcyonacea; family: Primnoidae; genus: -; taxonRank: Family; scientificNameAuthorship: Milne Edwards, 1857; **Location:** waterBody: Indian Ocean; stateProvince: Rodriguez Triple Junction; locality: Vent site 4; verbatimLocality: Cluster 5; maximumDepthInMeters: 2632; locationRemarks: RV Pelagia Cruise INDEX2018 Leg 2; geodeticDatum: WGS84; coordinateUncertaintyInMeters: 26; **Identification:** identifiedBy: Tina Molodtsova; identificationRemarks: Identified only from imagery - branched morphotype; identificationQualifier: gen. indet.; **Event:** eventDate: 2018-12-11; eventTime: 4:31:09 am; year: 2018; fieldNumber: INDEX2018-99ROPOS; fieldNotes: 1.8°C, 34.7 ppt; **Record Level:** language: en; institutionCode: DZMB; datasetName: INDEX; basisOfRecord: Human Observation

##### Notes

Fig. 116

#### Primnoidae gen. indet. (DZMB_2021_0049)

FFCF6318-800F-5240-B7D3-CDB39336C39F

##### Materials

- **Type status:** Other material. **Occurrence:** recordedBy: ROPOS.COM; individualCount: 1; lifeStage: Adult; behavior: on basalt; occurrenceStatus: present; preparations: Imaged only; associatedMedia: R2096_00253.jpg; **Taxon:** taxonConceptID: Primnoidae gen. indet. (DZMB_2021_0049); kingdom: Animalia; phylum: Cnidaria; class: Anthozoa; order: Alcyonacea; family: Primnoidae; genus: -; taxonRank: Family; scientificNameAuthorship: Milne Edwards, 1857; **Location:** waterBody: Indian Ocean; stateProvince: South East Indian Ridge; locality: Vent site 6; verbatimLocality: Cluster 12; maximumDepthInMeters: 2483; locationRemarks: RV Pelagia Cruise INDEX2018 Leg 2; geodeticDatum: WGS84; coordinateUncertaintyInMeters: 24; **Identification:** identifiedBy: Tina Molodtsova; identificationRemarks: Identified only from imagery - unbranched morphotype; identificationQualifier: gen. indet.; **Event:** eventDate: 2018-11-24; eventTime: 10:50:42 am; year: 2018; fieldNumber: INDEX2018-65ROPOS; fieldNotes: 1.8°C; **Record Level:** language: en; institutionCode: DZMB; datasetName: INDEX; basisOfRecord: Human Observation

##### Notes

Fig. 117

#### Alcyonacea/ Antipatharia

Lamouroux, 1812

8A994930-88ED-5826-92DD-80124C75DD74

#### Stalk of Alcyonacea or Antipatharia ord. inc.

AB6725F9-38D2-59C2-89C1-636B9C6E1B4B

##### Materials

- **Type status:** Other material. **Occurrence:** recordedBy: BGR; individualCount: 1; lifeStage: Adult; behavior: attached to basalt; occurrenceStatus: present; preparations: Imaged only; associatedMedia: 17MFT Fotos 2013-289_Stalk of Gorgonian.jpg; **Taxon:** taxonConceptID: Stalk of Alcyonacea or Antipatharia ord. inc.; kingdom: Animalia; phylum: Cnidaria; class: Anthozoa; order: Alcyonacea/ Antipatharia; family: -; genus: -; taxonRank: Order; scientificNameAuthorship: Lamouroux, 1812/ -; **Location:** waterBody: Indian Ocean; stateProvince: Central Indian Ridge; locality: MESO; verbatimLocality: outside INDEX claim; maximumDepthInMeters: 2816; locationRemarks: FS Sonne Cruise INDEX2013 Leg 1; decimalLatitude: -23.3919; decimalLongitude: 69.2420; geodeticDatum: WGS84; coordinateUncertaintyInMeters: 28; **Identification:** identifiedBy: Tina Molodtsova; identificationRemarks: Identified only from imagery; identificationQualifier: ord. inc.; **Event:** eventDate: 2013-11-25; eventTime: 3:27:11 am; year: 2013; fieldNumber: INDEX2013-17MFT; fieldNotes: 1.8°C, 34.7 ppt; **Record Level:** language: en; institutionCode: DZMB; datasetName: INDEX; basisOfRecord: Human Observation

##### Notes

Fig. 118

#### Antipatharia

36A423F9-6542-582C-B47B-9E187BA223BF

#### Cladopathidae

Kinoshita, 1910

4F29B4A9-C0EF-50B8-8416-1016E6A60D83

#### Heteropathes

Opresko, 2011

BD4BE27A-0BC7-510F-8B96-C1645A83C8EC

#### Heteropathes sp. indet.

BBA5E5D3-4713-5BC5-AD12-E4118BACD0B7

##### Materials

- **Type status:** Other material. **Occurrence:** recordedBy: ROPOS.COM; individualCount: 1; lifeStage: Adult; behavior: attached to basalt; occurrenceStatus: present; preparations: Imaged only; associatedMedia: R2095_00020.jpg; **Taxon:** taxonConceptID: Heteropathes sp. indet.; kingdom: Animalia; phylum: Cnidaria; class: Anthozoa; order: Antipatharia; family: Cladopathidae; genus: Heteropathes; taxonRank: Genus; scientificNameAuthorship: Opresko, 2011; **Location:** waterBody: Indian Ocean; stateProvince: South East Indian Ridge; locality: Vent site 6; verbatimLocality: Cluster 12; maximumDepthInMeters: 2385; locationRemarks: RV Pelagia Cruise INDEX2018 Leg 2; geodeticDatum: WGS84; coordinateUncertaintyInMeters: 24; **Identification:** identifiedBy: Tina Molodtsova; identificationRemarks: Identified only from imagery; identificationQualifier: sp. indet.; **Event:** eventDate: 2018-11-23; eventTime: 5:46:04 am; year: 2018; fieldNumber: INDEX2018-63ROPOS; fieldNotes: 1.8°C; **Record Level:** language: en; institutionCode: DZMB; datasetName: INDEX; basisOfRecord: Human Observation

##### Notes

Fig. 119

#### Heteropathes americana sp. inc.

(Opresko, 2003)

F234E57C-9D8E-5464-A972-9062F94457A2

##### Materials

- **Type status:** Other material. **Occurrence:** recordedBy: ROPOS.COM; individualCount: 1; lifeStage: Adult; behavior: attached to basalt; occurrenceStatus: present; preparations: Imaged only; associatedMedia: R2092_00428-2.jpg; **Taxon:** taxonConceptID: Heteropathesamericana sp. inc.; scientificName: Heteropathesamericana; kingdom: Animalia; phylum: Cnidaria; class: Anthozoa; order: Antipatharia; family: Cladopathidae; genus: Heteropathes; taxonRank: Species; scientificNameAuthorship: (Opresko, 2003); **Location:** waterBody: Indian Ocean; stateProvince: South East Indian Ridge; locality: Vent site 6; verbatimLocality: Cluster 12; maximumDepthInMeters: 2508; locationRemarks: RV Pelagia Cruise INDEX2018 Leg 2; geodeticDatum: WGS84; coordinateUncertaintyInMeters: 25; **Identification:** identifiedBy: Tina Molodtsova; identificationRemarks: Identified only from imagery; identificationQualifier: sp. inc.; **Event:** eventDate: 2018-11-20; eventTime: 7:46:49 am; year: 2018; fieldNumber: INDEX2018-57ROPOS; fieldNotes: 1.8°C, 34.7 ppt; **Record Level:** language: en; institutionCode: DZMB; datasetName: INDEX; basisOfRecord: Human Observation

##### Notes

Fig. 120

#### Schizopathidae

Brook, 1889

6E9078B8-A8C1-5619-9DD5-E9ECFDC6B1C5

#### Bathypathes

Brook, 1889

FB4A2EBD-D864-52F3-884B-8BD5791A9438

#### Bathypathes sp. indet. (DZMB_2021_0050)

F5987DE2-5FC1-59AF-AD1B-ACD839FFF048

##### Materials

- **Type status:** Other material. **Occurrence:** recordedBy: ROPOS.COM; individualCount: 1; lifeStage: Adult; behavior: attached to basalt; occurrenceStatus: present; preparations: Imaged only; associatedMedia: R2107_00045.jpg; **Taxon:** taxonConceptID: Bathypathes sp. indet. (DZMB_2021_0050); kingdom: Animalia; phylum: Cnidaria; class: Anthozoa; order: Antipatharia; family: Schizopathidae; genus: Bathypathes; taxonRank: Genus; scientificNameAuthorship: Brook, 1889; **Location:** waterBody: Indian Ocean; stateProvince: Rodriguez Triple Junction; locality: Vent site 4; verbatimLocality: Cluster 5; maximumDepthInMeters: 2662; locationRemarks: RV Pelagia Cruise INDEX2018 Leg 2; geodeticDatum: WGS84; coordinateUncertaintyInMeters: 26; **Identification:** identifiedBy: Tina Molodtsova; identificationRemarks: Identified only from imagery; identificationQualifier: sp. indet.; **Event:** eventDate: 2018-12-11; eventTime: 4:46:55 am; year: 2018; fieldNumber: INDEX2018-99ROPOS; fieldNotes: 1.8°C, 34.7 ppt; **Record Level:** language: en; institutionCode: DZMB; datasetName: INDEX; basisOfRecord: Human Observation

##### Notes

Fig. 121

#### Bathypathes gen. inc. (DZMB_2021_0051)

82CD20DF-AC66-5097-B055-A2AC77E81D1C

##### Materials

- **Type status:** Other material. **Occurrence:** recordedBy: ROPOS.COM; individualCount: 1; lifeStage: Adult; behavior: attached to basalt; occurrenceStatus: present; preparations: Imaged only; associatedMedia: R2097_00145-2.jpg; **Taxon:** taxonConceptID: Bathypathes gen. inc. (DZMB_2021_0051); kingdom: Animalia; phylum: Cnidaria; class: Anthozoa; order: Antipatharia; family: Schizopathidae; genus: Bathypathes; taxonRank: Genus; scientificNameAuthorship: Brook, 1889; **Location:** waterBody: Indian Ocean; stateProvince: South East Indian Ridge; locality: Vent site 6; verbatimLocality: Cluster 12; maximumDepthInMeters: 2374; locationRemarks: RV Pelagia Cruise INDEX2018 Leg 2; geodeticDatum: WGS84; coordinateUncertaintyInMeters: 24; **Identification:** identifiedBy: Tina Molodtsova; identificationRemarks: Identified only from imagery; identificationQualifier: gen. inc.; **Event:** eventDate: 2018-11-25; eventTime: 8:14:41 am; year: 2018; fieldNumber: INDEX2018-67ROPOS; fieldNotes: 1.8°C; **Record Level:** language: en; institutionCode: DZMB; datasetName: INDEX; basisOfRecord: Human Observation

##### Notes

Fig. 122

#### Bathypathes patula sp. inc.

Brook, 1889

61C033F0-BCA7-5D17-B9F9-6CA172E8050B

##### Materials

- **Type status:** Other material. **Occurrence:** recordedBy: ROPOS.COM; individualCount: 1; lifeStage: Adult; behavior: attached to basalt; occurrenceStatus: present; preparations: Imaged only; associatedMedia: R1908_00414.jpg; **Taxon:** taxonConceptID: Bathypathespatula sp. inc.; scientificName: Bathypathespatula; kingdom: Animalia; phylum: Cnidaria; class: Anthozoa; order: Antipatharia; family: Schizopathidae; genus: Bathypathes; taxonRank: Species; scientificNameAuthorship: Brook, 1889; **Location:** waterBody: Indian Ocean; stateProvince: Central Indian Ridge; locality: Vent site 1; verbatimLocality: Cluster 4; maximumDepthInMeters: 3065; locationRemarks: RV Pelagia Cruise INDEX2015 Leg 2; geodeticDatum: WGS84; coordinateUncertaintyInMeters: 30; **Identification:** identifiedBy: Tina Molodtsova; identificationRemarks: Identified only from imagery; identificationQualifier: sp. inc.; **Event:** eventDate: 2015-12-01; eventTime: 7:17:48 am; year: 2015; fieldNumber: INDEX2015-47ROV; fieldNotes: 1.8°C, 34.7 ppt; **Record Level:** language: en; institutionCode: DZMB; datasetName: INDEX; basisOfRecord: Human Observation

##### Notes

Fig. 123

#### Schizopathes

Brook, 1889

9565D81E-6036-51BC-A35A-184EF7FD98FB

#### Schizopathes spp. indet.

7444271D-4126-5029-91DD-C4A2484C5E07

##### Materials

- **Type status:** Other material. **Occurrence:** recordedBy: ROPOS.COM; individualCount: 1; lifeStage: Adult; behavior: attached to basalt; occurrenceStatus: present; preparations: Imaged only; associatedMedia: R2105_00337.jpg; **Taxon:** taxonConceptID: Schizopathes spp. indet.; kingdom: Animalia; phylum: Cnidaria; class: Anthozoa; order: Antipatharia; family: Schizopathidae; genus: Schizopathes; taxonRank: Genus; scientificNameAuthorship: Brook, 1889; **Location:** waterBody: Indian Ocean; stateProvince: Rodriguez Triple Junction; locality: Vent site 4; verbatimLocality: Cluster 5; maximumDepthInMeters: 2712; locationRemarks: RV Pelagia Cruise INDEX2018 Leg 2; geodeticDatum: WGS84; coordinateUncertaintyInMeters: 26; **Identification:** identifiedBy: Tina Molodtsova; identificationRemarks: Identified only from imagery; identificationQualifier: spp. indet.; **Event:** eventDate: 2018-12-09; eventTime: 10:19:38 am; year: 2018; fieldNumber: INDEX2018-95ROPOS; fieldNotes: 1.8°C, 34.7 ppt; **Record Level:** language: en; institutionCode: DZMB; datasetName: INDEX; basisOfRecord: Human Observation

##### Notes

Fig. 124

#### Pennatulacea

Verrill, 1865

929A373C-E2D8-529D-9B59-09D127340FDD

#### Pennatulacea ord. inc. (DZMB_2021_0052)

987DB3E2-4983-56A7-A360-9B843850B490

##### Materials

- **Type status:** Other material. **Occurrence:** recordedBy: BGR; individualCount: 1; lifeStage: Adult; behavior: on sediment; occurrenceStatus: present; preparations: Imaged only; associatedMedia: 17MFT Fotos 2013-139.jpg; **Taxon:** taxonConceptID: Pennatulacea ord. inc. (DZMB_2021_0052); kingdom: Animalia; phylum: Cnidaria; class: Anthozoa; order: Pennatulacea; family: -; genus: -; taxonRank: Order; scientificNameAuthorship: Verrill, 1865; **Location:** waterBody: Indian Ocean; stateProvince: Central Indian Ridge; locality: MESO; verbatimLocality: outside INDEX claim; maximumDepthInMeters: 2846; locationRemarks: FS Sonne Cruise INDEX2013 Leg 1; decimalLatitude: -23.3839; decimalLongitude: 69.2377; geodeticDatum: WGS84; coordinateUncertaintyInMeters: 28; **Identification:** identifiedBy: Tina Molodtsova; identificationRemarks: Identified only from imagery; identificationQualifier: ord. inc.; **Event:** eventDate: 2013-11-25; eventTime: 5:31:31 am; year: 2013; fieldNumber: INDEX2013-17MFT; fieldNotes: 1.8°C, 34.7 ppt; **Record Level:** language: en; institutionCode: DZMB; datasetName: INDEX; basisOfRecord: Human Observation

##### Notes

Fig. 125

#### Pennatulacea fam. indet. (DZMB_2021_0053)

EF1C8016-F52A-5CCF-9828-460496813705

##### Materials

- **Type status:** Other material. **Occurrence:** recordedBy: ROPOS.COM; individualCount: 1; lifeStage: Adult; behavior: on sediment; occurrenceStatus: present; preparations: Imaged only; associatedMedia: R1914_00241.jpg; **Taxon:** taxonConceptID: Pennatulacea fam. indet. (DZMB_2021_0053); kingdom: Animalia; phylum: Cnidaria; class: Anthozoa; order: Pennatulacea; family: -; genus: -; taxonRank: Order; scientificNameAuthorship: Verrill, 1865; **Location:** waterBody: Indian Ocean; stateProvince: Central Indian Ridge; locality: EGS; verbatimLocality: Cluster 4; maximumDepthInMeters: 3111; locationRemarks: RV Pelagia Cruise INDEX2015 Leg 2; decimalLatitude: -23.9343; decimalLongitude: 69.6114; geodeticDatum: WGS84; coordinateUncertaintyInMeters: 31; **Identification:** identifiedBy: Tina Molodtsova; identificationRemarks: Identified only from imagery; identificationQualifier: fam. indet.; **Event:** eventDate: 2015-12-07; eventTime: 9:32:22 am; year: 2015; fieldNumber: INDEX2015-60ROV; fieldNotes: 1.8°C, 34.7 ppt; **Record Level:** language: en; institutionCode: DZMB; datasetName: INDEX; basisOfRecord: Human Observation

##### Notes

Fig. 126

#### Kophobelemnidae

Gray, 1860

E1CF23C8-B592-5425-B2C5-E13483FD3B25

#### Kophobelemnon

Asbjörnsen, 1856

60091628-9102-5B0A-8AC8-9A7584D5CA18

#### Pennatulacea Kophobelemnon ord. inc.

00888B9A-3064-58C1-99CB-33DEA8DCBFBE

##### Materials

- **Type status:** Other material. **Occurrence:** recordedBy: BGR; individualCount: 1; lifeStage: Adult; behavior: attached to basalt; occurrenceStatus: present; preparations: Imaged only; associatedMedia: 17MFT Fotos 2013-417-2.jpg; **Taxon:** taxonConceptID: Pennatulacea Kophobelemnon ord. inc.; kingdom: Animalia; phylum: Cnidaria; class: Anthozoa; order: Pennatulacea; family: Kophobelemnidae; genus: Kophobelemnon; taxonRank: Genus; scientificNameAuthorship: Asbjörnsen, 1856; **Location:** waterBody: Indian Ocean; stateProvince: Central Indian Ridge; locality: MESO; verbatimLocality: Cluster 3; maximumDepthInMeters: 2825; locationRemarks: FS Sonne Cruise INDEX2013 Leg 1; decimalLatitude: -23.3926; decimalLongitude: 69.2426; geodeticDatum: WGS84; coordinateUncertaintyInMeters: 28; **Identification:** identifiedBy: Tina Molodtsova; identificationRemarks: Identified only from imagery; identificationQualifier: ord. inc.; **Event:** eventDate: 2013-11-25; eventTime: 3:15:06 am; year: 2013; fieldNumber: INDEX2013-17MFT; fieldNotes: 1.8°C, 34.7 ppt; **Record Level:** language: en; institutionCode: DZMB; datasetName: INDEX; basisOfRecord: Human Observation

##### Notes

Fig. 127

#### Umbellulidae

Kölliker, 1880

7B1587A1-ABEE-57BE-A06D-B0B5244BE3B2

#### Umbellula

Gray, 1870

92ACF397-EA1B-58AB-BCD6-48C380F7D1D3

#### Umbellula sp. indet. (DZMB_2021_0054)

4B5D5EBC-D9F3-57E4-983F-E76286E92A7D

##### Materials

- **Type status:** Other material. **Occurrence:** recordedBy: BGR; individualCount: 1; lifeStage: Adult; behavior: on sediment; occurrenceStatus: present; preparations: Imaged only; associatedMedia: 38MFT Fotos 2013-160.jpg; **Taxon:** taxonConceptID: Umbellula sp. indet. (DZMB_2021_0054); kingdom: Animalia; phylum: Cnidaria; class: Anthozoa; order: Pennatulacea; family: Umbellulidae; genus: Umbellula; taxonRank: Genus; scientificNameAuthorship: Gray, 1870; **Location:** waterBody: Indian Ocean; stateProvince: Central Indian Ridge; locality: Edmond; verbatimLocality: Cluster 4; maximumDepthInMeters: 3271; locationRemarks: FS Sonne Cruise INDEX2013 Leg 2; decimalLatitude: -23.8781; decimalLongitude: 69.6004; geodeticDatum: WGS84; coordinateUncertaintyInMeters: 33; **Identification:** identifiedBy: Tina Molodtsova; identificationRemarks: Identified only from imagery; identificationQualifier: sp. indet.; **Event:** eventDate: 2013-12-09; eventTime: 1:12:01 am; year: 2013; fieldNumber: INDEX2013-38MFT; fieldNotes: 1.8°C, 34.7 ppt; **Record Level:** language: en; institutionCode: DZMB; datasetName: INDEX; basisOfRecord: Human Observation

##### Notes

Fig. 128

#### Umbellula sp. indet. (DZMB_2021_0055)

86073267-3381-5ABE-9C2E-B3EE6E3C1882

##### Materials

- **Type status:** Other material. **Occurrence:** recordedBy: ROPOS.COM; individualCount: 1; lifeStage: Adult; behavior: on sediment; occurrenceStatus: present; preparations: Imaged only; associatedMedia: R2104_00100.jpg; **Taxon:** taxonConceptID: Umbellula sp. indet. (DZMB_2021_0055); kingdom: Animalia; phylum: Cnidaria; class: Anthozoa; order: Pennatulacea; family: Umbellulidae; genus: Umbellula; taxonRank: Genus; scientificNameAuthorship: Gray, 1870; **Location:** waterBody: Indian Ocean; stateProvince: Rodriguez Triple Junction; locality: Vent site 4; verbatimLocality: Cluster 5; maximumDepthInMeters: 2541; locationRemarks: RV Pelagia Cruise INDEX2018 Leg 2; geodeticDatum: WGS84; coordinateUncertaintyInMeters: 25; **Identification:** identifiedBy: Tina Molodtsova; identificationRemarks: Identified only from imagery; identificationQualifier: sp. indet.; **Event:** eventDate: 2018-12-04; eventTime: 7:57:30 am; year: 2018; fieldNumber: INDEX2018-85ROPOS; fieldNotes: 1.8°C, 34.7 ppt; **Record Level:** language: en; institutionCode: DZMB; datasetName: INDEX; basisOfRecord: Human Observation

##### Notes

Fig. 129

#### Zoantharia

Gray, 1832

8162D958-35FE-5218-94A9-367EC8A0797A

#### Zoantharia fam. indet. (DZMB_2021_0056)

FFCEE15E-5B64-5702-8902-C6E35A038E83

##### Materials

- **Type status:** Other material. **Occurrence:** recordedBy: ROPOS.COM; individualCount: 100; lifeStage: Adult; behavior: attached to basalt; occurrenceStatus: present; preparations: Imaged only; associatedMedia: R2098_00275-2.jpg; **Taxon:** taxonConceptID: Zoantharia fam. indet. (DZMB_2021_0056); kingdom: Animalia; phylum: Cnidaria; class: Anthozoa; order: Zoantharia; family: -; genus: -; taxonRank: Order; scientificNameAuthorship: Gray, 1832; **Location:** waterBody: Indian Ocean; stateProvince: South East Indian Ridge; locality: Vent site 3; verbatimLocality: Cluster 12; maximumDepthInMeters: 2547; locationRemarks: RV Pelagia Cruise INDEX2018 Leg 2; geodeticDatum: WGS84; coordinateUncertaintyInMeters: 26; **Identification:** identifiedBy: Tina Molodtsova; identificationRemarks: Identified only from imagery; identificationQualifier: fam. indet.; **Event:** eventDate: 2018-11-26; eventTime: 10:39:16 am; year: 2018; fieldNumber: INDEX2018-70ROPOS; fieldNotes: 1.8°C, 34.8 ppt; **Record Level:** language: en; institutionCode: DZMB; datasetName: INDEX; basisOfRecord: Human Observation

##### Notes

Fig. 130

#### Zoantharia fam. indet. (DZMB_2021_0057)

96A212C5-A028-53CA-931D-A683B8577B8A

##### Materials

- **Type status:** Other material. **Occurrence:** recordedBy: ROPOS.COM; individualCount: 100; lifeStage: Adult; behavior: attached to sulphides/ basalt; occurrenceStatus: present; preparations: Imaged only; associatedMedia: R2096_00102.jpg; **Taxon:** taxonConceptID: Zoantharia fam. indet. (DZMB_2021_0057); kingdom: Animalia; phylum: Cnidaria; class: Anthozoa; order: Zoantharia; family: -; genus: -; taxonRank: Order; scientificNameAuthorship: Gray, 1832; **Location:** waterBody: Indian Ocean; stateProvince: South East Indian Ridge; locality: Vent site 6; verbatimLocality: Cluster 12; maximumDepthInMeters: 2431; locationRemarks: RV Pelagia Cruise INDEX2018 Leg 2; geodeticDatum: WGS84; coordinateUncertaintyInMeters: 24; **Identification:** identifiedBy: Tina Molodtsova; identificationRemarks: Identified only from imagery; identificationQualifier: fam. indet.; **Event:** eventDate: 2018-11-24; eventTime: 7:24:03 am; year: 2018; fieldNumber: INDEX2018-65ROPOS; fieldNotes: 1.8°C, 34.7 ppt; **Record Level:** language: en; institutionCode: DZMB; datasetName: INDEX; basisOfRecord: Human Observation

##### Notes

Fig. 131

#### Zoantharia fam. indet. (DZMB_2021_0058)

04B98235-9A10-583B-AC65-BCF4CFA84FF7

##### Materials

- **Type status:** Other material. **Occurrence:** recordedBy: ROPOS.COM; individualCount: 100; lifeStage: Adult; behavior: attached to sulphides; occurrenceStatus: present; preparations: Imaged only; associatedMedia: R2097_00212-3.jpg; **Taxon:** taxonConceptID: Zoantharia fam. indet. (DZMB_2021_0058); kingdom: Animalia; phylum: Cnidaria; class: Anthozoa; order: Zoantharia; family: -; genus: -; taxonRank: Order; scientificNameAuthorship: Gray, 1832; **Location:** waterBody: Indian Ocean; stateProvince: South East Indian Ridge; locality: Vent site 6; verbatimLocality: Cluster 12; maximumDepthInMeters: 2489; locationRemarks: RV Pelagia Cruise INDEX2018 Leg 2; geodeticDatum: WGS84; coordinateUncertaintyInMeters: 24; **Identification:** identifiedBy: Tina Molodtsova; identificationRemarks: Identified only from imagery; identificationQualifier: fam. indet.; **Event:** eventDate: 2018-11-25; eventTime: 10:06:31 am; year: 2018; fieldNumber: INDEX2018-67ROPOS; fieldNotes: 1.8°C, 34.7 ppt; **Record Level:** language: en; institutionCode: DZMB; datasetName: INDEX; basisOfRecord: Human Observation

##### Notes

Fig. 132

#### Epizoanthidae

Delage & Hérouard, 1901

940B170A-27DF-5660-8BDF-A673DC3520F8

#### Epizoanthus

Gray, 1867

C3C8C292-A538-5AB2-8F16-686751DB890A

#### Epizoanthus sp. indet.

9422BEC5-1688-5047-8B2F-3FE128FB1B75

##### Materials

- **Type status:** Other material. **Occurrence:** recordedBy: ROPOS.COM; individualCount: 1; lifeStage: Adult; behavior: in symbiosis with hermit crab; occurrenceStatus: present; preparations: Imaged only; associatedMedia: R1905_00014.jpg; associatedOccurrences: Paguroidea superfam. inc.; **Taxon:** taxonConceptID: Epizoanthus sp. indet.; kingdom: Animalia; phylum: Cnidaria; class: Anthozoa; order: Zoantharia; family: Epizoanthidae; genus: Epizoanthus; taxonRank: Genus; scientificNameAuthorship: Gray, 1867; **Location:** waterBody: Indian Ocean; stateProvince: Central Indian Ridge; locality: Vent site 1; verbatimLocality: Cluster 4; maximumDepthInMeters: 3072; locationRemarks: RV Pelagia Cruise INDEX2015 Leg 2; geodeticDatum: WGS84; coordinateUncertaintyInMeters: 30; **Identification:** identifiedBy: Tina Molodtsova; identificationRemarks: Identified only from imagery; identificationQualifier: sp. indet.; **Event:** eventDate: 2015-11-27; eventTime: 9:20:39 am; year: 2015; fieldNumber: INDEX2015-37ROV; fieldNotes: 1.8°C, 34.7 ppt; **Record Level:** language: en; institutionCode: DZMB; datasetName: INDEX; basisOfRecord: Human Observation

##### Notes

Fig. 133

#### Hydrozoa

Owen, 1843

C4CE8E15-A525-54B4-95D5-D69DE0303AAE

#### Hydrozoa ord. indet. (DZMB_2021_0059)

3C8CF86A-B76E-5F5E-BDDA-6E1AD70FF368

##### Materials

- **Type status:** Other material. **Occurrence:** recordedBy: ROPOS.COM; individualCount: 3; lifeStage: Adult; behavior: attached to sulphides; occurrenceStatus: present; preparations: Imaged only; associatedMedia: R2095_00138-2.jpg; **Taxon:** taxonConceptID: Hydrozoa ord. indet. (DZMB_2021_0059); kingdom: Animalia; phylum: Cnidaria; class: Hydrozoa; taxonRank: Class; scientificNameAuthorship: Owen, 1843; **Location:** waterBody: Indian Ocean; stateProvince: South East Indian Ridge; locality: Vent site 6; verbatimLocality: Cluster 12; maximumDepthInMeters: 2471; locationRemarks: RV Pelagia Cruise INDEX2018 Leg 2; geodeticDatum: WGS84; coordinateUncertaintyInMeters: 24; **Identification:** identifiedBy: Tina Molodtsova; identificationRemarks: Identified only from imagery; identificationQualifier: ord. indet.; **Event:** eventDate: 2018-11-23; eventTime: 9:45:31 am; year: 2018; fieldNumber: INDEX2018-63ROPOS; fieldNotes: 1.8°C; **Record Level:** language: en; institutionCode: DZMB; datasetName: INDEX; basisOfRecord: Human Observation

##### Notes

Fig. 134

#### Hydrozoa ord. indet. (DZMB_2021_0060)

0C6C5834-23BE-577C-ABE2-70C7D6FA912D

##### Materials

- **Type status:** Other material. **Occurrence:** recordedBy: ROPOS.COM; individualCount: 100; lifeStage: Adult; behavior: attached to sulphides; occurrenceStatus: present; preparations: Imaged only; associatedMedia: R2107_00010.jpg; **Taxon:** taxonConceptID: Hydrozoa ord. indet. (DZMB_2021_0060); kingdom: Animalia; phylum: Cnidaria; class: Hydrozoa; taxonRank: Class; scientificNameAuthorship: Owen, 1843; **Location:** waterBody: Indian Ocean; stateProvince: Rodriguez Triple Junction; locality: Vent site 4; verbatimLocality: Cluster 5; maximumDepthInMeters: 2628; locationRemarks: RV Pelagia Cruise INDEX2018 Leg 2; geodeticDatum: WGS84; coordinateUncertaintyInMeters: 26; **Identification:** identifiedBy: Tina Molodtsova; identificationRemarks: Identified only from imagery; identificationQualifier: ord. indet.; **Event:** eventDate: 2018-12-11; eventTime: 3:38:22 am; year: 2018; fieldNumber: INDEX2018-99ROPOS; fieldNotes: 1.8°C, 34.7 ppt; **Record Level:** language: en; institutionCode: DZMB; datasetName: INDEX; basisOfRecord: Human Observation

##### Notes

Fig. 135

#### Hydrozoa ord. indet. (DZMB_2021_0061)

AA16C1AF-285B-5138-847C-C31BF75E0F7C

##### Materials

- **Type status:** Other material. **Occurrence:** recordedBy: ROPOS.COM; individualCount: 100; lifeStage: Adult; behavior: attached to sulphides; occurrenceStatus: present; preparations: Imaged only; associatedMedia: R2107_00117-3.jpg; **Taxon:** taxonConceptID: Hydrozoa ord. indet. (DZMB_2021_0061); kingdom: Animalia; phylum: Cnidaria; class: Hydrozoa; order: -; taxonRank: Class; scientificNameAuthorship: Owen, 1843; **Location:** waterBody: Indian Ocean; stateProvince: Rodriguez Triple Junction; locality: Vent site 4; verbatimLocality: Cluster 5; maximumDepthInMeters: 2629; locationRemarks: RV Pelagia Cruise INDEX2018 Leg 2; geodeticDatum: WGS84; coordinateUncertaintyInMeters: 26; **Identification:** identifiedBy: Tina Molodtsova; identificationRemarks: Identified only from imagery; identificationQualifier: ord. indet.; **Event:** eventDate: 2018-12-11; eventTime: 8:30:44 am; year: 2018; fieldNumber: INDEX2018-99ROPOS; fieldNotes: 1.8°C, 34.7 ppt; **Record Level:** language: en; institutionCode: DZMB; datasetName: INDEX; basisOfRecord: Human Observation

##### Notes

Fig. 136

#### Hydrozoa ord. indet. (DZMB_2021_0062)

26E70AE4-FFBE-5978-8F2C-EBCB3B5692A1

##### Materials

- **Type status:** Other material. **Occurrence:** recordedBy: ROPOS.COM; individualCount: 2; lifeStage: Adult; behavior: attached to sulphides; occurrenceStatus: present; preparations: Imaged only; associatedMedia: R2096_00253-2.jpg; **Taxon:** taxonConceptID: Hydrozoa ord. indet. (DZMB_2021_0062); kingdom: Animalia; phylum: Cnidaria; class: Hydrozoa; taxonRank: Class; scientificNameAuthorship: Owen, 1843; **Location:** waterBody: Indian Ocean; stateProvince: South East Indian Ridge; locality: Vent site 6; verbatimLocality: Cluster 12; maximumDepthInMeters: 2483; locationRemarks: RV Pelagia Cruise INDEX2018 Leg 2; geodeticDatum: WGS84; coordinateUncertaintyInMeters: 24; **Identification:** identifiedBy: Tina Molodtsova; identificationRemarks: Identified only from imagery; identificationQualifier: ord. indet.; **Event:** eventDate: 2018-11-24; eventTime: 10:50:42 am; year: 2018; fieldNumber: INDEX2018-65ROPOS; fieldNotes: 1.8°C; **Record Level:** language: en; institutionCode: DZMB; datasetName: INDEX; basisOfRecord: Human Observation

##### Notes

Fig. 137

#### Hydrozoa ord. indet. (DZMB_2021_0063)

5AD4BDCB-4C6C-51FB-B7E3-9775E5159553

##### Materials

- **Type status:** Other material. **Occurrence:** recordedBy: ROPOS.COM; individualCount: 1; lifeStage: Adult; behavior: attached to basalt; occurrenceStatus: present; preparations: Imaged only; associatedMedia: R2107_00097.jpg; associatedOccurrences: Glyptelasma gen. inc.; **Taxon:** taxonConceptID: Hydrozoa ord. indet. (DZMB_2021_0063); kingdom: Animalia; phylum: Cnidaria; class: Hydrozoa; taxonRank: Class; scientificNameAuthorship: Owen, 1843; **Location:** waterBody: Indian Ocean; stateProvince: Rodriguez Triple Junction; locality: Vent site 4; verbatimLocality: Cluster 5; maximumDepthInMeters: 2638; locationRemarks: RV Pelagia Cruise INDEX2018 Leg 2; geodeticDatum: WGS84; coordinateUncertaintyInMeters: 26; **Identification:** identifiedBy: Tina Molodtsova; identificationRemarks: Identified only from imagery; identificationQualifier: ord. indet.; **Event:** eventDate: 2018-12-11; eventTime: 8:12:05 am; year: 2018; fieldNumber: INDEX2018-99ROPOS; fieldNotes: 1.8°C, 34.7 ppt; **Record Level:** language: en; institutionCode: DZMB; datasetName: INDEX; basisOfRecord: Human Observation

##### Notes

Fig. 138

#### Hydrozoa ord. indet. (DZMB_2021_0064)

159D9E39-DC93-5CA9-8BB3-1956503ECDC5

##### Materials

- **Type status:** Other material. **Occurrence:** recordedBy: ROPOS.COM; individualCount: 100; lifeStage: Adult; behavior: attached to basalt; occurrenceStatus: present; preparations: Imaged only; associatedMedia: R2107_00117.jpg; **Taxon:** taxonConceptID: Hydrozoa ord. indet. (DZMB_2021_0064); kingdom: Animalia; phylum: Cnidaria; class: Hydrozoa; taxonRank: Class; scientificNameAuthorship: Owen, 1843; **Location:** waterBody: Indian Ocean; stateProvince: Rodriguez Triple Junction; locality: Vent site 4; verbatimLocality: Cluster 5; maximumDepthInMeters: 2629; locationRemarks: RV Pelagia Cruise INDEX2018 Leg 2; geodeticDatum: WGS84; coordinateUncertaintyInMeters: 26; **Identification:** identifiedBy: Tina Molodtsova; identificationRemarks: Identified only from imagery; identificationQualifier: ord. indet.; **Event:** eventDate: 2018-12-11; eventTime: 8:30:44 am; year: 2018; fieldNumber: INDEX2018-99ROPOS; fieldNotes: 1.8°C, 34.7 ppt; **Record Level:** language: en; institutionCode: DZMB; datasetName: INDEX; basisOfRecord: Human Observation

##### Notes

Fig. 139

#### Hydrozoa ord. indet. (DZMB_2021_0065)

D9320715-1565-5628-AECD-F6AE56437CFA

##### Materials

- **Type status:** Other material. **Occurrence:** recordedBy: ROPOS.COM; individualCount: 1; lifeStage: Adult; behavior: on sediment; occurrenceStatus: present; preparations: Imaged only; associatedMedia: R2103_00133.jpg; **Taxon:** taxonConceptID: Hydrozoa ord. indet. (DZMB_2021_0065); kingdom: Animalia; phylum: Cnidaria; class: Hydrozoa; taxonRank: Class; scientificNameAuthorship: Owen, 1843; **Location:** waterBody: Indian Ocean; stateProvince: Rodriguez Triple Junction; locality: RTJ; verbatimLocality: Cluster 5; maximumDepthInMeters: 2515; locationRemarks: RV Pelagia Cruise INDEX2018 Leg 2; geodeticDatum: WGS84; coordinateUncertaintyInMeters: 25; **Identification:** identifiedBy: Tina Molodtsova; identificationRemarks: Identified only from imagery; identificationQualifier: ord. indet.; **Event:** eventDate: 2018-12-03; eventTime: 7:15:59 am; year: 2018; fieldNumber: INDEX2018-82ROPOS; fieldNotes: 1.8°C, 34.7 ppt; **Record Level:** language: en; institutionCode: DZMB; datasetName: INDEX; basisOfRecord: Human Observation

##### Notes

Fig. 140

#### Anthoathecata

Cornelius, 1992

625B5466-7F06-5858-AB96-F79A3AEA2197

#### Candelabridae

Stechow, 1921

2838266C-F6D5-54FD-A76C-55BEF1F92BA7

#### Candelabrum

de Blainville, 1830

11D41AAA-076E-5DF9-B81F-3A2805AC6584

#### Candelabrum sp. indet.

8E8E5F71-F1DD-5A28-A3AB-2A40744DC310

##### Materials

- **Type status:** Other material. **Occurrence:** recordedBy: ROPOS.COM; individualCount: 4; lifeStage: Adult; behavior: attached to basalt/sulphides; occurrenceStatus: present; preparations: Imaged only; associatedMedia: R1913_01914.jpg; **Taxon:** taxonConceptID: Candelabrum sp. indet.; kingdom: Animalia; phylum: Cnidaria; class: Hydrozoa; order: Anthoathecata; family: Candelabridae; genus: Candelabrum; taxonRank: Genus; scientificNameAuthorship: de Blainville, 1830; **Location:** waterBody: Indian Ocean; stateProvince: Central Indian Ridge; locality: EGS; verbatimLocality: Cluster 4; maximumDepthInMeters: 3345; locationRemarks: RV Pelagia Cruise INDEX2015 Leg 2; geodeticDatum: WGS84; coordinateUncertaintyInMeters: 33; **Identification:** identifiedBy: Tina Molodtsova; identificationRemarks: Identified only from imagery; identificationQualifier: sp. indet.; **Event:** eventDate: 2015-12-06; eventTime: 10:25:54 am; year: 2015; fieldNumber: INDEX2015-58ROV; fieldNotes: 1.8°C, 34.7 ppt; **Record Level:** language: en; institutionCode: DZMB; datasetName: INDEX; basisOfRecord: Human Observation

##### Notes

Fig. 141

#### Corymorphidae

Allman, 1872

28BF6B86-6D93-5DEC-B9A4-DA9E702CA137

#### Corymorphidae gen. indet.

ECA4EB52-AFB9-5AA5-BB1D-1554E89D06D9

##### Materials

- **Type status:** Other material. **Occurrence:** recordedBy: ROPOS.COM; individualCount: 1; lifeStage: Adult; behavior: attached to basalt; occurrenceStatus: present; preparations: Imaged only; associatedMedia: R2101_00040-3.jpg; **Taxon:** taxonConceptID: Corymorphidae gen. indet.; kingdom: Animalia; phylum: Cnidaria; class: Hydrozoa; order: Anthoathecata; family: Corymorphidae; genus: -; taxonRank: Family; scientificNameAuthorship: Allman, 1872; **Location:** waterBody: Indian Ocean; stateProvince: South East Indian Ridge; locality: Vent site 5; verbatimLocality: Cluster 11; maximumDepthInMeters: 2909; locationRemarks: RV Pelagia Cruise INDEX2018 Leg 2; geodeticDatum: WGS84; coordinateUncertaintyInMeters: 29; **Identification:** identifiedBy: Tina Molodtsova; identificationRemarks: Identified only from imagery; identificationQualifier: gen. indet.; **Event:** eventDate: 2018-11-29; eventTime: 6:44:09 am; year: 2018; fieldNumber: INDEX2018-75ROPOS; fieldNotes: 1.8°C, 34.7 ppt; **Record Level:** language: en; institutionCode: DZMB; datasetName: INDEX; basisOfRecord: Human Observation

##### Notes

Fig. 142

#### Siphonophorae

Eschscholtz, 1829

F9D8189A-1527-56FD-AB4E-223E5728C3C1

#### Rhodaliidae

Haeckel, 1888

9282056A-56E3-5A3F-96BA-9242E00C2041

#### Thermopalia

Pugh, 1983

277BABC2-C15F-5EA7-B536-C212DB3EEA53

#### Siphonophorae Rhodaliidae Thermopalia gen. inc.

394DBCDA-4094-50F0-B572-2B4C4F4DEBE8

##### Materials

- **Type status:** Other material. **Occurrence:** recordedBy: ROPOS.COM; individualCount: 1; lifeStage: Adult; behavior: attached to basalt; occurrenceStatus: present; preparations: Imaged only; associatedMedia: R2103_00254.jpg; **Taxon:** taxonConceptID: Siphonophorae Rhodaliidae Thermopalia gen. inc.; kingdom: Animalia; phylum: Cnidaria; class: Hydrozoa; order: Siphonophorae; family: Rhodaliidae; genus: Thermopalia; taxonRank: Genus; scientificNameAuthorship: Pugh, 1983; **Location:** waterBody: Indian Ocean; stateProvince: Rodriguez Triple Junction; locality: RTJ; verbatimLocality: Cluster 5; maximumDepthInMeters: 2665; locationRemarks: RV Pelagia Cruise INDEX2018 Leg 2; geodeticDatum: WGS84; coordinateUncertaintyInMeters: 25; **Identification:** identifiedBy: Tina Molodtsova; identificationRemarks: Identified only from imagery; identificationQualifier: gen. inc.; **Event:** eventDate: 2018-12-03; eventTime: 11:05:38 am; year: 2018; fieldNumber: INDEX2018-82ROPOS; fieldNotes: 1.8°C, 34.7 ppt; **Record Level:** language: en; institutionCode: DZMB; datasetName: INDEX; basisOfRecord: Human Observation

##### Notes

Fig. 143

#### Echinodermata

Bruguiere, 1791 [ex Klein, 1734]

DB0540EA-606C-5F7F-97BC-FF19330A5A09

#### Asteroidea

de Blainville, 1830

5C57B4F1-7490-50C6-BBE2-97BD648ABBB9

#### Brisingida

Fisher, 1928

7B0FD243-CE03-5C06-B8B1-29840D5D0C22

#### Brisingidae

G. O. Sars, 1875

F0CAD20B-71EC-5AA8-A251-B89CF750E40C

#### Hymenodiscus

Perrier, 1884

00C01DEA-6F34-54B9-91C3-64E9D9EF71BA

#### Hymenodiscus gen. inc.

9B729A4E-F8A8-5DF1-BA82-348C49F27934

##### Materials

- **Type status:** Other material. **Occurrence:** recordedBy: ROPOS.COM; individualCount: 1; lifeStage: Adult; behavior: on seafloor; occurrenceStatus: present; preparations: Imaged only; associatedMedia: R2103_00100.jpg; **Taxon:** taxonConceptID: Hymenodiscus gen. inc.; kingdom: Animalia; phylum: Echinodermata; class: Asteroidea; order: Brisingida; family: Brisingidae; genus: Hymenodiscus; taxonRank: Genus; scientificNameAuthorship: Perrier, 1884; **Location:** waterBody: Indian Ocean; stateProvince: Rodriguez Triple Junction; locality: RTJ; verbatimLocality: Cluster 5; maximumDepthInMeters: 2505; locationRemarks: RV Pelagia Cruise INDEX2018 Leg 2; geodeticDatum: WGS84; coordinateUncertaintyInMeters: 25; **Identification:** identifiedBy: Christopher Mah; identificationRemarks: Identified only from imagery; identificationQualifier: gen. inc.; **Event:** eventDate: 2018-12-03; eventTime: 6:30:22 am; year: 2018; fieldNumber: INDEX2018-82ROPOS; fieldNotes: 1.8°C, 34.7 ppt; **Record Level:** language: en; institutionCode: DZMB; datasetName: INDEX; basisOfRecord: Human Observation

##### Notes

Fig. 144

#### Freyellidae

Downey, 1986

3749AF05-74EB-5B0E-BB2E-EDE62127716F

#### Freyellidae fam. inc.

343B0ABD-9622-5240-B8DA-9B5D750148C8

##### Materials

- **Type status:** Other material. **Occurrence:** recordedBy: IFREMER; individualCount: 1; lifeStage: Adult; behavior: on basalt; occurrenceStatus: present; preparations: Imaged only; associatedMedia: 160119094531155_01_1080i copy.jpg; **Taxon:** taxonConceptID: Freyellidae fam. inc.; kingdom: Animalia; phylum: Echinodermata; class: Asteroidea; order: Brisingida; family: Freyellidae; taxonRank: Family; scientificNameAuthorship: Downey, 1986; **Location:** waterBody: Indian Ocean; stateProvince: South East Indian Ridge; locality: Pelagia; verbatimLocality: Cluster 8; maximumDepthInMeters: 3668; locationRemarks: RV Pourqoui pas? Cruise INDEX2016 Leg 1; geodeticDatum: WGS84; coordinateUncertaintyInMeters: 37; **Identification:** identifiedBy: Christopher Mah; identificationRemarks: Identified only from imagery; identificationQualifier: fam. inc.; **Event:** eventDate: 2016-01-19; eventTime: 9:45:31 am; year: 2016; fieldNumber: INDEX2016-16ROV; fieldNotes: 1.7°C, 34.7 ppt; **Record Level:** language: en; institutionCode: DZMB; datasetName: INDEX; basisOfRecord: Human Observation

##### Notes

Fig. 145

#### Freyastera

Downey, 1986

45FE4FC3-1E66-5EC3-8CF0-2FF850242119

#### Freyastera gen. inc.

9AD88F7A-F6CF-5A71-9E4E-8CFCDFA6AD76

##### Materials

- **Type status:** Other material. **Occurrence:** recordedBy: BGR/ GEOMAR; individualCount: 1; lifeStage: Adult; behavior: on sediment; occurrenceStatus: present; preparations: Imaged only; associatedMedia: 2013-12-12_10-48-06_Sonne_INDEX2013-2_049ROV06_Logo.jpg; **Taxon:** taxonConceptID: Freyastera gen. inc.; kingdom: Animalia; phylum: Echinodermata; class: Asteroidea; order: Brisingida; family: Freyellidae; genus: Freyastera; taxonRank: Genus; scientificNameAuthorship: Downey, 1986; **Location:** waterBody: Indian Ocean; stateProvince: Central Indian Ridge; locality: Edmond/ vent site 2; verbatimLocality: Cluster 4; maximumDepthInMeters: 3234; locationRemarks: FS Sonne Cruise INDEX2013 Leg 2; geodeticDatum: WGS84; coordinateUncertaintyInMeters: 31; **Identification:** identifiedBy: Christopher Mah; identificationRemarks: Identified only from imagery; identificationQualifier: gen. inc.; **Event:** eventDate: 2013-12-12; eventTime: 10:48:06 am; year: 2013; fieldNumber: INDEX2013-49ROV; **Record Level:** language: en; institutionCode: DZMB; datasetName: INDEX; basisOfRecord: Human Observation

##### Notes

Fig. 146

#### Freyella

Perrier, 1885

EE15FEAB-FC2A-5D18-902D-BA3241B1D8F0

#### Freyella gen. inc.

F6494624-5F3F-5401-B11C-067C26AA3B47

##### Materials

- **Type status:** Other material. **Occurrence:** recordedBy: ROPOS.COM; individualCount: 1; lifeStage: Adult; behavior: on seafloor; occurrenceStatus: present; preparations: Imaged only; associatedMedia: R2107_00109.jpg; **Taxon:** taxonConceptID: Freyella gen. inc.; kingdom: Animalia; phylum: Echinodermata; class: Asteroidea; order: Brisingida; family: Freyellidae; genus: Freyella; taxonRank: Genus; scientificNameAuthorship: Perrier, 1885; **Location:** waterBody: Indian Ocean; stateProvince: Rodriguez Triple Junction; locality: Vent site 4; verbatimLocality: Cluster 5; maximumDepthInMeters: 2632; locationRemarks: RV Pelagia Cruise INDEX2018 Leg 2; geodeticDatum: WGS84; coordinateUncertaintyInMeters: 26; **Identification:** identifiedBy: Christopher Mah; identificationRemarks: Identified only from imagery; identificationQualifier: gen. inc.; **Event:** eventDate: 2018-12-11; eventTime: 8:21:51 am; year: 2018; fieldNumber: INDEX2018-99ROPOS; fieldNotes: 1.8°C, 34.7 ppt; **Record Level:** language: en; institutionCode: DZMB; datasetName: INDEX; basisOfRecord: Human Observation

##### Notes

Fig. 147

#### Paxillosida

Perrier, 1884

468B47A9-FB8E-58D1-AA0F-41BE941E4E56

#### Porcellanasteridae

Sladen, 1883

2D076DE8-BEF2-5AF2-936C-83BCCFAC766F

#### Styracaster

Sladen, 1883

5F1BAB92-5A9F-56CD-93D2-E5F01B3F8ED6

#### Styracaster gen. inc.

3BFD2A4D-6CE0-5ED1-9442-51C07A27191F

##### Materials

- **Type status:** Other material. **Occurrence:** recordedBy: BGR; individualCount: 1; lifeStage: Adult; behavior: on sediment; occurrenceStatus: present; preparations: Imaged only; associatedMedia: 17MFT Fotos 2013-321-2.jpg; **Taxon:** taxonConceptID: Styracaster gen. inc.; kingdom: Animalia; phylum: Echinodermata; class: Asteroidea; order: Paxillosida; family: Porcellanasteridae; genus: Styracaster; taxonRank: Genus; scientificNameAuthorship: Sladen, 1883; **Location:** waterBody: Indian Ocean; stateProvince: Central Indian Ridge; locality: MESO; verbatimLocality: outside INDEX claim; maximumDepthInMeters: 2840; locationRemarks: FS Sonne Cruise INDEX2013 Leg 1; decimalLatitude: -23.3860; decimalLongitude: 69.2390; geodeticDatum: WGS84; coordinateUncertaintyInMeters: 28; **Identification:** identifiedBy: Christopher Mah; identificationRemarks: Identified only from imagery; identificationQualifier: gen. inc.; **Event:** eventDate: 2013-11-25; eventTime: 4:58:30 am; year: 2013; fieldNumber: INDEX2013-17MFT; fieldNotes: 1.8°C, 34.7 ppt; **Record Level:** language: en; institutionCode: DZMB; datasetName: INDEX; basisOfRecord: Human Observation

##### Notes

Fig. 148

#### Spinulosida

Perrier, 1884

FBC782DD-CD29-5C1D-9053-8A56A322CA4D

#### Echinasteridae

Verrill, 1867

CE6F1FBD-253D-58A5-AB0C-BB7A7C0380F9

#### Henricia

Gray, 1840

953992C1-ED76-54A7-82A9-A1494E790FF1

#### Henricia gen. inc.

CCB53BB1-AFA3-5C45-985A-149BDD51CA01

##### Materials

- **Type status:** Other material. **Occurrence:** recordedBy: ROPOS.COM; individualCount: 1; lifeStage: Adult; behavior: on basalt; occurrenceStatus: present; preparations: Imaged only; associatedMedia: R2107_00042.jpg; **Taxon:** taxonConceptID: Henricia gen. inc.; kingdom: Animalia; phylum: Echinodermata; class: Asteroidea; order: Spinulosida; family: Echinasteridae; genus: Henricia; taxonRank: Genus; scientificNameAuthorship: Gray, 1840; **Location:** waterBody: Indian Ocean; stateProvince: Rodriguez Triple Junction; locality: Vent site 4; verbatimLocality: Cluster 5; maximumDepthInMeters: 2653; locationRemarks: RV Pelagia Cruise INDEX2018 Leg 2; geodeticDatum: WGS84; coordinateUncertaintyInMeters: 26; **Identification:** identifiedBy: Christopher Mah; identificationRemarks: Identified only from imagery; identificationQualifier: gen. inc.; **Event:** eventDate: 2018-12-11; eventTime: 4:39:08 am; year: 2018; fieldNumber: INDEX2018-99ROPOS; fieldNotes: 1.8°C, 34.7 ppt; **Record Level:** language: en; institutionCode: DZMB; datasetName: INDEX; basisOfRecord: Human Observation

##### Notes

Fig. 149

#### Valvatida

Perrier, 1884

E512424C-600F-5987-AEB4-8E8C16BEBCED

#### Goniasteridae

Forbes, 1841

866010C5-6973-57C2-AF7E-82D25862949A

#### Goniasteridae gen. indet. (DZMB_2021_0066)

**Figure 2.**
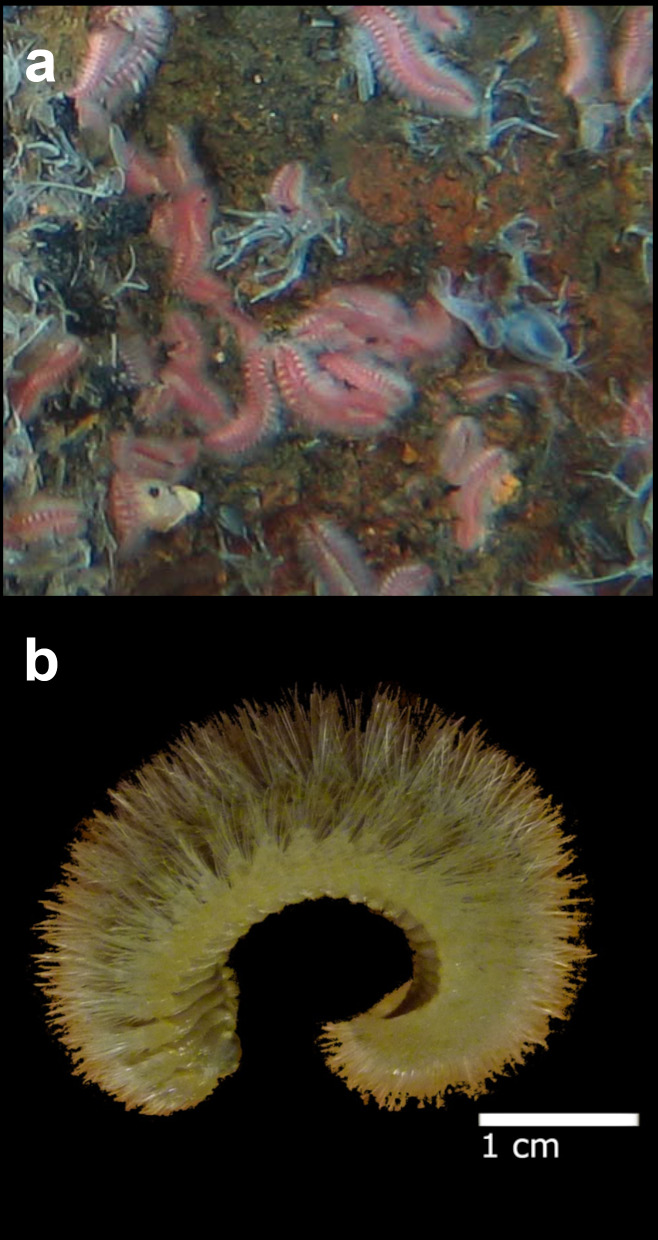
*Archinomejasoni* in situ (a) and sampled specimen (b) within the Kairei hydrothermal vent field in Cluster 5 of the INDEX area. Image corresponds with the data (Image attribution: BGR and GEOMAR).

8D7ABB2D-2724-5FF5-9C13-FC4141B49DC7

##### Materials

- **Type status:** Other material. **Occurrence:** recordedBy: ROPOS.COM; individualCount: 1; lifeStage: Adult; behavior: on hard substrates; occurrenceStatus: present; preparations: Imaged only; associatedMedia: R2095_00067.jpg; **Taxon:** taxonConceptID: Goniasteridae gen. indet. (DZMB_2021_0066); kingdom: Animalia; phylum: Echinodermata; class: Asteroidea; order: Valvatida; family: Goniasteridae; taxonRank: Family; scientificNameAuthorship: Forbes, 1841; **Location:** waterBody: Indian Ocean; stateProvince: South East Indian Ridge; locality: Vent site 6; verbatimLocality: Cluster 12; maximumDepthInMeters: 2355; locationRemarks: RV Pelagia Cruise INDEX2018 Leg 2; geodeticDatum: WGS84; coordinateUncertaintyInMeters: 24; **Identification:** identifiedBy: Christopher Mah; identificationRemarks: Identified only from imagery; identificationQualifier: gen. indet.; **Event:** eventDate: 2018-11-23; eventTime: 6:24:30 am; year: 2018; fieldNumber: INDEX2018-63ROPOS; **Record Level:** language: en; institutionCode: DZMB; datasetName: INDEX; basisOfRecord: Human Observation

##### Notes

Fig. 150

#### Goniasteridae gen. indet. (DZMB_2021_0067)

6E923E12-A631-5BF9-8CA4-C4E515590C7E

##### Materials

- **Type status:** Other material. **Occurrence:** recordedBy: BGR; individualCount: 1; lifeStage: Adult; behavior: on sediment; occurrenceStatus: present; preparations: Imaged only; associatedMedia: 44MFT Fotos 2013-447_Circeaster MAYBE.jpg; **Taxon:** taxonConceptID: Goniasteridae gen. indet. (DZMB_2021_0067); kingdom: Animalia; phylum: Echinodermata; class: Asteroidea; order: Valvatida; family: Goniasteridae; taxonRank: Family; scientificNameAuthorship: Forbes, 1841; **Location:** waterBody: Indian Ocean; stateProvince: Central Indian Ridge; locality: Edmond; verbatimLocality: Cluster 4; maximumDepthInMeters: 3328; locationRemarks: FS Sonne Cruise INDEX2013 Leg 2; decimalLatitude: -23.8791; decimalLongitude: 69.6003; geodeticDatum: WGS84; coordinateUncertaintyInMeters: 33; **Identification:** identifiedBy: Christopher Mah; identificationRemarks: Identified only from imagery; identificationQualifier: gen. indet.; **Event:** eventDate: 2013-12-10; eventTime: 12:16:39 am; year: 2013; fieldNumber: INDEX2013-44MFT; fieldNotes: 1.8°C, 34.7 ppt; **Record Level:** language: en; institutionCode: DZMB; datasetName: INDEX; basisOfRecord: Human Observation

##### Notes

Fig. 151

#### Circeaster

Koehler, 1909

C949CBA8-C776-5E62-A13D-8CB1C769F769

#### Circeaster gen. inc.

5833610A-D6DD-5137-B221-31A2CE56D68C

##### Materials

- **Type status:** Other material. **Occurrence:** recordedBy: ROPOS.COM; individualCount: 1; lifeStage: Adult; behavior: on hard substrates; occurrenceStatus: present; preparations: Imaged only; associatedMedia: R2094_01142.jpg; **Taxon:** taxonConceptID: Circeaster gen. inc.; kingdom: Animalia; phylum: Echinodermata; class: Asteroidea; order: Valvatida; family: Goniasteridae; genus: Circeaster; taxonRank: Genus; scientificNameAuthorship: Koehler, 1909; **Location:** waterBody: Indian Ocean; stateProvince: South East Indian Ridge; locality: Vent site 6; verbatimLocality: Cluster 12; maximumDepthInMeters: 2473; locationRemarks: RV Pelagia Cruise INDEX2018 Leg 2; geodeticDatum: WGS84; coordinateUncertaintyInMeters: 25; **Identification:** identifiedBy: Christopher Mah; identificationRemarks: Identified only from imagery; identificationQualifier: gen. inc.; **Event:** eventDate: 2018-11-22; eventTime: 10:33:44 am; year: 2018; fieldNumber: INDEX2018-61ROPOS; fieldNotes: 1.9°C, 34.7 ppt; **Record Level:** language: en; institutionCode: DZMB; datasetName: INDEX; basisOfRecord: Human Observation

##### Notes

Fig. 152

#### Evoplosoma

Fisher, 1906

1032254F-C8C9-5F1B-8F86-3D403CD9EC0E

#### Evoplosoma gen. inc.

5E26B49E-3661-594C-B85B-33923D0CAD51

##### Materials

- **Type status:** Other material. **Occurrence:** recordedBy: ROPOS.COM; individualCount: 1; lifeStage: Adult; behavior: Attached to coral stalk; occurrenceStatus: present; preparations: Imaged only; associatedMedia: R2092_00603.jpg; associatedOccurrences: Alcyonacea fam. indet.; **Taxon:** taxonConceptID: Evoplosoma gen. inc.; kingdom: Animalia; phylum: Echinodermata; class: Asteroidea; order: Valvatida; family: Goniasteridae; genus: Evoplosoma; taxonRank: Genus; scientificNameAuthorship: Fisher, 1906; **Location:** waterBody: Indian Ocean; stateProvince: South East Indian Ridge; locality: Vent site 6; verbatimLocality: Cluster 12; maximumDepthInMeters: 2465; locationRemarks: RV Pelagia Cruise INDEX2018 Leg 2; geodeticDatum: WGS84; coordinateUncertaintyInMeters: 25; **Identification:** identifiedBy: Christopher Mah; identificationRemarks: Identified only from imagery; identificationQualifier: gen. inc.; **Event:** eventDate: 2018-11-20; eventTime: 12:21:14 pm; year: 2018; fieldNumber: INDEX2018-57ROPOS; fieldNotes: 1.8°C, 34.7 ppt; **Record Level:** language: en; institutionCode: DZMB; datasetName: INDEX; basisOfRecord: Human Observation

##### Notes

Fig. 153

#### Lydiaster

Koehler, 1909

FA1F974A-7429-500B-AA5B-099615D3AB99

#### Lydiaster johannae sp. inc.

Koehler, 1909

C7253ED0-6ECD-57F8-8A6D-805303A83E02

##### Materials

- **Type status:** Other material. **Occurrence:** recordedBy: BGR/ GEOMAR; individualCount: 1; lifeStage: Adult; behavior: on sediment; occurrenceStatus: present; preparations: Imaged only; associatedMedia: 2013_INDEX2013_049ROV06.jpg; associatedOccurrences: Polynoidae fam. inc.; **Taxon:** taxonConceptID: Lydiasterjohannae sp. inc.; scientificName: Lydiasterjohannae; kingdom: Animalia; phylum: Echinodermata; class: Asteroidea; order: Valvatida; family: Goniasteridae; genus: Lydiaster; taxonRank: Species; scientificNameAuthorship: Koehler, 1909; **Location:** waterBody: Indian Ocean; stateProvince: Central Indian Ridge; locality: Edmond/ vent site 2; verbatimLocality: Cluster 4; maximumDepthInMeters: 3240; locationRemarks: FS Sonne Cruise INDEX2013 Leg 2; geodeticDatum: WGS84; coordinateUncertaintyInMeters: 31; **Identification:** identifiedBy: Christopher Mah; identificationRemarks: Identified only from imagery; identificationQualifier: sp. inc.; **Event:** eventDate: 2013-12-12; eventTime: 10:39:59 am; year: 2013; fieldNumber: INDEX2013-49ROV; **Record Level:** language: en; institutionCode: DZMB; datasetName: INDEX; basisOfRecord: Human Observation

##### Notes

Fig. 154

#### Solasteridae

Viguier, 1878

0621EB30-4C11-57C5-9838-0C547608A345

#### Solasteridae fam. inc.

AE9A8543-A793-5CC4-906A-F3422EEF8616

##### Materials

- **Type status:** Other material. **Occurrence:** recordedBy: NIOZ; individualCount: 1; lifeStage: Adult; behavior: on hard substrates; occurrenceStatus: present; preparations: Imaged only; associatedMedia: 20141117090809540.jpg; **Taxon:** taxonConceptID: Solasteridae fam. inc.; kingdom: Animalia; phylum: Echinodermata; class: Asteroidea; order: Valvatida; family: Solasteridae; taxonRank: Family; scientificNameAuthorship: Viguier, 1878; **Location:** waterBody: Indian Ocean; stateProvince: South East Indian Ridge; locality: Pelagia; verbatimLocality: Cluster 8; maximumDepthInMeters: 3465; locationRemarks: RV Pelagia Cruise INDEX2014 Leg 1; geodeticDatum: WGS84; coordinateUncertaintyInMeters: 35; **Identification:** identifiedBy: Christopher Mah; identificationRemarks: Identified only from imagery; identificationQualifier: fam. inc.; **Event:** eventDate: 2014-11-17; eventTime: 9:08:09 am; year: 2014; fieldNumber: INDEX2014-28VS; fieldNotes: 1.7°C; **Record Level:** language: en; institutionCode: DZMB; datasetName: INDEX; basisOfRecord: Human Observation

##### Notes

Fig. 155

#### Velatida

Perrier, 1884

BA9E659A-A0B4-5B1C-831A-3C1C0C6DA120

#### Myxasteridae

Perrier, 1885

296A1B0A-85DA-5443-B545-95631D4323FB

#### Asthenactis

Fisher, 1906

913F152C-8D6D-5DDA-BAE7-60EC59814734

#### Asthenactis gen. inc.

1593E85C-8F74-5468-9588-54CCF437A80B

##### Materials

- **Type status:** Other material. **Occurrence:** recordedBy: NIOZ; individualCount: 1; lifeStage: Adult; behavior: on seafloor; occurrenceStatus: present; preparations: Imaged only; associatedMedia: 20141128145057005.jpg; **Taxon:** taxonConceptID: Asthenactis gen. inc.; kingdom: Animalia; phylum: Echinodermata; class: Asteroidea; order: Velatida; family: Myxasteridae; genus: Asthenactis; taxonRank: Genus; scientificNameAuthorship: Fisher, 1906; **Location:** waterBody: Indian Ocean; stateProvince: South East Indian Ridge; locality: SEIR; verbatimLocality: Cluster 9; maximumDepthInMeters: 3472; locationRemarks: RV Pelagia Cruise INDEX2014 Leg 1; geodeticDatum: WGS84; coordinateUncertaintyInMeters: 36; **Identification:** identifiedBy: Christopher Mah; identificationRemarks: Identified only from imagery; identificationQualifier: gen. inc.; **Event:** eventDate: 2014-11-28; eventTime: 2:50:57 pm; year: 2014; fieldNumber: INDEX2014-44VS; fieldNotes: 1.7°C; **Record Level:** language: en; institutionCode: DZMB; datasetName: INDEX; basisOfRecord: Human Observation

##### Notes

Fig. 156

#### Pterasteridae

Perrier, 1875

C4F93925-B303-5BA9-9F2A-22101F1ABCF6

#### Hymenaster

Wyville Thomson, 1873

72D4EC46-1C62-5652-90E0-994B7DFA6FE7

#### Hymenaster sp. indet.

13FE177F-55F1-5C0B-8171-D41B966D96C2

##### Materials

- **Type status:** Other material. **Occurrence:** recordedBy: NIOZ; individualCount: 1; lifeStage: Adult; behavior: on hard substrates; occurrenceStatus: present; preparations: Imaged only; associatedMedia: 20141203172801863.jpg; **Taxon:** taxonConceptID: Hymenaster sp. indet.; kingdom: Animalia; phylum: Echinodermata; class: Asteroidea; order: Velatida; family: Pterasteridae; genus: Hymenaster; taxonRank: Genus; scientificNameAuthorship: Wyville Thomson, 1873; **Location:** waterBody: Indian Ocean; stateProvince: South East Indian Ridge; locality: SEIR; verbatimLocality: Cluster 6; maximumDepthInMeters: 3588; locationRemarks: RV Pelagia Cruise INDEX2014 Leg 1; geodeticDatum: WGS84; coordinateUncertaintyInMeters: 38; **Identification:** identifiedBy: Christopher Mah; identificationRemarks: Identified only from imagery; identificationQualifier: sp. indet.; **Event:** eventDate: 2014-12-03; eventTime: 5:28:01 pm; year: 2014; fieldNumber: INDEX2014-54VS; fieldNotes: 1.7°C; **Record Level:** language: en; institutionCode: DZMB; datasetName: INDEX; basisOfRecord: Human Observation

##### Notes

Fig. 157

#### Pteraster

Müller & Troschel, 1842

3B890C11-E840-59A6-82F3-16329E4F4989

#### Pteraster gen. inc.

8CFB8FEB-C8BF-520A-BC6C-B2155BBA9B37

##### Materials

- **Type status:** Other material. **Occurrence:** recordedBy: BGR; individualCount: 1; lifeStage: Adult; behavior: on basalt; occurrenceStatus: present; preparations: Imaged only; associatedMedia: IMG_4517.jpg; **Taxon:** taxonConceptID: Pteraster gen. inc.; kingdom: Animalia; phylum: Echinodermata; class: Asteroidea; order: Velatida; family: Pterasteridae; genus: Pteraster; taxonRank: Genus; scientificNameAuthorship: Müller & Troschel, 1842; **Location:** waterBody: Indian Ocean; stateProvince: South East Indian Ridge; locality: SEIR; verbatimLocality: Cluster 11; maximumDepthInMeters: 2892; locationRemarks: FS Sonne Cruise INDEX2017 Leg 1; decimalLatitude: -27.2563; decimalLongitude: 72.7241; geodeticDatum: WGS84; coordinateUncertaintyInMeters: 29; **Identification:** identifiedBy: Christopher Mah; identificationRemarks: Identified only from imagery; identificationQualifier: gen. inc.; **Event:** eventDate: 2017-09-24; eventTime: 3:25:09 pm; year: 2017; fieldNumber: INDEX2017-83STR; fieldNotes: 1.7°C, 34.7 ppt; **Record Level:** language: en; institutionCode: DZMB; datasetName: INDEX; basisOfRecord: Human Observation

##### Notes

Fig. 158

#### Crinoidea

Miller, 1821

44621C2E-3E47-5D67-B1EE-1310E9DB67EF

#### Comatulida

E1993FA4-9503-5CE1-BA9A-04BE4BE9661F

#### Antedonidae

Norman, 1865

E418FF02-C4AC-59A5-8F96-3429CB1C8D72

#### Antedonidae gen. indet. (DZMB_2021_0068)

8F97612B-3DFF-5FC4-BFDD-4FAC513E77E9

##### Materials

- **Type status:** Other material. **Occurrence:** recordedBy: ROPOS.COM; individualCount: 1; lifeStage: Adult; behavior: attached to basalt; occurrenceStatus: present; preparations: Imaged only; associatedMedia: R2101_00131.jpg; **Taxon:** taxonConceptID: Antedonidae gen. indet. (DZMB_2021_0068); kingdom: Animalia; phylum: Echinodermata; class: Crinoidea; order: Comatulida; family: Antedonidae; taxonRank: Family; scientificNameAuthorship: Norman, 1865; **Location:** waterBody: Indian Ocean; stateProvince: South East Indian Ridge; locality: Vent site 5; verbatimLocality: Cluster 11; maximumDepthInMeters: 2909; locationRemarks: RV Pelagia Cruise INDEX2018 Leg 2; geodeticDatum: WGS84; coordinateUncertaintyInMeters: 29; **Identification:** identifiedBy: Charles G. Messing; identificationRemarks: Identified only from imagery; identificationQualifier: gen. indet.; **Event:** eventDate: 2018-11-29; eventTime: 10:01:21 am; year: 2018; fieldNumber: INDEX2018-75ROPOS; fieldNotes: 1.7°C, 34.7 ppt; **Record Level:** language: en; institutionCode: DZMB; datasetName: INDEX; basisOfRecord: Human Observation

##### Notes

Fig. 159

#### Antedonidae fam. inc. (DZMB_2021_0069)

8CD0C83E-CE31-50E8-87F7-A0F638B8742C

##### Materials

- **Type status:** Other material. **Occurrence:** recordedBy: BGR/ GEOMAR; individualCount: 1; lifeStage: Adult; behavior: on basalt; occurrenceStatus: present; preparations: Imaged only; associatedMedia: 2013-12-14_11-52-16_Sonne_INDEX2013-2_055ROV08_Logo.jpg; **Taxon:** taxonConceptID: Antedonidae fam. inc. (DZMB_2021_0069); kingdom: Animalia; phylum: Echinodermata; class: Crinoidea; order: Comatulida; family: Antedonidae; taxonRank: Family; scientificNameAuthorship: Norman, 1865; **Location:** waterBody: Indian Ocean; stateProvince: Central Indian Ridge; locality: Edmond/ Vent site 7; verbatimLocality: Cluster 4; maximumDepthInMeters: 3245; locationRemarks: FS Sonne Cruise INDEX2013 Leg 2; geodeticDatum: WGS84; coordinateUncertaintyInMeters: 32; **Identification:** identifiedBy: Charles G. Messing; identificationRemarks: Identified only from imagery; identificationQualifier: fam. inc.; **Event:** eventDate: 2013-12-14; eventTime: 11:52:16 am; year: 2013; fieldNumber: INDEX2013-55ROV; **Record Level:** language: en; institutionCode: DZMB; datasetName: INDEX; basisOfRecord: Human Observation

##### Notes

Fig. 160

#### Bathymetra

AH Clark, 1908

1BCE37F6-2472-54E2-906B-44DA80B22D4C

#### Bathymetra gen. inc.

3922C4E8-E890-59E6-8605-BC1C6DB66C5F

##### Materials

- **Type status:** Other material. **Occurrence:** recordedBy: IFREMER; lifeStage: Adult; behavior: attached to basalt; occurrenceStatus: present; preparations: Imaged only; associatedMedia: 160121215033072_01_1080i Kopie.jpg; **Taxon:** taxonConceptID: Bathymetra gen. inc.; kingdom: Animalia; phylum: Echinodermata; class: Crinoidea; order: Comatulida; family: Antedonidae; genus: Bathymetra; taxonRank: Genus; scientificNameAuthorship: AH Clark, 1908; **Location:** waterBody: Indian Ocean; stateProvince: South East Indian Ridge; locality: Pelagia; verbatimLocality: Cluster 8; maximumDepthInMeters: 3677; locationRemarks: RV Pourqoui pas? Cruise INDEX2016 Leg 1; geodeticDatum: WGS84; coordinateUncertaintyInMeters: 37; **Identification:** identifiedBy: Charles G. Messing; identificationRemarks: Identified only from imagery; identificationQualifier: gen. inc.; **Event:** eventDate: 2016-01-21; eventTime: 9:50:33 pm; year: 2016; fieldNumber: INDEX2016-20ROV; fieldNotes: 1.7°C, 34.7 ppt; **Record Level:** language: en; institutionCode: DZMB; datasetName: INDEX; basisOfRecord: Human Observation

##### Notes

Fig. 161

#### Pentametrocrinidae

AH Clark, 1908

2358AC0C-8A8B-543B-B547-E650805991DE

#### Pentametrocrinus

AH Clark, 1908

54B97FC7-CEFB-53DE-B716-559BD500C7BF

#### Pentametrocrinus sp. indet.

0F8C8B9E-575C-5A9C-84A2-664CCA12F7B9

##### Materials

- **Type status:** Other material. **Occurrence:** recordedBy: BGR; individualCount: 1; lifeStage: Adult; behavior: attached to basalt; occurrenceStatus: present; preparations: Imaged only; associatedMedia: 17MFT Fotos 2013-314-3_Probably CRINOID.jpg; **Taxon:** taxonConceptID: Pentametrocrinus sp. indet.; kingdom: Animalia; phylum: Echinodermata; class: Crinoidea; order: Comatulida; family: Pentametrocrinidae; genus: Pentametrocrinus; taxonRank: Genus; scientificNameAuthorship: AH Clark, 1908; **Location:** waterBody: Indian Ocean; stateProvince: Central Indian Ridge; locality: MESO; verbatimLocality: outside INDEX claim; maximumDepthInMeters: 2827; locationRemarks: FS Sonne Cruise INDEX2013 Leg 1; decimalLatitude: -23.3924; decimalLongitude: 69.2425; geodeticDatum: WGS84; coordinateUncertaintyInMeters: 28; **Identification:** identifiedBy: Charles G. Messing; identificationRemarks: Identified only from imagery; identificationQualifier: sp. indet.; **Event:** eventDate: 2013-11-25; eventTime: 3:17:19 am; year: 2013; fieldNumber: INDEX2013-17MFT; fieldNotes: 1.8°C, 34.7 ppt; **Record Level:** language: en; institutionCode: DZMB; datasetName: INDEX; basisOfRecord: Human Observation

##### Notes

Fig. 162

#### Hyocrinida

Rasmussen, 1978

AAA3B948-462D-5ECE-9126-35D822E3E6D1

#### Hyocrinidae

Carpenter, 1884

86C40F67-D4F7-578C-8273-9A205E0D83B5

#### Hyocrinidae gen. indet.

6C788253-F7E1-547C-962E-8FC06D3394A9

##### Materials

- **Type status:** Other material. **Occurrence:** recordedBy: IFREMER; individualCount: 1; lifeStage: likely juvenile; behavior: attached to basalt; occurrenceStatus: present; preparations: Imaged only; associatedMedia: 160122005356857_16_1080i Kopie.jpg; **Taxon:** taxonConceptID: Hyocrinidae gen. indet.; kingdom: Animalia; phylum: Echinodermata; class: Crinoidea; order: Hyocrinida; family: Hyocrinidae; taxonRank: Family; scientificNameAuthorship: Carpenter, 1884; **Location:** waterBody: Indian Ocean; stateProvince: South East Indian Ridge; locality: Pelagia; verbatimLocality: Cluster 8; maximumDepthInMeters: 3676; locationRemarks: RV Pourqoui pas? Cruise INDEX2016 Leg 1; geodeticDatum: WGS84; coordinateUncertaintyInMeters: 37; **Identification:** identifiedBy: Charles G. Messing; identificationRemarks: Identified only from imagery; identificationQualifier: gen. indet.; **Event:** eventDate: 2016-01-22; eventTime: 12:53:56 am; year: 2016; fieldNumber: INDEX2016-20ROV; fieldNotes: 1.7°C, 34.7 ppt; **Record Level:** language: en; institutionCode: DZMB; datasetName: INDEX; basisOfRecord: Human Observation

##### Notes

Fig. 163

#### Echinoidea

Leske, 1778

98242E0B-9EF7-5FB3-A7C5-0D52DA046F55

#### Infraclass Irregularia

Latreille, 1825

BD1148C3-EFEF-5C90-AD8B-C80D4A9A0190

#### Irregularia

infracl. inc.

1F9BC229-4CCC-5431-98AA-F1EA502A7794

##### Materials

- **Type status:** Other material. **Occurrence:** recordedBy: NIOZ; individualCount: 1; lifeStage: Adult; behavior: on sediment; occurrenceStatus: present; preparations: Imaged only; associatedMedia: 20141128124848010.jpg; **Taxon:** taxonConceptID: Irregularia infracl. inc.; kingdom: Animalia; phylum: Echinodermata; class: Echinoidea; taxonRank: Infraclass; scientificNameAuthorship: Latreille, 1825; **Location:** waterBody: Indian Ocean; stateProvince: South East Indian Ridge; locality: SEIR; verbatimLocality: Cluster 9; maximumDepthInMeters: 3354; locationRemarks: RV Pelagia Cruise INDEX2014 Leg 1; geodeticDatum: WGS84; coordinateUncertaintyInMeters: 36; **Identification:** identifiedBy: Andreas Kroh; identificationRemarks: Identified only from imagery; identificationQualifier: infracl. inc.; **Event:** eventDate: 2014-11-28; eventTime: 12:48:48 pm; year: 2014; fieldNumber: INDEX2014-44VS; fieldNotes: 1.7°C; **Record Level:** language: en; institutionCode: DZMB; datasetName: INDEX; basisOfRecord: Human Observation

##### Notes

Remarks: The animal seen in Fig. 164 appears to be an irregular sea urchin, based on the overall shape and darker radial regions possibly representing ambulacra. It could belong to a number of different groups, including holasteroids, spatangoids and cassiduloids. The blurred nature of the image renders a more refined identification impossible.

#### Cidaroida

Claus, 1880

43CC205D-C43B-52EA-9E9D-28CE35ACEE4A

#### Cidaroida fam. indet.

4FF15BDA-E400-55F3-91ED-D17D1DFA0F4D

##### Materials

- **Type status:** Other material. **Occurrence:** recordedBy: ROPOS.COM; individualCount: 1; lifeStage: Adult; behavior: on sediment; occurrenceStatus: present; preparations: Imaged only; associatedMedia: R2104_00106.jpg; **Taxon:** taxonConceptID: Cidaroida fam. indet.; kingdom: Animalia; phylum: Echinodermata; class: Echinoidea; order: Cidaroida; taxonRank: Order; scientificNameAuthorship: Claus, 1880; **Location:** waterBody: Indian Ocean; stateProvince: Rodriguez Triple Junction; locality: Vent site 4; verbatimLocality: Cluster 5; maximumDepthInMeters: 2508; locationRemarks: RV Pelagia Cruise INDEX2018 Leg 2; geodeticDatum: WGS84; coordinateUncertaintyInMeters: 25; **Identification:** identifiedBy: Andreas Kroh; identificationRemarks: Identified only from imagery; identificationQualifier: fam. indet.; **Event:** eventDate: 2018-12-04; eventTime: 8:09:09 am; year: 2018; fieldNumber: INDEX2018-85ROPOS; fieldNotes: 1.8°C, 34.7 ppt; **Record Level:** language: en; institutionCode: DZMB; datasetName: INDEX; basisOfRecord: Human Observation

##### Notes

Fig. 165

#### Echinothurioida

Claus, 1880

88569EF9-9D10-5C60-BF54-35C7B92017A4

#### Echinothuriidae

Thomson, 1872

D2214C41-ABC2-582B-A94B-43E5ADCE7231

#### Hapalosoma

Mortensen, 1903

1356444D-5D12-5FB5-BAD7-B629CED214F7

#### Hapalosoma sp. indet.

FCA96FEE-5EE9-5FC4-B25B-61C475AA8302

##### Materials

- **Type status:** Other material. **Occurrence:** recordedBy: BGR/ GEOMAR; individualCount: 1; lifeStage: Adult; behavior: on sediment; occurrenceStatus: present; preparations: DNA voucher and animal stored in 96% ethanol; associatedMedia: 2013-12-14_10-56-53_Sonne_INDEX2013-2_055ROV08_Logo.jpg; associatedOccurrences: none; associatedSequences: COI; **Taxon:** taxonID: I13_379; scientificNameID: Sperosoma biseriatum; taxonConceptID: Hapalosoma sp. indet.; kingdom: Animalia; phylum: Echinodermata; class: Echinoidea; order: Echinothurioida; family: Echinothuriidae; genus: Hapalosoma; taxonRank: Genus; scientificNameAuthorship: Mortensen, 1903; **Location:** waterBody: Indian Ocean; stateProvince: Central Indian Ridge; locality: Edmond/ Vent site 7; verbatimLocality: Cluster 4; maximumDepthInMeters: 3286; locationRemarks: FS Sonne Cruise INDEX2013 Leg 2; decimalLatitude: -23.8780; decimalLongitude: 69.6014; geodeticDatum: WGS84; coordinateUncertaintyInMeters: 32; **Identification:** identifiedBy: Andreas Kroh; identificationRemarks: Identified by morphology and DNA of collected specimen; identificationQualifier: sp. indet.; **Event:** eventDate: 2013-12-14; eventTime: 10:56:53 am; year: 2013; fieldNumber: INDEX2013-55ROV; **Record Level:** language: en; institutionCode: DZMB; collectionCode: I13_55RO_BB_1; datasetName: INDEX; basisOfRecord: Human Observation

##### Notes

Remarks: The echinoid seen in Fig. 166 clearly is a member of the subfamily Echinothuriinae, based on the presence of primary spines with their characteristic terminal hoofs. Identification to genus and species level is difficult in echinothurioids, specifically when based on images, since many forms are largely differentiated by details of their plate arrangement and pedicellarial morphology. In the present case, however, some information on the pedicellariae can be gained from the images. The specimen possesses very large tridentate pedicellariae with rounded blades which broaden towards the tip from a narrow base, a feature only known from species of the genus *Hapalosoma* (see Mortensen 1935, Anderson 2013). Four species of this genus are known (Anderson 2013), but none of them occurring in the Indian Ocean (having been reported from the New Zealand region, Malay Archipelago and Sagami Sea). That fact, combined with the divergent colouration of the observed specimen, suggests that it might belong to a new, yet undescribed species of *Hapalosoma*.

Less detail can be recognszed in an individual seen in images R1915_00209 and R1915_00213, but the high similarity to the specimen in Fig. 166 suggests that this might be a second representative of this putative new species.

#### Salenioida

Delage & Herouard, 1903

3F93EE25-8EFC-5FCC-9E9A-97D4CD214CBD

#### Saleniidae

Agassiz, 1838

A7C36197-E718-534C-8D77-D02095659419

#### Salenocidaris

Agassiz, 1869

0DFBD0E4-263C-5A78-A4D7-A037EA3A2DE7

#### Salenocidaris sp. indet.

6A19FC49-ABCB-5665-A6B2-B0FA4FC879A7

##### Materials

- **Type status:** Other material. **Occurrence:** recordedBy: ROPOS.COM; individualCount: 1; lifeStage: Adult; behavior: on sediment; occurrenceStatus: present; preparations: Imaged only; associatedMedia: R2095_00122.jpg; **Taxon:** taxonConceptID: Salenocidaris sp. indet.; kingdom: Animalia; phylum: Echinodermata; class: Echinoidea; order: Salenioida; family: Saleniidae; genus: Salenocidaris; taxonRank: Genus; scientificNameAuthorship: Agassiz, 1869; **Location:** waterBody: Indian Ocean; stateProvince: South East Indian Ridge; locality: Vent site 6; verbatimLocality: Cluster 12; maximumDepthInMeters: 2387; locationRemarks: RV Pelagia Cruise INDEX2018 Leg 2; geodeticDatum: WGS84; coordinateUncertaintyInMeters: 24; **Identification:** identifiedBy: Andreas Kroh; identificationRemarks: Identified only from imagery; identificationQualifier: sp. indet.; **Event:** eventDate: 2018-11-23; eventTime: 8:11:50 am; year: 2018; fieldNumber: INDEX2018-63ROPOS; **Record Level:** language: en; institutionCode: DZMB; datasetName: INDEX; basisOfRecord: Human Observation

##### Notes

Remarks: The presence of relatively few, long and slender spines with upturned distal ends characterise the observed echinoids as saleniids. This is further supported by the excentric position of the periproct and large apical disc seen in Fig. 167. Of extant saleniids, three genera are known, two of these (*Salenia* and *Bathysalenia*) are characterised by distinctly banded spines (with the exception of *Saleniaunicolor* Mortensen 1934 from the Sagami Sea and the Celebes Sea) and can be excluded. *Saleniaunicolor* is reported to have greenish-white primary spines and a greyish-purple test (Mortensen 1935), unlike the observed animals which have white spines and a white to light purple test. The observed specimens are thus assigned to the genus *Salenocidaris*. Amongst *Salenocidaris*, the most likely candidate seems to be *S.hastigera* (Agassiz 1879), which fits in terms of colouration and has been reported from the Malay Archipelago and the Indian Ocean (Mortensen 1935). The latter were assigned to a separate variety (now considered a subspecies) *S.hastigeraacuminata*, based on the long and pointed ambulacral spines (Mortensen 1934) – a feature which cannot be evaluated in the *in-situ* images available. A second possible candidate species is *Salenocidarisincrassata* Mortensen, 1934 described from the Celebes Sea. It is characterised by non-contiguous areoles of the primary interambulacral tubercles and distally thickened secondary spines – again features not visible in the available imagery. All other *Salenocidaris* species are either occurring in different oceans or are characterised by violet to dark purple tests and can, therefore, easily be excluded.

#### Holothuroidea

70A2B885-4F20-57BA-8BD2-6D37C57F2BEB

#### Apodida

Brandt, 1835

A65CD8D7-0438-5D43-9118-C8F40FE454A7

#### Chiridotidae

Östergren, 1898

26A1E6CC-A625-5DD4-B305-C26EBE3B4986

#### Chiridota

Eschscholtz, 1829

4BFEB766-02E2-5215-AA67-733EA7316CB8

#### Chiridota hydrothermica sp. inc.

Smirnov & Gebruk, 2000

35C070BA-A7DE-55A7-96E8-AA480D060C2B

##### Materials

- **Type status:** Other material. **Occurrence:** recordedBy: BGR/ GEOMAR; individualCount: 1; lifeStage: Adult; behavior: on sulphides; occurrenceStatus: present; preparations: DNA voucher and animal stored in 96% ethanol; associatedMedia: 2013-12-12_06-23-30_Sonne_INDEX2013-2_049ROV06_Logo.jpg; associatedOccurrences: none; associatedSequences: COI; **Taxon:** taxonID: I13_380; scientificNameID: Chiridota sp. 1; taxonConceptID: Chiridotahydrothermica sp. inc.; scientificName: Chiridotahydrothermica; kingdom: Animalia; phylum: Echinodermata; class: Holothuroidea; order: Apodida; family: Chiridotidae; genus: Chiridota; taxonRank: Species; scientificNameAuthorship: Smirnov & Gebruk, 2000; **Location:** waterBody: Indian Ocean; stateProvince: Central Indian Ridge; locality: Edmond; verbatimLocality: Cluster 4; maximumDepthInMeters: 3281; locationRemarks: FS Sonne Cruise INDEX2013 Leg 2; decimalLatitude: -23.8767; decimalLongitude: 69.5964; geodeticDatum: WGS84; coordinateUncertaintyInMeters: 31; **Identification:** identifiedBy: Andrey Gebruk, Antonina Kremenetskaia; identificationRemarks: Identified by morphology and DNA of collected specimen; identificationQualifier: sp. inc.; **Event:** eventDate: 2013-12-12; eventTime: 6:23:30 am; year: 2013; fieldNumber: INDEX2013-49ROV; **Record Level:** language: en; institutionCode: DZMB; collectionCode: I13_49RO_SG2_2; datasetName: INDEX; basisOfRecord: Human Observation

##### Notes

Fig. 168

#### Elasipodida

Théel, 1882

C038886D-B9D7-5E27-B14B-4B5941471E97

#### Elpidiidae

Théel, 1882

C434ED54-7797-5355-B02C-D008EAD74A58

#### Elpidiidae gen. indet. (DZMB_2021_0070)

9F68A11F-1ABE-5207-93ED-C0D4EA5BDC42

##### Materials

- **Type status:** Other material. **Occurrence:** recordedBy: BGR; individualCount: 1; lifeStage: Adult; behavior: on basalt; occurrenceStatus: present; preparations: Imaged only; associatedMedia: 17MFT Fotos 2013-289-7.jpg; **Taxon:** taxonConceptID: Elpidiidae gen. indet. (DZMB_2021_0070); kingdom: Animalia; phylum: Echinodermata; class: Holothuroidea; order: Elasipodida; family: Elpidiidae; taxonRank: Family; scientificNameAuthorship: Théel, 1882; **Location:** waterBody: Indian Ocean; stateProvince: Central Indian Ridge; locality: MESO; verbatimLocality: outside INDEX claim; maximumDepthInMeters: 2820; locationRemarks: FS Sonne Cruise INDEX2013 Leg 1; decimalLatitude: -23.3919; decimalLongitude: 69.2420; geodeticDatum: WGS84; coordinateUncertaintyInMeters: 28; **Identification:** identifiedBy: Andrey Gebruk, Antonina Kremenetskaia; identificationRemarks: Identified only from imagery; identificationQualifier: gen. indet.; **Event:** eventDate: 2013-11-25; eventTime: 3:27:11 am; year: 2013; fieldNumber: INDEX2013-17MFT; fieldNotes: 1.8°C, 34.7 ppt; **Record Level:** language: en; institutionCode: DZMB; datasetName: INDEX; basisOfRecord: Human Observation

##### Notes

Fig. 169

#### Elpidiidae gen. indet. (DZMB_2021_0071)

878E945D-1A48-5002-8457-5D334C524ACB

##### Materials

- **Type status:** Other material. **Occurrence:** recordedBy: ROPOS.COM; individualCount: 1; lifeStage: Adult; behavior: on sediment; occurrenceStatus: present; preparations: Imaged only; associatedMedia: R2105_00055.jpg; **Taxon:** taxonConceptID: Elpidiidae gen. indet. (DZMB_2021_0071); kingdom: Animalia; phylum: Echinodermata; class: Holothuroidea; order: Elasipodida; family: Elpidiidae; taxonRank: Family; scientificNameAuthorship: Théel, 1882; **Location:** waterBody: Indian Ocean; stateProvince: Rodriguez Triple Junction; locality: Vent site 4; verbatimLocality: Cluster 5; maximumDepthInMeters: 2604; locationRemarks: RV Pelagia Cruise INDEX2018 Leg 2; geodeticDatum: WGS84; coordinateUncertaintyInMeters: 26; **Identification:** identifiedBy: Andrey Gebruk, Antonina Kremenetskaia; identificationRemarks: Identified only from imagery; identificationQualifier: gen. indet.; **Event:** eventDate: 2018-12-09; eventTime: 6:06:13 am; year: 2018; fieldNumber: INDEX2018-95ROPOS; fieldNotes: 1.8°C, 34.7 ppt; **Record Level:** language: en; institutionCode: DZMB; datasetName: INDEX; basisOfRecord: Human Observation

##### Notes

Fig. 170

#### Elpidiidae gen. indet. (DZMB_2021_0072)

48768CE6-8F42-50A0-BBCB-0685CD8582AA

##### Materials

- **Type status:** Other material. **Occurrence:** recordedBy: NIOZ; individualCount: 1; lifeStage: Adult; behavior: on seafloor; occurrenceStatus: present; preparations: Imaged only; associatedMedia: 20141128112903004.jpg; **Taxon:** taxonConceptID: Elpidiidae gen. indet. (DZMB_2021_0072); kingdom: Animalia; phylum: Echinodermata; class: Holothuroidea; order: Elasipodida; family: Elpidiidae; taxonRank: Family; scientificNameAuthorship: Théel, 1882; **Location:** waterBody: Indian Ocean; stateProvince: South East Indian Ridge; locality: SEIR; verbatimLocality: Cluster 9; maximumDepthInMeters: 3345; locationRemarks: RV Pelagia Cruise INDEX2014 Leg 1; geodeticDatum: WGS84; coordinateUncertaintyInMeters: 36; **Identification:** identifiedBy: Andrey Gebruk, Antonina Kremenetskaia; identificationRemarks: Identified only from imagery; identificationQualifier: gen. indet.; **Event:** eventDate: 2014-11-28; eventTime: 11:29:03 am; year: 2014; fieldNumber: INDEX2014-44VS; fieldNotes: 1.7°C; **Record Level:** language: en; institutionCode: DZMB; datasetName: INDEX; basisOfRecord: Human Observation

##### Notes

Fig. 171

#### Peniagone

Théel, 1882

56829685-32EA-567A-9F75-3488E816B43D

#### Peniagone purpurea

(Théel, 1882)

6BA7E2CE-6501-5237-8463-E6E63C9EB3D7

##### Materials

- **Type status:** Other material. **Occurrence:** recordedBy: ROPOS.COM; lifeStage: Adult; behavior: on sediment; occurrenceStatus: present; preparations: Imaged only; associatedMedia: R1914_00060.jpg; **Taxon:** taxonConceptID: Peniagonepurpurea; scientificName: Peniagonepurpurea; kingdom: Animalia; phylum: Echinodermata; class: Holothuroidea; order: Elasipodida; family: Elpidiidae; genus: Peniagone; taxonRank: Species; scientificNameAuthorship: (Théel, 1882); **Location:** waterBody: Indian Ocean; stateProvince: Central Indian Ridge; locality: EGS; verbatimLocality: Cluster 4; maximumDepthInMeters: 3155; locationRemarks: RV Pelagia Cruise INDEX2015 Leg 2; geodeticDatum: WGS84; coordinateUncertaintyInMeters: 31; **Identification:** identifiedBy: Andrey Gebruk, Antonina Kremenetskaia; identificationRemarks: Identified only from imagery; **Event:** eventDate: 2015-12-07; eventTime: 5:22:16 am; year: 2015; fieldNumber: INDEX2015-60ROV; fieldNotes: 1.8°C, 34.7 ppt; **Record Level:** language: en; institutionCode: DZMB; datasetName: INDEX; basisOfRecord: Human Observation

##### Notes

Fig. 172

#### Laetmogonidae

Ekman, 1926

CDDC2C6A-BF68-5486-BE2F-0DB9064BCEFD

#### Laetmogonidae gen. indet.

F30F4F0C-FD56-53E2-8682-67F7A7944F56

##### Materials

- **Type status:** Other material. **Occurrence:** recordedBy: ROPOS.COM; individualCount: 1; lifeStage: Adult; behavior: on seafloor; occurrenceStatus: present; preparations: Imaged only; associatedMedia: R2092_00427.jpg; **Taxon:** taxonConceptID: Laetmogonidae gen. indet.; kingdom: Animalia; phylum: Echinodermata; class: Holothuroidea; order: Elasipodida; family: Laetmogonidae; taxonRank: Family; scientificNameAuthorship: Ekman, 1926; **Location:** waterBody: Indian Ocean; stateProvince: South East Indian Ridge; locality: Vent site 6; verbatimLocality: Cluster 12; maximumDepthInMeters: 2509; locationRemarks: RV Pelagia Cruise INDEX2018 Leg 2; geodeticDatum: WGS84; coordinateUncertaintyInMeters: 25; **Identification:** identifiedBy: Andrey Gebruk, Antonina Kremenetskaia; identificationRemarks: Identified only from imagery; identificationQualifier: gen. indet.; **Event:** eventDate: 2018-11-20; eventTime: 7:46:20 am; year: 2018; fieldNumber: INDEX2018-57ROPOS; fieldNotes: 1.8°C, 34.7 ppt; **Record Level:** language: en; institutionCode: DZMB; datasetName: INDEX; basisOfRecord: Human Observation

##### Notes

Fig. 173

#### Pelagothuriidae

Ludwig, 1893

2CB3F6F5-3E4A-525E-B535-2CEA50DD1B00

#### Enypniastes

Théel, 1882

7A3C6828-17D7-5127-A314-FB1117A52CCC

#### Enypniastes eximia

Théel, 1882

28C342A0-3041-598A-A40B-ACC35B4E96A5

##### Materials

- **Type status:** Other material. **Occurrence:** recordedBy: ROPOS.COM; individualCount: 1; lifeStage: Adult; behavior: Swimming; occurrenceStatus: present; preparations: Imaged only; associatedMedia: R1915_00155.jpg; **Taxon:** taxonConceptID: Enypniasteseximia; scientificName: Enypniasteseximia; kingdom: Animalia; phylum: Echinodermata; class: Holothuroidea; order: Elasipodida; family: Pelagothuriidae; genus: Enypniastes; taxonRank: Species; scientificNameAuthorship: Théel, 1882; **Location:** waterBody: Indian Ocean; stateProvince: Central Indian Ridge; locality: EGS; verbatimLocality: Cluster 4; maximumDepthInMeters: 3313; locationRemarks: RV Pelagia Cruise INDEX2015 Leg 2; geodeticDatum: WGS84; coordinateUncertaintyInMeters: 32; **Identification:** identifiedBy: Andrey Gebruk, Antonina Kremenetskaia; identificationRemarks: Identified only from imagery; **Event:** eventDate: 2015-12-08; eventTime: 6:41:13 am; year: 2015; fieldNumber: INDEX2015-62ROV; **Record Level:** language: en; institutionCode: DZMB; datasetName: INDEX; basisOfRecord: Human Observation

##### Notes

Fig. 174

#### Psychropotidae

Théel, 1882

EA894FF6-B30E-5BE6-9AB6-0E17CB3D59C8

#### Benthodytes

Théel, 1882

5C5382BE-723F-56A9-9D37-25C2B5317BA9

#### Benthodytes sp. indet.

87C2C001-CA67-5411-999A-8016C93F9DCE

##### Materials

- **Type status:** Other material. **Occurrence:** recordedBy: ROPOS.COM; individualCount: 1; lifeStage: Adult; behavior: on sediment; occurrenceStatus: present; preparations: Imaged only; associatedMedia: R2103_00152.jpg; **Taxon:** taxonConceptID: Benthodytes sp. indet.; kingdom: Animalia; phylum: Echinodermata; class: Holothuroidea; order: Elasipodida; family: Psychropotidae; genus: Benthodytes; taxonRank: Genus; scientificNameAuthorship: Théel, 1882; **Location:** waterBody: Indian Ocean; stateProvince: Rodriguez Triple Junction; locality: RTJ; verbatimLocality: Cluster 5; maximumDepthInMeters: 2468; locationRemarks: RV Pelagia Cruise INDEX2018 Leg 2; geodeticDatum: WGS84; coordinateUncertaintyInMeters: 25; **Identification:** identifiedBy: Andrey Gebruk, Antonina Kremenetskaia; identificationRemarks: Identified only from imagery; identificationQualifier: sp. indet.; **Event:** eventDate: 2018-12-03; eventTime: 7:51:11 am; year: 2018; fieldNumber: INDEX2018-82ROPOS; fieldNotes: 1.8°C, 34.7 ppt; **Record Level:** language: en; institutionCode: DZMB; datasetName: INDEX; basisOfRecord: Human Observation

##### Notes

Fig. 175

#### Persiculida

Miller, Kerr, Paulay, Reich, Wilson, Carvajal & Rouse, 2017

A00F927D-9769-5822-9463-89D80A749DA8

#### Benthothuria

Perrier R., 1898

FF80A41E-56ED-5480-9B21-0C9D3B966821

#### Benthothuria gen. inc.

3063CD4B-4C7A-5D0F-AEFE-FC7FB08DF050

##### Materials

- **Type status:** Other material. **Occurrence:** recordedBy: NIOZ; individualCount: 1; lifeStage: Adult; behavior: on sediment; occurrenceStatus: present; preparations: Imaged only; associatedMedia: 20141112222610407.jpg; **Taxon:** taxonConceptID: Benthothuria gen. inc.; kingdom: Animalia; phylum: Echinodermata; class: Holothuroidea; order: Persiculida; genus: Benthothuria; taxonRank: Genus; scientificNameAuthorship: Perrier R., 1898; **Location:** waterBody: Indian Ocean; stateProvince: South East Indian Ridge; locality: SEIR; verbatimLocality: Cluster 6; maximumDepthInMeters: 3123; locationRemarks: RV Pelagia Cruise INDEX2014 Leg 1; geodeticDatum: WGS84; coordinateUncertaintyInMeters: 32; **Identification:** identifiedBy: Andrey Gebruk, Antonina Kremenetskaia; identificationRemarks: Identified only from imagery; identificationQualifier: gen. inc.; **Event:** eventDate: 2014-11-12; eventTime: 10:26:10 pm; year: 2014; fieldNumber: INDEX2014-24VS; fieldNotes: 1.7°C; **Record Level:** language: en; institutionCode: DZMB; datasetName: INDEX; basisOfRecord: Human Observation

##### Notes

Fig. 176

#### Pseudostichopodidae

Miller, Kerr, Paulay, Reich, Wilson, Carvajal & Rouse, 2017

566FF76B-CA05-543F-A208-9A511CBD11ED

#### Pseudostichopus

Théel, 1886

4C0BD2AD-96F2-541A-8F9B-913EFA6D3E95

#### Pseudostichopus gen. inc. (DZMB_2021_0073)

27FC17A3-5C9F-5690-9EBB-A1529A2C2CA1

##### Materials

- **Type status:** Other material. **Occurrence:** recordedBy: NIOZ; individualCount: 1; lifeStage: Adult; behavior: on sediment; occurrenceStatus: present; preparations: Imaged only; associatedMedia: 20141128074843177.jpg; **Taxon:** taxonConceptID: Pseudostichopus gen. inc. (DZMB_2021_0073); kingdom: Animalia; phylum: Echinodermata; class: Holothuroidea; order: Persiculida; family: Pseudostichopodidae; genus: Pseudostichopus; taxonRank: Genus; scientificNameAuthorship: Théel, 1886; **Location:** waterBody: Indian Ocean; stateProvince: South East Indian Ridge; locality: SEIR; verbatimLocality: Cluster 9; maximumDepthInMeters: 2710; locationRemarks: RV Pelagia Cruise INDEX2014 Leg 1; geodeticDatum: WGS84; coordinateUncertaintyInMeters: 27; **Identification:** identifiedBy: Andrey Gebruk, Antonina Kremenetskaia; identificationRemarks: Identified only from imagery; identificationQualifier: gen. inc.; **Event:** eventDate: 2014-11-28; eventTime: 7:48:43 am; year: 2014; fieldNumber: INDEX2014-43VS; fieldNotes: 1.7°C; **Record Level:** language: en; institutionCode: DZMB; datasetName: INDEX; basisOfRecord: Human Observation

##### Notes

Fig. 177

#### Pseudostichopus sp. indet. (DZMB_2021_0074)

D9BA4F75-9517-5D55-AFEE-17AB67CC3F04

##### Materials

- **Type status:** Other material. **Occurrence:** recordedBy: ROPOS.COM; individualCount: 1; lifeStage: Adult; behavior: on sediment; occurrenceStatus: present; preparations: Imaged only; associatedMedia: R2092_00588.jpg; **Taxon:** taxonConceptID: Pseudostichopus sp. indet. (DZMB_2021_0074); kingdom: Animalia; phylum: Echinodermata; class: Holothuroidea; order: Persiculida; family: Pseudostichopodidae; genus: Pseudostichopus; taxonRank: Genus; scientificNameAuthorship: Théel, 1886; **Location:** waterBody: Indian Ocean; stateProvince: South East Indian Ridge; locality: Vent site 6; verbatimLocality: Cluster 12; maximumDepthInMeters: 2496; locationRemarks: RV Pelagia Cruise INDEX2018 Leg 2; geodeticDatum: WGS84; coordinateUncertaintyInMeters: 25; **Identification:** identifiedBy: Andrey Gebruk, Antonina Kremenetskaia; identificationRemarks: Identified only from imagery; identificationQualifier: sp. indet.; **Event:** eventDate: 2018-11-20; eventTime: 11:44:51 am; year: 2018; fieldNumber: INDEX2018-57ROPOS; fieldNotes: 1.8°C, 34.7 ppt; **Record Level:** language: en; institutionCode: DZMB; datasetName: INDEX; basisOfRecord: Human Observation

##### Notes

Fig. 178

#### Synallactida

Miller, Kerr, Paulay, Reich, Wilson, Carvajal & Rouse, 2017

1CFAF210-3939-5A24-8FE2-B2C2AAEAB9AF

#### Deimatidae

Théel, 1882

36A9A211-51EC-5DB9-9E57-59A71BAC9C04

#### Oneirophanta

Théel, 1879

A5E934A4-E716-5195-8C8E-9FB787B74869

#### Oneirophanta sp. indet.

6B079D44-0213-5B6B-B2AD-6AA8749F5CA7

##### Materials

- **Type status:** Other material. **Occurrence:** recordedBy: BGR; individualCount: 1; lifeStage: Adult; behavior: on seafloor; occurrenceStatus: present; preparations: Imaged only; associatedMedia: 17MFT Fotos 2013-308.jpg; **Taxon:** taxonConceptID: Oneirophanta sp. indet.; kingdom: Animalia; phylum: Echinodermata; class: Holothuroidea; order: Synallactida; family: Deimatidae; genus: Oneirophanta; taxonRank: Genus; scientificNameAuthorship: Théel, 1879; **Location:** waterBody: Indian Ocean; stateProvince: Central Indian Ridge; locality: MESO; verbatimLocality: outside INDEX claim; maximumDepthInMeters: 2850; locationRemarks: FS Sonne Cruise INDEX2013 Leg 1; decimalLatitude: -23.3839; decimalLongitude: 69.2378; geodeticDatum: WGS84; coordinateUncertaintyInMeters: 28; **Identification:** identifiedBy: Andrey Gebruk, Antonina Kremenetskaia; identificationRemarks: Identified only from imagery; identificationQualifier: sp. indet.; **Event:** eventDate: 2013-11-25; eventTime: 5:30:51 am; year: 2013; fieldNumber: INDEX2013-17MFT; fieldNotes: 1.7°C, 34.7 ppt; **Record Level:** language: en; institutionCode: DZMB; datasetName: INDEX; basisOfRecord: Human Observation

##### Notes

Fig. 179

#### Synallactidae

Ludwig, 1894

254261E7-8634-590C-AB54-0937A6A36884

#### Synallactidae gen. indet. (DZMB_2021_0075)

EA2AD2E3-8659-59D1-A99E-18D4CDE602DA

##### Materials

- **Type status:** Other material. **Occurrence:** recordedBy: BGR/ GEOMAR; individualCount: 1; lifeStage: Adult; behavior: on basalt; occurrenceStatus: present; preparations: Imaged only; associatedMedia: 2013-12-15_10-35-24_Sonne_INDEX2013-2_057ROV09_Logo.jpg; **Taxon:** taxonConceptID: Synallactidae gen. indet. (DZMB_2021_0075); kingdom: Animalia; phylum: Echinodermata; class: Holothuroidea; order: Synallactida; family: Synallactidae; taxonRank: Family; scientificNameAuthorship: Ludwig, 1894; **Location:** waterBody: Indian Ocean; stateProvince: Central Indian Ridge; locality: MESO; verbatimLocality: outside INDEX claim; maximumDepthInMeters: 2826; locationRemarks: FS Sonne Cruise INDEX2013 Leg 2; geodeticDatum: WGS84; coordinateUncertaintyInMeters: 30; **Identification:** identifiedBy: Andrey Gebruk, Antonina Kremenetskaia; identificationRemarks: Identified only from imagery; identificationQualifier: gen. indet.; **Event:** eventDate: 2013-12-15; eventTime: 10:35:24 am; year: 2013; fieldNumber: INDEX2013-57ROV; **Record Level:** language: en; institutionCode: DZMB; datasetName: INDEX; basisOfRecord: Human Observation

##### Notes

Fig. 180

#### Synallactidae gen. indet. (DZMB_2021_0076)

**Figure 95.**
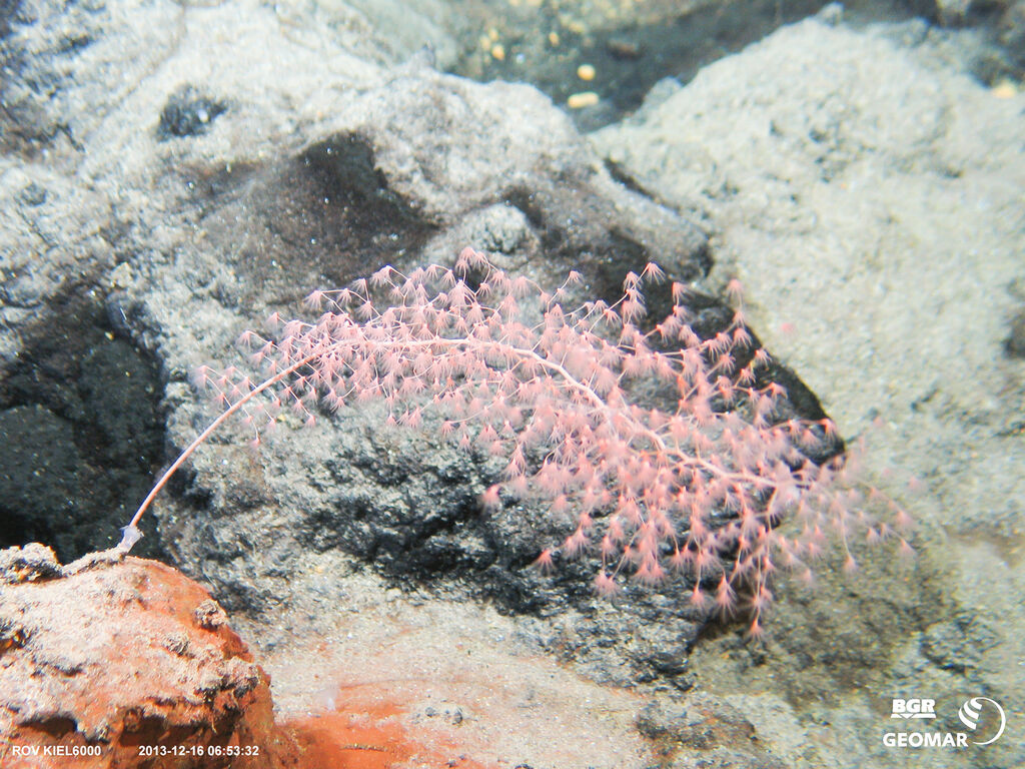
*Chrysogorgia* sp. indet. (DZMB_2021_0034) in situ at the Central Indian Ridge within the MESO area outside the INDEX area. Image corresponds with the data (Image attribution: BGR/ GEOMAR).

**Figure 164.**
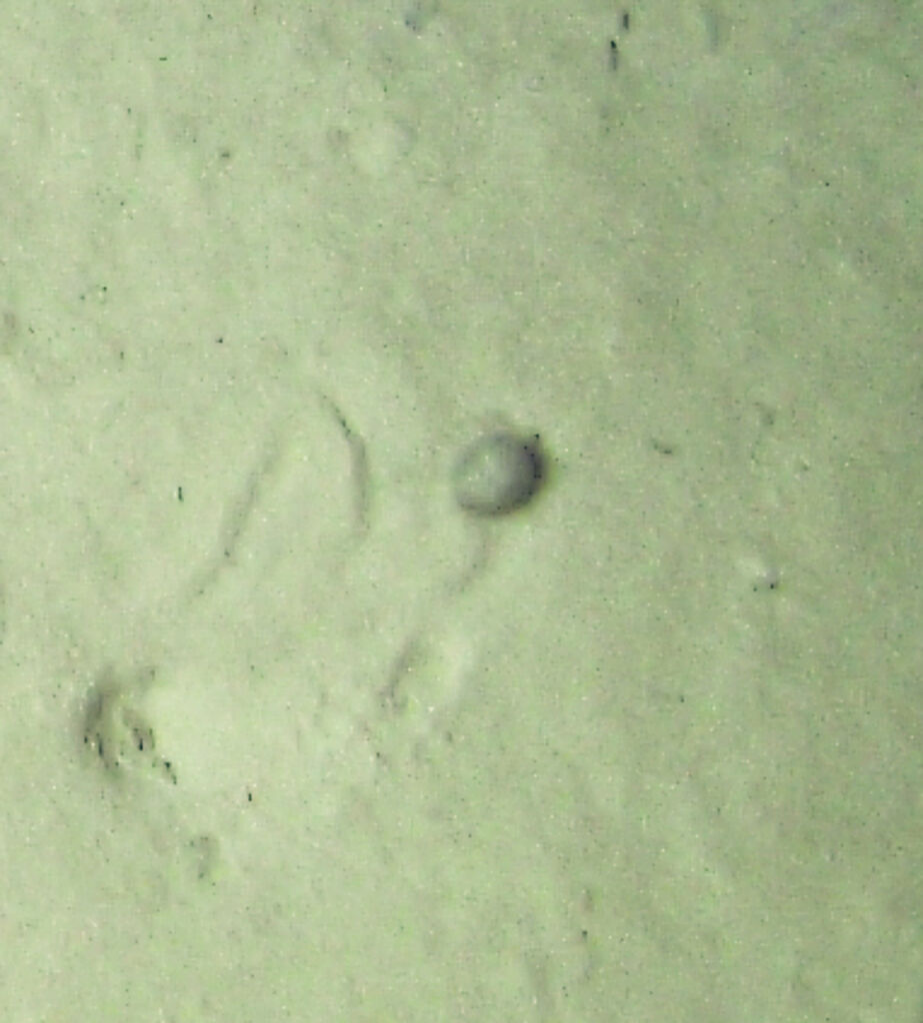
Irregularia infracl. inc. in situ at the South East Indian Ridge in Cluster 9 of the INDEX area. Image corresponds with the data (Image attribution: BGR).

**Figure 166.**
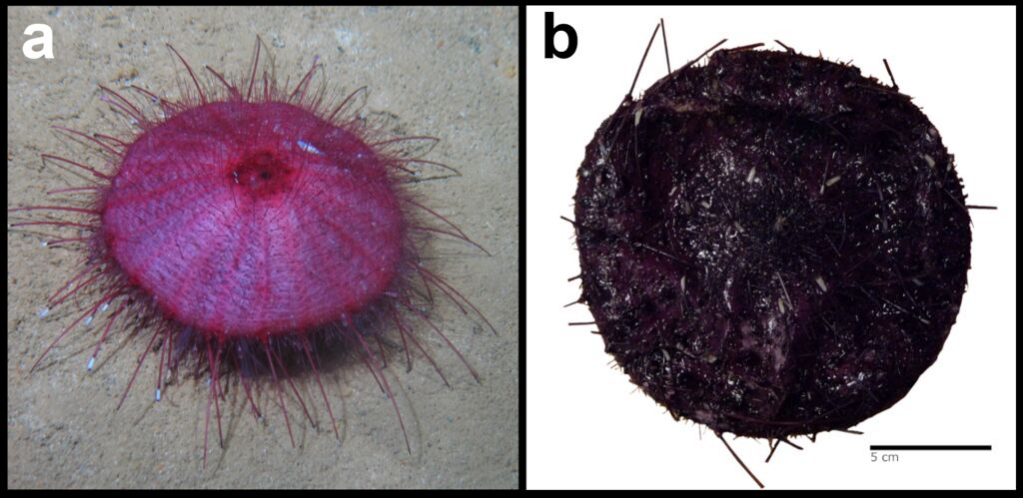
*Hapalosoma* sp. indet. in situ (a) and sampled specimen (b) in the surrounding area of the Edmond hydrothermal vent field in Cluster 4 of the INDEX area. Image corresponds with the data (Image attribution: BGR and GEOMAR).

**Figure 167.**
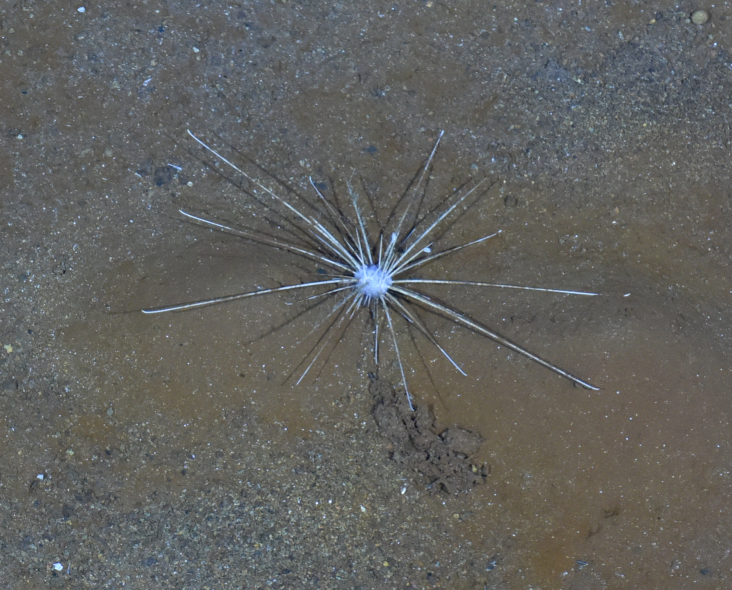
*Salenocidaris* sp. indet. in situ in the surrounding area of the vent site 6 hydrothermal vent field in Cluster 12 of the INDEX area. Image corresponds with the data (Image attribution: BGR).

F1AE7282-DEBF-5C91-8DA9-9ABB95C9475B

##### Materials

- **Type status:** Other material. **Occurrence:** recordedBy: ROPOS.COM; individualCount: 1; lifeStage: Adult; behavior: on seafloor; occurrenceStatus: present; preparations: Imaged only; associatedMedia: R1914_00119.jpg; **Taxon:** taxonConceptID: Synallactidae gen. indet. (DZMB_2021_0076); kingdom: Animalia; phylum: Echinodermata; class: Holothuroidea; order: Synallactida; family: Synallactidae; taxonRank: Family; scientificNameAuthorship: Ludwig, 1894; **Location:** waterBody: Indian Ocean; stateProvince: Central Indian Ridge; locality: EGS; verbatimLocality: Cluster 4; maximumDepthInMeters: 3199; locationRemarks: RV Pelagia Cruise INDEX2015 Leg 2; geodeticDatum: WGS84; coordinateUncertaintyInMeters: 31; **Identification:** identifiedBy: Andrey Gebruk, Antonina Kremenetskaia; identificationRemarks: Identified only from imagery; identificationQualifier: gen. indet.; **Event:** eventDate: 2015-12-07; eventTime: 6:11:49 am; year: 2015; fieldNumber: INDEX2015-60ROV; fieldNotes: 1.8°C, 34.7 ppt; **Record Level:** language: en; institutionCode: DZMB; datasetName: INDEX; basisOfRecord: Human Observation

##### Notes

Fig. 181

#### Synallactidae gen. indet. (DZMB_2021_0077)

8B701AA8-1E72-5A0D-B7FC-A511FE86C576

##### Materials

- **Type status:** Other material. **Occurrence:** recordedBy: ROPOS.COM; individualCount: 1; lifeStage: Adult; behavior: on basalt; occurrenceStatus: present; preparations: Imaged only; associatedMedia: R2106_00172.jpg; **Taxon:** taxonConceptID: Synallactidae gen. indet. (DZMB_2021_0077); kingdom: Animalia; phylum: Echinodermata; class: Holothuroidea; order: Synallactida; family: Synallactidae; taxonRank: Family; scientificNameAuthorship: Ludwig, 1894; **Location:** waterBody: Indian Ocean; stateProvince: Rodriguez Triple Junction; locality: Vent site 4; verbatimLocality: Cluster 5; maximumDepthInMeters: 2500; locationRemarks: RV Pelagia Cruise INDEX2018 Leg 2; geodeticDatum: WGS84; coordinateUncertaintyInMeters: 23; **Identification:** identifiedBy: Andrey Gebruk, Antonina Kremenetskaia; identificationRemarks: Identified only from imagery; identificationQualifier: gen. indet.; **Event:** eventDate: 2018-12-10; eventTime: 9:26:25 am; year: 2018; fieldNumber: INDEX2018-97ROPOS; fieldNotes: 1.9°C, 34.7 ppt; **Record Level:** language: en; institutionCode: DZMB; datasetName: INDEX; basisOfRecord: Human Observation

##### Notes

Fig. 182

#### Synallactidae gen. indet. (DZMB_2021_0078)

C35E747C-A2F4-52C9-8522-CB0121B1EE42

##### Materials

- **Type status:** Other material. **Occurrence:** recordedBy: IFREMER; individualCount: 1; lifeStage: Adult; behavior: on basalt; occurrenceStatus: present; preparations: Imaged only; associatedMedia: 160122004312598_16_1080i Kopie.jpg; **Taxon:** taxonConceptID: Synallactidae gen. indet. (DZMB_2021_0078); kingdom: Animalia; phylum: Echinodermata; class: Holothuroidea; order: Synallactida; family: Synallactidae; taxonRank: Family; scientificNameAuthorship: Ludwig, 1894; **Location:** waterBody: Indian Ocean; stateProvince: South East Indian Ridge; locality: Pelagia; verbatimLocality: Cluster 8; maximumDepthInMeters: 3687; locationRemarks: RV Pourqoui pas? Cruise INDEX2016 Leg 1; geodeticDatum: WGS84; coordinateUncertaintyInMeters: 37; **Identification:** identifiedBy: Andrey Gebruk, Antonina Kremenetskaia; identificationRemarks: Identified only from imagery; identificationQualifier: gen. indet.; **Event:** eventDate: 2016-01-22; eventTime: 12:43:12 am; year: 2016; fieldNumber: INDEX2016-20ROV; fieldNotes: 1.7°C, 34.7 ppt; **Record Level:** language: en; institutionCode: DZMB; datasetName: INDEX; basisOfRecord: Human Observation

##### Notes

Fig. 183

#### Synallactidae fam. inc. (DZMB_2021_0079)

19E6CFDB-7A60-5B6F-83F7-A9F62B44A1F7

##### Materials

- **Type status:** Other material. **Occurrence:** recordedBy: BGR; individualCount: 1; lifeStage: Adult; behavior: on seafloor; occurrenceStatus: present; preparations: Imaged only; associatedMedia: 17MFT Fotos 2013-292.jpg; **Taxon:** taxonConceptID: Synallactidae fam. inc. (DZMB_2021_0079); kingdom: Animalia; phylum: Echinodermata; class: Holothuroidea; order: Synallactida; family: Synallactidae; taxonRank: Family; scientificNameAuthorship: Ludwig, 1894; **Location:** waterBody: Indian Ocean; stateProvince: Central Indian Ridge; locality: MESO; verbatimLocality: outside INDEX claim; maximumDepthInMeters: 2826; locationRemarks: FS Sonne Cruise INDEX2013 Leg 1; decimalLatitude: -23.3930; decimalLongitude: 69.2428; geodeticDatum: WGS84; coordinateUncertaintyInMeters: 28; **Identification:** identifiedBy: Andrey Gebruk, Antonina Kremenetskaia; identificationRemarks: Identified only from imagery; identificationQualifier: fam. inc.; **Event:** eventDate: 2013-11-25; eventTime: 3:04:50 am; year: 2013; fieldNumber: INDEX2013-17MFT; fieldNotes: 1.8°C, 34.7 ppt; **Record Level:** language: en; institutionCode: DZMB; datasetName: INDEX; basisOfRecord: Human Observation

##### Notes

Fig. 184

#### Synallactes

Ludwig, 1894

28EA3DD9-CB34-5A52-A1CC-51E0F175ADCC

#### Synallactes sp. indet.

164494F9-D2C9-51AF-AA96-87B09541A933

##### Materials

- **Type status:** Other material. **Occurrence:** recordedBy: ROPOS.COM; individualCount: 1; lifeStage: Adult; behavior: on sulphides; occurrenceStatus: present; preparations: Imaged only; associatedMedia: R2098_00180.jpg; **Taxon:** taxonConceptID: Synallactes sp. indet.; kingdom: Animalia; phylum: Echinodermata; class: Holothuroidea; order: Synallactida; family: Synallactidae; genus: Synallactes; taxonRank: Genus; scientificNameAuthorship: Ludwig, 1894; **Location:** waterBody: Indian Ocean; stateProvince: South East Indian Ridge; locality: Vent site 3; verbatimLocality: Cluster 12; maximumDepthInMeters: 2530; locationRemarks: RV Pelagia Cruise INDEX2018 Leg 2; geodeticDatum: WGS84; coordinateUncertaintyInMeters: 26; **Identification:** identifiedBy: Andrey Gebruk, Antonina Kremenetskaia; identificationRemarks: Identified only from imagery; identificationQualifier: sp. indet.; **Event:** eventDate: 2018-11-26; eventTime: 8:11:10 am; year: 2018; fieldNumber: INDEX2018-70ROPOS; fieldNotes: 1.8°C, 34.7 ppt; **Record Level:** language: en; institutionCode: DZMB; datasetName: INDEX; basisOfRecord: Human Observation

##### Notes

Fig. 185

#### Ophiuroidea

Gray, 1840

56E2A5E8-E352-5858-A5F3-C63802DD54EB

#### Amphilepidida

O’Hara, Hugall, Thuy, Stöhr & Martynov, 2017

045A5D14-2E42-5F89-92D6-F43B0274C511

#### Amphilepidida ord. inc.

E98057C7-AA3E-5621-96CE-1F3A1A85B80E

##### Materials

- **Type status:** Other material. **Occurrence:** recordedBy: ROPOS.COM; individualCount: 1; lifeStage: Adult; behavior: on seafloor; occurrenceStatus: present; preparations: Imaged only; associatedMedia: R2106_00236.jpg; **Taxon:** taxonConceptID: Amphilepidida ord. inc.; kingdom: Animalia; phylum: Echinodermata; class: Ophiuroidea; order: Amphilepidida; taxonRank: Order; scientificNameAuthorship: O'Hara, Hugall, Thuy, Stöhr & Martynov, 2017; **Location:** waterBody: Indian Ocean; stateProvince: Rodriguez Triple Junction; locality: Vent site 4; verbatimLocality: Cluster 5; maximumDepthInMeters: 2476; locationRemarks: RV Pelagia Cruise INDEX2018 Leg 2; geodeticDatum: WGS84; coordinateUncertaintyInMeters: 23; **Identification:** identifiedBy: Sabine Stöhr; identificationRemarks: Identified only from imagery; identificationQualifier: ord. inc.; **Event:** eventDate: 2018-12-10; eventTime: 10:55:46 am; year: 2018; fieldNumber: INDEX2018-97ROPOS; fieldNotes: 1.8°C, 34.7 ppt; **Record Level:** language: en; institutionCode: DZMB; datasetName: INDEX; basisOfRecord: Human Observation

##### Notes

Fig. 186

#### Euryalida

Lamarck, 1816

6AA09ACA-882B-55ED-B983-C656030A9076

#### Asteronychidae

Ljungman, 1867

A0A34354-8CF0-5431-8690-8EB3AC7312F4

#### Asteronyx

Müller & Troschel, 1842

5C886C0A-2226-5E87-B3E7-B34861B2C84F

#### Asteronyx gen. inc.

7345316E-0966-543F-AB83-F535A173D7A6

##### Materials

- **Type status:** Other material. **Occurrence:** recordedBy: ROPOS.COM; individualCount: 1; lifeStage: Adult; behavior: sitting on coral stalk; occurrenceStatus: present; preparations: Imaged only; associatedMedia: R2097_00145.jpg; **Taxon:** taxonConceptID: Asteronyx gen. inc.; kingdom: Animalia; phylum: Echinodermata; class: Ophiuroidea; order: Euryalida; family: Asteronychidae; genus: Asteronyx; taxonRank: Genus; scientificNameAuthorship: Müller & Troschel, 1842; **Location:** waterBody: Indian Ocean; stateProvince: South East Indian Ridge; locality: Vent site 6; verbatimLocality: Cluster 12; maximumDepthInMeters: 2374; locationRemarks: RV Pelagia Cruise INDEX2018 Leg 2; geodeticDatum: WGS84; coordinateUncertaintyInMeters: 24; **Identification:** identifiedBy: Sabine Stöhr; identificationRemarks: Identified only from imagery; identificationQualifier: gen. inc.; **Event:** eventDate: 2018-11-25; eventTime: 8:14:41 am; year: 2018; fieldNumber: INDEX2018-67ROPOS; **Record Level:** language: en; institutionCode: DZMB; datasetName: INDEX; basisOfRecord: Human Observation

##### Notes

Fig. 187

#### Ophiacanthida

O’Hara, Hugall, Thuy, Stöhr & Martynov, 2017

53D46FA3-FC3B-5BBE-8888-6F9C90DB3FB0

#### Ophiacanthida ord. inc.

09EAB9DA-96E0-595C-A43A-C582BD4662FF

##### Materials

- **Type status:** Other material. **Occurrence:** recordedBy: ROPOS.COM; individualCount: 1; lifeStage: Adult; behavior: sitting on porifera; occurrenceStatus: present; preparations: Imaged only; associatedMedia: R2101_00144.jpg; **Taxon:** taxonConceptID: Ophiacanthida ord. inc.; kingdom: Animalia; phylum: Echinodermata; class: Ophiuroidea; order: Ophiacanthida; taxonRank: Order; scientificNameAuthorship: O'Hara, Hugall, Thuy, Stöhr & Martynov, 2017; **Location:** waterBody: Indian Ocean; stateProvince: South East Indian Ridge; locality: Vent site 5; verbatimLocality: Cluster 11; maximumDepthInMeters: 2909; locationRemarks: RV Pelagia Cruise INDEX2018 Leg 2; geodeticDatum: WGS84; coordinateUncertaintyInMeters: 29; **Identification:** identifiedBy: Sabine Stöhr; identificationRemarks: Identified only from imagery; identificationQualifier: ord. inc.; **Event:** eventDate: 2018-11-29; eventTime: 10:07:41 am; year: 2018; fieldNumber: INDEX2018-75ROPOS; fieldNotes: 1.7°C, 34.7 ppt; **Record Level:** language: en; institutionCode: DZMB; datasetName: INDEX; basisOfRecord: Human Observation

##### Notes

Fig. 188

#### Ophiurida

Müller & Troschel, 1840 sensu O’Hara et al., 2017

AD567D01-9A3A-56AB-B026-A76B6CDBE9AB

#### Ophiophyllum

Lyman, 1878

CF396094-C7F0-5F45-A20D-EB5631DF9413

#### Ophiophyllum petilum sp. inc.

Lyman, 1878

18C38C0C-3418-5E5C-B901-C739DA3A4B0A

##### Materials

- **Type status:** Other material. **Occurrence:** recordedBy: ROPOS.COM; individualCount: 1; lifeStage: Adult; behavior: on seafloor; occurrenceStatus: present; preparations: DNA voucher and animal stored in 96% ethanol; associatedMedia: R2102_00129.jpg; associatedOccurrences: none; associatedSequences: COI; **Taxon:** taxonID: I18_1297; scientificNameID: -; taxonConceptID: Ophiophyllumpetilum sp. inc.; scientificName: Ophiophyllumpetilum; kingdom: Animalia; phylum: Echinodermata; class: Ophiuroidea; order: Ophiurida; genus: Ophiophyllum; taxonRank: Species; scientificNameAuthorship: Lyman, 1878; **Location:** waterBody: Indian Ocean; stateProvince: South East Indian Ridge; locality: Vent site 5; verbatimLocality: Cluster 11; maximumDepthInMeters: 2943; locationRemarks: RV Pelagia Cruise INDEX2018 Leg 2; geodeticDatum: WGS84; coordinateUncertaintyInMeters: 30; **Identification:** identifiedBy: Sabine Stöhr; identificationRemarks: Identified by morphology and DNA of collected specimen; identificationQualifier: sp. inc.; **Event:** eventDate: 2018-12-01; eventTime: 9:41:50 am; year: 2018; fieldNumber: INDEX2018-80ROPOS; fieldNotes: 1.7°C, 34.7 ppt; **Record Level:** language: en; institutionCode: DZMB; collectionCode: I18_080RO_A_004; datasetName: INDEX; basisOfRecord: Human Observation

##### Notes

Fig. 189

#### Ophiosphalmidae

O’Hara, Stöhr, Hugall, Thuy & Martynov, 2018

DF44DEF9-4FF4-5A69-927A-E71B7CA3847E

#### Ophiosphalma

H.L. Clark, 1941

3A26537D-DB08-50A9-8CC0-5DA090DBDC4A

#### Ophiosphalma gen. inc.

C54ED6B4-7B84-5A71-B6F3-BFDE53D1CE94

##### Materials

- **Type status:** Other material. **Occurrence:** recordedBy: ROPOS.COM; individualCount: 1; lifeStage: Adult; behavior: on seafloor; occurrenceStatus: present; preparations: Imaged only; associatedMedia: R2094_01004.jpg; **Taxon:** taxonConceptID: Ophiosphalma gen. inc.; kingdom: Animalia; phylum: Echinodermata; class: Ophiuroidea; order: Ophiurida; family: Ophiosphalmidae; genus: Ophiosphalma; taxonRank: Genus; scientificNameAuthorship: H.L. Clark, 1941; **Location:** waterBody: Indian Ocean; stateProvince: South East Indian Ridge; locality: Vent site 6; verbatimLocality: Cluster 12; maximumDepthInMeters: 2458; locationRemarks: RV Pelagia Cruise INDEX2018 Leg 2; geodeticDatum: WGS84; coordinateUncertaintyInMeters: 25; **Identification:** identifiedBy: Sabine Stöhr; identificationRemarks: Identified only from imagery; identificationQualifier: gen. inc.; **Event:** eventDate: 2018-11-22; eventTime: 5:52:04 am; year: 2018; fieldNumber: INDEX2018-61ROPOS; fieldNotes: 1.8°C, 34.7 ppt; **Record Level:** language: en; institutionCode: DZMB; datasetName: INDEX; basisOfRecord: Human Observation

##### Notes

Fig. 190

#### Ophiosphalma armigerum sp. inc.

(Lyman, 1878)

A8FE64ED-B537-52F7-BC2E-7AC583766A4E

##### Materials

- **Type status:** Other material. **Occurrence:** recordedBy: ROPOS.COM; individualCount: 1; lifeStage: Adult; behavior: on seafloor; occurrenceStatus: present; preparations: DNA voucher and animal stored freeze dried; associatedMedia: R2092_00505.jpg; **Taxon:** taxonID: I18_0990; taxonConceptID: Ophiosphalmaarmigerum sp. inc.; scientificName: Ophiosphalmaarmigerum; kingdom: Animalia; phylum: Echinodermata; class: Ophiuroidea; order: Ophiurida; family: Ophiosphalmidae; genus: Ophiosphalma; taxonRank: Species; scientificNameAuthorship: (Lyman, 1878); **Location:** waterBody: Indian Ocean; stateProvince: South East Indian Ridge; locality: Vent site 6; verbatimLocality: Cluster 12; maximumDepthInMeters: 2498; locationRemarks: RV Pelagia Cruise INDEX2018 Leg 2; geodeticDatum: WGS84; coordinateUncertaintyInMeters: 25; **Identification:** identifiedBy: Sabine Stöhr; identificationRemarks: Identified by morphology and DNA of collected specimen; identificationQualifier: sp. inc.; **Event:** eventDate: 2018-11-20; eventTime: 9:22:46 am; year: 2018; fieldNumber: INDEX2018-57ROPOS; fieldNotes: 1.8°C, 34.7 ppt; **Record Level:** language: en; institutionCode: DZMB; collectionCode: I18_057RO_PC6_001; datasetName: INDEX; basisOfRecord: Human Observation

##### Notes

Fig. 191

#### Hemichordata

Bateson, 1885

F588D945-F201-550E-BF8B-67EDFB630EC0

#### Enteropneusta

Gegenbaur, 1870

2FCD7B91-C53B-50F6-B076-EB106A35956D

#### Enteropneusta

E15AC012-2B7A-5583-95B7-1830B22E9282

#### Torquaratoridae

Holland, Clague, Gordon, Gebruk, Pawson & Vecchione, 2005

A20A9075-8A79-5C82-9752-7910DED28731

#### Torquaratoridae fam. inc.

4C1F922B-EE33-5EB0-9D3B-3FDB880826F9

##### Materials

- **Type status:** Other material. **Occurrence:** recordedBy: NIOZ; individualCount: 1; lifeStage: Adult; behavior: on sediment; occurrenceStatus: present; preparations: Imaged only; associatedMedia: 20141128124542012.jpg; **Taxon:** taxonConceptID: Torquaratoridae fam. inc.; kingdom: Animalia; phylum: Hemichordata; class: Enteropneusta; order: Enteropneusta; family: Torquaratoridae; taxonRank: Family; scientificNameAuthorship: Holland, Clague, Gordon, Gebruk, Pawson & Vecchione, 2005; **Location:** waterBody: Indian Ocean; stateProvince: South East Indian Ridge; locality: SEIR; verbatimLocality: Cluster 9; maximumDepthInMeters: 3356; locationRemarks: RV Pelagia Cruise INDEX2014 Leg 1; geodeticDatum: WGS84; coordinateUncertaintyInMeters: 36; **Identification:** identifiedBy: Terue C. Kihara, Klaas Gerdes; identificationRemarks: Identified only from imagery; identificationQualifier: fam. inc.; **Event:** eventDate: 2014-11-28; eventTime: 12:45:42 pm; year: 2014; fieldNumber: INDEX2014-44VS; fieldNotes: 1.7°C; **Record Level:** language: en; institutionCode: DZMB; datasetName: INDEX; basisOfRecord: Human Observation

##### Notes

Fig. 192

#### Mollusca

B951976A-A9B2-5580-B3FC-CF4F40D1D128

#### Bivalvia

Linnaeus, 1758

2B8D9C12-1AC4-5D51-8727-0582EFDF5795

#### Mytilida

Ferussac, 1822

8559DC3A-303D-5B51-B9C6-8EC91D7587F8

#### Mytilidae

Rafinesque, 1815

0E5C358C-E57E-58FF-B245-8DCC21CBB963

#### Bathymodiolus

Kenk & B. R. Wilson, 1985

F2BB93C0-7F86-58FE-94E0-C21833C8767B

#### Bathymodiolus septemdierum sp. inc.

Hashimoto & Okutani, 1994

F3C0A312-DD84-5F9B-AB0A-C545BD02999C

##### Materials

- **Type status:** Other material. **Occurrence:** recordedBy: ROPOS.COM; individualCount: 100; lifeStage: Adult; behavior: on sulphides; occurrenceStatus: present; preparations: Imaged only; associatedMedia: R2098_00281.jpg; **Taxon:** taxonConceptID: Bathymodiolusseptemdierum sp. inc.; scientificName: Bathymodiolusseptemdierum; kingdom: Animalia; phylum: Mollusca; class: Bivalvia; order: Mytilida; family: Mytilidae; genus: Bathymodiolus; taxonRank: Species; scientificNameAuthorship: Hashimoto & Okutani, 1994; **Location:** waterBody: Indian Ocean; stateProvince: South East Indian Ridge; locality: Vent site 3; verbatimLocality: Cluster 12; maximumDepthInMeters: 2537; locationRemarks: RV Pelagia Cruise INDEX2018 Leg 2; geodeticDatum: WGS84; coordinateUncertaintyInMeters: 26; **Identification:** identifiedBy: Leon Hoffman; identificationRemarks: Identified only from imagery; identificationQualifier: sp. inc.; **Event:** eventDate: 2018-11-26; eventTime: 10:47:02 am; year: 2018; fieldNumber: INDEX2018-70ROPOS; fieldNotes: 1.8°C, 34.7 ppt; **Record Level:** language: en; institutionCode: DZMB; datasetName: INDEX; basisOfRecord: Human Observation

##### Notes

Fig. 193

#### Cephalopoda

Cuvier, 1795

A8687E24-CD6A-5DA8-9E69-72A989B2E31A

#### Octopoda

Leach, 1818

8ADFC54F-C462-56F3-80FA-4E7059BEC613

#### Bathypolypodidae

Robson, 1929

C07DCFC1-8A43-582D-8F89-9B5DE37253D9

#### Bathypolypus

Grimpe, 1921

B043C3D3-AD0B-5623-B160-8F5540235234

#### Bathypolypus sp. indet.

FAA5FD9C-6F93-597D-A630-BF8C2B8E8703

##### Materials

- **Type status:** Other material. **Occurrence:** recordedBy: ROPOS.COM; lifeStage: Adult; behavior: Crawling on seafloor; occurrenceStatus: present; preparations: Imaged only; associatedMedia: R2100_00033.jpg; **Taxon:** taxonConceptID: Bathypolypus sp. indet.; kingdom: Animalia; phylum: Mollusca; class: Cephalopoda; order: Octopoda; family: Bathypolypodidae; genus: Bathypolypus; taxonRank: Genus; scientificNameAuthorship: Grimpe, 1921; **Location:** waterBody: Indian Ocean; stateProvince: South East Indian Ridge; locality: Vent site 5; verbatimLocality: Cluster 11; maximumDepthInMeters: 2908; locationRemarks: RV Pelagia Cruise INDEX2018 Leg 2; geodeticDatum: WGS84; coordinateUncertaintyInMeters: 29; **Identification:** identifiedBy: Kathrin Bolstad; identificationRemarks: Identified only from imagery; identificationQualifier: sp. indet.; **Event:** eventDate: 2018-11-28; eventTime: 6:31:00 am; year: 2018; fieldNumber: INDEX2018-73ROPOS; fieldNotes: 1.7°C, 34.7 ppt; **Record Level:** language: en; institutionCode: DZMB; datasetName: INDEX; basisOfRecord: Human Observation

##### Notes

Fig. 194

#### Cirroteuthidae

Keferstein, 1866

4BF9A356-9890-53DB-9F18-0CFA4F897237

#### Cirroteuthis

Eschricht, 1838

73E016DF-1449-5A9D-B3E8-9B214AA00BC7

#### Cirroteuthis sp. indet.

5A5968C6-CB6A-5877-9F7B-EB1456071E42

##### Materials

- **Type status:** Other material. **Occurrence:** recordedBy: ROPOS.COM; lifeStage: Adult; behavior: Swimming; occurrenceStatus: present; preparations: Imaged only; associatedMedia: R2106_00183.jpg; **Taxon:** taxonConceptID: Cirroteuthis sp. indet.; kingdom: Animalia; phylum: Mollusca; class: Cephalopoda; order: Octopoda; family: Cirroteuthidae; genus: Cirroteuthis; taxonRank: Genus; scientificNameAuthorship: Eschricht, 1838; **Location:** waterBody: Indian Ocean; stateProvince: Rodriguez Triple Junction; locality: Vent site 4; verbatimLocality: Cluster 5; maximumDepthInMeters: 2412; locationRemarks: RV Pelagia Cruise INDEX2018 Leg 2; geodeticDatum: WGS84; coordinateUncertaintyInMeters: 23; **Identification:** identifiedBy: Kathrin Bolstad; identificationRemarks: Identified only from imagery; identificationQualifier: sp. indet.; **Event:** eventDate: 2018-12-10; eventTime: 9:47:41 am; year: 2018; fieldNumber: INDEX2018-97ROPOS; fieldNotes: 1.9°C, 34.7 ppt; **Record Level:** language: en; institutionCode: DZMB; datasetName: INDEX; basisOfRecord: Human Observation

##### Notes

Fig. 195

#### Opisthoteuthidae

Verrill, 1896

3EDDE338-B300-5ECD-A239-FC2B5BE0004F

#### Grimpoteuthis

Robson, 1932

914339AD-99C7-5F2D-AB9C-5CB63586668D

#### Grimpoteuthis gen. inc.

9DC122E0-4BAF-5637-9B4E-37B59F2B71D9

##### Materials

- **Type status:** Other material. **Occurrence:** recordedBy: ROPOS.COM; lifeStage: Adult; behavior: Swimming; occurrenceStatus: present; preparations: Imaged only; associatedMedia: R2103_00310.jpg; **Taxon:** taxonConceptID: Grimpoteuthis gen. inc.; kingdom: Animalia; phylum: Mollusca; class: Cephalopoda; order: Octopoda; family: Opisthoteuthidae; genus: Grimpoteuthis; taxonRank: Genus; scientificNameAuthorship: Robson, 1932; **Location:** waterBody: Indian Ocean; stateProvince: Rodriguez Triple Junction; locality: RTJ; verbatimLocality: Cluster 5; maximumDepthInMeters: 2501; locationRemarks: RV Pelagia Cruise INDEX2018 Leg 2; geodeticDatum: WGS84; coordinateUncertaintyInMeters: 25; **Identification:** identifiedBy: Kathrin Bolstad; identificationRemarks: Identified only from imagery; identificationQualifier: gen. inc.; **Event:** eventDate: 2018-12-03; eventTime: 12:50:35 pm; year: 2018; fieldNumber: INDEX2018-82ROPOS; fieldNotes: 1.8°C, 34.7 ppt; **Record Level:** language: en; institutionCode: DZMB; datasetName: INDEX; basisOfRecord: Human Observation

##### Notes

Fig. 196

#### Oegopsida

d’Orbigny, 1845

127AE24A-D92E-506A-B712-88D6F6EDA8F2

#### Magnapinnidae

Vecchione & Young, 1998

54767869-4F3F-5E1E-942B-89058E7A9FCF

#### Magnapinna

Vecchione & Young, 1998

1F9AABBF-18C2-59B3-8CBD-F4B7B05E5D0D

#### Magnapinna sp. indet.

564224FA-36D2-579C-B81B-D20D193CBB55

##### Materials

- **Type status:** Other material. **Occurrence:** recordedBy: IFREMER; lifeStage: Adult; behavior: Swimming; occurrenceStatus: present; preparations: Imaged only; associatedMedia: 160119090731111_15_1080i copy.jpg; **Taxon:** taxonConceptID: Magnapinna sp. indet.; kingdom: Animalia; phylum: Mollusca; class: Cephalopoda; order: Oegopsida; family: Magnapinnidae; genus: Magnapinna; taxonRank: Genus; scientificNameAuthorship: Vecchione & Young, 1998; **Location:** waterBody: Indian Ocean; stateProvince: South East Indian Ridge; locality: Pelagia; verbatimLocality: Cluster 8; maximumDepthInMeters: 3664; locationRemarks: RV Pourqoui pas? Cruise INDEX2016 Leg 1; geodeticDatum: WGS84; coordinateUncertaintyInMeters: 37; **Identification:** identifiedBy: Kathrin Bolstad; identificationRemarks: Identified only from imagery; identificationQualifier: sp. indet.; **Event:** eventDate: 2016-01-19; eventTime: 9:07:31 am; year: 2016; fieldNumber: INDEX2016-16ROV; fieldNotes: 1.7°C, 34.7 ppt; **Record Level:** language: en; institutionCode: DZMB; datasetName: INDEX; basisOfRecord: Human Observation

##### Notes

Fig. 197

#### Gastropoda

Cuvier, 1795

4AB32B80-4D84-5AC9-8877-C38E8C9653E4

#### Caenogastropoda

Cox, 1960

1F0A0437-7D79-501C-8E89-3489D386E546

#### Abyssochrysoidea

Tomlin, 1927

783362FD-255E-5594-B664-0285CE7C5B5C

#### Abyssochrysoidea superfam. inc.

8E1F0D09-28FD-5622-87A3-633B0D39A1B2

##### Materials

- **Type status:** Other material. **Occurrence:** recordedBy: IFREMER; individualCount: 100; lifeStage: Adult; behavior: attached to hard substrates; occurrenceStatus: present; preparations: Imaged only; associatedMedia: 160121163921145_01_1080i Kopie.jpg; **Taxon:** taxonConceptID: Abyssochrysoidea superfam. inc.; kingdom: Animalia; phylum: Mollusca; class: Gastropoda; order: Caenogastropoda; taxonRank: Superfamily; scientificNameAuthorship: Tomlin, 1927; **Location:** waterBody: Indian Ocean; stateProvince: South East Indian Ridge; locality: Pelagia; verbatimLocality: Cluster 8; maximumDepthInMeters: 3685; locationRemarks: RV Pourqoui pas? Cruise INDEX2016 Leg 1; geodeticDatum: WGS84; coordinateUncertaintyInMeters: 37; **Identification:** identifiedBy: Leon Hoffman; identificationRemarks: Identified only from imagery; identificationQualifier: superfam. inc.; **Event:** eventDate: 2016-01-21; eventTime: 4:39:21 pm; year: 2016; fieldNumber: INDEX2016-20ROV; fieldNotes: 1.7°C, 34.7 ppt; **Record Level:** language: en; institutionCode: DZMB; datasetName: INDEX; basisOfRecord: Human Observation

##### Notes

Fig. 198

#### Cerithiopsidae

H. Adams & A. Adams, 1853

5B1D3DAF-458E-54F1-9429-165F42CF2609

#### Speculator

Waren & Bouchet, 2001

2562D797-4BA3-5BFD-8991-C4191C94BBA0

#### Speculator gen. inc.

0916D69F-EAB4-5FEE-81D4-A872D7DF113D

##### Materials

- **Type status:** Other material. **Occurrence:** recordedBy: ROPOS.COM; individualCount: 2; lifeStage: Adult; behavior: on sediment; occurrenceStatus: present; preparations: Imaged only; associatedMedia: R1905_00010.jpg; **Taxon:** taxonConceptID: Speculator gen. inc.; kingdom: Animalia; phylum: Mollusca; class: Gastropoda; order: Caenogastropoda; family: Cerithiopsidae; genus: Speculator; taxonRank: Genus; scientificNameAuthorship: Waren & Bouchet, 2001; **Location:** waterBody: Indian Ocean; stateProvince: Central Indian Ridge; locality: Vent site 1; verbatimLocality: Cluster 4; maximumDepthInMeters: 3083; locationRemarks: RV Pelagia Cruise INDEX2015 Leg 2; geodeticDatum: WGS84; coordinateUncertaintyInMeters: 30; **Identification:** identifiedBy: Leon Hoffman; identificationRemarks: Identified only from imagery; identificationQualifier: gen. inc.; **Event:** eventDate: 2015-11-27; eventTime: 9:11:52 am; year: 2015; fieldNumber: INDEX2015-37ROV; fieldNotes: 1.8°C, 34.7 ppt; **Record Level:** language: en; institutionCode: DZMB; datasetName: INDEX; basisOfRecord: Human Observation

##### Notes

Fig. 199

#### Provannidae

Waren & Ponder, 1991

A082499C-5DD2-5C5C-997E-E2A26047C3CD

#### Alviniconcha

Okutani & Ohta, 1988

69013ACB-1713-5852-8B38-FCCB13567E0F

#### Alviniconcha marisindica

Okutani, 2014

89EBE8BC-FBCB-50B5-A25E-34838EC5300F

##### Materials

- **Type status:** Other material. **Occurrence:** recordedBy: ROPOS.COM; individualCount: 100; lifeStage: Adult; behavior: on sulphides; occurrenceStatus: present; preparations: Imaged only; associatedMedia: R2097_00186.jpg; **Taxon:** taxonConceptID: Alviniconchamarisindica; scientificName: Alviniconchamarisindica; kingdom: Animalia; phylum: Mollusca; class: Gastropoda; order: Caenogastropoda; family: Provannidae; genus: Alviniconcha; taxonRank: Species; scientificNameAuthorship: Okutani, 2014; **Location:** waterBody: Indian Ocean; stateProvince: South East Indian Ridge; locality: Vent site 6; verbatimLocality: Cluster 12; maximumDepthInMeters: 2466; locationRemarks: RV Pelagia Cruise INDEX2018 Leg 2; geodeticDatum: WGS84; coordinateUncertaintyInMeters: 24; **Identification:** identifiedBy: Leon Hoffman; identificationRemarks: Identified only from imagery; **Event:** eventDate: 2018-11-25; eventTime: 9:48:47 am; year: 2018; fieldNumber: INDEX2018-67ROPOS; **Record Level:** language: en; institutionCode: DZMB; datasetName: INDEX; basisOfRecord: Human Observation

##### Notes

Fig. 200

#### Lepetellida

Mosakalev, 1971

A4FA6333-F580-522C-9597-EBA726848E1B

#### Lepetodrilidae

McLean, 1988

A0858DA9-E69E-5D2D-B013-F0B5C9B90B0D

#### Lepetodrilidae fam. inc.

C4D2E4CB-F667-5BB6-969E-4E483E351674

##### Materials

- **Type status:** Other material. **Occurrence:** recordedBy: ROPOS.COM; individualCount: 100; lifeStage: Adult; behavior: moving on active chimney; occurrenceStatus: present; preparations: Imaged only; associatedMedia: R2097_00079.jpg; **Taxon:** taxonConceptID: Lepetodrilidae fam. inc.; kingdom: Animalia; phylum: Mollusca; class: Gastropoda; order: Lepetellida; family: Lepetodrilidae; taxonRank: Family; scientificNameAuthorship: McLean, 1988; **Location:** waterBody: Indian Ocean; stateProvince: South East Indian Ridge; locality: Vent site 6; verbatimLocality: Cluster 12; maximumDepthInMeters: 2320; locationRemarks: RV Pelagia Cruise INDEX2018 Leg 2; geodeticDatum: WGS84; coordinateUncertaintyInMeters: 24; **Identification:** identifiedBy: Leon Hoffman; identificationRemarks: Identified only from imagery; identificationQualifier: fam. inc.; **Event:** eventDate: 2018-11-25; eventTime: 6:52:10 am; year: 2018; fieldNumber: INDEX2018-67ROPOS; **Record Level:** language: en; institutionCode: DZMB; datasetName: INDEX; basisOfRecord: Human Observation

##### Notes

Fig. 201

#### Lepetodrilus

McLean, 1988

D17C0BB5-B4D6-5BDE-AAB2-86D89E243A4F

#### Lepetodrilus gen. inc.

E584AA4A-BCC7-50C8-9825-E354868C1C7D

##### Materials

- **Type status:** Other material. **Occurrence:** recordedBy: IFREMER; individualCount: 100; lifeStage: Adult; behavior: moving on active chimney; occurrenceStatus: present; preparations: Imaged only; associatedMedia: 160119104217205_01_1080i copy.jpg; **Taxon:** taxonConceptID: Lepetodrilus gen. inc.; kingdom: Animalia; phylum: Mollusca; class: Gastropoda; order: Lepetellida; family: Lepetodrilidae; genus: Lepetodrilus; taxonRank: Genus; scientificNameAuthorship: McLean, 1988; **Location:** waterBody: Indian Ocean; stateProvince: South East Indian Ridge; locality: Pelagia; verbatimLocality: Cluster 8; maximumDepthInMeters: 3676; locationRemarks: RV Pourqoui pas? Cruise INDEX2016 Leg 1; geodeticDatum: WGS84; coordinateUncertaintyInMeters: 37; **Identification:** identifiedBy: Leon Hoffman; identificationRemarks: Identified only from imagery; identificationQualifier: gen. inc.; **Event:** eventDate: 2016-01-19; eventTime: 10:42:17 am; year: 2016; fieldNumber: INDEX2016-16ROV; fieldNotes: 1.7°C, 34.7 ppt; **Record Level:** language: en; institutionCode: DZMB; datasetName: INDEX; basisOfRecord: Human Observation

##### Notes

Fig. 202

#### Lepetodrilidae Lepetodrilus sp. indet.

FB770AE6-F915-58DB-AEFA-F5B2F3C74A72

##### Materials

- **Type status:** Other material. **Occurrence:** recordedBy: IFREMER; individualCount: 100; lifeStage: Adult; behavior: moving on active chimney; occurrenceStatus: present; preparations: DNA voucher and animal stored in 96% ethanol; associatedMedia: 160111080932A Kopie.jpg; associatedOccurrences: none; associatedSequences: COI; **Taxon:** taxonID: I16_28; scientificNameID: Lepetodrilus sp. 2 SBJ-2008; taxonConceptID: Lepetodrilidae Lepetodrilus sp. indet.; kingdom: Animalia; phylum: Mollusca; class: Gastropoda; order: Lepetellida; family: Lepetodrilidae; genus: Lepetodrilus; taxonRank: Genus; scientificNameAuthorship: McLean, 1988; **Location:** waterBody: Indian Ocean; stateProvince: Rodriguez Triple Junction; locality: Kairei; verbatimLocality: Cluster 5; maximumDepthInMeters: 2420; locationRemarks: RV Pourqoui pas? Cruise INDEX2016 Leg 1; decimalLatitude: -25.3204; decimalLongitude: 70.0404; geodeticDatum: WGS84; coordinateUncertaintyInMeters: 25; **Identification:** identifiedBy: Leon Hoffman; identificationRemarks: Identified by morphology and DNA of collected specimen; identificationQualifier: sp. indet.; **Event:** eventDate: 2016-01-11; eventTime: 8:09:32 am; year: 2016; fieldNumber: INDEX2016-06ROV; **Record Level:** language: en; institutionCode: DZMB; collectionCode: I16_6RO_BB_124; datasetName: INDEX; basisOfRecord: Human Observation

##### Notes

Fig. 203

#### Neogastropoda

Wenz, 1938

E9F3DB86-CED4-5EA2-ACDD-263A5A04AB9D

#### Raphitomidae

Bellardi, 1875

DF641169-68C5-54EC-A404-05888444A008

#### Phymorhynchus

Dall, 1908

7C2499B8-7AB7-5261-877B-42C966B1CDB2

#### Phymorhynchus sp. indet.

9078AF54-F93E-5781-BCE4-7C1E66ADEAC4

##### Materials

- **Type status:** Other material. **Occurrence:** recordedBy: ROPOS.COM; individualCount: 100; lifeStage: Adult; behavior: on sulphides/ basalt; occurrenceStatus: present; preparations: Imaged only; associatedMedia: R2098_00304.jpg; **Taxon:** taxonConceptID: Phymorhynchus sp. indet.; kingdom: Animalia; phylum: Mollusca; class: Gastropoda; order: Neogastropoda; family: Raphitomidae; genus: Phymorhynchus; taxonRank: Genus; scientificNameAuthorship: Dall, 1908; **Location:** waterBody: Indian Ocean; stateProvince: South East Indian Ridge; locality: Vent site 3; verbatimLocality: Cluster 12; maximumDepthInMeters: 2530; locationRemarks: RV Pelagia Cruise INDEX2018 Leg 2; geodeticDatum: WGS84; coordinateUncertaintyInMeters: 26; **Identification:** identifiedBy: Leon Hoffman; identificationRemarks: Identified only from imagery; identificationQualifier: sp. indet.; **Event:** eventDate: 2018-11-26; eventTime: 11:46:54 am; year: 2018; fieldNumber: INDEX2018-70ROPOS; fieldNotes: 1.8°C, 34.7 ppt; **Record Level:** language: en; institutionCode: DZMB; datasetName: INDEX; basisOfRecord: Human Observation

##### Notes

Fig. 204

#### Phymorhynchus sp. indet. (Egg capsules)

D485B50B-4F13-5C3C-AF63-5E2F89E3CA8B

##### Materials

- **Type status:** Other material. **Occurrence:** recordedBy: ROPOS.COM; individualCount: 100; lifeStage: Eggs; behavior: attached to basalt; occurrenceStatus: present; preparations: Imaged only; associatedMedia: R2098_00304.jpg; **Taxon:** taxonConceptID: Phymorhynchus sp. indet. (Egg capsules); kingdom: Animalia; phylum: Mollusca; class: Gastropoda; order: Neogastropoda; family: Raphitomidae; genus: Phymorhynchus; taxonRank: Genus; scientificNameAuthorship: Dall, 1908; **Location:** waterBody: Indian Ocean; stateProvince: South East Indian Ridge; locality: Vent site 3; verbatimLocality: Cluster 12; maximumDepthInMeters: 2530; locationRemarks: RV Pelagia Cruise INDEX2018 Leg 2; geodeticDatum: WGS84; coordinateUncertaintyInMeters: 26; **Identification:** identifiedBy: Terue C. Kihara, Klaas Gerdes; identificationRemarks: Identified only from imagery; identificationQualifier: sp. indet.; **Event:** eventDate: 2018-11-26; eventTime: 11:46:54 am; year: 2018; fieldNumber: INDEX2018-70ROPOS; fieldNotes: 1.8°C, 34.7 ppt; **Record Level:** language: en; institutionCode: DZMB; datasetName: INDEX; basisOfRecord: Human Observation

##### Notes

Fig. 205

#### Neomphalida

22F95336-4B90-56C2-B6F1-01C333936BEA

#### Melanodrymiidae

Salvini-Plawen & Steiner, 1995

2899C449-1D55-5458-A14F-37A350DDA2AE

#### Melanodrymiidae fam. inc.

0B7B145C-E84E-5C02-B9C4-9074499F74C8

##### Materials

- **Type status:** Other material. **Occurrence:** recordedBy: ROPOS.COM; individualCount: 100; lifeStage: Adult; behavior: on sulphides; occurrenceStatus: present; preparations: Imaged only; associatedMedia: R2105_00319.jpg; **Taxon:** taxonConceptID: Melanodrymiidae fam. inc.; kingdom: Animalia; phylum: Mollusca; class: Gastropoda; order: Neomphalida; family: Melanodrymiidae; taxonRank: Family; scientificNameAuthorship: Salvini-Plawen & Steiner, 1995; **Location:** waterBody: Indian Ocean; stateProvince: Rodriguez Triple Junction; locality: Vent site 4; verbatimLocality: Cluster 5; maximumDepthInMeters: 2652; locationRemarks: RV Pelagia Cruise INDEX2018 Leg 2; geodeticDatum: WGS84; coordinateUncertaintyInMeters: 26; **Identification:** identifiedBy: Leon Hoffman; identificationRemarks: Identified only from imagery; identificationQualifier: fam. inc.; **Event:** eventDate: 2018-12-09; eventTime: 9:36:47 am; year: 2018; fieldNumber: INDEX2018-95ROPOS; fieldNotes: 1.8°C, 34.7 ppt; **Record Level:** language: en; institutionCode: DZMB; datasetName: INDEX; basisOfRecord: Human Observation

##### Notes

Fig. 206

#### Peltospiridae

McLean, 1989

4EF57D11-6BCF-5399-AF83-A26538089B17

#### Chrysomallon

C. Chen, Linse, Copley & Rogers, 2015

6382F6CF-90BB-5FB2-B325-930DC805A011

#### Chrysomallon squamiferum

C. Chen, Linse, Copley & Rogers, 2015

C462858E-17D7-56C2-B93F-447E510FA5FD

##### Materials

- **Type status:** Other material. **Occurrence:** recordedBy: ROPOS.COM; individualCount: 100; lifeStage: Adult; behavior: moving on active chimney; occurrenceStatus: present; preparations: Imaged only; associatedMedia: R2094_01112.jpg; **Taxon:** taxonConceptID: Chrysomallonsquamiferum; scientificName: Chrysomallonsquamiferum; kingdom: Animalia; phylum: Mollusca; class: Gastropoda; order: Neomphalida; family: Peltospiridae; genus: Chrysomallon; taxonRank: Species; scientificNameAuthorship: C. Chen, Linse, Copley & Rogers, 2015; **Location:** waterBody: Indian Ocean; stateProvince: South East Indian Ridge; locality: Vent site 6; verbatimLocality: Cluster 12; maximumDepthInMeters: 2474; locationRemarks: RV Pelagia Cruise INDEX2018 Leg 2; geodeticDatum: WGS84; coordinateUncertaintyInMeters: 25; **Identification:** identifiedBy: Leon Hoffman; identificationRemarks: Identified only from imagery; **Event:** eventDate: 2018-11-22; eventTime: 9:14:14 am; year: 2018; fieldNumber: INDEX2018-61ROPOS; fieldNotes: 1.9°C, 34.7 ppt; **Record Level:** language: en; institutionCode: DZMB; datasetName: INDEX; basisOfRecord: Human Observation

##### Notes

Fig. 207

#### Scaphopoda

Bronn, 1862

BD21C21A-9F0B-58E1-822F-404742E142AD

#### Scaphopoda ord. indet.

DA1F4099-331B-5951-BD27-3389CEEEA2F1

##### Materials

- **Type status:** Other material. **Occurrence:** recordedBy: ROPOS.COM; individualCount: 1; lifeStage: Adult; behavior: on sediment; occurrenceStatus: present; preparations: Imaged only; associatedMedia: R2092_00433.jpg; **Taxon:** taxonConceptID: Scaphopoda ord. indet.; kingdom: Animalia; phylum: Mollusca; class: Scaphopoda; taxonRank: Class; scientificNameAuthorship: Bronn, 1862; **Location:** waterBody: Indian Ocean; stateProvince: South East Indian Ridge; locality: Vent site 6; verbatimLocality: Cluster 12; maximumDepthInMeters: 2510; locationRemarks: RV Pelagia Cruise INDEX2018 Leg 2; geodeticDatum: WGS84; coordinateUncertaintyInMeters: 25; **Identification:** identifiedBy: Andrew J. Gooday; identificationRemarks: Identified only from imagery; identificationQualifier: ord. indet.; **Event:** eventDate: 2018-11-20; eventTime: 7:48:13 am; year: 2018; fieldNumber: INDEX2018-57ROPOS; fieldNotes: 1.8°C, 34.7 ppt; **Record Level:** language: en; institutionCode: DZMB; datasetName: INDEX; basisOfRecord: Human Observation

##### Notes

Fig. 208

#### Solenogastres

Gegenbaur, 1878

403B162A-C719-577E-A5F6-1E7ECA716200

#### Solenogastres ord. indet.

B15E5604-0765-5791-A7A3-34A03D906922

##### Materials

- **Type status:** Other material. **Occurrence:** recordedBy: IFREMER; individualCount: 1; lifeStage: Adult; behavior: attached to active chimney; occurrenceStatus: present; preparations: Imaged only; associatedMedia: 160119171357176_15_1080i copy.jpg; **Taxon:** taxonConceptID: Solenogastres ord. indet.; kingdom: Animalia; phylum: Mollusca; class: Solenogastres; taxonRank: Class; scientificNameAuthorship: Gegenbaur, 1878; **Location:** waterBody: Indian Ocean; stateProvince: South East Indian Ridge; locality: Pelagia; verbatimLocality: Cluster 8; maximumDepthInMeters: 3671; locationRemarks: RV Pourqoui pas? Cruise INDEX2016 Leg 1; geodeticDatum: WGS84; coordinateUncertaintyInMeters: 37; **Identification:** identifiedBy: Terue C. Kihara, Klaas Gerdes; identificationRemarks: Identified only from imagery; identificationQualifier: ord. indet.; **Event:** eventDate: 2016-01-19; eventTime: 5:13:57 pm; year: 2016; fieldNumber: INDEX2016-16ROV; fieldNotes: 1.7°C, 34.7 ppt; **Record Level:** language: en; institutionCode: DZMB; datasetName: INDEX; basisOfRecord: Human Observation

##### Notes

Fig. 209

#### Nemertea

38826F06-7A9F-5BA5-858E-796D402CA504

#### Hoplonemertea

Hubrecht, 1879

2BEC7DC8-2403-5B8A-88BF-0FEBAC153051

#### Monostilifera

Brinkmann, 1917

DC7EDC3E-950F-5083-BAB1-F97CE0852CDF

#### Emplectonematidae

Bürger, 1904

51FE3A43-39C8-5150-AFAF-BEC65A2A24BE

#### Thermanemertes

Rogers, Gibson & Tunnicliffe, 1996

BC25A481-4169-5685-A9D5-B3FA63EA6DFF

#### Thermanemertes gen. inc.

E2247960-4DA6-5CF0-ABA3-DFBC9C933832

##### Materials

- **Type status:** Other material. **Occurrence:** recordedBy: IFREMER; individualCount: 100; lifeStage: Adult; behavior: moving on active chimney; occurrenceStatus: present; preparations: DNA voucher and animal stored in 96% ethanol; associatedMedia: 160111085640277_15_1080i Kopie.jpg; associatedOccurrences: none; associatedSequences: COI; **Taxon:** taxonID: I16_30, I16_31; scientificNameID: Eumonostilifera sp. 1; taxonConceptID: Thermanemertes gen. inc.; kingdom: Animalia; phylum: Nemertea; class: Hoplonemertea; order: Monostilifera; family: Emplectonematidae; genus: Thermanemertes; taxonRank: Genus; scientificNameAuthorship: Rogers, Gibson & Tunnicliffe, 1996; **Location:** waterBody: Indian Ocean; stateProvince: Rodriguez Triple Junction; locality: Kairei; verbatimLocality: Cluster 5; maximumDepthInMeters: 2420; locationRemarks: RV Pourqoui pas? Cruise INDEX2016 Leg 1; decimalLatitude: -25.3204; decimalLongitude: 70.0404; geodeticDatum: WGS84; coordinateUncertaintyInMeters: 25; **Identification:** identifiedBy: Jon L. Norenburg; identificationRemarks: Identified by morphology and DNA of collected specimen; identificationQualifier: gen. inc.; **Event:** eventDate: 2016-01-11; eventTime: 8:56:40 am; year: 2016; fieldNumber: INDEX2016-06ROV; **Record Level:** language: en; institutionCode: DZMB; collectionCode: I16_6RO_BB_118; datasetName: INDEX; basisOfRecord: Human Observation

##### Notes

Fig. 210

#### Platyhelminthes

Minot, 1876

624A0D25-5DB6-5635-A0E9-B128C71EBD83

#### Rhabditophora

Ehlers, 1985

88C96689-B20A-5058-BCD4-7263E9FDAEBC

#### Polycladida

Lang, 1884

9ADC9088-010A-57B8-86A8-EE026BBA5529

#### Polycladida fam. indet.

BF70AC0F-2F8C-5C01-B236-F6B4724118C3

##### Materials

- **Type status:** Other material. **Occurrence:** recordedBy: IFREMER; individualCount: 100; lifeStage: Adult; behavior: on base of active chimney; occurrenceStatus: present; preparations: DNA voucher and animal stored in 96% ethanol; associatedMedia: 160115211609738_01_1080i copy.jpg; associatedOccurrences: none; associatedSequences: COI; **Taxon:** taxonID: I16_162; scientificNameID: Polycladida sp. 1; taxonConceptID: Polycladida fam. indet.; kingdom: Animalia; phylum: Platyhelminthes; order: Polycladida; taxonRank: Order; scientificNameAuthorship: Lang, 1884; **Location:** waterBody: Indian Ocean; stateProvince: Rodriguez Triple Junction; locality: Kairei; verbatimLocality: Cluster 5; maximumDepthInMeters: 2420; locationRemarks: RV Pourqoui pas? Cruise INDEX2016 Leg 1; decimalLatitude: -25.3204; decimalLongitude: 70.0403; geodeticDatum: WGS84; coordinateUncertaintyInMeters: 24; **Identification:** identifiedBy: Terue C. Kihara, Klaas Gerdes; identificationRemarks: Identified by morphology and DNA of collected specimen; identificationQualifier: fam. indet.; **Event:** eventDate: 2016-01-15; eventTime: 9:16:09 pm; year: 2016; fieldNumber: INDEX2016-12ROV; **Record Level:** language: en; institutionCode: DZMB; collectionCode: I16_12RO_SG4_2; datasetName: INDEX; basisOfRecord: Human Observation

##### Notes

Fig. 211

#### Porifera

Grant, 1836

FE723435-6EA3-52A7-AC89-A764C094D314

#### Paleodictyon

Giuseppe Meneghini, 1850

BB1A4342-6C6F-5327-AA2D-A7FEB02E989A

#### Paleodictyon nodosum sp. inc.

Seilacher, 1977

EC6B8571-C2BD-5BDB-A920-41CF665960F2

##### Materials

- **Type status:** Other material. **Occurrence:** recordedBy: ROPOS.COM; individualCount: 21; lifeStage: Adult; behavior: in sediment; occurrenceStatus: present; preparations: Imaged only; associatedMedia: R2101_00115.jpg; **Taxon:** taxonConceptID: Paleodictyonnodosum sp. inc.; scientificName: Paleodictyonnodosum; kingdom: Animalia; phylum: Porifera; genus: Paleodictyon; taxonRank: Species; scientificNameAuthorship: Seilacher, 1977; **Location:** waterBody: Indian Ocean; stateProvince: South East Indian Ridge; locality: Vent site 5; verbatimLocality: Cluster 11; maximumDepthInMeters: 2907; locationRemarks: RV Pelagia Cruise INDEX2018 Leg 2; geodeticDatum: WGS84; coordinateUncertaintyInMeters: 29; **Identification:** identifiedBy: Terue C. Kihara, Klaas Gerdes; identificationRemarks: Identified only from imagery; identificationQualifier: sp. inc.; **Event:** eventDate: 2018-11-29; eventTime: 9:30:02 am; year: 2018; fieldNumber: INDEX2018-75ROPOS; fieldNotes: 1.7°C, 34.7 ppt; **Record Level:** language: en; institutionCode: DZMB; datasetName: INDEX; basisOfRecord: Human Observation

##### Notes

Fig. 212

#### Chromista

Cavelier Smith, 1981

1C7F8894-9865-50C3-BF75-DA169C2D59DF

#### Foraminifera

D’Orbigny, 1826

3A853836-BF13-59D2-8E6E-9F5D7EF45E09

#### Monothalamea

Haeckel, 1862 (as emended by Pawlowski et al. 2013)

6632C216-EF05-5659-820E-0DD6D81D97C8

#### Monothalamea ord. indet. (DZMB_2021_0080)

88A9F025-9FC1-5260-9B8C-577BFE00FCDF

##### Materials

- **Type status:** Other material. **Occurrence:** recordedBy: ROPOS.COM; individualCount: 1; lifeStage: Adult; behavior: attached to basalt; occurrenceStatus: present; preparations: Imaged only; associatedMedia: R2104_00027.jpg; **Taxon:** taxonConceptID: Monothalamea ord. indet. (DZMB_2021_0080); kingdom: Chromista; phylum: Foraminifera; class: Monothalamea; taxonRank: Class; scientificNameAuthorship: Haeckel, 1862 (as emended by Pawlowski et al., 2013); **Location:** waterBody: Indian Ocean; stateProvince: Rodriguez Triple Junction; locality: Vent site 4; verbatimLocality: Cluster 5; maximumDepthInMeters: 2628; locationRemarks: RV Pelagia Cruise INDEX2018 Leg 2; geodeticDatum: WGS84; coordinateUncertaintyInMeters: 25; **Identification:** identifiedBy: Andrew J. Gooday; identificationRemarks: Identified only from imagery - Organism most closely resembles Pelosina, but identification cannot be confirmed from photographs (Indeterminate arborescent foraminiferan); identificationQualifier: ord. indet.; **Event:** eventDate: 2018-12-04; eventTime: 5:50:29 am; year: 2018; fieldNumber: INDEX2018-85ROPOS; fieldNotes: 1.8°C, 34.7 ppt; **Record Level:** language: en; institutionCode: DZMB; datasetName: INDEX; basisOfRecord: Human Observation

##### Notes

Fig. 213

#### Monothalamea ord. indet. (DZMB_2021_0081)

67E164EF-D283-584B-8BC9-D0BE86A152D7

##### Materials

- **Type status:** Other material. **Occurrence:** recordedBy: BGR; individualCount: 1; lifeStage: Adult; behavior: on sediment; occurrenceStatus: present; preparations: Imaged only; associatedMedia: 44MFT Fotos 2013-15-3.jpg; **Taxon:** taxonConceptID: Monothalamea ord. indet. (DZMB_2021_0081); kingdom: Chromista; phylum: Foraminifera; class: Monothalamea; taxonRank: Class; scientificNameAuthorship: Haeckel, 1862 (as emended by Pawlowski et al. 2013); **Location:** waterBody: Indian Ocean; stateProvince: Central Indian Ridge; locality: Edmond; verbatimLocality: Cluster 4; maximumDepthInMeters: 3238; locationRemarks: FS Sonne Cruise INDEX2013 Leg 2; decimalLatitude: -23.8781; decimalLongitude: 69.6035; geodeticDatum: WGS84; coordinateUncertaintyInMeters: 33; **Identification:** identifiedBy: Andrew J. Gooday; identificationRemarks: Identified only from imagery - Similar to Plate-like morphotypes 10: Groups of curved plates from the ISA megafauna catalogue, but it is impossible to determine if they represent the same morphotype based on photographs (Curved plate-like morphotype); identificationQualifier: ord. indet.; **Event:** eventDate: 2013-12-10; eventTime: 7:28:37 pm; year: 2013; fieldNumber: INDEX2013-44MFT; fieldNotes: 1.8°C, 34.7 ppt; **Record Level:** language: en; institutionCode: DZMB; datasetName: INDEX; basisOfRecord: Human Observation

##### Notes

Fig. 214

#### Monothalamea ord. indet. (DZMB_2021_0082)

9FF0D4A9-DD6B-5CA8-BA48-43E612537D04

##### Materials

- **Type status:** Other material. **Occurrence:** recordedBy: BGR; individualCount: 1; lifeStage: Adult; behavior: attached to basalt; occurrenceStatus: present; preparations: Imaged only; associatedMedia: Axinellida sp_17MFT Fotos 2013-358-4.jpg; **Taxon:** taxonConceptID: Monothalamea ord. indet. (DZMB_2021_0082); kingdom: Chromista; phylum: Foraminifera; class: Monothalamea; taxonRank: Class; scientificNameAuthorship: Haeckel, 1862 (as emended by Pawlowski et al., 2013); **Location:** waterBody: Indian Ocean; stateProvince: Central Indian Ridge; locality: MESO; verbatimLocality: outside INDEX claim; maximumDepthInMeters: 2847; locationRemarks: FS Sonne Cruise INDEX2013 Leg 1; decimalLatitude: -23.3842; decimalLongitude: 69.2377; geodeticDatum: WGS84; coordinateUncertaintyInMeters: 28; **Identification:** identifiedBy: Andrew J. Gooday; identificationRemarks: Identified only from imagery - Similar to Plate-like morphotypes 7: radiating plates from the ISA megafauna catalogue (Plate-like morphotype); identificationQualifier: ord. indet.; **Event:** eventDate: 2013-11-25; eventTime: 5:27:56 am; year: 2013; fieldNumber: INDEX2013-17MFT; fieldNotes: 1.7°C, 34.7 ppt; **Record Level:** language: en; institutionCode: DZMB; datasetName: INDEX; basisOfRecord: Human Observation

##### Notes

Fig. 215

#### Astrorhizida

Lankester, 1885

75704A69-7375-506B-849C-9A44D650BAF2

#### Arboramminidae

Shires, Gooday & Jones, 1994

F2A82CAE-8A7C-59A9-9AE7-513600264F30

#### Luffammina

Kamenskaya, Bagirov & Simdianov, 2002

95E801E9-6282-5164-9338-DC4065F2AE55

#### Luffammina gen. inc.

4907280E-9145-5BF5-80B9-12509707DA75

##### Materials

- **Type status:** Other material. **Occurrence:** recordedBy: BGR/ GEOMAR; individualCount: 100; lifeStage: Adult; behavior: attached to basalt; occurrenceStatus: present; preparations: Imaged only; associatedMedia: 2013-12-14_11-52-16_Sonne_INDEX2013-2_055ROV08_Logo-2.jpg; **Taxon:** taxonConceptID: Luffammina gen. inc.; kingdom: Chromista; phylum: Foraminifera; class: Monothalamea; order: Astrorhizoida; family: Arboramminidae; genus: Luffammina; taxonRank: Genus; scientificNameAuthorship: Kamenskaya, Bagirov & Simdianov, 2002; **Location:** waterBody: Indian Ocean; stateProvince: Central Indian Ridge; locality: Edmond/ Vent site 7; verbatimLocality: Cluster 4; maximumDepthInMeters: 3245; locationRemarks: FS Sonne Cruise INDEX2013 Leg 2; geodeticDatum: WGS84; coordinateUncertaintyInMeters: 32; **Identification:** identifiedBy: Andrew J. Gooday; identificationRemarks: Identified only from imagery - The structures resemble Luffamina atlantica from the Rainbow area of Mid-Atlantic Ridge, but their identification as a foraminifera cannot be confirmed from the photograph; identificationQualifier: gen. inc.; **Event:** eventDate: 2013-12-14; eventTime: 11:52:16 am; year: 2013; fieldNumber: INDEX2013-55ROV; **Record Level:** language: en; institutionCode: DZMB; datasetName: INDEX; basisOfRecord: Human Observation

##### Notes

Fig. 216

#### Psamminidae

7A2D053F-3353-56DD-A46F-56C119CDA644

#### Psammina

Haeckel, 1889

8F1EACFB-5E66-52A2-9F81-27FCDB3C1D83

#### Psammina gen. inc. (DZMB_2021_0083)

BBBDCDE7-78A6-55A5-9DD9-2FC089E92E6A

##### Materials

- **Type status:** Other material. **Occurrence:** recordedBy: BGR; individualCount: 1; lifeStage: Adult; behavior: attached to basalt; occurrenceStatus: present; preparations: Imaged only; associatedMedia: 17MFT Fotos 2013-361-4.jpg; **Taxon:** taxonConceptID: Psammina gen. inc. (DZMB_2021_0083); kingdom: Chromista; phylum: Foraminifera; class: Monothalamea; family: Psamminidae; genus: Psammina; taxonRank: Genus; scientificNameAuthorship: Haeckel, 1889; **Location:** waterBody: Indian Ocean; stateProvince: Central Indian Ridge; locality: MESO; verbatimLocality: outside INDEX claim; maximumDepthInMeters: 2865; locationRemarks: FS Sonne Cruise INDEX2013 Leg 1; decimalLatitude: -23.3820; decimalLongitude: 69.2365; geodeticDatum: WGS84; coordinateUncertaintyInMeters: 28; **Identification:** identifiedBy: Andrew J. Gooday; identificationRemarks: Identified only from imagery - it is impossible to confirm the generic identification from photographs; identificationQualifier: gen. inc.; **Event:** eventDate: 2013-11-25; eventTime: 6:01:42 am; year: 2013; fieldNumber: INDEX2013-17MFT; fieldNotes: 1.7°C, 34.7 ppt; **Record Level:** language: en; institutionCode: DZMB; datasetName: INDEX; basisOfRecord: Human Observation

##### Notes

Fig. 217

#### Psammina gen. inc. (DZMB_2021_0084)

6DE4D348-E109-5108-87EF-FCC3A7B2C330

##### Materials

- **Type status:** Other material. **Occurrence:** recordedBy: BGR; individualCount: 1; lifeStage: Adult; behavior: attached to basalt; occurrenceStatus: present; preparations: Imaged only; associatedMedia: IMG_2268.jpg; **Taxon:** taxonConceptID: Psammina gen. inc. (DZMB_2021_0084); kingdom: Chromista; phylum: Foraminifera; class: Monothalamea; family: Psamminidae; genus: Psammina; taxonRank: Genus; scientificNameAuthorship: Haeckel, 1889; **Location:** waterBody: Indian Ocean; stateProvince: South East Indian Ridge; locality: SEIR; verbatimLocality: Cluster 12; locationRemarks: FS Sonne Cruise INDEX2017 Leg 1; decimalLatitude: -27.7060; decimalLongitude: 73.7330; geodeticDatum: WGS84; coordinateUncertaintyInMeters: 34; **Identification:** identifiedBy: Andrew J. Gooday; identificationRemarks: Identified only from imagery - it is impossible to confirm the generic identification from photographs; identificationQualifier: gen. inc.; **Event:** eventDate: 2017-09-19; eventTime: 3:03:43 pm; year: 2017; fieldNumber: INDEX2017-74STR; fieldNotes: 1.7°C, 34.7 ppt; **Record Level:** language: en; institutionCode: DZMB; datasetName: INDEX; basisOfRecord: Human Observation

##### Notes

Fig. 218

#### Stannomidae

Haeckel, 1889

9D9EFC20-2E91-5099-9BB4-A5F11879B973

#### Stannoma

Haeckel, 1889

78FF1B21-2C19-5756-805D-906523235A79

#### Stannoma gen. inc.

F7CE7C64-DFF7-5126-991B-0E3CD59B1B0D

##### Materials

- **Type status:** Other material. **Occurrence:** recordedBy: BGR; individualCount: 1; lifeStage: Adult; behavior: attached to basalt; occurrenceStatus: present; preparations: Imaged only; associatedMedia: 44MFT Fotos 2013-452-2.jpg; **Taxon:** taxonConceptID: Stannoma gen. inc.; kingdom: Chromista; phylum: Foraminifera; class: Monothalamea; family: Stannomidae; genus: Stannoma; taxonRank: Genus; scientificNameAuthorship: Haeckel, 1889; **Location:** waterBody: Indian Ocean; stateProvince: Central Indian Ridge; locality: Edmond; verbatimLocality: Cluster 4; maximumDepthInMeters: 3332; locationRemarks: FS Sonne Cruise INDEX2013 Leg 2; decimalLatitude: -23.8788; decimalLongitude: 69.6000; geodeticDatum: WGS84; coordinateUncertaintyInMeters: 33; **Identification:** identifiedBy: Andrew J. Gooday; identificationRemarks: Identified only from imagery - it is impossible to confirm the generic identification from photographs; identificationQualifier: gen. inc.; **Event:** eventDate: 2013-12-10; eventTime: 12:20:20 am; year: 2013; fieldNumber: INDEX2013-44MFT; fieldNotes: 1.8°C, 34.7 ppt; **Record Level:** language: en; institutionCode: DZMB; datasetName: INDEX; basisOfRecord: Human Observation

##### Notes

Fig. 219

## Discussion

Despite the majority of the 218 taxa identified solely on imagery and physical samples existing for a considerably lower number, this is the first image atlas of the deep-sea benthic megafauna for the GLA, covering the southern CIR and northern SEIR in the Indian Ocean. Specifically, the atlas consists of the first collection of imaged taxon occurrences within active hydrothermal vents and their periphery, inactive vent sites and the non-vent areas in a region potentially exposed to mining and, thus, presents valuable biological baseline information.

Amongst the 218 taxa, the phylum Cnidaria are represented by the most taxa (77), followed by the phyla Echinodermata (48), Chordata (30), Arthropoda (22), Mollusca (17), Annelida (9) and Bryozoa (4). The phyla Hemichordata, Nemertea, Platyhelminthes and Porifera are each represented by one taxon. However, the latter phylum was excluded from this catalogue as noted in the Methods section, but has a considerably higher number of taxa (Table 5).

In the Kingdom Chromista, protists, belonging to phylum Foraminifera, contributed seven taxa that could be distinguished, based on imagery. Some are tentatively identified to genus level, but these identifications cannot be confirmed from photographs alone. The identification of xenophyophores from photographs is particularly problematic. Nevertheless, these protistan taxa were consistently identified in all years and by all sampling gear.

### Active hydrothermal vent fields

A total of 93 megafaunal taxa were recognised from the active hydrothermal vents, based on imagery (Table 6). This number includes non-vent species occurring in close proximity to active hydrothermal active vent fields without directly depending on-, or being influenced by, fluid discharge. Of all visually observed taxa, 18 could be confirmed taxonomically or based on molecular methods via sampling in Edmond (CIR, 7 taxa), Kairei (RTJ, 15 taxa), vent site 1 (CIR, 8 taxa), vent site 4 (RTJ, 2 taxa), vent site 5 (SEIR, 2 taxa), vent site 6 (SEIR, 2 taxa) and Pelagia (SEIR, 9 taxa) (vent field data and names not published). A single *Freyella* gen. inc. was observed within vent site 4, but this taxon is generally considered a non-vent species.

Of the total of 93 taxa found within and in close proximity to hydrothermal vents, 29 identified megafaunal taxa were considered endemic at active vent fields. This number is comparable to the total of 46 megafaunal taxa identified from imagery or physical sampling at Dodo, Solitaire, Edmond and Kairei vent fields, stretching along the CIR and at Longqi and Tiancheng along the South West Indian Ridge (SWIR) (Watanabe and Beedessee 2015, Sun et al. 2020). In particular, four species were identified at the Dodo vent field; 18 taxa were initially identified within Solitaire (Nakamura et al. 2012) and subsequent physical sampling and taxonomic work revealed 22 taxa (Watanabe and Beedessee 2015). At Longqi, 21 were initially identified through observation and sampling (Copley et al. 2016) and 32 taxa have since been recognised (Zhou et al. 2018). A total of 23 species and morphotypes were recognised at Tiancheng vent field, including the active venting area and the periphery of the vent field (Sun et al. 2020). At Kairei vent field, 26 taxa were reported when the vent field was discovered (Hashimoto et al. 2001), with a subsequent increase to 34 known taxa (Watanabe and Beedessee 2015). For the Edmond vent field, Watanabe and Beedessee (2015) described six taxa.

### Inactive hydrothermal vent field

We recognised a total of 69 taxa at inactive vent fields and inactive parts of active vent fields (Table 7), of which 37 are shared species with non-vent areas and 30 with active vent fields. Twenty two taxa were found exclusively within or close to inactive vent fields, 15 of these within inactive sites.

The few studies focusing on megafauna at inactive vent fields found that most taxa are known from other hard substrates and were not endemic or strictly dependent on inactive hydrothermal areas (Boschen et al. 2013, Boschen et al. 2016). Nevertheless, the faunal composition and abundance of these non-vent taxa were different within inactive areas compared to those observed in non-vent areas on hard substrates.

Some species were found exclusively at inactive vents, including two limpet species from the East Pacific Rise (McLean 1990) and one polynoid polychaeta from the Galapagos Spreading Center (Pettibone 1989). At the SWIR, within the Longqi hydrothermal vent field, an unknown ampharetid polychaete was sampled that Zhou et al. (2018) suggested to be adapted to inactive sites.

In the Pacific Ocean aggregations of non-vent fauna, such as solitary tunicates, brisingid sea stars, crinoids, sponges, anemones and brachiopods are found on inactive hydrothermal sulphides at Gorda Ridge (Van Dover et al. 1990). Observations from inactive sites close to Rumble II West Seamount off New Zealand revealed aggregations of comatulid crinoids, actiniarian anemones, sponges, ascidians, brachiopods and several coral species (Boschen et al. 2016). Similar suspension-feeding communities in comparably high abundance are reported from inactive sulphides at the Manus Basin (Galkin 1997, Sen et al. 2014) and Brothers Seamount (Boschen et al. 2015).

On inactive chimney complexes at Longqi, Zhou et al. (2018) reported occasional occurrences of *Munidopsis*-type galatheids; these inactive chimneys are relatively close to active vent sites, which might influence their faunal composition. *Munidopsis* species are also present within inactive sites in the INDEX area that lacks any recent hydrothermal activity.

### Non-vent area

A total of 134 megafauna taxa were identified in the non-vent areas (Table 8), of which many were observed within inactive vents and in the periphery of active vents. Many of these shared taxa represent mobile individuals, especially of the phylum Chordata.

Information on the benthic deep-sea megafauna of the Indian Ocean from both samples and imagery are rather scarce (Ingole and Koslow 2005, Sautya et al. 2011) and often focuses on the shelf (Hunter et al. 2011). Sautya et al. (2011) identified 58 megafaunal taxa using video transects and TV-grab samples from the Andaman Sea, a back-arc basin in the northern part of the Indian Ocean, although the highest diversity and density was observed on the flanks or summit of seamounts and only seven were found in the Andaman Basin on fine sediments (2,876 –2,917 m).

These seven taxa were observed at similar depths to those recorded here from the CIR and SEIR area, all on soft substrates (Sautya et al. 2011). The imagery samples from the seamounts, representing hard substrates, were taken at a maximum depth of 1,424 m (Sautya et al. 2011), much shallower and more influenced by surface primary production compared to the deeper and oligotrophic study area (Harms et al. 2019).

Ingole and Koslow (2005) recognised 38 megafaunal taxa from video transects and sampling in the Central Ocean Basin. Other studies in the Indian Ocean focused on macrofauna (Ingole 2003) and responses of macrofauna to disturbance (Ingole et al. 2001, Jones et al. 2017).

### Megafauna in the German licence area

The GLA covers three regions; the southern CIR, the RTJ and the northern SEIR, a distance spanning 1000 km from the northern to the southern border. Several taxa are widespread throughout the INDEX area and others have restricted distribution patterns (Tables 6, 7, 8). For the active vent fields, 19 species occur on the CIR, SEIR and RTJ, including the shrimp *Rimicariskairei*, the bristle worm *Archinomejasoni*, the fish *Pachycaraangeloi* and the mussel *Bathymodiolusseptemdierum* (Table 6). Fifty taxa were found only in one region, including the bristle worm *Branchipolynoe* gen. inc. on the CIR, the sea spider Pantopoda ord. inc. on the SEIR and the ribbon worm *Thermanemertes* gen. inc. at the RTJ (Table 6). The remaining 26 taxa were observed in two of the three regions, ten of them on the CIR and SEIR, but not within the RTJ in between these two regions, thereby probably reflecting the sampling effort.

The widespread distribution throughout the INDEX area of many typical active vent field taxa found in this study confirms the Indian Ocean as a standalone biogeographic province (Van Dover et al. 2001, Zhou et al. 2018, Sun et al. 2020). The majority of spatially-restricted taxa were either smaller taxa, as reported for polychaetes from Longqi (Copley et al. 2016, Zhou et al. 2018) or were occasional vent field residents also found in more distant non-vent areas (compare Table 6 and Table 8 for shared taxa). These taxa probably contribute to the small-scale differences described for Indian Ocean vent fields (Copley et al. 2016, Zhou et al. 2018, Sun et al. 2020) and can also be confirmed for the active vent fields within the INDEX area.

At inactive hydrothermal vent fields, the majority of taxa were shared with active vent fields and non-vent areas and only 22 of the 69 taxa were observed only within - or close to - inactive areas. Fifteen taxa were observed exclusively within inactive areas on sulphides. Of the 22 taxa observed at inactive sites, 19 showed a restricted occurrence in one region, including the isopod Munnopsidae fam. inc. (DZMB_2021_0007) at the RTJ, the Bryozoan Cheilostomatida fam. indet. (DZMB_2021_0009) on the SEIR and the fish *Histiobranchus* gen. inc. on the CIR; the remaining three taxa were restricted to two regions each (Table 7). Fifteen taxa showed a spatial distribution across two regions and only Synaphobranchidae gen. indet. showed a widespread distribution in all three regions, but this taxon is not restricted to inactive vent fields and was also observed in active vent fields. The taxa at inactive areas show highly localised distribution patterns in low to medium abundance (Table 7). In addition and in contrast to the high number of shared taxa at active vent fields in this study and beyond the INDEX area (Breusing et al. 2015, Sun et al. 2020), the inactive vent fields had a considerably lower number of shared taxa between inactive sites and no widespread distribution of taxa across the INDEX area.

The non-vent area showed the highest species diversity in medium to low abundance with a high number of locally-restricted taxa (Table 8). Eighty-two taxa occurred in one region, 39 in two regions and only 13 taxa were observed throughout the INDEX area, the majority in medium to low abundance (Table 8).

In the Indian Ocean, three additional licence areas for polymetallic sulphides have been issued to China on the SWIR, India on the southern CIR and SWIR and Korea on the CIR, with a number of shared species between the licence areas and hydrothermal vent fields outside exploration claims (Nakamura et al. 2012, Zhou et al. 2018, Sun et al. 2020). For example, the gastropod *Chrysomallonsquamiferum* has been found in both the Chinese and the German exploration claim areas in addition to the Solitaire vent field outside exploration areas (Chen et al. 2015). The low connectivity of the known populations at these three vent sites in the Indian Ocean, of which two are within national licence claim areas, has led to classification of this taxon as endangered on the IUCN Red List (Sigwart et al. 2019). The IUCN Red List might, therefore, be used to draw attention to vulnerable deep-sea habitats and serve as a basis for protecting them (Sigwart et al. 2019). To clarify the population connectivity of *C.squamiferum* and other locally-restricted species, it is necessary to conduct biodiversity assessments in additional hydrothermal vent areas.

Other taxa, such as *Rimicariskairei*, show a greater connectivity and a more widespread distribution across spatially-separated vent fields in this yet undisturbed environment (Hashimoto et al. 2001, Zhou et al. 2018, Gerdes et al. 2019).

Although potential mining activities are focusing on inactive hydrothermal vents, they could affect nearby active hydrothermal vents and the surrounding non-vent area (Levin et al. 2016, Miller et al. 2018, Van Dover 2019).

Mitigation strategies to avoid 'serious harm' to the environment include Environmental Impact Assessment studies (EIA), which require baseline studies for time series and monitoring to understand and explain succession, resilience and the recovery potential of the fauna against anthropogenic disturbances (Gollner et al. 2017, Van Dover 2019).

The impact of mining activities for inactive hydrothermal vent sites is far from understood due to a lack of qualitative and quantitative studies as pointed out in several reviews addressing the potential consequences of mining-related disturbances (Van Dover 2014, Miller et al. 2018, Van Dover 2019). For the non-vent areas in the vicinity of hydrothermal vent fields, mining effects are even less studied and require proactive research (Van Dover 2014).

This megafauna catalogue is a valuable baseline study that offers an impression of the diversity present within the GLA, which can serve as a basis for monitoring the deep-sea benthic megafauna in the context of potential SMS mining activities. A drawback of this fauna catalogue is that the occurrences of taxa are based chiefly on imagery alone; additional physical samples are needed for taxonomic and molecular confirmation.

## Supplementary Material

AF888768-C139-5536-B519-975B340F1B32 10.3897/BDJ.9.e69955.suppl1 Supplementary material 1 Megafauna table with all occurrences of the German exploration licence area for seafloor massive sulphides along the Central and South East Indian Ridge (Indian Ocean) Data type plain-text table with tab-separated fields in UTF-8 encoding and plain line endings (Unix LF) File: oo_553002.txt https://binary.pensoft.net/file/553002 Gerdes KH, Martínez Arbizu P, Kuhn T, Schwarz-Schampera U, Mah C, Norenburg J, Linley TD, Shalaeva K, Macpherson E, Gordon D, Stöhr S, Messing CG, Bober S, Guggolz TM, Christodoulou M, Gebruk A, Kremenetskaia A, Kroh A, Sanamyan K, Bolstad K, Hoffman L, Gooday AJ, Molodtsova T, Kihara TC

## Figures and Tables

**Figure 3.**
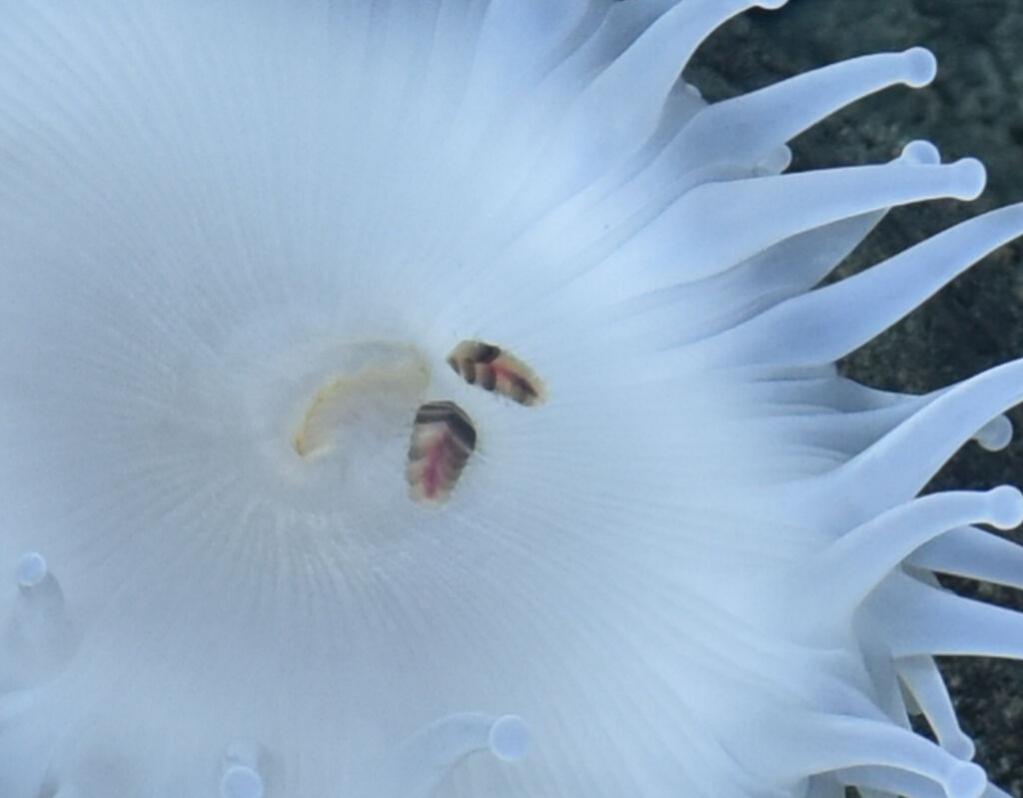
Commensal Polynoidae gen. indet. in situ on Actiniaria anemone in Cluster 5 of the INDEX area. Image corresponds with the data (Image attribution: BGR).

**Figure 4.**
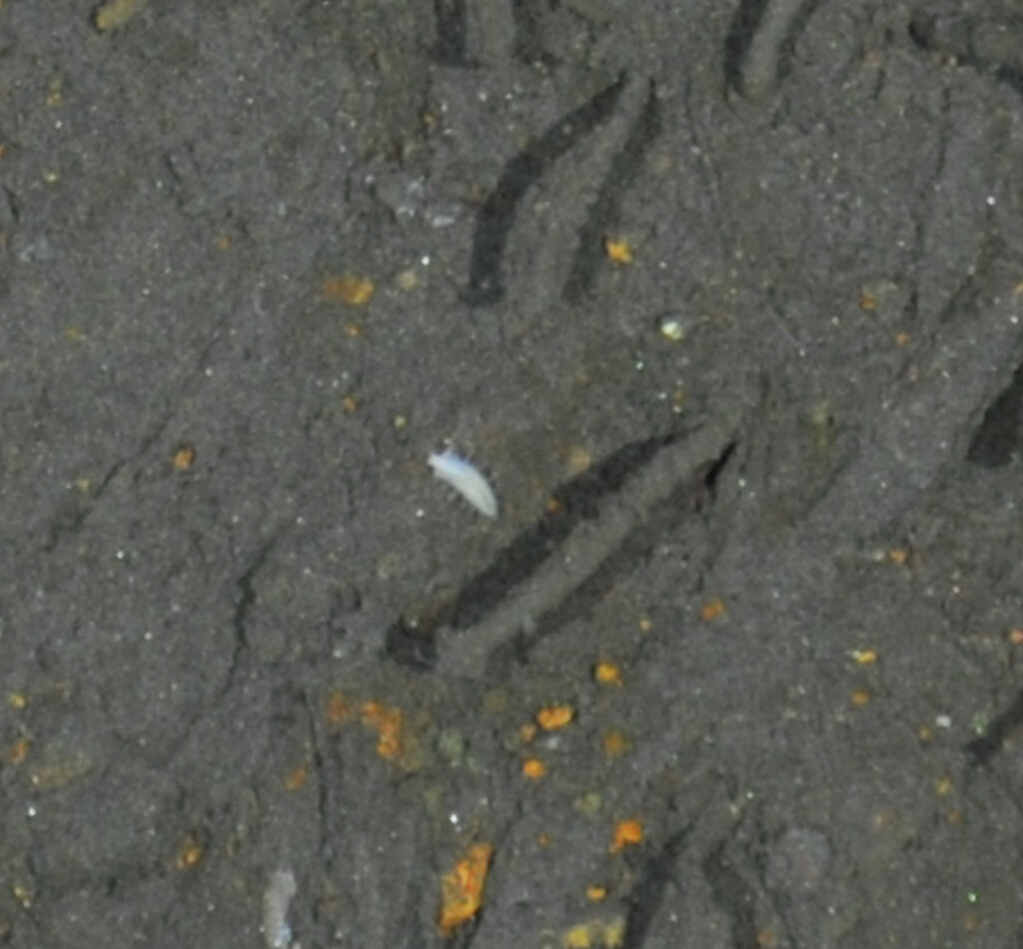
*Branchipolynoe* gen. inc. in situ within the Edmond hydrothermal vent field in Cluster 4 of the INDEX area. Image corresponds with the data (Image attribution: BGR).

**Figure 5.**
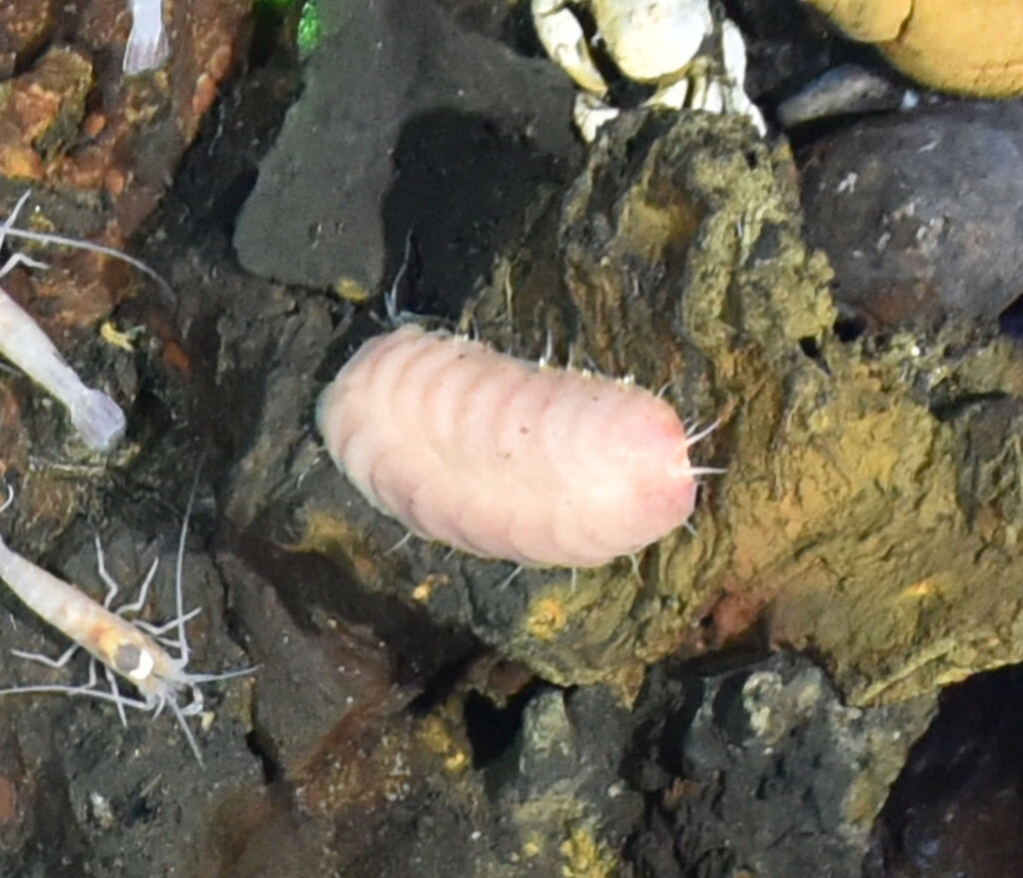
*Lepidonotopodium* gen. inc. (DZMB_2021_0001) in situ within the vent site 4 hydrothermal vent field in Cluster 5 of the INDEX area. Image corresponds with the data (Image attribution: BGR).

**Figure 6.**
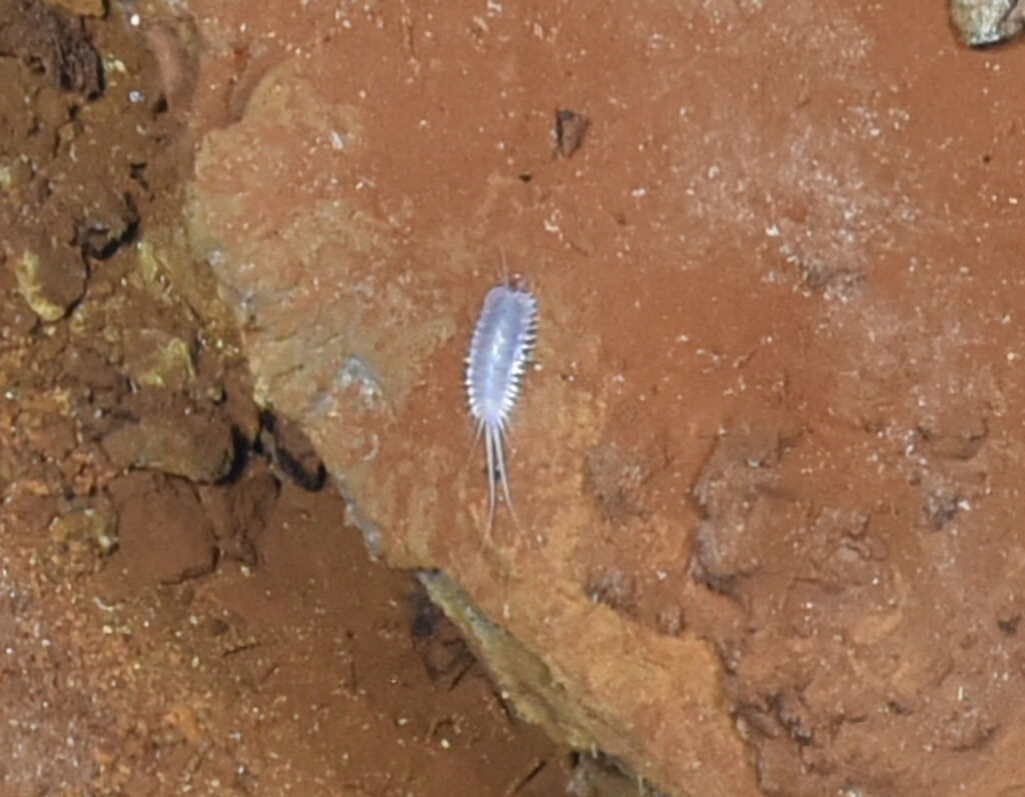
*Lepidonotopodium* gen. inc. (DZMB_2021_0002) in situ within the vent site 5 hydrothermal vent field in Cluster 11 of the INDEX area. Image corresponds with the data (Image attribution: BGR).

**Figure 7.**
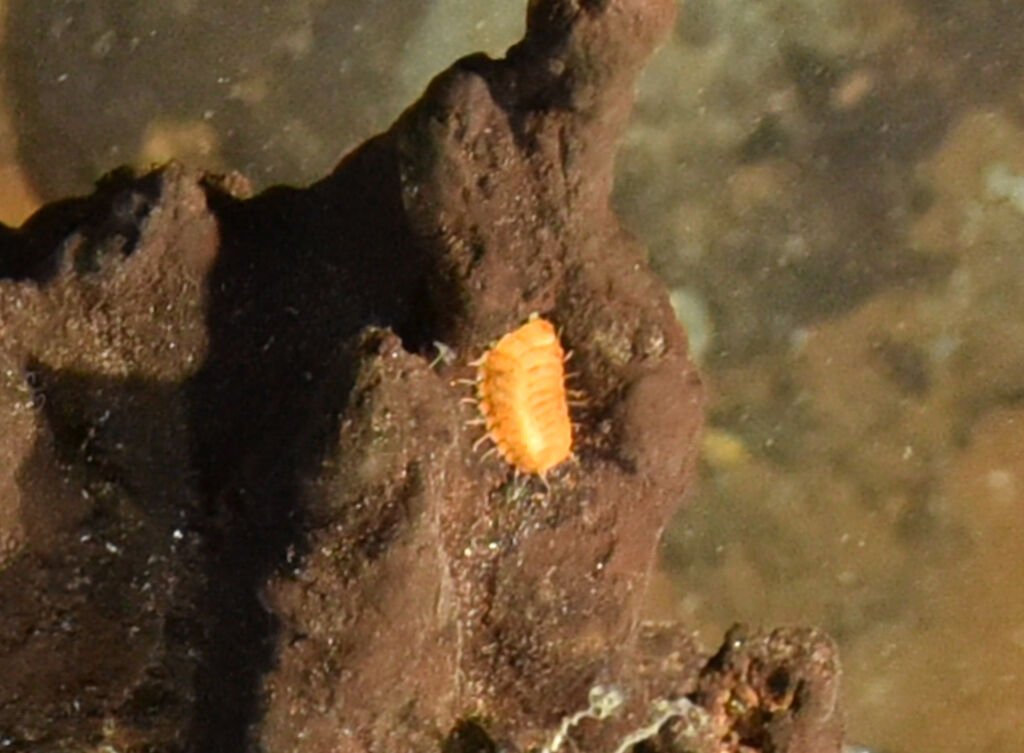
*Lepidonotopodium* gen. inc. (DZMB_2021_0003) in situ within the vent site 6 hydrothermal vent field in Cluster 12 of the INDEX area. Image corresponds with the data (Image attribution: BGR).

**Figure 8.**
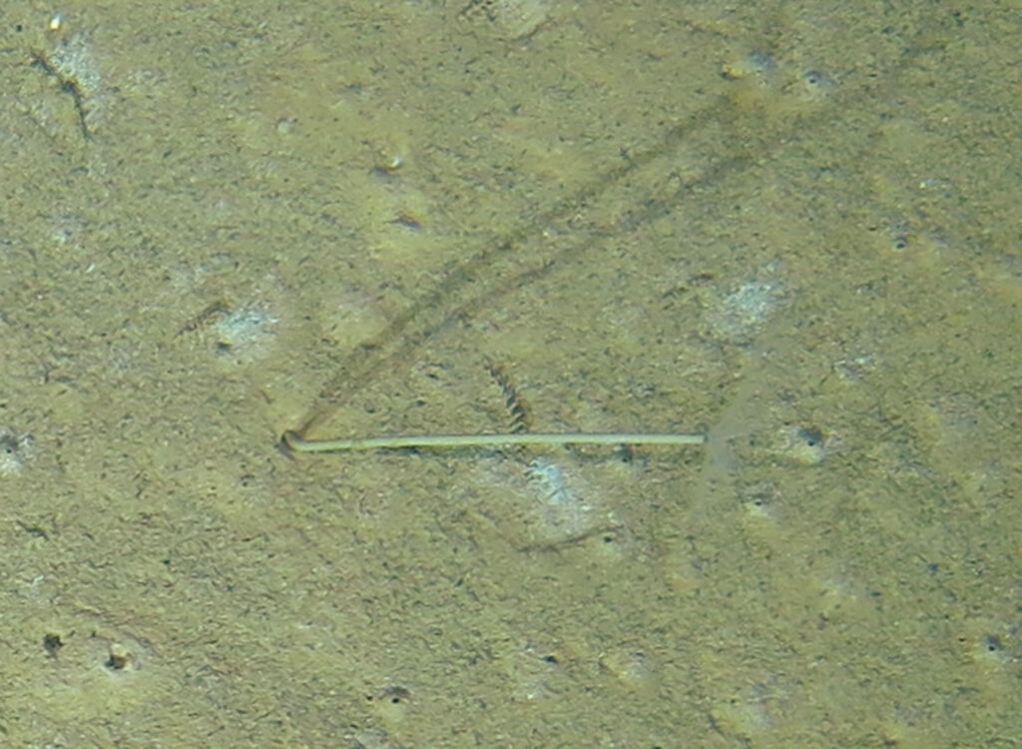
Sabellidae gen. indet. in situ on the seafloor in Cluster 11 of the INDEX area. Image corresponds with the data (Image attribution: BGR).

**Figure 9.**
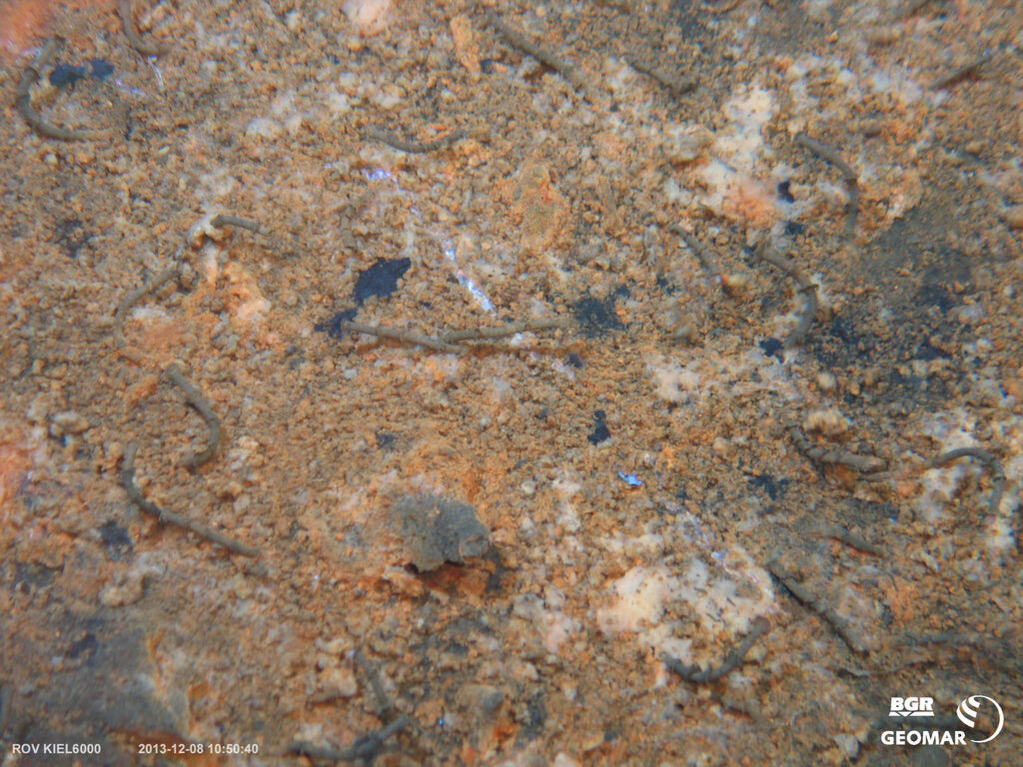
*Oasisia* gen. inc. in situ within the Edmond hydrothermal vent field in Cluster 4 of the INDEX area. Image corresponds with the data (Image attribution: BGR and GEOMAR).

**Figure 10.**
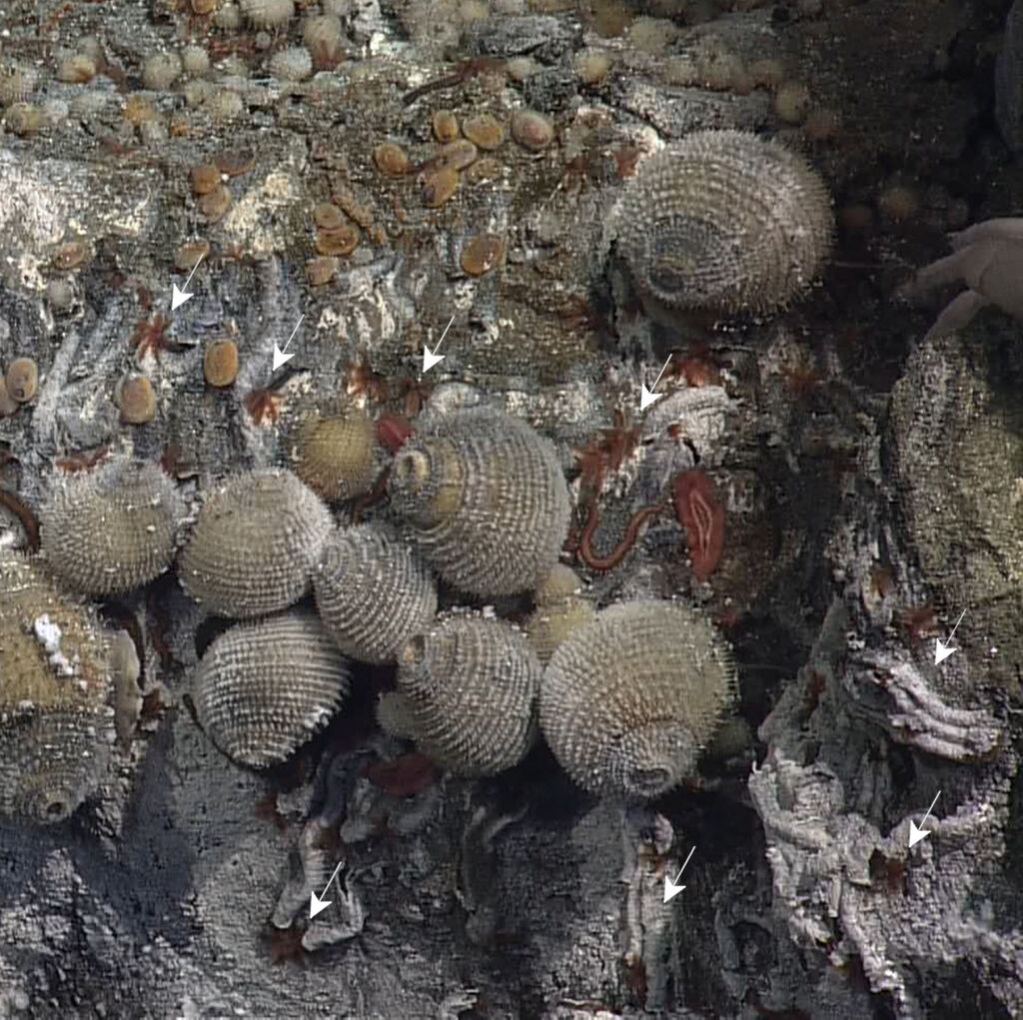
*Alvinella* gen. inc. in situ (white arrow) within the vent site 6 hydrothermal vent field in Cluster 12 of the INDEX area. Image corresponds with the data (Image attribution: BGR).

**Figure 11.**
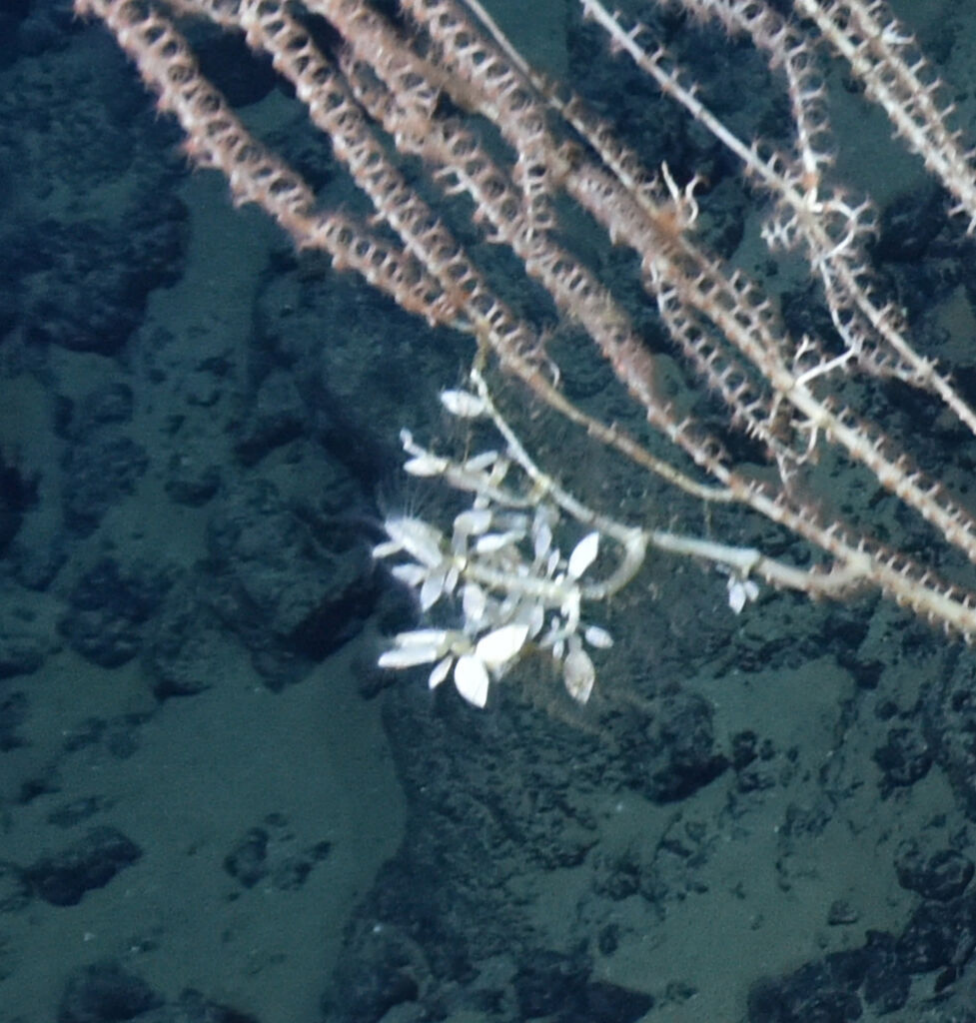
*Glyptelasma* gen. inc. in situ within the vent site 6 hydrothermal vent field in Cluster 12 of the INDEX area. Image corresponds with the data (Image attribution: BGR).

**Figure 12.**
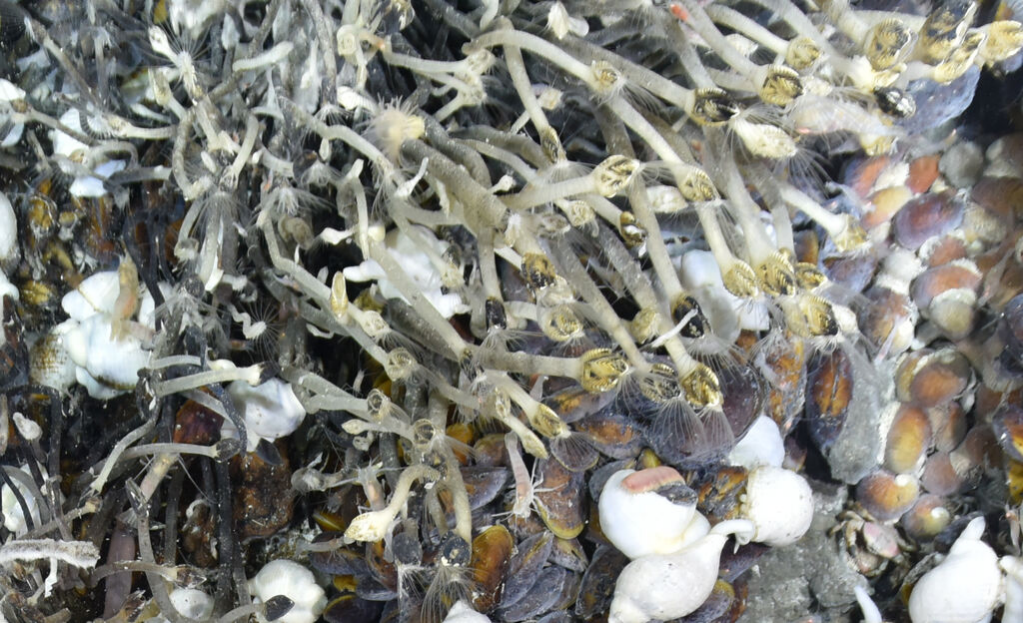
*Neolepasmarisindica* sp. inc. in situ within the vent site 6 hydrothermal vent field in Cluster 12 of the INDEX area. Image corresponds with the data (Image attribution: BGR).

**Figure 13.**
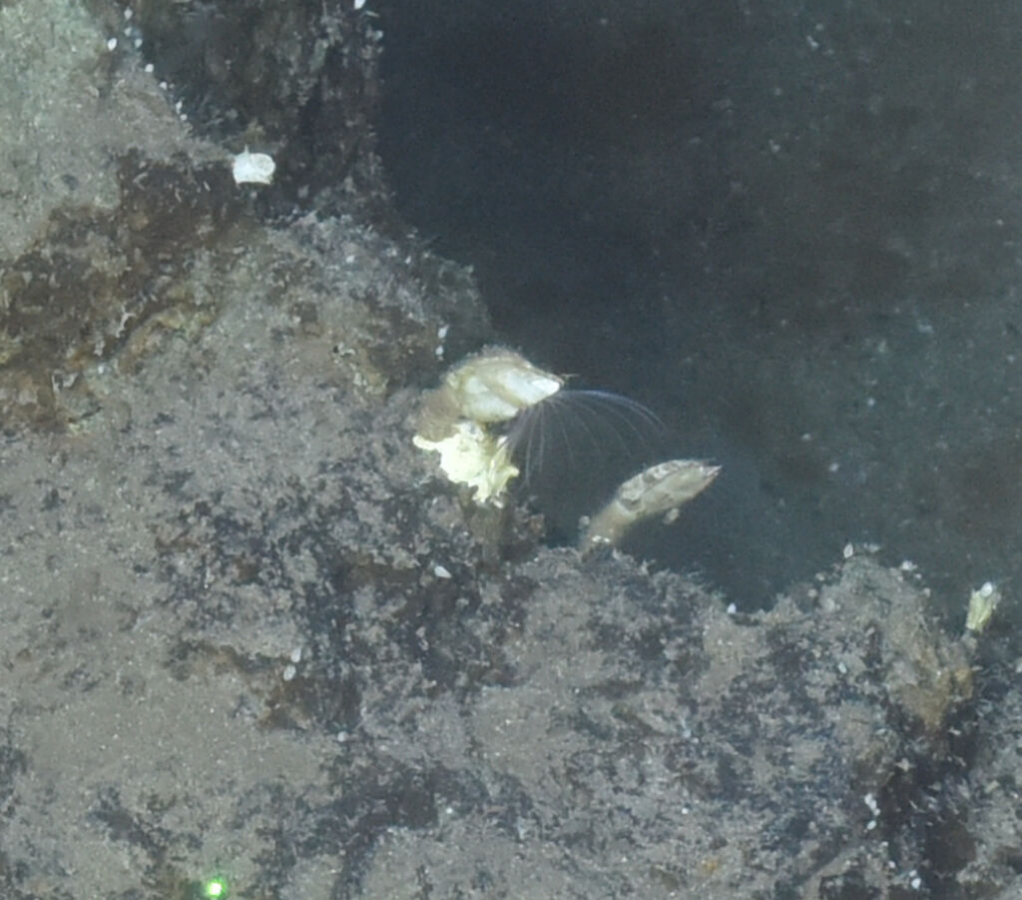
*Regioscalpellumregium* sp. inc. in situ within the vent site 6 hydrothermal vent field in Cluster 12 of the INDEX area. Image corresponds with the data (Image attribution: BGR).

**Figure 14.**
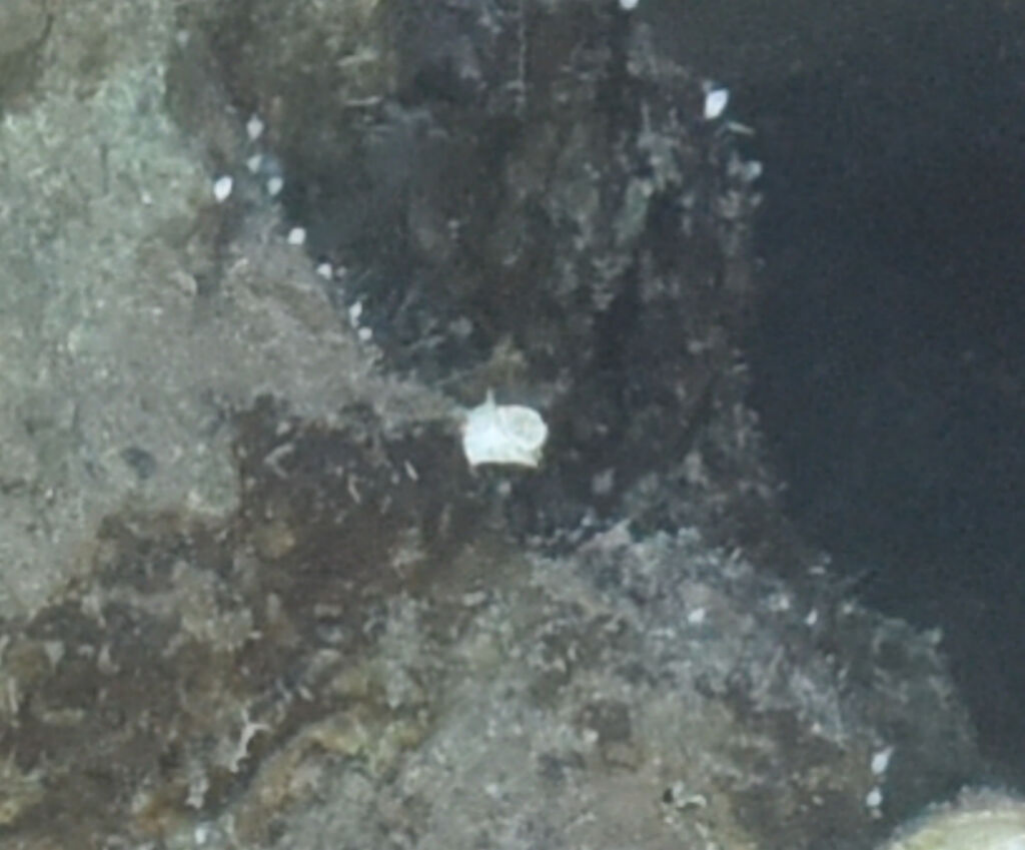
Verrucidae fam. inc. in situ within the vent site 6 hydrothermal vent field in Cluster 12 of the INDEX area. Image corresponds with the data (Image attribution: BGR).

**Figure 15.**
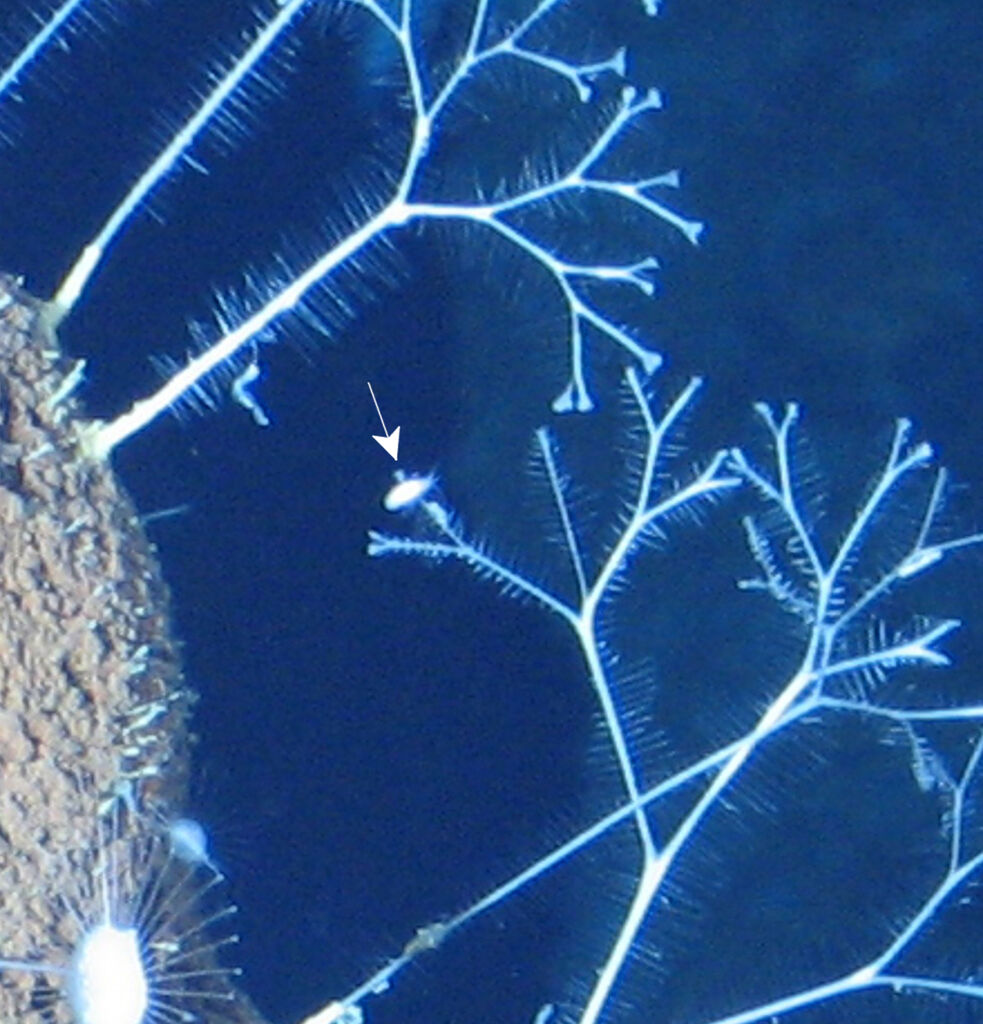
Amphipoda ord. inc. in situ within the Kairei hydrothermal vent field in Cluster 5 of the INDEX area. Image corresponds with the data (Image attribution: BGR).

**Figure 16.**
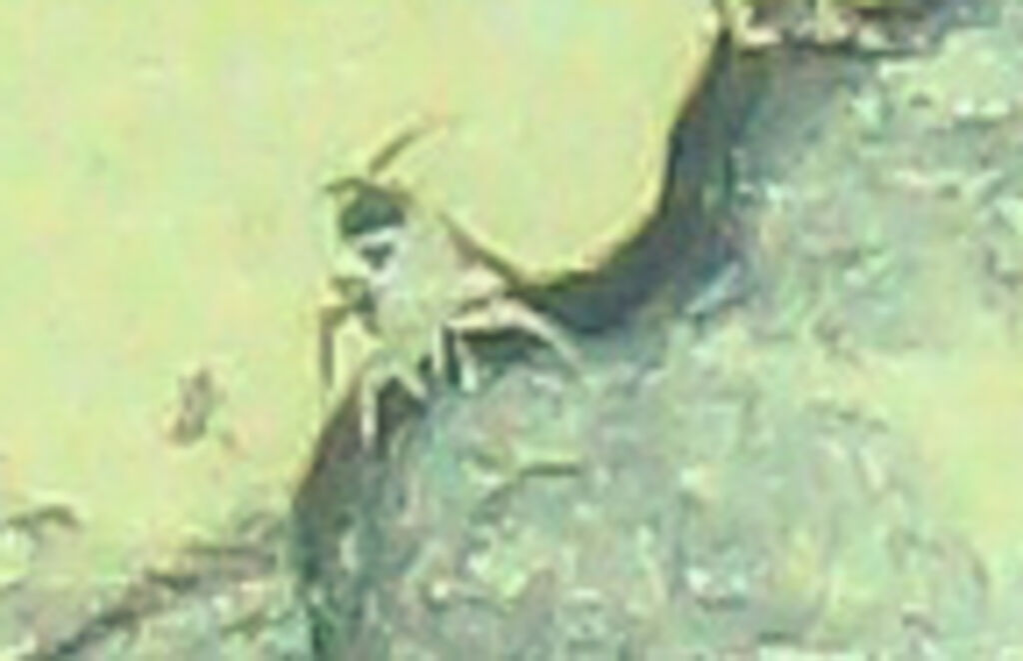
Anomura fam. indet. in situ within the MESO area outside the INDEX area. Image corresponds with the data (Image attribution: BGR).

**Figure 17.**
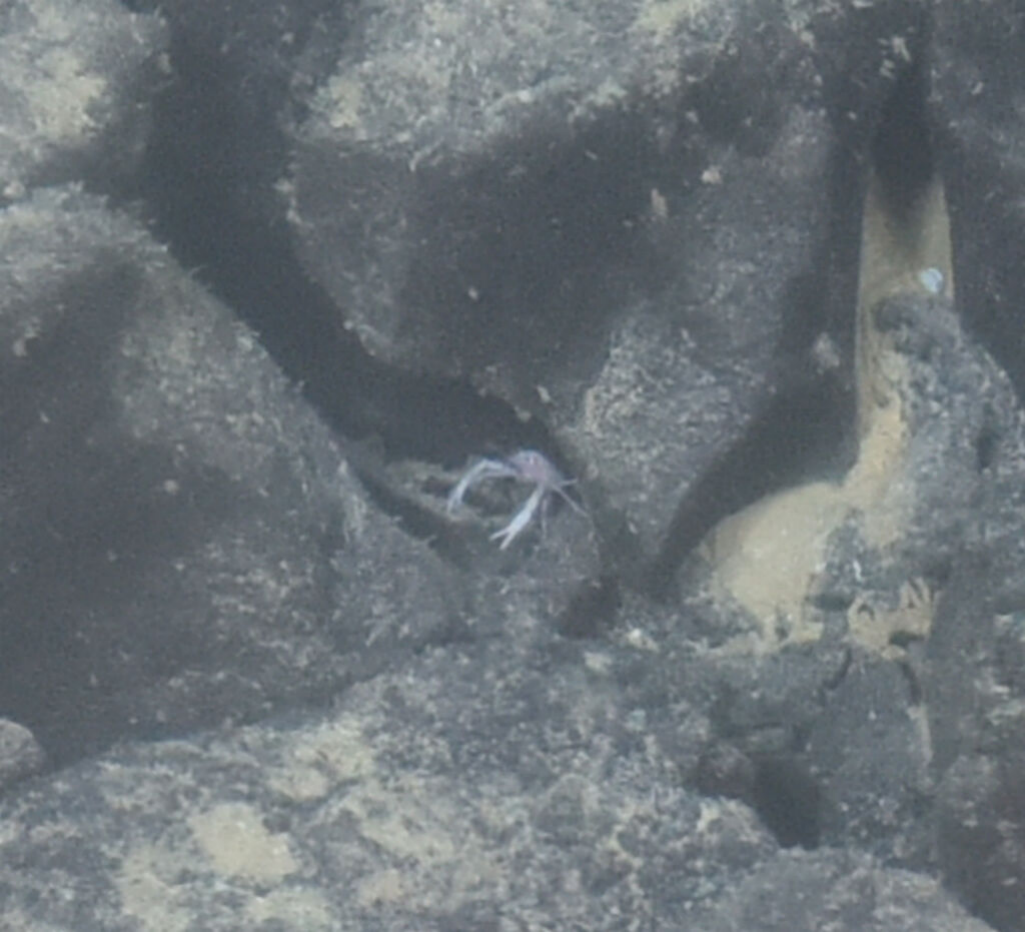
Galatheidae fam. inc. in situ close to the vent site 3 hydrothermal vent field in Cluster 12 of the INDEX area. Image corresponds with the data (Image attribution: BGR).

**Figure 18.**
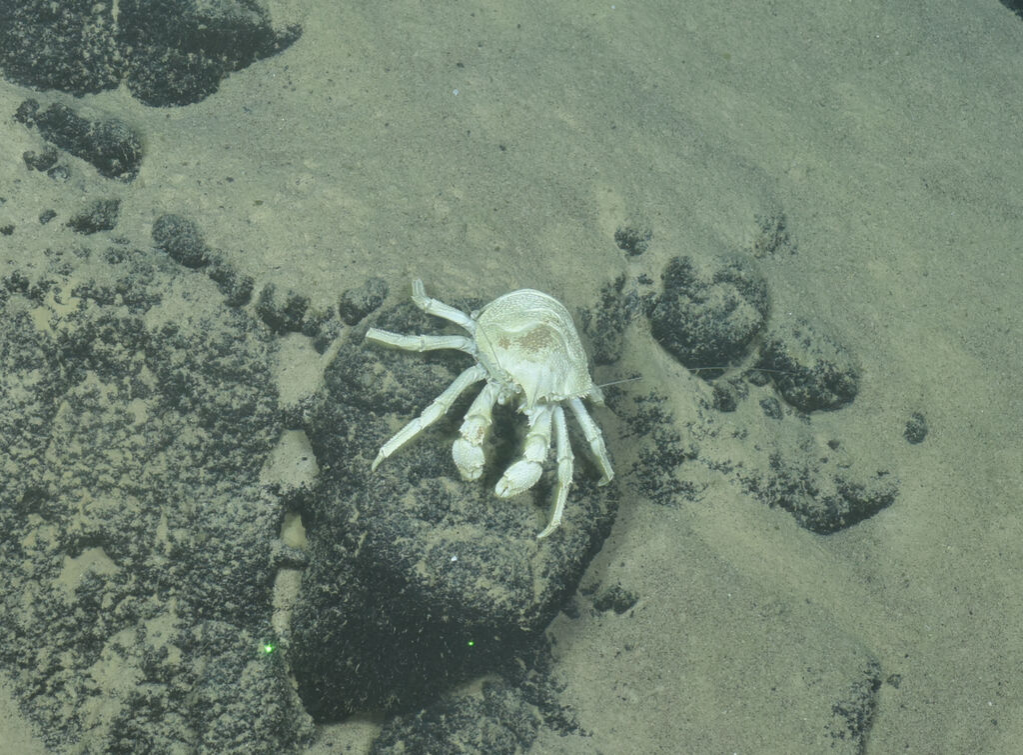
*Munidopsisaries* sp. inc. in situ at the border of the vent site 4 hydrothermal vent field in Cluster 5 of the INDEX area. Image corresponds with the data (Image attribution: BGR).

**Figure 19.**
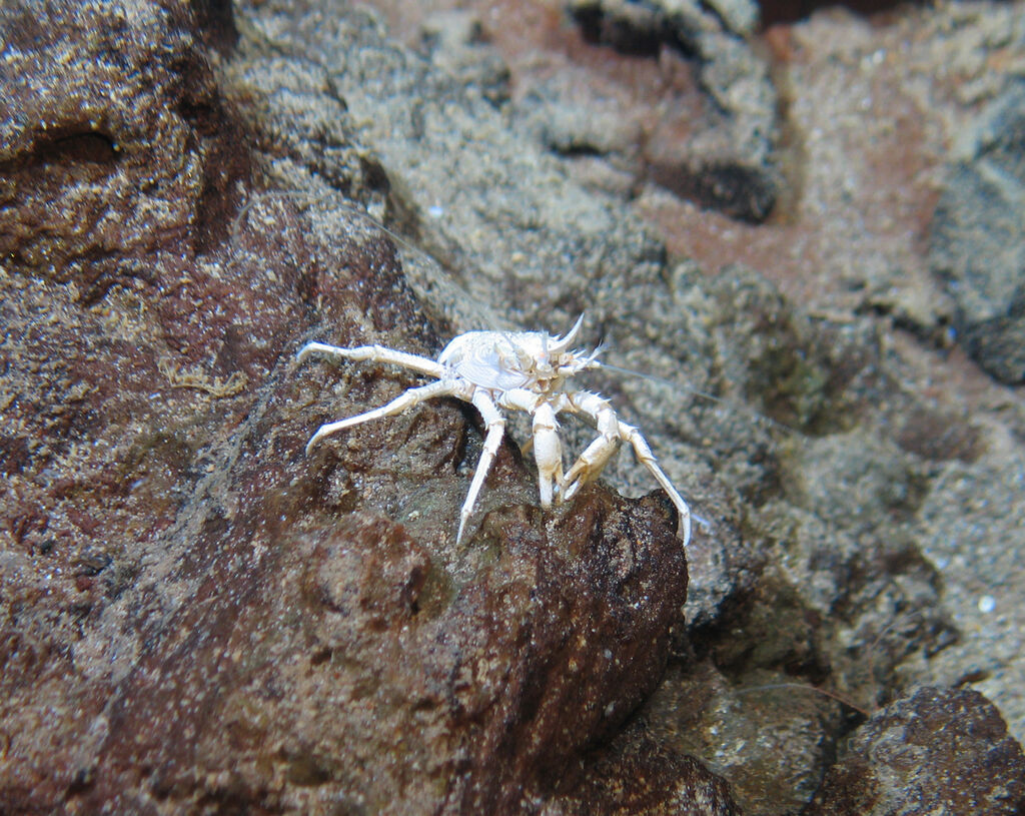
*Munidopsispallida* sp. inc. in situ within the inactive vent site 2 hydrothermal vent field in Cluster 4 of the INDEX area. Image corresponds with the data (Image attribution: BGR and GEOMAR).

**Figure 20.**
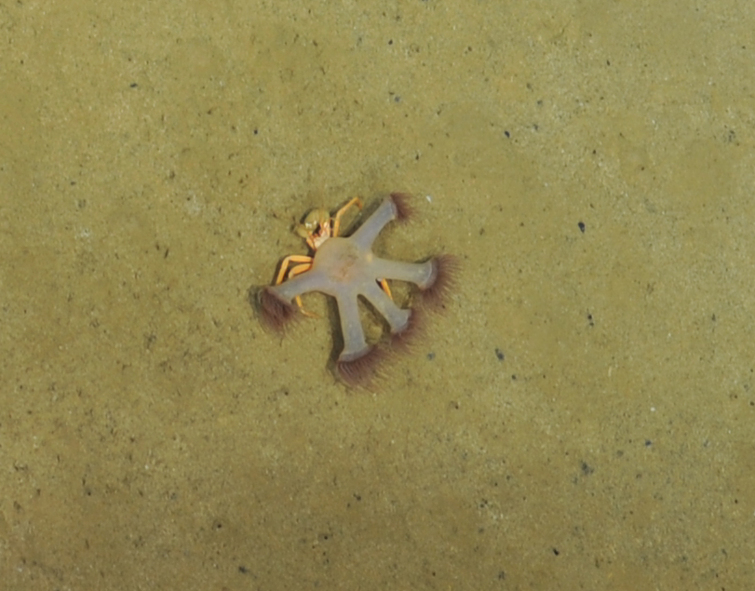
Paguroidea superfam. inc. (in symbiosis with *Epizoanthus* sp. indet.) in situ within the vent site 1 area in Cluster 4 of the INDEX area. Image corresponds with the data (Image attribution: BGR).

**Figure 21.**
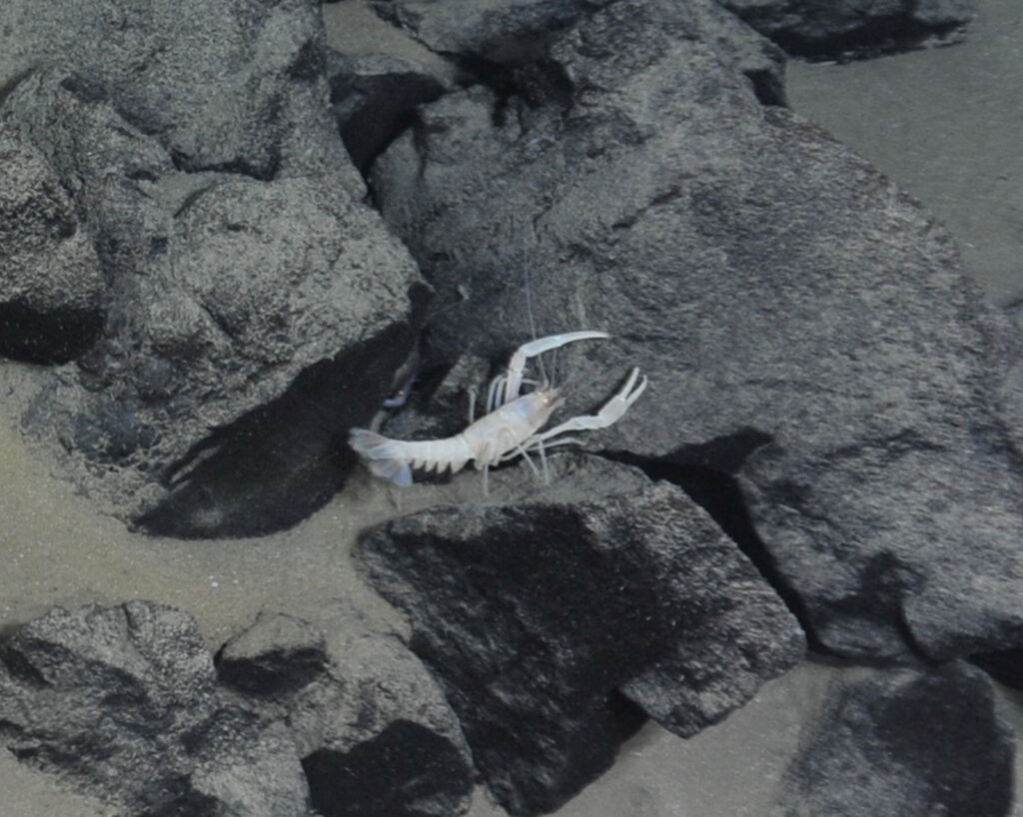
*Thymopideslaurentae* sp. inc. in situ within the vent site 1 area in Cluster 4 of the INDEX area. Image corresponds with the data (Image attribution: BGR).

**Figure 22.**
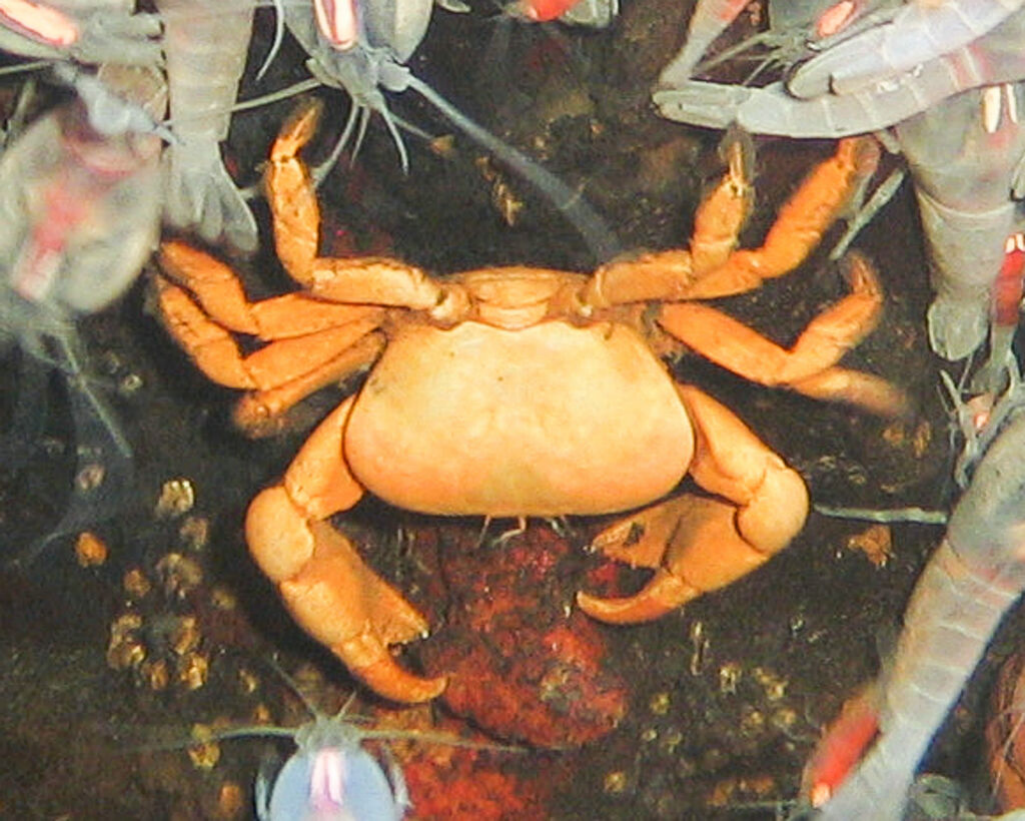
*Austinograearodriguezensis* in situ within the Kairei hydrothermal vent field in Cluster 5 of the INDEX area. Image corresponds with the data (Image attribution: BGR and GEOMAR).

**Figure 23.**
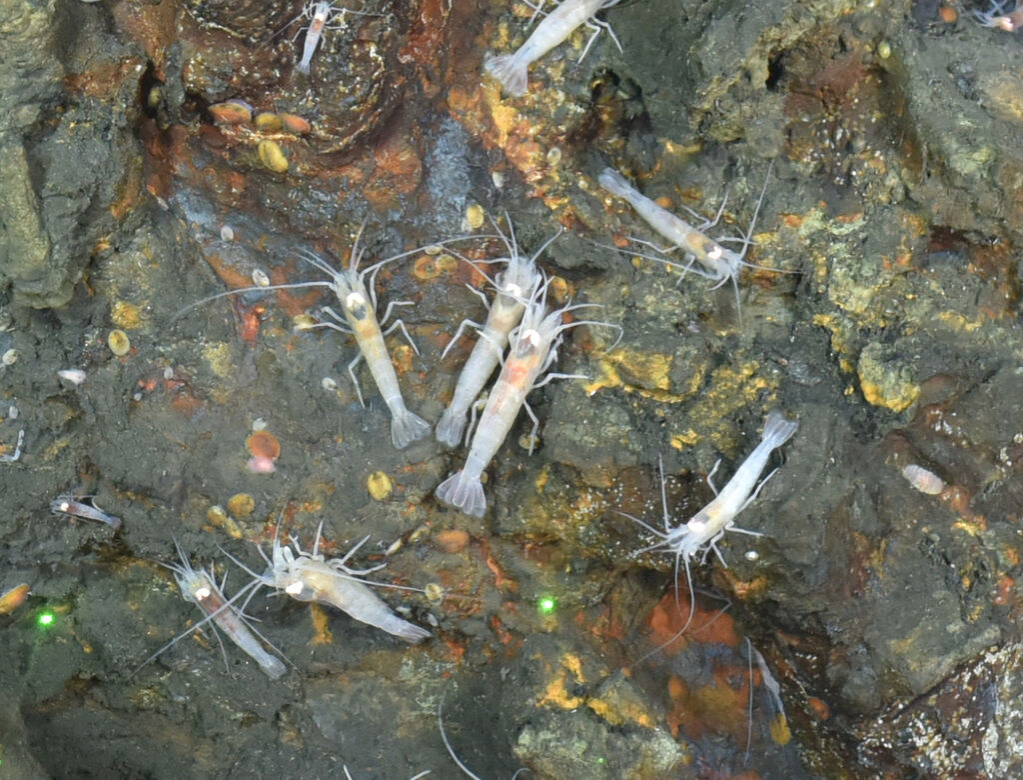
*Alvinocarissolitaire* sp. inc. in situ within the vent site 4 hydrothermal vent field in Cluster 5 of the INDEX area. Image corresponds with the data (Image attribution: BGR).

**Figure 24.**
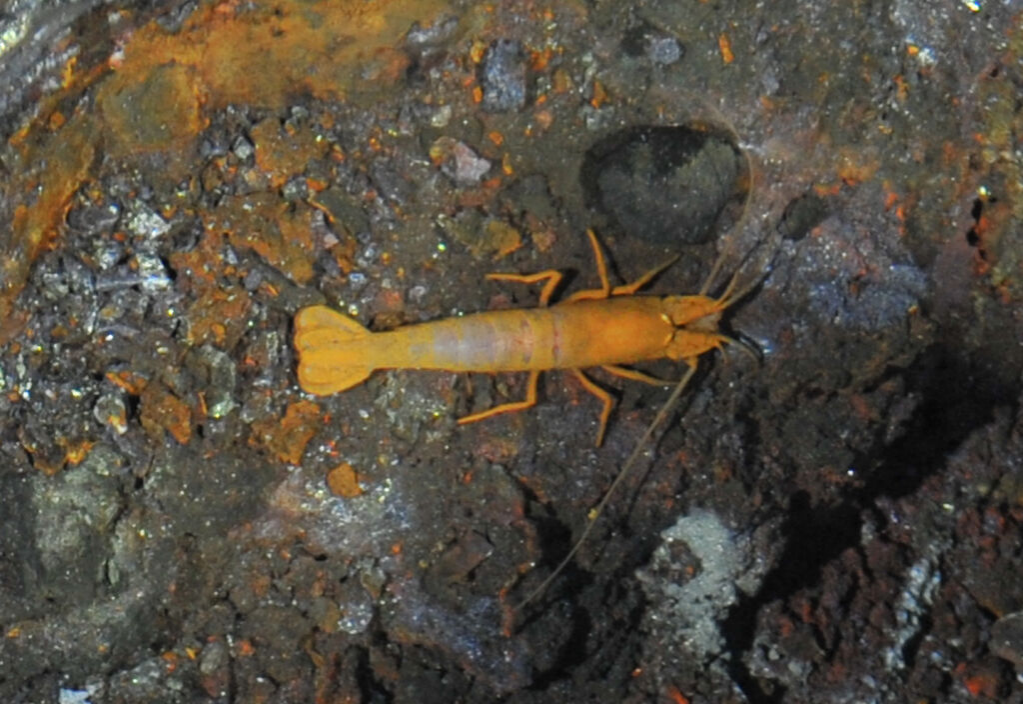
*Mirocarisindica* sp. inc. in situ within the Edmond hydrothermal vent field in Cluster 4 of the INDEX area. Image corresponds with the data (Image attribution: BGR).

**Figure 25.**
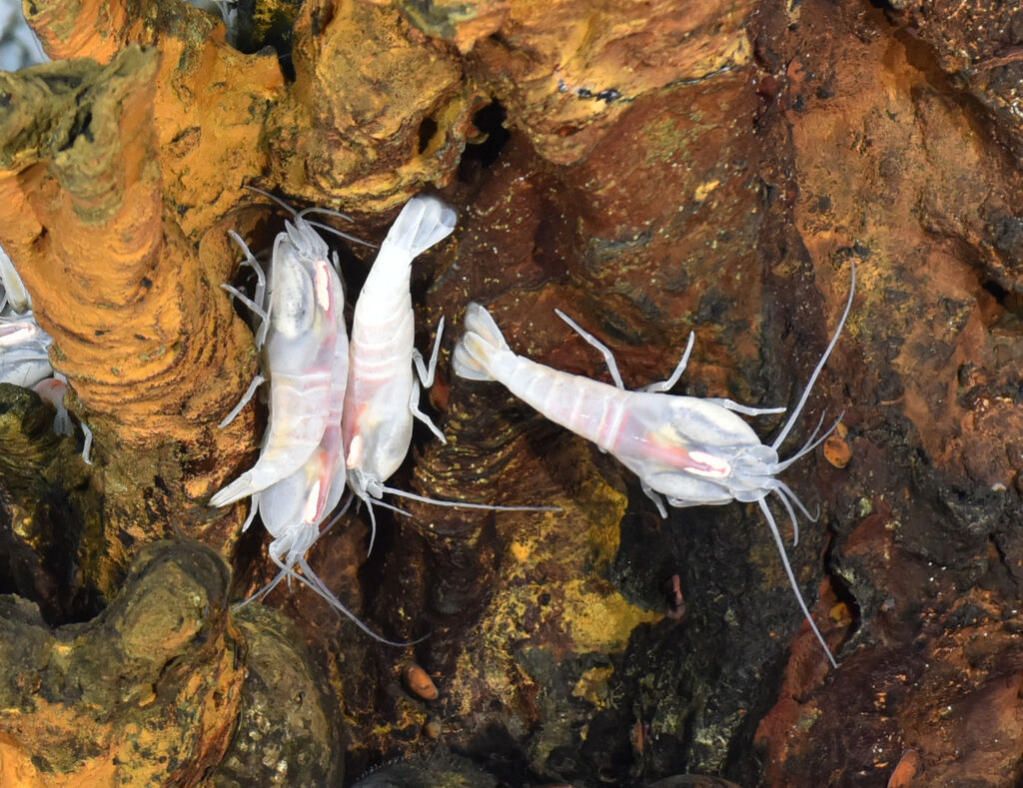
*Rimicariskairei* in situ within the vent site 4 hydrothermal vent field in Cluster 5 of the INDEX area. Image corresponds with the data (Image attribution: BGR).

**Figure 26.**
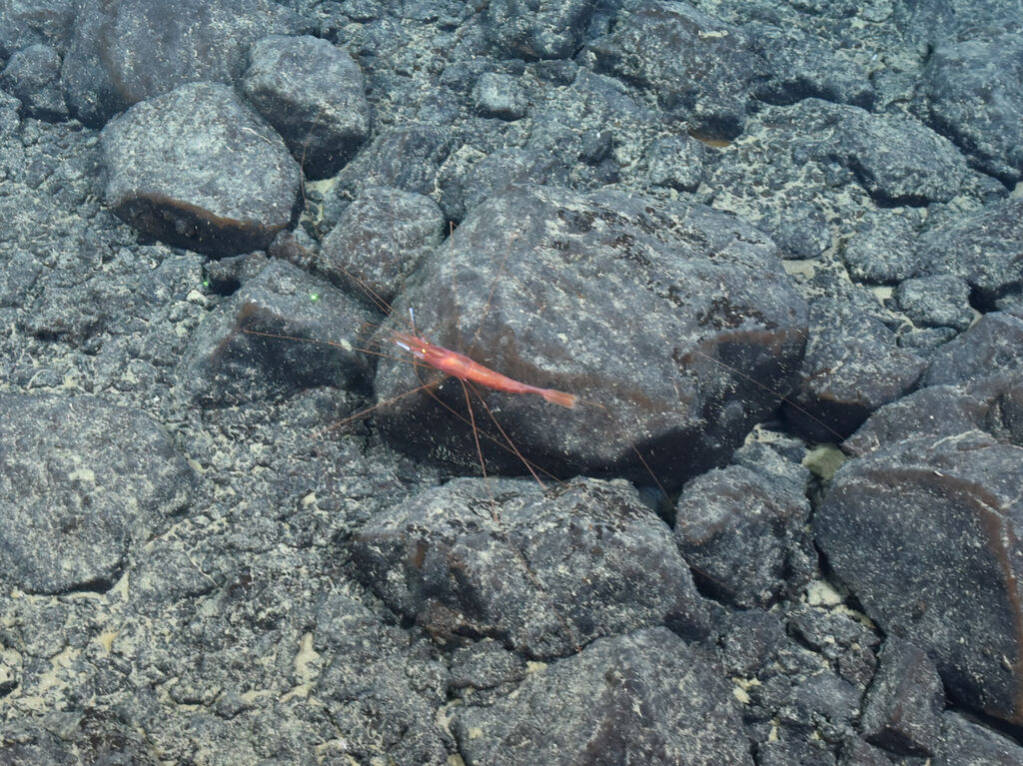
*Nematocarcinus* gen. inc. (DZMB_2021_0004) in situ at the Rodriguez Triple Junction in Cluster 5 of the INDEX area. Image corresponds with the data (Image attribution: BGR).

**Figure 27.**
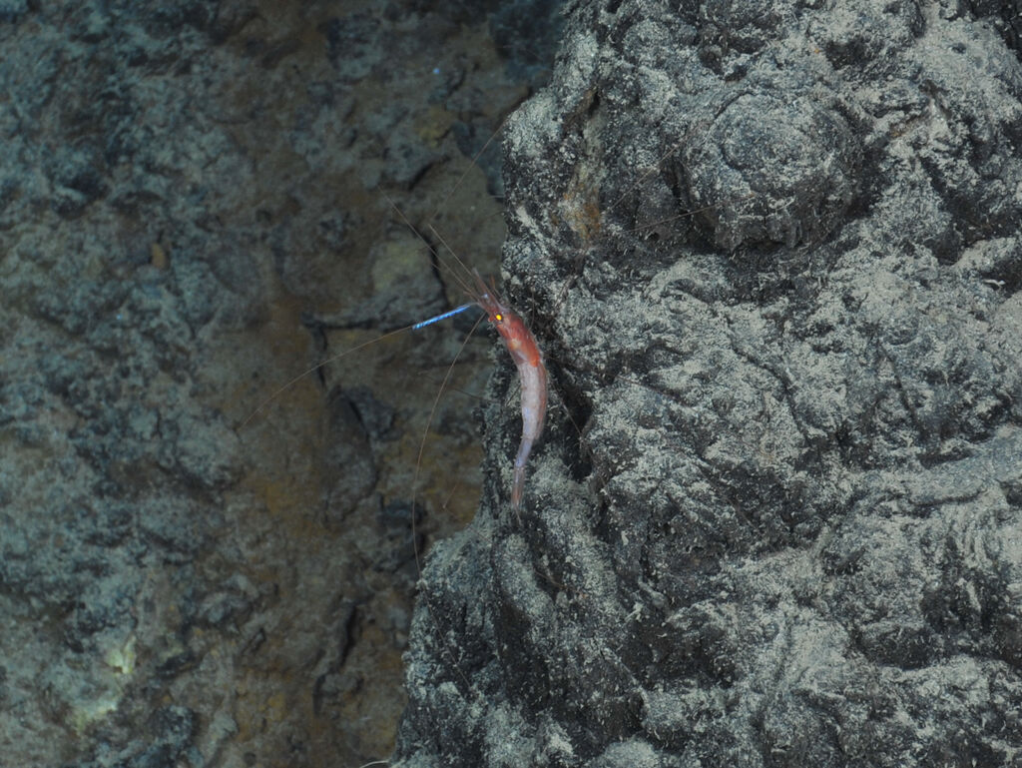
*Nematocarcinus* gen. inc. (DZMB_2021_0005) in situ within the Edmond-vent site 2-vent site 7 hydrothermal area in Cluster 4 of the INDEX area. Image corresponds with the data (Image attribution: BGR).

**Figure 28.**
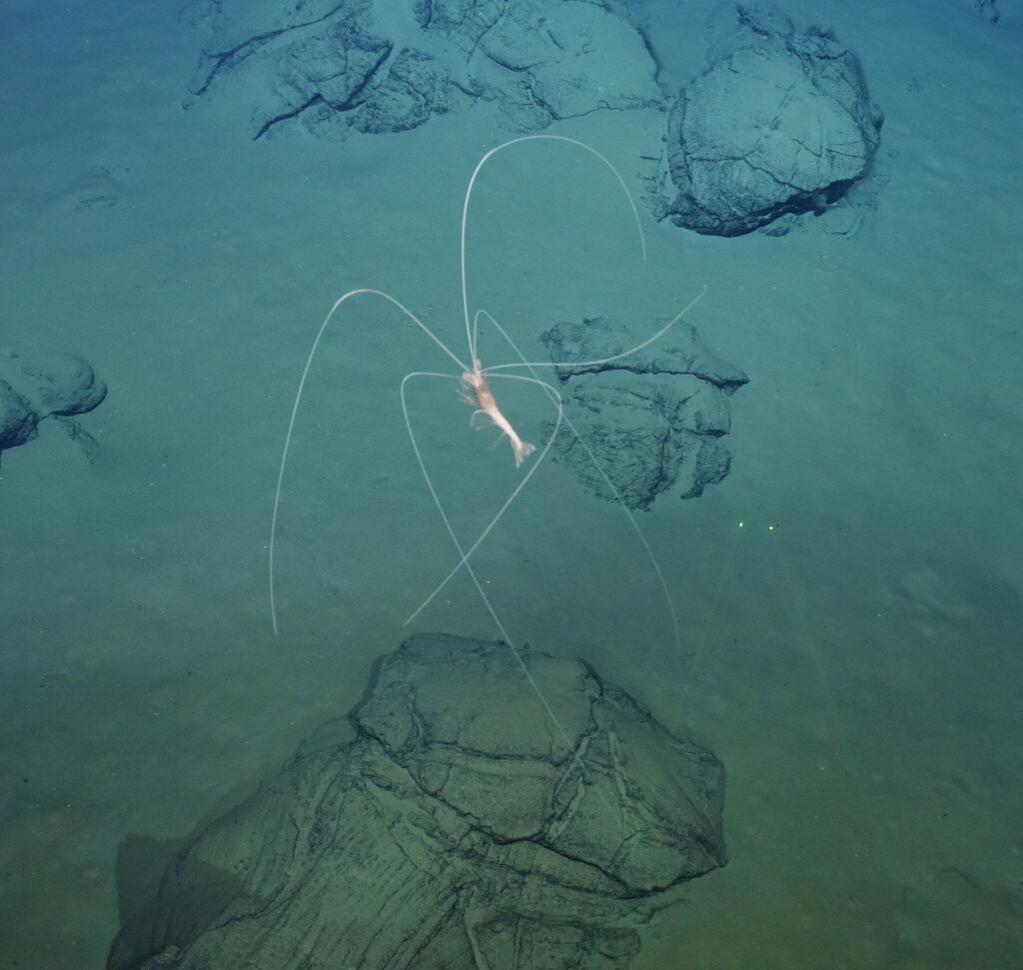
Dendrobranchiata subord. inc. in situ in the surrounding area of the vent site 5 hydrothermal vent field in Cluster 11 of the INDEX area. Image corresponds with the data (Image attribution: BGR).

**Figure 29.**
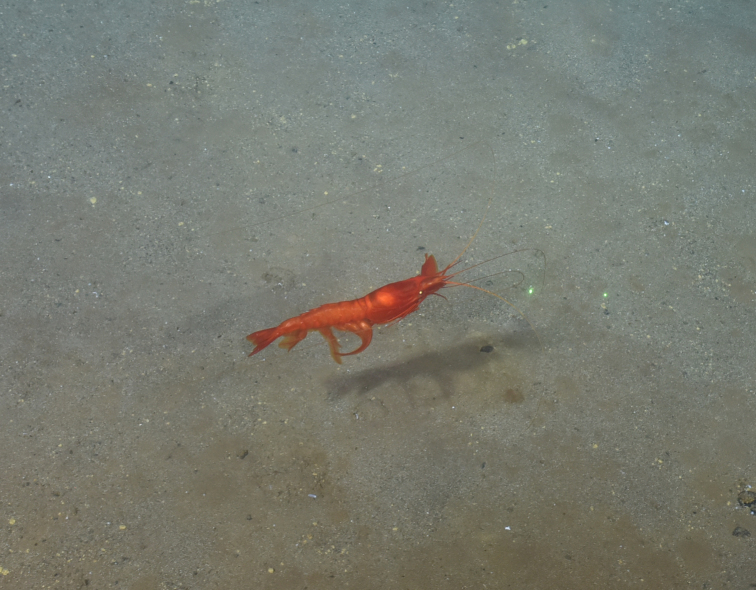
*Cerataspismonstrosus* sp. inc. in situ in the surrounding area of the vent site 6 hydrothermal vent field in Cluster 12 of the INDEX area. Image corresponds with the data (Image attribution: BGR).

**Figure 30.**
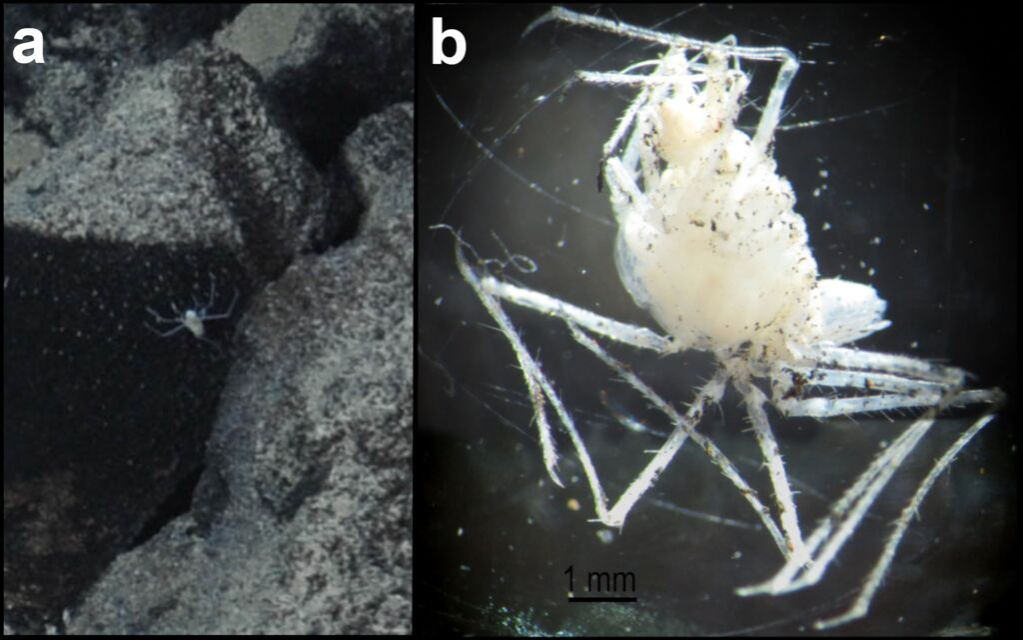
Munnopsidae fam. inc. (DZMB_2021_0006) in situ (a) and sampled specimen (b) within the vent site 1 area in Cluster 4 of the INDEX area. Image corresponds with the data (Image attribution: BGR).

**Figure 31.**
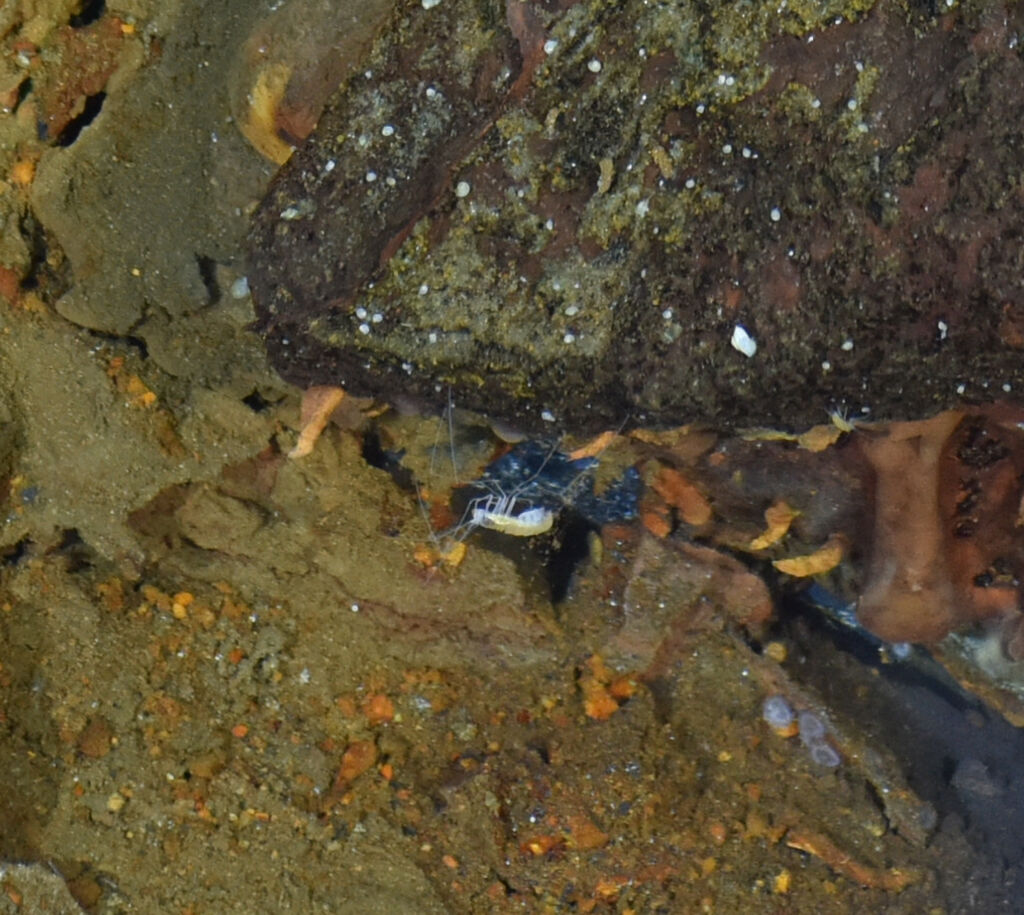
Munnopsidae fam. inc. (DZMB_2021_0007) in situ within the vent site 4 hydrothermal vent field in Cluster 5 of the INDEX area. Image corresponds with the data (Image attribution: BGR).

**Figure 32.**
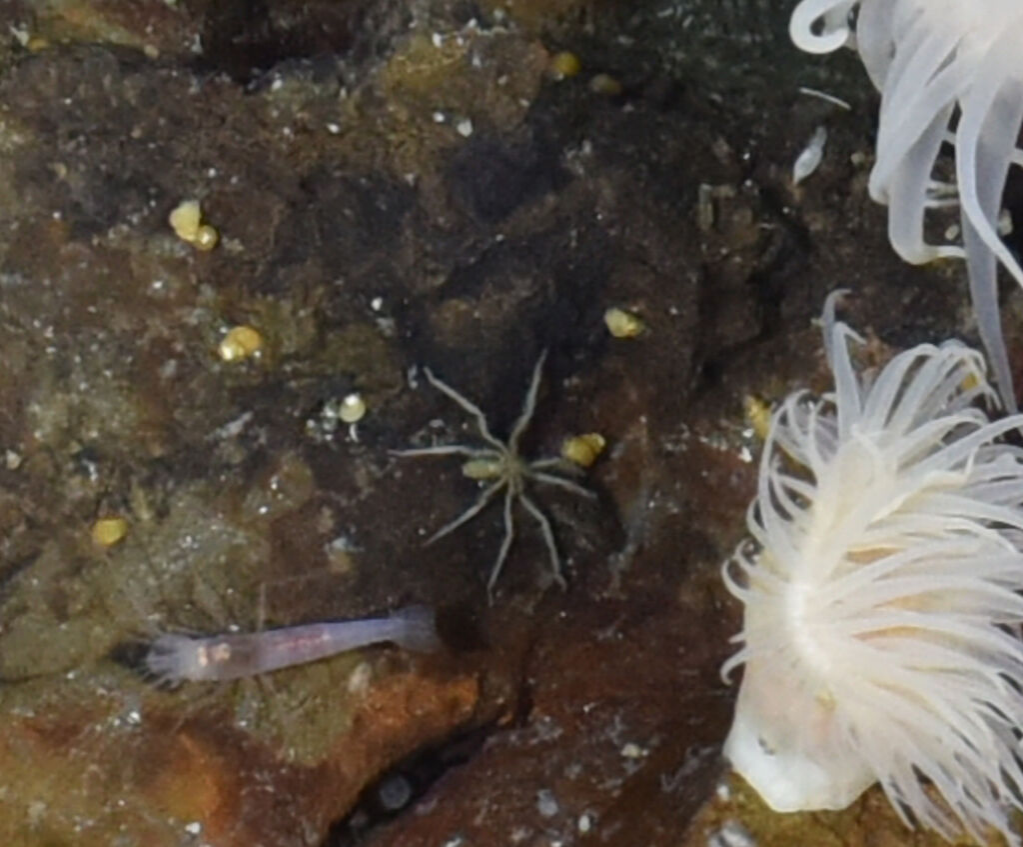
Pantopoda ord. inc. in situ within the vent site 5 hydrothermal vent field in Cluster 11 of the INDEX area. Image corresponds with the data below (Image attribution: BGR).

**Figure 33.**
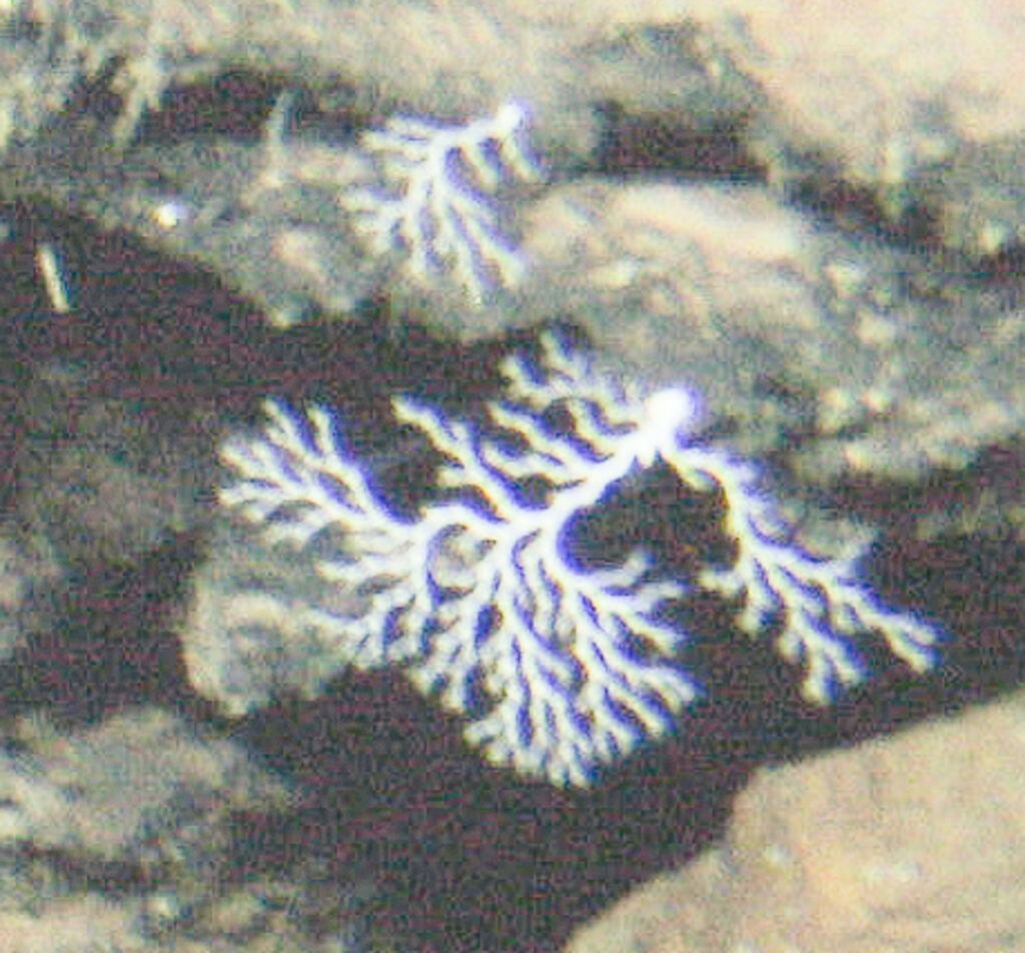
Cheilostomatida fam. indet. (DZMB_2021_0008) in situ in the MESO area outside the INDEX area. Image corresponds with the data (Image attribution: BGR and GEOMAR).

**Figure 34.**
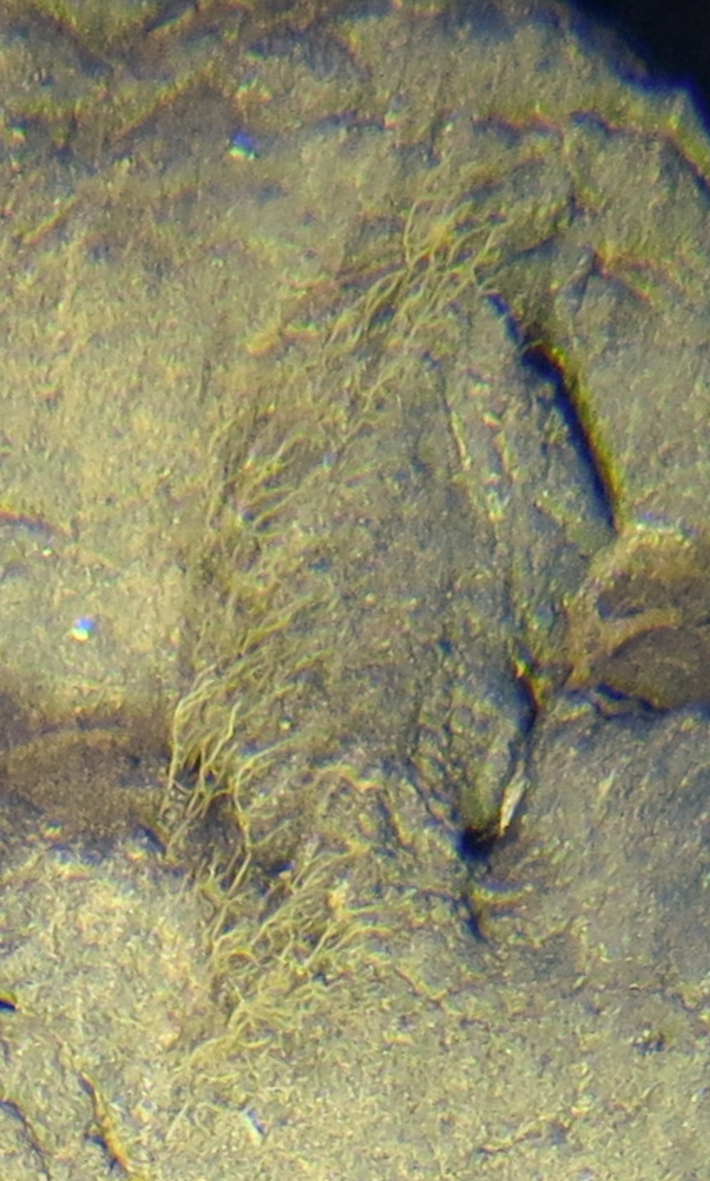
Cheilostomatida fam. indet. (DZMB_2021_0009) in situ close to the vent site 5 hydrothermal vent field in Cluster 11 of the INDEX area. Image corresponds with the data (Image attribution: BGR).

**Figure 35.**
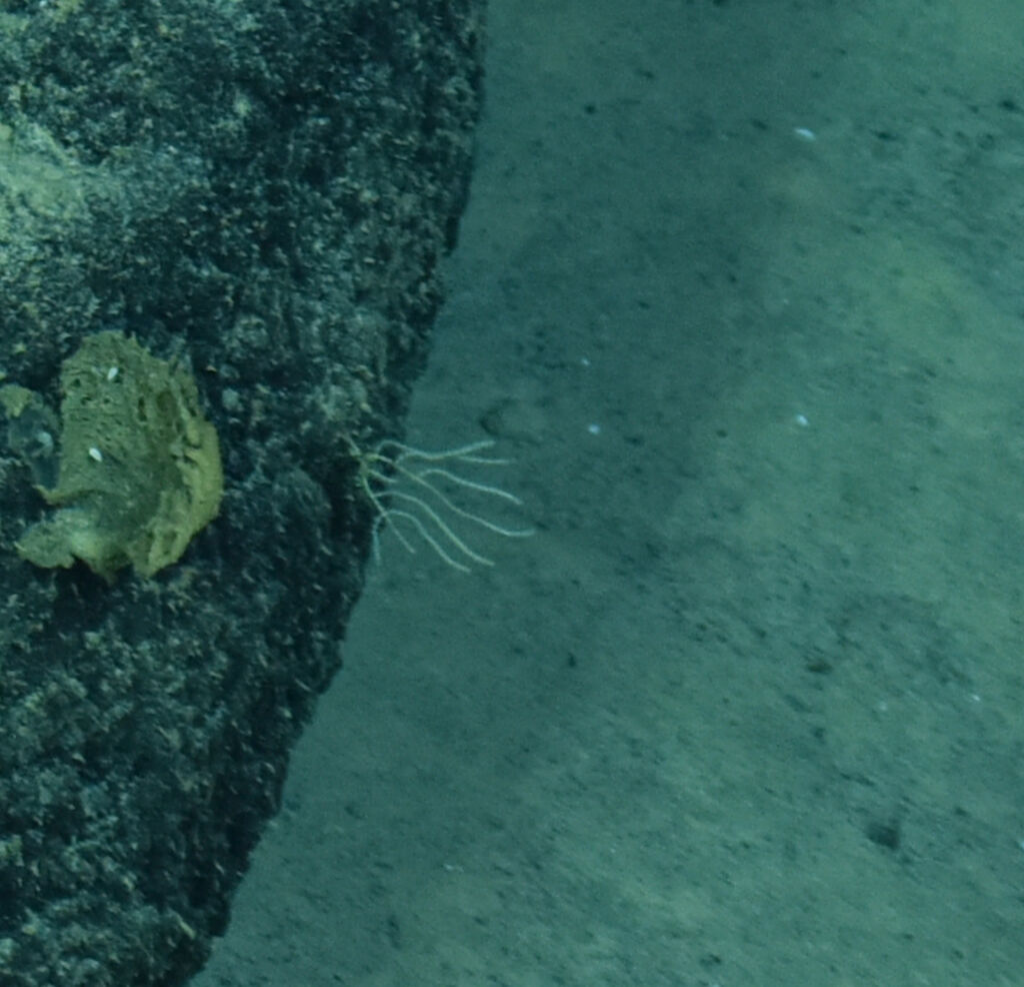
*Bifaxaria* gen. inc. in situ close to the vent site 5 hydrothermal vent field in Cluster 11 of the INDEX area. Image corresponds with the data (Image attribution: BGR).

**Figure 36.**
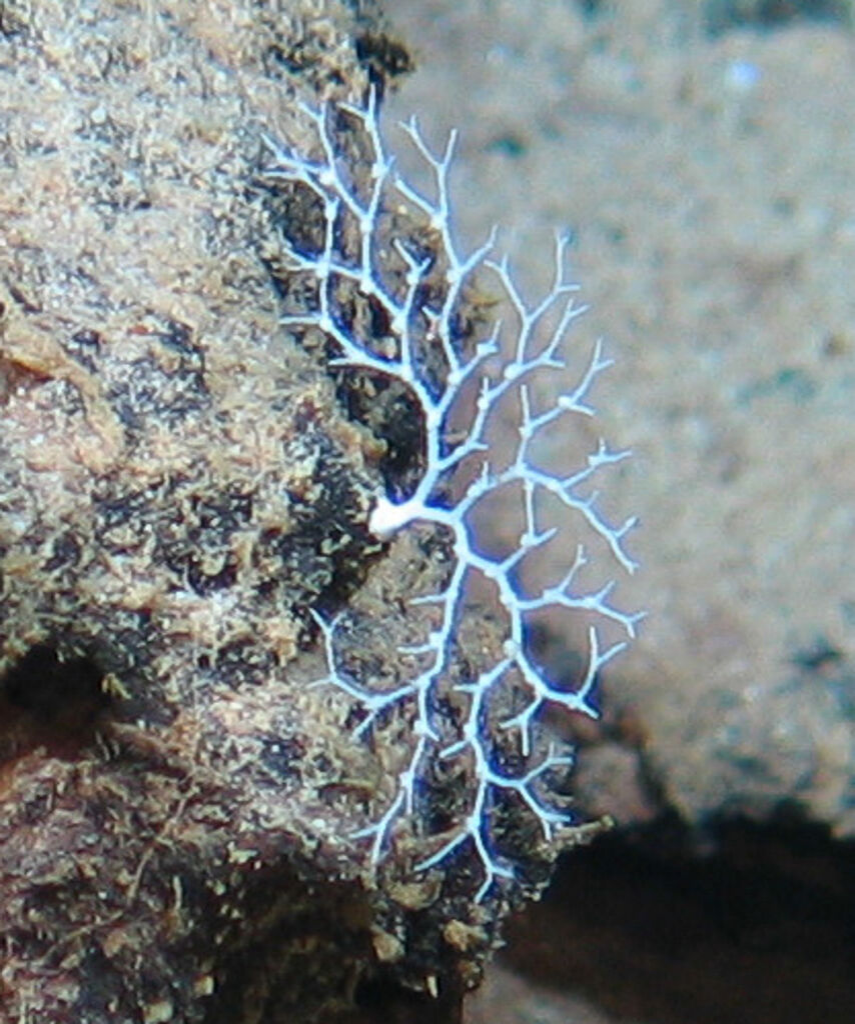
*Tessaradoma* gen. inc. in situ within the MESO area outside the INDEX area. Image corresponds with the data (Image attribution: BGR and GEOMAR).

**Figure 37.**
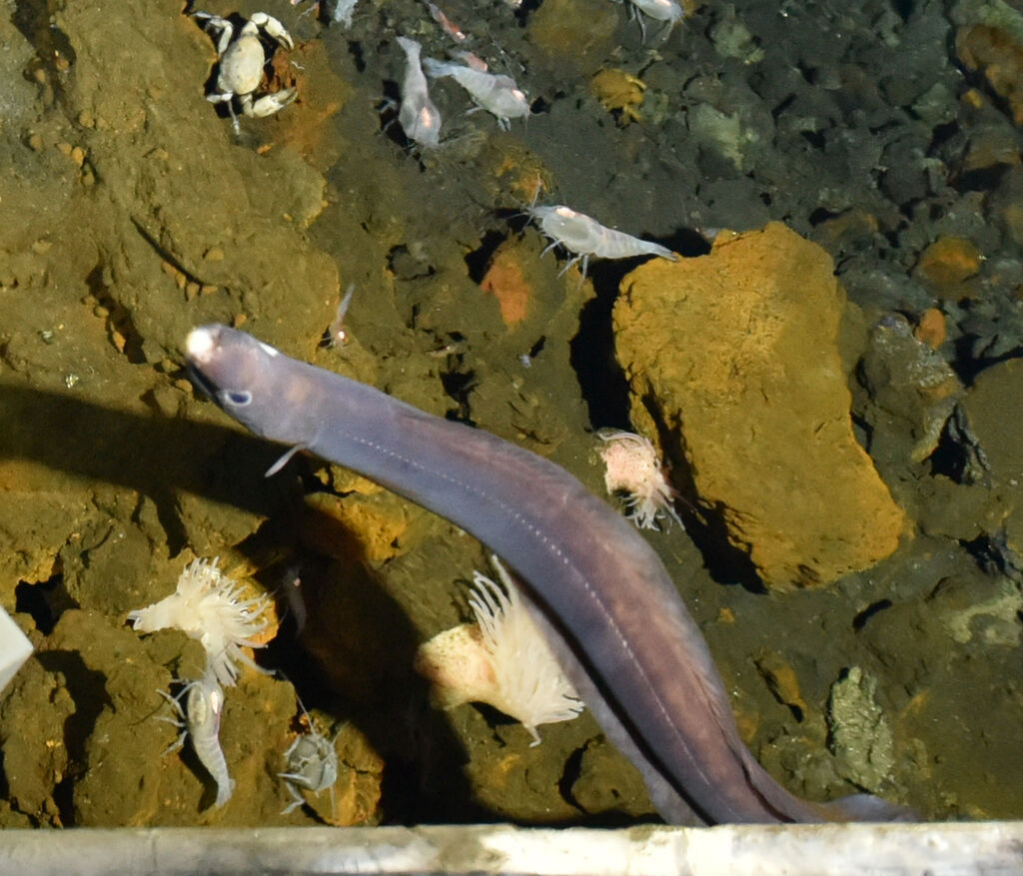
Synaphobranchidae gen. indet. in situ within the vent site 6 hydrothermal vent field in Cluster 12 of the INDEX area. Image corresponds with the data (Image attribution: BGR).

**Figure 38.**
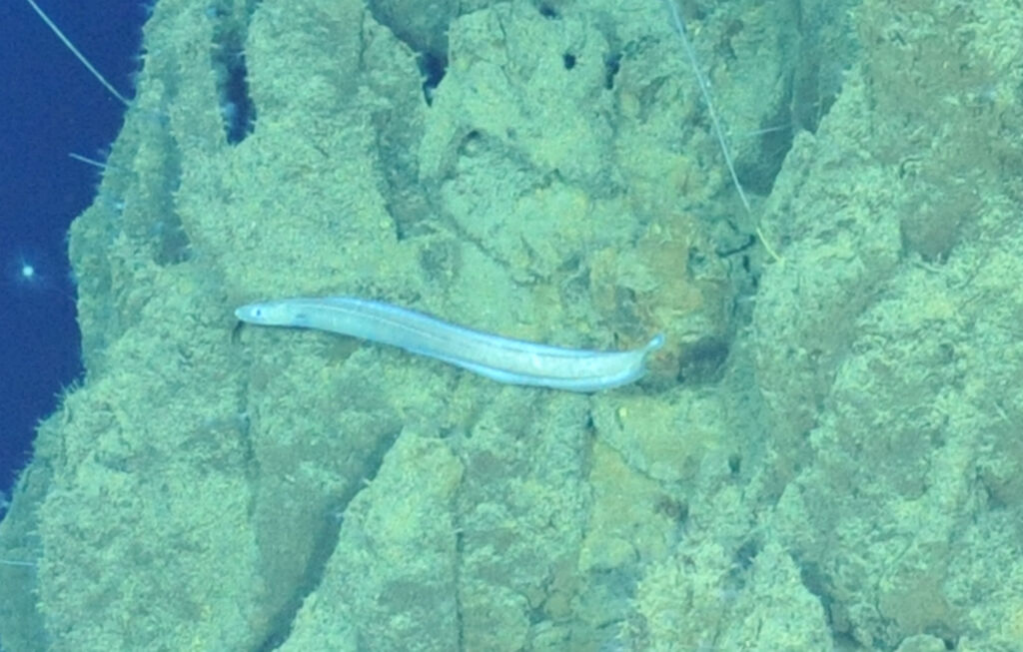
*Histiobranchus* gen. inc. in situ within the vent site 1 area in Cluster 4 of the INDEX area. Image corresponds with the data (Image attribution: BGR).

**Figure 39.**
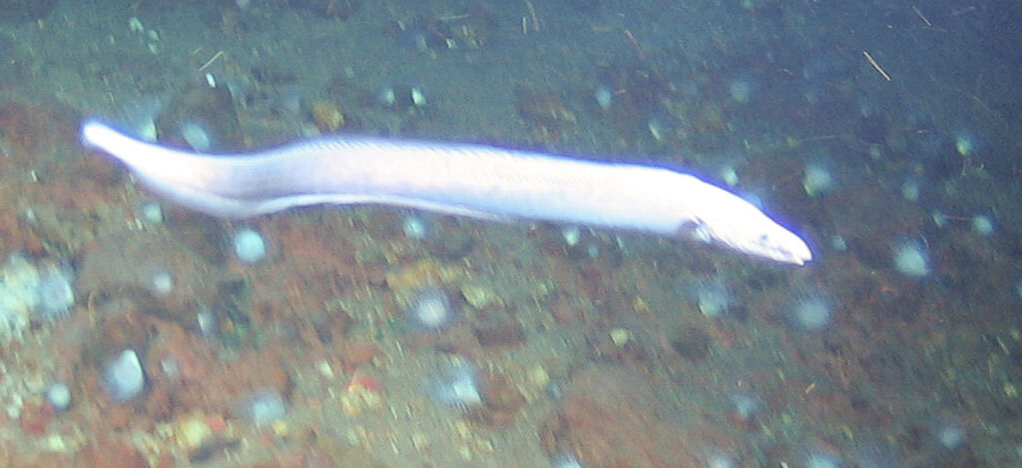
Synaphobranchidae *Ilyophisbrunneus* fam. inc. in situ within the Kairei hydrothermal vent field in Cluster 5 of the INDEX area. Image corresponds with the data (Image attribution: BGR and GEOMAR).

**Figure 40.**
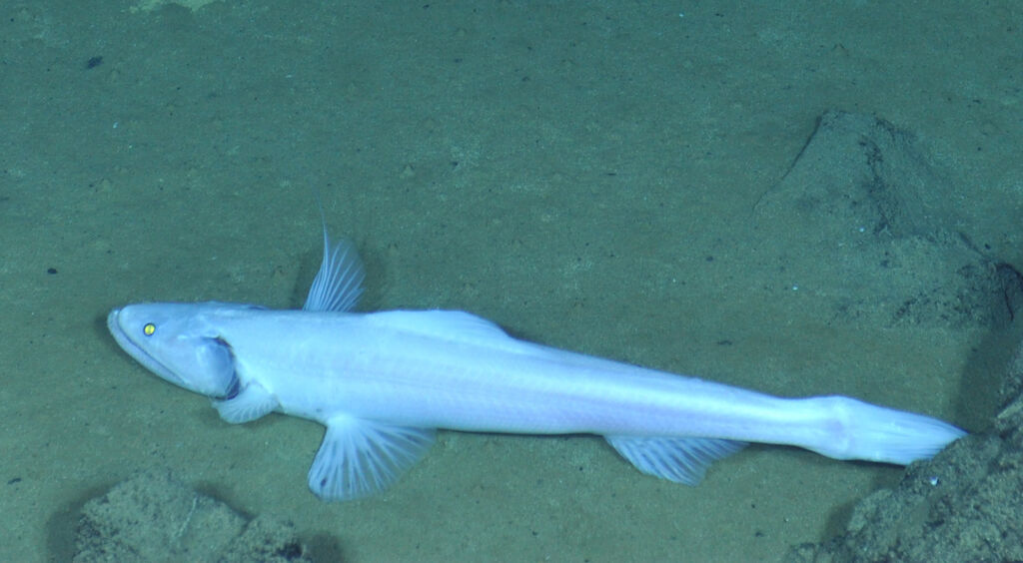
*Bathysaurusmollis* sp. inc. in situ within the vent site 1 area in Cluster 4 of the INDEX area. Image corresponds with the data (Image attribution: BGR).

**Figure 41.**
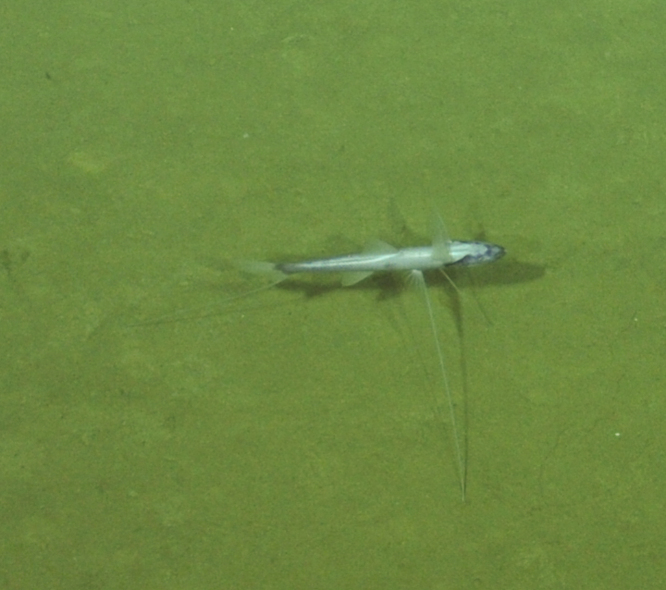
*Bathypterois* sp. indet. in situ in the Edmond-vent site 2-vent site 7 area in Cluster 4 of the INDEX area. Image corresponds with the data (Image attribution: BGR).

**Figure 42.**
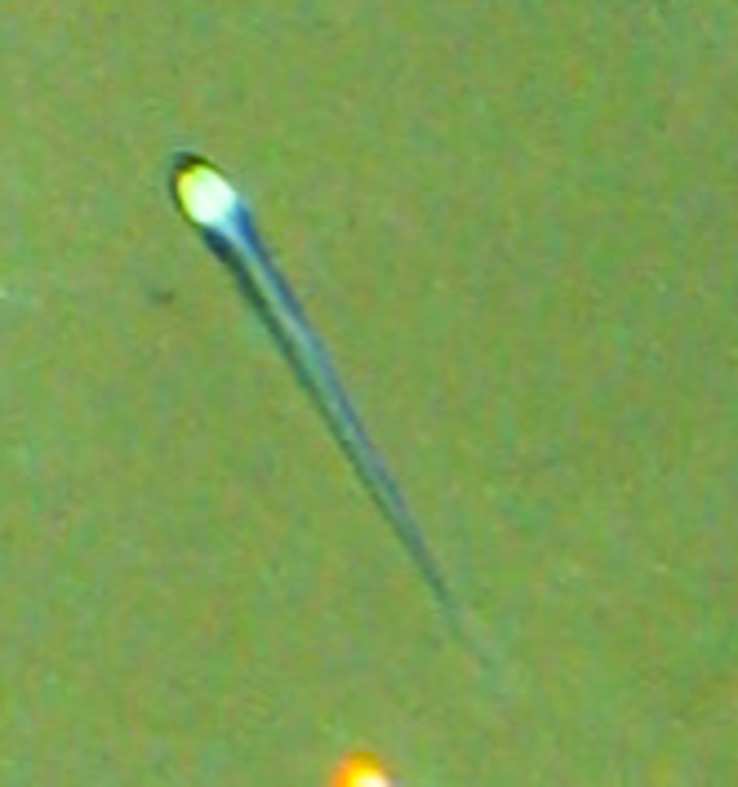
*Ipnopsagassizii* sp. inc. in situ close to the Edmond hydrothermal vent field in Cluster 4 of the INDEX area. Image corresponds with the data (Image attribution: BGR).

**Figure 43.**
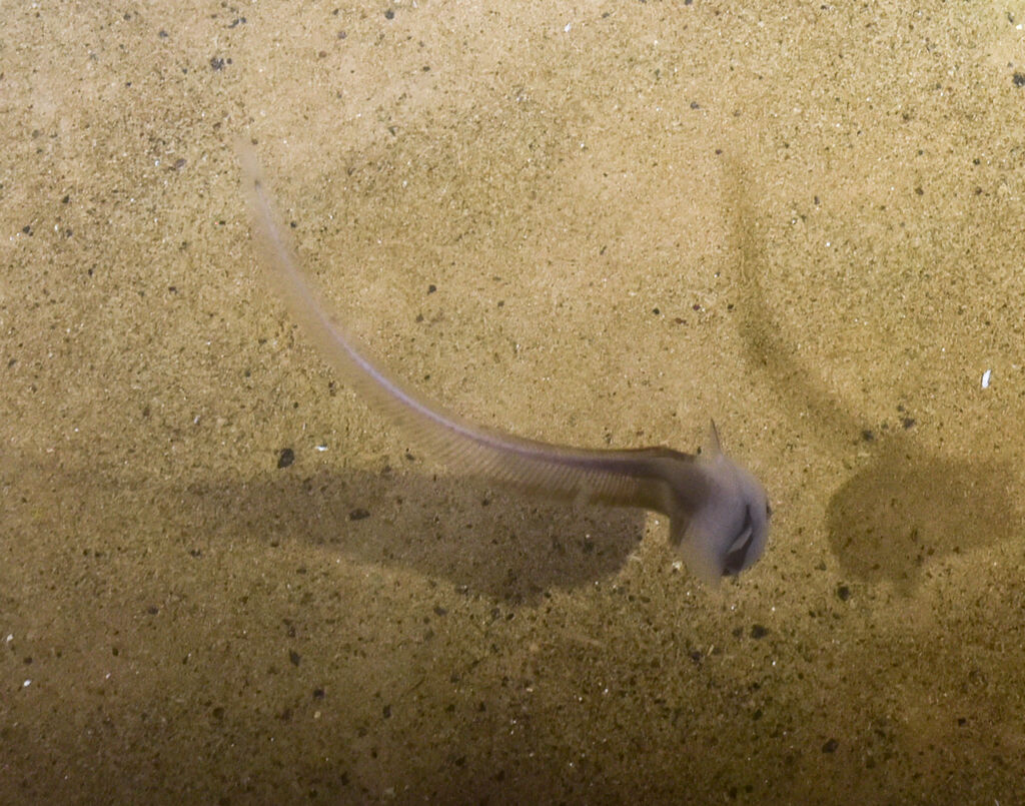
Gadiformes Macrouridae ord. inc. (DZMB_2021_0010) in situ close to the vent site 6 hydrothermal vent field in Cluster 12 of the INDEX area. Image corresponds with the data (Image attribution: BGR).

**Figure 44.**
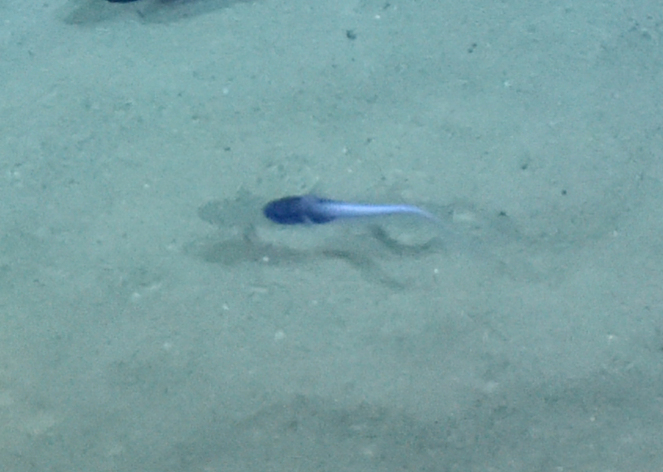
Gadiformes Macrouridae ord. inc. (DZMB_2021_0011) in situ close to the vent site 6 hydrothermal vent field in Cluster 12 of the INDEX area. Image corresponds with the data (Image attribution: BGR).

**Figure 45.**
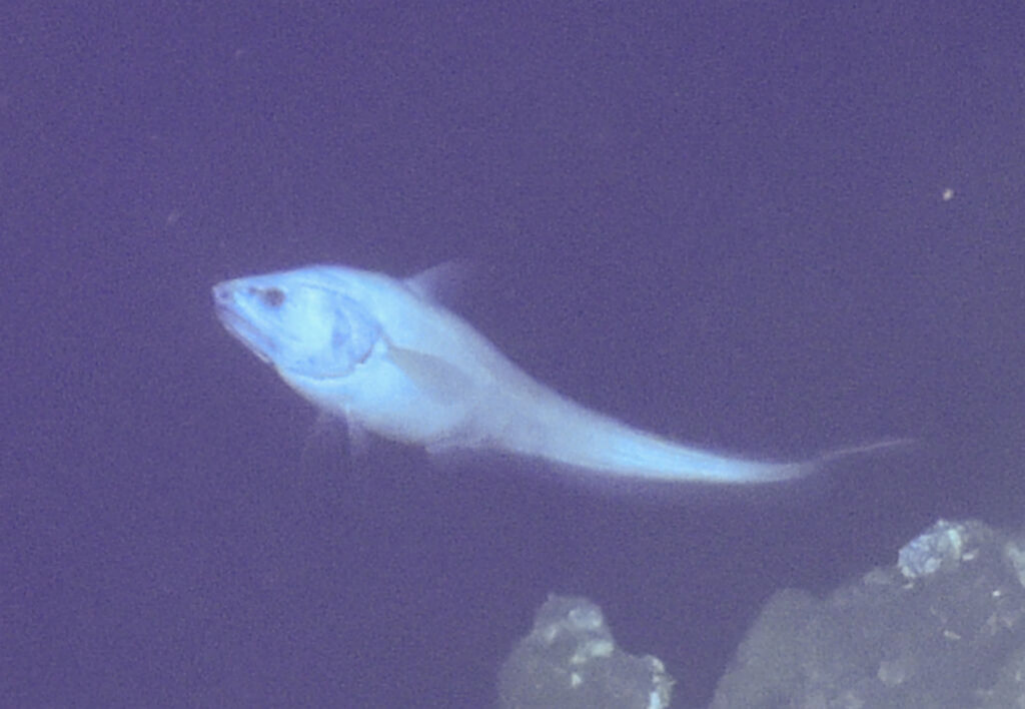
*Coryphaenoides* gen. inc. (DZMB_2021_0012) in situ within the vent site 5 hydrothermal vent field in Cluster 11 of the INDEX area. Image corresponds with the data (Image attribution: BGR).

**Figure 46.**
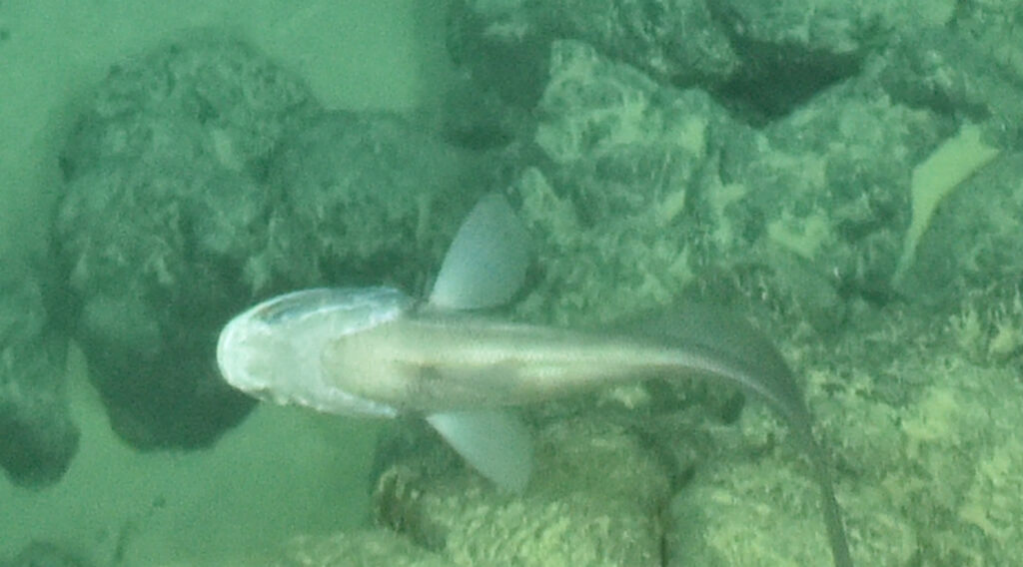
*Coryphaenoides* gen. inc. (DZMB_2021_0013) in situ close to the vent site 6 hydrothermal vent field in Cluster 12 of the INDEX area. Image corresponds with the data (Image attribution: BGR).

**Figure 47.**
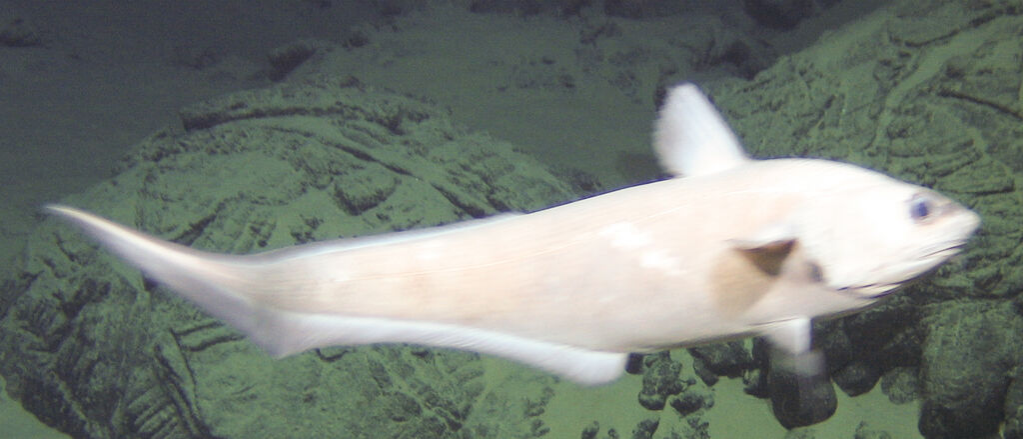
*Coryphaenoidesarmatus* sp. inc. in situ within the MESO area outside the INDEX area. Image corresponds with the data (Image attribution: BGR and GEOMAR).

**Figure 48.**
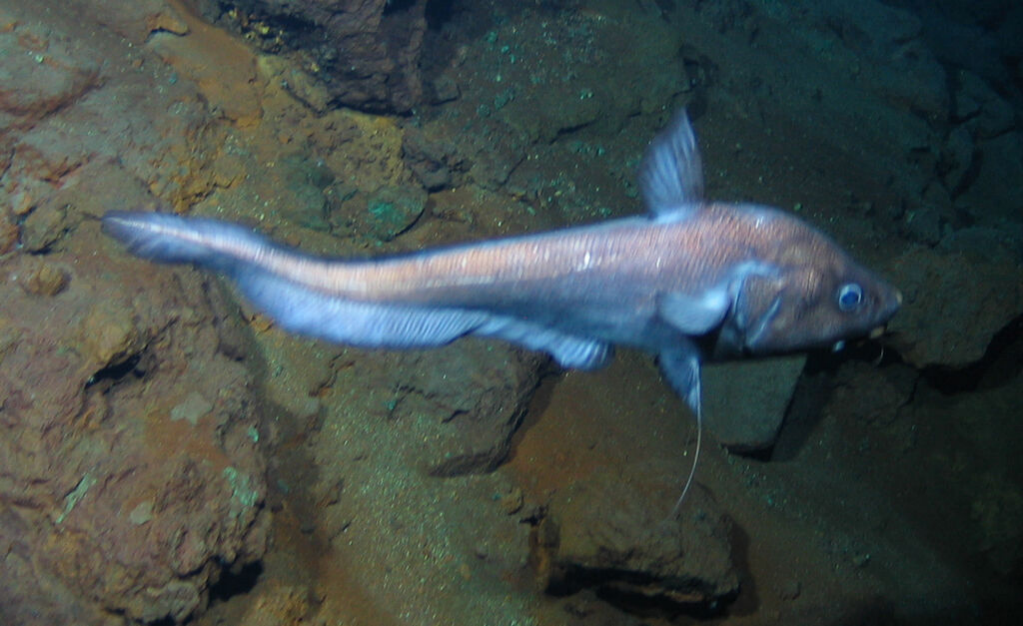
*Coryphaenoideslongifilis* sp. inc. in situ within the Kairei hydrothermal vent field in Cluster 5 of the INDEX area. Image corresponds with the data (Image attribution: BGR and GEOMAR).

**Figure 49.**
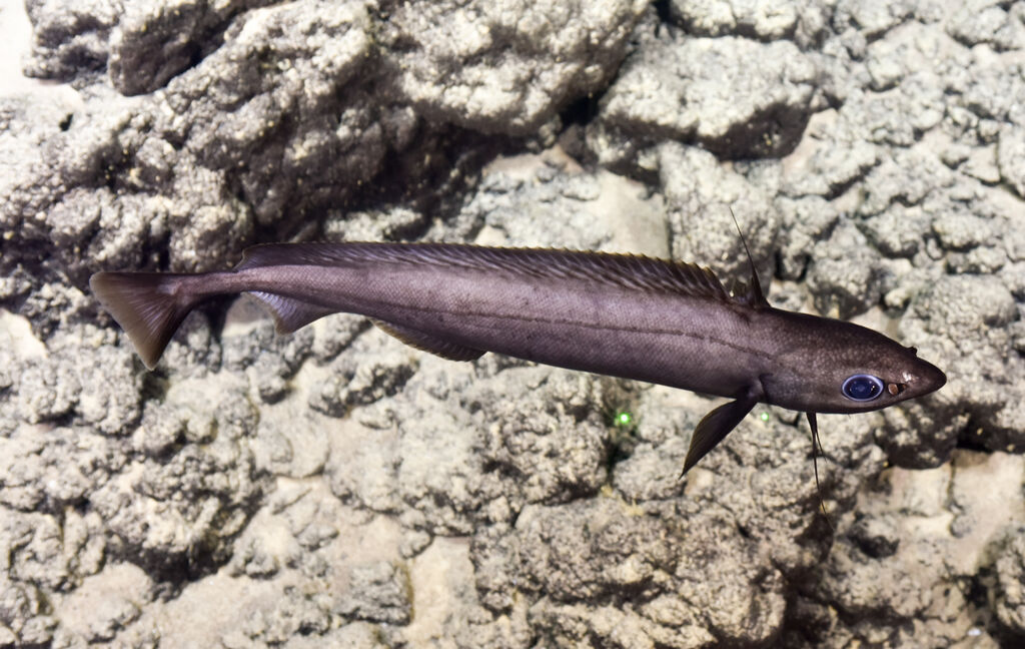
*Antimorarostrata* in situ in the surrounding area of the vent site 4 hydrothermal vent field in Cluster 5 of the INDEX area. Image corresponds with the data (Image attribution: BGR).

**Figure 50.**
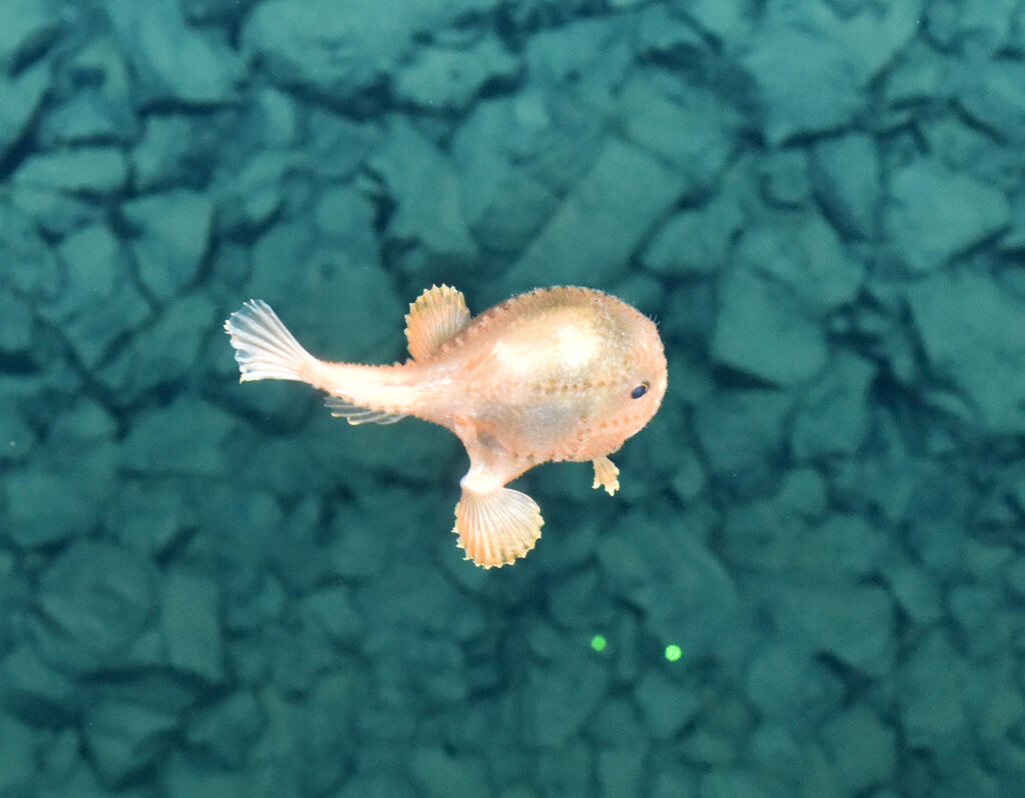
*Chaunacops* gen. inc. in situ in the surrounding area of the vent site 5 hydrothermal vent field in Cluster 11 of the INDEX area. Image corresponds with the data (Image attribution: BGR).

**Figure 51.**
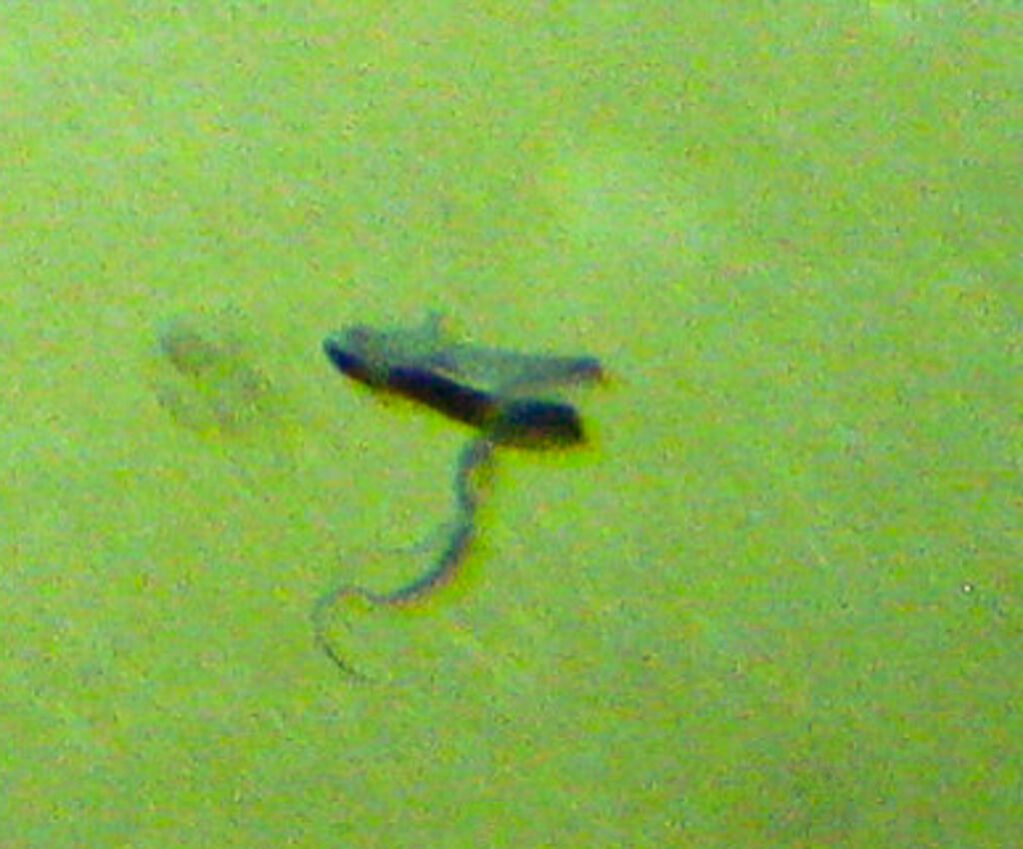
Notacanthiformes ord. inc. in situ in the surrounding area of the Edmond hydrothermal vent field in Cluster 4 of the INDEX area. Image corresponds with the data (Image attribution: BGR).

**Figure 52.**
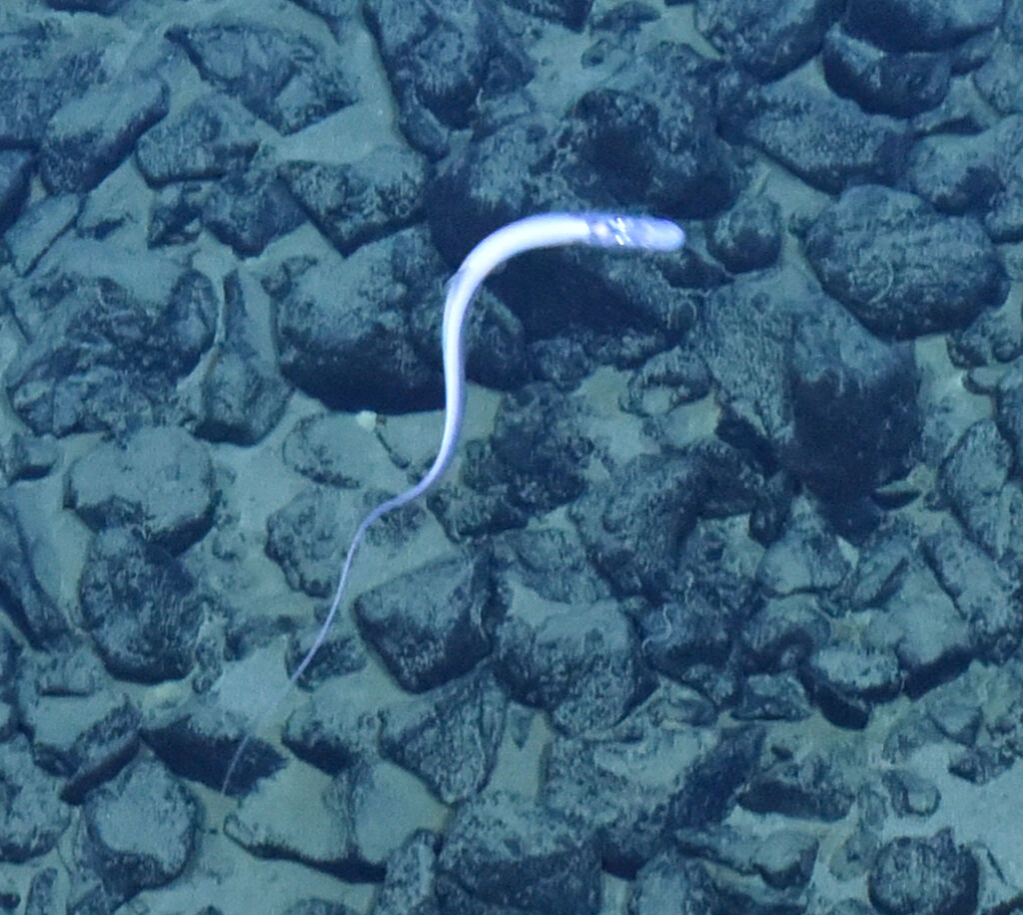
*Aldrovandiaaffinis* gen. inc. in situ in the surrounding area of the vent site 6 hydrothermal vent field in Cluster 12 of the INDEX area. Image corresponds with the data (Image attribution: BGR).

**Figure 53.**
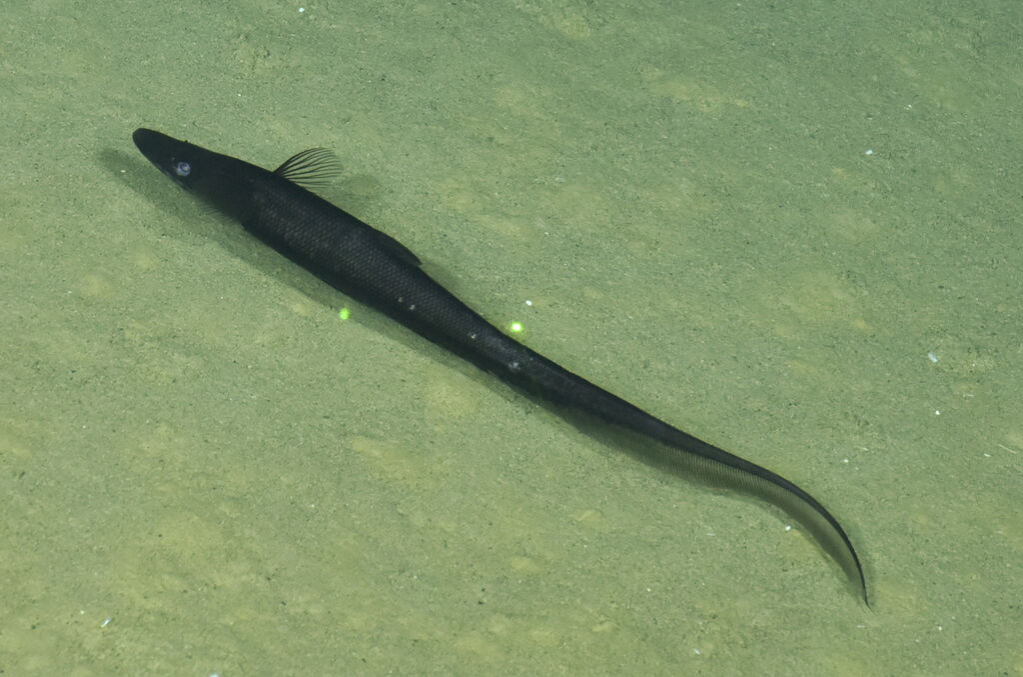
*Halosauropsismacrochir* gen. inc. in situ at the Rodriguez Triple Junction in Cluster 5 of the INDEX area. Image corresponds with the data (Image attribution: BGR).

**Figure 54.**
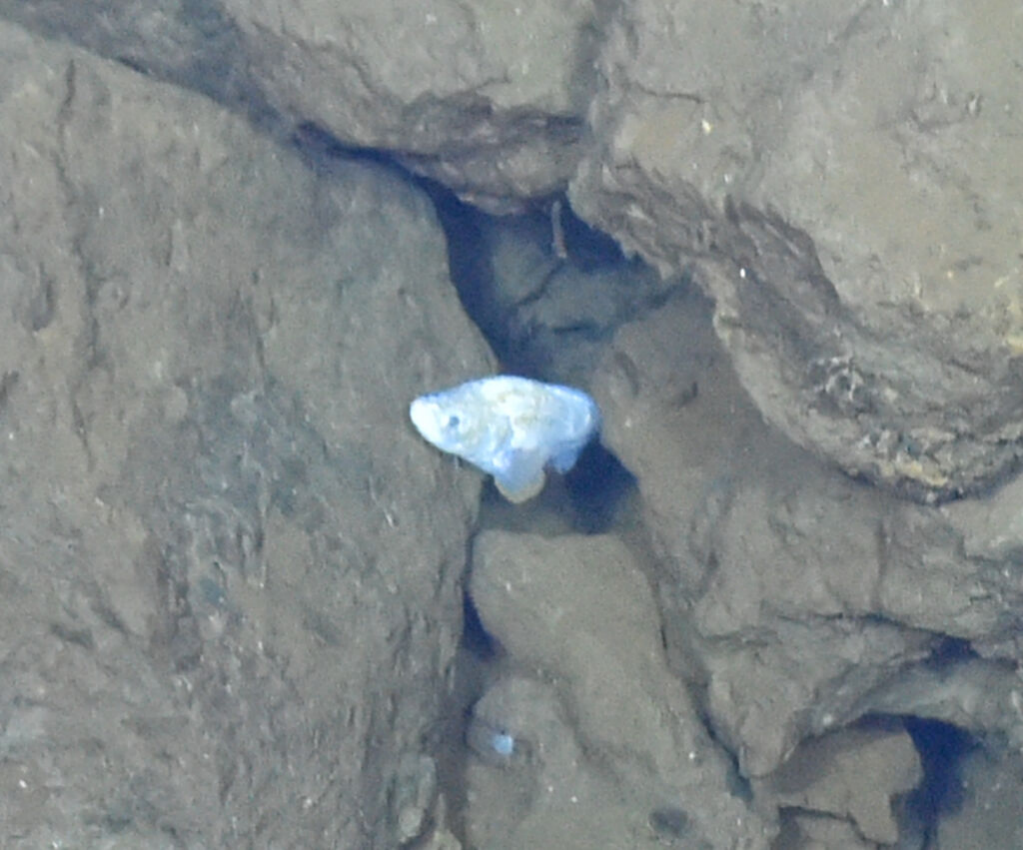
Ophidiidae gen. indet. (DZMB_2021_0014) in situ close to the vent site 6 hydrothermal vent field in Cluster 12 of the INDEX area. Image corresponds with the data (Image attribution: BGR).

**Figure 55.**
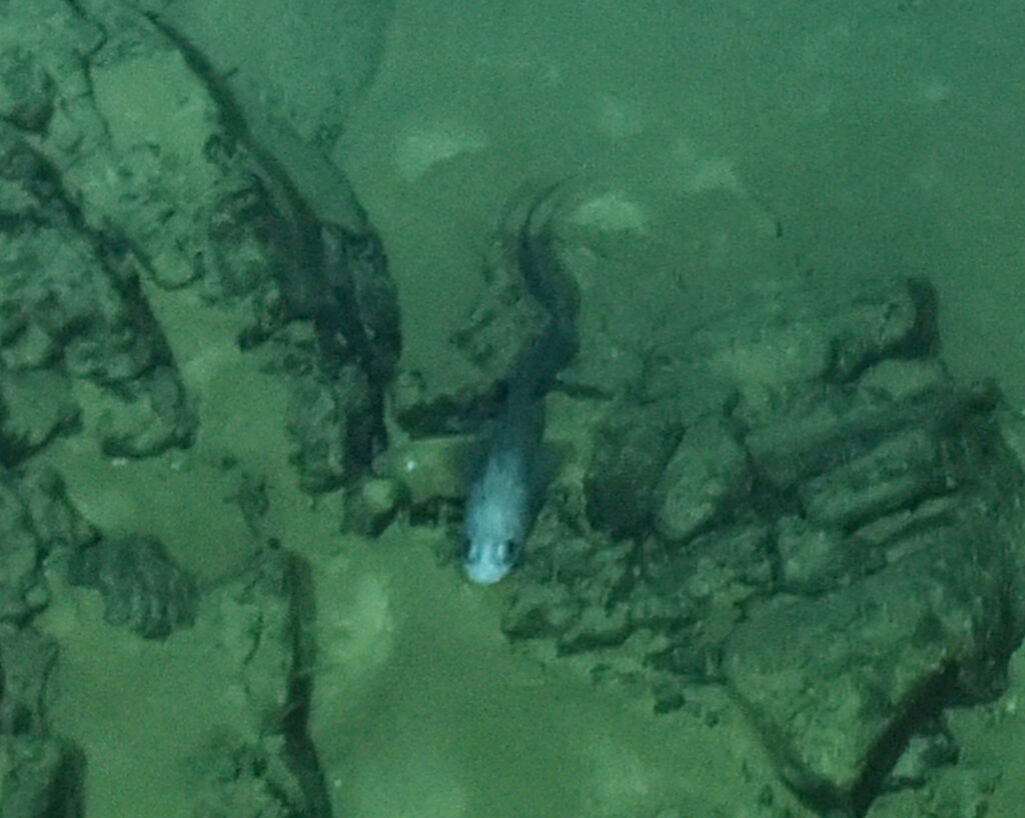
Ophidiidae gen. indet. (DZMB_2021_0015) in situ at the Rodriguez Triple Junction in Cluster 5 of the INDEX area. Image corresponds with the data (Image attribution: BGR).

**Figure 56.**
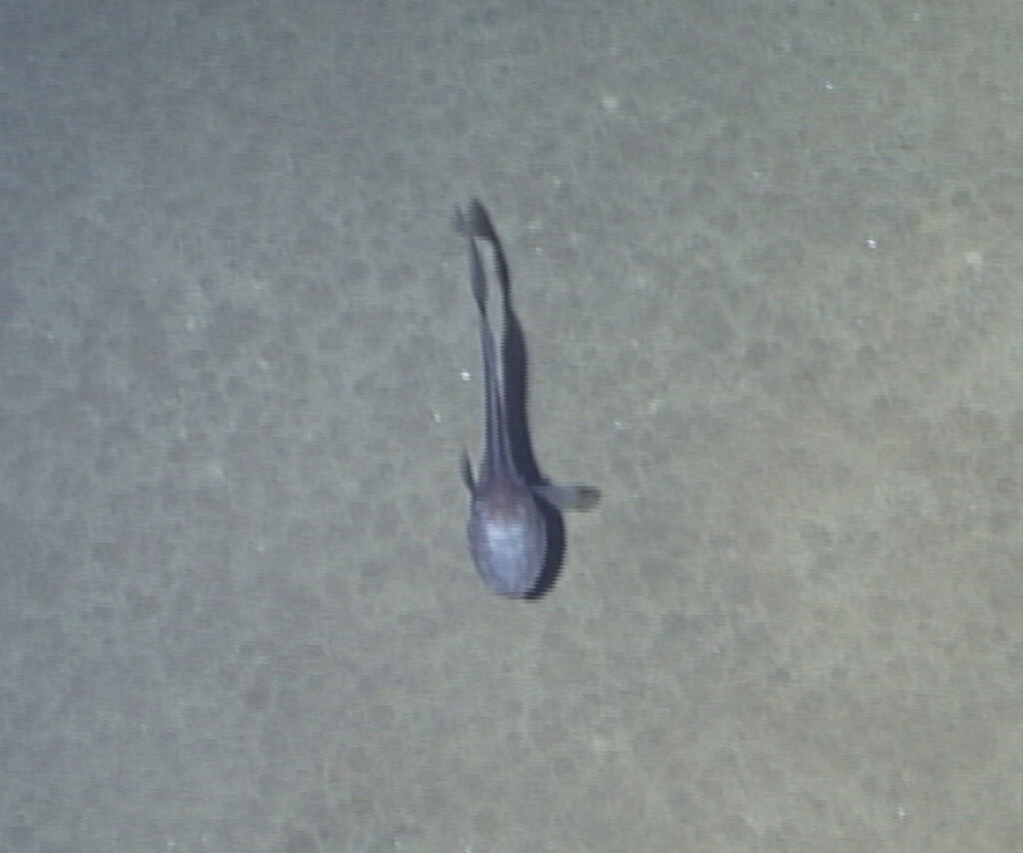
Ophidiidae fam. inc. (DZMB_2021_0016) in situ in the surrounding area of the Kairei hydrothermal vent field in Cluster 5 of the INDEX area. Image corresponds with the data (Image attribution: BGR).

**Figure 57.**
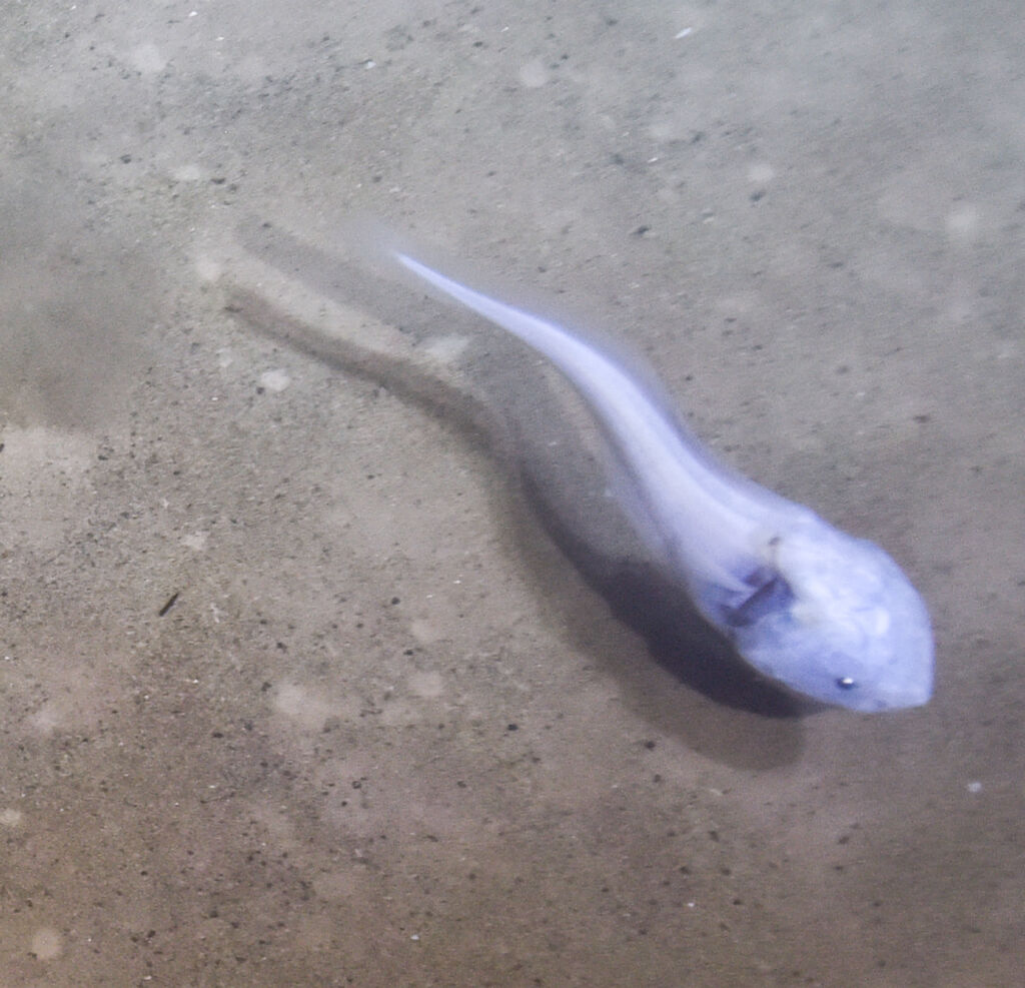
*Acanthonusarmatus* gen. inc. in situ in the surrounding area of the vent site 6 hydrothermal vent field in Cluster 12 of the INDEX area. Image corresponds with the data (Image attribution: BGR).

**Figure 58.**
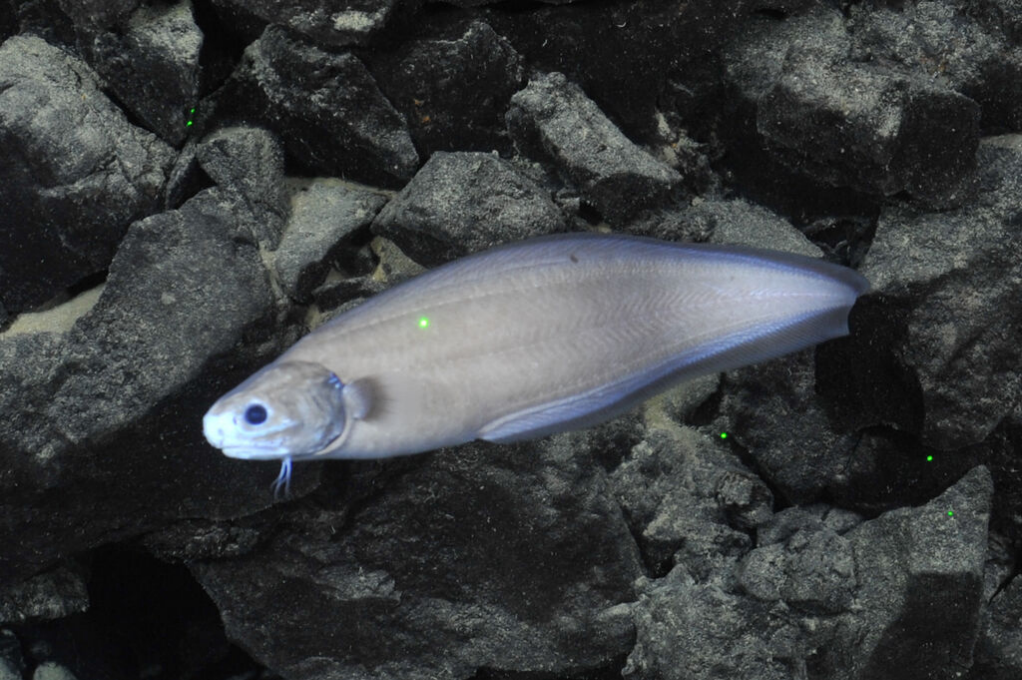
*Barathritesiris* gen. inc. in situ within the vent site 1 area in Cluster 4 of the INDEX area. Image corresponds with the data (Image attribution: BGR).

**Figure 59.**
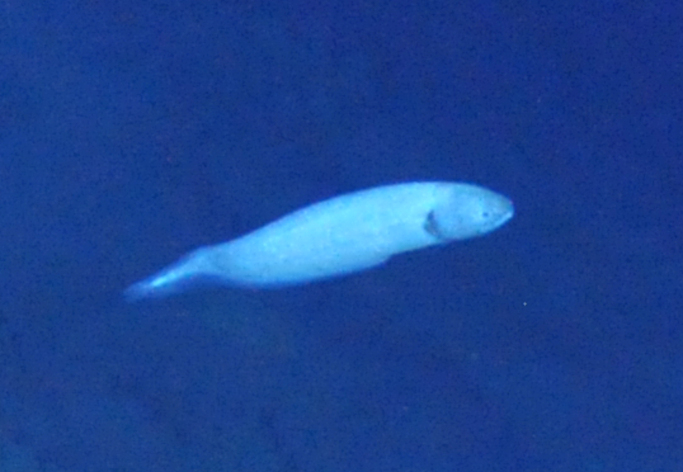
*Bassozetus* gen. inc. in situ in the Edmond-vent site 2-vent site 7 area in Cluster 4 of the INDEX area. Image corresponds with the data (Image attribution: BGR).

**Figure 60.**
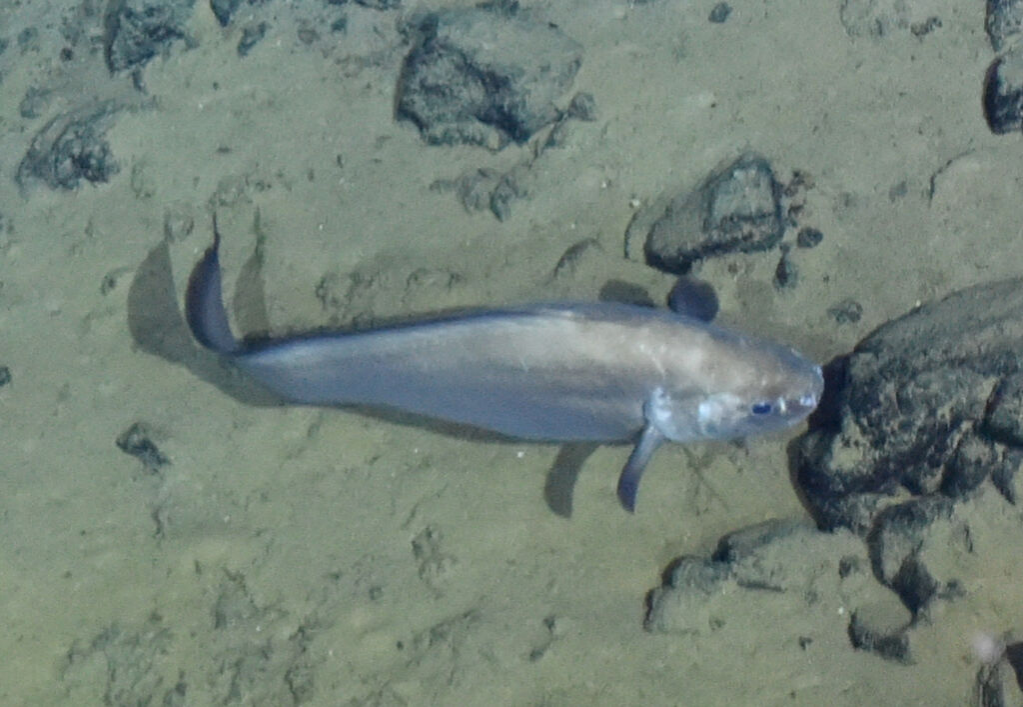
*Spectrunculuscrassus* sp. inc. in situ in the surrounding area of the vent site 6 hydrothermal vent field in Cluster 12 of the INDEX area. Image corresponds with the data (Image attribution: BGR).

**Figure 61.**
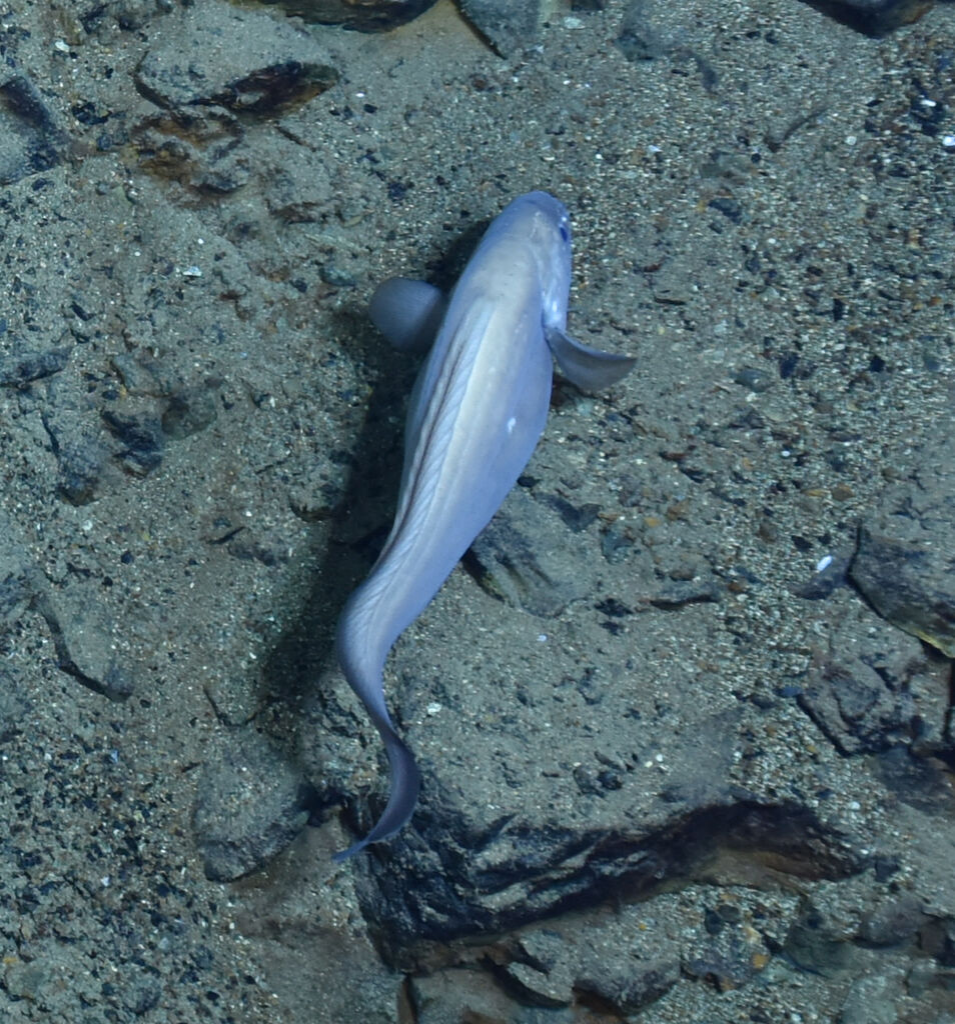
*Spectrunculusgrandis* sp. inc. in situ in the surrounding area of the vent site 5 hydrothermal vent field in Cluster 11 of the INDEX area. Image corresponds with the data (Image attribution: BGR).

**Figure 62.**
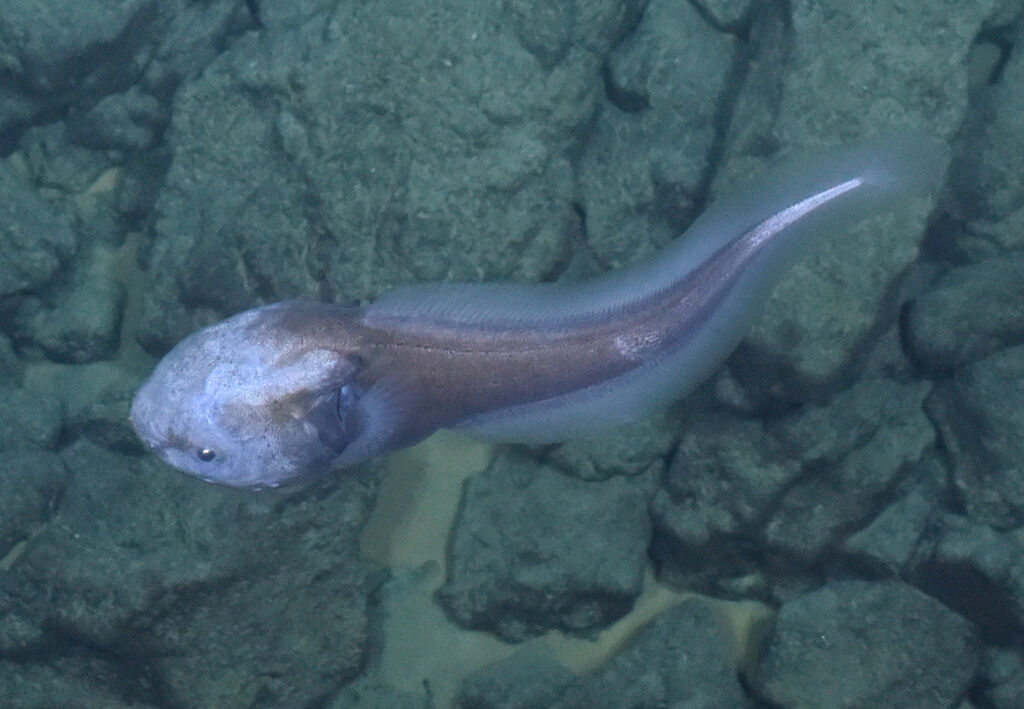
*Xyelacybamyersi* gen. inc. in situ in the area of the Rodriguez Triple Junction in Cluster 5 of the INDEX area. Image corresponds with the data (Image attribution: BGR).

**Figure 63.**
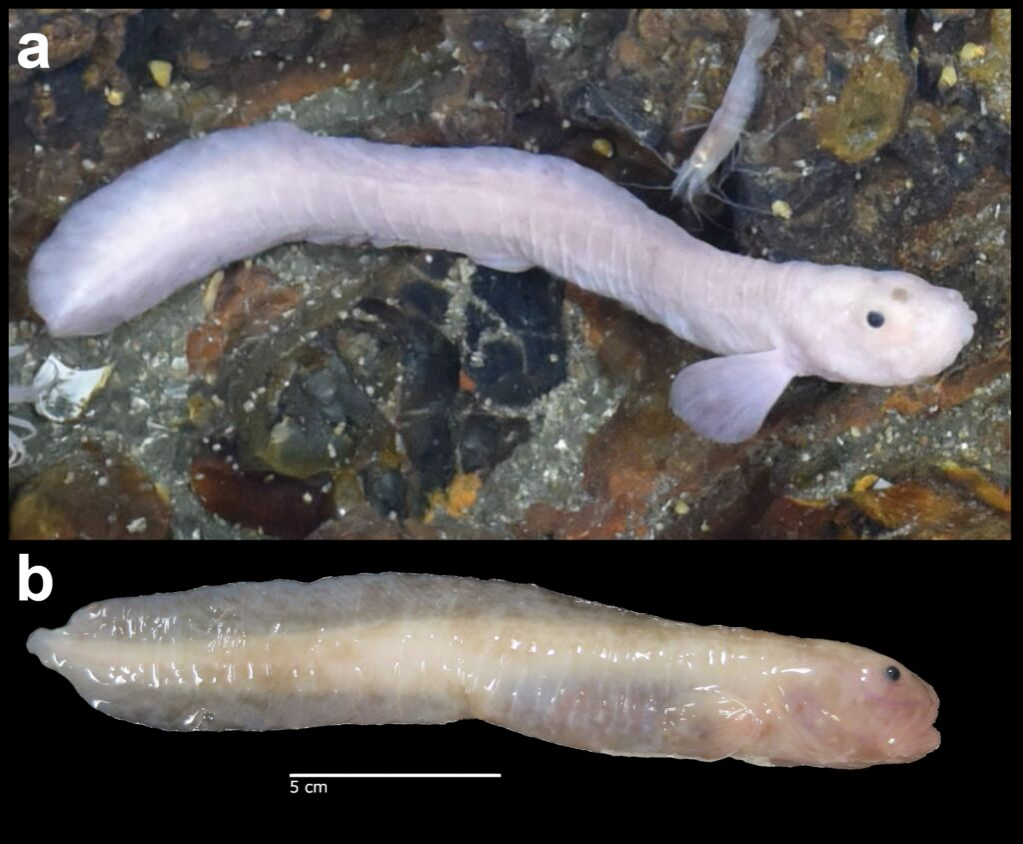
*Pachycaraangeloi* in situ (a) and sampled specimen (b) within the vent site 5 hydrothermal vent field in Cluster 11 of the INDEX area. Image corresponds with the data (Image attribution: BGR).

**Figure 64.**
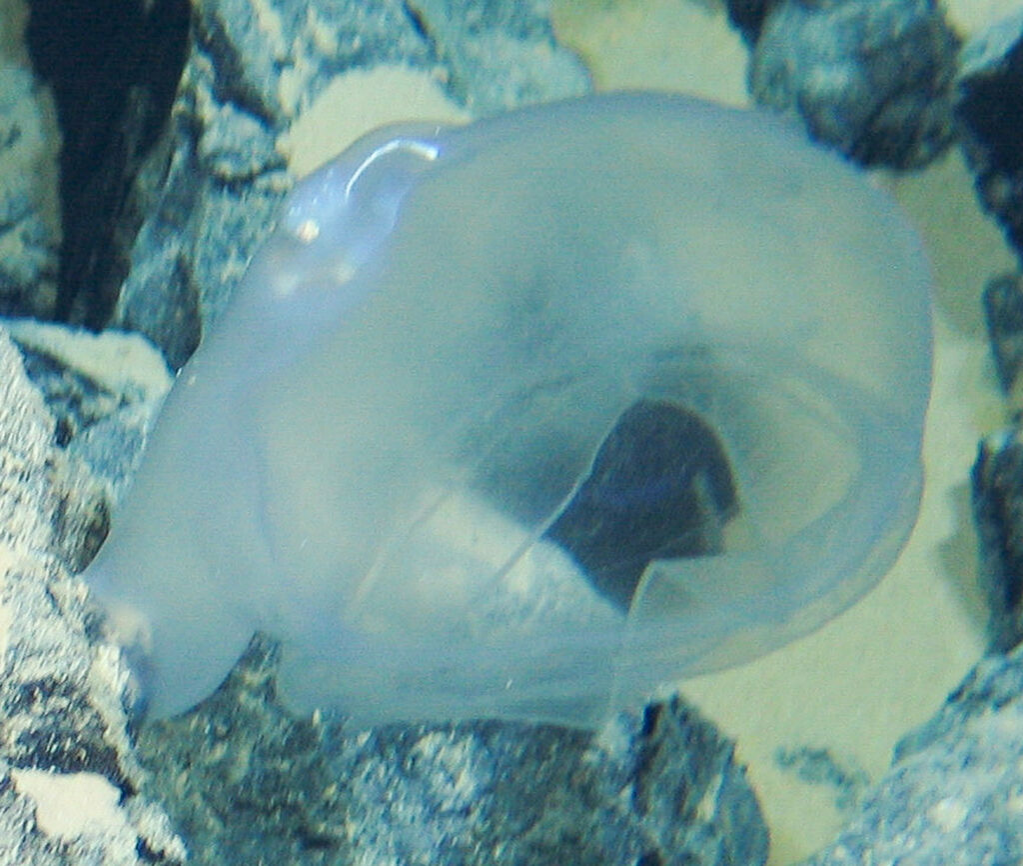
Octacnemidae gen. indet. in situ in the MESO area outside the INDEX area. Image corresponds with the data (Image attribution: BGR and GEOMAR).

**Figure 65.**
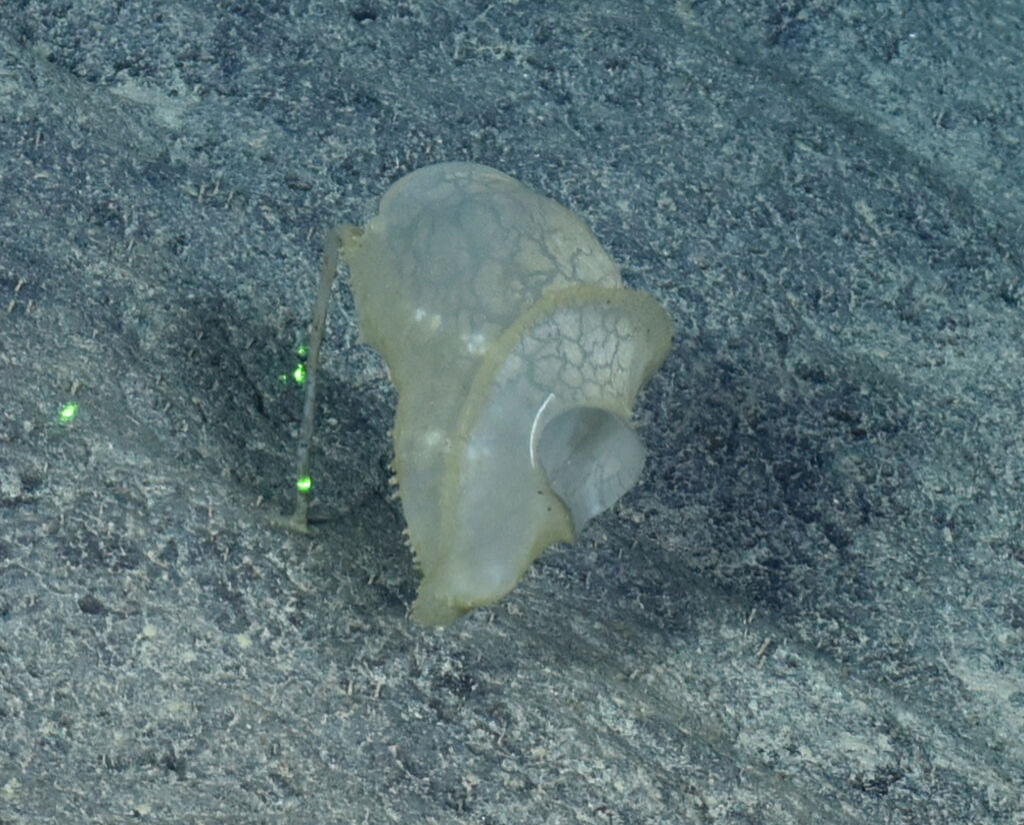
*Culeolus* sp. indet. in situ in the surrounding area of the vent site 4 hydrothermal vent field in Cluster 5 of the INDEX area. The individual is an example for the species complex *Culeolus* spp. indet., with more images and entries in the supplementary imagery and data table. Image corresponds with the data (Image attribution: BGR).

**Figure 66.**
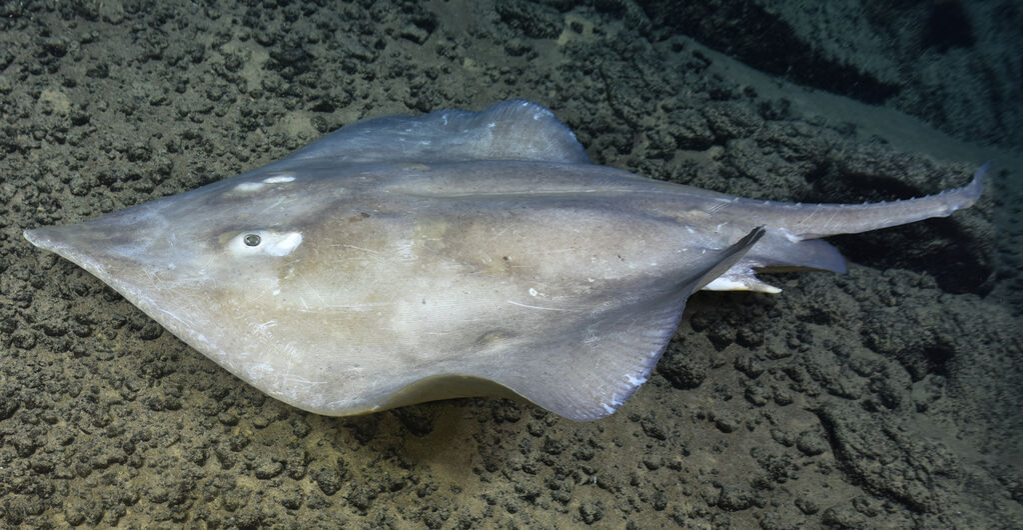
*Bathyrajatunae* sp. inc. in situ in the surrounding area of the vent site 4 hydrothermal vent field in Cluster 5 of the INDEX area. Image corresponds with the data (Image attribution: BGR).

**Figure 67.**
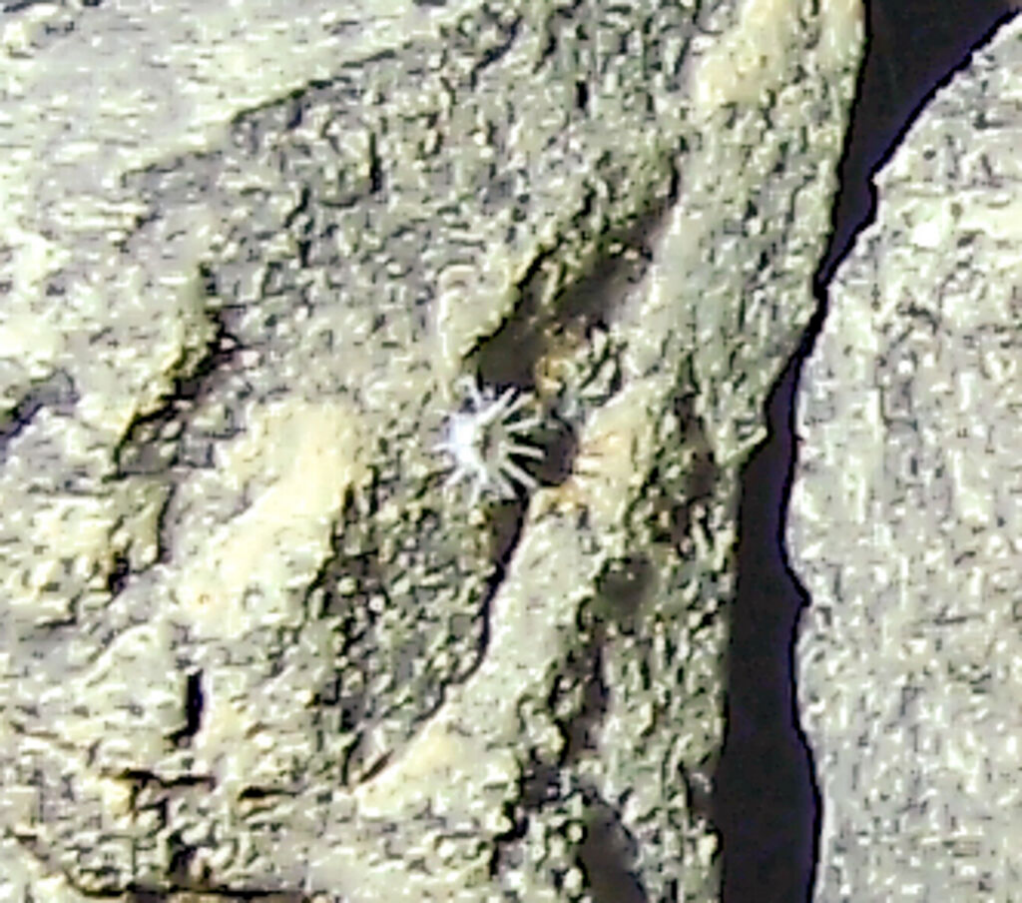
Cnidaria cl. indet. in situ at the South East Indian Ridge in Cluster 6 of the INDEX area. Image corresponds with the data (Image attribution: BGR).

**Figure 68.**
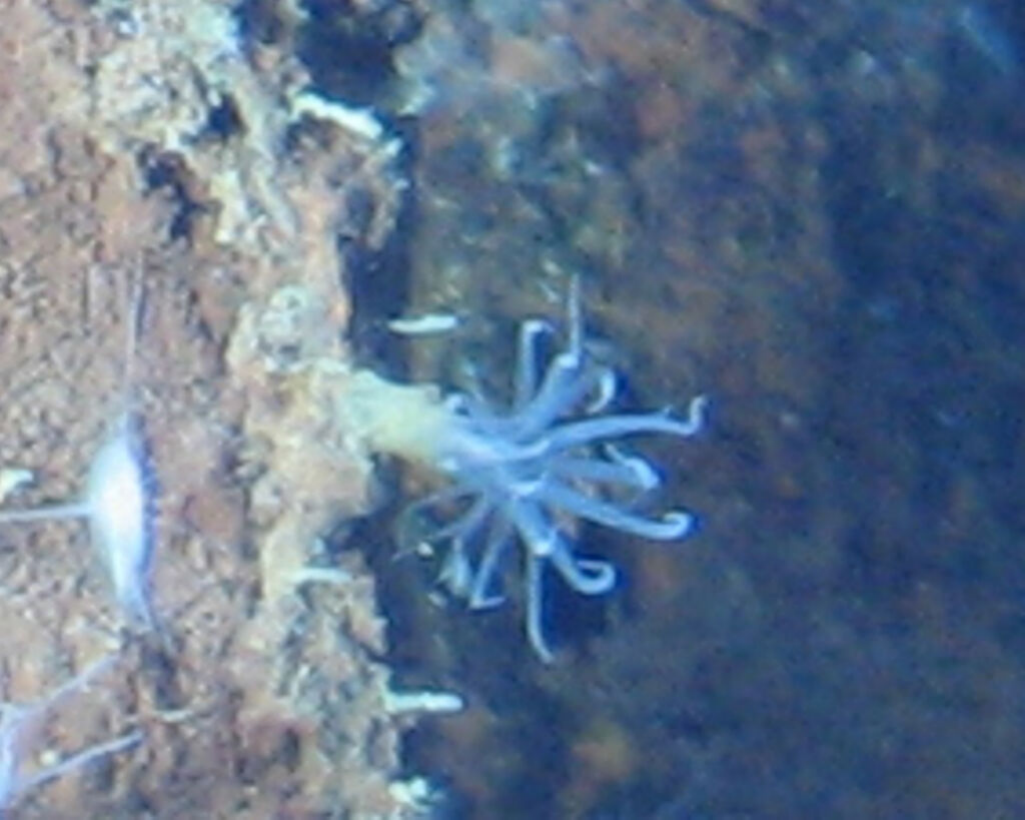
Ceriantharia ord. indet. in situ at the Rodriguez Triple Junction within the Kairei hydrothermal vent field in Cluster 5 of the INDEX area. Image corresponds with the data (Image attribution: BGR).

**Figure 69.**
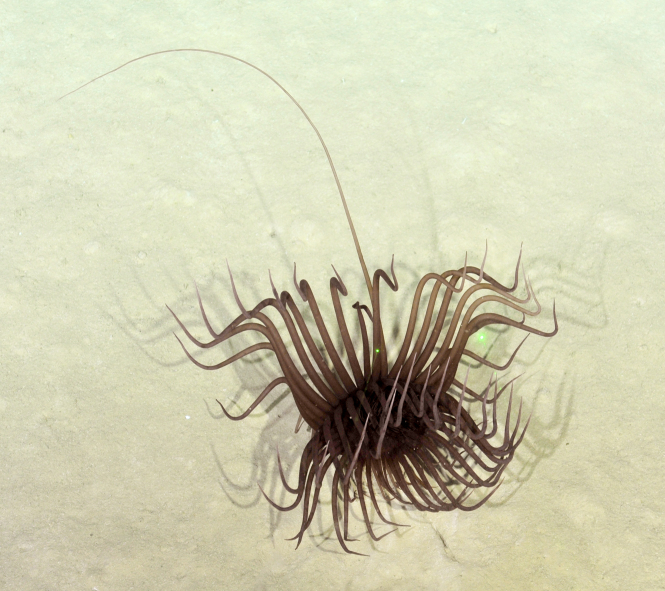
Spirularia fam. indet. in situ at the Central Indian Ridge in the Edmond-Vent site 2-vent site 7 area in Cluster 4 of the INDEX area. Image corresponds with the data (Image attribution: BGR).

**Figure 70.**
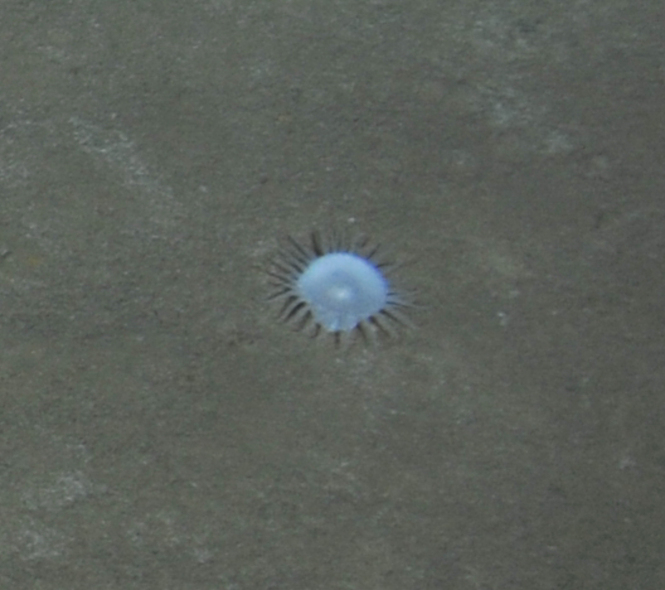
Actiniaria fam. indet. (DZMB_2021_0017) in situ at the Central Indian Ridge in the Edmond-Vent site 2-vent site 7 area in Cluster 4 of the INDEX area. Image corresponds with the data below (Image attribution: BGR).

**Figure 71.**
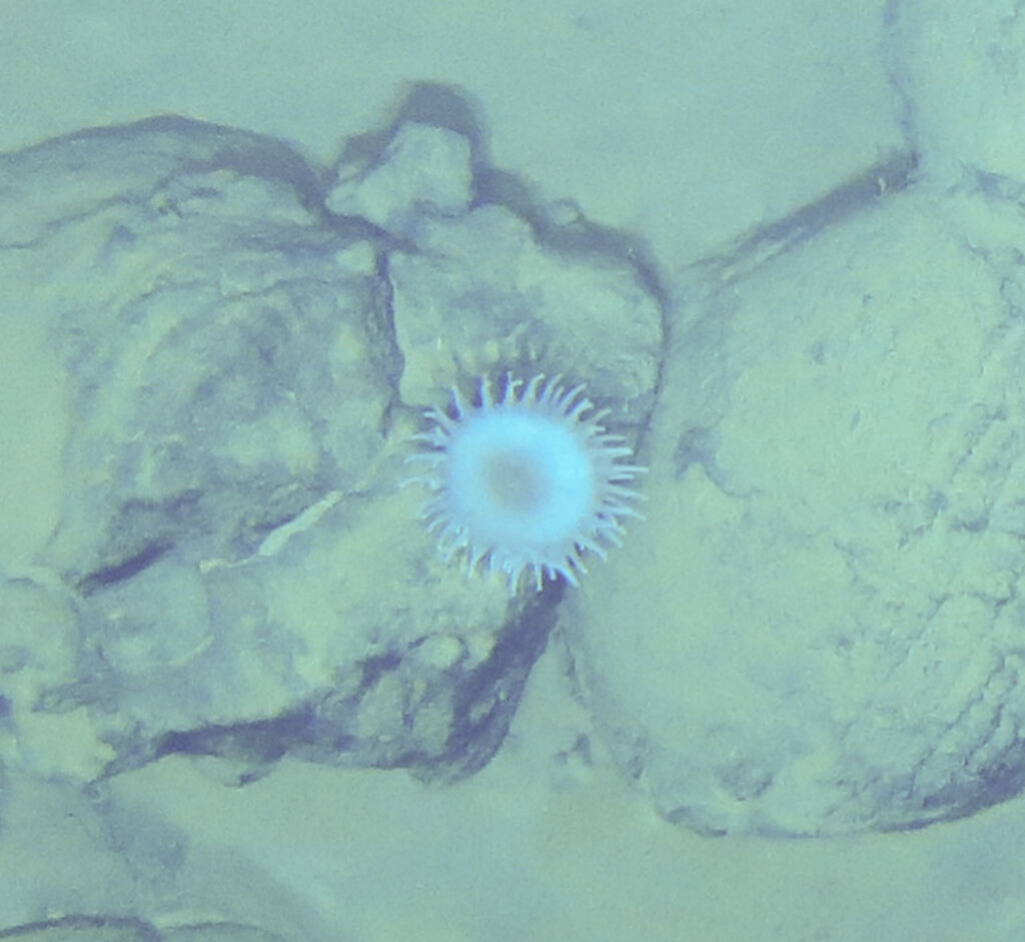
Actiniaria fam. indet. (DZMB_2021_0018) in situ at the South East Indian Ridge in Cluster 11 of the INDEX area. Image corresponds with the data (Image attribution: BGR).

**Figure 72.**
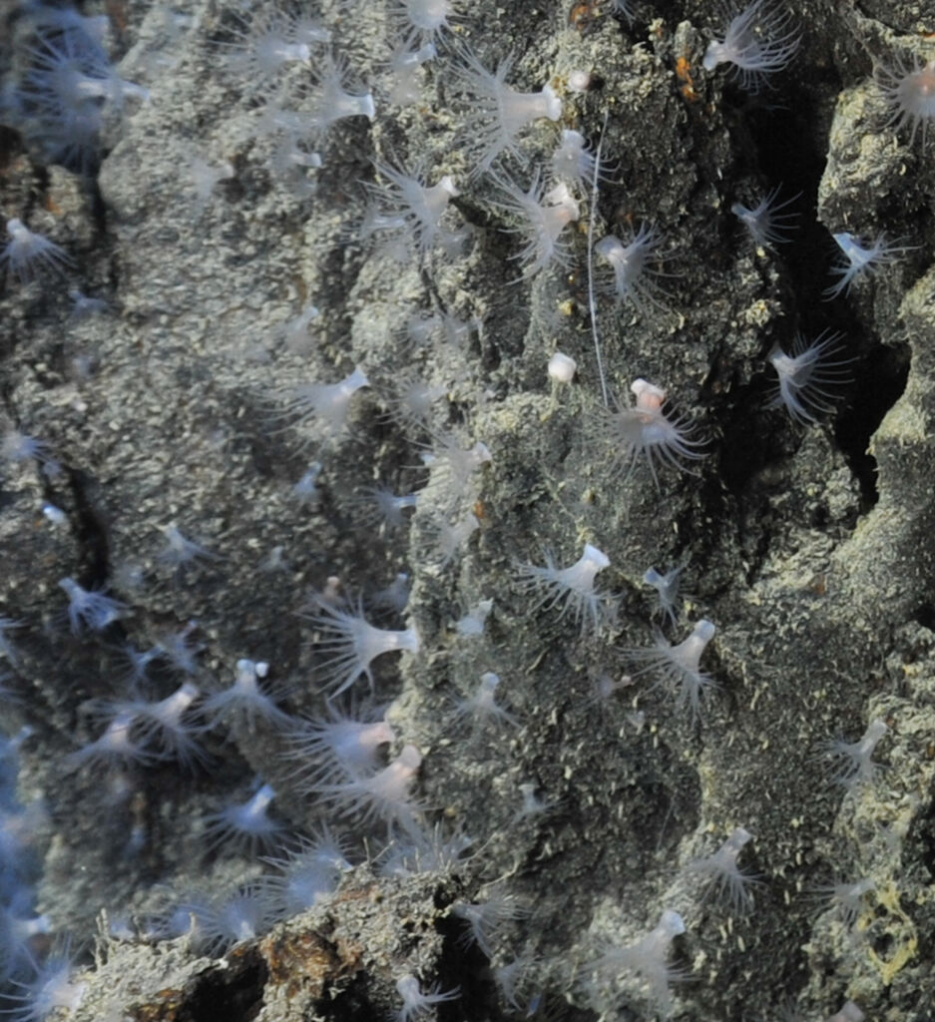
Actiniaria fam. indet. (DZMB_2021_0019) in situ at the Central Indian Ridge within Vent site 1 in Cluster 4 of the INDEX area. Image corresponds with the data (Image attribution: BGR).

**Figure 73.**
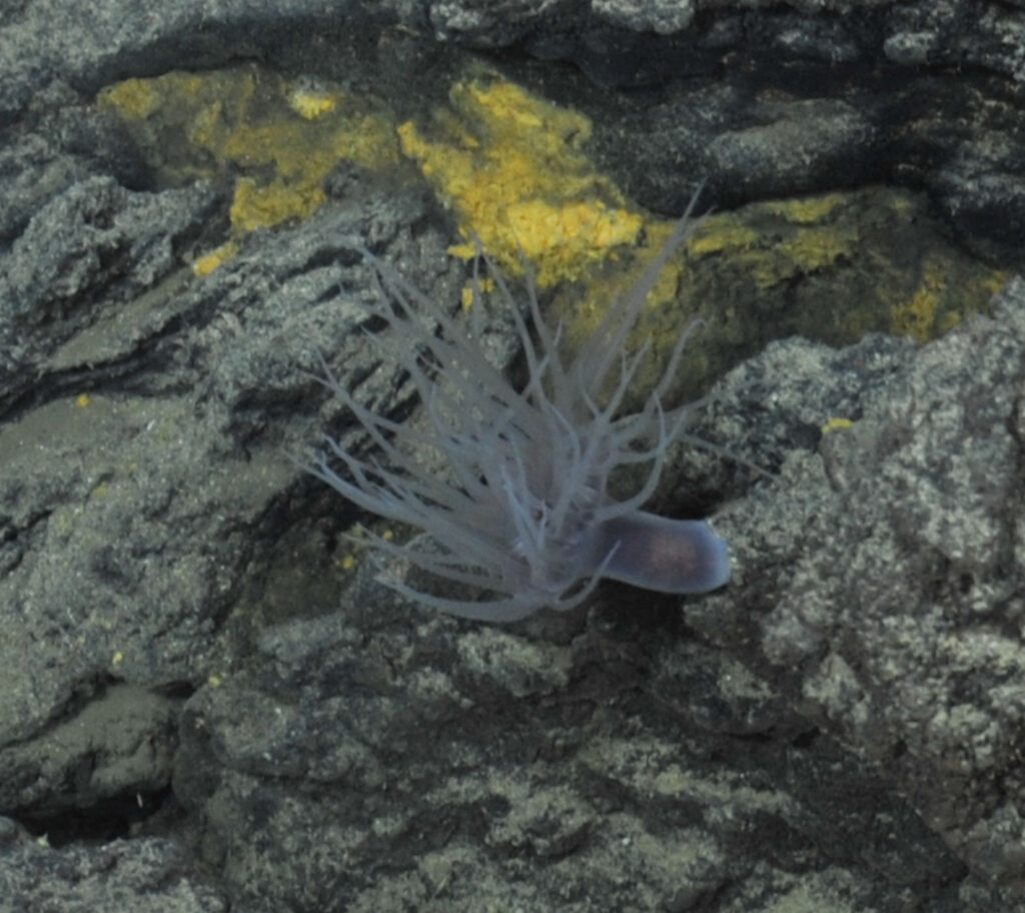
Actiniaria fam. indet. (DZMB_2021_0020) in situ at the Central Indian Ridge within Vent site 1 in Cluster 4 of the INDEX area. Image corresponds with the data (Image attribution: BGR).

**Figure 74.**
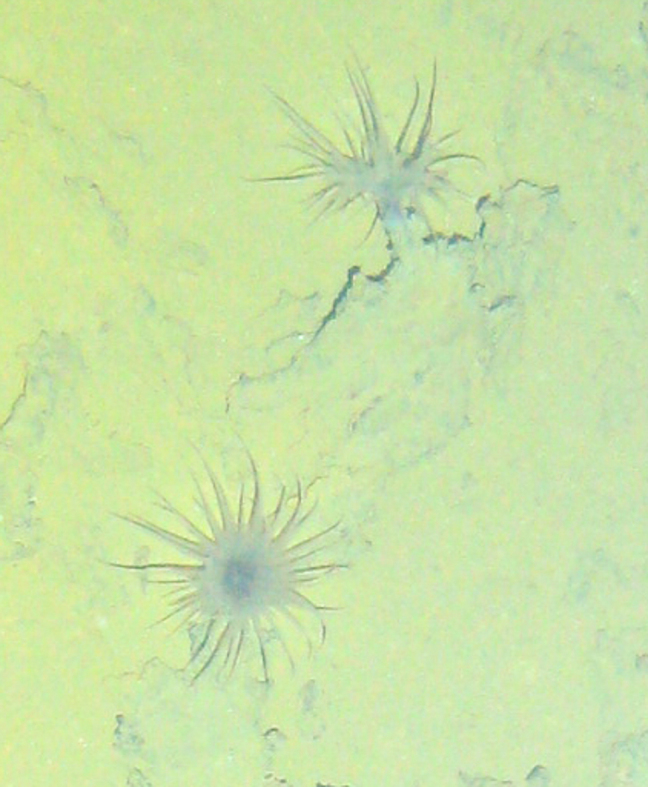
Actiniaria fam. indet. (DZMB_2021_0021) in situ at the Central Indian Ridge within the Edmond hydrothermal vent field in Cluster 4 of the INDEX area. Image corresponds with the data (Image attribution: BGR).

**Figure 75.**
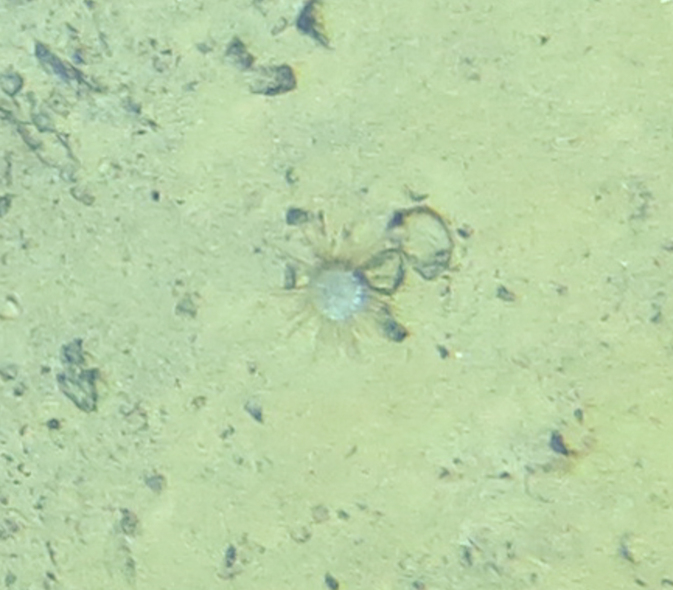
Actiniaria fam. indet. (DZMB_2021_0022) in situ at the South East Indian Ridge in Cluster 11 of the INDEX area. Image corresponds with the data (Image attribution: BGR).

**Figure 76.**
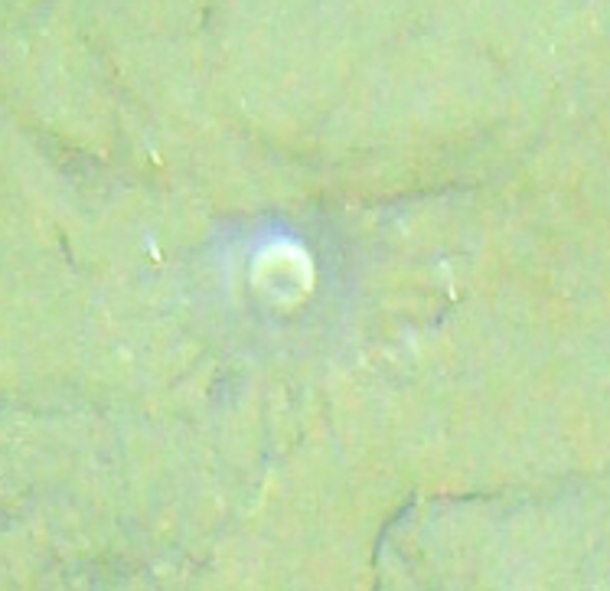
Actiniaria fam. indet. (DZMB_2021_0023) in situ at the Central Indian Ridge within the Edmond hydrothermal vent field in Cluster 4 of the INDEX area. Image corresponds with the data (Image attribution: BGR).

**Figure 77.**
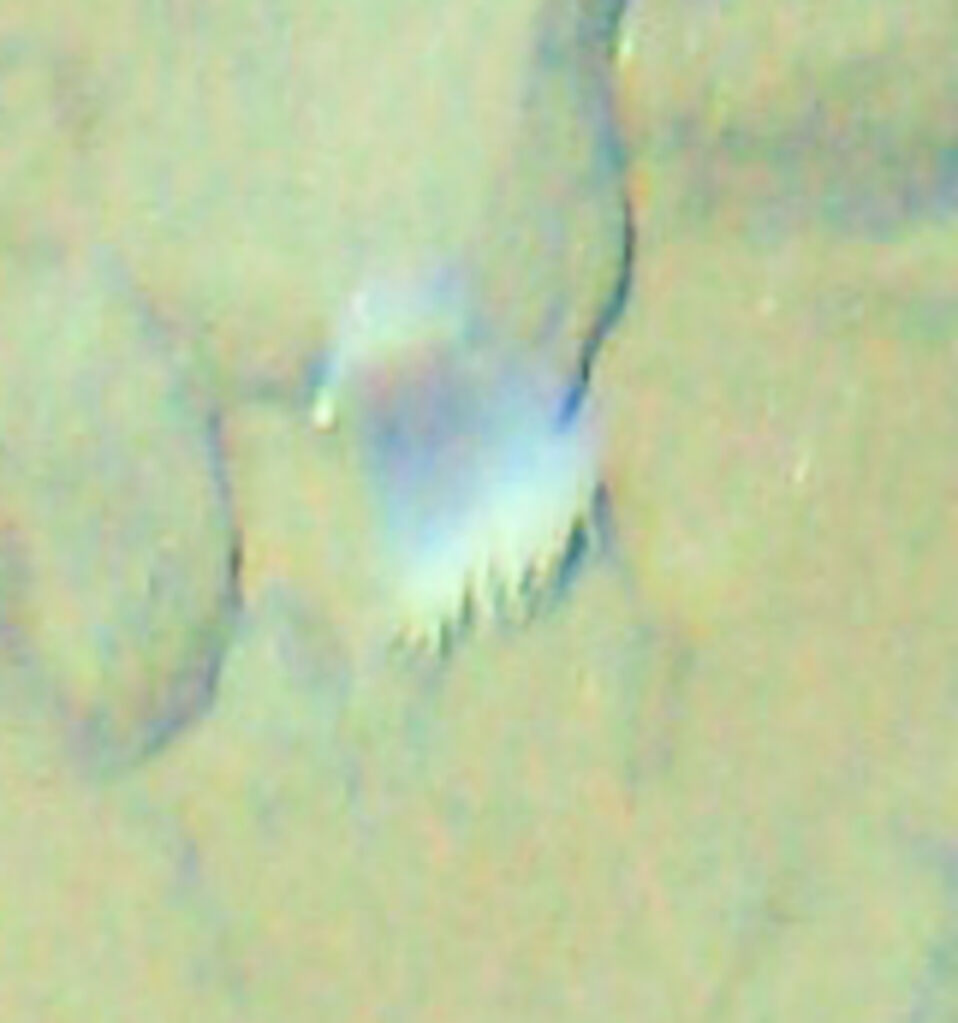
Actiniaria fam. indet. (DZMB_2021_0024) in situ at the Central Indian Ridge within the Edmond hydrothermal vent field in Cluster 4 of the INDEX area. Image corresponds with the data (Image attribution: BGR).

**Figure 78.**
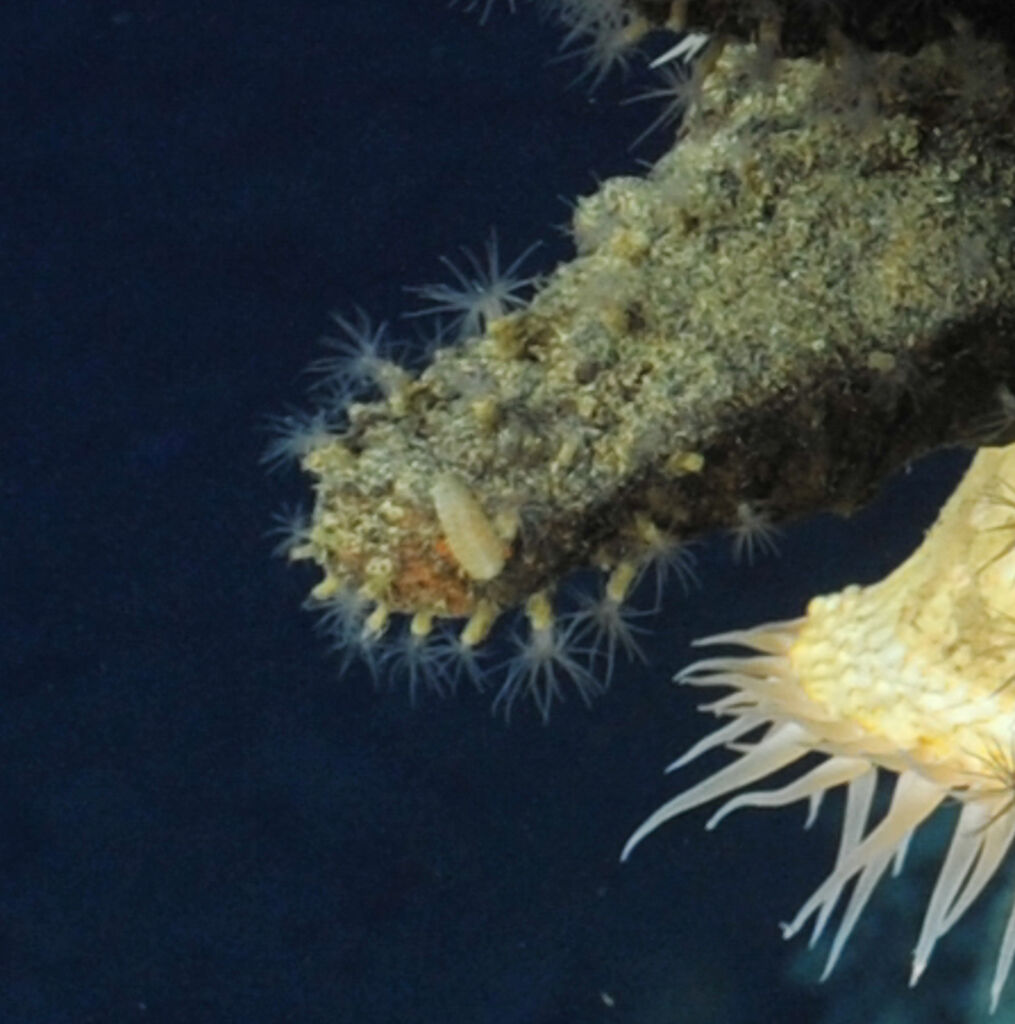
Actiniaria fam. indet. (DZMB_2021_0025) in situ at the Central Indian Ridge within Vent site 1 in Cluster 4 of the INDEX area. Image corresponds with the data (Image attribution: BGR).

**Figure 79.**
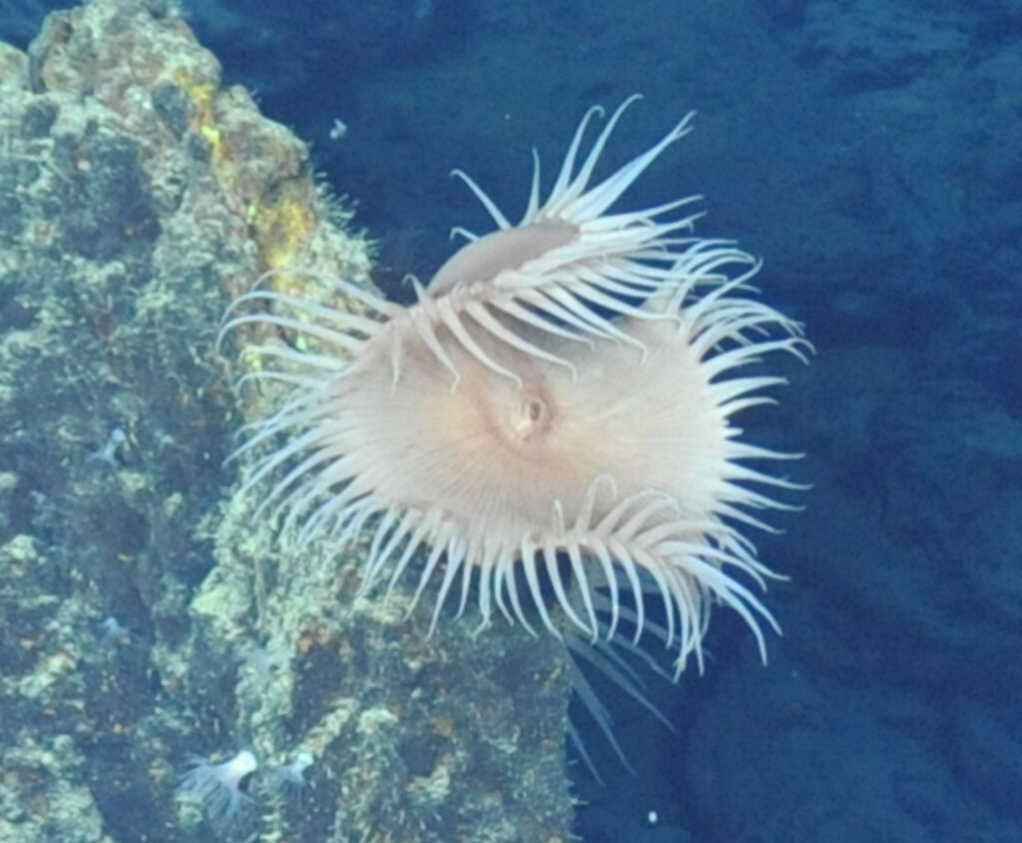
Actinoscyphiidae gen. indet. (DZMB_2021_0026) in situ at the Central Indian Ridge within Vent site 1 in Cluster 4 of the INDEX area. Image corresponds with the data (Image attribution: BGR).

**Figure 80.**
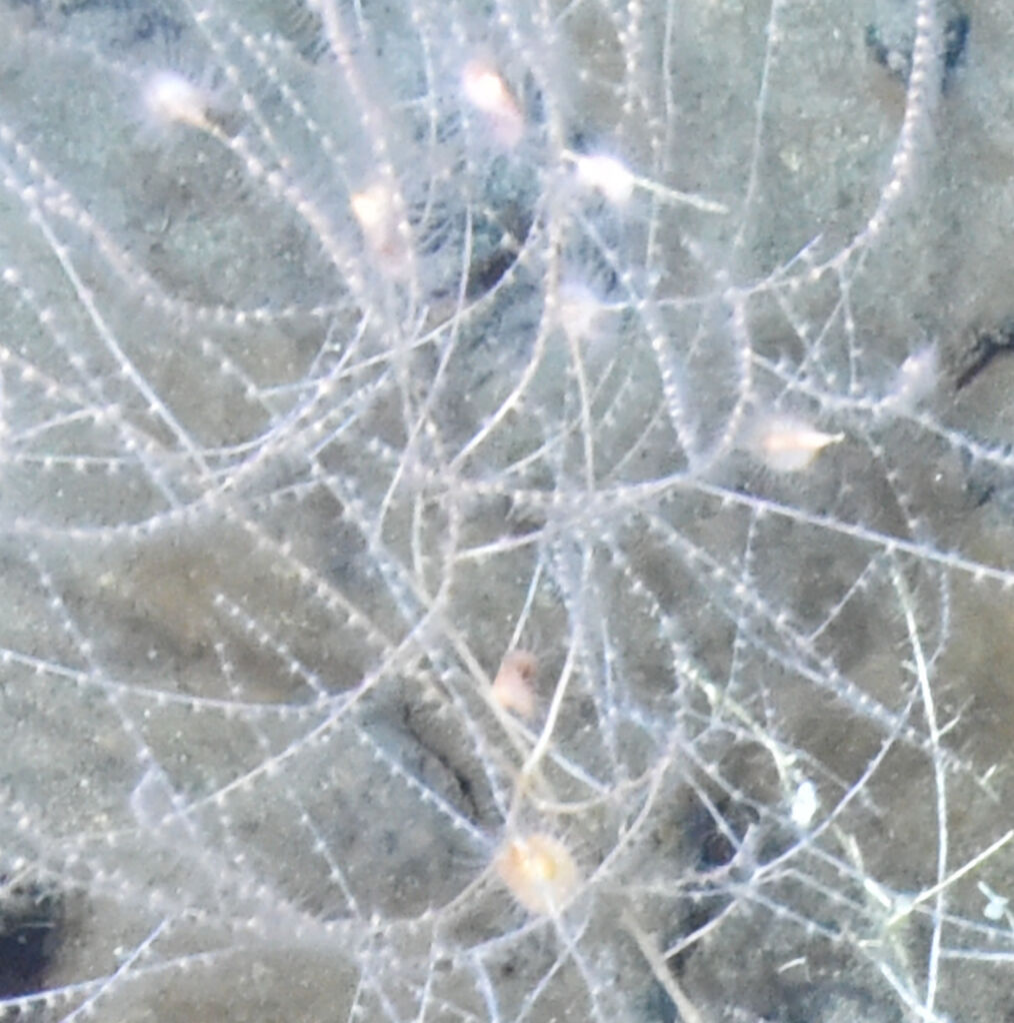
Actinoscyphiidae gen. indet. (DZMB_2021_0027) in situ at the South East Indian Ridge within Vent site 6 in Cluster 12 of the INDEX area. Image corresponds with the data (Image attribution: BGR).

**Figure 81.**
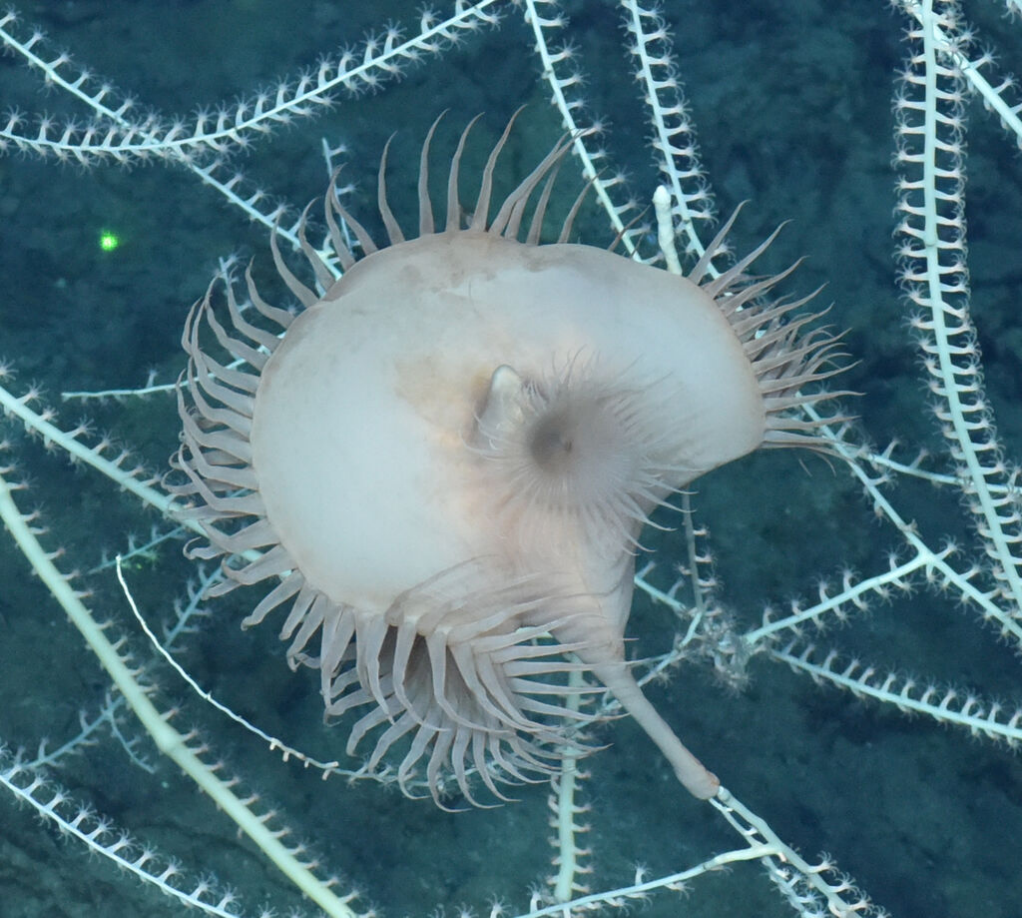
*Actinoscyphia* sp. indet. in situ at the Rodriguez Triple Junction within Vent site 4 in Cluster 5 of the INDEX area. Image corresponds with the data (Image attribution: BGR).

**Figure 82.**
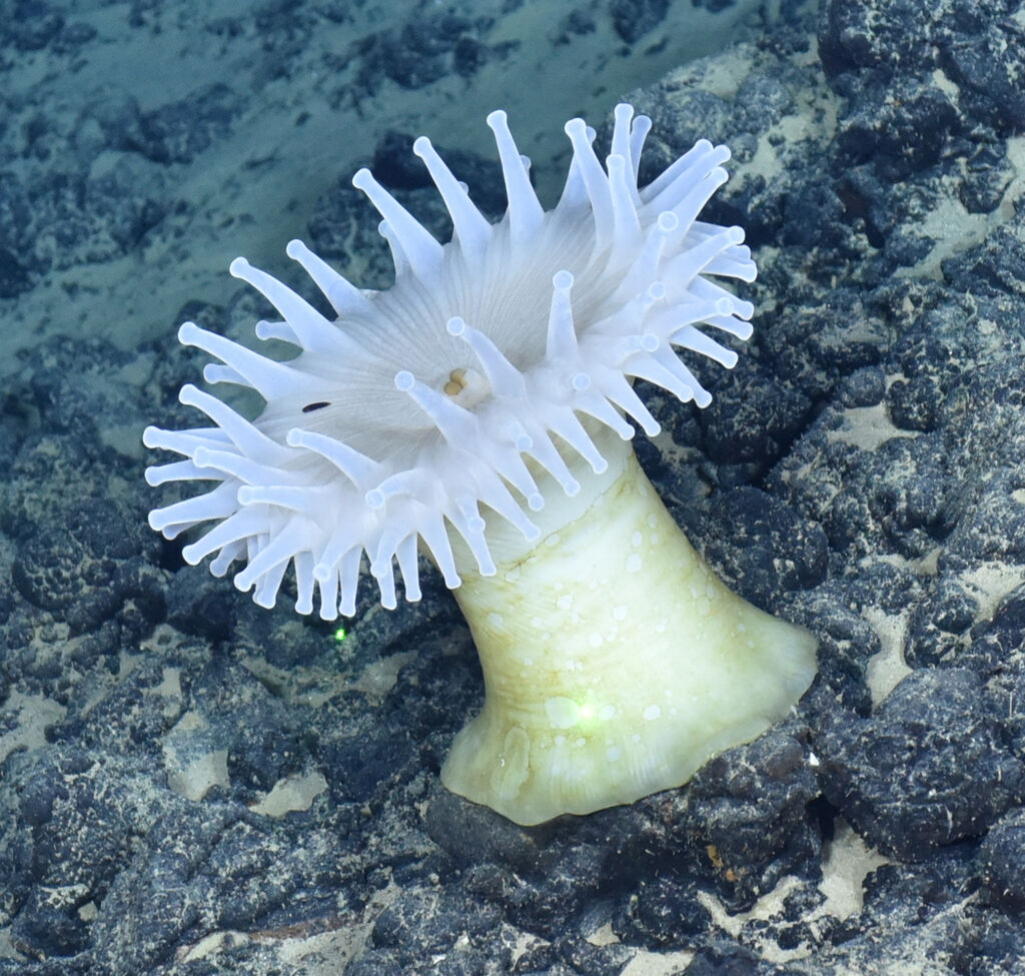
Actinostolidae gen. indet. in situ at the South East Indian Ridge within Vent site 6 in Cluster 12 of the INDEX area. Image corresponds with the data (Image attribution: BGR).

**Figure 83.**
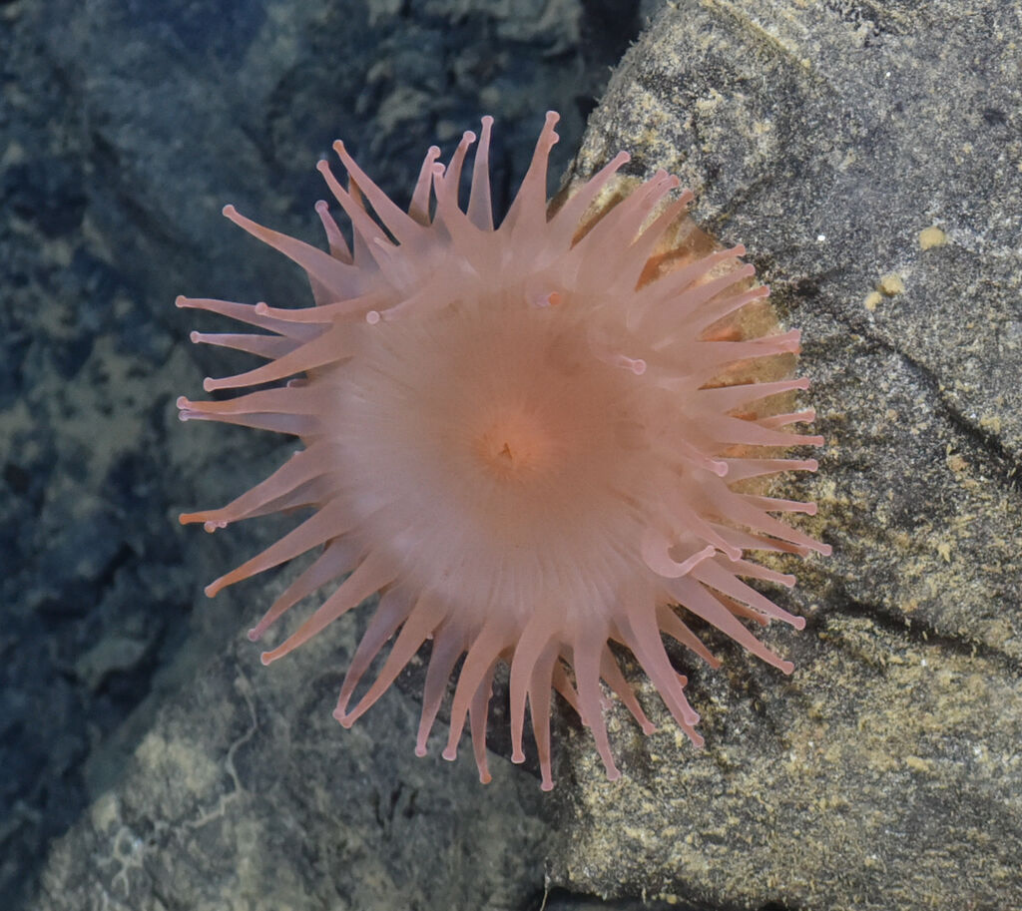
*Actinostola* sp. indet. (DZMB_2021_0028) in situ at the South East Indian Ridge within Vent site 5 in Cluster 11 of the INDEX area. Image corresponds with the data (Image attribution: BGR).

**Figure 84.**
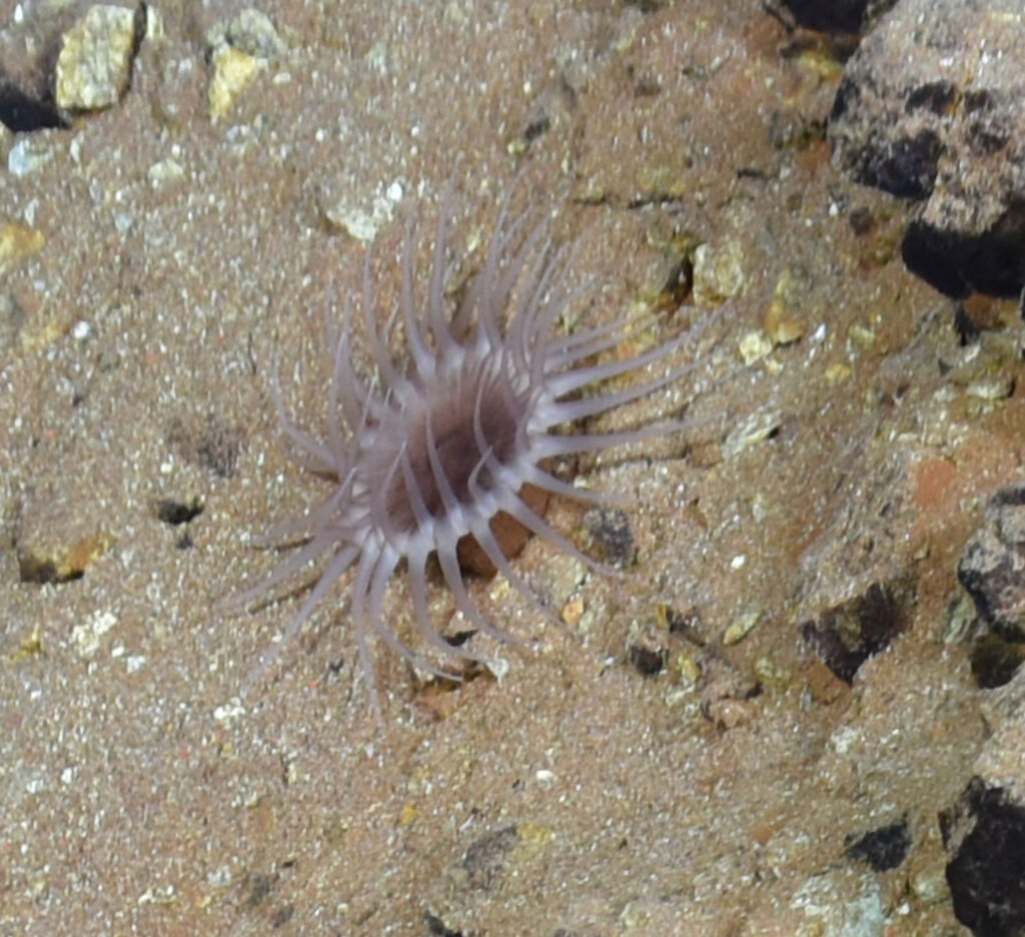
*Actinostola* sp. indet. (DZMB_2021_0029) in situ at the South East Indian Ridge within the Vent site 5 in Cluster 11 of the INDEX area. Image corresponds with the data (Image attribution: BGR).

**Figure 85.**
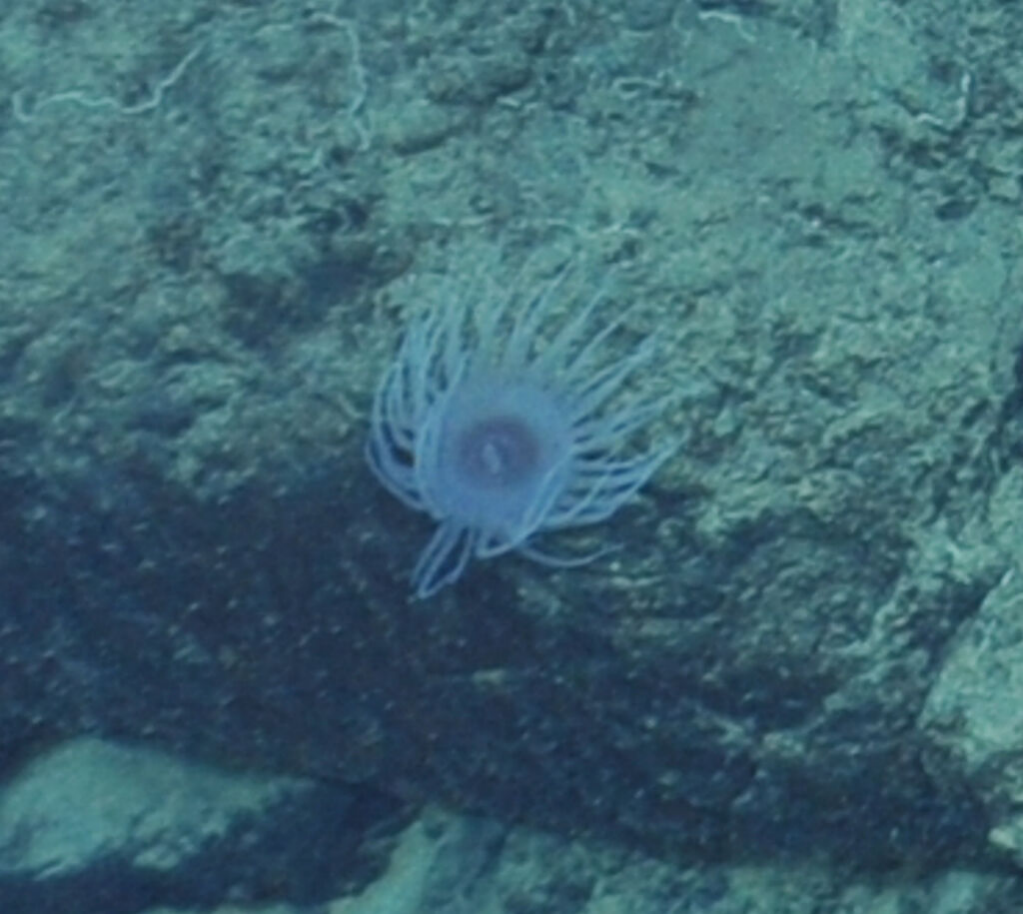
*Actinostola* sp. indet. (DZMB_2021_0030) in situ at the Central Indian Ridge within Vent site 1 in Cluster 4 of the INDEX area. Image corresponds with the data (Image attribution: BGR).

**Figure 86.**
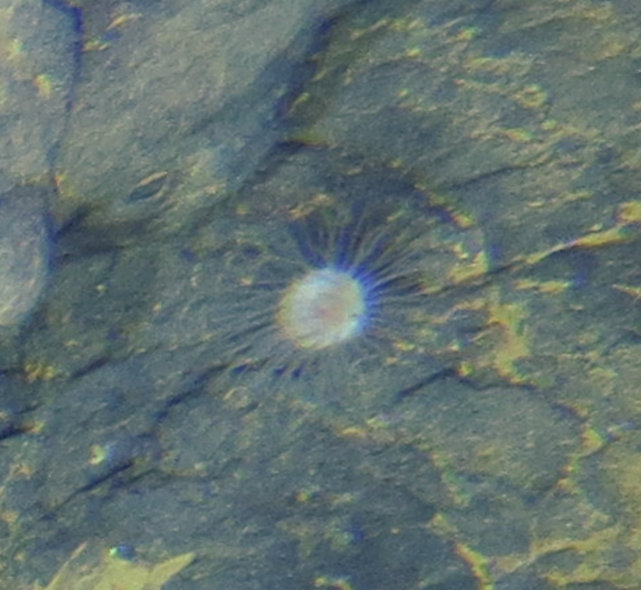
*Actinostola* sp. indet. (DZMB_2021_0031) in situ at the South East Indian Ridge within Vent site 5 in Cluster 11 of the INDEX area. Image corresponds with the data (Image attribution: BGR).

**Figure 87.**
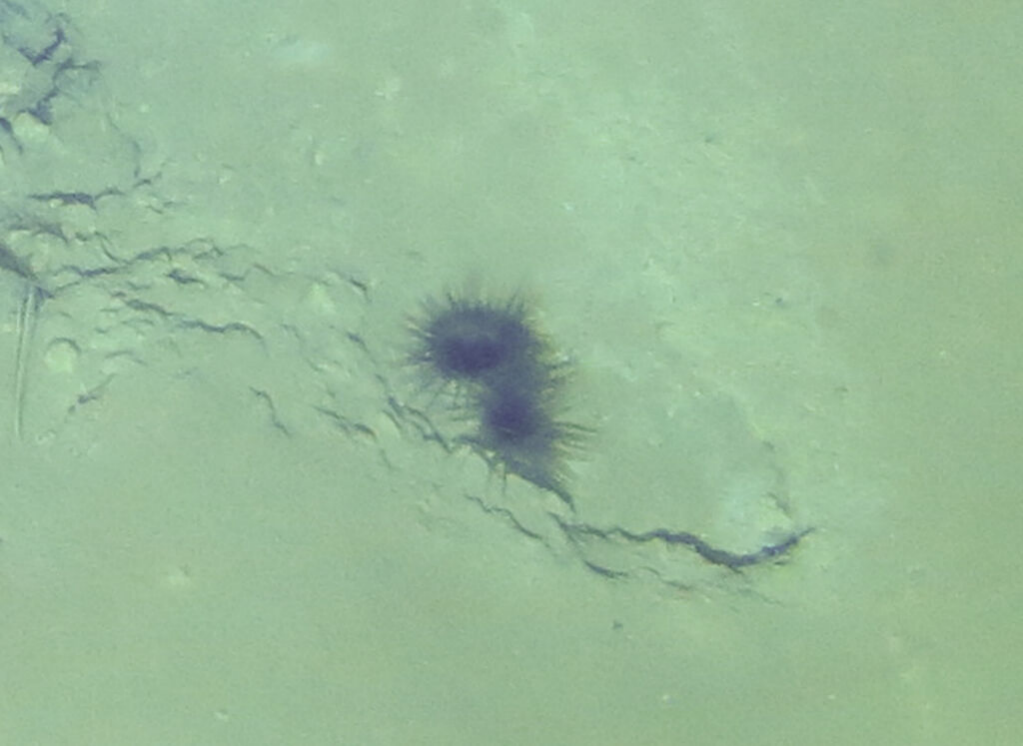
*Bathyphellia* sp. indet. (DZMB_2021_0032) in situ at the South East Indian Ridge in Cluster 11 of the INDEX area. Image corresponds with the data (Image attribution: BGR).

**Figure 88.**
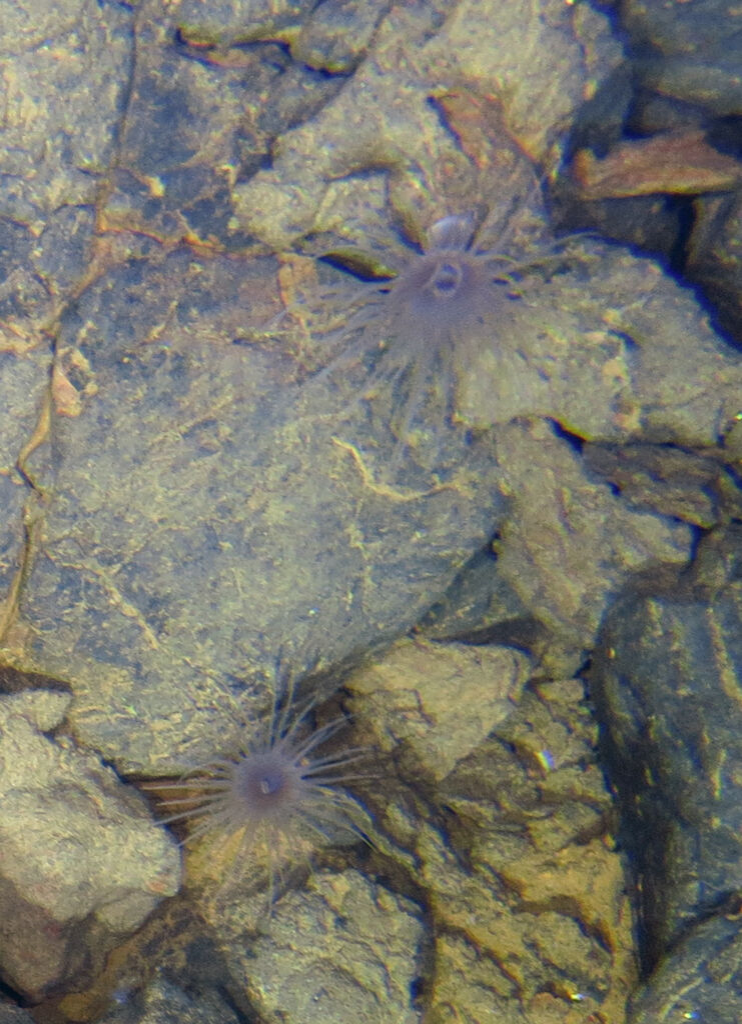
*Bathyphellia* sp. indet. (DZMB_2021_0033) in situ at the South East Indian Ridge within Vent site 5 in Cluster 11 of the INDEX area. Image corresponds with the data (Image attribution: BGR).

**Figure 89.**
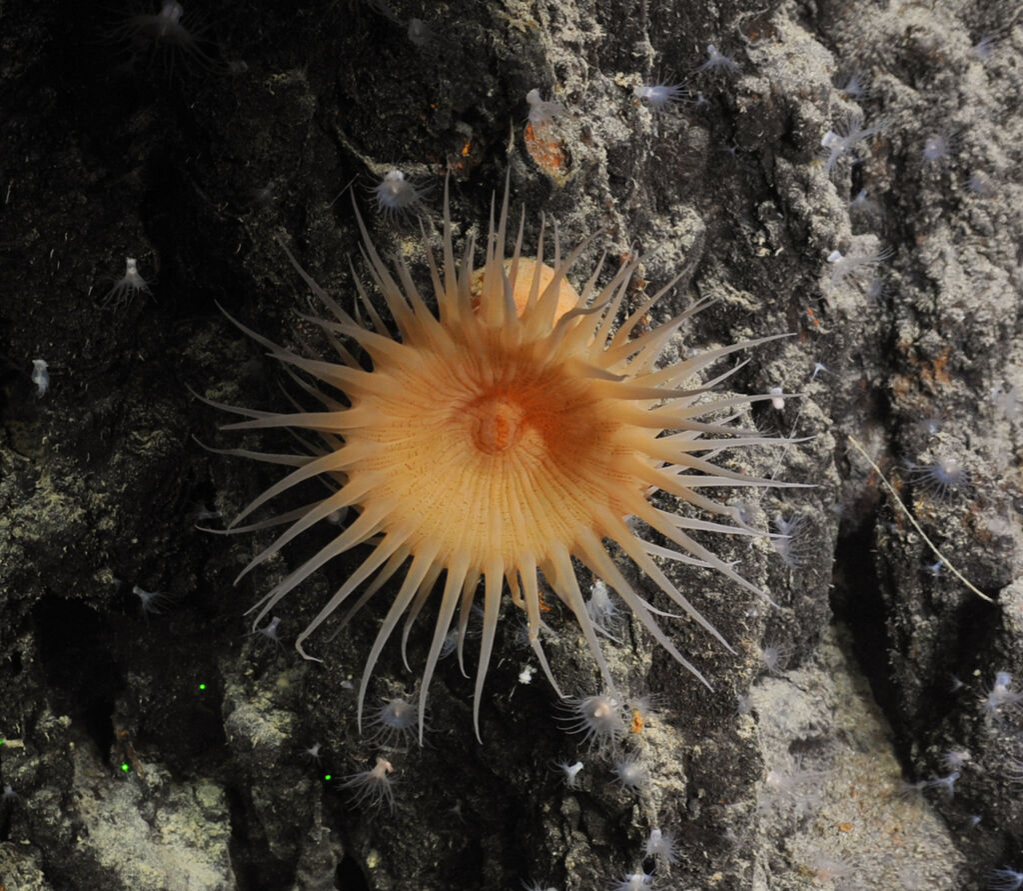
*Chondrophellia* sp. indet. in situ at the Central Indian Ridge within Vent site 1 in Cluster 4 of the INDEX area. Image corresponds with the data (Image attribution: BGR).

**Figure 90.**
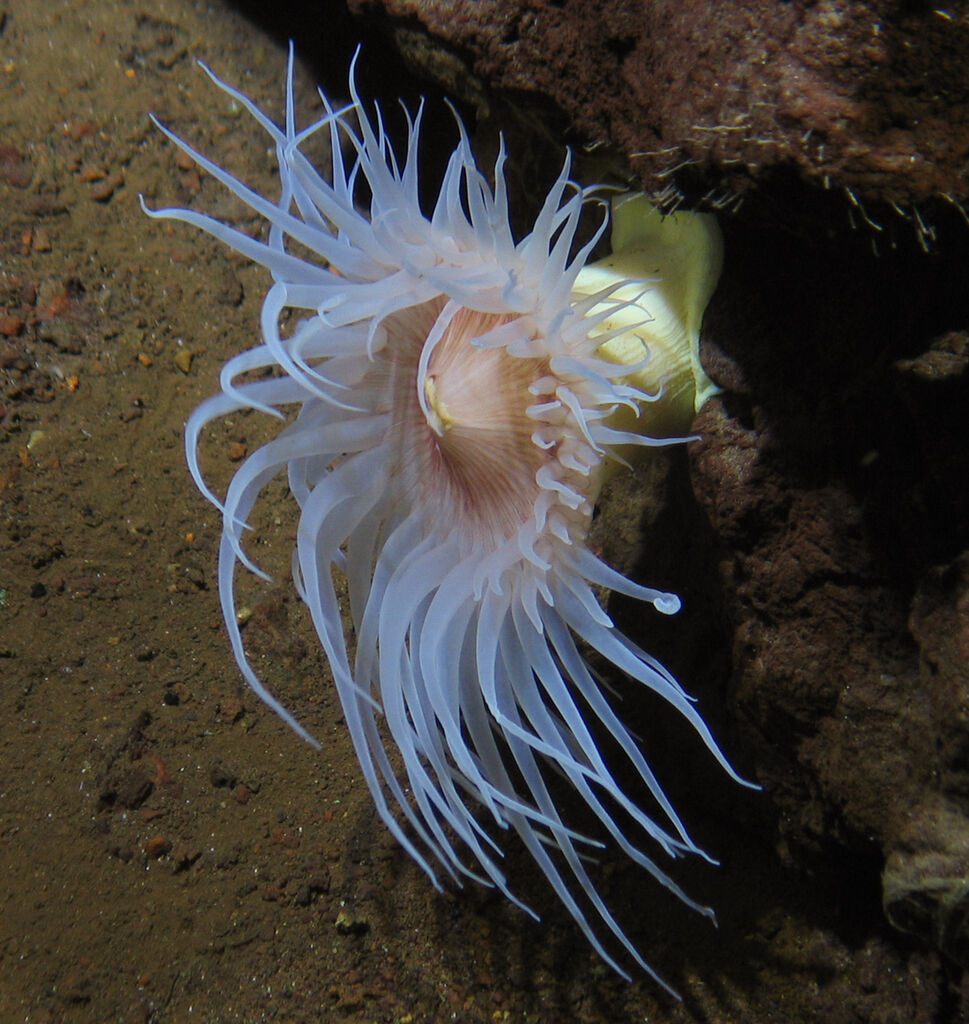
*Maractis* sp. indet. in situ at the Rodriguez Triple Junction within the Kairei hydrothermal vent field in Cluster 5 of the INDEX area. Image corresponds with the data (Image attribution: BGR).

**Figure 91.**
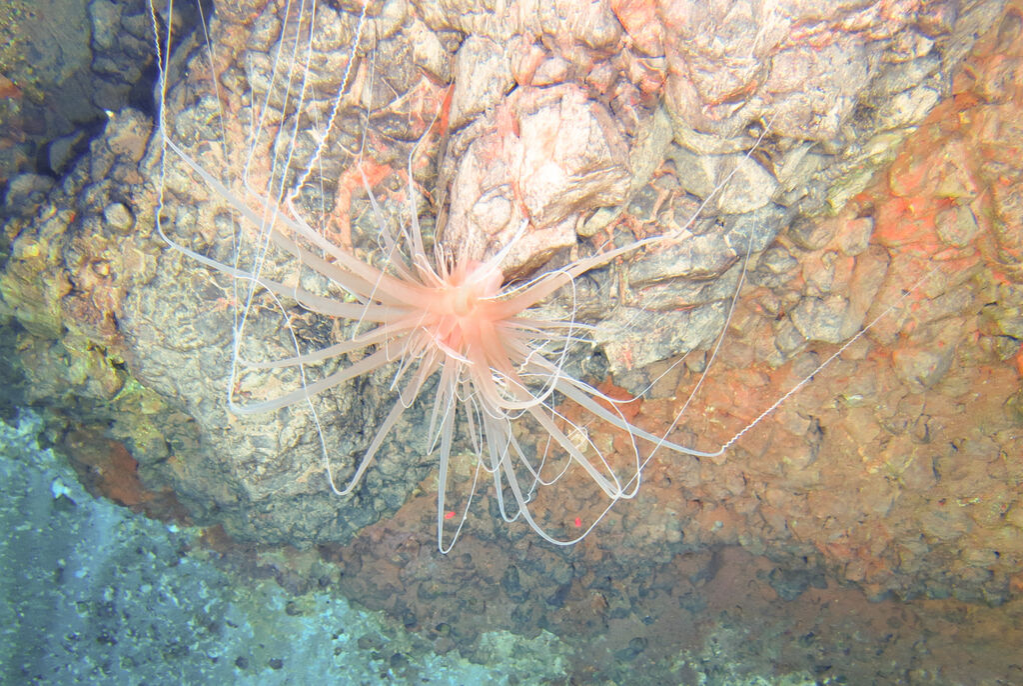
*Relicanthusdaphneae* sp. inc. in situ at the South East Indian Ridge within Vent site 5 in Cluster 11 of the INDEX area. Image corresponds with the data (Image attribution: BGR).

**Figure 92.**
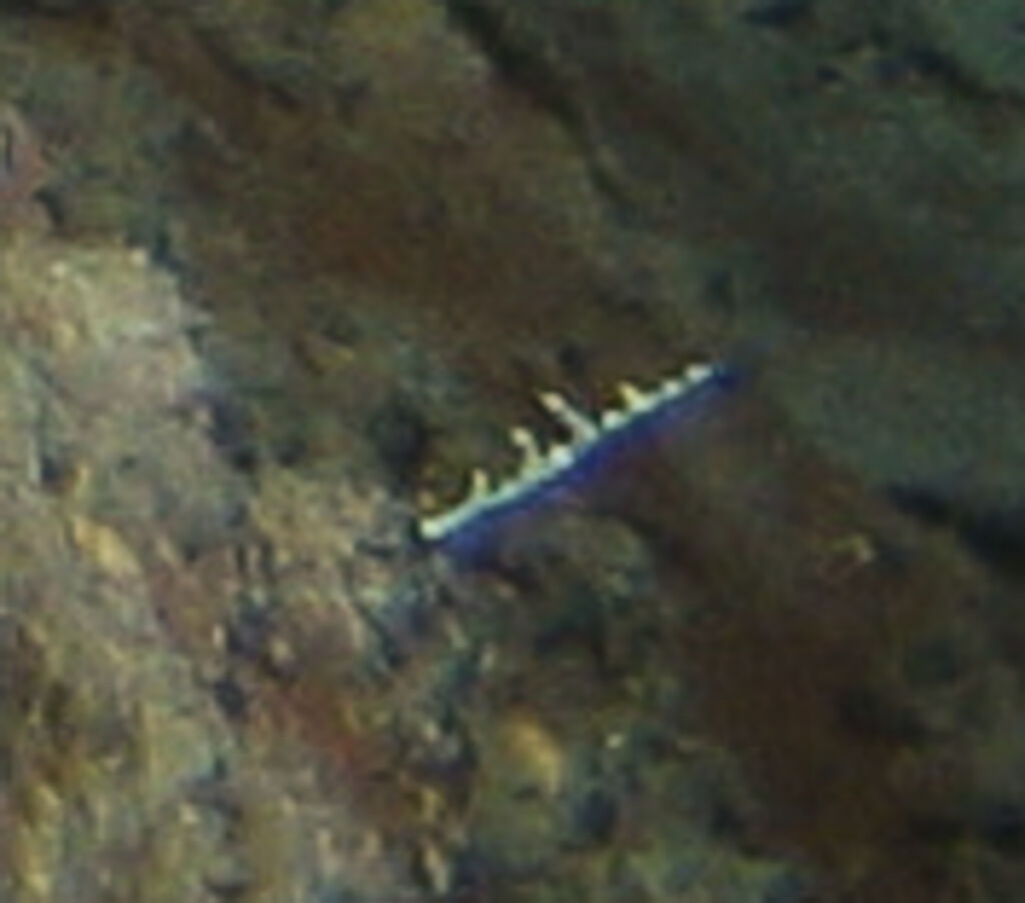
Alcyonacea fam. indet. in situ at the Central Indian Ridge within the MESO area outside the INDEX area. Image corresponds with the data (Image attribution: BGR).

**Figure 93.**
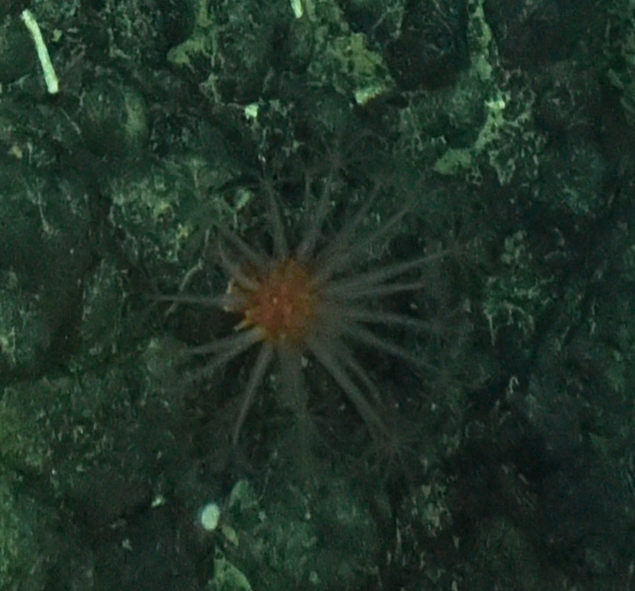
Alcyonacea *Anthomastus* gen. inc. in situ at the South East Indian Ridge within Vent site 6 in Cluster 12 of the INDEX area. Image corresponds with the data (Image attribution: BGR).

**Figure 94.**
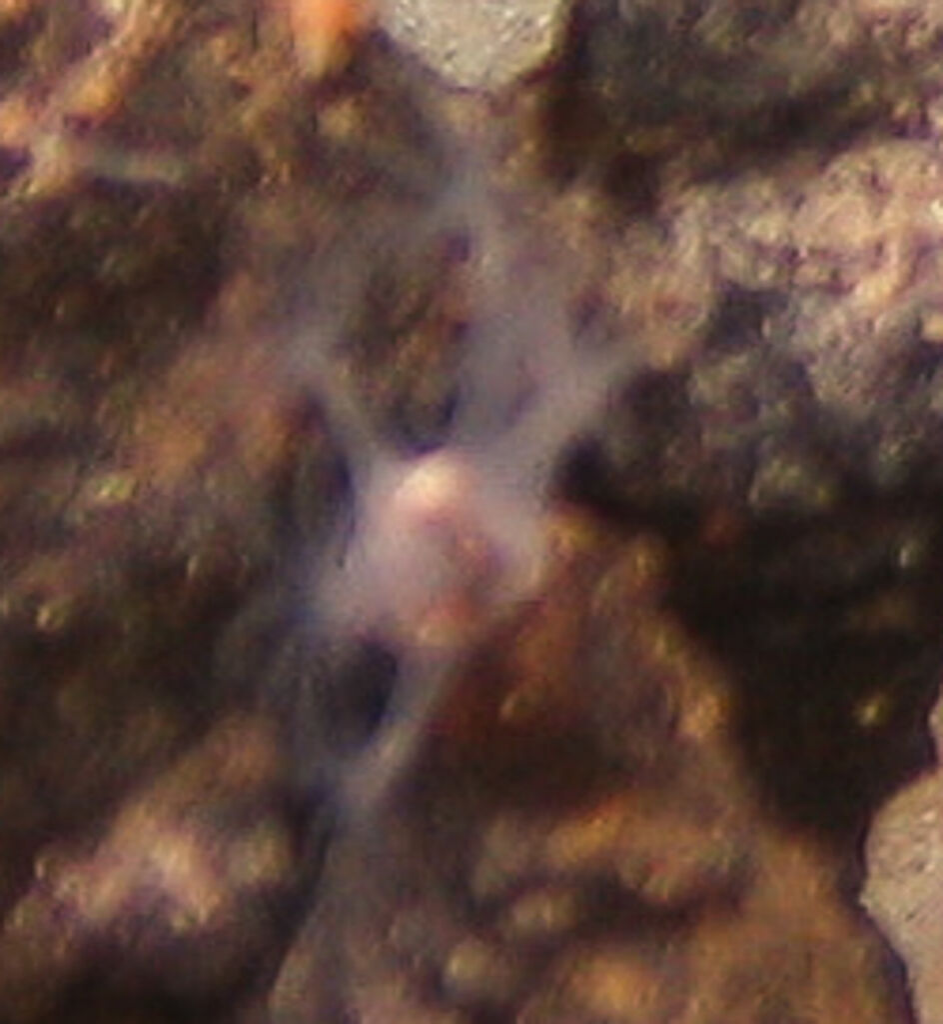
*Anthomastus* sp. indet. in situ at the Central Indian Ridge within the MESO area outside the INDEX area. Image corresponds with the data (Image attribution: BGR/ GEOMAR).

**Figure 96.**
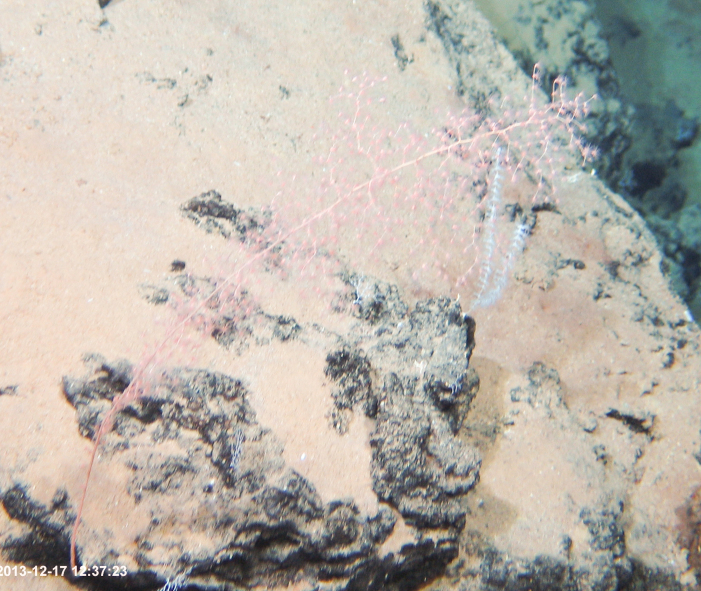
*Chrysogorgia* sp. indet. (DZMB_2021_0035) in situ at the Central Indian Ridge within the MESO area outside the INDEX area. Image corresponds with the data (Image attribution: BGR/ GEOMAR).

**Figure 97.**
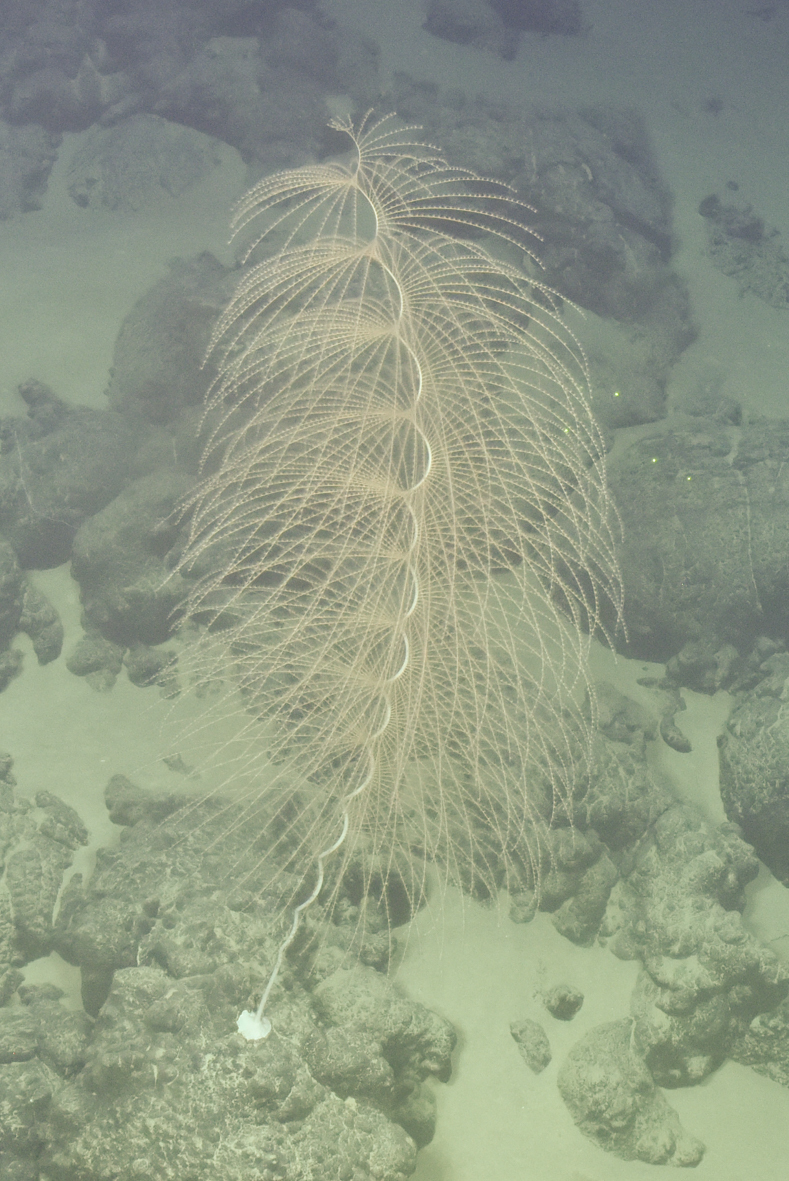
*Iridogorgiamagnispiralis* sp. inc. in situ at the South East Indian Ridge within Vent site 6 in Cluster 12 of the INDEX area. Image corresponds with the data (Image attribution: BGR).

**Figure 98.**
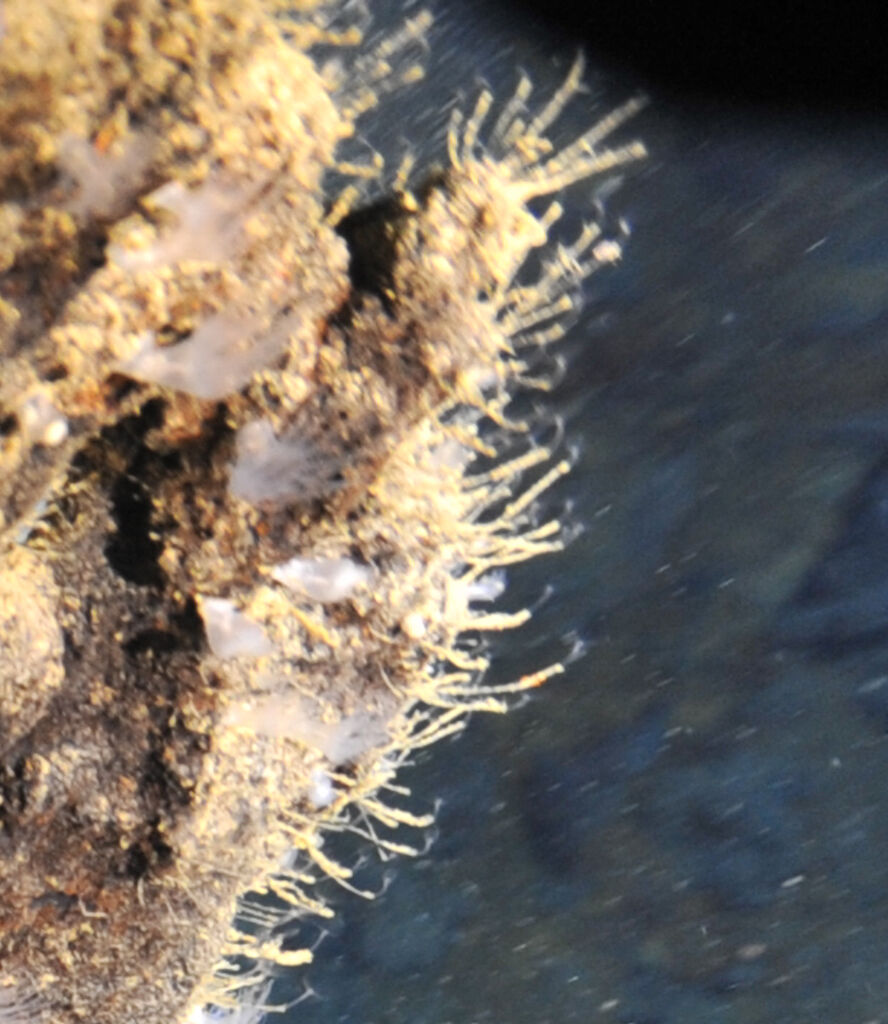
Clavulariidae gen. indet. (DZMB_2021_0036) in situ at the Central Indian Ridge within Vent site 1 in Cluster 4 of the INDEX area. Image corresponds with the data (Image attribution: BGR).

**Figure 99.**
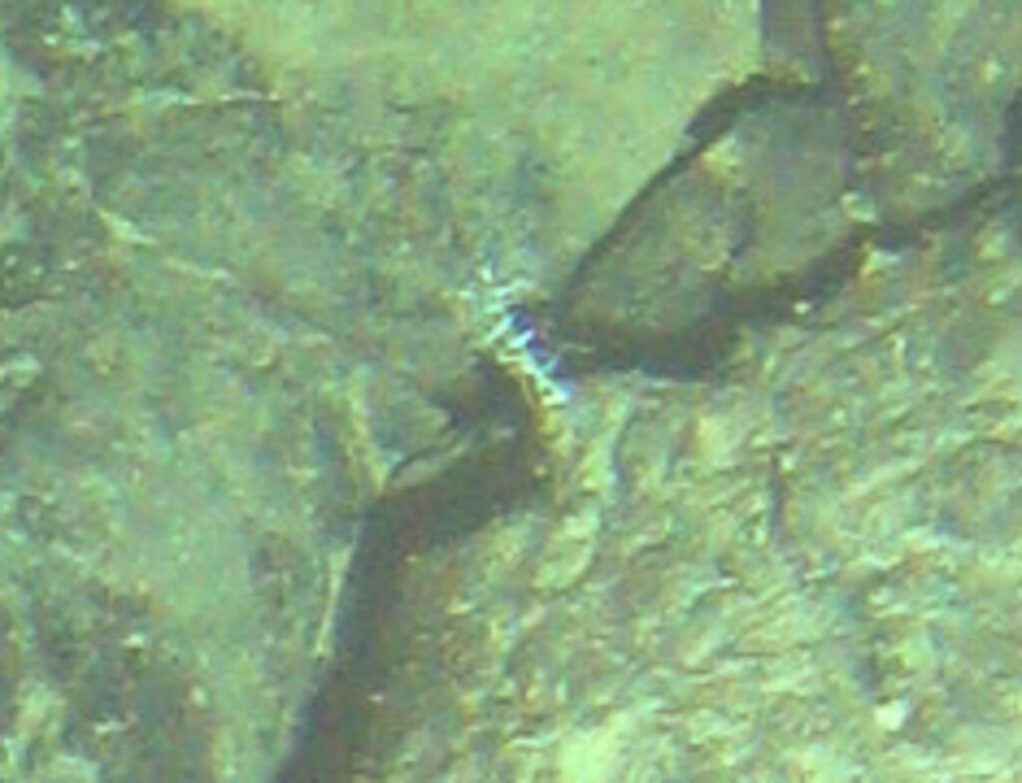
Clavulariidae gen. indet. (DZMB_2021_0037) in situ at the Central Indian Ridge within the MESO area outside the INDEX area. Image corresponds with the data (Image attribution: BGR).

**Figure 100.**
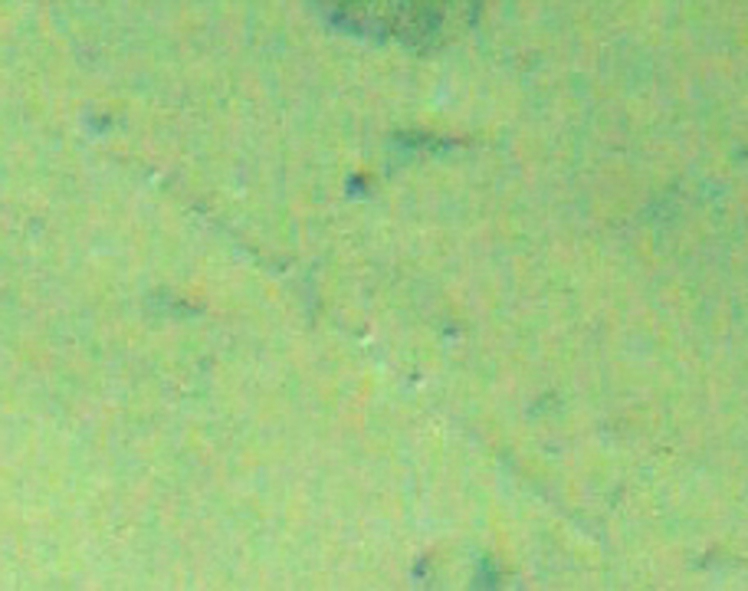
Clavulariidae fam. inc. (DZMB_2021_0038) in situ at the Central Indian Ridge within the MESO area outside the INDEX area. Image corresponds with the data (Image attribution: BGR).

**Figure 101.**
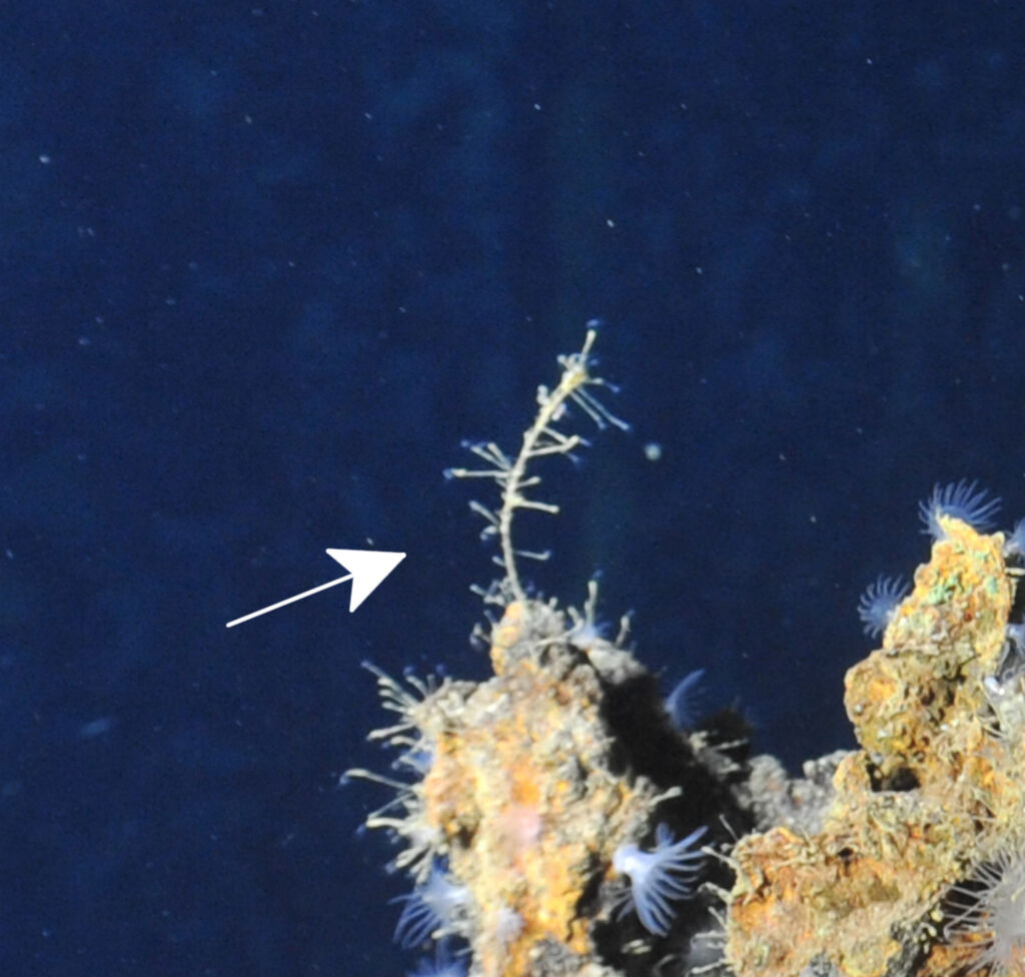
Clavulariidae fam. inc. (DZMB_2021_0039) in situ at the Central Indian Ridge within Vent site 1 in Cluster 4 of the INDEX area. Image corresponds with the data (Image attribution: BGR).

**Figure 102.**
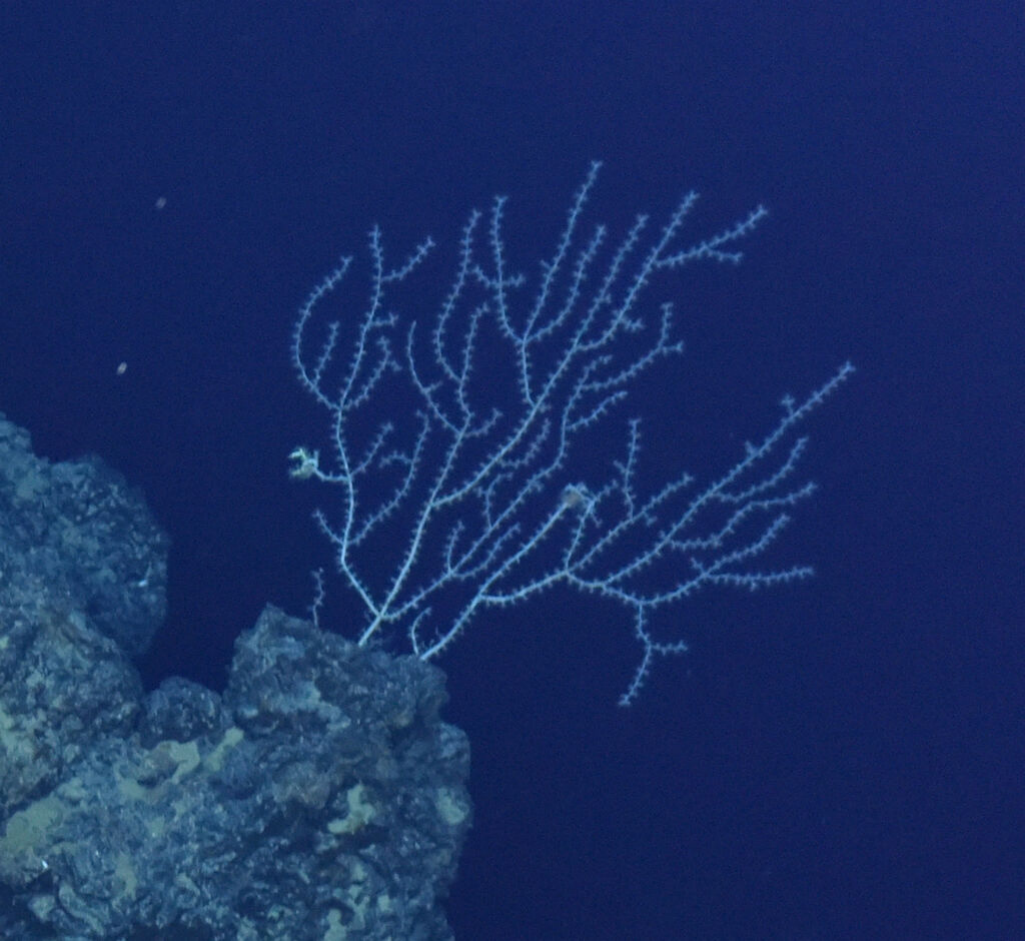
Isididae gen. indet. (DZMB_2021_0040) in situ at the South East Indian Ridge within Vent site 6 in Cluster 12 of the INDEX area. Image corresponds with the data (Image attribution: BGR).

**Figure 103.**
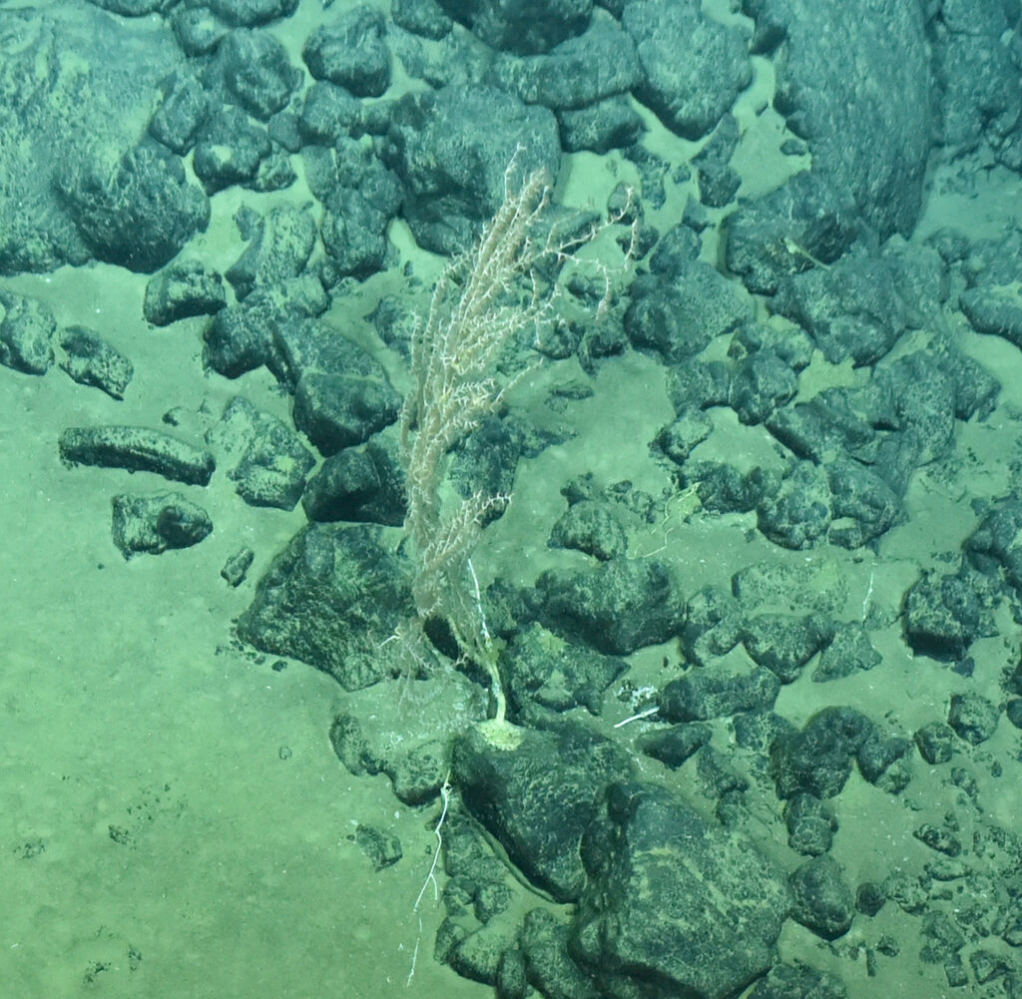
Isididae gen. indet. (DZMB_2021_0041) in situ at the South East Indian Ridge within Vent site 6 in Cluster 12 of the INDEX area. Image corresponds with the data (Image attribution: BGR).

**Figure 104.**
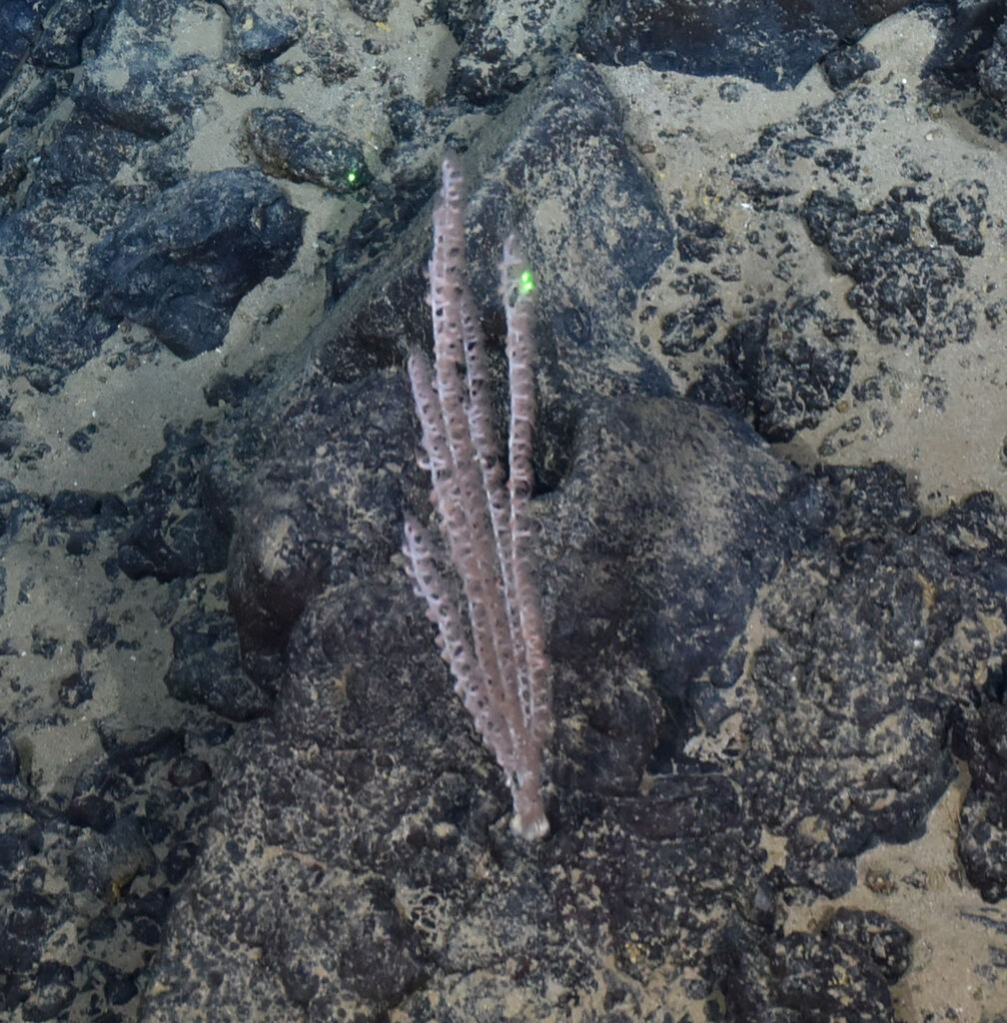
Isididae gen. indet. (DZMB_2021_0042) in situ at the South East Indian Ridge within Vent site 6 in Cluster 12 of the INDEX area. Image corresponds with the data (Image attribution: BGR).

**Figure 105.**
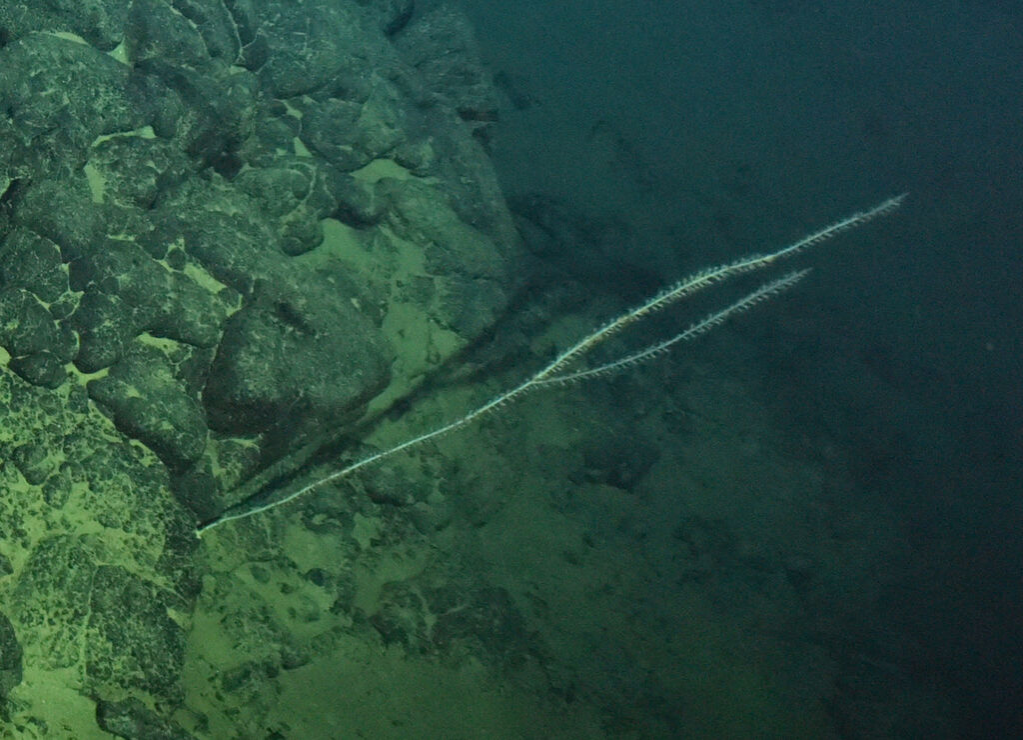
Isididae gen. indet. (DZMB_2021_0043) in situ at the South East Indian Ridge within Vent site 6 in Cluster 12 of the INDEX area. Image corresponds with the data (Image attribution: BGR).

**Figure 106.**
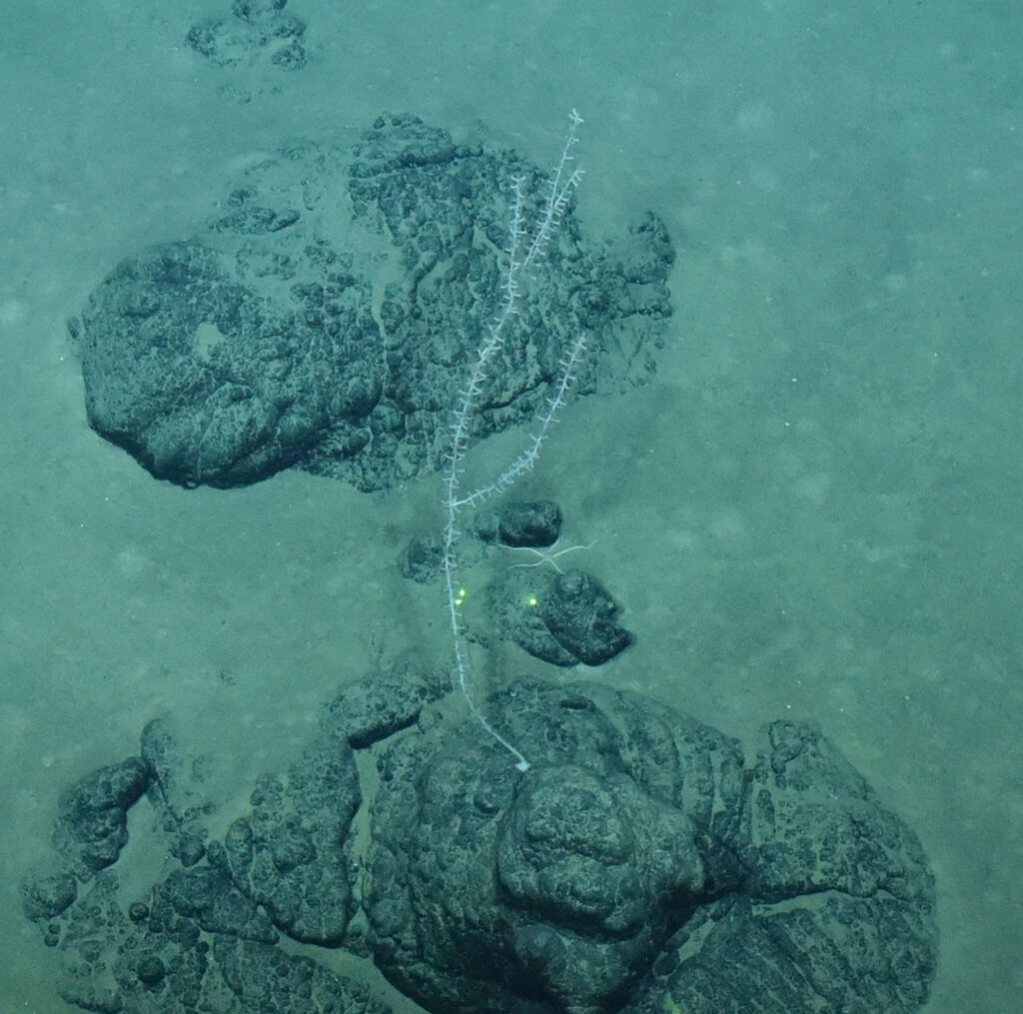
Isididae fam. inc. (DZMB_2021_0044) in situ at the South East Indian Ridge within Vent site 6 in Cluster 12 of the INDEX area. Image corresponds with the data (Image attribution: BGR).

**Figure 107.**
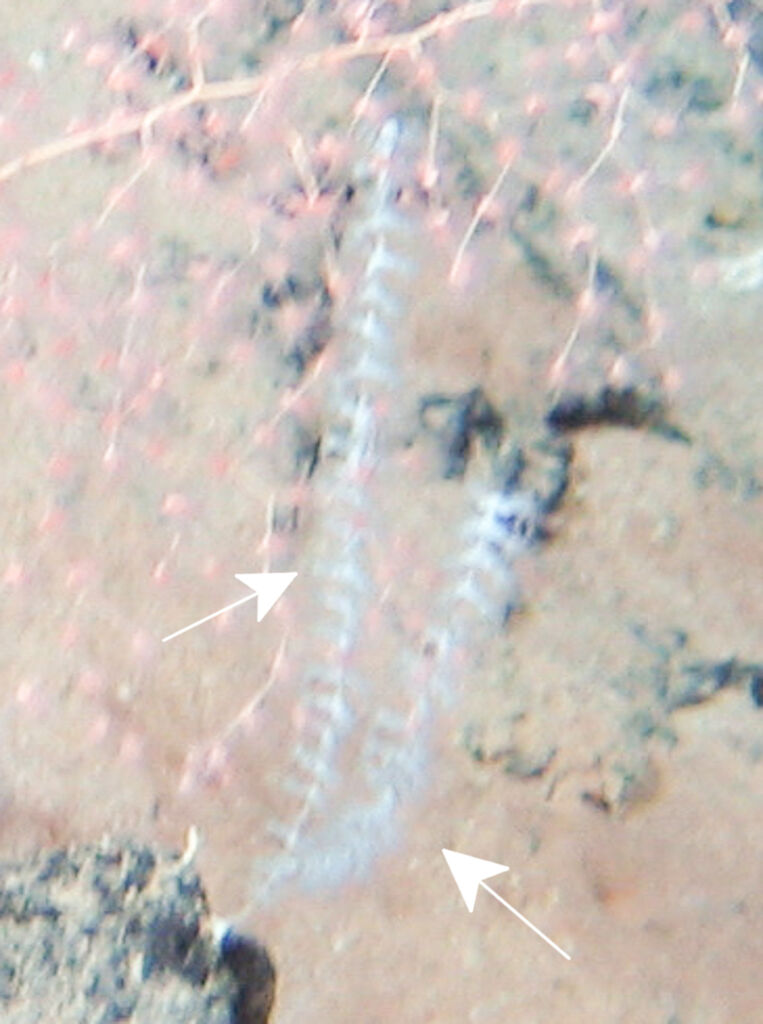
Isididae gen. indet. (DZMB_2021_0045) in situ at the Central Indian Ridge within the MESO area outside the INDEX area. Image corresponds with the data (Image attribution: BGR).

**Figure 108.**
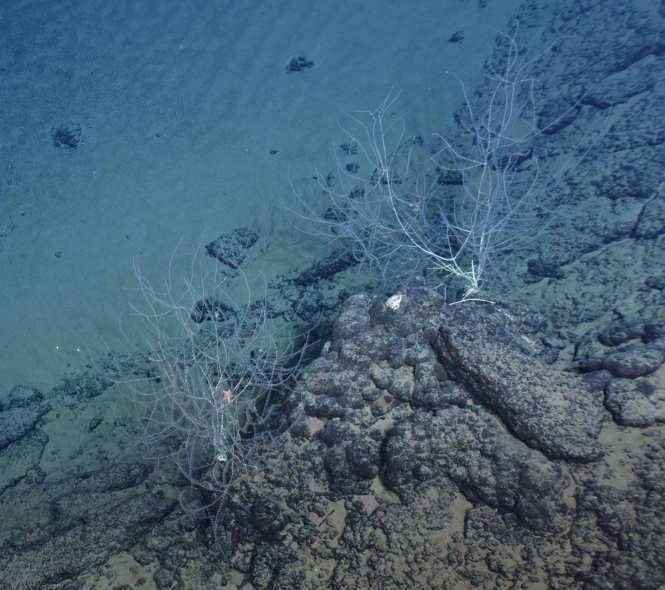
Isididae *Acanella* gen. inc. in situ at the South East Indian Ridge within Vent site 6 in Cluster 12 of the INDEX area. Image corresponds with the data (Image attribution: BGR).

**Figure 109.**
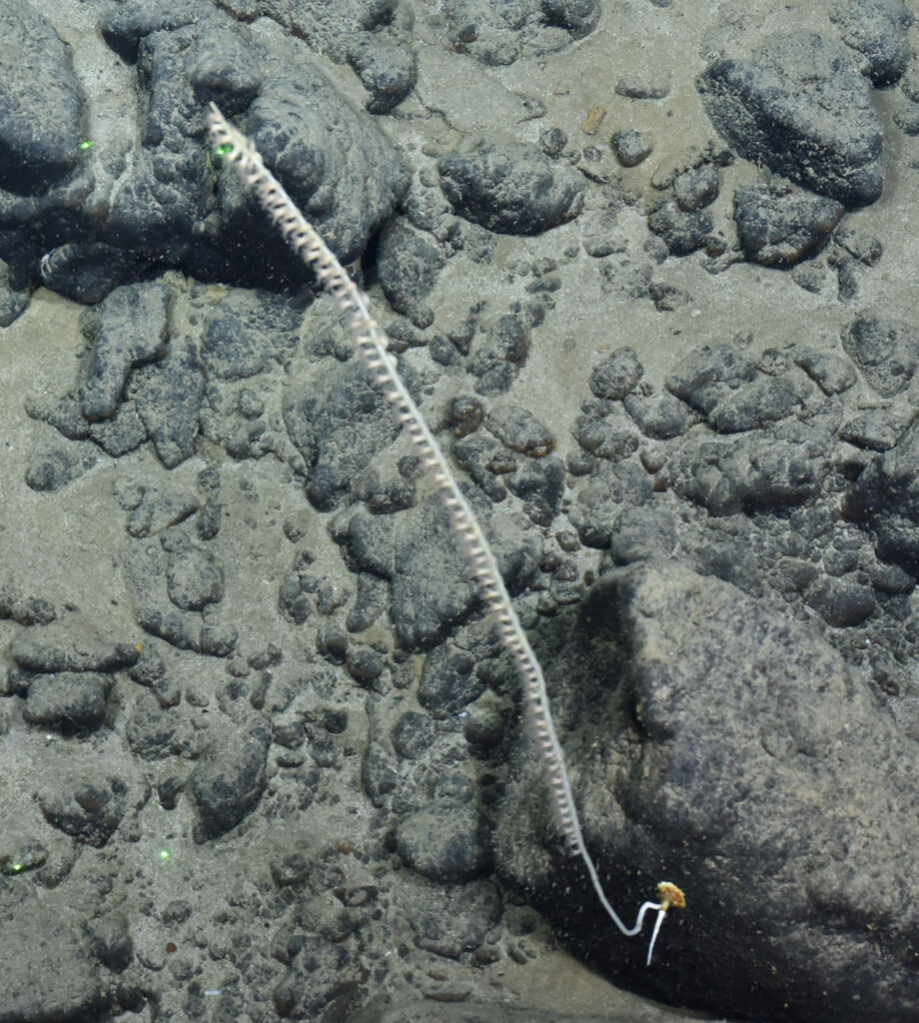
Isididae *Bathygorgia* gen. inc. in situ at the Rodriguez Triple Junction within Vent site 4 in Cluster 5 of the INDEX area. Image corresponds with the data (Image attribution: BGR).

**Figure 110.**
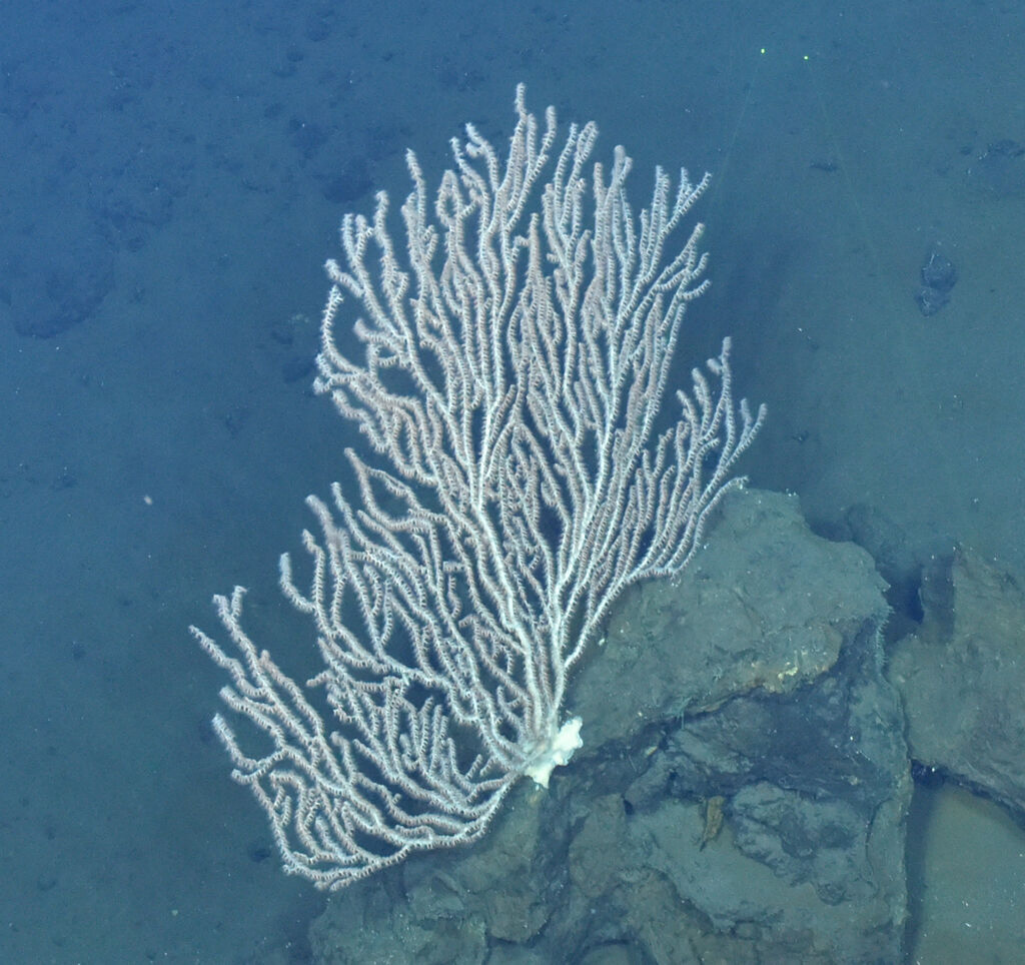
Isididae *Jasonisis* gen. inc. in situ at the South East Indian Ridge within Vent site 6 in Cluster 12 of the INDEX area. Image corresponds with the data (Image attribution: BGR).

**Figure 111.**
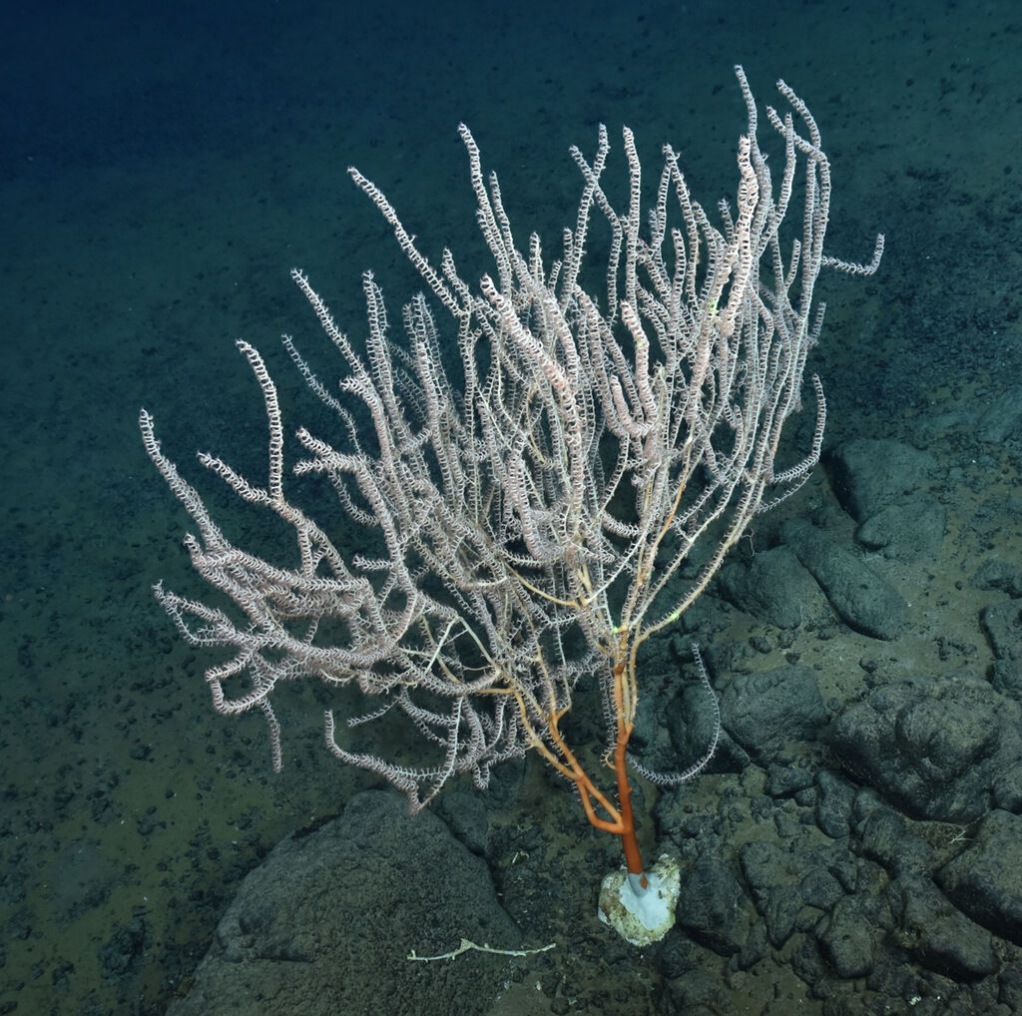
Isididae *Keratoisis* gen. inc. (DZMB_2021_0046) in situ at the Rodriguez Triple Junction within Vent site 4 in Cluster 5 of the INDEX area. Image corresponds with the data (Image attribution: BGR).

**Figure 112.**
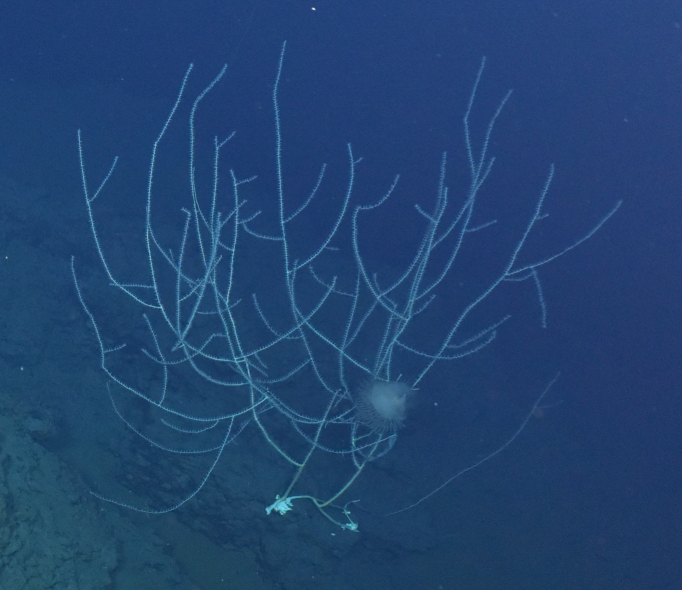
Isididae *Keratoisis* gen. inc. (DZMB_2021_0047) in situ at the Rodriguez Triple Junction within Vent site 4 in Cluster 5 of the INDEX area. Image corresponds with the data (Image attribution: BGR).

**Figure 113.**
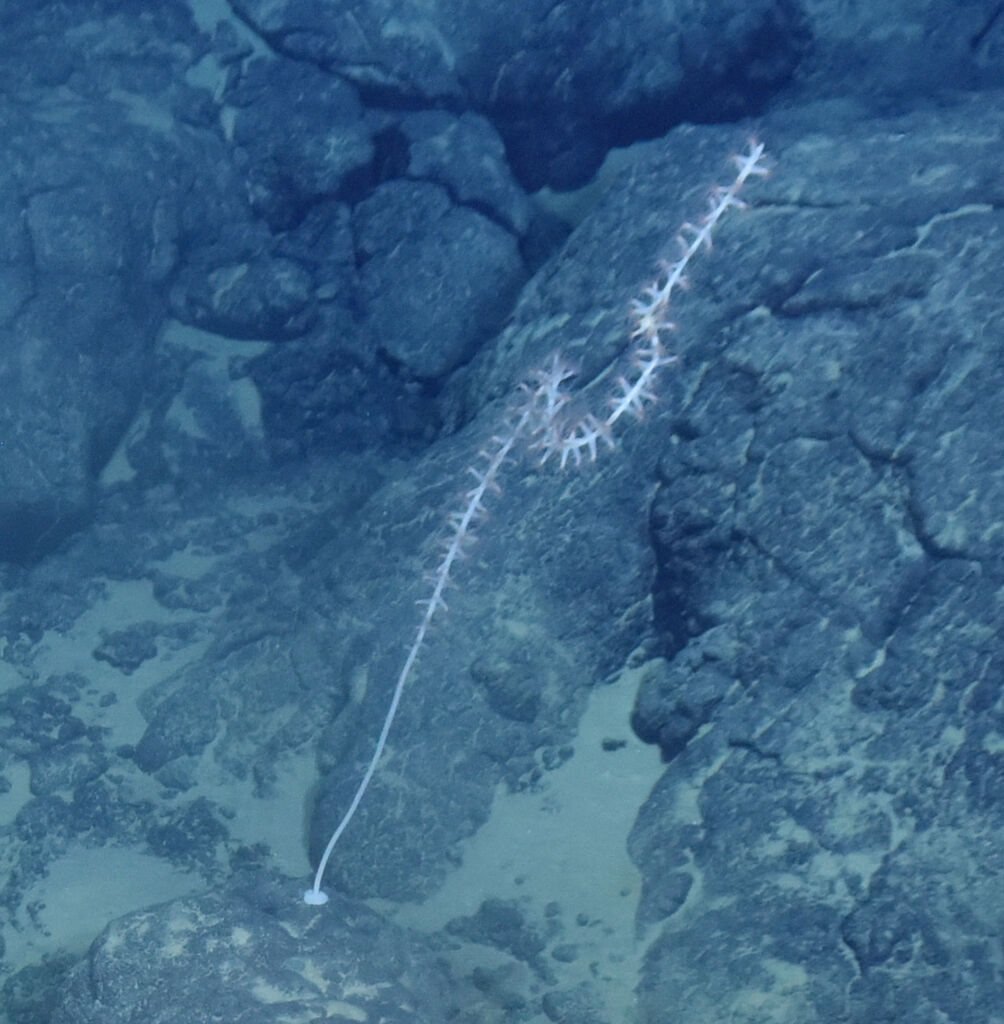
Isididae *Lepidisis* gen. inc. in situ at the South East Indian Ridge at the border of Vent site 3 in Cluster 12 of the INDEX area. Image corresponds with the data (Image attribution: BGR).

**Figure 114.**
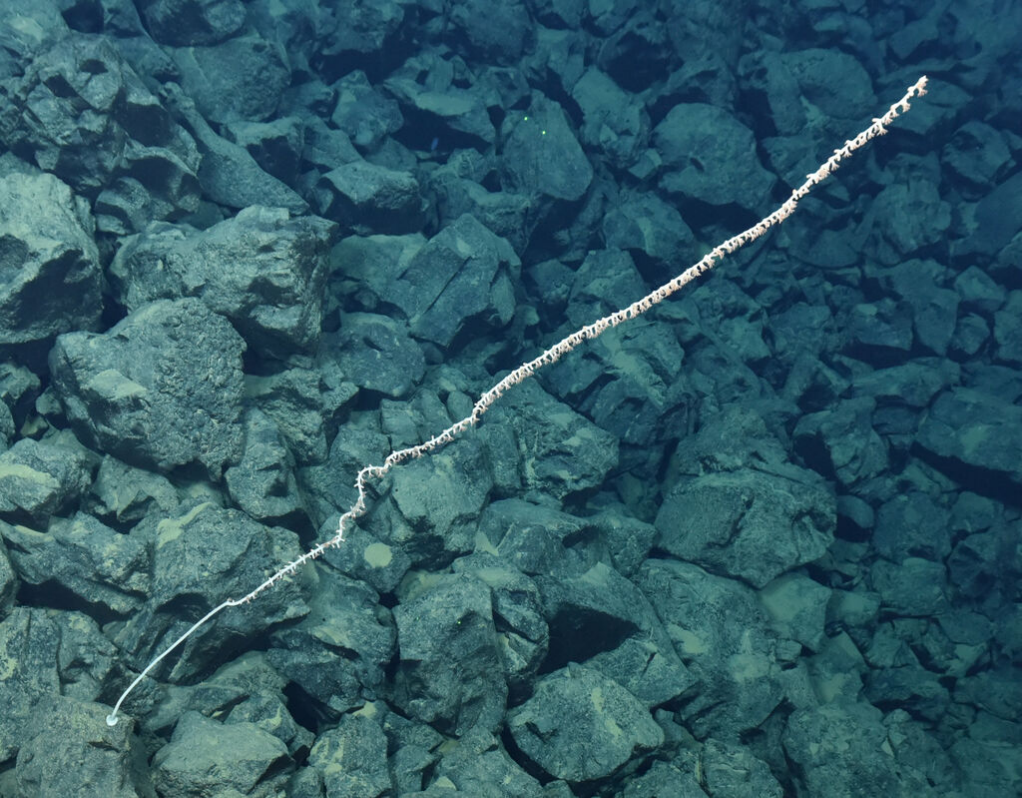
*Lepidisis* sp. indet. in situ at the South East Indian Ridge within Vent site 5 in Cluster 11 of the INDEX area. The individual is an example for the species complex *Lepidisis* spp. indet., with more images and entries in the supplementary imagery and data table. Image corresponds with the data (Image attribution: BGR).

**Figure 115.**
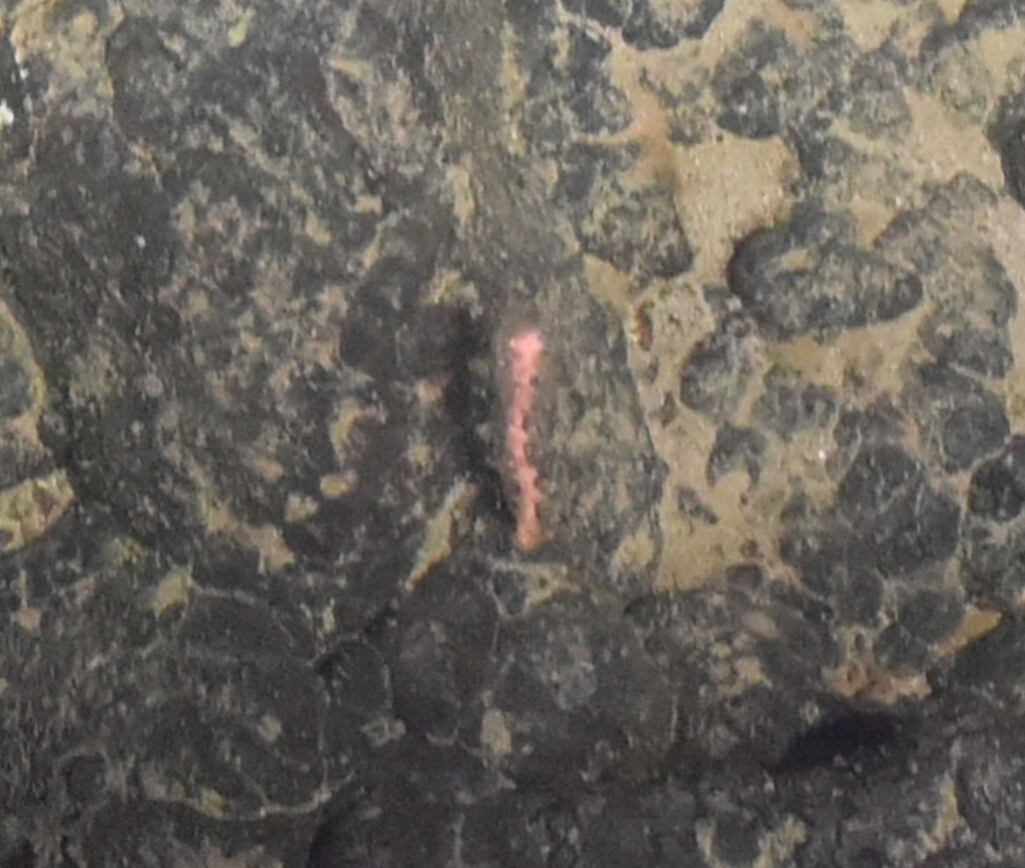
Paragorgiidae fam. inc. in situ at the South East Indian Ridge within Vent site 6 in Cluster 12 of the INDEX area. Image corresponds with the data (Image attribution: BGR).

**Figure 116.**
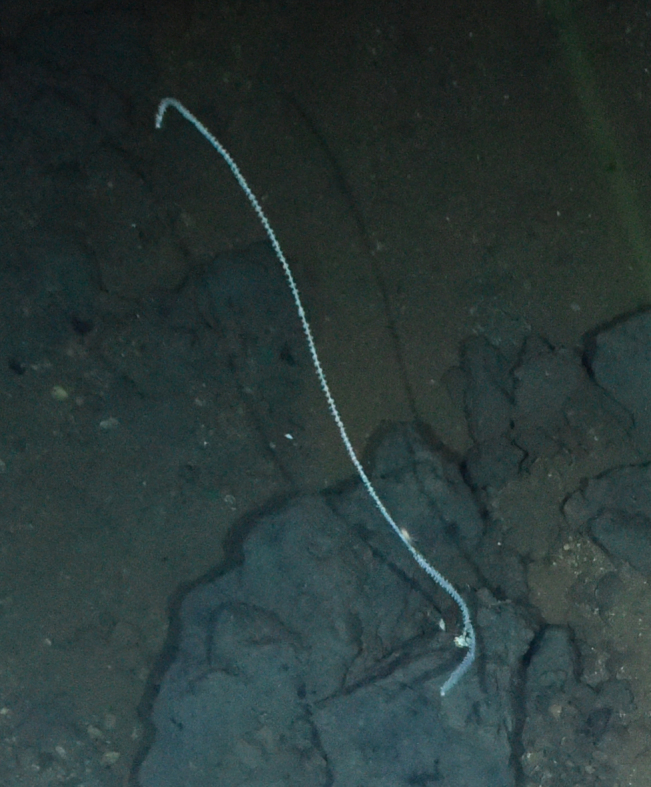
Primnoidae gen. indet. (DZMB_2021_0048) in situ at the Rodriguez Triple Junction within Vent site 4 in Cluster 5 of the INDEX area. Image corresponds with the data (Image attribution: BGR).

**Figure 117.**
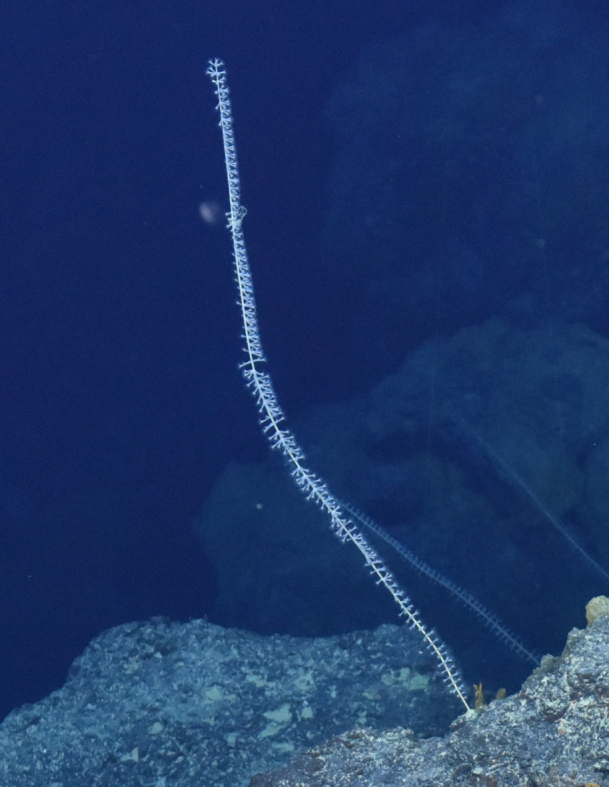
Primnoidae gen. indet. (DZMB_2021_0049) in situ at the South East Indian Ridge at the border of Vent site 6 in Cluster 12 of the INDEX area. Image corresponds with the data (Image attribution: BGR).

**Figure 118.**
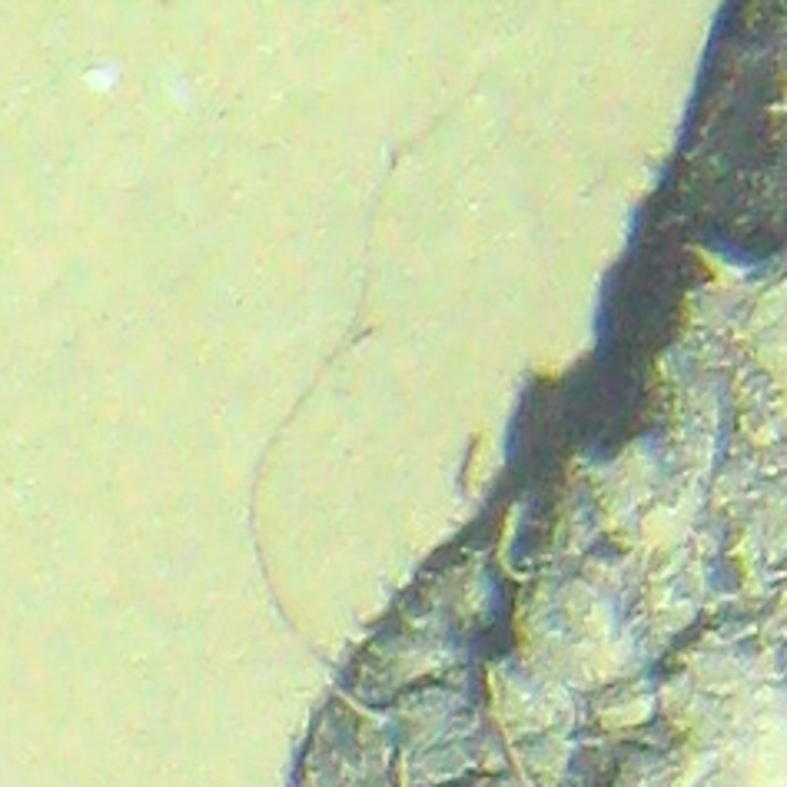
Stalk of Alcyonacea or Antipatharia ord. inc. in situ at the Central Indian Ridge within the MESO area outside the INDEX area. Image corresponds with the data (Image attribution: BGR).

**Figure 119.**
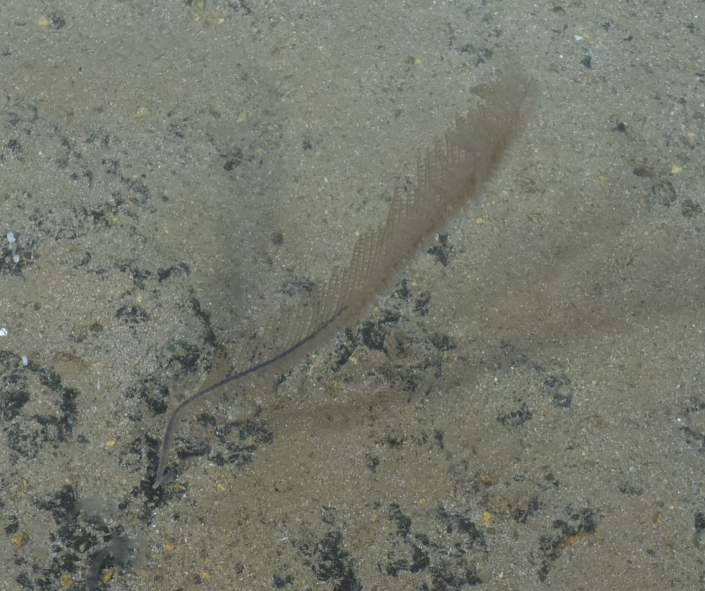
*Heteropathes* sp. indet. in situ at the South East Indian Ridge at the border of Vent site 6 in Cluster 12 of the INDEX area. Image corresponds with the data (Image attribution: BGR).

**Figure 120.**
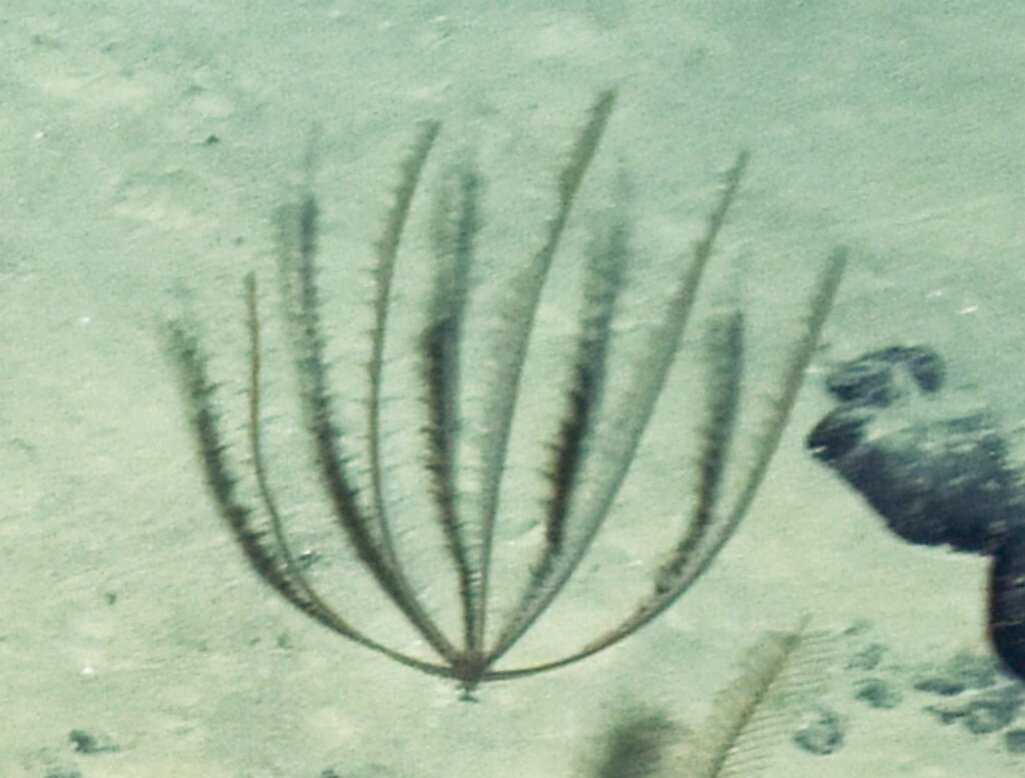
*Heteropathesamericana* sp. inc. in situ at the South East Indian Ridge at the border of Vent site 6 in Cluster 12 of the INDEX area. Image corresponds with the data (Image attribution: BGR).

**Figure 121.**
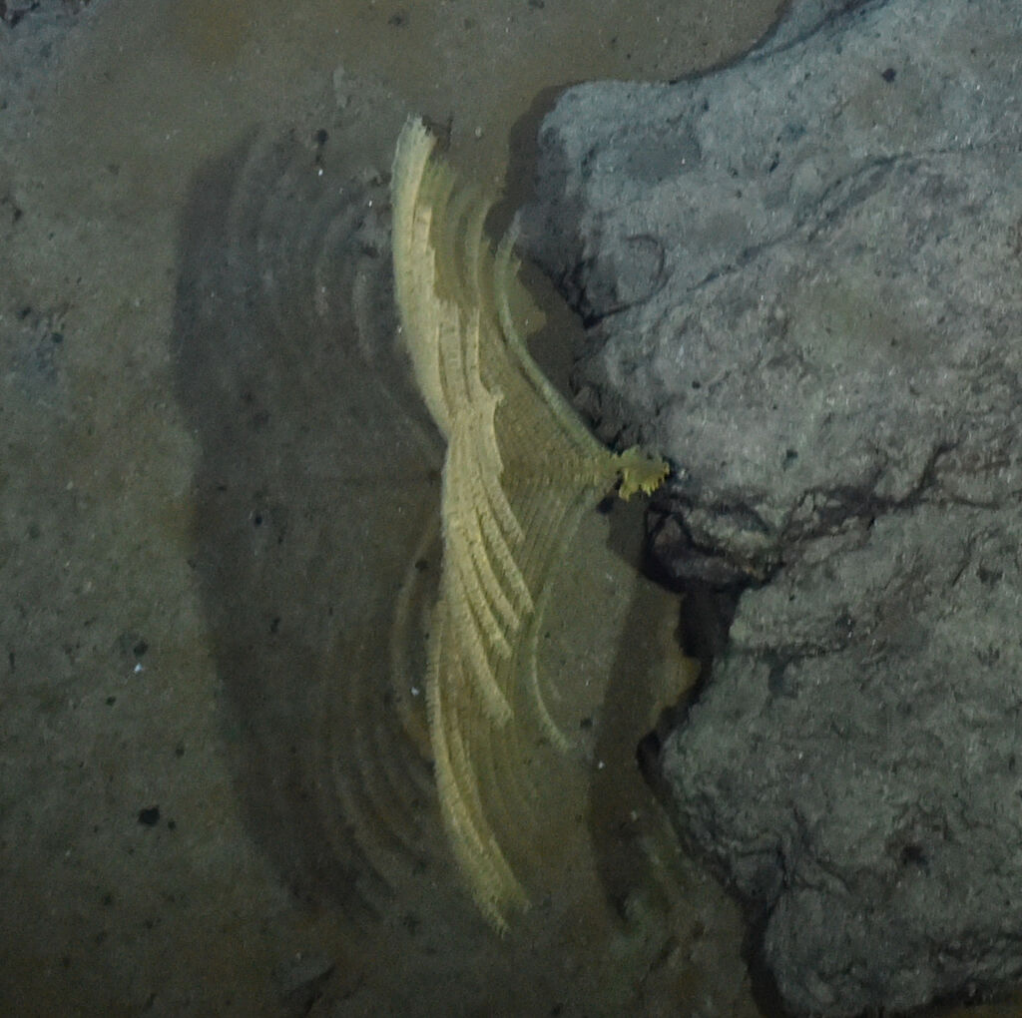
*Bathypathes* sp. indet. (DZMB_2021_0050) in situ at the Rodriguez Triple Junction at the border of Vent site 4 in Cluster 5 of the INDEX area. Image corresponds with the data (Image attribution: BGR).

**Figure 122.**
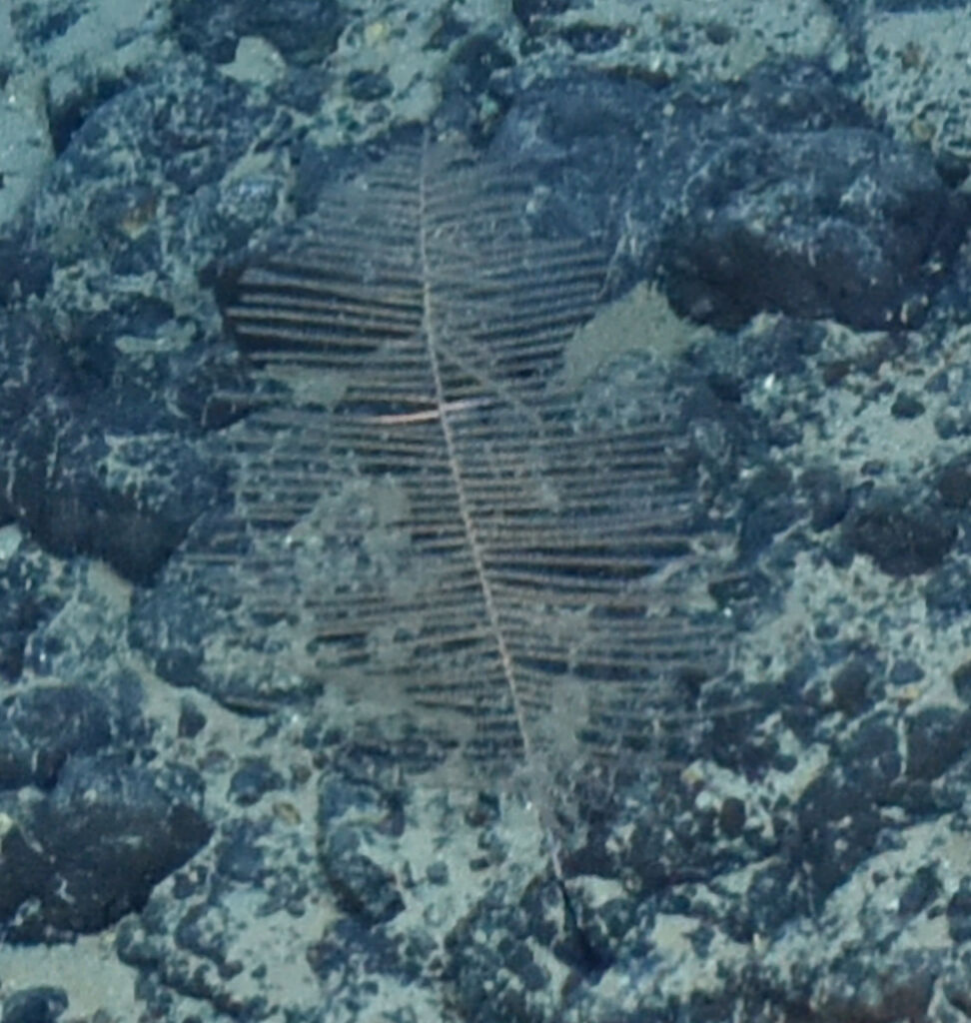
*Bathypathes* gen. inc. (DZMB_2021_0051) in situ at the South East Indian Ridge at the border of Vent site 6 in Cluster 12 of the INDEX area. Image corresponds with the data (Image attribution: BGR).

**Figure 123.**
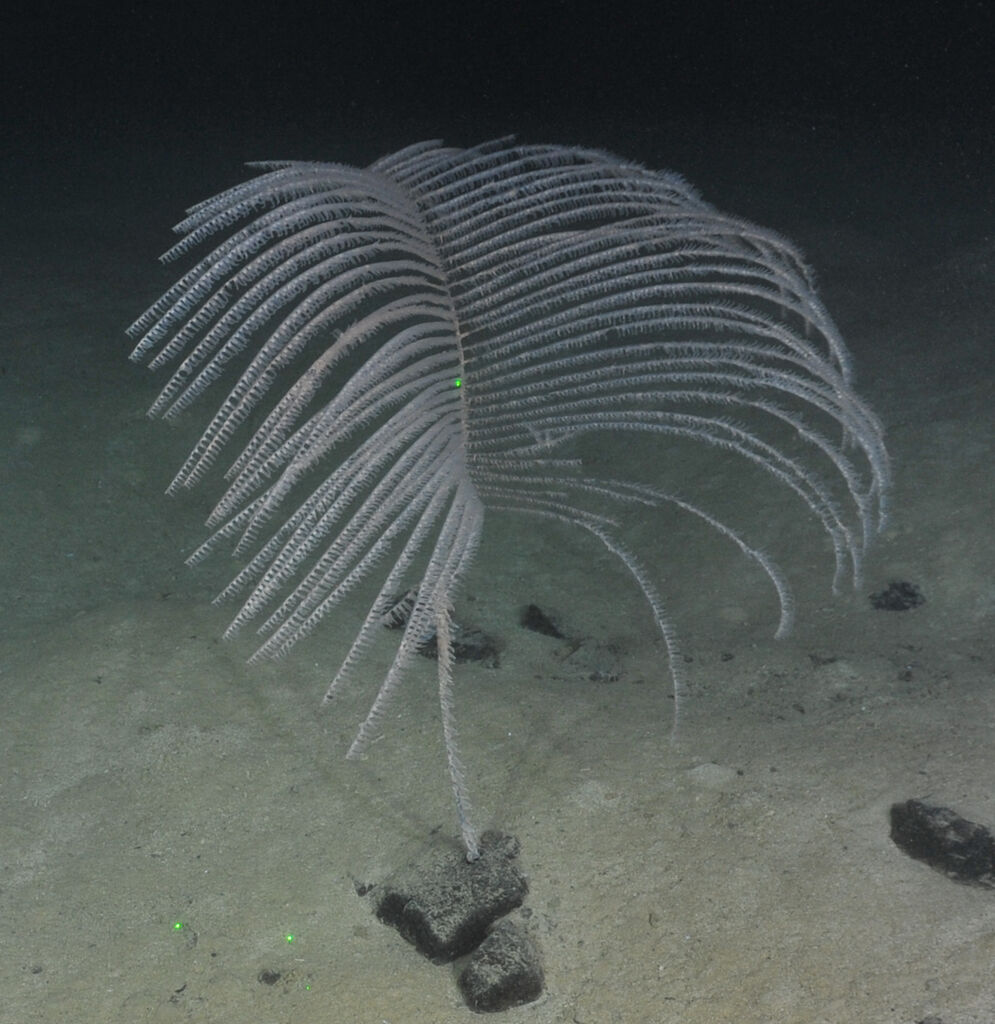
*Bathypathespatula* sp. inc. in situ at the Central Indian Ridge at the border of Vent site 1 in Cluster 4 of the INDEX area. Image corresponds with the data (Image attribution: BGR).

**Figure 124.**
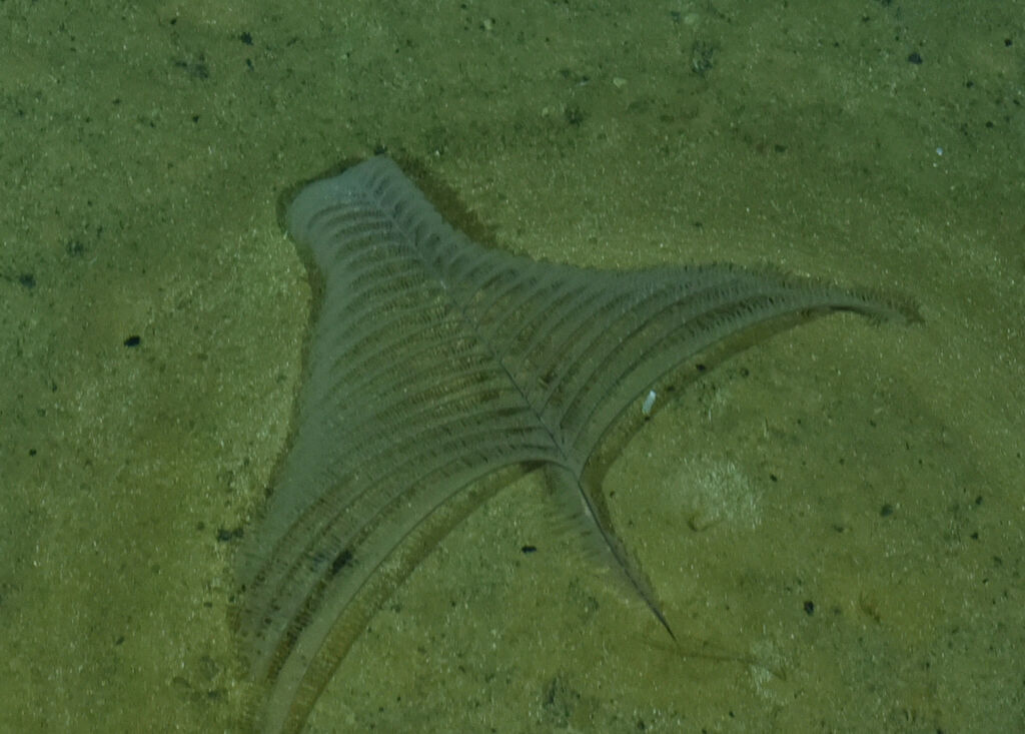
*Schizopathes* sp. indet. in situ at the Rodriguez Triple Junction at the border of Vent site 4 in Cluster 5 of the INDEX area. The individual is an example for the species complex *Schizopathes* spp. indet., with more images and entries in the supplementary imagery and data table. Image corresponds with the data (Image attribution: BGR).

**Figure 125.**
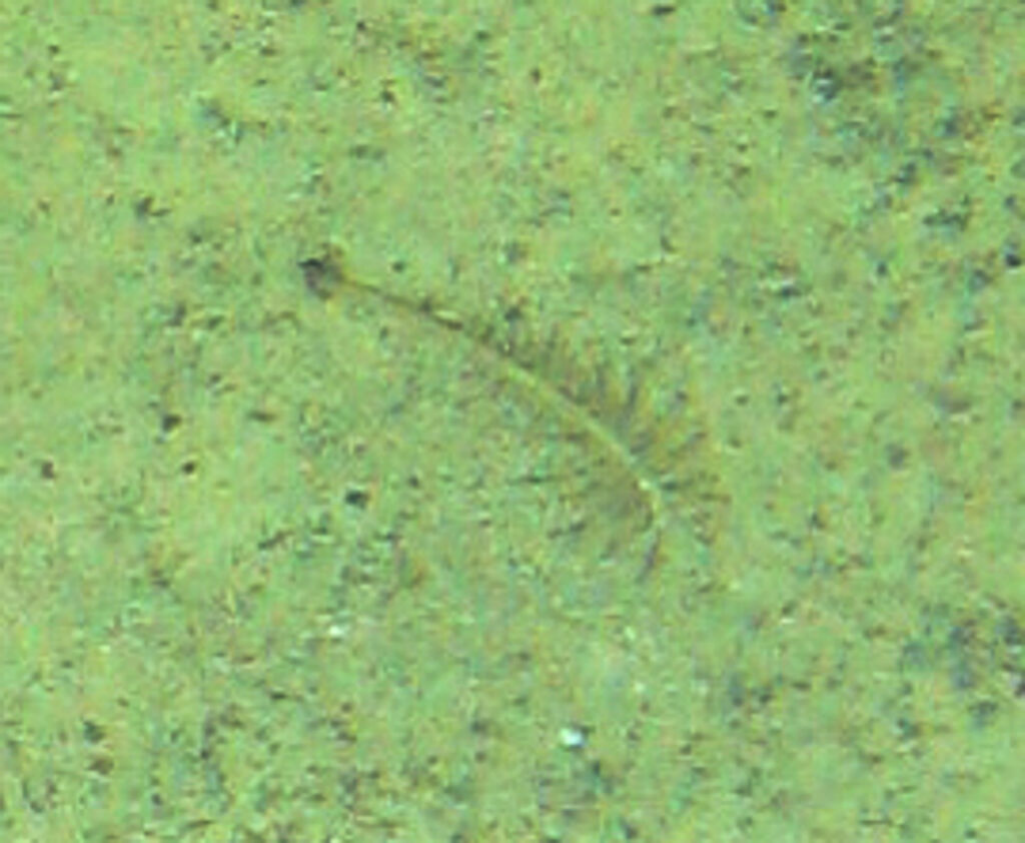
Pennatulacea ord. inc. (DZMB_2021_0052) in situ at the Central Indian Ridge within the MESO area outside the INDEX area. Image corresponds with the data (Image attribution: BGR).

**Figure 126.**
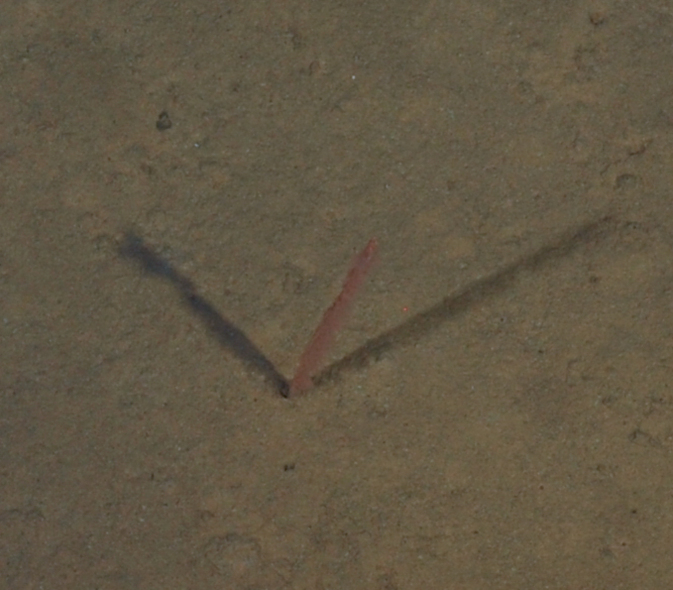
Pennatulacea fam. indet. (DZMB_2021_0053) in situ at the Central Indian Ridge within the Edmond-Vent site 2-vent site 7 area in Cluster 4 of the INDEX area. Image corresponds with the data (Image attribution: BGR).

**Figure 127.**
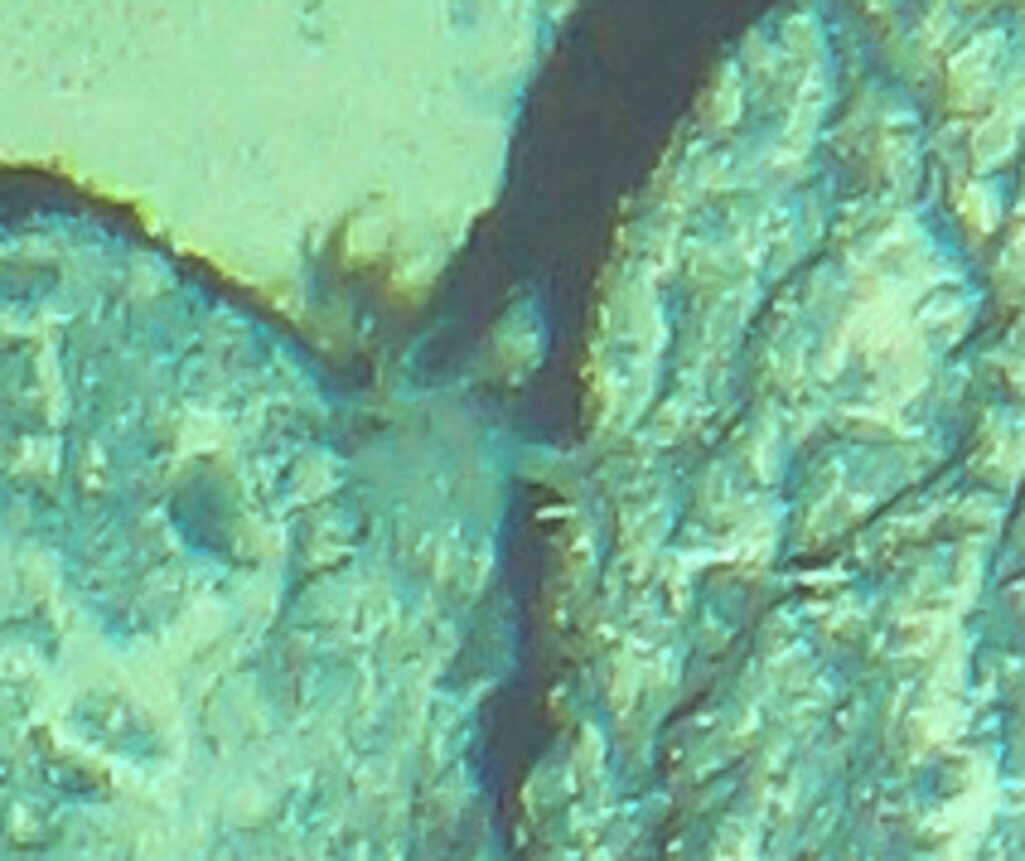
Pennatulacea *Kophobelemnon* ord. inc. incertae sedis in situ at the Central Indian Ridge within the MESO area outside the INDEX area. Image corresponds with the data (Image attribution: BGR).

**Figure 128.**
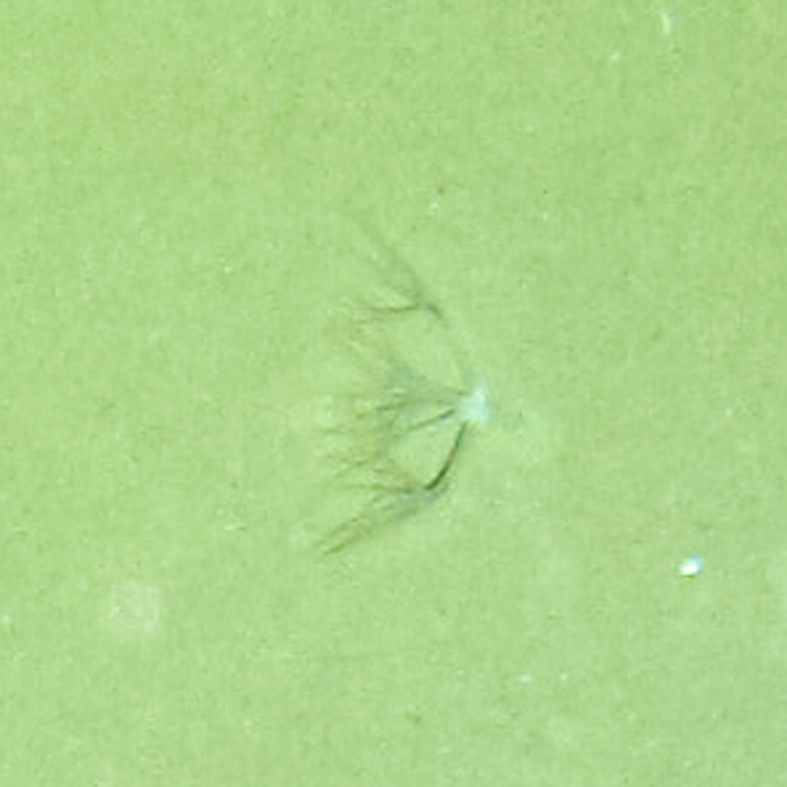
*Umbellula* sp. indet. (DZMB_2021_0054) in situ at the Central Indian Ridge at the border of the Edmond hydrothermal vent field in Cluster 4 of the INDEX area. Image corresponds with the data (Image attribution: BGR).

**Figure 129.**
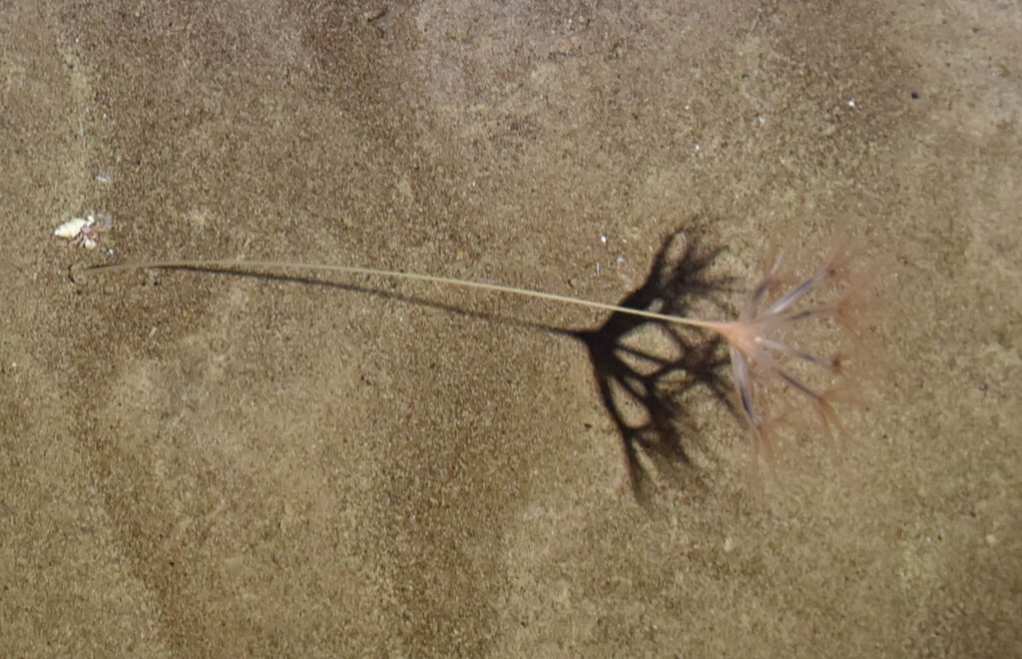
*Umbellula* sp. indet. (DZMB_2021_0055) in situ at the Rodriguez Triple Junction at the border of Vent site 4 in Cluster 5 of the INDEX area. Image corresponds with the data (Image attribution: BGR).

**Figure 130.**
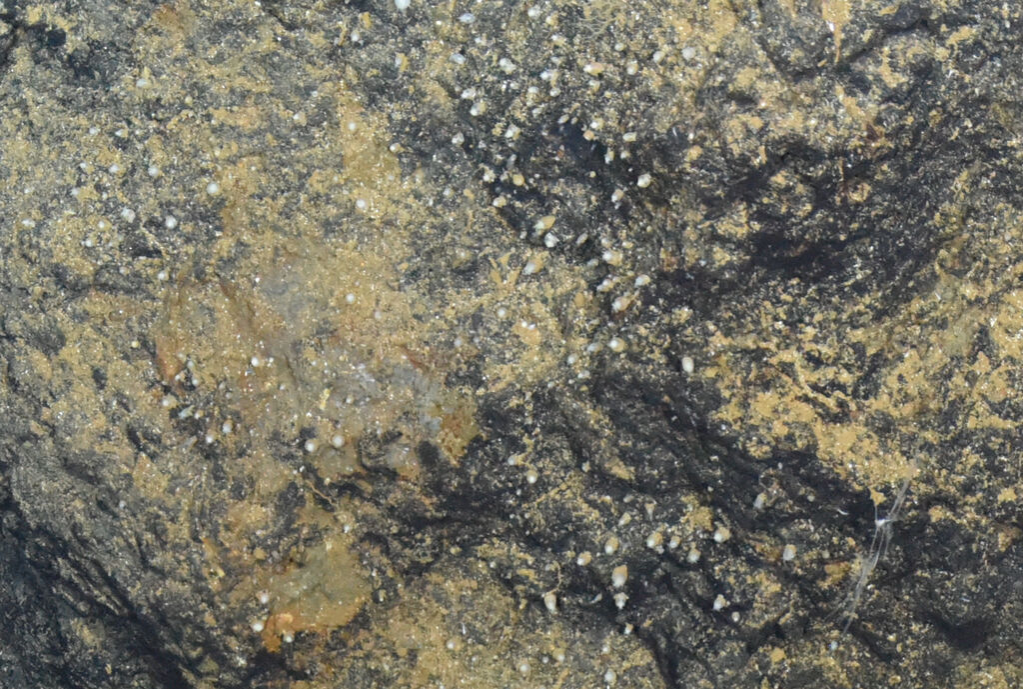
Zoantharia fam. indet. (DZMB_2021_0056) in situ at the South East Indian Ridge within Vent site 3 in Cluster 12 of the INDEX area. Image corresponds with the data (Image attribution: BGR).

**Figure 131.**
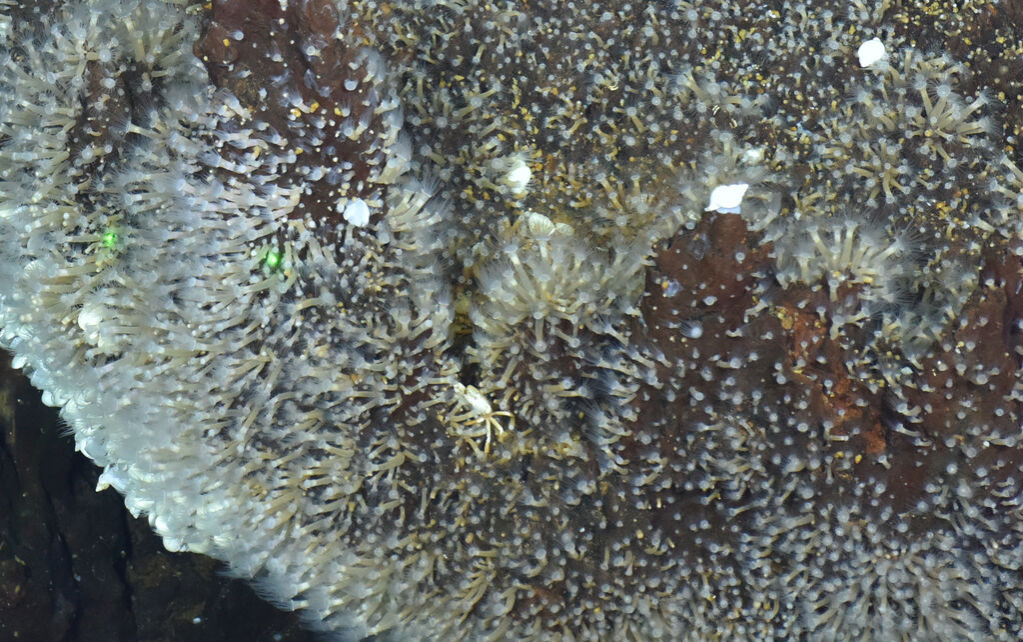
Zoantharia fam. indet. (DZMB_2021_0057) in situ at the South East Indian Ridge within Vent site 6 in Cluster 12 of the INDEX area. Image corresponds with the data (Image attribution: BGR).

**Figure 132.**
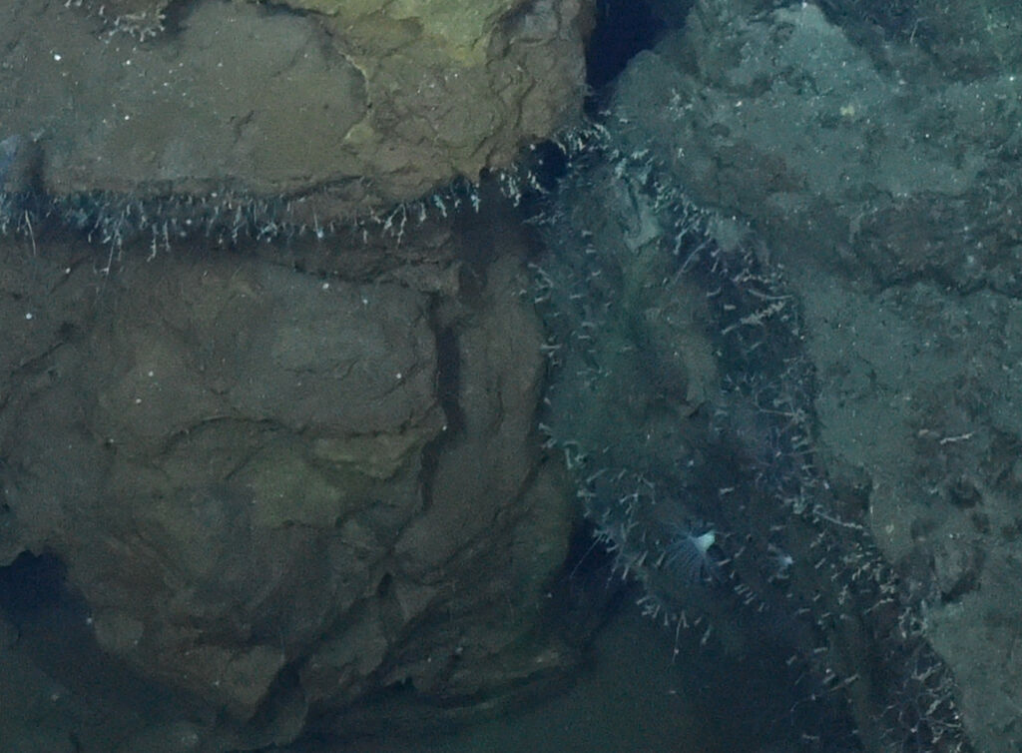
Zoantharia fam. indet. (DZMB_2021_0058) in situ at the South East Indian Ridge within Vent site 6 in Cluster 12 of the INDEX area. Image corresponds with the data (Image attribution: BGR).

**Figure 133.**
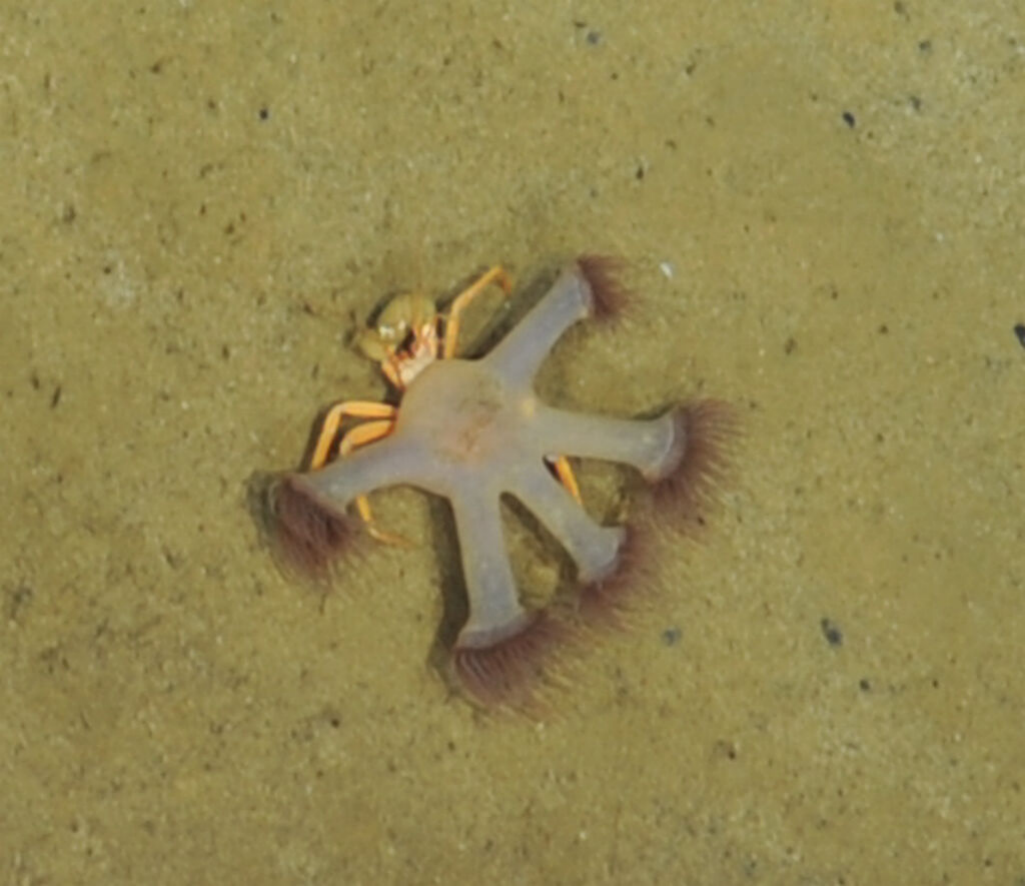
*Epizoanthus* sp. indet. (in symbiosis with Paguroidea superfam. inc.) in situ at the Central Indian Ridge in the surrounding of Vent site 1 in Cluster 4 of the INDEX area. Image corresponds with the data (Image attribution: BGR).

**Figure 134.**
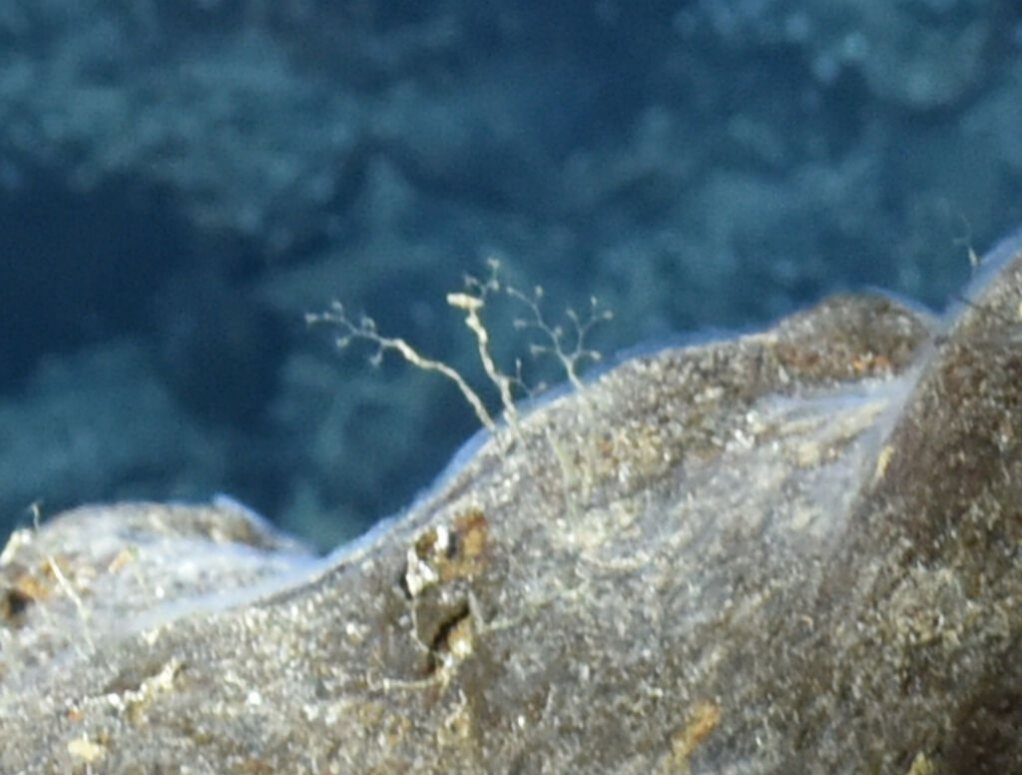
Hydrozoa ord. indet. (DZMB_2021_0059) in situ at the South East Indian Ridge within Vent site 6 in Cluster 12 of the INDEX area. Image corresponds with the data (Image attribution: BGR).

**Figure 135.**
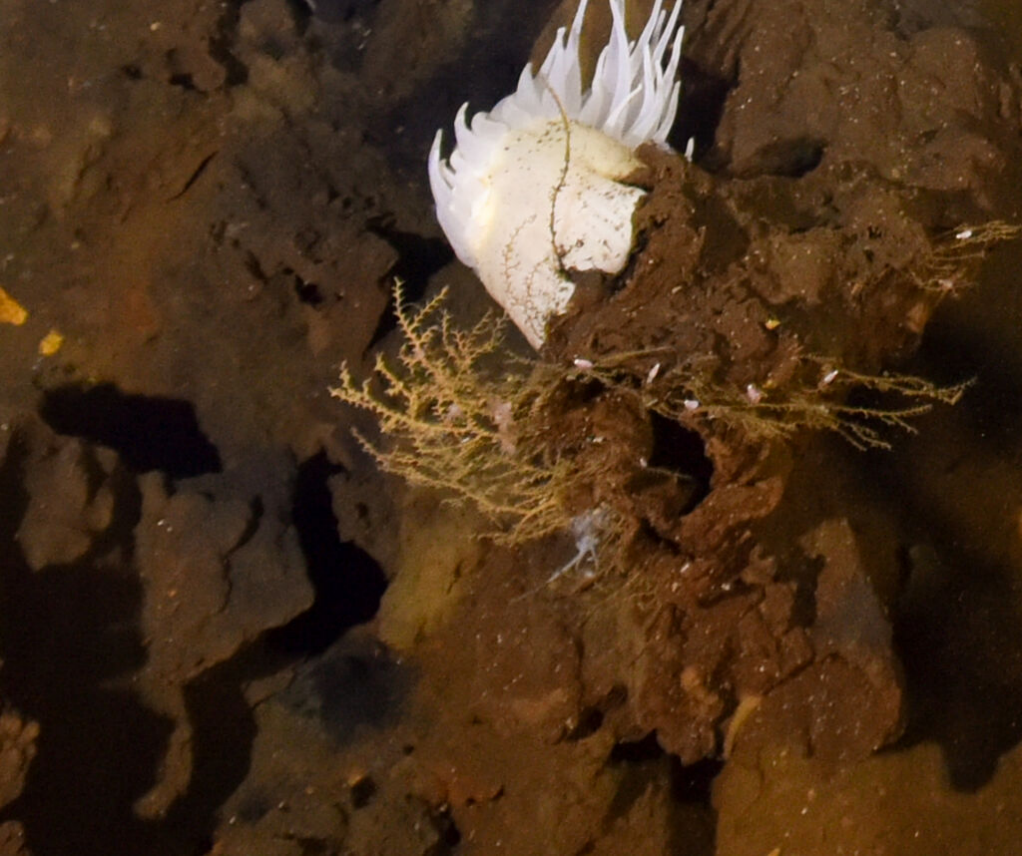
Hydrozoa ord. indet. (DZMB_2021_0060) in situ at the Rodriguez Triple Junction within Vent site 4 in Cluster 5 of the INDEX area. Image corresponds with the data (Image attribution: BGR).

**Figure 136.**
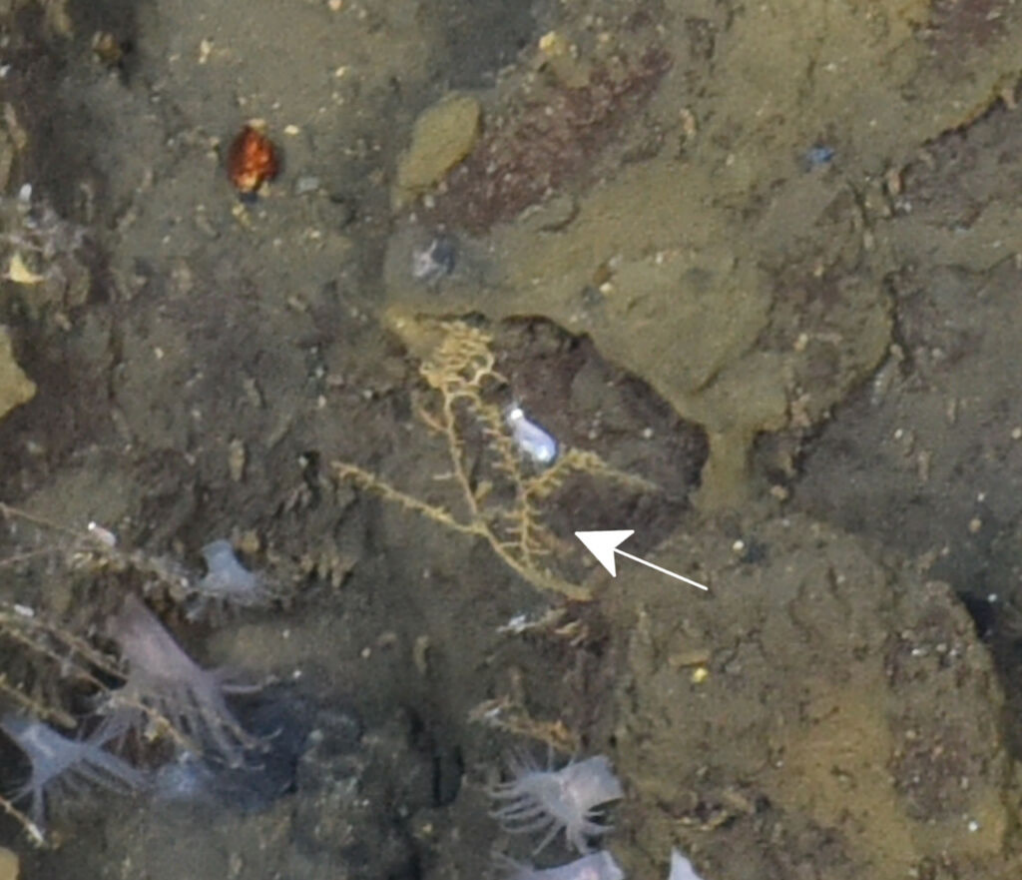
Hydrozoa ord. indet. (DZMB_2021_0061) in situ at the Rodriguez Triple Junction within Vent site 4 in Cluster 5 of the INDEX area. Image corresponds with the data (Image attribution: BGR).

**Figure 137.**
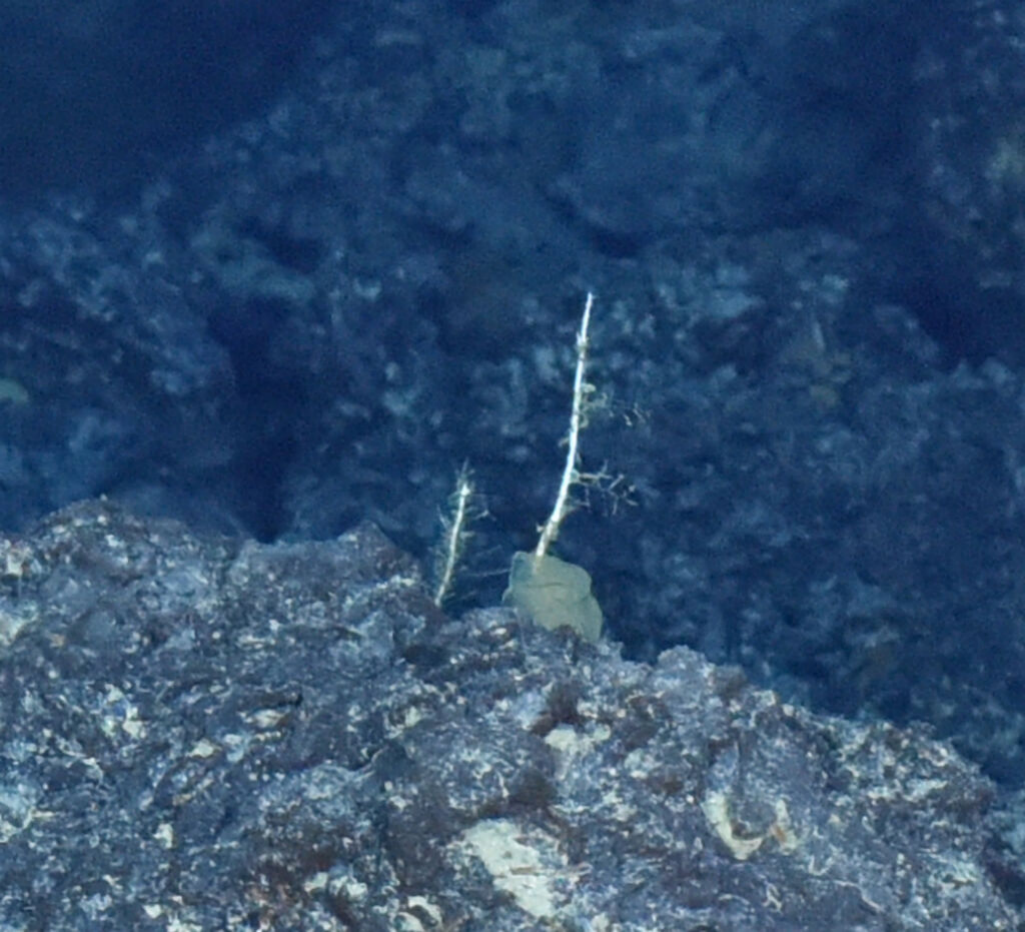
Hydrozoa ord. indet. (DZMB_2021_0062) in situ at the South East Indian Ridge at the border of Vent site 6 in Cluster 12 of the INDEX area. Image corresponds with the data (Image attribution: BGR).

**Figure 138.**
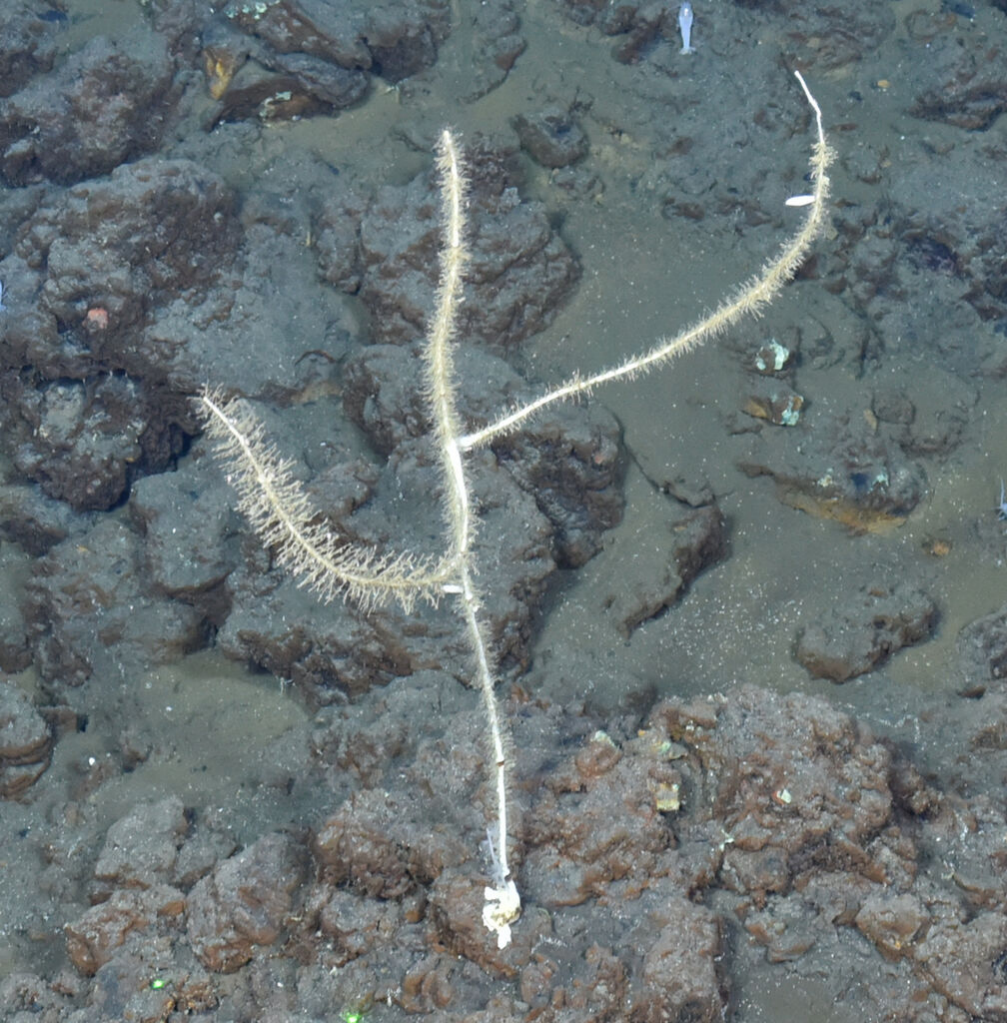
Hydrozoa ord. indet. (DZMB_2021_0063) in situ at the Rodriguez Triple Junction within Vent site 4 in Cluster 5 of the INDEX area. Image corresponds with the data (Image attribution: BGR).

**Figure 139.**
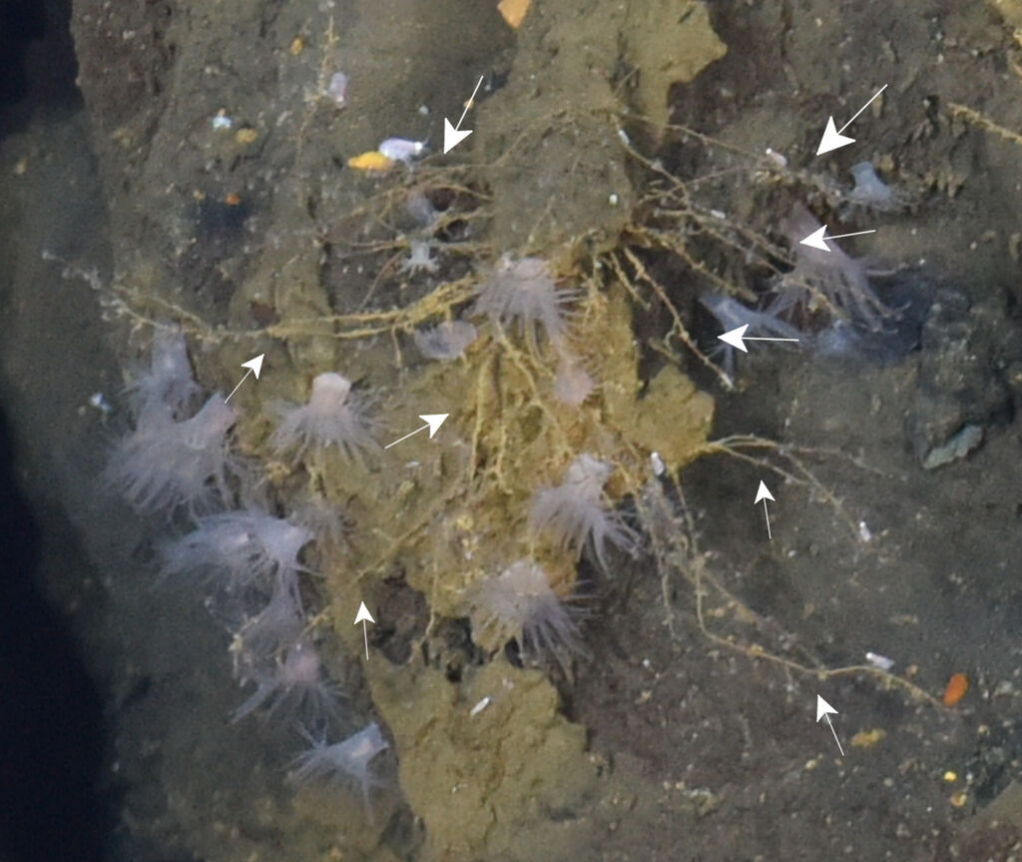
Hydrozoa ord. indet. (DZMB_2021_0064) in situ at the Rodriguez Triple Junction within Vent site 4 in Cluster 5 of the INDEX area. Image corresponds with the data (Image attribution: BGR).

**Figure 140.**
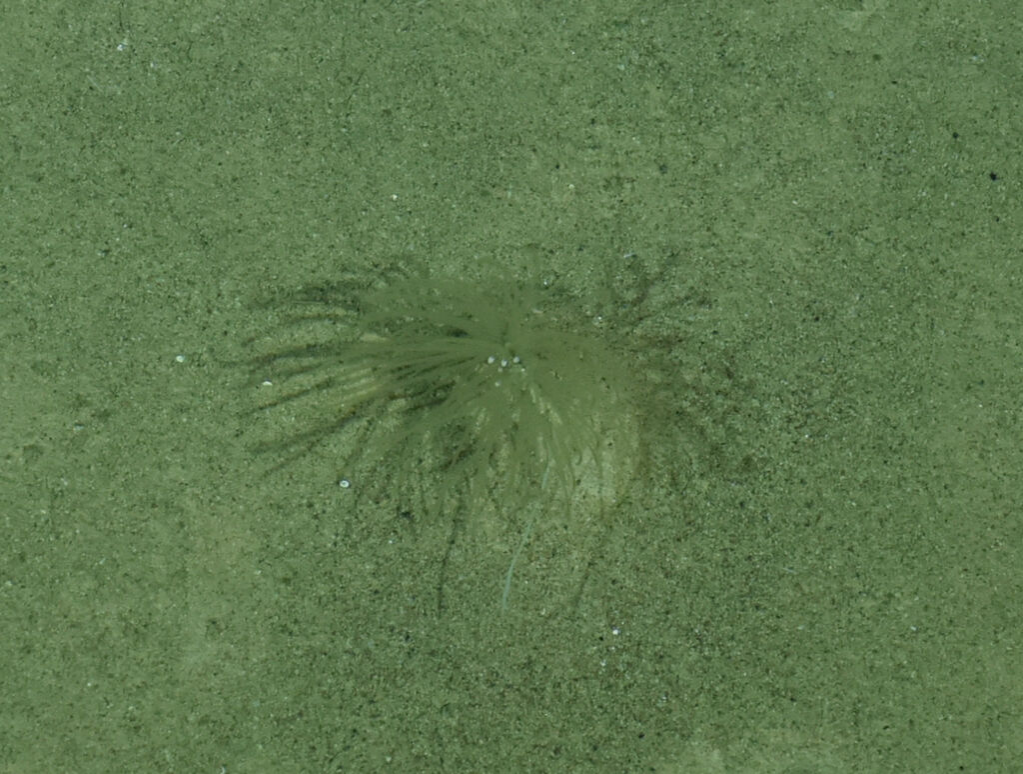
Hydrozoa ord. indet. (DZMB_2021_0065) in situ at the Rodriguez Triple Junction in Cluster 5 of the INDEX area. Image corresponds with the data (Image attribution: BGR).

**Figure 141.**
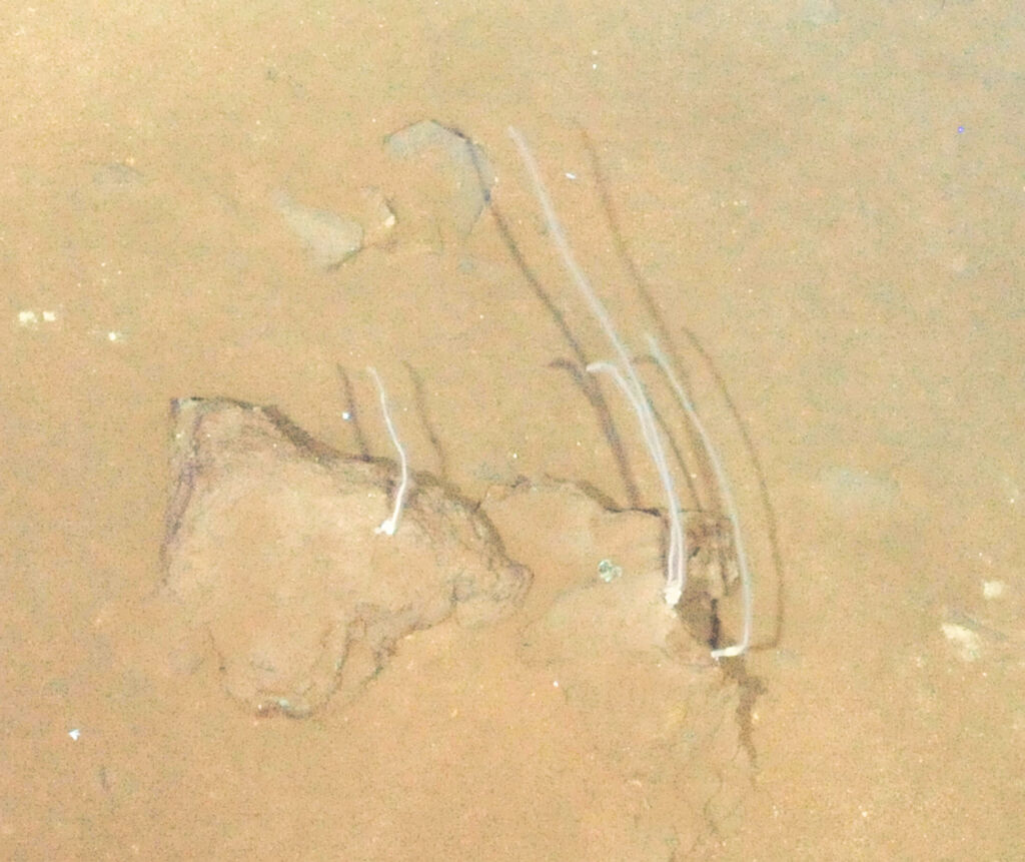
*Candelabrum* sp. indet. in situ at the Central Indian Ridge within the Edmond-Vent site 2-vent site 7 area in Cluster 4 of the INDEX area. Image corresponds with the data (Image attribution: BGR).

**Figure 142.**
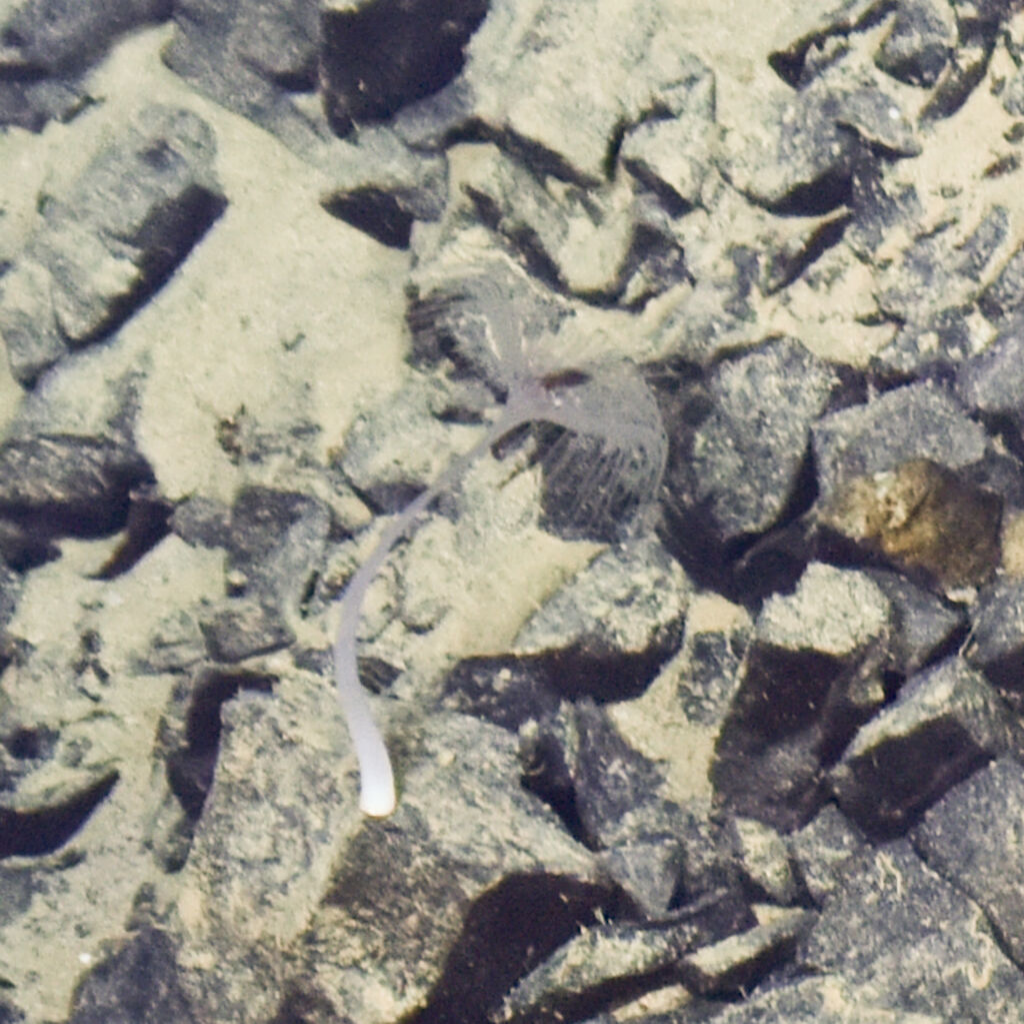
Corymorphidae gen. indet. in situ at the South East Indian Ridge at the border of Vent site 5 in Cluster 11 of the INDEX area. Image corresponds with the data (Image attribution: BGR).

**Figure 143.**
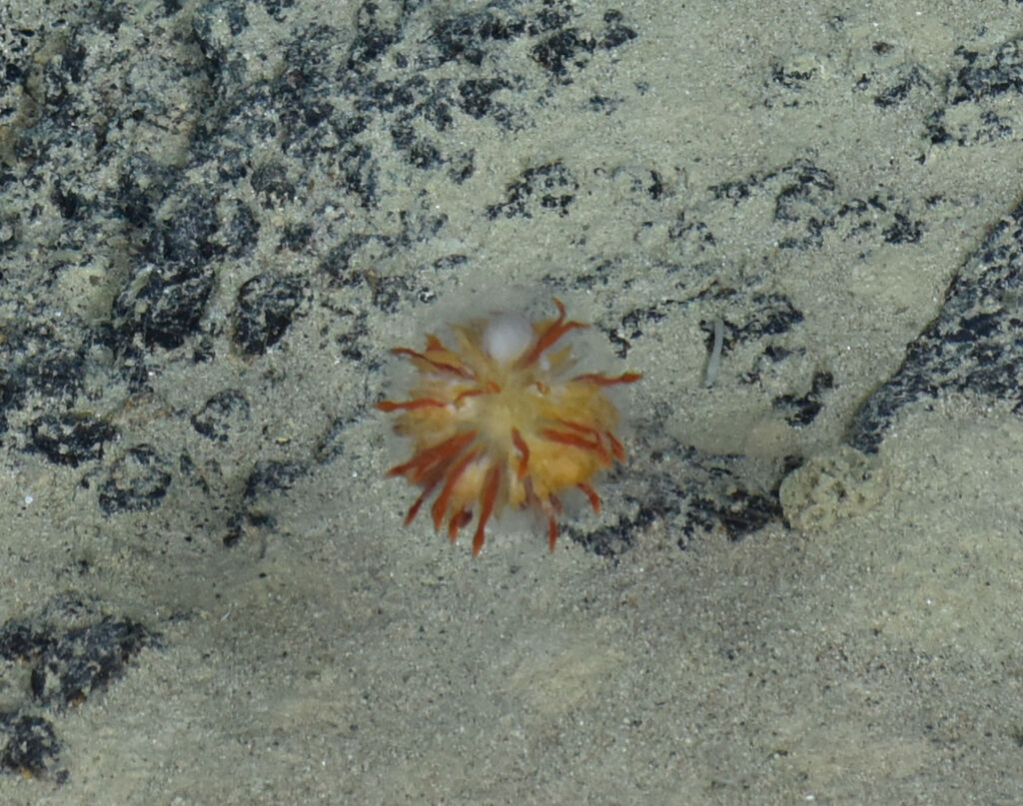
Siphonophorae Rhodaliidae *Thermopalia* gen. inc. in situ at the Rodriguez Triple Junction in Cluster 5 of the INDEX area. Image corresponds with the data (Image attribution: BGR).

**Figure 144.**
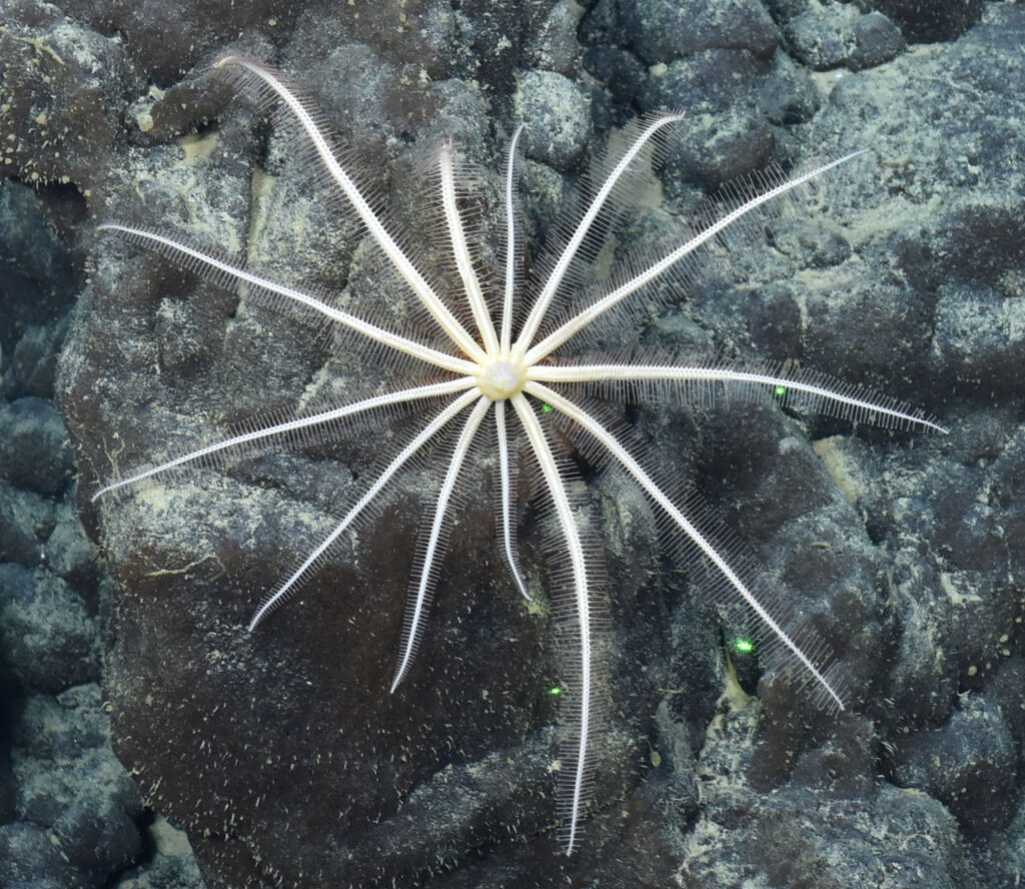
*Hymenodiscus* gen. inc. in situ at the Rodriguez Triple Junction in Cluster 5 of the INDEX area. Image corresponds with the data (Image attribution: BGR).

**Figure 145.**
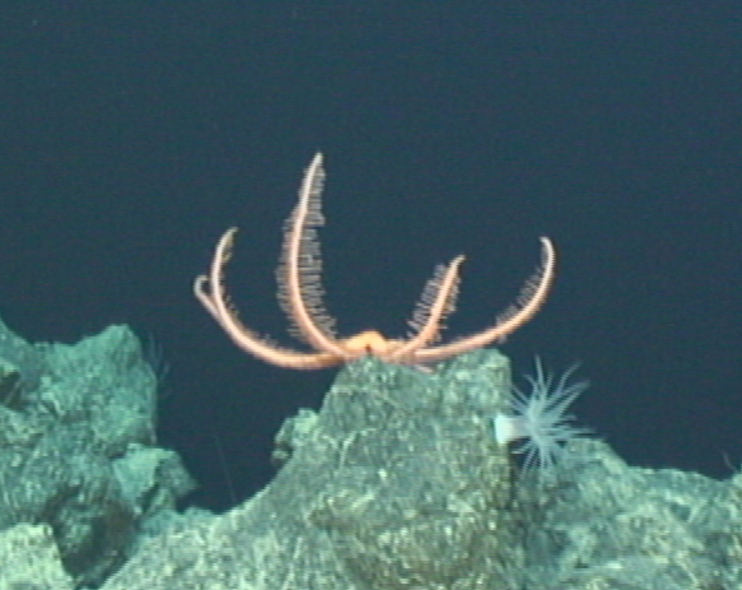
Freyellidae fam. inc. in situ in the surrounding area of the Pelagia hydrothermal vent field in Cluster 8 of the INDEX area. Image corresponds with the data (Image attribution: BGR).

**Figure 146.**
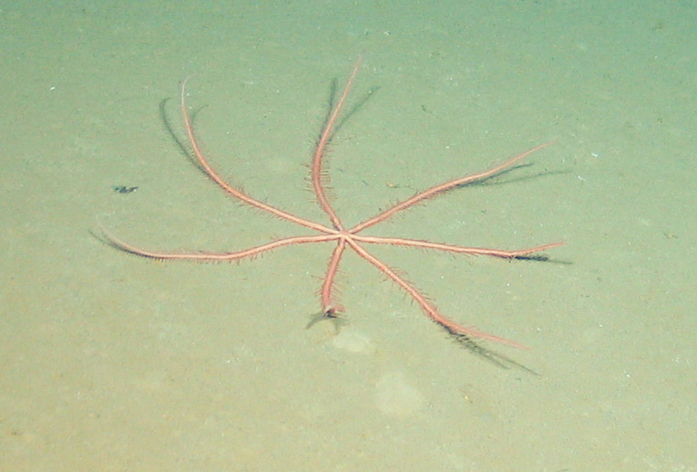
*Freyastera* gen. inc. in situ in the surrounding area of the Edmond hydrothermal vent field in Cluster 4 of the INDEX area. Image corresponds with the data (Image attribution: BGR and GEOMAR).

**Figure 147.**
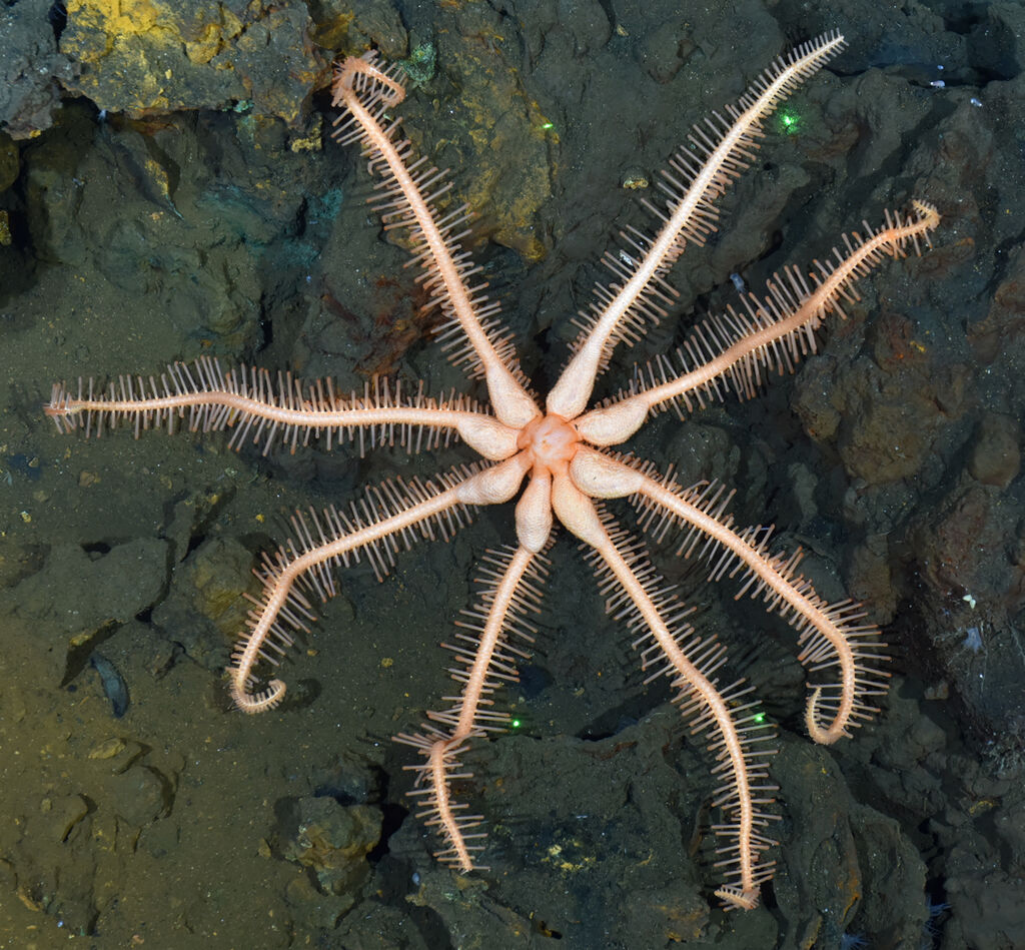
*Freyella* gen. inc. in situ at the border of the vent site 4 hydrothermal vent field in Cluster 5 of the INDEX area. Image corresponds with the data (Image attribution: BGR).

**Figure 148.**
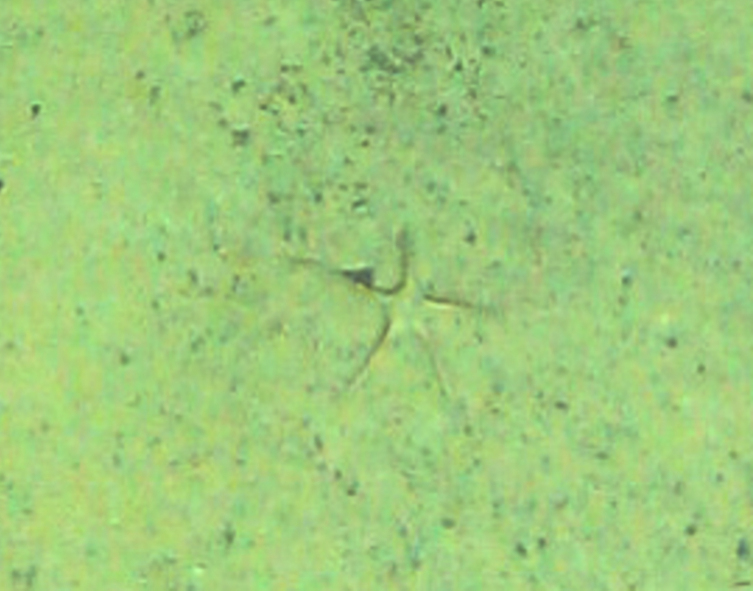
*Styracaster* gen. inc. in situ in the MESO area outside the INDEX area. Image corresponds with the data (Image attribution: BGR).

**Figure 149.**
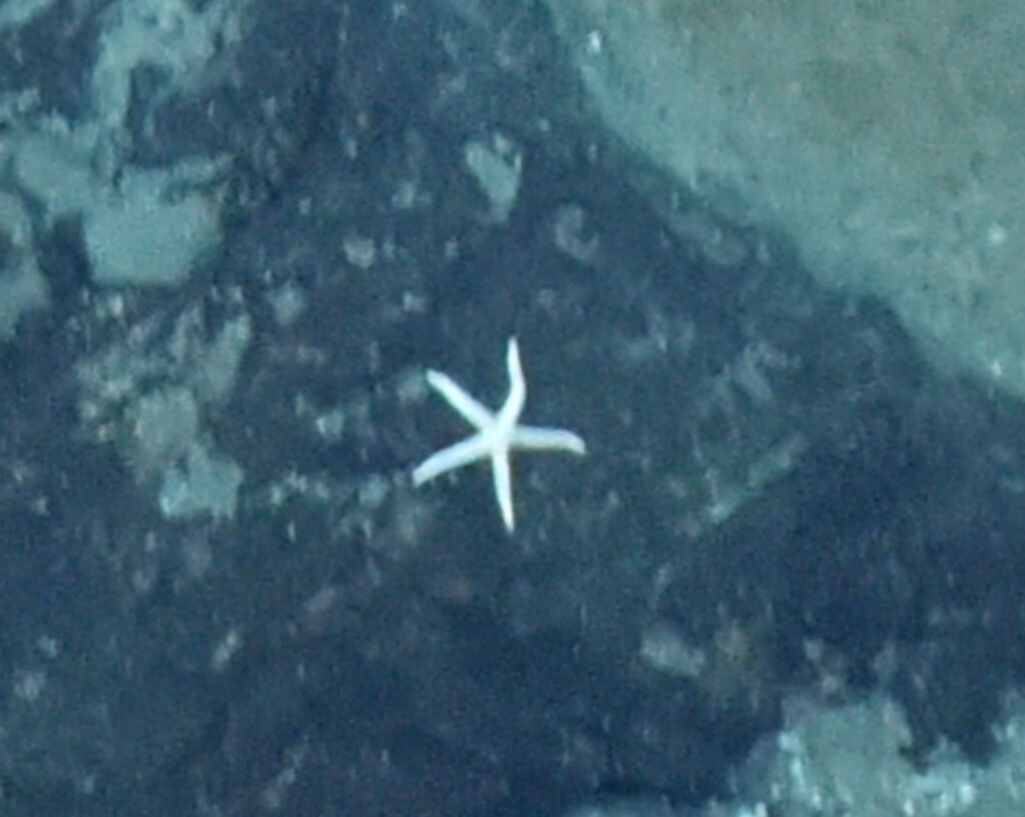
*Henricia* gen. inc. in situ at the border of the vent site 4 hydrothermal vent field in Cluster 5 of the INDEX area. Image corresponds with the data (Image attribution: BGR).

**Figure 150.**
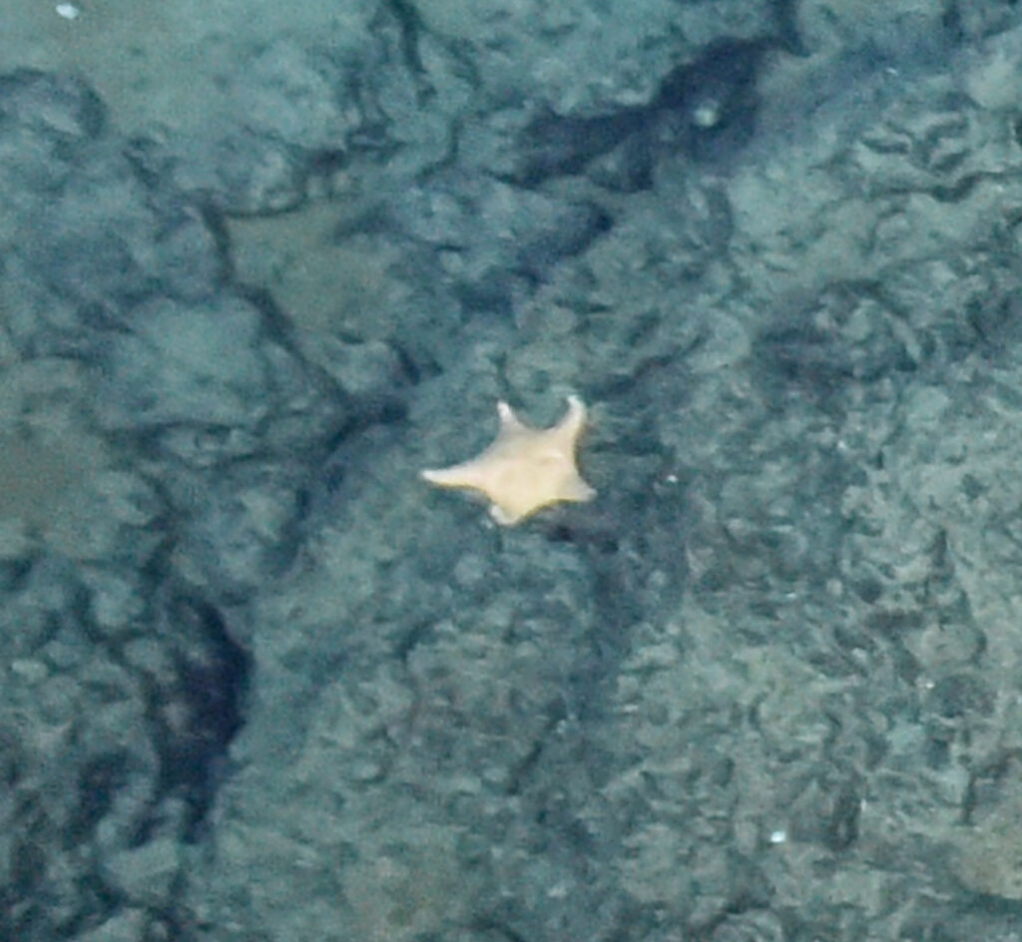
Goniasteridae gen. indet. (DZMB_2021_0066) in situ at the border of the vent site 6 hydrothermal vent field in Cluster 12 of the INDEX area. Image corresponds with the data (Image attribution: BGR).

**Figure 151.**
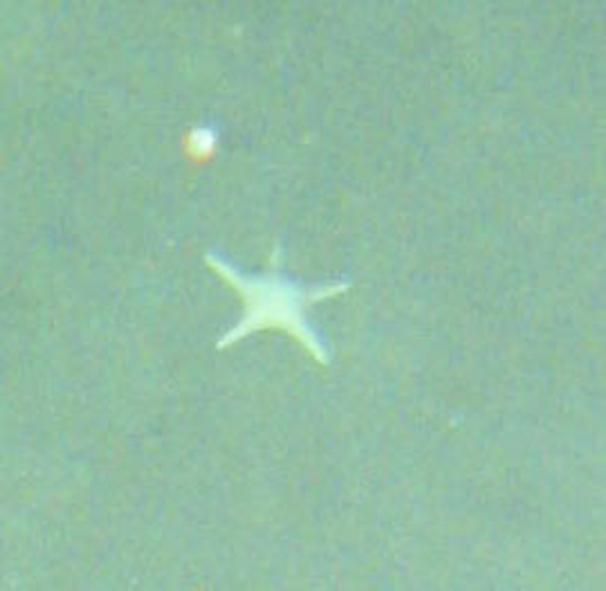
Goniasteridae gen. indet. (DZMB_2021_0067) in situ in the surrounding area of the Edmond hydrothermal vent field in Cluster 4 of the INDEX area. Image corresponds with the data (Image attribution: BGR).

**Figure 152.**
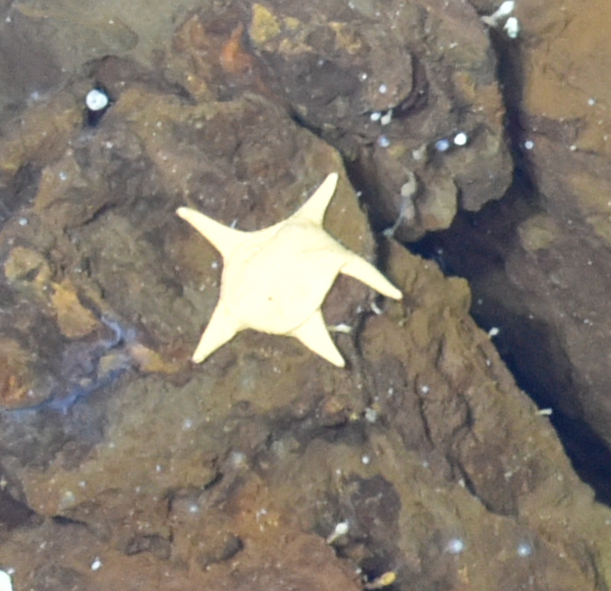
*Circeaster* gen. inc. in situ at the South East Indian Ridge in Cluster 12 of the INDEX area. Image corresponds with the data (Image attribution: BGR).

**Figure 153.**
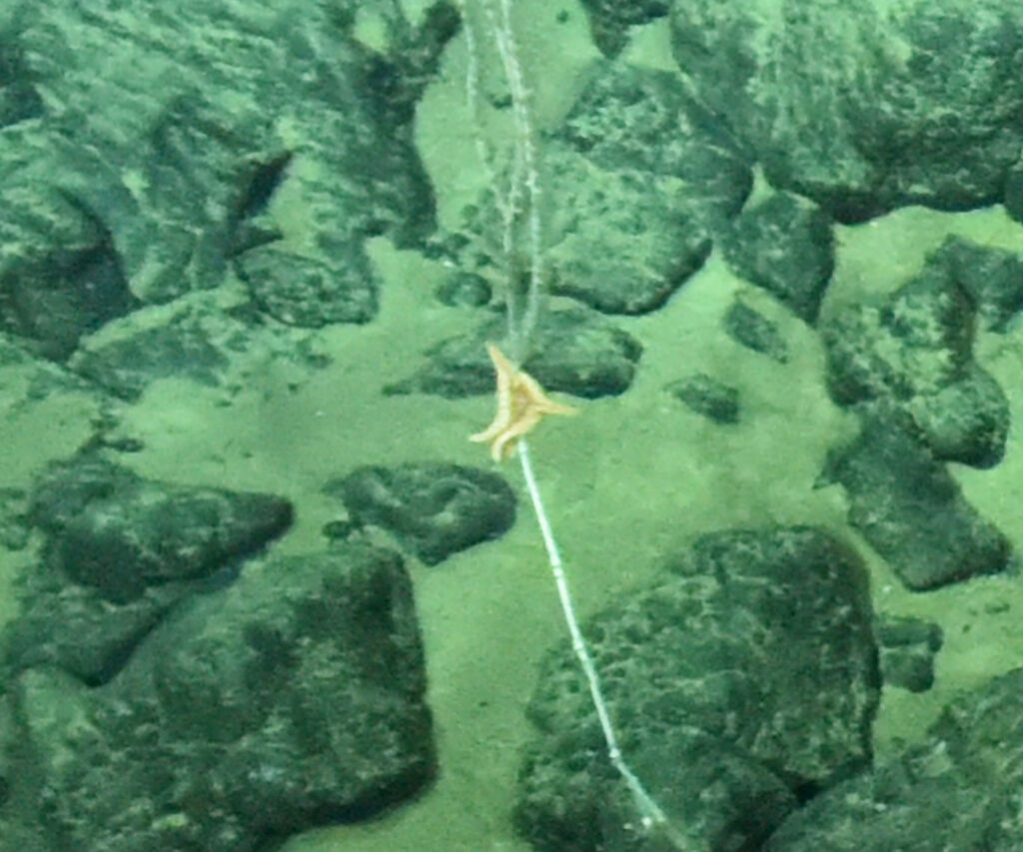
*Evoplosoma* gen. inc. in situ at the border of the vent site 6 hydrothermal vent field in Cluster 12 of the INDEX area. Image corresponds with the data (Image attribution: BGR).

**Figure 154.**
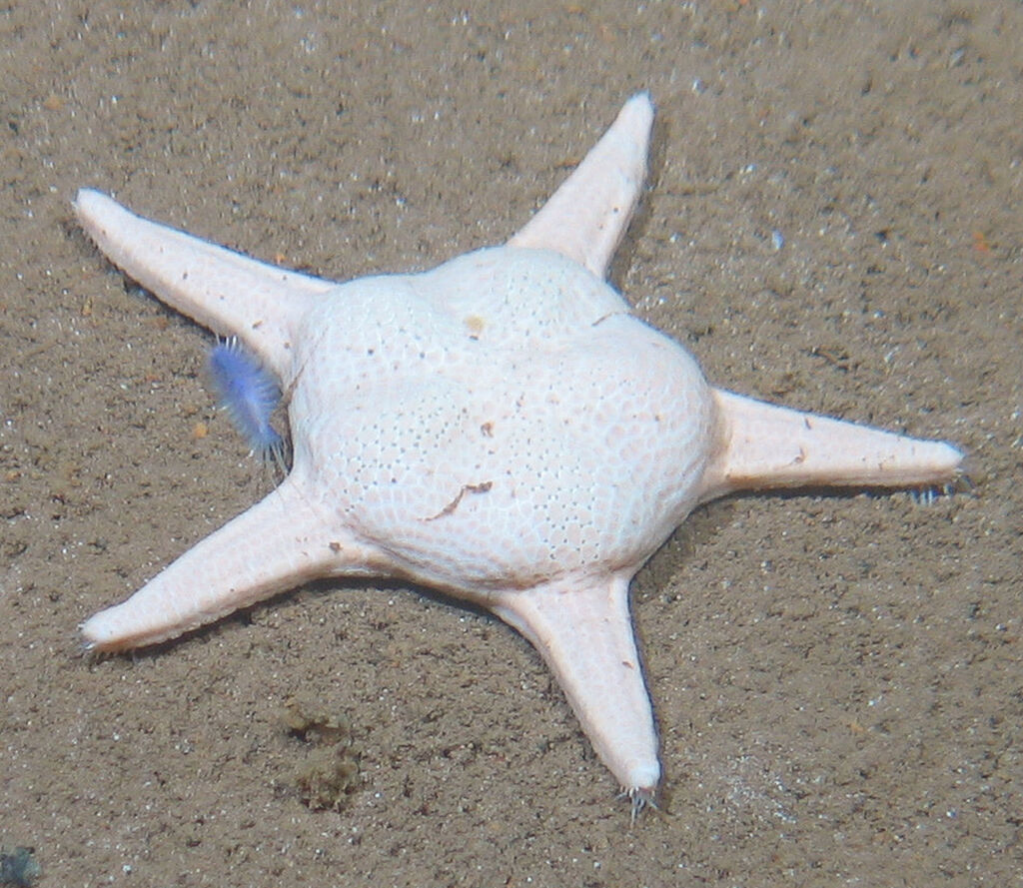
*Lydiasterjohannae* sp. inc. in situ in the surrounding area of the Edomd hydrothermal vent field in Cluster 4 of the INDEX area. Image corresponds with the data (Image attribution: BGR and GEOMAR).

**Figure 155.**
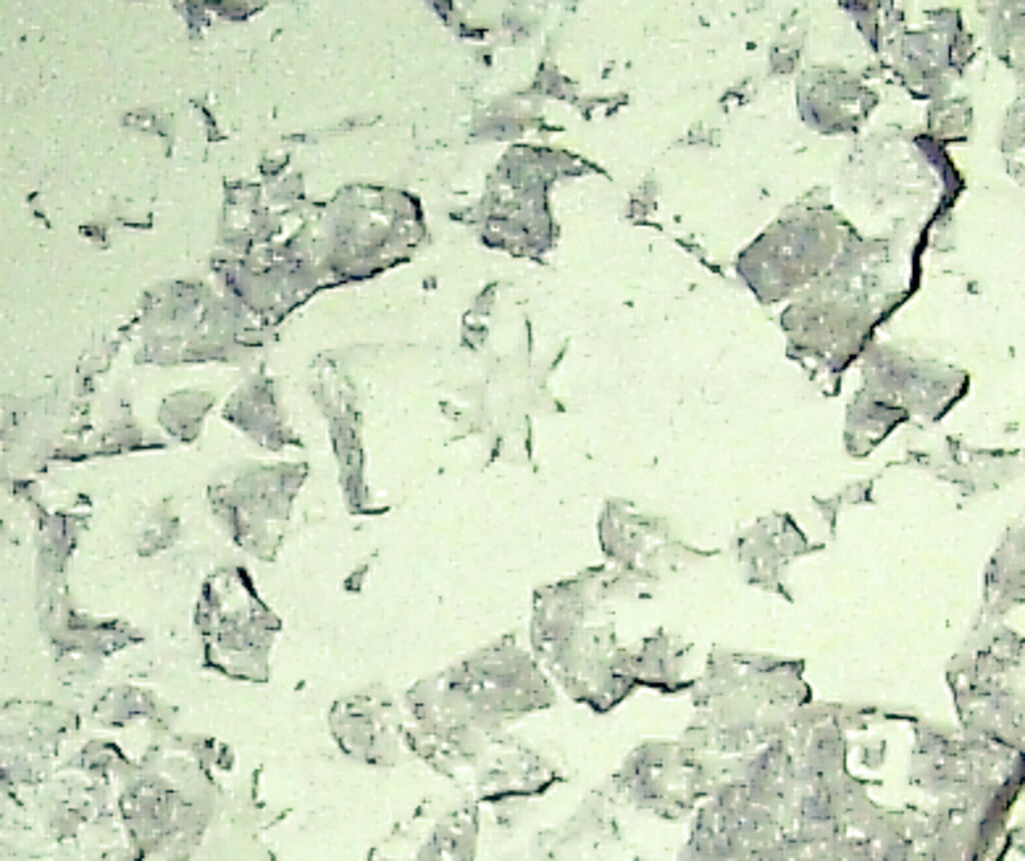
Solasteridae fam. inc. in situ in the surrounding area of the Pelagia hydrothermal vent field in Cluster 8 of the INDEX area. Image corresponds with the data (Image attribution: BGR).

**Figure 156.**
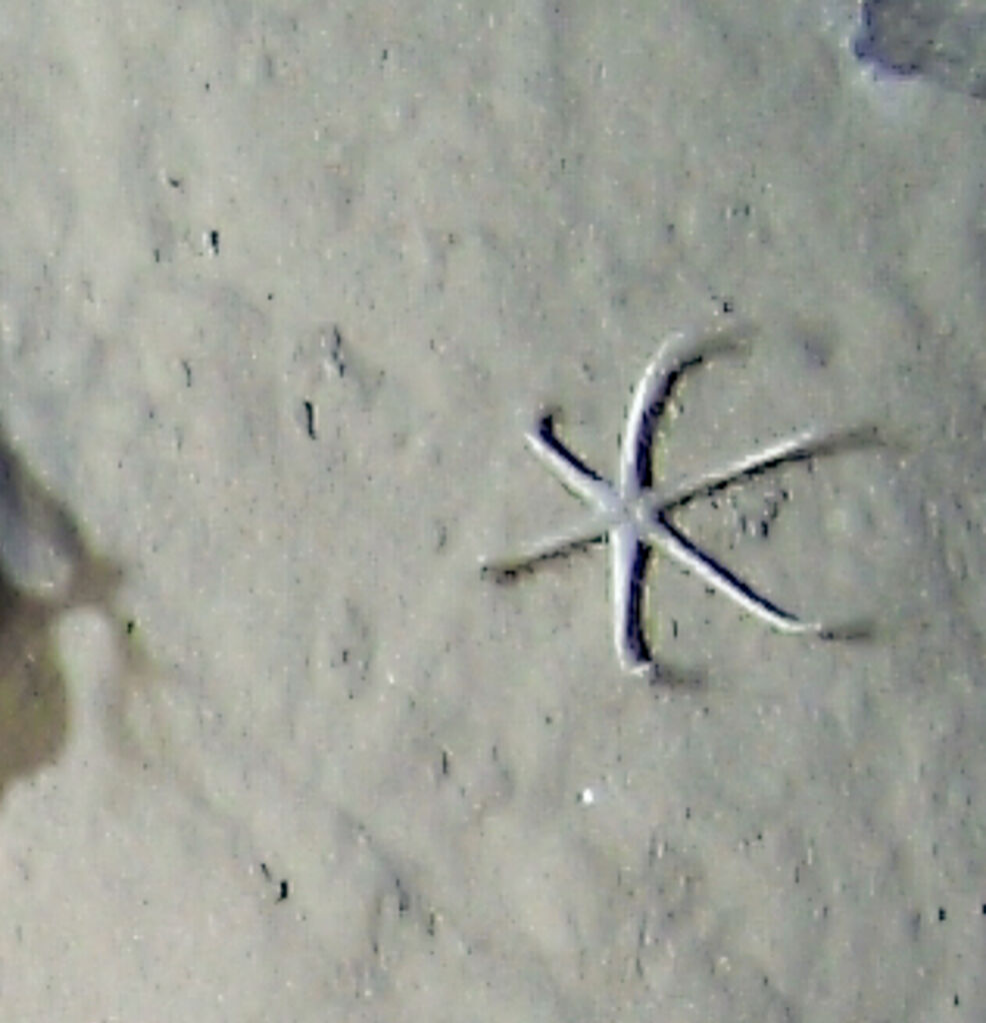
*Asthenactis* gen. inc. in situ at the South East Indian Ridge in Cluster 9 of the INDEX area. Image corresponds with the data (Image attribution: BGR).

**Figure 157.**
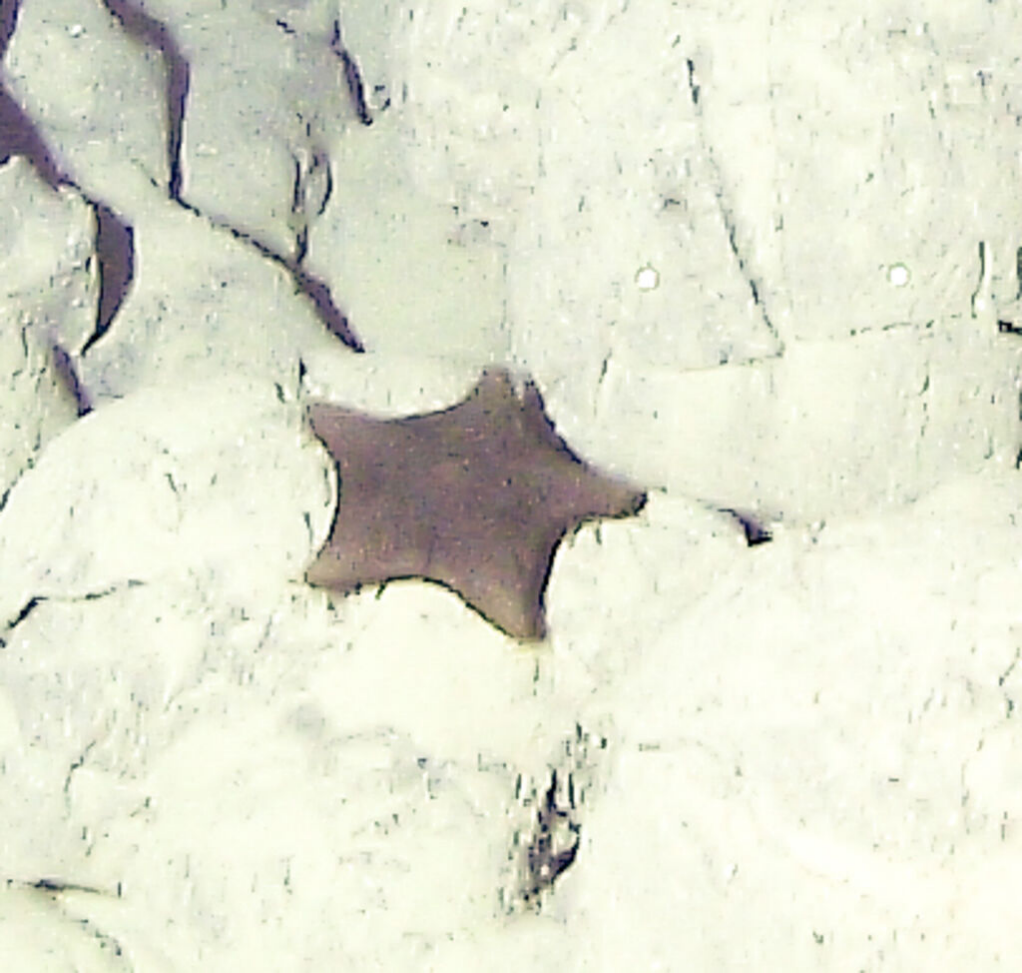
*Hymenaster* sp. indet. in situ at the South East Indian Ridge in Cluster 6 of the INDEX area. Image corresponds with the data (Image attribution: BGR).

**Figure 158.**
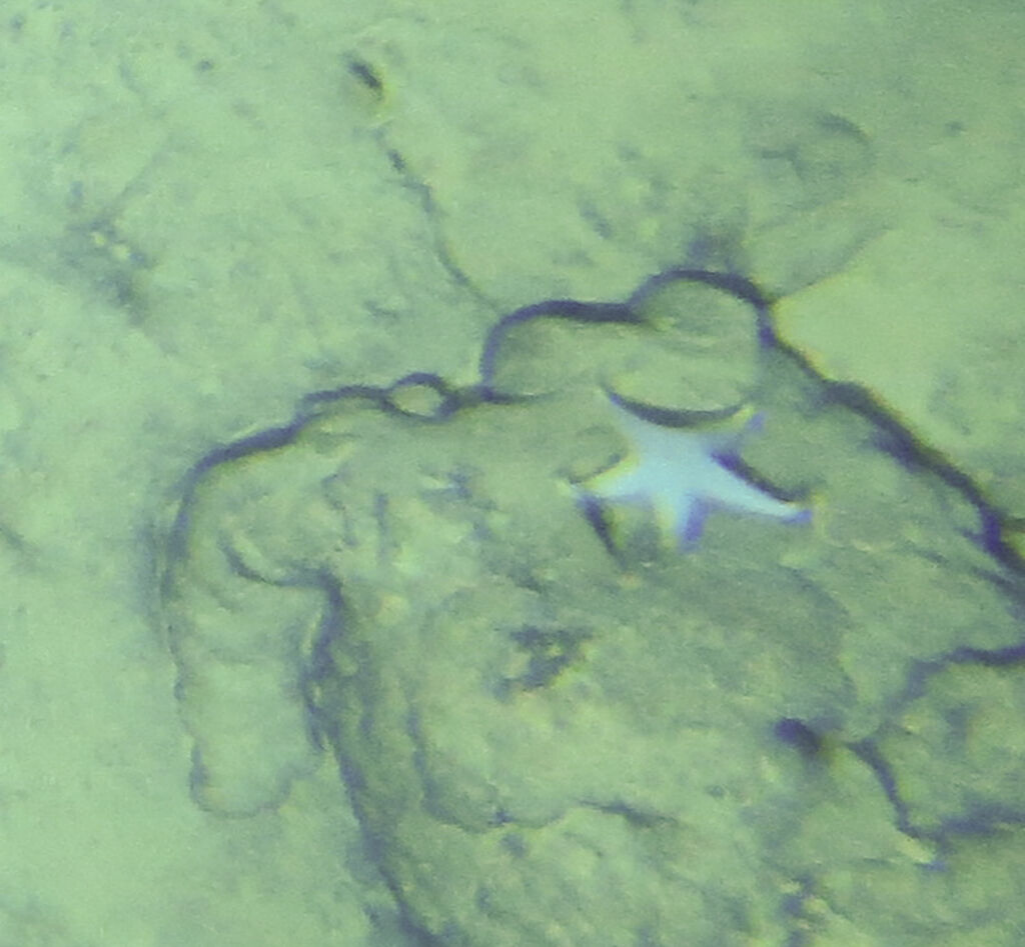
*Pteraster* gen. inc. in situ at the South East Indian Ridge in Cluster 11 of the INDEX area. Image corresponds with the data (Image attribution: BGR).

**Figure 159.**
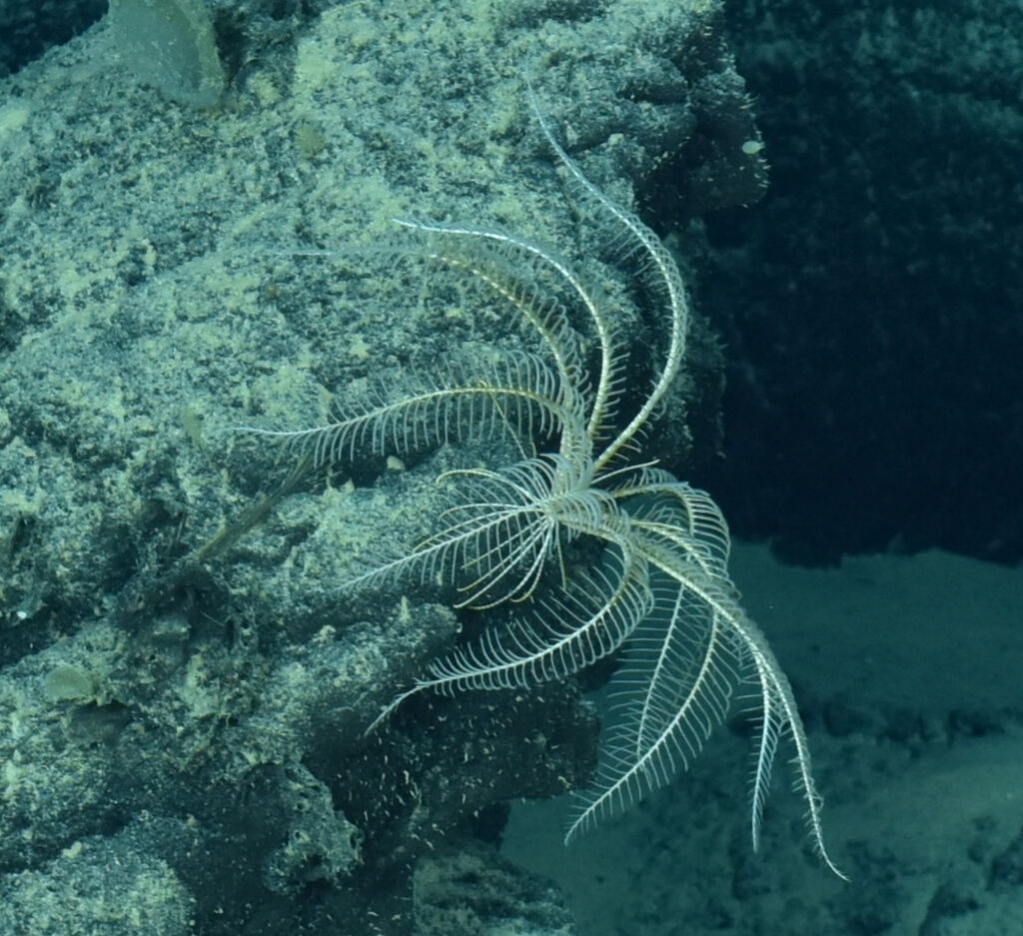
Antedonidae gen. indet. (DZMB_2021_0068) in situ in the surrounding area of the vent site 5 hydrothermal vent field in Cluster 11 of the INDEX area. Image corresponds with the data (Image attribution: BGR).

**Figure 160.**
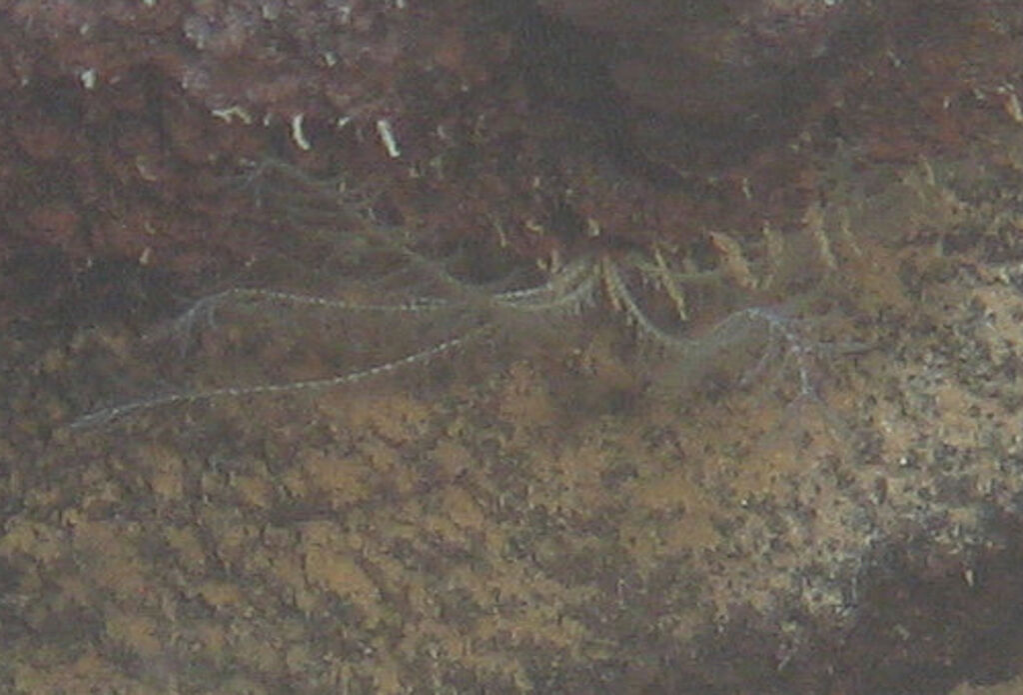
Antedonidae fam. inc. (DZMB_2021_0069) in situ in the surrounding area of the Edmond hydrothermal vent field in Cluster 4 of the INDEX area. Image corresponds with the data (Image attribution: BGR and GEOMAR).

**Figure 161.**
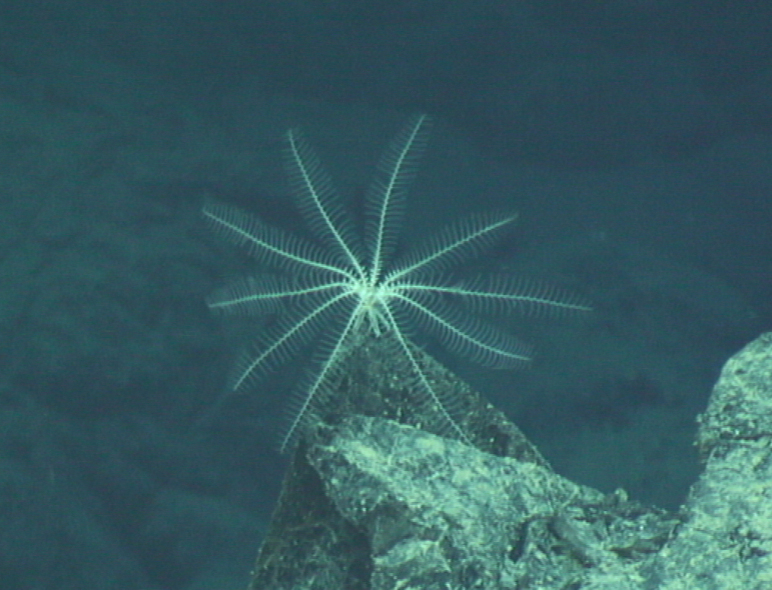
*Bathymetra* gen. inc. in situ in the surrounding area of the Pelagia hydrothermal vent field in Cluster 8 of the INDEX area. Image corresponds with the data (Image attribution: BGR).

**Figure 162.**
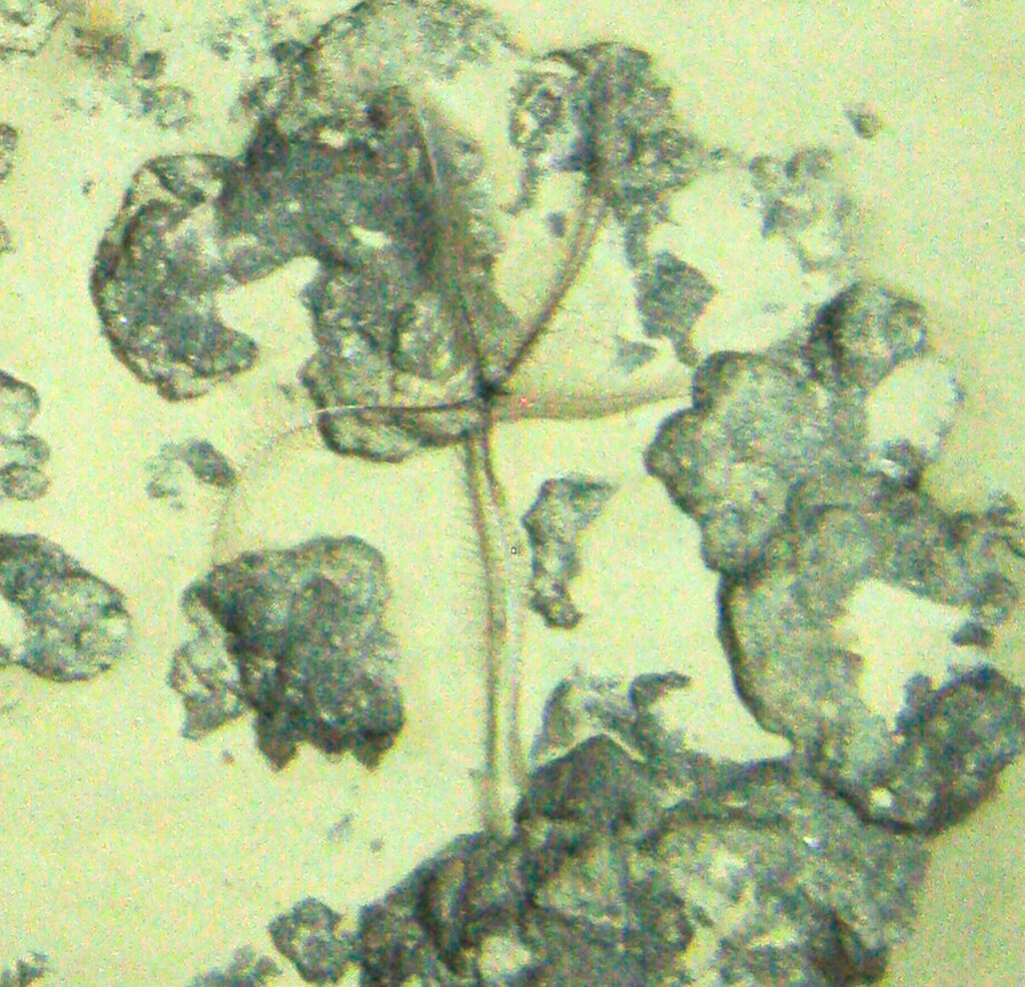
*Pentametrocrinus* sp. indet. in situ in the MESO area outside the INDEX area. Image corresponds with the data (Image attribution: BGR).

**Figure 163.**
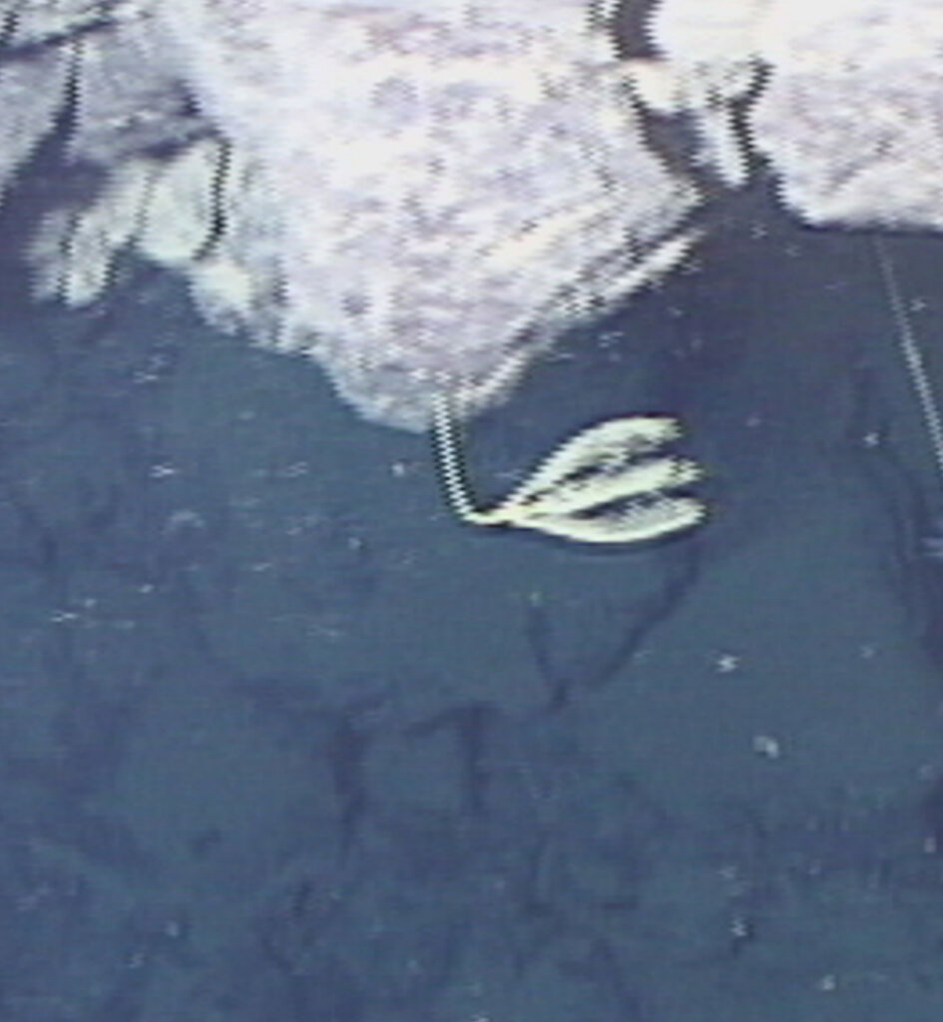
Hyocrinidae gen. indet. in situ in the surrounding area of the Pelagia hydrothermal vent field in Cluster 8 of the INDEX area. Image corresponds with the data (Image attribution: BGR).

**Figure 165.**
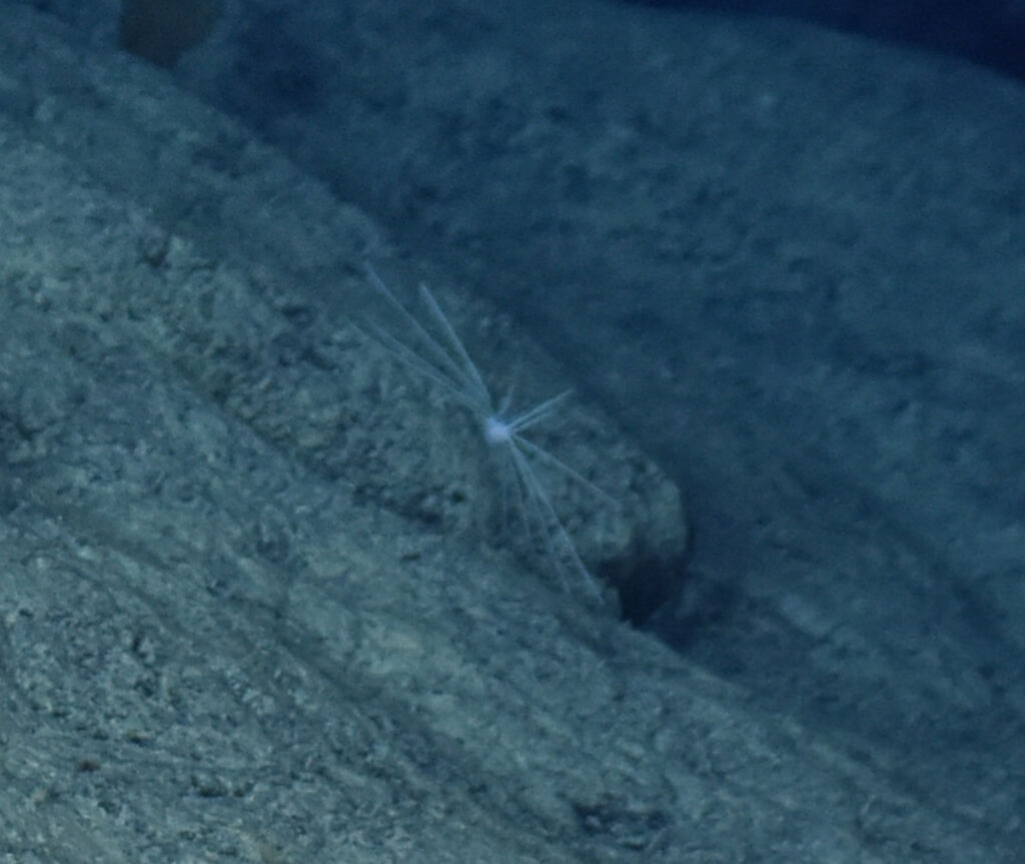
Cidaroida fam. indet. in situ in the surrounding area of the Rodriguez Triple Junction in Cluster 5 of the INDEX area. Image corresponds with the data (Image attribution: BGR).

**Figure 168.**
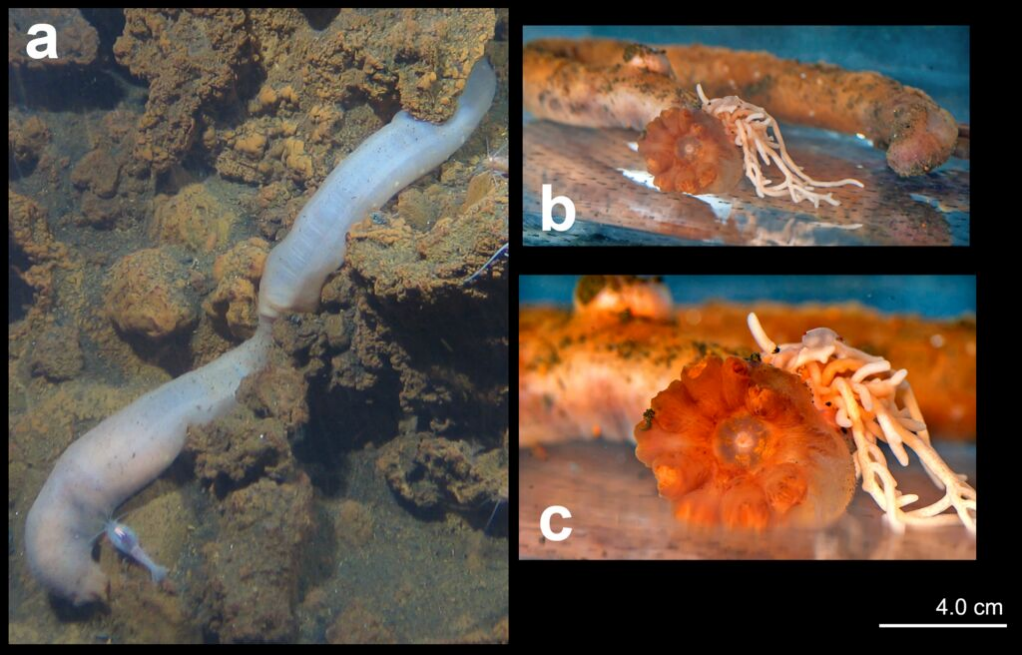
*Chiridotahydrothermica* sp. inc. in situ (a) and sampled specimen (b+c) within the Edmond hydrothermal vent field in Cluster 4 of the INDEX area. Image corresponds with the data (Image attribution: BGR and GEOMAR).

**Figure 169.**
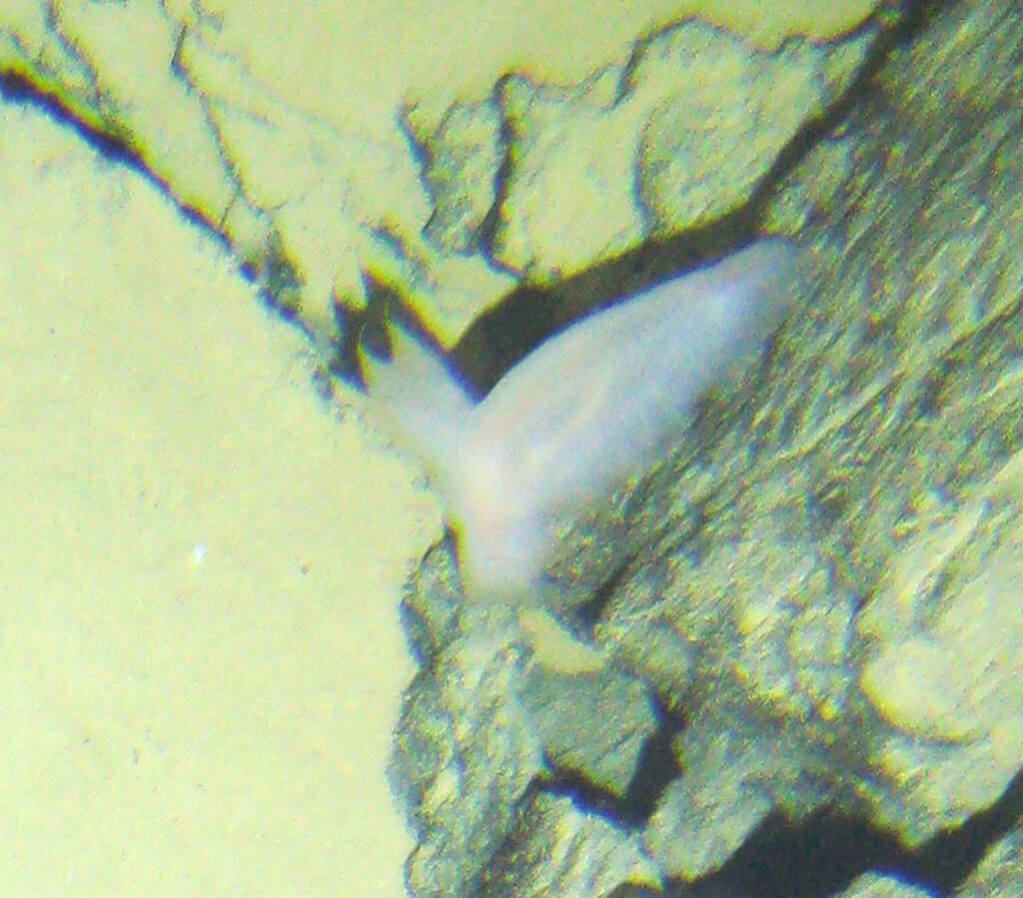
Elpidiidae gen. indet. (DZMB_2021_0070) in situ in the MESO area outside the INDEX area. Image corresponds with the data (Image attribution: BGR).

**Figure 170.**
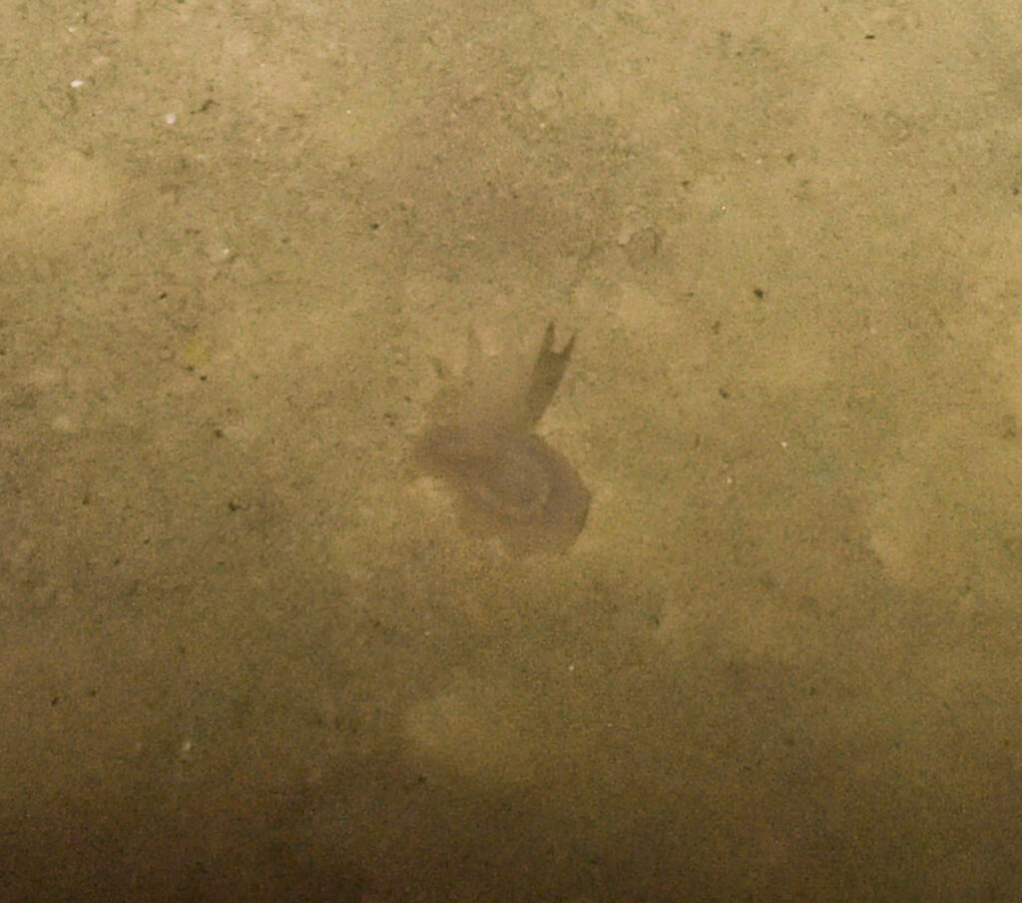
Elpidiidae gen. indet. (DZMB_2021_0071) in situ in the area of the Rodriguez Triple Junction in Cluster 5 of the INDEX area. Image corresponds with the data (Image attribution: BGR).

**Figure 171.**
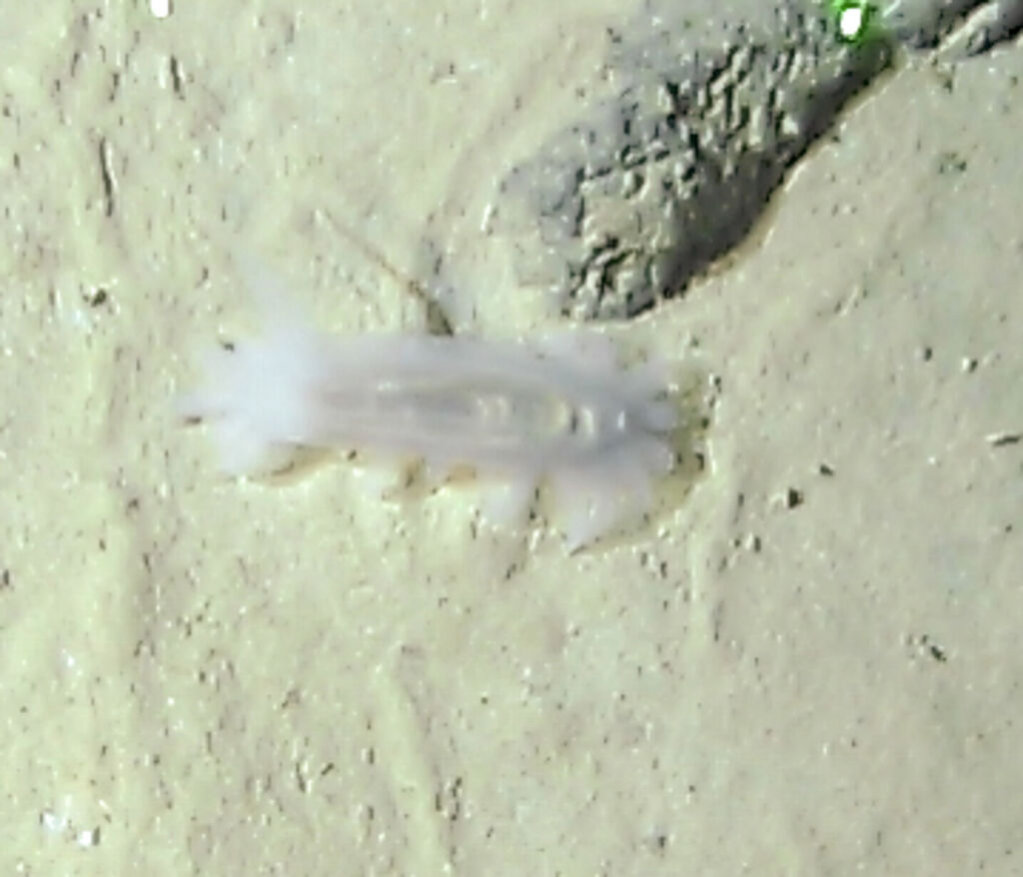
Elpidiidae gen. indet. (DZMB_2021_0072) in situ at the South East Indian Ridge in Cluster 9 of the INDEX area. Image corresponds with the data (Image attribution: BGR).

**Figure 172.**
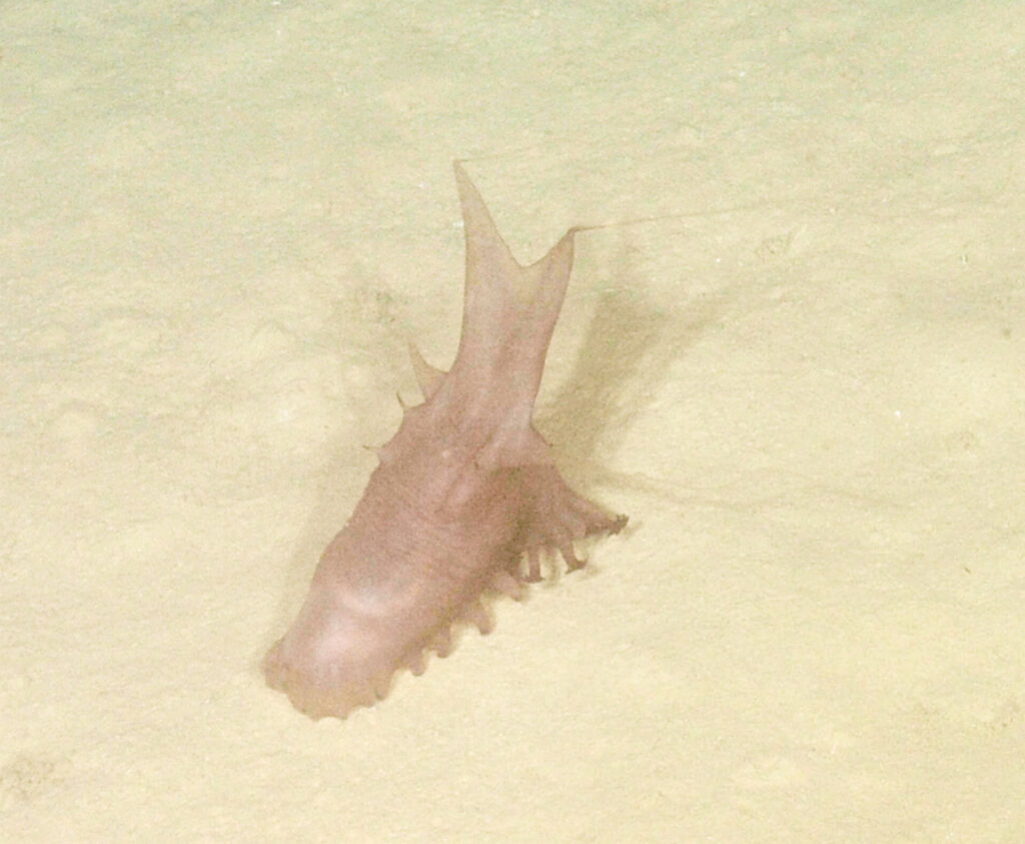
*Peniagonepurpurea* in situ at the border of the Edmond hydrothermal vent field in Cluster 4 of the INDEX area. Image corresponds with the data (Image attribution: BGR).

**Figure 173.**
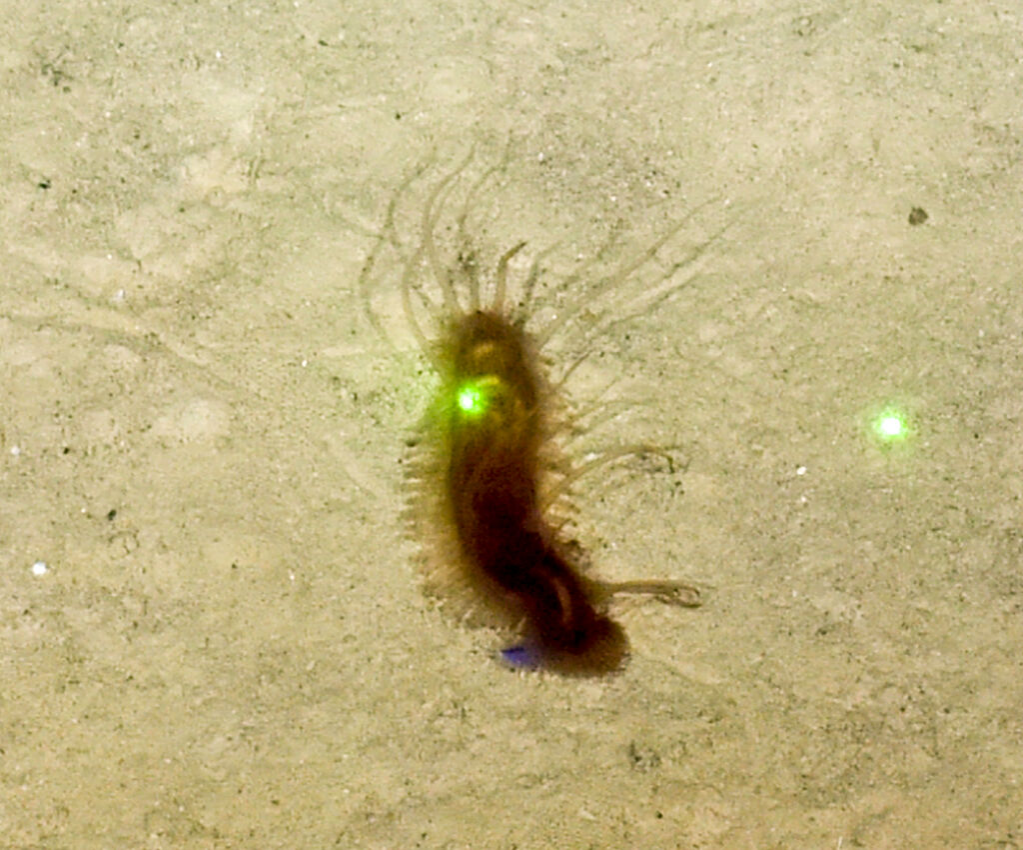
Laetmogonidae gen. indet. in situ at the South East Indian Ridge in Cluster 12 of the INDEX area. Image corresponds with the data (Image attribution: BGR).

**Figure 174.**
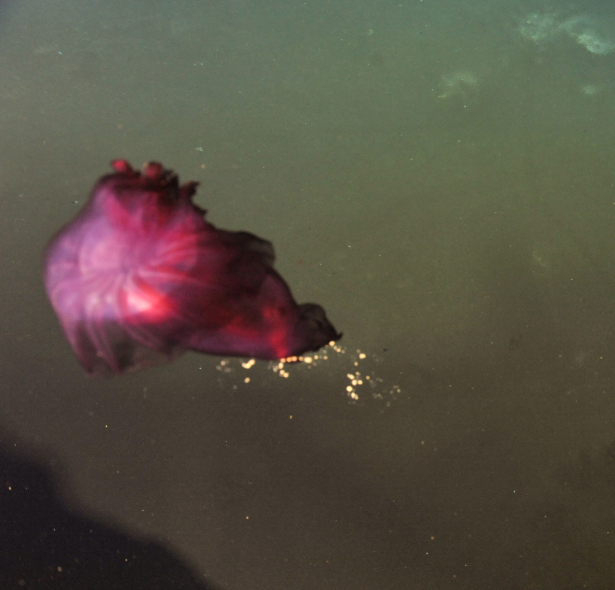
*Enypniasteseximia* in situ within the Edmond-vent site 2-vent site 7 area field in Cluster 4 of the INDEX area. Image corresponds with the data (Image attribution: BGR).

**Figure 175.**
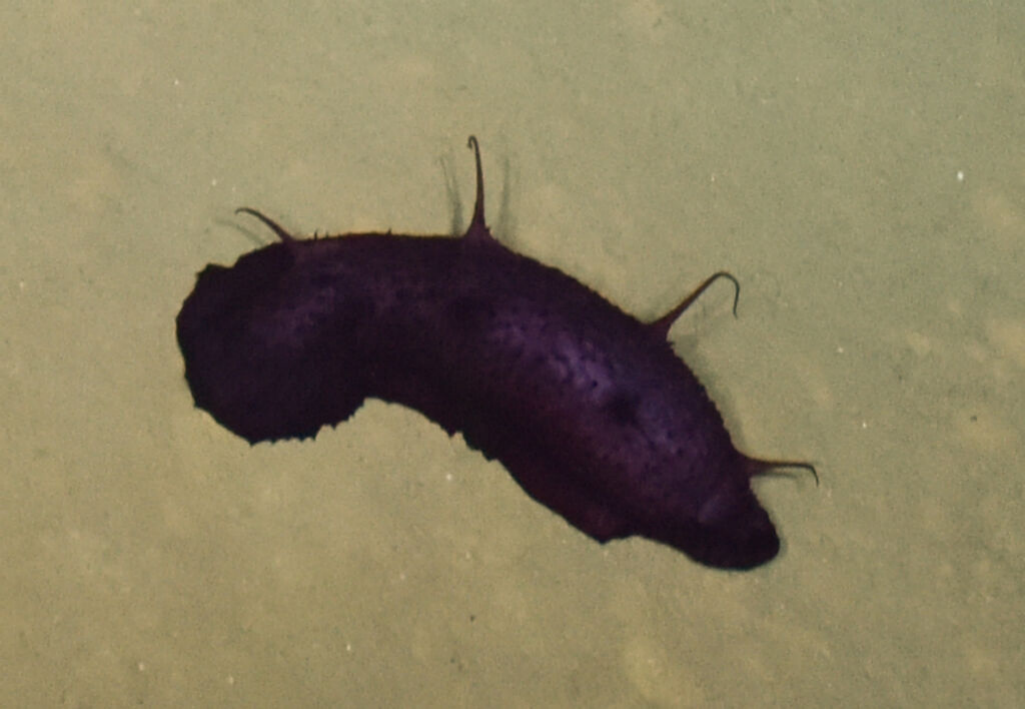
*Benthodytes* sp. indet. in situ at the Rodriguez Triple Junction in Cluster 5 of the INDEX area. Image corresponds with the data (Image attribution: BGR).

**Figure 176.**
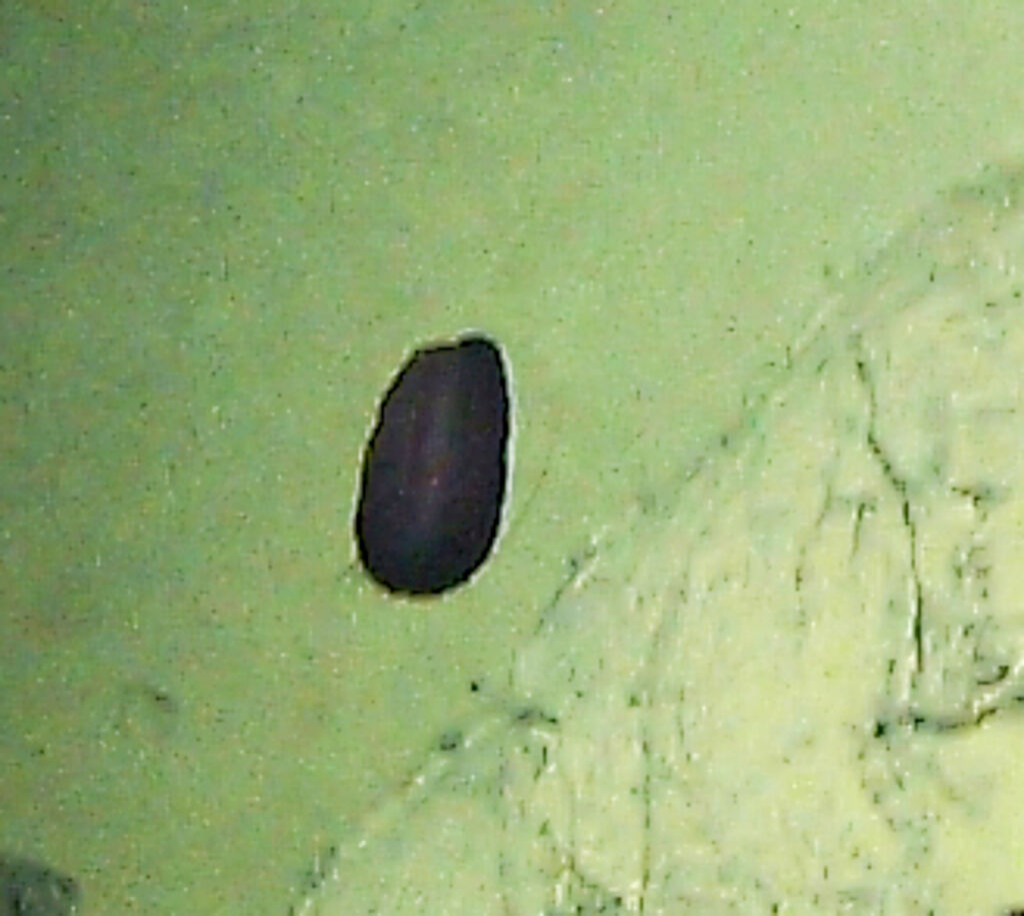
*Benthothuria* gen. inc. in situ at the South East Indian Ridge in Cluster 6 of the INDEX area. Image corresponds with the data (Image attribution: BGR).

**Figure 177.**
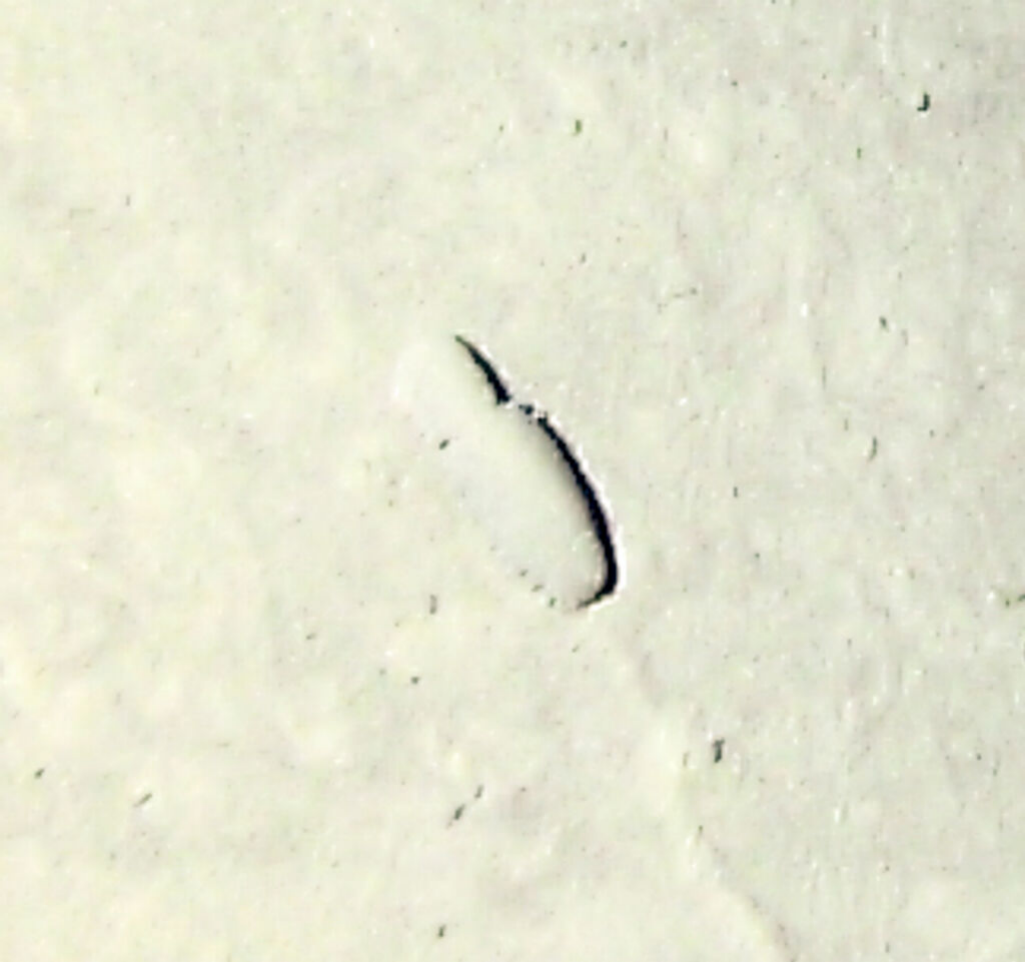
*Pseudostichopus* gen. inc. (DZMB_2021_0073) in situ at the South East Indian Ridge in Cluster 9 of the INDEX area. Image corresponds with the data (Image attribution: BGR).

**Figure 178.**
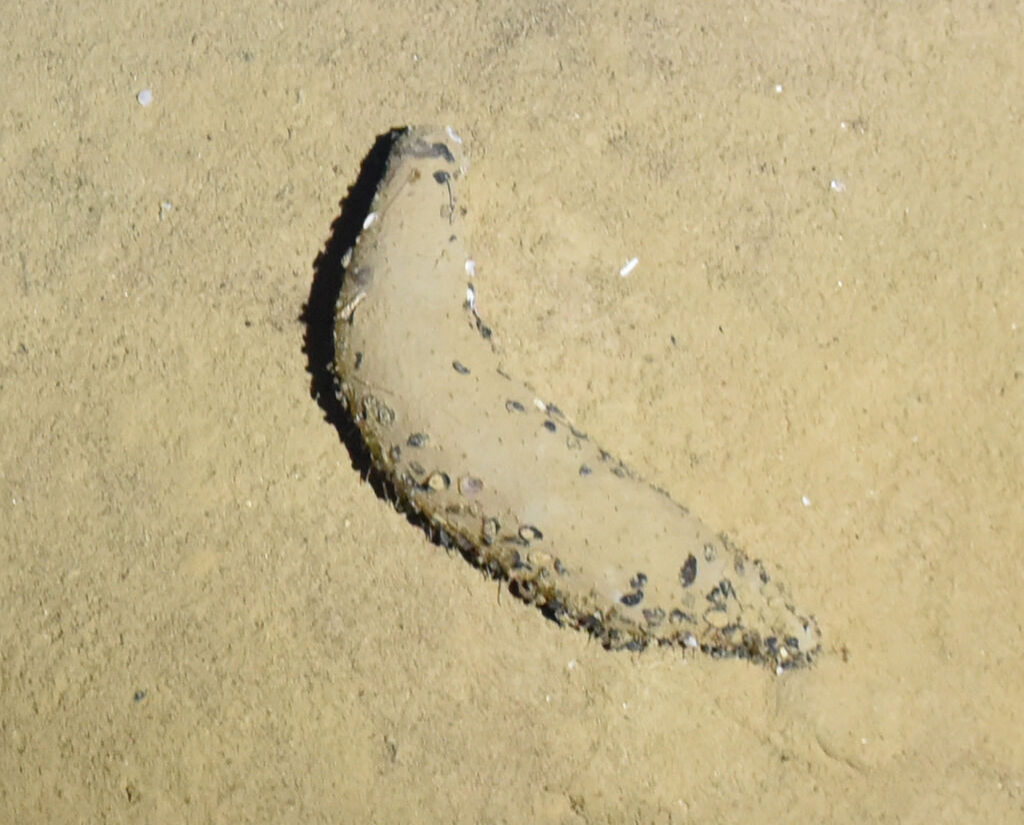
*Pseudostichopus* sp. indet. (DZMB_2021_0074) in situ in the surrounding area of the vent site 6 hydrothermal vent field in Cluster 12 of the INDEX area. Image corresponds with the data (Image attribution: BGR).

**Figure 179.**
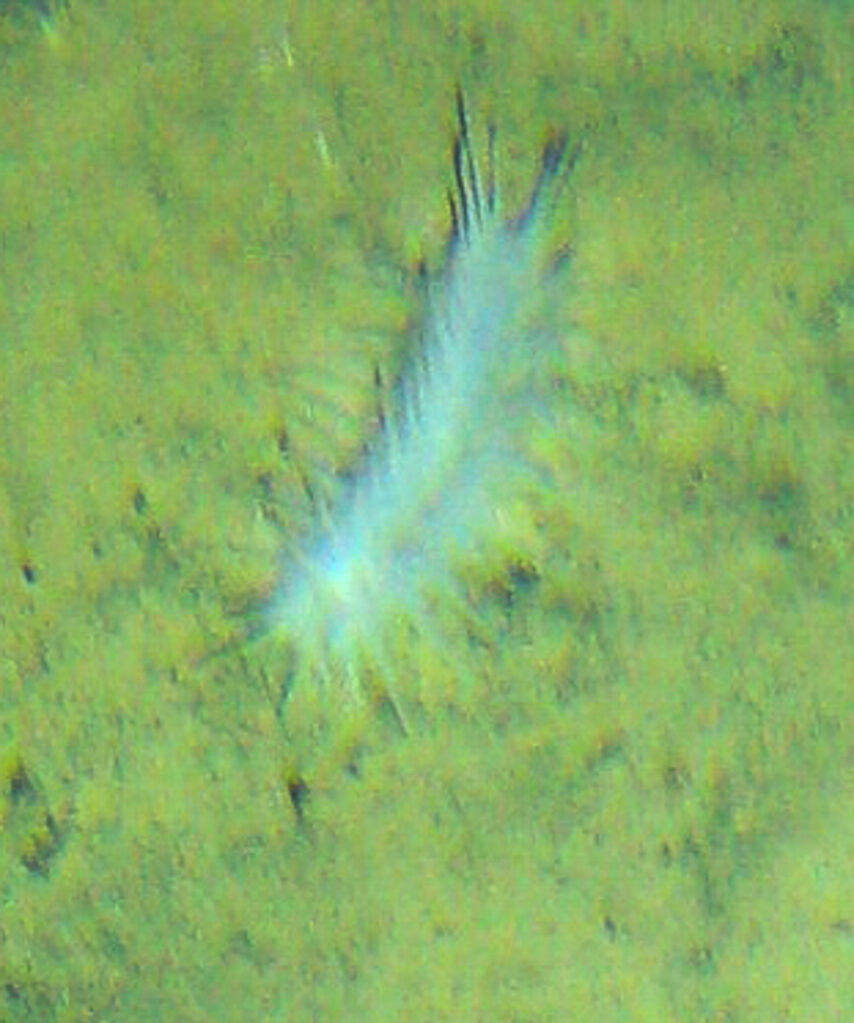
*Oneirophanta* sp. indet. in situ in the MESO area outside the INDEX area. Image corresponds with the data (Image attribution: BGR).

**Figure 180.**
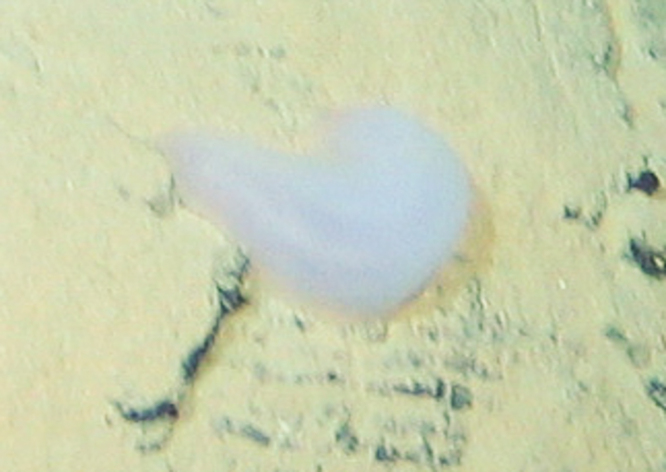
Synallactidae gen. indet. (DZMB_2021_0075) in situ in the MESO area outside the INDEX area. Image corresponds with the data (Image attribution: BGR and GEOMAR).

**Figure 181.**
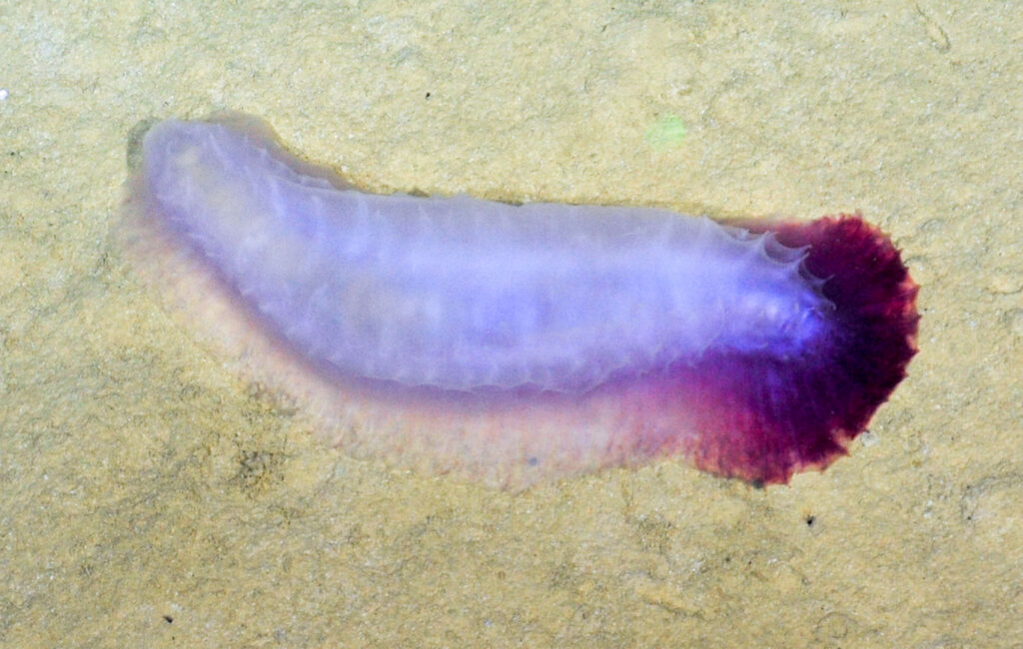
Synallactidae gen. indet. (DZMB_2021_0076) in situ in the Edmond-vent site 2-vent site 7 area in Cluster 4 of the INDEX area. Image corresponds with the data (Image attribution: BGR).

**Figure 182.**
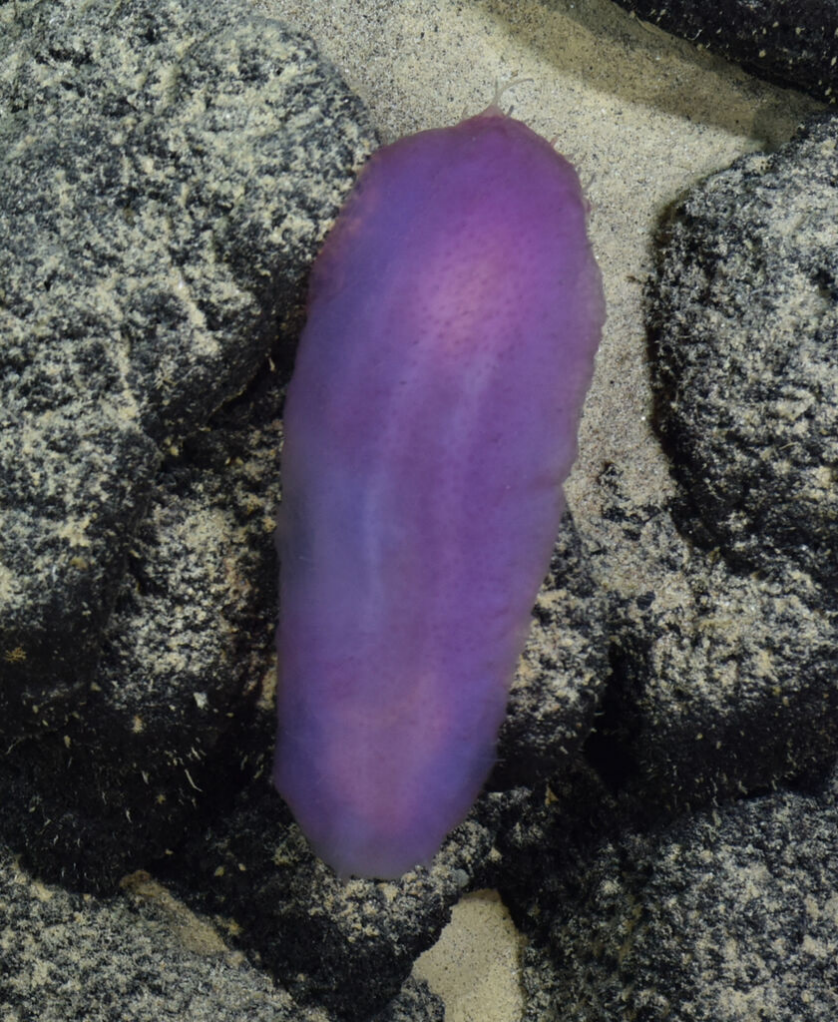
Synallactidae gen. indet. (DZMB_2021_0077) in situ in the surrounding area of the vent site 4 hydrothermal vent field in Cluster 5 of the INDEX area. Image corresponds with the data (Image attribution: BGR).

**Figure 183.**
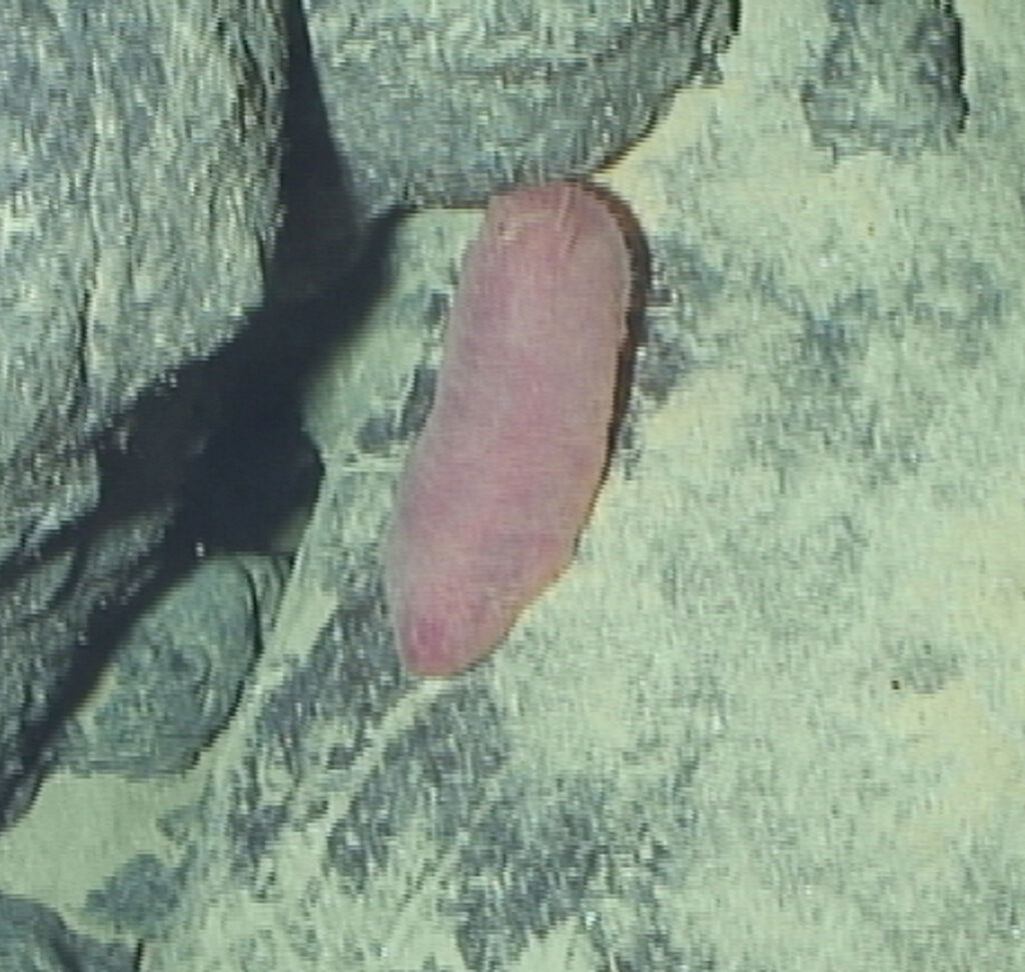
Synallactidae gen. indet. (DZMB_2021_0078) in situ in the surrounding area of the Pelagia hydrothermal vent field in Cluster 8 of the INDEX area. Image corresponds with the data (Image attribution: BGR).

**Figure 184.**
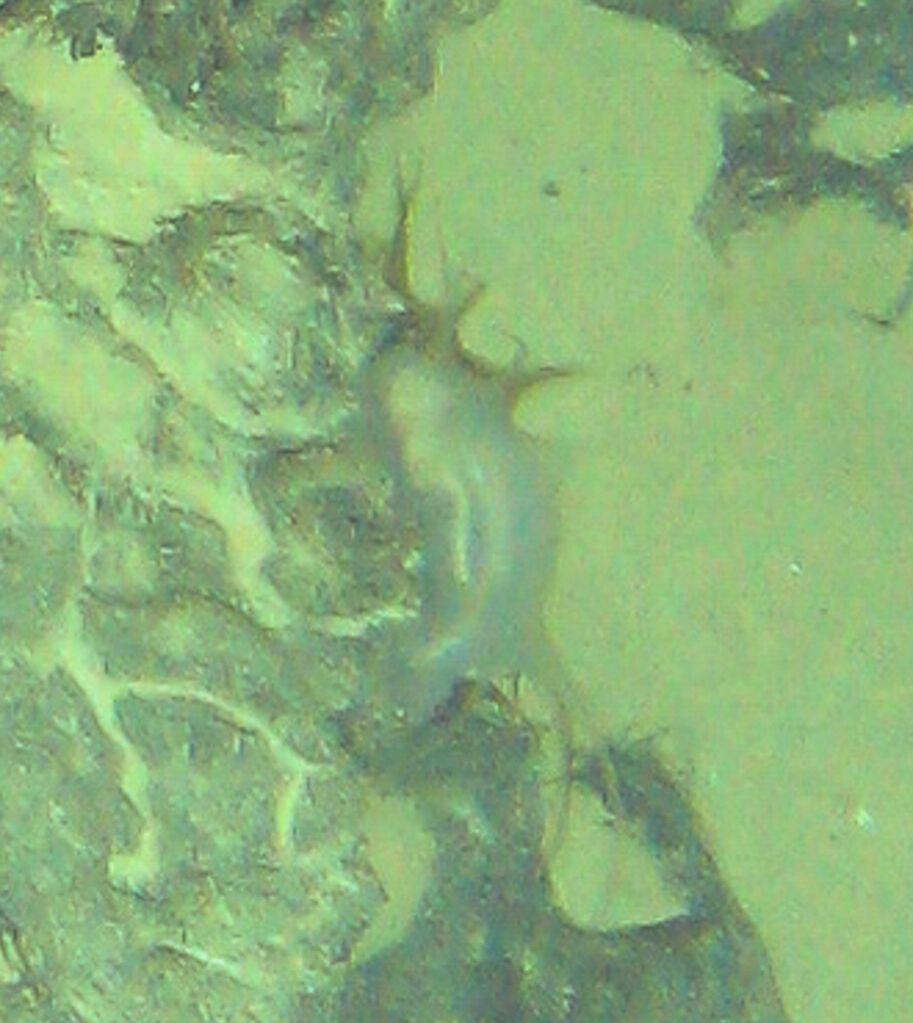
Synallactidae fam. inc. (DZMB_2021_0079) in situ in the MESO area outside the INDEX area. Image corresponds with the data (Image attribution: BGR).

**Figure 185.**
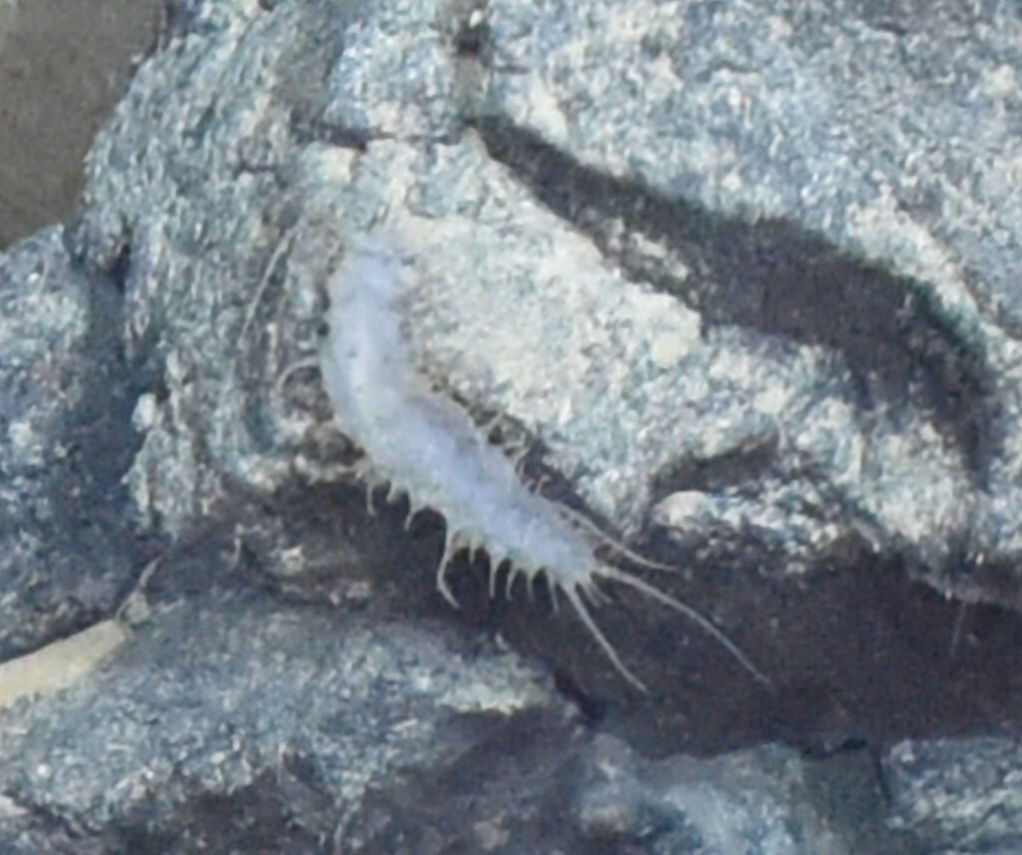
*Synallactes* sp. indet. in situ at the border of the vent site 3 hydrothermal vent field in Cluster 12 of the INDEX area. Image corresponds with the data (Image attribution: BGR).

**Figure 186.**
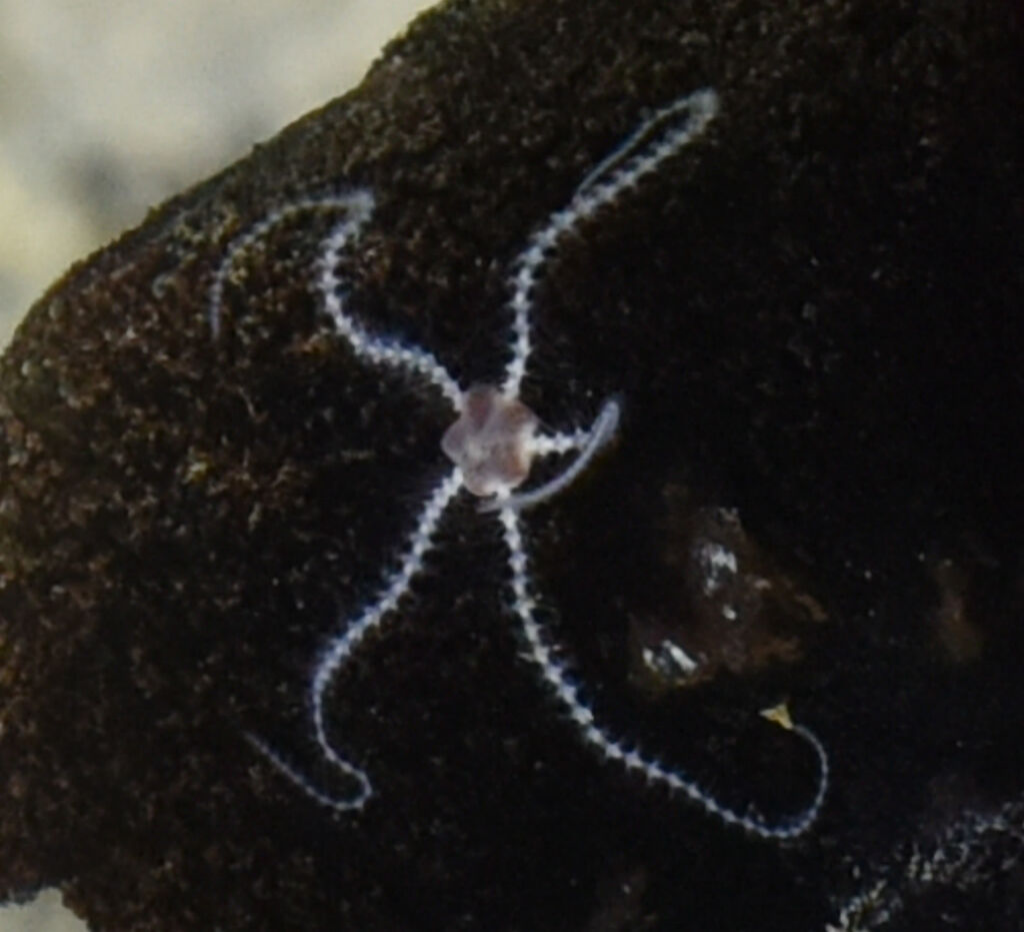
Amphilepidida ord. inc. in situ in the surrounding area of the vent site 4 hydrothermal vent field in Cluster 5 of the INDEX area. Image corresponds with the data (Image attribution: BGR).

**Figure 187.**
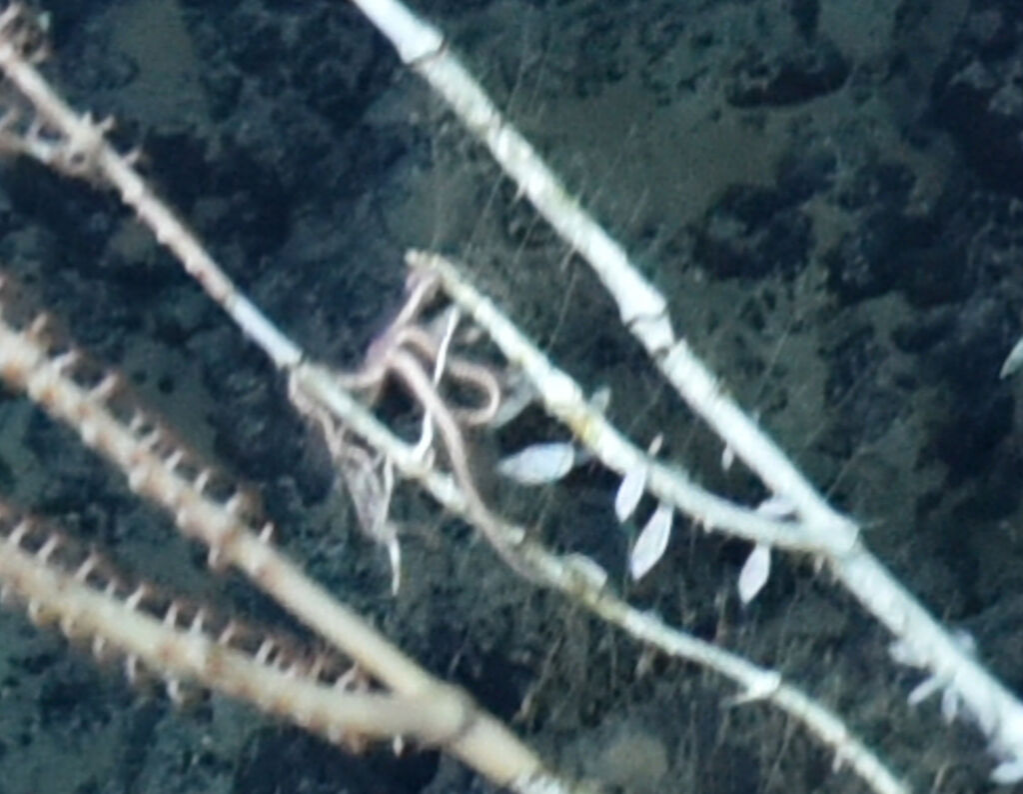
*Asteronyx* gen. inc. in situ at the border of the vent site 6 hydrothermal vent field in Cluster 12 of the INDEX area. Image corresponds with the data (Image attribution: BGR).

**Figure 188.**
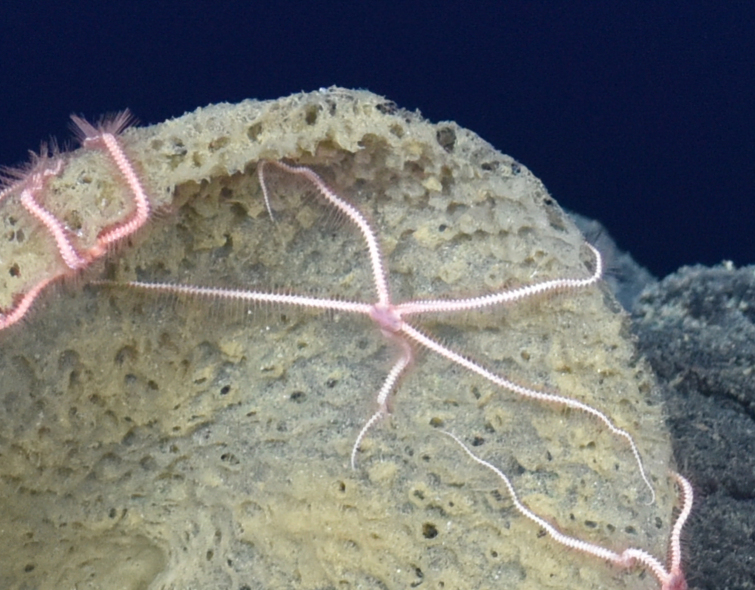
Ophiacanthida ord. inc. in situ in the surrounding area of the vent site 5 hydrothermal vent field in Cluster 11 of the INDEX area. Image corresponds with the data (Image attribution: BGR).

**Figure 189.**
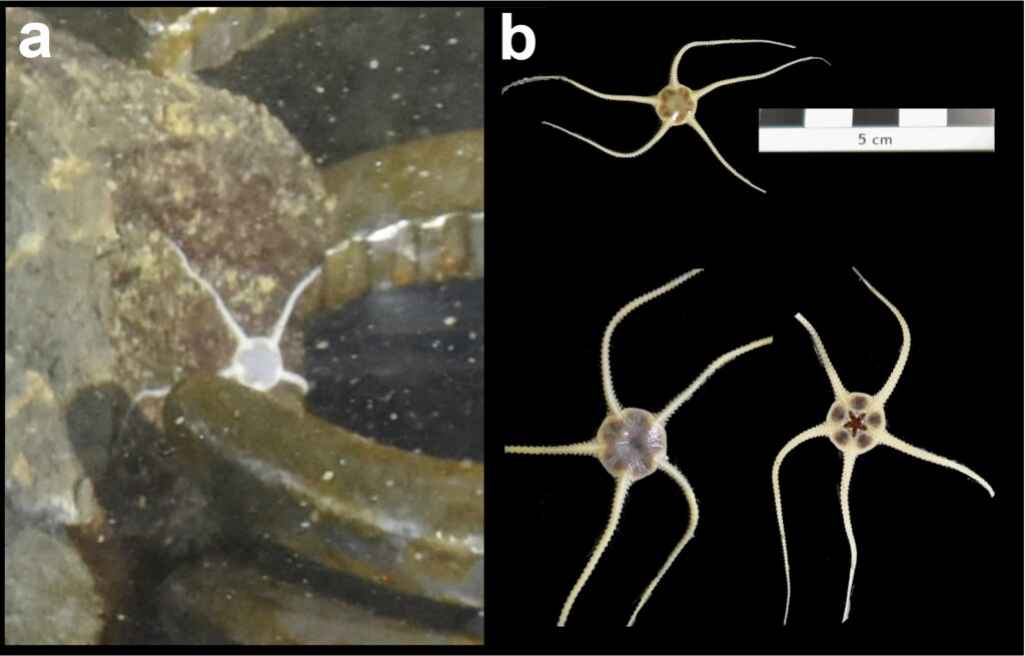
*Ophiophyllumpetilum* sp. inc. in situ (a) and sampled specimen (b) in the surrounding area of the vent site 5 hydrothermal vent field in Cluster 11 of the INDEX area. Image corresponds with the data (Image attribution: BGR).

**Figure 190.**
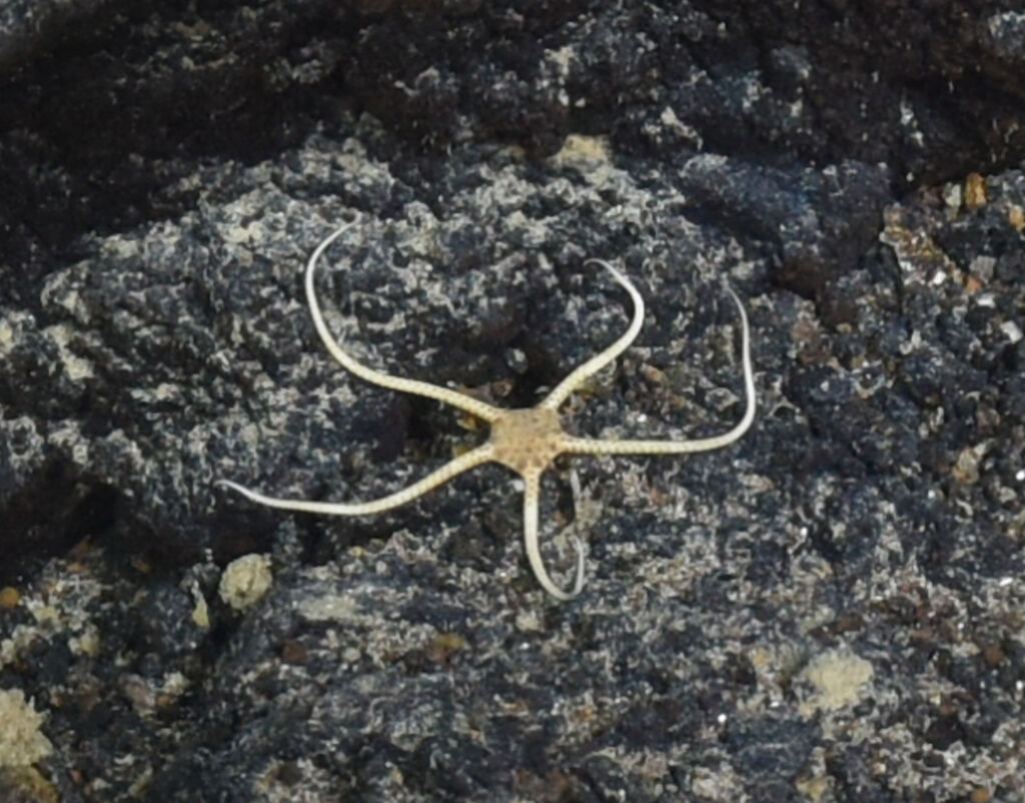
*Ophiosphalma* gen. inc. in situ in the surrounding area of the vent site 6 hydrothermal vent field in Cluster 12 of the INDEX area. Image corresponds with the data (Image attribution: BGR).

**Figure 191.**
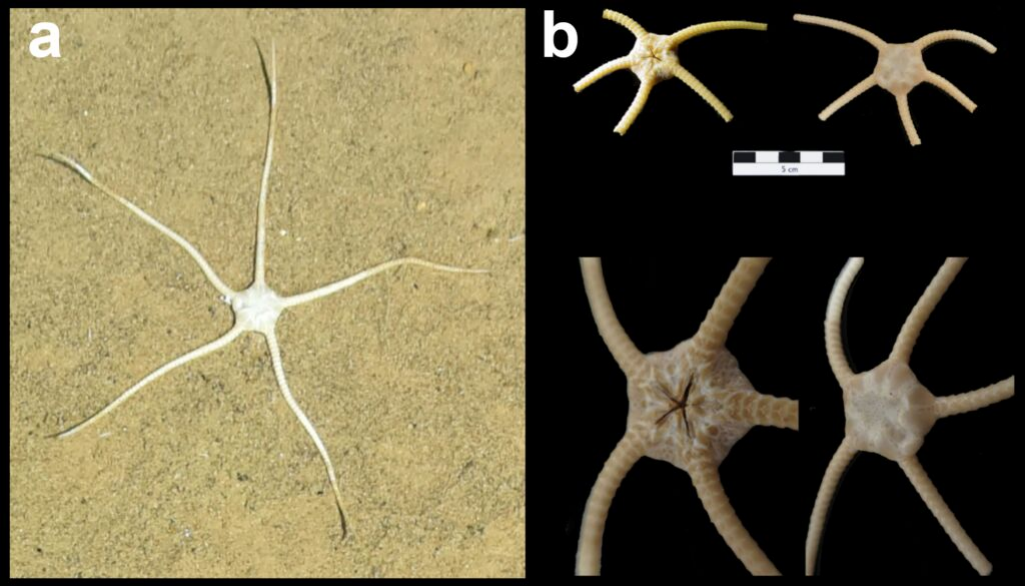
*Ophiosphalmaarmigerum* sp. inc. in situ (a) and sampled specimen (b) at the Rodriguez Triple Junction in Cluster 12 of the INDEX area. Image corresponds with the data (Image attribution: BGR).

**Figure 192.**
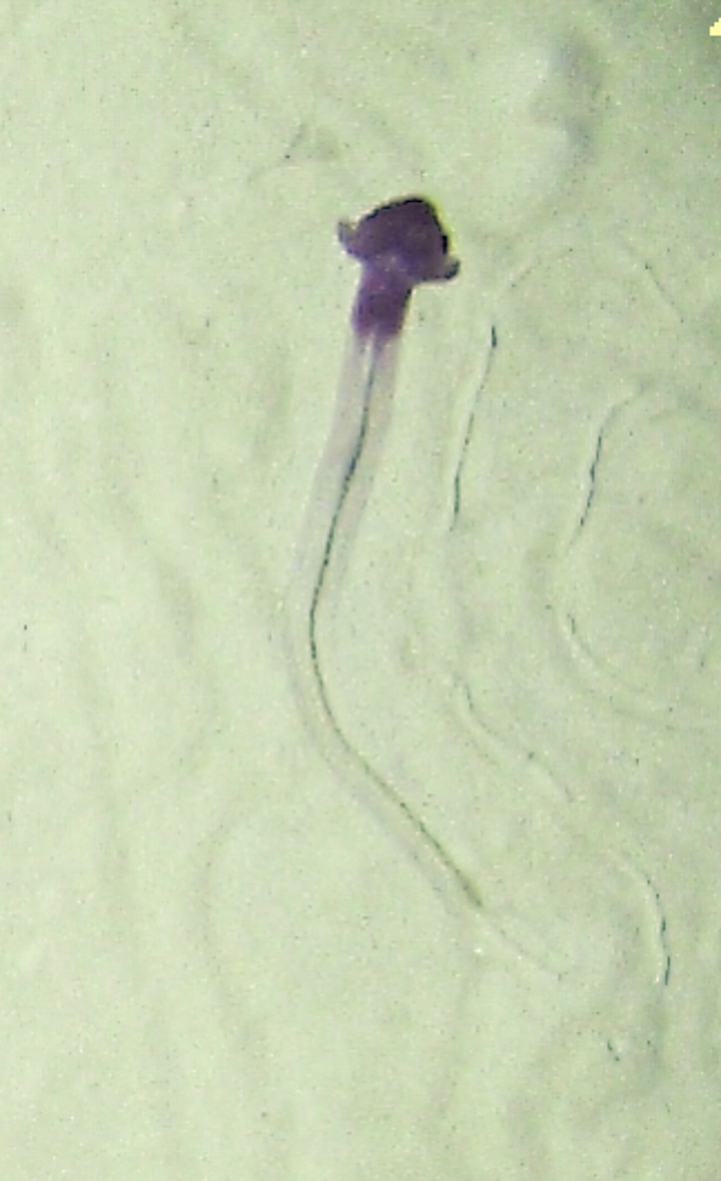
Torquaratoridae fam. inc. in situ at the South East Indian Ridge in Cluster 9 of the INDEX area. Image corresponds with the data (Image attribution: BGR).

**Figure 193.**
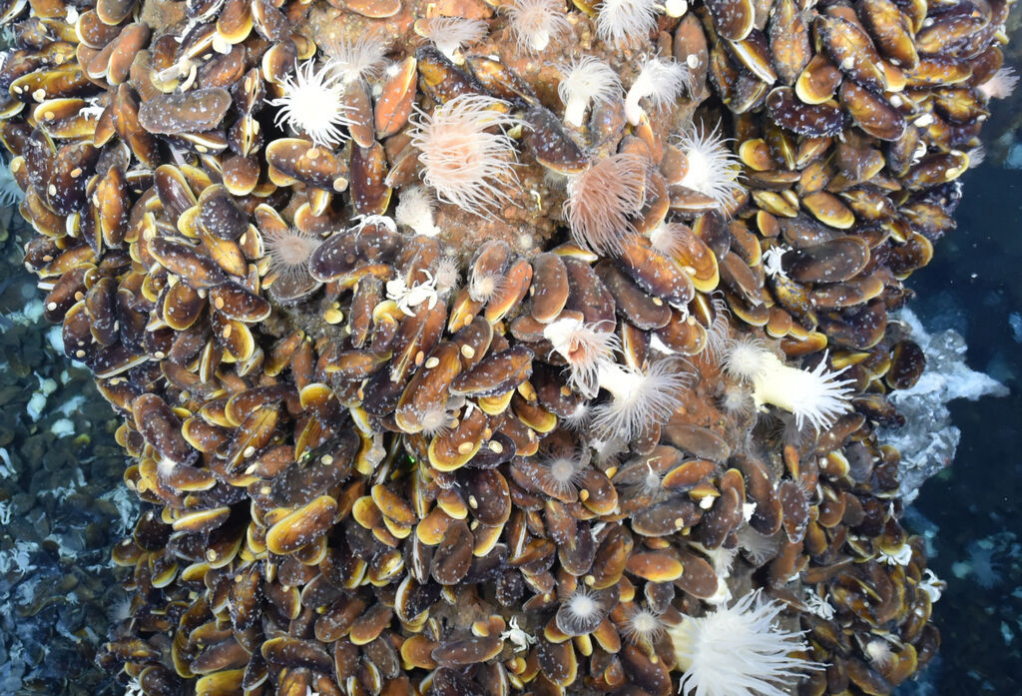
*Bathymodiolusseptemdierum* sp. inc. in situ within the vent site 3 hydrothermal vent field in Cluster 12 of the INDEX area. Image corresponds with the data (Image attribution: BGR and GEOMAR).

**Figure 194.**
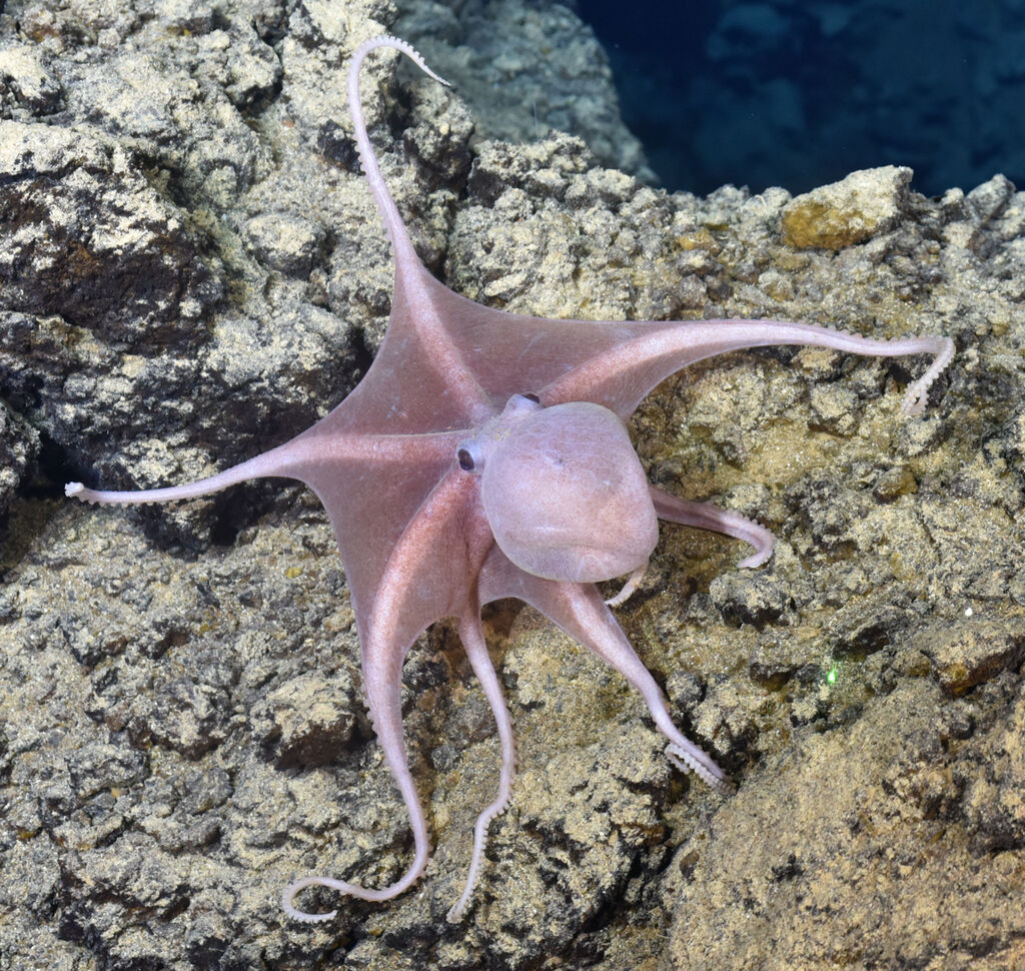
*Bathypolypus* sp. indet. in situ in the surrounding of the vent site 5 hydrothermal vent field in Cluster 11 of the INDEX area. Image corresponds with the data (Image attribution: BGR).

**Figure 195.**
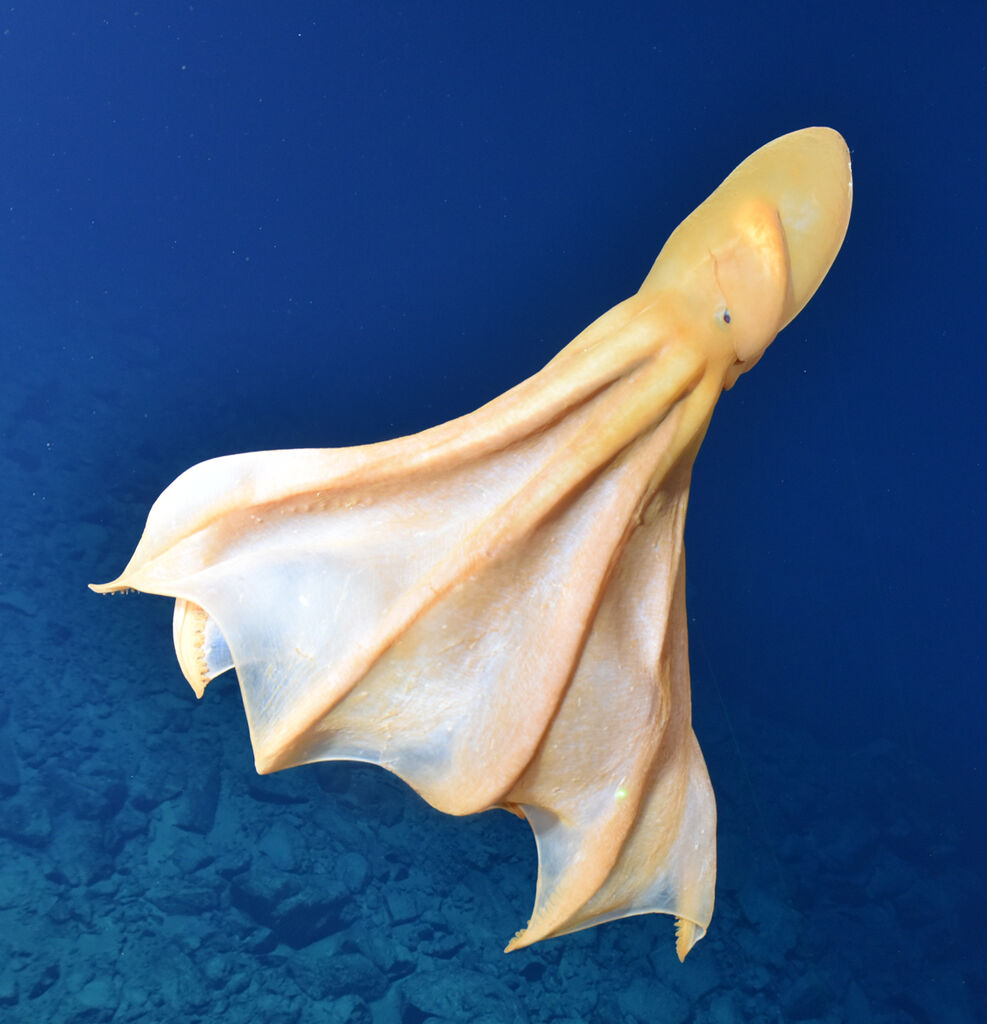
*Cirroteuthis* sp. indet. in situ in the surrounding area of the vent site 4 hydrothermal vent field in Cluster 5 of the INDEX area. Image corresponds with the data (Image attribution: BGR).

**Figure 196.**
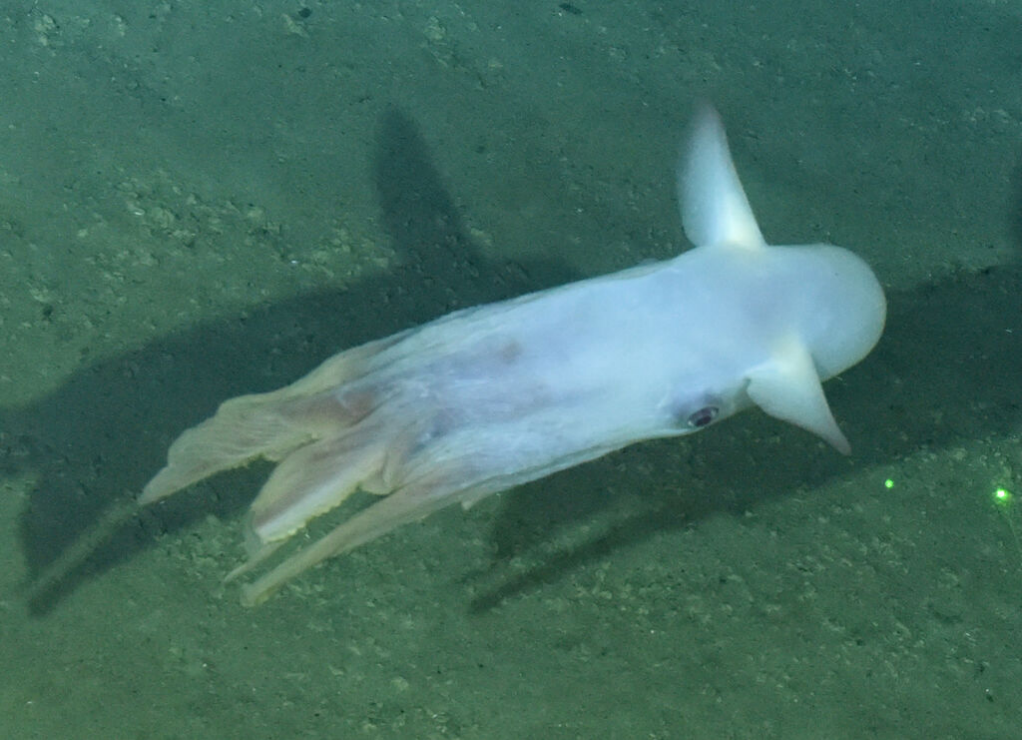
*Grimpoteuthis* gen. inc. in situ at the Rodriguez Triple Junction in Cluster 5 of the INDEX area. Image corresponds with the data (Image attribution: BGR).

**Figure 197.**
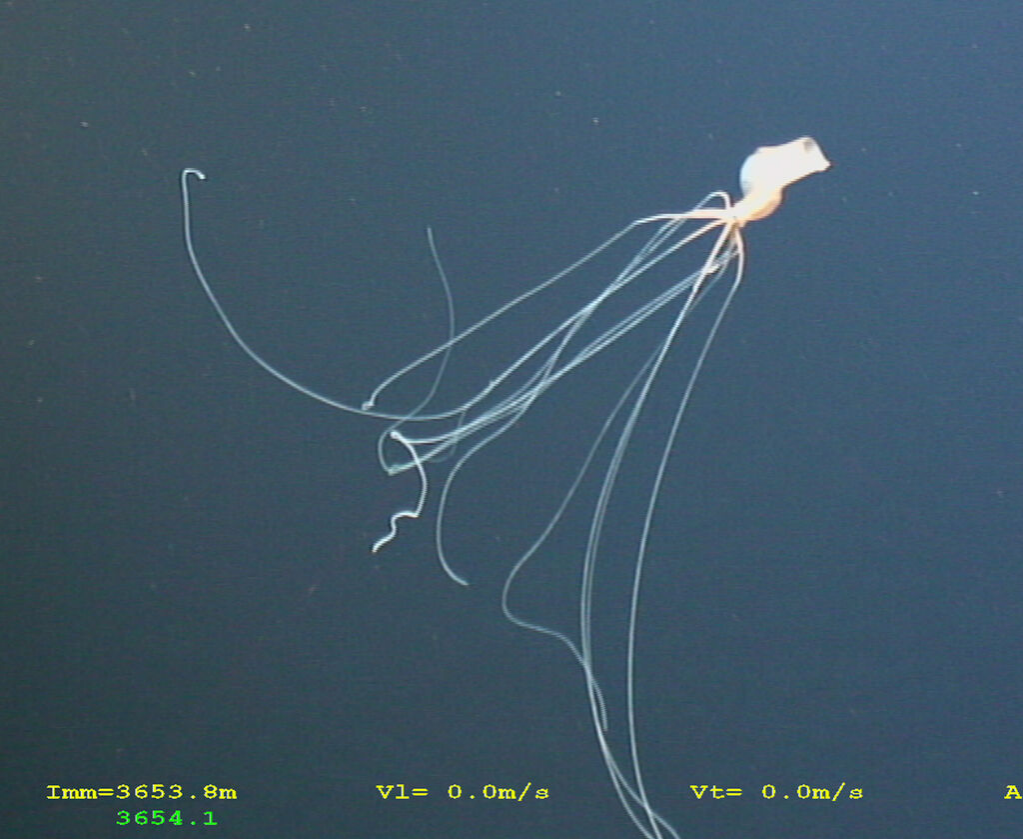
*Magnapinna* sp. indet. in situ at the border of the Pelagia hydrothermal vent field in Cluster 8 of the INDEX area. Image corresponds with the data (Image attribution: BGR).

**Figure 198.**
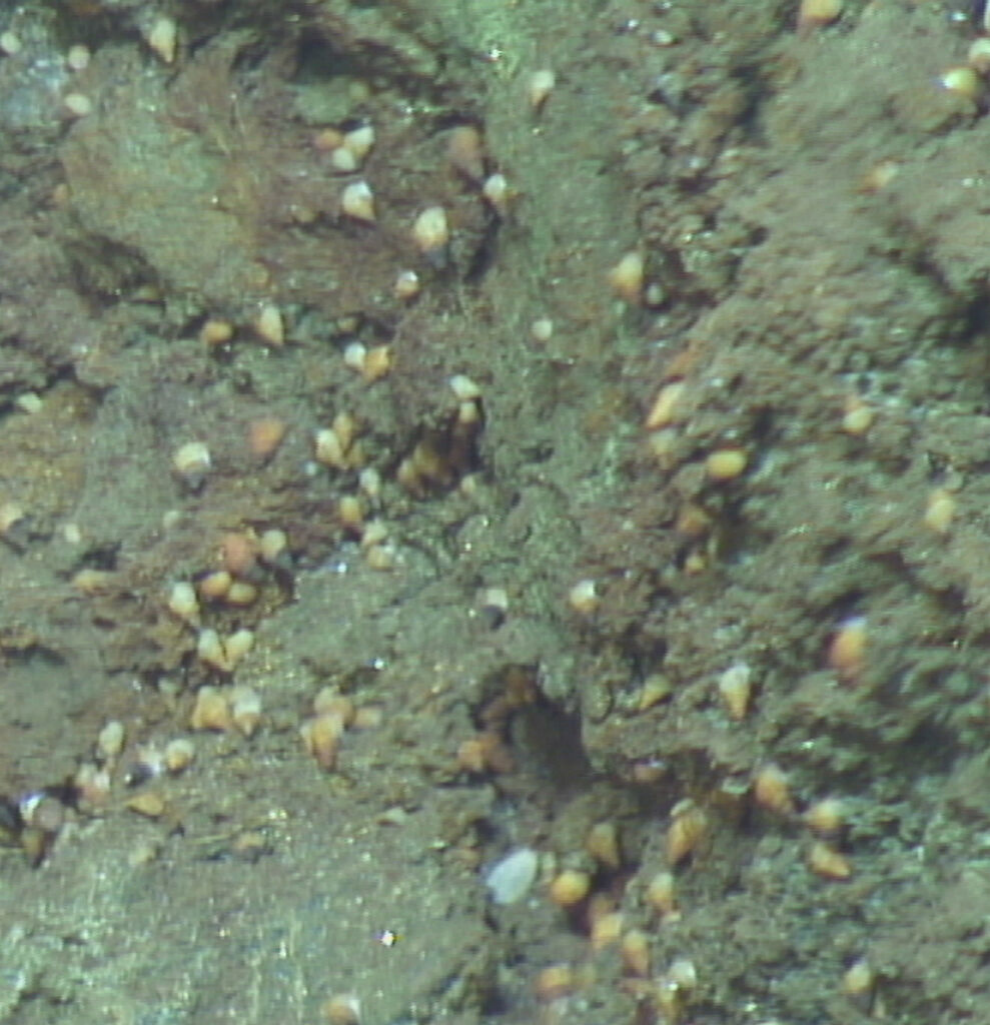
Abyssochrysoidea superfam. inc. in situ within the Pelagia hydrothermal vent field in Cluster 8 of the INDEX area. Image corresponds with the data (Image attribution: BGR).

**Figure 199.**
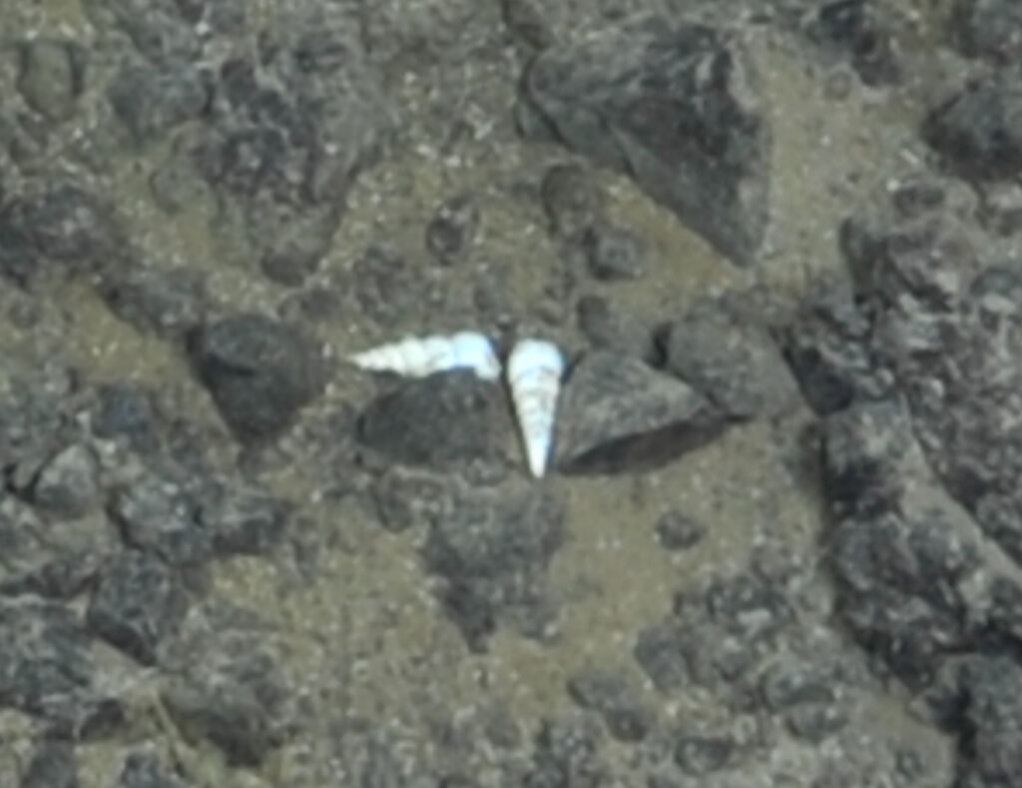
*Speculator* gen. inc. in situ at the border of the vent site 1 hydrothermal vent field in Cluster 4 of the INDEX area. Image corresponds with the data (Image attribution: BGR).

**Figure 200.**
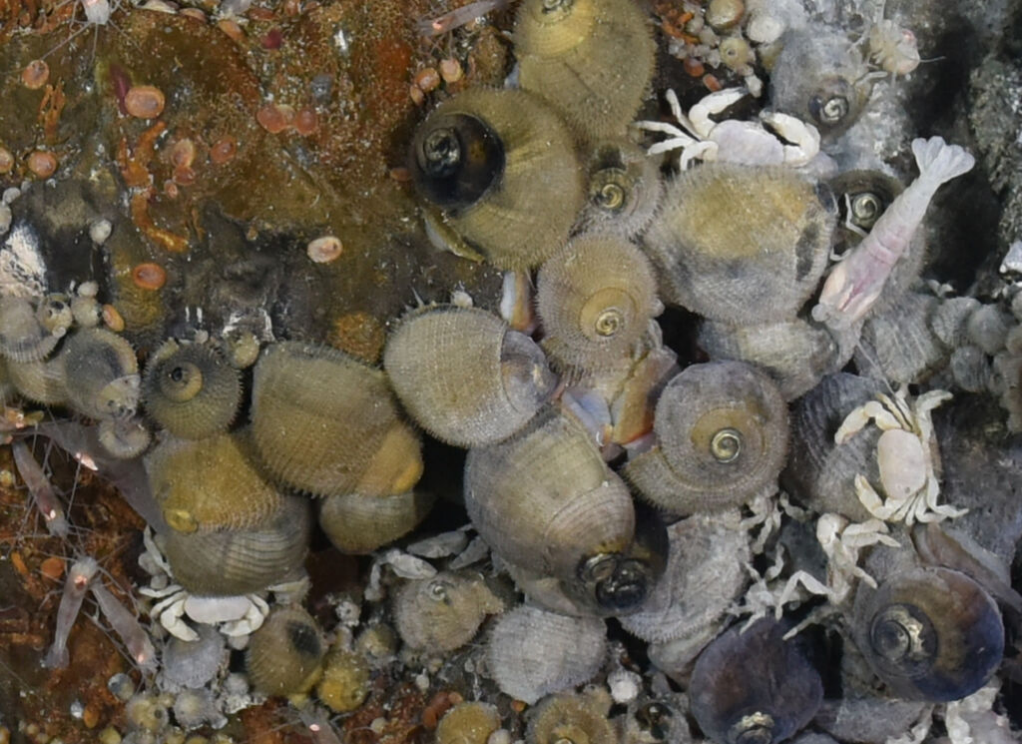
*Alviniconchamarisindica* in situ within the vent site 6 hydrothermal vent field in Cluster 12 of the INDEX area. Image corresponds with the data (Image attribution: BGR).

**Figure 201.**
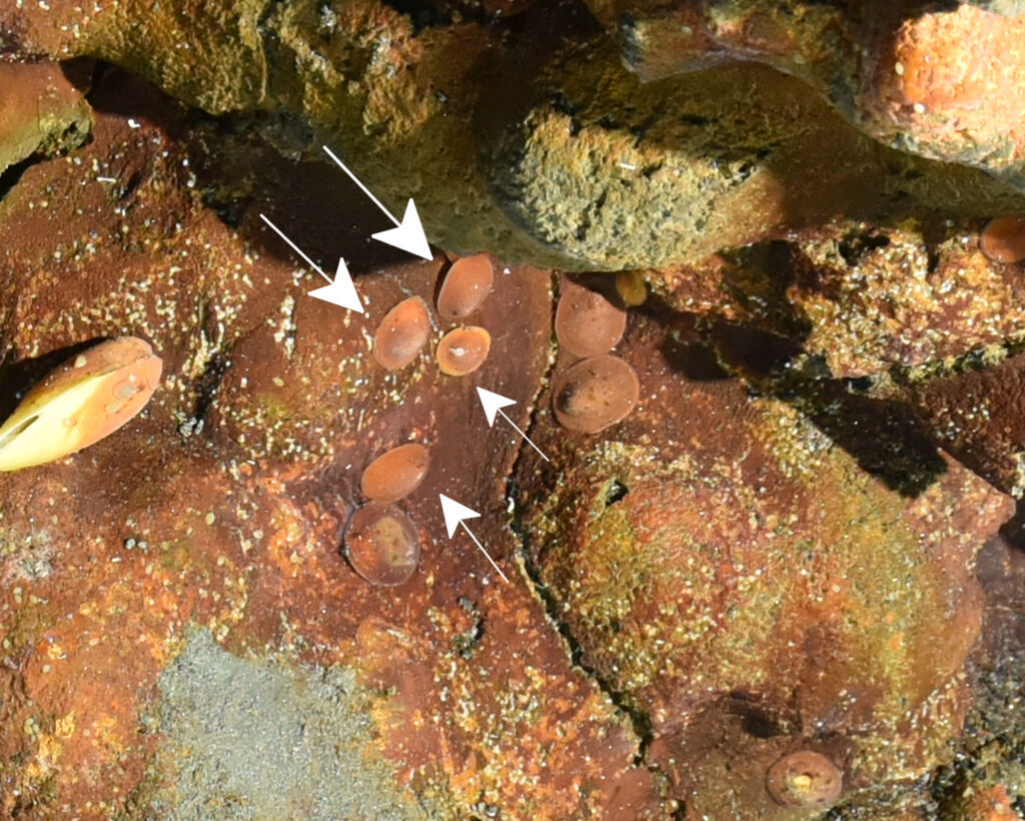
Lepetodrilidae fam. inc. in situ within the vent site 6 hydrothermal vent field in Cluster 12 of the INDEX area. Image corresponds with the data (Image attribution: BGR).

**Figure 202.**
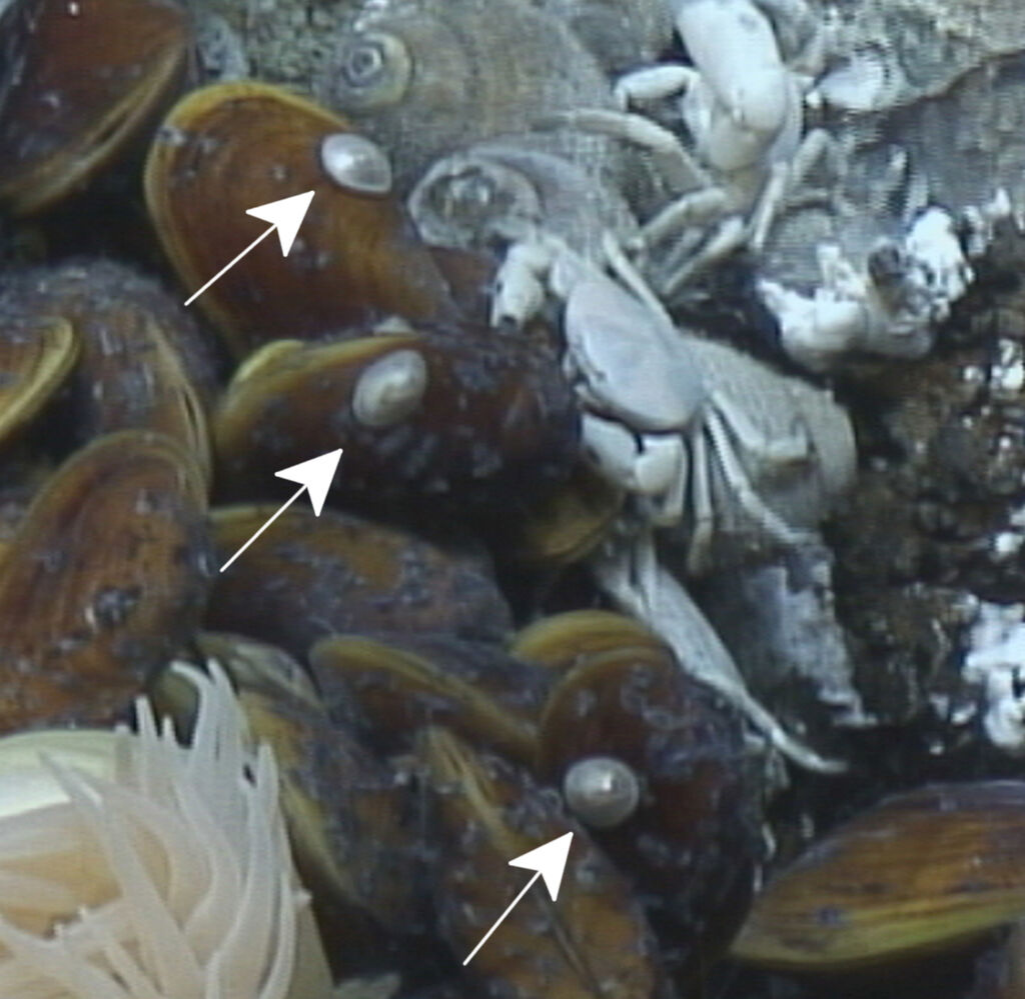
*Lepetodrilus* gen. inc. in situ within the Pelagia hydrothermal vent field in Cluster 8 of the INDEX area. Image corresponds with the data (Image attribution: BGR).

**Figure 203.**
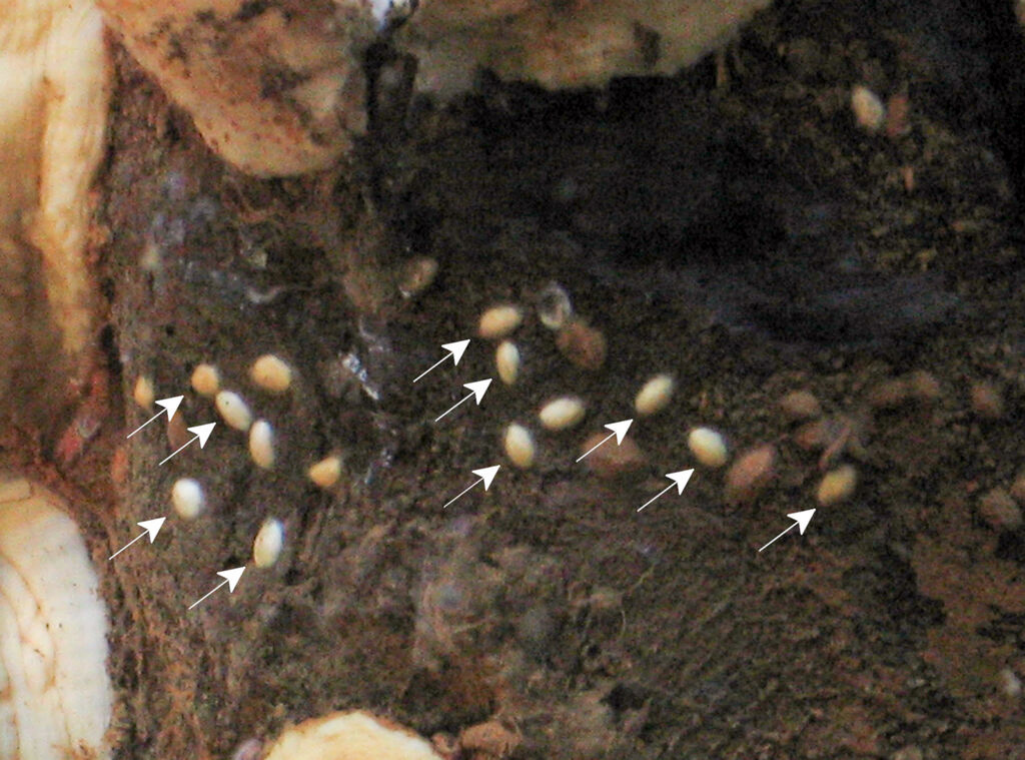
Lepetodrilidae *Lepetodrilus* sp. indet. in situ within the Kairei hydrothermal vent field in Cluster 5 of the INDEX area. Image corresponds with the data (Image attribution: BGR).

**Figure 204.**
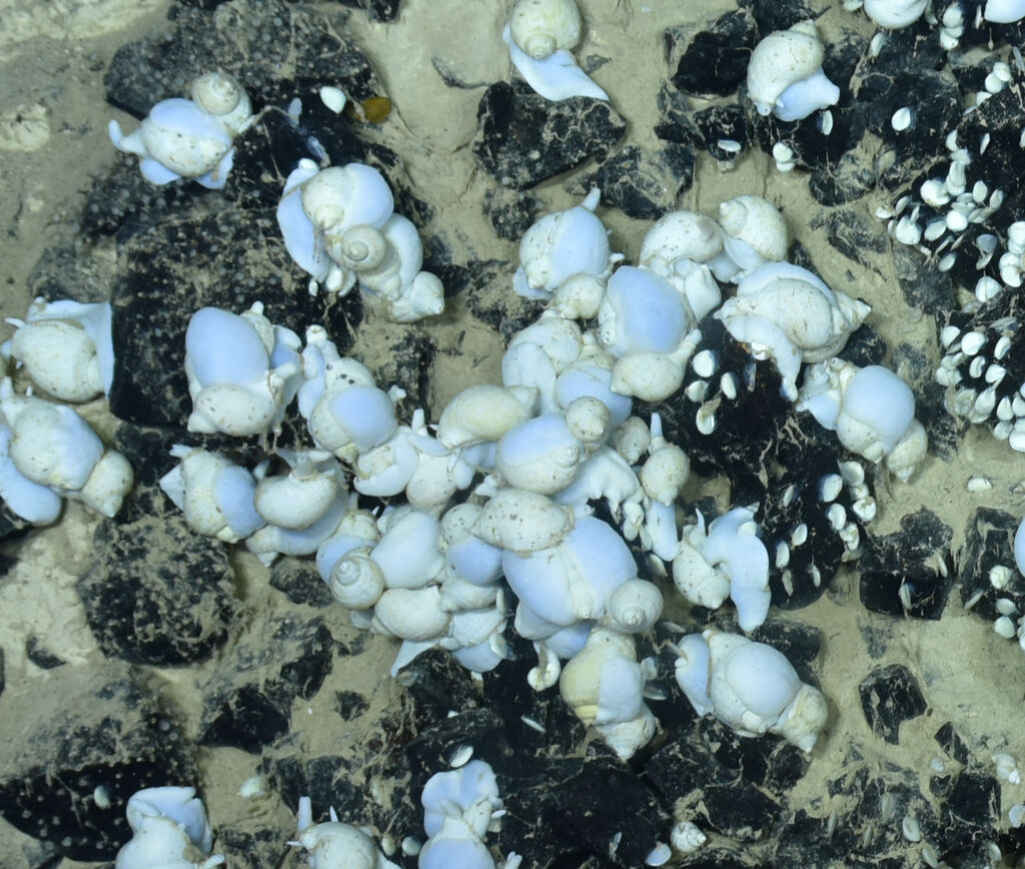
*Phymorhynchus* sp. indet. in situ within the vent site 3 hydrothermal vent field in Cluster 12 of the INDEX area. Image corresponds with the data (Image attribution: BGR).

**Figure 205.**
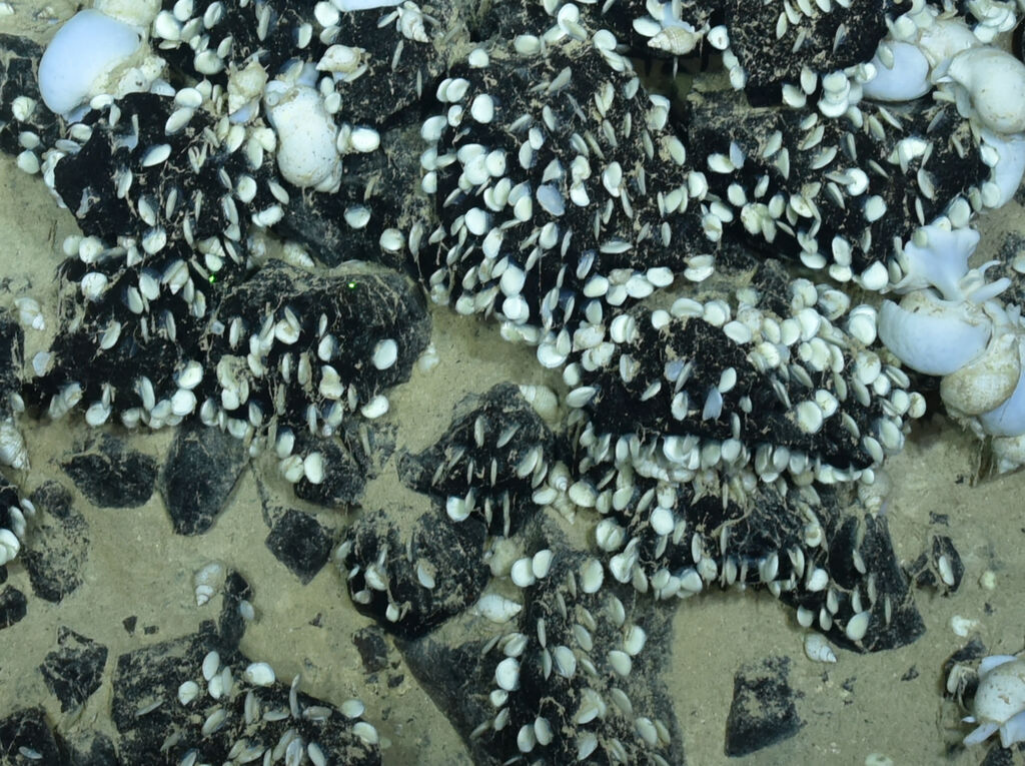
*Phymorhynchus* sp. indet. (Egg capsules) in situ at the border of the vent site 3 hydrothermal vent field in Cluster 12 of the INDEX area. Image corresponds with the data (Image attribution: BGR).

**Figure 206.**
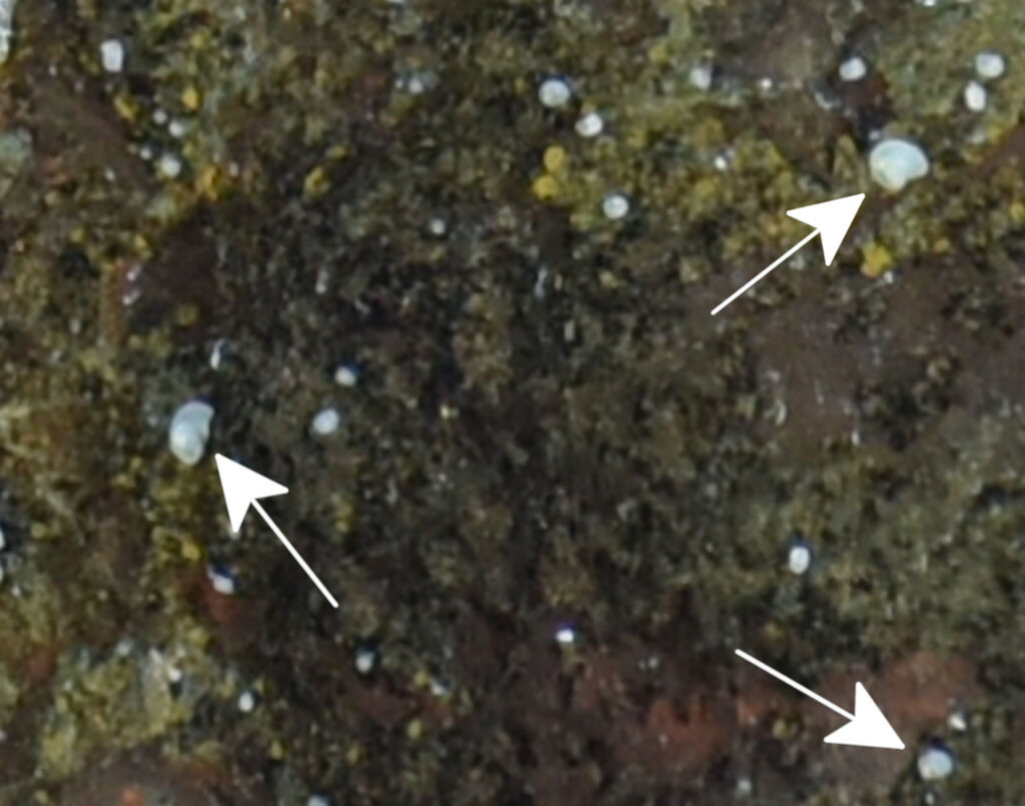
Melanodrymiidae fam. inc. in situ within the vent site 4 hydrothermal vent field in Cluster 5 of the INDEX area. Image corresponds with the data (Image attribution: BGR).

**Figure 207.**
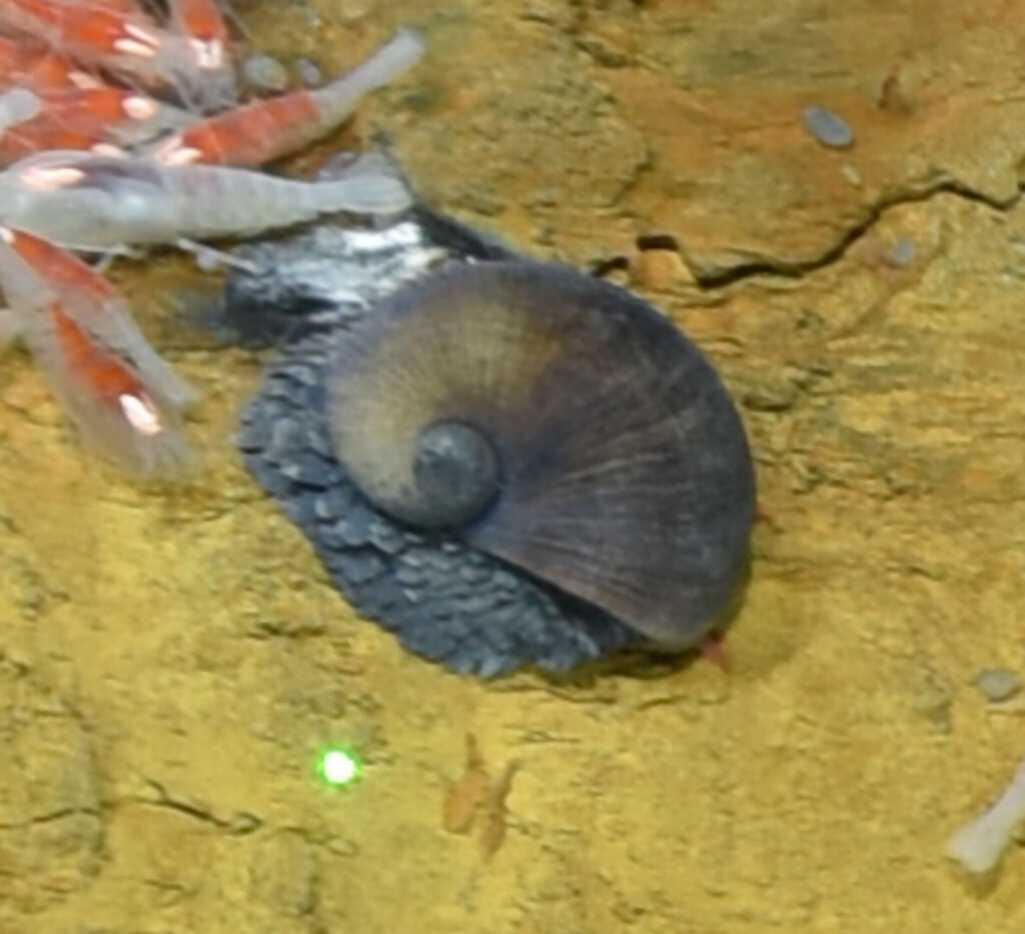
*Chrysomallonsquamiferum* in situ within the vent site 6 hydrothermal vent field in Cluster 12 of the INDEX area. Image corresponds with the data (Image attribution: BGR).

**Figure 208.**
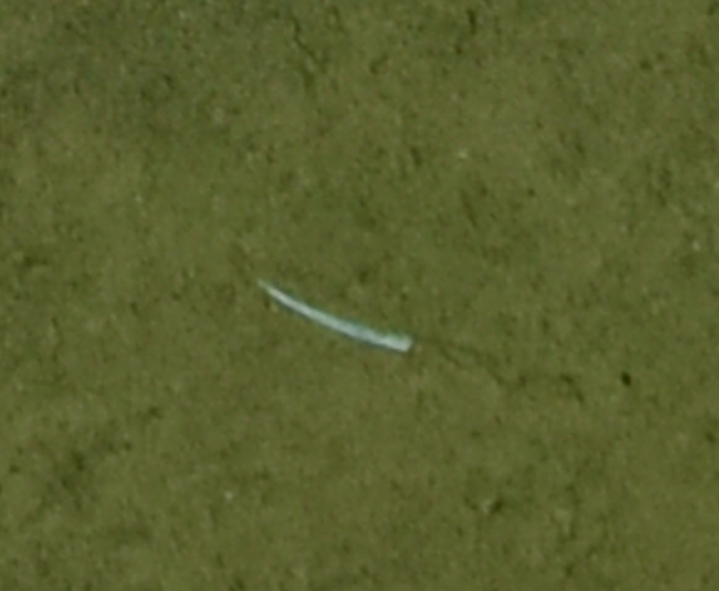
Scaphopoda ord. indet. in situ in the surrounding area of the vent site 6 hydrothermal vent field in Cluster 12 of the INDEX area. Image corresponds with the data (Image attribution: BGR).

**Figure 209.**
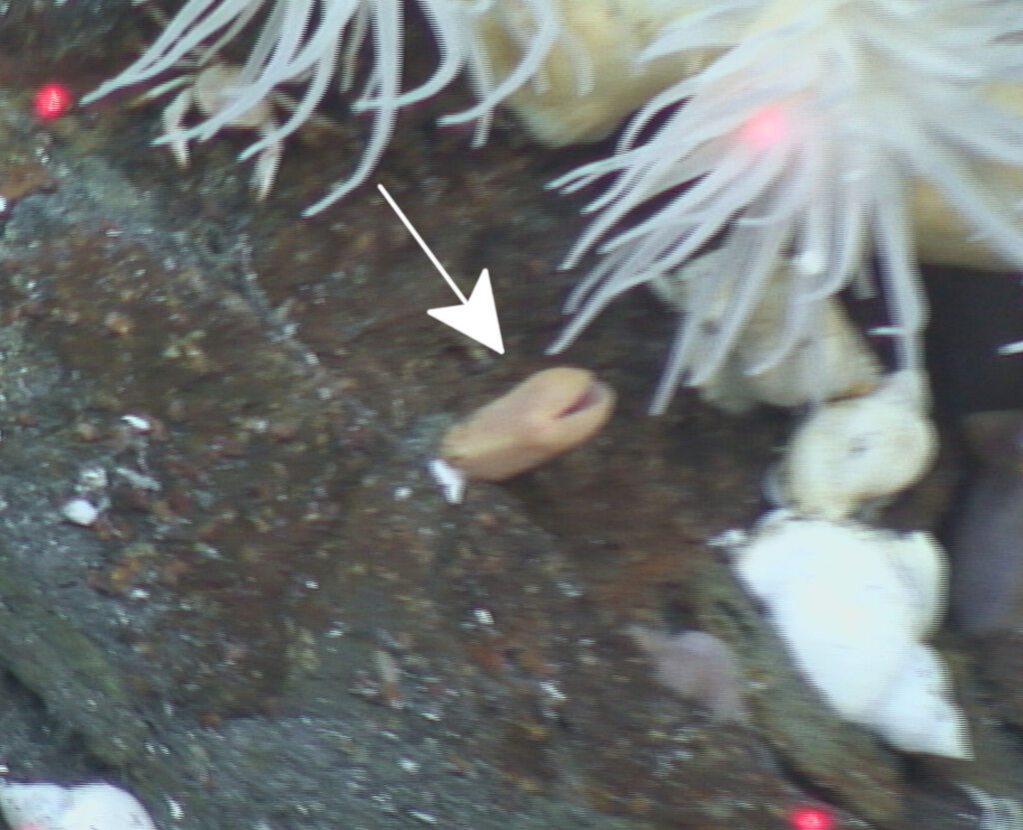
Solenogastres ord. indet. in situ within the Pelagia hydrothermal vent field in Cluster 8 of the INDEX area. Image corresponds with the data (Image attribution: BGR).

**Figure 210.**
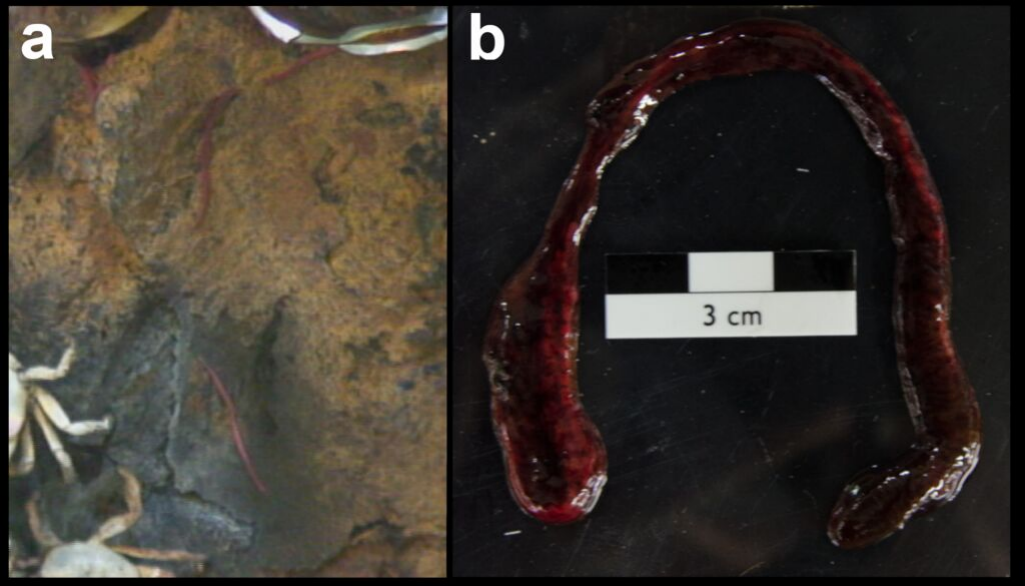
*Thermanemertes* gen. inc. in situ (a) and sampled specimen (b) within the Kairei hydrothermal vent field in Cluster 5 of the INDEX area. Image corresponds with the data (Image attribution: BGR).

**Figure 211.**
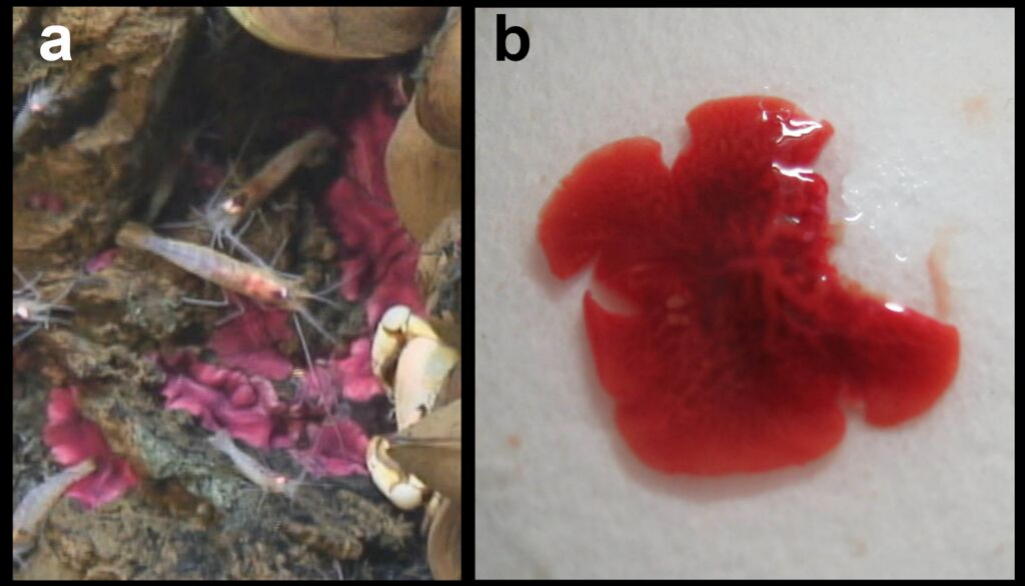
Polycladida fam. indet. in situ (a) and sampled specimen (b) within the Kairei hydrothermal vent field in Cluster 5 of the INDEX area. Image corresponds with the data (Image attribution: BGR).

**Figure 212.**
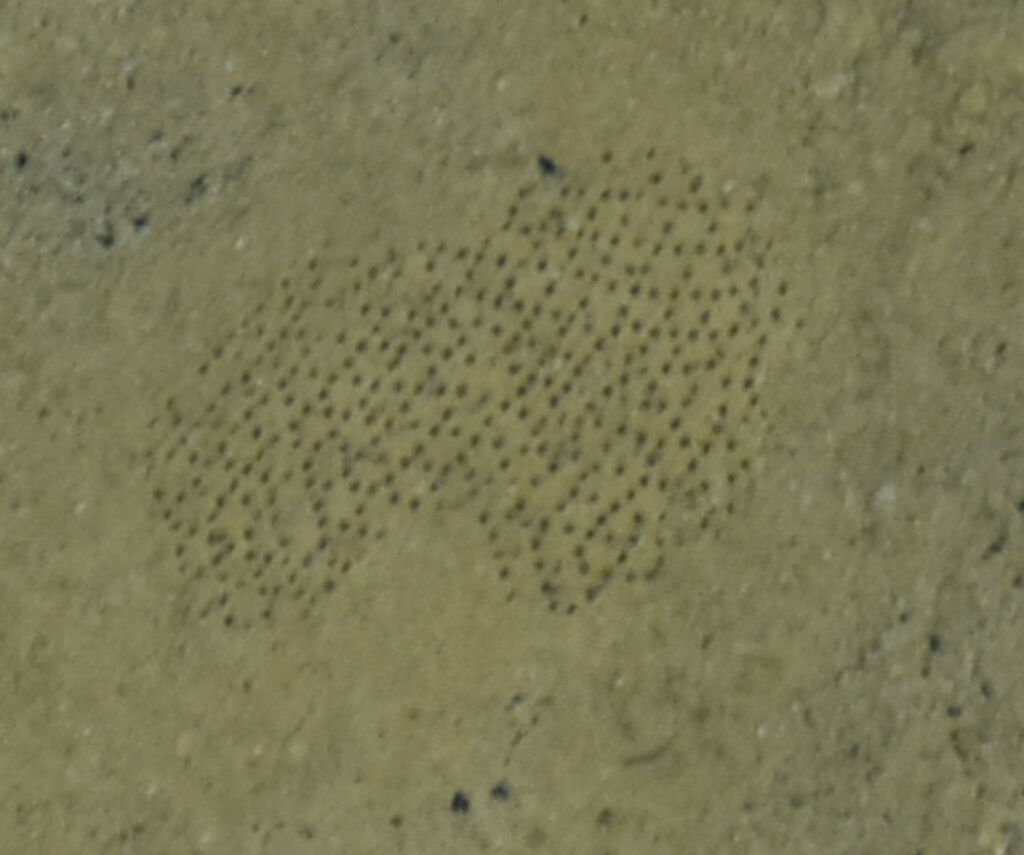
*Paleodictyonnodosum* sp. inc. in situ in the surrounding area of the vent site 5 hydrothermal vent field in Cluster 11 of the INDEX area. Image corresponds with the data (Image attribution: BGR).

**Figure 213.**
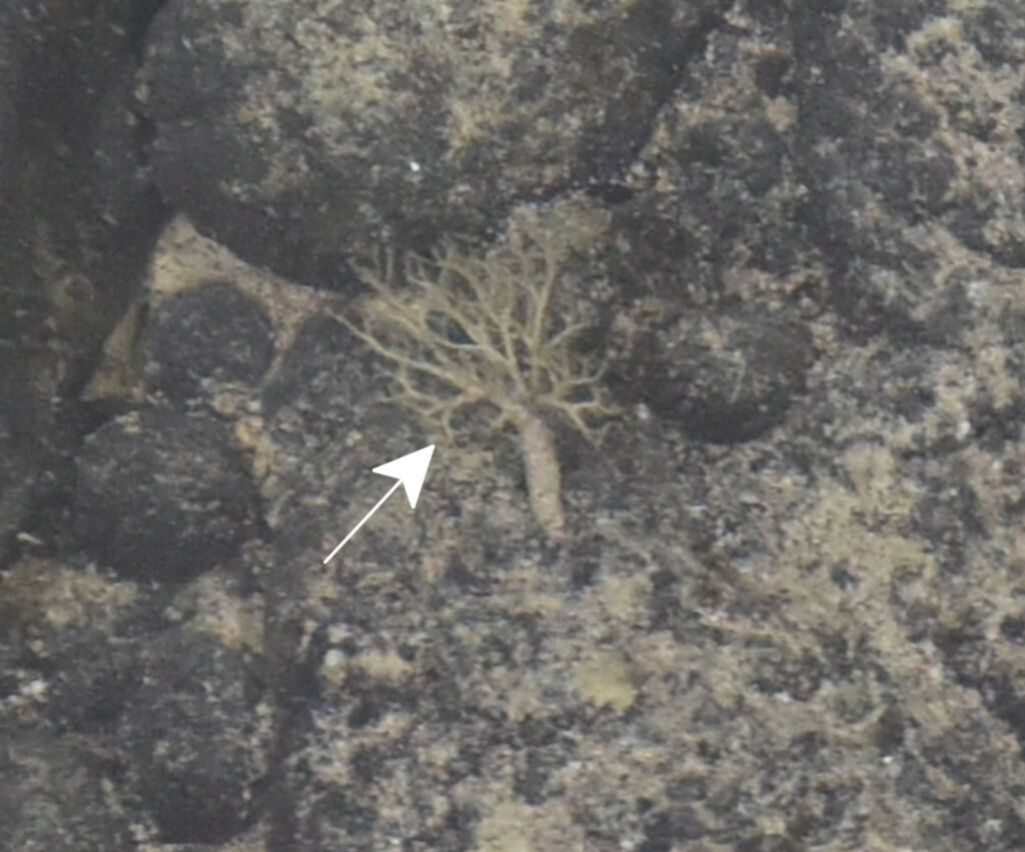
Monothalamea ord. indet. (DZMB_2021_0080) in situ in the surrounding area of the vent site 4 hydrothermal vent field in Cluster 5 of the INDEX area. Image corresponds with the data (Image attribution: BGR).

**Figure 214.**
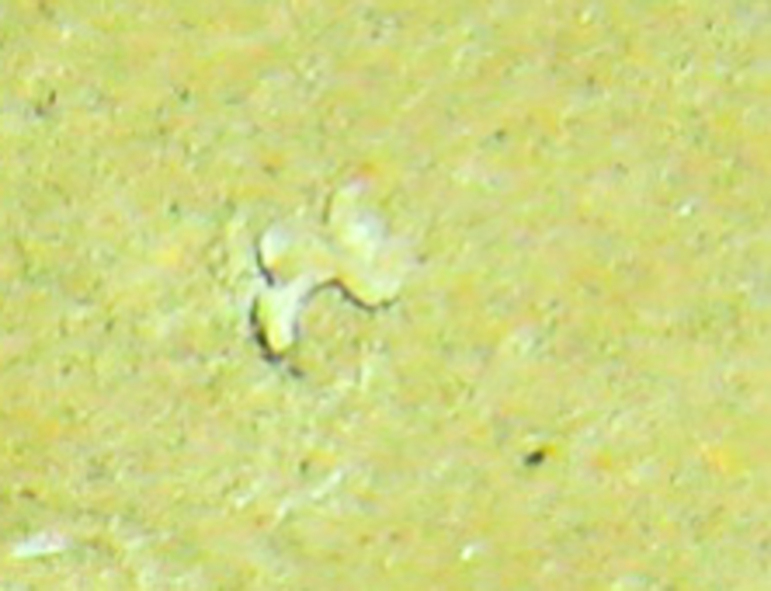
Monothalamea ord. indet. (DZMB_2021_0081) in situ in the area surrounding the Edmond hydrothermal vent field in Cluster 4 of the INDEX area. Image corresponds with the data (Image attribution: BGR).

**Figure 215.**
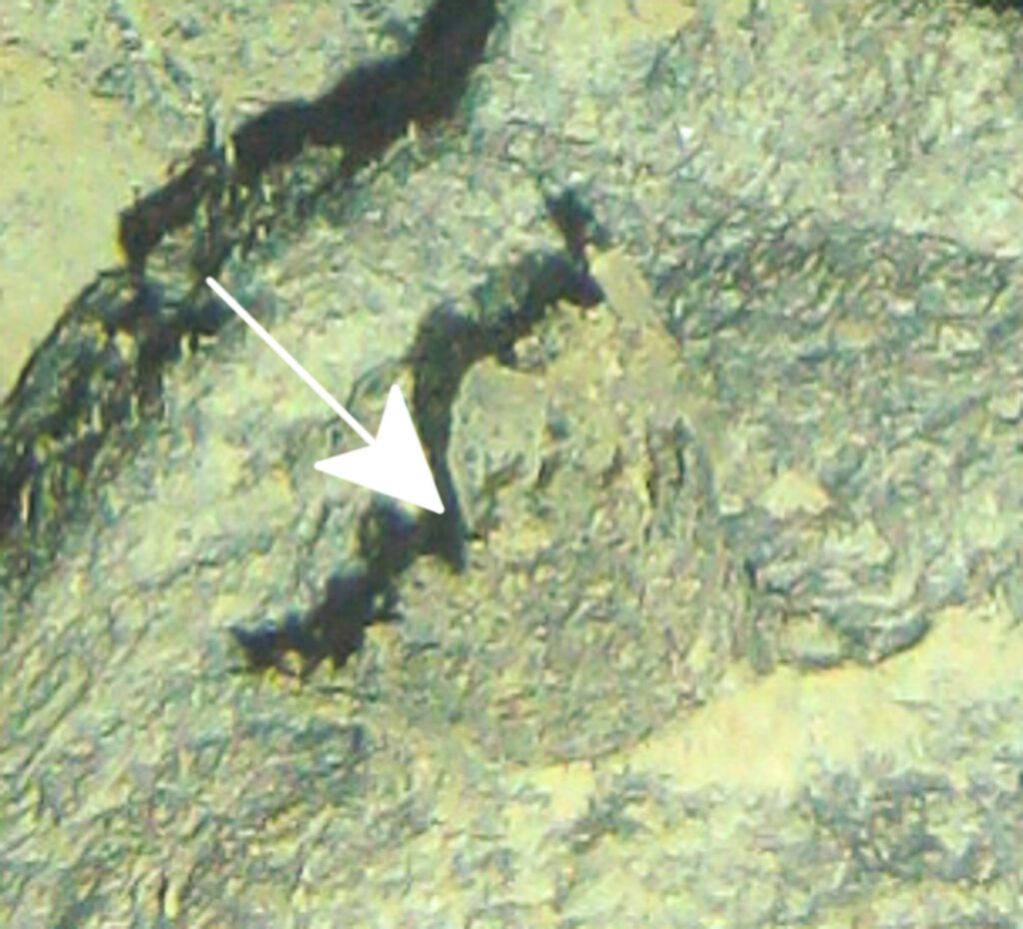
Monothalamea ord. indet. (DZMB_2021_0082) in situ in the MESO area outside the INDEX area. Image corresponds with the data (Image attribution: BGR).

**Figure 216.**
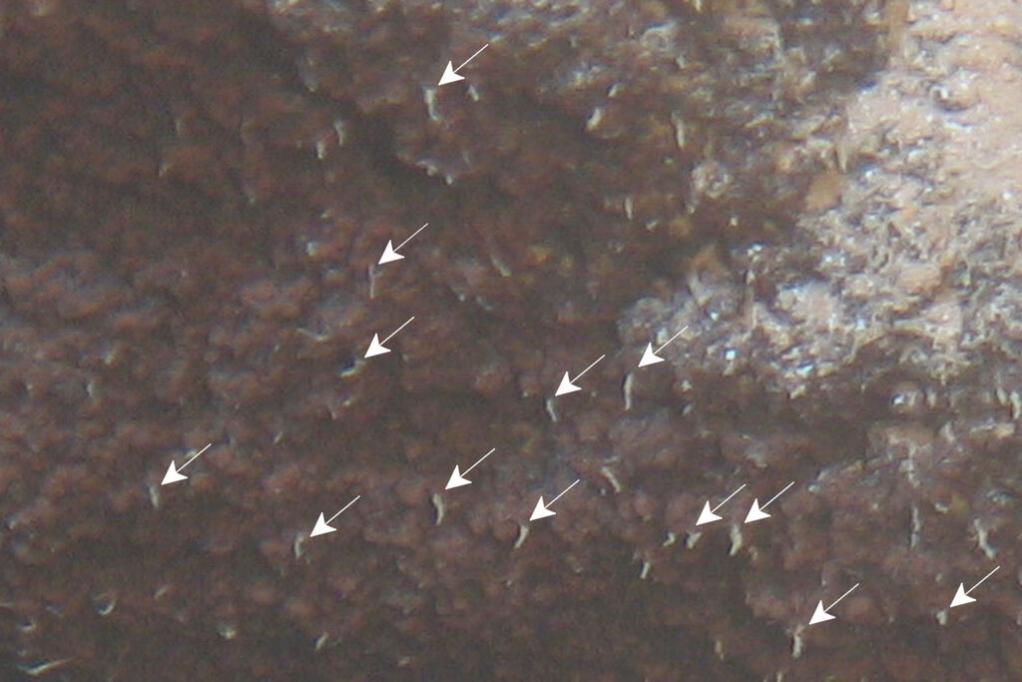
*Luffammina* gen. inc. in situ in the Edmond-vent site 2-vent site 7 area in Cluster 4 of the INDEX area. Image corresponds with the data (Image attribution: BGR and GEOMAR).

**Figure 217.**
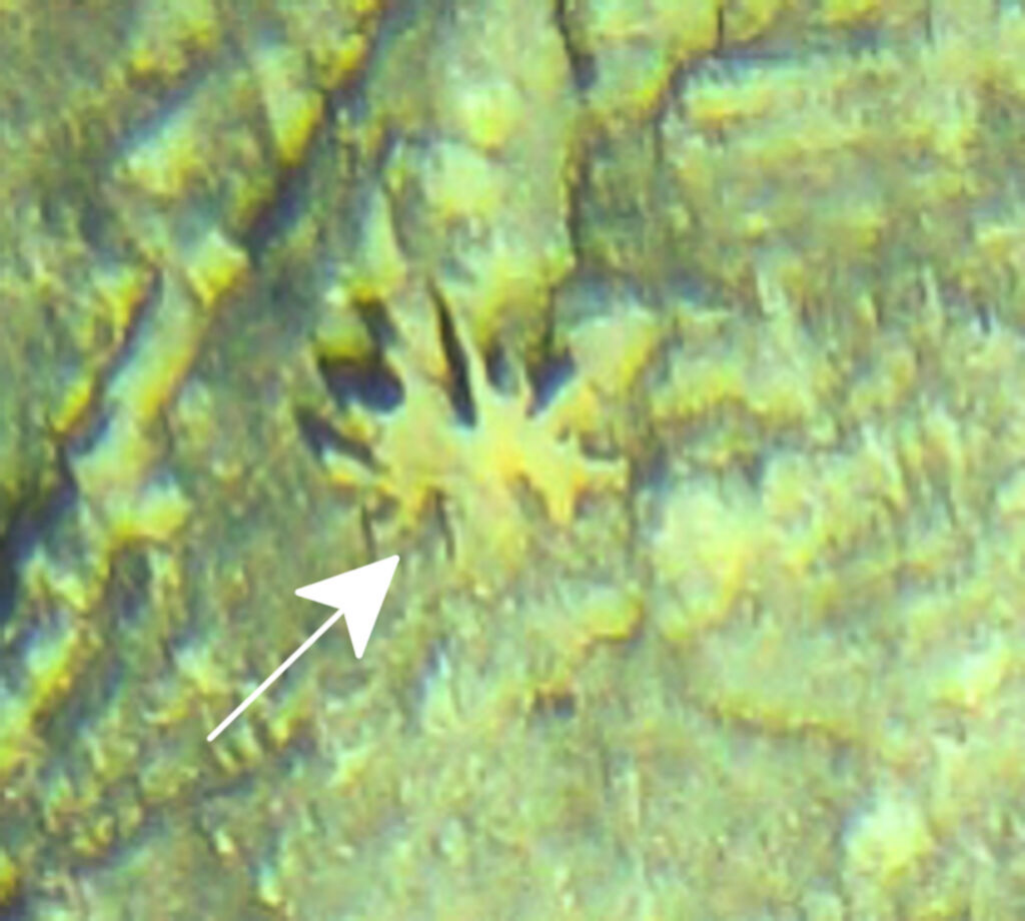
*Psammina* gen. inc. (DZMB_2021_0083) in situ in the MESO area outside the INDEX area. Image corresponds with the data (Image attribution: BGR).

**Figure 218.**
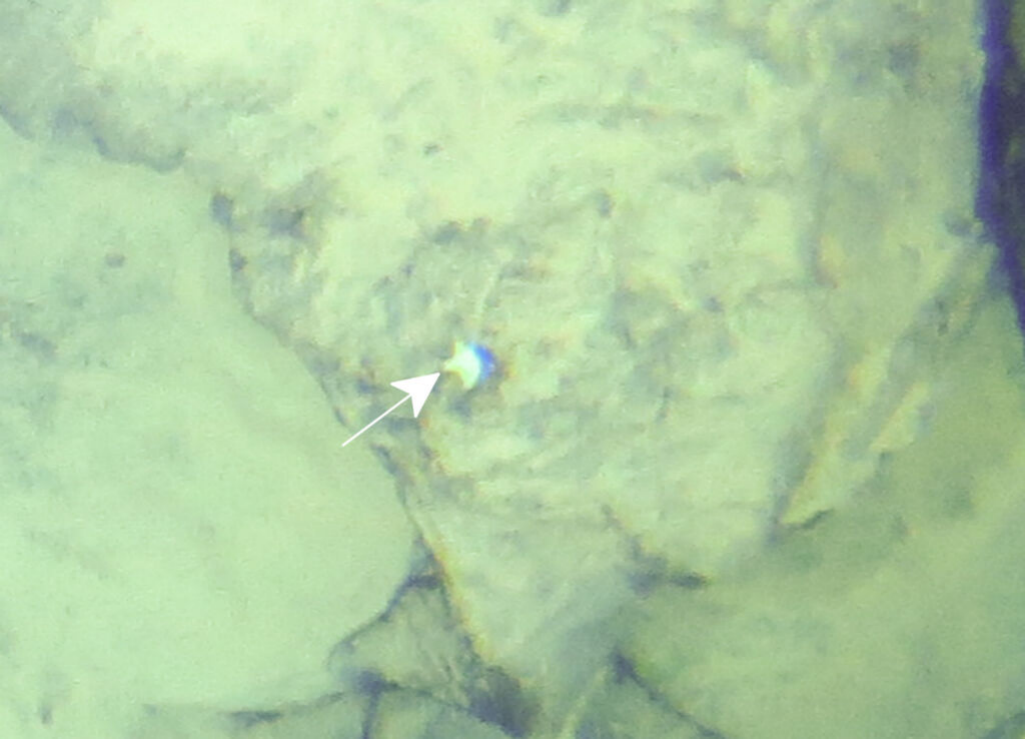
*Psammina* gen. inc. (DZMB_2021_0084) in situ at the South East Indian Ridge in Cluster 12 of the INDEX area. Image corresponds with the data (Image attribution: BGR).

**Figure 219.**
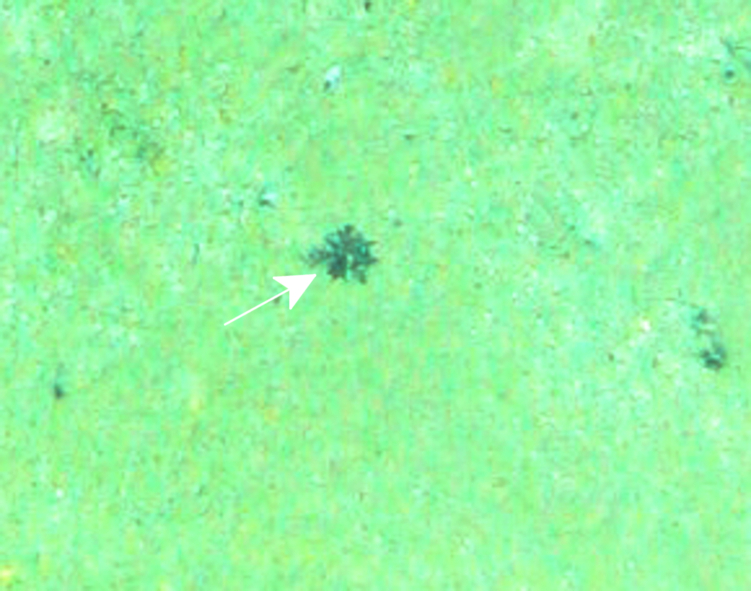
*Stannoma* gen. inc. in situ in the Edmond-vent site 2-vent site 7 area in Cluster 4 of the INDEX area. Image corresponds with the data (Image attribution: BGR).

**Table 1.** Detailed information of each expedition, including gear used, number of stations, photographs and video collected and distance covered. EGS = Edmond-vent site 2-vent site 7, SEIR = South East Indian Ridge, RTJ = Rodriguez Triple Junction, MFT = Multifunctional Tool, ROV = Remotely Operated Vehicle, VS = Video Sledge, ROPOS = Remotely Operated Platform for Ocean Science, STR = STROMER.

**Expedition**	**Vessel**	**Year**	**Time period**	**Cluster**	**Locality**	**Gear**	**No. of Stations**	**Photographs**	**Video (h: min)**	**Distance (m)**
**INDEX 2013**	RV Sonne	2013	23Oct – 21Dec	4,5	MESO, Kairei, EGS	MFT	4	15,124	-	11,529
						ROV Kiel 6000	11	595	105:57	18,617
**INDEX 2014**	RV Pelagia	2014	11Nov – 9Dec	6,8,9	SEIR, Pelagia	VS	11	64,606	-	33,820
**INDEX 2015**	RV Pelagia	2015	12Oct – 12Dec	4	vent site 1, EGS	VS	8	15,243	-	28,529
						ROV ROPOS	11	5,608	68:36	22,541
**INDEX 2016**	RV Pourquoi pas?	2016	3Jan – 3Feb	5,8	Kairei, Pelagia	ROV Victor 6000	5	3,402	81:52	38,277
**INDEX 2017**	RV Sonne	2017	25Aug – 13Oct	11,12	SEIR, vent site 5	STR	4	4,522	-	16,460
**INDEX 2018**	RV Pelagia	2018	10Oct – 17Dec	5,11,12	RTJ, vent sites 3-6	ROV ROPOS	16	4,817 (9,001 frame grabs)	110:45	51,210

**Table 2.** Detailed information of each sampling gear, including altitude above seafloor, speed range during imagery collection, camera angle, camera system, interval of taking still photos and information regarding the resolution (dots per inch (dpi); Megapixel (mp); Bit rate (kBits sec^-1^); Size of video imagery in pixel X pixel). All gears used flat port pressure bottles for video and still cameras with the exception of the ROV Victor 6000, which used dome port pressure bottles.

**Gear specifications**				**Camera specifications**				**Video specifications**			
**Gear**	**Altitude**	**Speed range**	**Angle**	**Camera system**	**Interval**	**Dots per inch**	**Megapixel**	**Frame rate**	**Bit rate**	**Pixel resolution**	**Megapixel**
**MFT**	0.5-5 m	0.3-1 knots	90°/ 45°	Canon Power Shot G9	10 sec	180 dpi	12 mp	25 fps	8,555 kBits sec^-1^	1440X1080	1.6 mp
**STR**	0.5-5 m	0.3-1 knots	90°/ 90°	Canon Power Shot G15	10 sec	180 dpi	12 mp	29 fps	15,186 kBits sec^-1^	1920X1080	2.1 mp
**VS**	0.5-5 m	0.3-1.5 knots	-/ 90°	-	-	-	-	25 fps	171,355 kBits sec^-1^	1920X1080	2.1 mp
**ROV Kiel 6000**	0.5-5 m	0.1-1 knots	10-90°	Canon Power Shot G5	-	180 dpi	5 mp	25 fps	4,128 kBits sec^-1^	704X576	0.4 mp
**ROV Victor 6000**	0.5-5 m	0.1-1 knots	10-90°	Video frame grabs	-	72 dpi	2.1 mp	25 fps	768 kBits sec^-1^	1440X1080	2.1 mp
**ROV Ropos**	0.5-8 m	0.1-1 knots	10-90°	Nikon D700/ D810	-	300 dpi	12 mp/ 36 mp	30 fps	50,384 kBits sec^-1^	1920X1080	2.1 mp

**Table 3.** List of all taxa in this fauna catalogue including phylum and the scientific name authority for the taxon. The asterisk and species names in squared brackets means that a taxon has been identified in several images, but a given identification level could not be supported in all images because not every single observation could be supported by morphological or molecular methods or contradictory results of different methods exist. Some putative taxa presented have an asterisk followed by an additional taxon name in squared brackets, as, for example, “Genus species *[Genus species sp. inc.]”, meaning that the image shown in the catalogue has the “Genus species” identification level, with further images, where the species level identification remains uncertain, indicated by “*[Genus species sp. inc.]”. The taxonomic ranks ˈcl.ˈ (“class”), ˈord.ˈ (“order”), ˈfam.ˈ (“family”), ˈgen.ˈ (“genus”) and ˈsp.ˈ (species) indicate the taxonomic rank and are always combined with the open nomenclature (ON) signs ˈindet.ˈ, ˈinc.ˈ according to Horton et al. (2021) or a unique code applied for this taxon (*taxon rank* (*unique code*)).

**Phylum**	**Taxon**	**Authority**
**Annelida**	*Archinomejasoni* *[*Archinomejasoni* sp. inc.]	Borda, Kudenov, Chevaldonné, Blake, Desbruyères, Fabri, Hourdez, Pleijel, Shank, Wilson, Schulze & Rouse, 2013
	Polynoidae gen. indet.	Kinberg, 1856
	*Branchipolynoe* gen. inc.	Pettibone, 1984
	*Lepidonotopodium* gen. inc. (DZMB_2021_0001)	Pettibone, 1983
	*Lepidonotopodium* gen. inc. (DZMB_2021_0002)	Pettibone, 1983
	*Lepidonotopodium* gen. inc. (DZMB_2021_0003)	Pettibone, 1983
	Sabellidae gen. indet.	Latreille, 1825
	*Oasisia* gen. inc.	Jones, 1985
	*Alvinella* gen. inc.	Desbruyeres & Laubier, 1980
**Arthropoda**	*Glyptelasma* gen. inc.	Pilsbry, 1907
	*Neolepasmarisindica* sp. inc.	Watanabe, Chen & Chan, 2018
	*Regioscalpellumregium* sp. inc.	(Wyville Thomson, 1873)
	Verrucidae fam. inc.	Darwin, 1854
	Amphipoda ord. inc.	Latreille, 1816
	Anomura fam. indet.	MacLeay, 1838
	Galatheidae fam. inc.	Samouelle, 1819
	*Munidopsisaries* sp. inc.	(A. Milne Edwards, 1880)
	*Munidopsispallida* sp. inc.	Alcock, 1894
	Paguroidea superfam. inc.	Latreille, 1802
	*Thymopideslaurentae* sp. inc.	Segonzac & Macpherson, 2003
	*Austinograearodriguezensis*	Tsuchida & Hashimoto, 2002
	*Alvinocarissolitaire* sp. inc. *[*Alvinocarissolitaire*]	Yahagi, Watanabe, Kojima & Beedesse, 2014
	*Mirocarisindica* sp. inc.	Komai, Martin, Zala, Tsuchida & Hashimoto, 2006
	*Rimicariskairei*	Watabe & Hashimoto, 2002
	*Nematocarcinus* gen. inc. (DZMB_2021_0004)	A. Milne-Edwards, 1881
	*Nematocarcinus* gen. inc. (DZMB_2021_0005)	A. Milne-Edwards, 1881
	Dendrobranchiata subord. inc.	Bate, 1888
	*Cerataspismonstrosus* sp. inc.	Gray, 1828
	Munnopsidae fam. inc. (DZMB_2021_0006)	Lilljeborg, 1864
	Munnopsidae fam. inc. (DZMB_2021_0007)	Lilljeborg, 1864
	Pantopoda ord. inc.	Gerstaecker, 1863
**Bryozoa**	Cheilostomatida fam. indet. (DZMB_2021_0008)	Busk, 1852
	Cheilostomatida fam. indet. (DZMB_2021_0009)	Busk, 1852
	*Bifaxaria* gen. inc.	Busk, 1884
	*Tessaradoma* gen. inc.	Norman, 1869
**Chordata**	Synaphobranchidae gen. indet.	Johnson, 1862
	*Histiobranchus* gen. inc.	Gill, 1883
	Synaphobranchidae*Ilyophisbrunneus* fam. inc.	Gilbert, 1891
	*Bathysaurusmollis* sp. inc.	Günther, 1878
	*Bathypterois* sp. indet.	Günther, 1878
	*Ipnopsagassizii* sp. inc.	Garman, 1899
	GadiformesMacrouridae ord. inc. (DZMB_2021_0010)	Bonaparte, 1831
	GadiformesMacrouridae ord. inc. (DZMB_2021_0011)	Bonaparte, 1831
	*Coryphaenoides* gen. inc. (DZMB_2021_0012)	Gunnerus, 1765
	*Coryphaenoides* gen. inc. (DZMB_2021_0013)	Gunnerus, 1765
	*Coryphaenoidesarmatus* sp. inc.	(Hector, 1875)
	*Coryphaenoideslongifilis* sp. inc.	Günther, 1877
	*Antimorarostrata*	(Günther, 1878)
	*Chaunacops* gen. inc.	Garman, 1899
	Notacanthiformes ord. inc.	L. S. Berg, 1947
	*Aldrovandiaaffinis* gen. inc.	(Günther, 1877)
	*Halosauropsismacrochir* gen. inc.	(Günther, 1878)
	Ophidiidae gen. indet. (DZMB_2021_0014)	Rafinesque, 1810
	Ophidiidae gen. indet. (DZMB_2021_0015)	Rafinesque, 1810
	Ophidiidae fam. inc. (DZMB_2021_0016)	Rafinesque, 1810
	*Acanthonusarmatus* gen. inc.	Günther, 1878
	*Barathritesiris* gen. inc.	Zugmayer, 1911
	*Bassozetus* gen. inc.	Gill, 1883
	*Spectrunculuscrassus* sp. inc.	(Vaillant, 1888)
	*Spectrunculusgrandis* sp. inc.	(Günther, 1877)
	*Xyelacybamyersi* gen. inc.	Cohen, 1961
	*Pachycaraangeloi*	Thiel, Knebelsberger, Kihara & Gerdes, 2021
	Octacnemidae gen. indet.	-
	*Culeolus* spp. indet.	Herdman, 1881
	*Bathyrajatunae* sp. inc.	Stehmann, 2005
**Cnidaria**	Cnidaria cl. indet.	Hatschek, 1888
	Ceriantharia ord. indet.	Perrier, 1893
	Spirularia fam. indet.	den Hartog, 1977
	Actiniaria fam. indet. (DZMB_2021_0017)	Hertwig, 1882
	Actiniaria fam. indet. (DZMB_2021_0018)	Hertwig, 1882
	Actiniaria fam. indet. (DZMB_2021_0019)	Hertwig, 1882
	Actiniaria fam. indet. (DZMB_2021_0020)	Hertwig, 1882
	Actiniaria fam. indet. (DZMB_2021_0021)	Hertwig, 1882
	Actiniaria fam. indet. (DZMB_2021_0022)	Hertwig, 1882
	Actiniaria fam. indet. (DZMB_2021_0023)	Hertwig, 1882
	Actiniaria fam. indet. (DZMB_2021_0024)	Hertwig, 1882
	Actiniaria fam. indet. (DZMB_2021_0025)	Hertwig, 1882
	Actinoscyphiidae gen. indet. (DZMB_2021_0026)	Stephenson, 1920
	Actinoscyphiidae gen. indet. (DZMB_2021_0027)	Stephenson, 1920
	*Actinoscyphia* sp. indet.	Stephenson, 1920
	Actinostolidae gen. indet.	Carlgren, 1932
	*Actinostola* sp. indet. (DZMB_2021_0028)	Verrill, 1883
	*Actinostola* sp. indet. (DZMB_2021_0029)	Verrill, 1883
	*Actinostola* sp. indet. (DZMB_2021_0030)	Verrill, 1883
	*Actinostola* sp. indet. (DZMB_2021_0031)	Verrill, 1883
	*Bathyphellia* sp. indet. (DZMB_2021_0032)	Carlgren, 1932
	*Bathyphellia* sp. indet. (DZMB_2021_0033)	Carlgren, 1932
	*Chondrophellia* sp. indet.	Carlgren, 1925
	*Maractis* sp. indet.	Fautin & Barber, 1999
	*Relicanthusdaphneae* sp. inc.	(Daly, 2006)
	Alcyonacea fam. indet.	Lamouroux, 1812
	Alcyonacea*Anthomastus* gen. inc.	Verrill, 1878
	*Anthomastus* sp. indet.	Verrill, 1878
	*Chrysogorgia* sp. indet. (DZMB_2021_0034)	Duchassaing & Michelotti, 1864
	*Chrysogorgia* sp. indet. (DZMB_2021_0035)	Duchassaing & Michelotti, 1864
	*Iridogorgiamagnispiralis* sp. inc.	Watling, 2007
	Clavulariidae gen. indet. (DZMB_2021_0036)	Hickson, 1894
	Clavulariidae gen. indet. (DZMB_2021_0037)	Hickson, 1894
	Clavulariidae fam. inc. (DZMB_2021_0038)	Hickson, 1894
	Clavulariidae fam. inc. (DZMB_2021_0039)	Hickson, 1894
	Isididae gen. indet. (DZMB_2021_0040)	Lamouroux, 1812
	Isididae gen. indet. (DZMB_2021_0041)	Lamouroux, 1812
	Isididae gen. indet. (DZMB_2021_0042)	Lamouroux, 1812
	Isididae gen. indet. (DZMB_2021_0043)	Lamouroux, 1812
	Isididae fam. inc. (DZMB_2021_0044)	Lamouroux, 1812
	Isididae gen. indet. (DZMB_2021_0045)	Lamouroux, 1812
	Isididae*Acanella* gen. inc.	Gray, 1870
	Isididae*Bathygorgia* gen. inc.	Wright, 1885
	Isididae*Jasonisis* gen. inc.	Alderslade & McFadden, 2012
	Isididae*Keratoisis* gen. inc. (DZMB_2021_0046)	Wright, 1869
	Isididae*Keratoisis* gen. inc. (DZMB_2021_0047)	Wright, 1869
	Isididae*Lepidisis* gen. inc.	Verrill, 1883
	*Lepidisis* spp. indet.	Verrill, 1883
	Paragorgiidae fam. inc.	Kükenthal, 1916
	Primnoidae gen. indet. (DZMB_2021_0048)	Milne Edwards, 1857
	Primnoidae gen. indet. (DZMB_2021_0049)	Milne Edwards, 1857
	Stalk of Alcyonacea or Antipatharia ord. inc.	Lamouroux, 1812/ -
	*Heteropathes* sp. indet.	Opresko, 2011
	*Heteropathesamericana* sp. inc.	(Opresko, 2003)
	*Bathypathes* sp. indet. (DZMB_2021_0050)	Brook, 1889
	*Bathypathes* gen. inc. (DZMB_2021_0051)	Brook, 1889
	*Bathypathespatula* sp. inc.	Brook, 1889
	*Schizopathes* spp. indet.	Brook, 1889
	Pennatulacea ord. inc. (DZMB_2021_0052)	Verrill, 1865
	Pennatulacea fam. indet. (DZMB_2021_0053)	Verrill, 1865
	Pennatulacea*Kophobelemnon* ord. inc.	Asbjörnsen, 1856
	*Umbellula* sp. indet. (DZMB_2021_0054)	Gray, 1870
	*Umbellula* sp. indet. (DZMB_2021_0055)	Gray, 1870
	Zoantharia fam. indet. (DZMB_2021_0056)	Gray, 1832
	Zoantharia fam. indet. (DZMB_2021_0057)	Gray, 1832
	Zoantharia fam. indet. (DZMB_2021_0058)	Gray, 1832
	*Epizoanthus* sp. indet.	Gray, 1867
	Hydrozoa ord. indet. (DZMB_2021_0059)	Owen, 1843
	Hydrozoa ord. indet. (DZMB_2021_0060)	Owen, 1843
	Hydrozoa ord. indet. (DZMB_2021_0061)	Owen, 1843
	Hydrozoa ord. indet. (DZMB_2021_0062)	Owen, 1843
	Hydrozoa ord. indet. (DZMB_2021_0063)	Owen, 1843
	Hydrozoa ord. indet. (DZMB_2021_0064)	Owen, 1843
	Hydrozoa ord. indet. (DZMB_2021_0065)	Owen, 1843
	*Candelabrum* sp. indet	de Blainville, 1830
	Corymorphidae gen. indet.	Allman, 1872
	SiphonophoraeRhodaliidae*Thermopalia* gen. inc.	Pugh, 1983
**Echinodermata**	*Hymenodiscus* gen. inc.	Perrier, 1884
	Freyellidae fam. inc.	Downey, 1986
	*Freyastera* gen. inc.	Downey, 1986
	*Freyella* gen. inc.	Perrier, 1885
	*Styracaster* gen. inc.	Sladen, 1883
	*Henricia* gen. inc.	Gray, 1840
	Goniasteridae gen. indet. (DZMB_2021_0066)	Forbes, 1841
	Goniasteridae gen. indet. (DZMB_2021_0067)	Forbes, 1841
	*Circeaster* gen. inc.	Koehler, 1909
	*Evoplosoma* gen. inc.	Fisher, 1906
	*Lydiasterjohannae* sp. inc.	Koehler, 1909
	Solasteridae fam. inc.	Viguier, 1878
	*Asthenactis* gen. inc.	Fisher, 1906
	*Hymenaster* sp. indet.	Wyville Thomson, 1873
	*Pteraster* gen. inc.	Müller & Troschel, 1842
	Antedonidae gen. indet. (DZMB_2021_0068)	Norman, 1865
	Antedonidae fam. inc. (DZMB_2021_0069)	Norman, 1865
	*Bathymetra* gen. inc.	AH Clark, 1908
	*Pentametrocrinus* sp. indet.	AH Clark, 1908
	Hyocrinidae gen. indet.	Carpenter, 1884
	Irregularia infracl. inc.	Latreille, 1825
	Cidaroida fam. indet.	Claus, 1880
	*Hapalosoma* sp. indet.	Mortensen, 1903
	*Salenocidaris* sp. indet.	Agassiz, 1869
	*Chiridotahydrothermica* sp. inc.	Smirnov & Gebruk, 2000
	Elpidiidae gen. indet. (DZMB_2021_0070)	Théel, 1882
	Elpidiidae gen. indet. (DZMB_2021_0071)	Théel, 1882
	Elpidiidae gen. indet. (DZMB_2021_0072)	Théel, 1882
	*Peniagonepurpurea*	(Théel, 1882)
	Laetmogonidae gen. indet.	Ekman, 1926
	*Enypniasteseximia*	Théel, 1882
	*Benthodytes* sp. indet.	Théel, 1882
	*Benthothuria* gen. inc.	Perrier R., 1898
	*Pseudostichopus* gen. inc. (DZMB_2021_0073)	Théel, 1886
	*Pseudostichopus* sp. indet. (DZMB_2021_0074)	Théel, 1886
	*Oneirophanta* sp. indet.	Théel, 1879
	Synallactidae gen. indet. (DZMB_2021_0075)	Ludwig, 1894
	Synallactidae gen. indet. (DZMB_2021_0076)	Ludwig, 1894
	Synallactidae gen. indet. (DZMB_2021_0077)	Ludwig, 1894
	Synallactidae gen. indet. (DZMB_2021_0078)	Ludwig, 1894
	Synallactidae fam. inc. (DZMB_2021_0079)	Ludwig, 1894
	*Synallactes* sp. indet.	Ludwig, 1894
	Amphilepidida ord. inc.	O'Hara, Hugall, Thuy, Stöhr & Martynov, 2017
	*Asteronyx* gen. inc.	Müller & Troschel, 1842
	Ophiacanthida ord. inc.	O'Hara, Hugall, Thuy, Stöhr & Martynov, 2017
	*Ophiophyllumpetilum* sp. inc.	Lyman, 1878
	*Ophiosphalma* gen. inc.	H.L. Clark, 1941
	*Ophiosphalmaarmigerum* sp. inc.	(Lyman, 1878)
**Hemichordata**	Torquaratoridae fam. inc.	Holland, Clague, Gordon, Gebruk, Pawson & Vecchione, 2005
**Mollusca**	*Bathymodiolusseptemdierum* *[*Bathymodiolusseptemdierum* sp. inc.]	Hashimoto & Okutani, 1994
	*Bathypolypus* sp. indet.	Grimpe, 1921
	*Cirroteuthis* sp. indet.	Eschricht, 1838
	*Grimpoteuthis* gen. inc.	Robson, 1932
	*Magnapinna* sp. indet.	Vecchione & Young, 1998
	Abyssochrysoidea superfam. inc.	Tomlin, 1927
	*Speculator* gen. inc.	Waren & Bouchet, 2001
	*Alviniconchamarisindica*	Okutani, 2014
	Lepetodrilidae fam. inc.	McLean, 1988
	*Lepetodrilus* gen. inc.	McLean, 1988
	Lepetodrilidae*Lepetodrilus* sp. indet. *[Lepetodrilidae*Lepetodrilus* gen. inc.]	McLean, 1988
	*Phymorhynchus* sp. indet.	Dall, 1908
	*Phymorhynchus* sp. indet. (Egg capsules)	Dall, 1908
	Melanodrymiidae fam. inc.	Salvini-Plawen & Steiner, 1995
	*Chrysomallonsquamiferum*	C. Chen, Linse, Copley & Rogers, 2015
	Scaphopoda ord. indet.	Bronn, 1862
	Solenogastres ord. indet.	Gegenbaur, 1878
**Nemertea**	*Thermanemertes* gen. inc.	Rogers, Gibson & Tunnicliffe, 1996
**Platyhelminthes**	Polycladida fam. indet. *[Polycladida ord. inc.]	Lang, 1884
**Porifera**	*Paleodictyonnodosum*	Seilacher, 1977
**Foraminifera**	Monothalamea ord. indet. (DZMB_2021_0080)	Haeckel, 1862 (as emended by Pawlowski et al., 2013)
	Monothalamea ord. indet. (DZMB_2021_0081)	Haeckel, 1862 (as emended by Pawlowski et al., 2013)
	Monothalamea ord. indet. (DZMB_2021_0082)	Haeckel, 1862 (as emended by Pawlowski et al., 2013)
	*Luffammina* gen. inc.	Kamenskaya, Bagirov & Simdianov, 2002
	*Psammina* gen. inc. (DZMB_2021_0083)	Haeckel, 1889
	*Psammina* gen. inc. (DZMB_2021_0084)	Haeckel, 1889
	*Stannoma* gen. inc.	Haeckel, 1889

**Table 4.** Brief description of the 78 fields of the Suppl. material 1, including the Field name and a Field data description in a separate column.

**Field name**	**Field data description**
**Field ID**	Unique ID for each data entry
**Cruise Name**	Name of the research expedition
**Research Vessel**	Name of the vessel on which the expedition took place
**Leg Number**	Contains the leg of a single expedition during which the data were generated
**Geographical Area**	Mid Ocean Ridge segment where the data were collected
**Area sector**	Contains information if the data were collected within or outside a cluster and in which cluster the data were collected. The clusters are within the German licence area for the exploration of polymetallic sulphide occurrences and were issued by the Federal Institute for Geosciences and Natural Resources (www.bgr.de) on behalf of the International Seabed Authority (www.isa.org.jm)
**Year**	Year of the expedition
**Sampling Date**	Date of data collection (year-month-day)
**Time stamp (on video/still photo)**	Time (hours:minutes:seconds) of the data collection in UTC
**Sampling start time**	Start time of the individual transect (hours:minutes)
**Sampling start depth**	Depth (m) at the starting point of the transect
**Sampling start latitude**	Latitude at the starting point of the transect
**Sampling start longitude**	Longitude at the starting point of the transect
**Sampling end time**	End time of the individual transect (hours:minutes)
**Sampling end depth**	Depth (m) at the end point of the transect
**Sampling end latitude**	Latitude at the end point of the transect
**Sampling end longitude**	Longitude at the end point of the transect
**Area or Volume sampled (m)**	Length (m) of the entire transect
**Locality**	Local name of the sampling area or hydrothermal vent field name
**Geodetic Datum**	Global reference frame for precisely measuring locations on Earth
**Coordinate uncertainty in Meters**	Accurracy deviation of the underwater acoustic Ultra-short-baseline (USBL) positioning in metres
**Station ID**	Name of individual station including the year of expedition
**Transect ID**	Name of the individual transect
**Sample ID**	Name of the individual sample collected
**Voucher Specimen Code**	Unique code for each sampled specimen
**Marker**	Genetic marker targeted
**PCR Result**	Descriptor for the success of extracting DNA with the Polymerase chain reaction (PCR)
**Sequence Result**	Descriptor for the successful sequencing result of the extracted DNA
**Photograph frame code of sampled specimen**	Photograph name of the sampled specimen
**Sampling Gear (code)**	Abbreviation of the tool or gear used for the data collection
**Preparations**	Descriptor for the preparation of collected data including information of the fixation of sampled specimens
**Institution Storing Imagery and Samples**	Abbreviation of Institute where samples are stored
**Recorded By**	Abbreviation of Institute that has the copyright of imagery
**Occurrence Status**	Indication for the occurrence status of the identified taxon in the data
**Identification Remarks**	Indicating potential limitations related to the available material and data type used for the identification (imagery, physical sample or both)
**Language**	Language of the data entry
**Basis of Record**	Descriptor if the identification record was based on Human observtaion (indirect, imagery only) or Preserved Specimen (direct, including physical samples)
**Dataset Name**	Name of the Dataset, equivalent with the project name INDEX (Indian Ocean Exploration Project)
**Number of sampled individuals**	Indicating how many of the photographed individuals were collected and are present as a physical sample
**Number of counted individuals**	Number of individuals of a specific taxon that were counted in the photograph present. The number "0" indicates different photograph or sample of an identical specimen, the number "100" indicates that precise counting was not possible and an uncountable number of individuals was present.
**Frame Code (on video/still photo)**	Name of the photograph or frame grab showing the identified taxon
**Area of image**	Showing the total area of each photograph or frame grab in pixels (length x width)
**Video/photo sled ID code**	Name of the tool or gear used for the data collection
**Technical specifications of camera equipment**	Specification of the camera used or if a frame grab was extracted from a high definition (hd) or standard definition (sd) video
**Kingdom**	Taxonomic classification hierarchy level: Kingdom
**Identification: Phylum**	Taxonomic classification hierarchy level: Phylum
**Identification: Class**	Taxonomic classification hierarchy level: Class
**Identification: Order**	Taxonomic classification hierarchy level: Order
**Identification: Family**	Taxonomic classification hierarchy level: Family
**Identification: Genus**	Taxonomic classification hierarchy level: Genus
**Identification: Species**	Taxonomic classification hierarchy level: Species
**Taxon rank**	Lowest possible identification level
**Identification Qualifier**	Descriptor for the confidence of the identification level
**Scientific Name authorship**	Authority and year of the original taxon description
**Identification: putative species name or number**	Putative taxon name regardless the identification level including the identification qualifier
**Identification Molecular**	Result of the molecular identification if present
**Morphological Taxonomist**	Responsible taxonomist who identified the taxon the putative taxon name or number
**Morphological Taxonomist E-mail**	Current email adress of the taxonomist
**Morphological Taxonomist Institution**	Current Institution of the taxonomist
**Behaviour**	Behaviour of the observed and identified individual
**Specimen Details: Life Stage**	Life stage of the identified individual, if possible
**Specimen Details : Tissue Descriptor**	Tissue used for the molecular DNA extraction
**Specimen Details: Associated Taxa**	Associated taxa in the close vicinity of the identified taxon
**Specimen Details : Associated Specimens**	Associated specimens in symbiosis or attached to the identified individual
**Hydrothermal activity**	Indicating if the identified taxon was observed in an area with hydrothermal activity or not
**Activity of hydrothermal vent site (active/inactive/dormant/diffuse flow)**	Indicating the level of hydrothermal activity from high to low/no activity in the categories "active", "diffuse flow", "inactive", "dormant", "non-vent", respectively
**Age of hydrothermal vent (100 - >10,000 years)**	Estimated and categorised age of hydrothermal vent field from young (100 years) to old (10,000 years)
**Water Body**	Ocean in which data or samples were collected
**Water Temperature (°C)**	Water temperature in degrees Celsius at the location of the observed individual (if measured)
**Salinity (ppt)**	Salinity in parts per thousand at the location of the observed individual (if measured)
**Depth (m)**	Depth (m) at the location of the observed individual
**Image Type**	Indicating if the data were derived from a photograph or from video imagery
**Exposure**	Exposure time of the camera used
**ISO-speed**	Indicating the sensitivity of the CMOS sensor towards light. A higher ISO speed indicates higher sensitivity to light.
**Focal length**	Measure of how strongly the camera converges the light
**Use of picture**	Descriptor of which photographs were extracted for identification, were extracted and send to taxonomists for precise identification and extracted, identified and shown in the publication as an example of that taxon
**Latitude**	Latitude in decimal degrees of the observed individual
**Longitude**	Longitude in decimal degrees of the observed individual

**Table 5.** Number of taxa/species in each phylum at the Central Indian Ridge (CIR), the South East Indian Ridge (SEIR) and the Rodriguez Triple Junction (RTJ). Numbers include active vent taxa, inactive vent taxa and non-vent taxa. Note, that the phylum Porifera was excluded from this catalogue, with the exception of a single species.

**Phylum**	**Taxa CIR**	**Taxa SEIR**	**Taxa RTJ**	**Taxa total**
**Annelida**	5	8	3	**9**
**Arthropoda**	15	17	12	**22**
**Bryozoa**	3	4	1	**4**
**Chordata**	16	19	16	**30**
**Cnidaria**	42	47	28	**77**
**Echinodermata**	26	37	12	**48**
**Hemichordata**	-	1	-	**1**
**Mollusca**	8	14	10	**17**
**Nemertea**	-	-	1	**1**
**Platyhelminthes**	1	-	1	**1**
**Porifera**	1	1	-	**1**
**Foraminifera**	5	4	2	**7**
**Total**	**122**	**152**	**86**	**218**

**Table 6.** List of active vent species for the Central Indian Ridge (CIR), the South East Indian Ridge (SEIR) and the Rodriguez Triple Junction (RTJ) within active hydrothermal vent fields in each region. Presence of each taxon indicated as low ('+'), medium ('++') or high ('+++') density. Low density = 1 specimen, medium = 2-9 specimens and high ≥ 10 specimens. Endemic active vent taxa are highlighted in bold.

**Phylum**	**Active vent taxa**	**CIR**	**SEIR**	**RTJ**
**Annelida**	*Archinomejasoni* *[*Archinomejasoni* sp. inc.]	+++	+++	+++
	***Branchipolynoe* gen. inc.**	+		
	***Lepidonotopodium* gen. inc. (DZMB_2021_0001)**	++	++	++
	***Oasisia* gen. inc.**	+++	+++	
	***Alvinella* gen. inc.**		+++	
**Arthropoda**	***Neolepasmarisindica* sp. inc.**	+++	+++	+++
	***Regioscalpellumregium* sp. inc.**		+++	
	**Verrucidae fam. inc.**		+++	+++
	*Munidopsispallida* sp. inc.		++	
	***Austinograearodriguezensis***	+++	+++	+++
	***Alvinocarissolitaire* sp. inc.**	+++	+++	+++
	***Mirocarisindica* sp. inc.**	+++	++	++
	***Rimicariskairei***	+++	+++	+++
	Pantopoda ord. inc.		++	
**Chordata**	Synaphobranchidae gen. indet.	+	++	
	Synaphobranchidae*Ilyophisbrunneus* fam. inc.			+
	Coryphaenoides gen. inc. (DZMB_2021_0012)	+	+	
	*Coryphaenoidesarmatus* sp. inc.		+	+
	*Halosauropsismacrochir* gen. inc.		+	
	*Spectrunculuscrassus* sp. inc.		++	
	*Spectrunculusgrandis* sp. inc.		++	+
	**Pachycaraangeloi**	++	++	++
**Cnidaria**	Ceriantharia ord. indet.			+
	Actiniaria fam. indet. (DZMB_2021_0017)	+++		
	Actiniaria fam. indet. (DZMB_2021_0018)		++	
	Actiniaria fam. indet. (DZMB_2021_0019)	+++	++	+++
	**Actiniaria fam. indet. (DZMB_2021_0020)**	+++		+
	Actiniaria fam. indet. (DZMB_2021_0021)	+++	++	
	Actiniaria fam. indet. (DZMB_2021_0022)	+		
	Actiniaria fam. indet. (DZMB_2021_0023)	+		
	**Actiniaria fam. indet. (DZMB_2021_0024)**	++		
	Actiniaria fam. indet. (DZMB_2021_0025)	++		
	Actinoscyphiidae gen. indet. (DZMB_2021_0026)	+	+	
	*Actinoscyphia* sp. indet.		+	++
	Actinostolidae gen. indet.	++	++	+++
	*Actinostola* sp. indet. (DZMB_2021_0028)		+++	+
	*Actinostola* sp. indet. (DZMB_2021_0029)		+	
	*Actinostola* sp. indet. (DZMB_2021_0030)	+	+++	
	*Actinostola* sp. indet. (DZMB_2021_0031)		+	
	*Bathyphellia* sp. indet. (DZMB_2021_0032)	++	++	
	*Bathyphellia* sp. indet. (DZMB_2021_0033)		+++	
	***Maractis* sp. indet.**	+++	+++	+++
	*Relicanthusdaphneae* sp. inc.	+	+	
	Alcyonacea*Anthomastus* gen. inc.		+	
	*Iridogorgiamagnispiralis* sp. inc.		++	
	Clavulariidae gen. indet. (DZMB_2021_0036)	+++	+++	+++
	Clavulariidae gen. indet. (DZMB_2021_0037)	+		
	Clavulariidae fam. inc. (DZMB_2021_0038)	++		
	**Clavulariidae fam. inc. (DZMB_2021_0039)**	++		
	Isididae gen. indet. (DZMB_2021_0040)		+	
	Isididae gen. indet. (DZMB_2021_0041)		+	
	Isididae gen. indet. (DZMB_2021_0043)		+	+
	Isididae fam. inc. (DZMB_2021_0044)		+	
	Isididae*Bathygorgia* gen. inc.		++	+
	Isididae*Jasonisis* gen. inc.		++	
	Isididae*Keratoisis* gen. inc. (DZMB_2021_0047)			++
	Isididae*Lepidisis* gen. inc.		++	
	*Lepidisis* spp. indet.		+++	++
	Paragorgiidae fam. inc.		+	
	Primnoidae gen. indet. (DZMB_2021_0048)			+
	Primnoidae gen. indet. (DZMB_2021_0049)		++	
	Stalk of Alcyonacea or Antipatharia ord. inc.	+		
	*Heteropathesamericana* sp. inc.		+	
	*Bathypathes* sp. indet. (DZMB_2021_0050)			+
	*Bathypathespatula* sp. inc.	++	++	++
	*Schizopathes* spp. indet.		+	+
	*Umbellula* sp. indet. (DZMB_2021_0054)	++		
	*Umbellula* sp. indet. (DZMB_2021_0055)		++	
	Zoantharia fam. indet. (DZMB_2021_0056)	+++		+++
	Zoantharia fam. indet. (DZMB_2021_0057)	+++	+++	+++
	Hydrozoa ord. indet. (DZMB_2021_0060)			+++
	Hydrozoa ord. indet. (DZMB_2021_0061)			+++
	Hydrozoa ord. indet. (DZMB_2021_0062)	+		
	Hydrozoa ord. indet. (DZMB_2021_0063)			++
	Hydrozoa ord. indet. (DZMB_2021_0064)			+++
	***Candelabrum* sp. indet.**	+++	+	++
	Corymorphidae gen. indet.		+	
	SiphonophoraeRhodaliidae*Thermopalia* gen. inc.	++		
**Echinodermata**	*Freyella* gen. inc.			+
	***Chiridotahydrothermica* sp. inc.**	++	+++	
**Mollusca**	*Bathymodiolusseptemdierum* *[*Bathymodiolusseptemdierum* sp. inc.]	+++	+++	+++
	**Abyssochrysoidea superfam. inc.**		+++	
	***Alviniconchamarisindica***	+++	+++	+++
	**Lepetodrilidae fam. inc.**		+++	+++
	***Lepetodrilus* gen. inc.**		+++	
	Lepetodrilidae*Lepetodrilus* sp. indet. *[Lepetodrilidae*Lepetodrilus* gen. inc.]		+++	+++
	***Phymorhynchus* sp. indet.**	+++	+++	+++
	***Phymorhynchus* sp. indet. (Egg capsules)**	+++	+++	+++
	Melanodrymiidae fam. inc.			+++
	***Chrysomallonsquamiferum***		+++	+++
	Solenogastres ord. indet.	+	+	
**Nemertea**	*Thermanemertes* gen. inc.			+++
**Platyhelminthes**	**Polycladida ord. inc.**	+++		+++

**Table 7.** List of inactive vent species for the Central Indian Ridge (CIR), the South East Indian Ridge (SEIR) and the Rodriguez Triple Junction (RTJ) within inactive hydrothermal vent fields in each region. Presence of each taxon indicated as low ('+'), medium ('++') or high ('+++') density. Low density = 1 specimen, medium = 2-9 specimens and high ≥ 10 specimens. Taxa found exclusively within or in close proximity to inactive hydrothermal vent fields are highlighted in bold, taxa observed only within inactive vent fields are additionally indicated by a plus.

**Phylum**	**Inactive vent taxa**	**CIR**	**SEIR**	**RTJ**
**Annelida**	Polynoidae gen. indet.		++	++
	***Lepidonotopodium* gen. inc. (DZMB_2021_0002) ^+^**		+	
	***Lepidonotopodium* gen. inc. (DZMB_2021_0003) ^+^**		+	
**Arthropoda**	***Glyptelasma* gen. inc.**		+++	+
	**Amphipoda ord. inc. ^+^**			+
	*Munidopsisaries* sp. inc.			+
	*Munidopsispallida* sp. inc.	++	++	
	*Thymopideslaurentae* sp. inc.		+	
	*Nematocarcinus* gen. inc. (DZMB_2021_0004)	+		
	*Nematocarcinus* gen. inc. (DZMB_2021_0005)	+	+	
	*Cerataspismonstrosus* sp. inc.		++	
	**Munnopsidae fam. inc. (DZMB_2021_0007) ^+^**			+++
**Bryozoa**	**Cheilostomatida fam. indet. (DZMB_2021_0009) ^+^**		+++	
**Chordata**	Synaphobranchidae gen. indet.	++	++	+
	***Histiobranchus* gen. inc.**	+		
	***Coryphaenoideslongifilis* sp. inc.**		+	++
	*Halosauropsismacrochir* gen. inc.		++	
	**Ophidiidae gen. indet. (DZMB_2021_0014**)		+	
	*Bassozetus* gen. inc.		+	
	*Spectrunculusgrandis* sp. inc.		++	
**Cnidaria**	Spirularia fam. indet.	++		
	Actiniaria fam. indet. (DZMB_2021_0017)	++		
	Actiniaria fam. indet. (DZMB_2021_0018)	+		
	Actiniaria fam. indet. (DZMB_2021_0020)		+	
	Actiniaria fam. indet. (DZMB_2021_0021)	+		
	Actiniaria fam. indet. (DZMB_2021_0022)	++		
	*Actinoscyphia* sp. indet	++	+	
	Actinostolidae gen. indet.	+	++	
	*Actinostola* sp. indet. (DZMB_2021_0028)	+	+	
	*Bathyphellia* sp. indet. (DZMB_2021_0032)	++		
	***Chondrophellia* sp. indet. ^+^**	++		
	**Alcyonacea fam. indet. ^+^**	++		
	***Anthomastus* sp. indet. ^+^**	++		
	***Chrysogorgia* sp. indet. (DZMB_2021_0034) ^+^**	+		
	***Chrysogorgia* sp. indet. (DZMB_2021_0035) ^+^**	++		
	*Iridogorgiamagnispiralis* sp. inc.		+++	
	Clavulariidae gen. indet. (DZMB_2021_0036)	+	++	
	Clavulariidae gen. indet. (DZMB_2021_0037)	+		
	Isididae gen. indet. (DZMB_2021_0040)		+	
	Isididae gen. indet. (DZMB_2021_0042)		++	
	**Isididae gen. indet. (DZMB_2021_0045) ^+^**	+		
	Isididae*Acanella* gen. inc.		++	
	Isididae*Bathygorgia* gen. inc.	++	++	
	Isididae*Jasonisis* gen. inc.		++	
	Isididae*Lepidisis* gen. inc.		++	
	*Lepidisis* spp. indet.	++	++	
	Primnoidae gen. indet. (DZMB_2021_0049)		++	
	Stalk of Alcyonacea or Antipatharia ord. inc.	++		
	*Heteropathes* sp. indet.		++	
	***Bathypathes* gen. inc. (DZMB_2021_0051)**		+	
	*Bathypathespatula* sp. inc.	++		
	**Pennatulacea ord. inc. (DZMB_2021_0052)**	+		
	**Pennatulacea*Kophobelemnon* ord. inc.**	+		
	Zoantharia fam. indet. (DZMB_2021_0056)	+++	+++	
	Zoantharia fam. indet. (DZMB_2021_0057)	+++	+++	
	**Zoantharia fam. indet. (DZMB_2021_0058) ^+^**		+++	
	**Hydrozoa ord. indet. (DZMB_2021_0059) ^+^**		+++	
	Hydrozoa ord. indet. (DZMB_2021_0062)		++	
**Echinodermata**	Freyellidae fam. inc.	+		
	*Freyella* gen. inc.	++		
	Goniasteridae gen. indet. (DZMB_2021_0066)		+	
	***Circeaster* gen. inc. ^+^**	+	++	
	*Hapalosoma* sp. indet.	+		
	Synallactidae gen. indet. (DZMB_2021_0077)			+
	*Synallactes* sp. indet.		++	
	***Ophiosphalma* gen. inc. ^+^**		+	
	*Ophiosphalmaarmigerum* sp. inc.		++	+
**Mollusca**	Abyssochrysoidea superfam. inc.		+++	
	*Phymorhynchus* sp. indet. (Egg capsules)		+++	

**Table 8.** List of non-vent species for the Central Indian Ridge (CIR), the South East Indian Ridge (SEIR) and the Rodriguez Triple Junction (RTJ). Presence of each taxon indicated as low ('+'), medium ('++') or high ('+++') density. Low density = 1 specimen, medium = 2-9 specimens and high ≥ 10 specimens.

**Phylum**	**Non-vent taxa**	**CIR**	**SEIR**	**RTJ**
**Annelida**	Polynoidae gen. indet.			++
	Sabellidae gen. indet.	++	+++	
**Arthropoda**	Anomura fam. indet.	++		
	Galatheidae fam. inc.	++	+	
	*Munidopsisaries* sp. inc.	++	+	+
	*Munidopsispallida* sp. inc.	++	++	
	Paguroidea superfam. inc.	++		
	*Thymopideslaurentae* sp. inc.	+		
	*Nematocarcinus* gen. inc. (DZMB_2021_0004)	+++	++	++
	*Nematocarcinus* gen. inc. (DZMB_2021_0005)	+++		
	Dendrobranchiata subord. inc.		+	
	*Cerataspismonstrosus* sp. inc.	++	+++	++
	Munnopsidae fam. inc. (DZMB_2021_0006)	+++		
**Bryozoa**	Cheilostomatida fam. indet. (DZMB_2021_0008)	++	++	
	*Bifaxaria* gen. inc.	++	++	+
	*Tessaradoma* gen. inc.	+++	+	
**Chordata**	Synaphobranchidae gen. indet.		++	++
	*Bathysaurusmollis* sp. inc.	++	++	++
	*Bathypterois* sp. indet.	++	+	
	*Ipnopsagassizii* sp. inc.	++	+	
	GadiformesMacrouridae ord. inc. (DZMB_2021_0010)		+	
	GadiformesMacrouridae ord. inc. (DZMB_2021_0011)		+	
	*Coryphaenoides* gen. inc. (DZMB_2021_0013)		++	
	*Coryphaenoidesarmatus* sp. inc.	++		+
	*Antimorarostrata*			++
	*Chaunacops* gen. inc.		+	
	Notacanthiformes ord. inc.	++		
	*Aldrovandiaaffinis* gen. inc.	++	+++	
	*Halosauropsismacrochir* gen. inc.		+++	++
	Ophidiidae gen. indet. (DZMB_2021_0015)			+
	Ophidiidae fam. inc. (DZMB_2021_0016)	+		++
	*Acanthonusarmatus* gen. inc.	+	++	+
	*Barathritesiris* gen. inc.	++		
	*Bassozetus* gen. inc.	+	++	
	*Spectrunculuscrassus* sp. inc.		++	+
	*Spectrunculusgrandis* sp. inc.	+	++	
	*Xyelacybamyersi* gen. inc.			++
	Octacnemidae gen. indet.	+		
	*Culeolus* spp. indet			++
	*Bathyrajatunae* sp. inc.			++
**Cnidaria**	Cnidaria cl. indet.		+	
	Spirularia fam. indet.	+		
	Actiniaria fam. indet. (DZMB_2021_0017)	+	+	
	Actiniaria fam. indet. (DZMB_2021_0018)		++	
	Actiniaria fam. indet. (DZMB_2021_0020)		+	
	Actiniaria fam. indet. (DZMB_2021_0022)		+	
	Actinostolidae gen. indet.			++
	Actinostola sp. indet. (DZMB_2021_0028)		++	+
	*Bathyphellia* sp. indet. (DZMB_2021_0032)		++	
	*Bathyphellia* sp. indet. (DZMB_2021_0033)		++	
	Alcyonacea*Anthomastus* gen. inc.		+	
	*Iridogorgiamagnispiralis* sp. inc.		+	
	Clavulariidae gen. indet. (DZMB_2021_0037)	+		
	Clavulariidae fam. inc. (DZMB_2021_0038)	+		
	Isididae gen. indet. (DZMB_2021_0042)		+	
	Isididae fam. inc. (DZMB_2021_0044)		+	
	Isididae*Acanella* gen. inc.		+	
	Isididae*Bathygorgia* gen. inc.		+	+
	Isididae*Jasonisis* gen. inc.		+	
	Isididae*Keratoisis* gen. inc. (DZMB_2021_0046)			+
	Isididae*Lepidisis* gen. inc.		+	
	*Lepidisis* spp. indet.		++	
	Paragorgiidae fam. inc.		+	
	Primnoidae gen. indet. (DZMB_2021_0049)		++	++
	*Heteropathes* sp. indet.		+	
	*Heteropathesamericana* sp. inc.		+	
	*Bathypathespatula* sp. inc.		+	+
	*Schizopathes* spp. indet.		+	
	Pennatulacea fam. indet (DZMB_2021_0053)	+		
	*Umbellula* sp. indet. (DZMB_2021_0054)	+		
	*Umbellula* sp. indet. (DZMB_2021_0055)			+
	*Epizoanthus* sp. indet.	++		
	Hydrozoa ord. indet. (DZMB_2021_0065)			++
	SiphonophoraeRhodaliidae*Thermopalia* gen. inc.			+
**Echinodermata**	*Hymenodiscus* gen. inc.		++	+
	Freyellidae fam. inc.	++	++	
	*Freyastera* gen. inc.	++	++	++
	*Freyella* gen. inc.	++	++	+
	*Styracaster* gen. inc.	++		
	*Henricia* gen. inc.	+		+
	Goniasteridae gen. indet. (DZMB_2021_0066)	+	++	
	Goniasteridae gen. indet. (DZMB_2021_0067)	++		
	*Evoplosoma* gen. inc.		++	
	*Lydiasterjohannae* sp. inc.	+		
	Solasteridae fam. inc.		+	
	*Asthenactis* gen. inc.		+	
	*Hymenaster* sp. indet.		+	
	*Pteraster* gen. inc.		+	
	Antedonidae gen. indet. (DZMB_2021_0068)	++	++	
	Antedonidae fam. inc. (DZMB_2021_0069)	++	++	
	cf. *Bathymetra* sp.	+	+	
	*Pentametrocrinus* sp. indet.	++	++	
	Hyocrinidae gen. indet.		++	
	Irregularia infracl. inc.		+	
	Cidaroida fam. indet.			+
	*Hapalosoma* sp. indet.	++	+	
	*Salenocidaris* sp. indet.		++	
	Elpidiidae gen. indet. (DZMB_2021_0070)	+	++	
	Elpidiidae gen. indet. (DZMB_2021_0071)			+
	Elpidiidae gen. indet. (DZMB_2021_0072)		++	
	*Peniagonepurpurea*	+		
	Laetmogonidae sp. indet.	++	++	
	*Enypniasteseximia*	++		
	*Benthodytes* sp. indet.	++	+	+
	*Benthothuria* gen. inc.		+	
	*Pseudostichopus* gen. inc. (DZMB_2021_0073)		+	
	*Pseudostichopus* sp. indet. (DZMB_2021_0074)		++	
	*Oneirophanta* sp. indet.	++		
	Synallactidae gen. indet. (DZMB_2021_0075)	++	+++	
	Synallactidae gen. indet. (DZMB_2021_0076)	+++	++	++
	Synallactidae gen. indet. (DZMB_2021_0077)		++	++
	Synallactidae gen. indet. (DZMB_2021_0078)		++	
	Synallactidae fam. inc. (DZMB_2021_0079)	+		
	*Synallactes* sp. indet.	++	++	
	Amphilepidida ord. inc.			+
	*Asteronyx* gen. inc.		++	
	Ophiacanthida ord. inc.		++	+
	*Ophiophyllumpetilum* sp. inc.		+	
	*Ophiosphalmaarmigerum* sp. inc.	++	++	++
**Hemichordata**	Torquaratoridae fam. inc.		+	
**Mollusca**	*Bathypolypus* sp. indet.		++	
	*Cirroteuthis* sp. indet.	+	+	++
	*Grimpoteuthis* gen. inc.			+
	*Magnapinna* sp. indet.		+	
	*Speculator* gen. inc.	++		
	Scaphopoda ord. indet.	++	+	
**Porifera**	*Paleodictyonnodosum*	+++	+++	
**Foraminifera**	Monothalamea ord. indet. (DZMB_2021_0080)			++
	Monothalamea ord. indet. (DZMB_2021_0081)	++	++	
	Monothalamea ord. indet. (DZMB_2021_0082)	+++		
	*Luffammina* gen. inc.	+++	+++	
	*Psammina* gen. inc. (DZMB_2021_0083)	+++	+	+
	*Psammina* gen. inc. (DZMB_2021_0084)		+++	
	*Stannoma* gen. inc.	+		
